# Supplementary material for: Stereoselective C–O silylation and stannylation of alkenyl acetates
Source: Nat Commun. 2023 Mar 15;14:1454. doi: 10.1038/s41467-023-37192-7 (PMC10017796; doi:10.1038/s41467-023-37192-7)
Supplement: Supplementary file 1 — Supplementary Information [file 41467_2023_37192_MOESM1_ESM.pdf]

Supplementary Information

for

Stereoselective C–O Silylation and Stannylation of Alkenyl Acetates

*Ying Hu,[1] Jiali Peng,[2],[3] Binjing Hu,[1] Jixin Wang,[1] Jing Jing,[1] Jie Lin,[1] Xingchen Liu,[1] Xiaotian Qi[2] and Jie Li[1]**

[1] Key Laboratory of Organic Synthesis of Jiangsu Province, Suzhou Key Laboratory of Pathogen Bioscience and Anti-infective Medicine, College of Chemistry, Chemical Engineering and Materials Science, Soochow University, Ren-Ai Road 199, 215123 Suzhou, P. R. China

[2] Engineering Research Center of Organosilicon Compounds & Materials, Ministry of Education, College of Chemistry and Molecular Sciences, Wuhan University, Wuhan, Hubei 430072, P. R. China.

[3] School of Chemistry and Chemical Engineering, Henan Institute of Science and Technology, Xinxiang Henan, 453003, P. R. China

* *E-mail:* jjackli@suda.edu.cn

Contents

Supplementary Methods··········2

1.1 General Remarks··········2

Supplementary Discussion··········3

2.1 Optimization for the *Cobalt-Catalyzed Silylation of Alkenyl Acetate* ··········3

2.2 Optimization for the *Cobalt-Catalyzed Stannylation of Alkenyl Acetate* ··········4

2.3 Additional Experiments··········5

2.4 Representative Procedures··········10

2.5 Characterization Data of Alkenyl Acetates **3**··········18

2.6 Characterization Data of Products **4–96**··········33

2.7 Late-Stage Modifications of Alkenyl Silanes and Stannanes··········68

2.8 Characterization Data of Products **97–110**··········68

Supplementary Notes··········75

3.1 NMR Spectra··········75

Supplementary References··········227

Supplementary Methods

1.1 General Remarks

Unless otherwise indicated, all reactions were carried out with magnetic stirring and in flame-dried glassware under nitrogen. Syringes used to transfer reagents and solvents were purged with N_2 prior to use. The following starting materials were synthesized according to previously described methods: ketoesters,[1-8] esters S3as-S3av,[9] alkenyl acetates.[10-13] Other chemicals were obtained from commercial sources and were used without further purification. Yields refer to isolated compounds, estimated to be > 95% pure as determined by ^{1}H-NMR and GC-analysis. Reactions were monitored by gas chromatography (GC and GC-MS) or thin layer chromatography (TLC). TLC were performed using aluminum plates covered with SiO_2 (Merck 60, F-254) and visualized by UV detection. Purification *via* column chromatography was performed using Merck silica gel 60 (40–63 mm 230–400 mesh ASTM from Merck). THF was continuously refluxed and freshly distilled from sodium benzophenone ketyl under nitrogen. NMR spectra were recorded in $CDCl_3$ and chemical shifts (δ) are reported in parts per million (ppm). Mass spectra and high resolution mass spectra (HR-MS) were recorded using electro ionization (EI) except where otherwise noted. GCs were recorded on machines of the type Hewlett-Packard 6890 (Hewlett Packard, 5% phenylmethylpolysiloxane; length: 15 m, diameter: 0.25 mm; film thickness: 0.25 μm).

Supplementary Discussion

Supplementary Table 1. Optimization for Cobalt-Catalyzed Silylation of Alkenyl Acetate 3a.

2.1 Optimization for the *Cobalt-Catalyzed Silylation of Alkenyl Acetates*

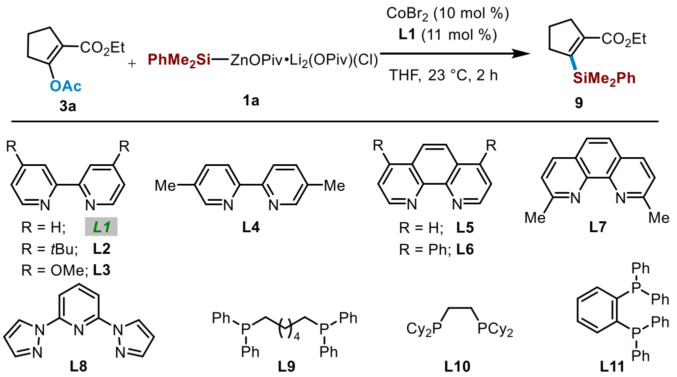

entry	modified conditions	yield (%)[b]
1	none	81
2	**L2** instead of **L1**	40
3	**L3** instead of **L1**	Trace
4	**L4** instead of **L1**	80
5	**L5** instead of **L1**	Trace
6	**L7** instead of **L1**	Trace
7	**L11** instead of **L1**	Trace
8	PhMe or NMP or MeCN	0-20
9	Under 0 °C	57
10	CoI2 instead of $CoBr_2$ Under 0 °C	55
11	CoCl2 instead of $CoBr_2$	62
12	Co(acac)2 instead of $CoBr_2$	33
13	NiCl2 or FeCl2 or CrCl2	0-8
14	w/o [Co]	0

Reaction conditions: **3a** (0.25 mmol, 1.0 equiv), **1a** (0.5 mmol, 2 equiv), $CoBr_2$ (10 mol %), **L1** (11 mol %), THF (1.5 mL), @ 23°C, 2 h. [b] Isolated yields.

Supplementary Table 2. Optimization for Cobalt-Catalyzed Stannylation of Alkenyl Acetate 5a.

2.2 Optimization for the *Cobalt-Catalyzed Stannylation of Alkenyl Acetates*

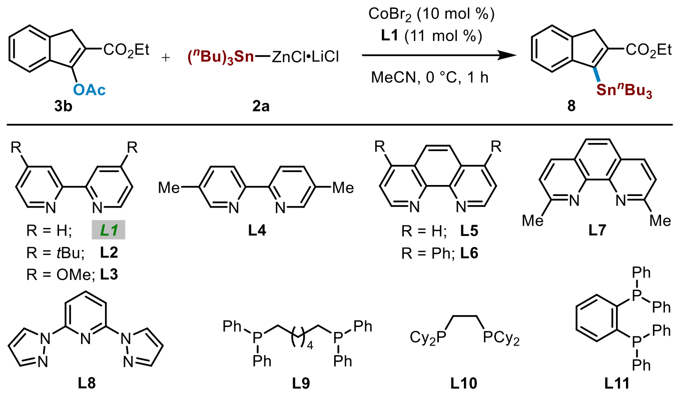

entry	modified conditions	yield (%)[a]
1	none	20
2	**L2** instead of **L1**	12
3	**L3** instead of **L1**	Trace
4	**L4** instead of **L1**	18
5	**L5** instead of **L1**	18
6	**L6** instead of **L1**	15
7	**L7** instead of **L1**	0
8	**L8** instead of **L1**	trace
9	**L9** instead of **L1**	14
10	**L10** instead of **L1**	18
11	**L11** instead of **L1**	20
12	THF, DMF, dioxane instead of MeCN	0
13	$NiCl_2$, $FeCl_2$, or $CrCl_2$ instead of $CoBr_2$	0–8
14	under -20 °C (or -30 °C)	53 (0r 0)
15	25 min; under -20 °C	54
**16**	**15 min; under -20 °C**	**66** (21)[b]

Reaction conditions: **3b** (0.25 mmol, 1.0 equiv), **2a** (0.325 mmol, 1.3 equiv), $CoBr_2$ (10 mol %), L1 (11 mol %), MeCN (1.5 mL), @ 0 °C, 1 h. [a] Isolated yields. [b] The number given in parentheses is the isolated yield of **6**.

2.3 Additional Experiments

(**a**) *radical-clock experiment with radical scavengers* ***82***

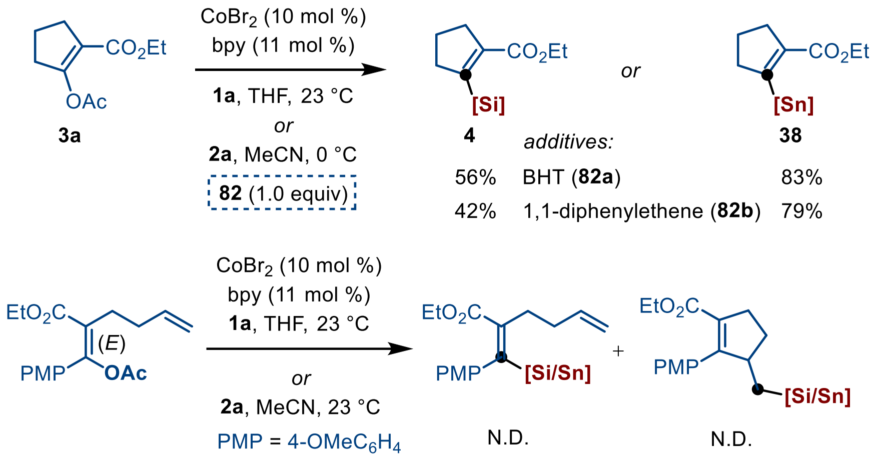

(**b**) *Co-catalyzed cross-couplings with Z- & E-alkenyl acetate*

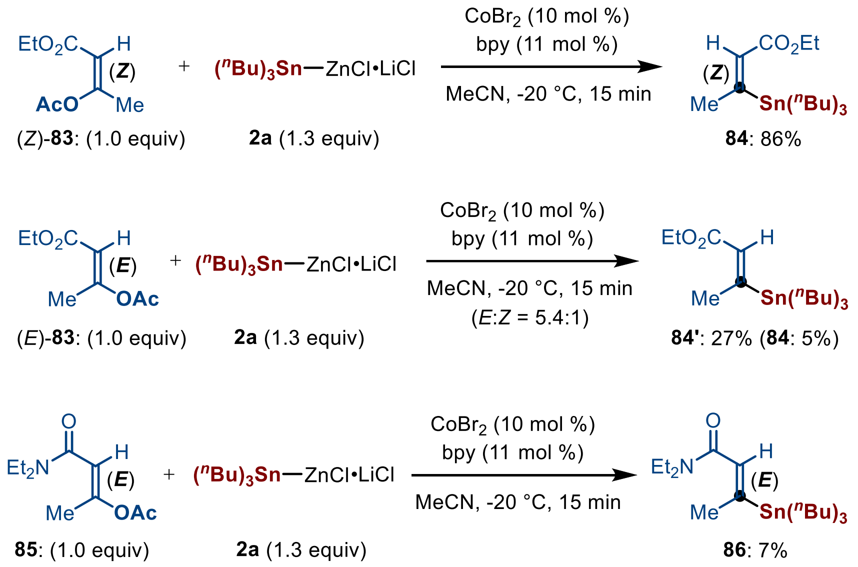

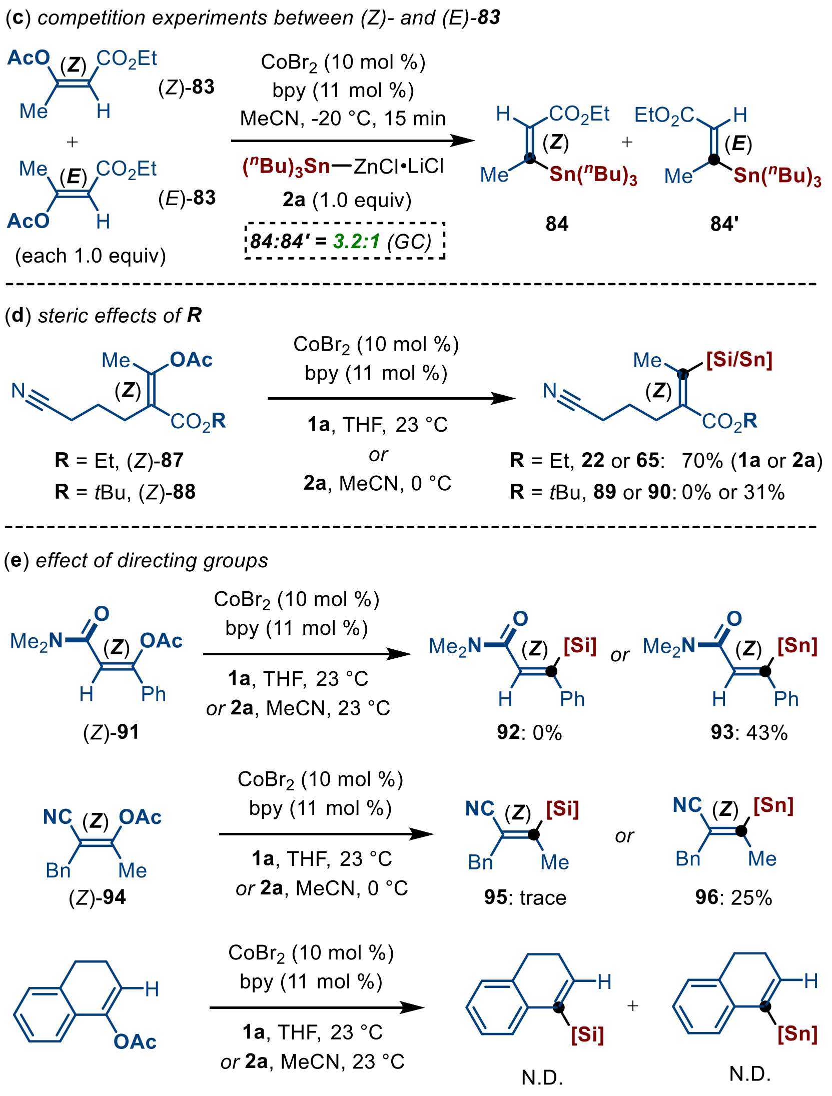

Supplementary Figure 1. **Mechanistic Studies for Cobalt-Catalyzed Stereoselective C–O Bond Silylation and Stannylation**. **a)** Control experiments with radical scavengers. **b)** Investigation of cobalt-catalyzed stereoselective C–O bond cleavage. **c)** Competition experiments of stereodefined alkenyl acetates. **d)** Steric effects of different esters. **e)** Chelation-assistance of directing groups.

Procedure for Figure 1a:

To a flame-dried 25 ml schlenk tube charged with stir bar, $CoBr_2$ (5.5 mg, 10 mol %) and 2,2'-bipyridyl (4.0 mg, 10 mol %) were added. After evacuated and backfilled nitrogen three times, the anhydrous THF (0.5 mL) was added via a syringe, followed by the addition of **3a** (0.25

mmol, 1.0 equiv) and **1,1-Diphenylethylene** or **BHT** (0.25 mmol, 1.0 equiv). Then the **PhMe$_2$Si-ZnOPiv** (0.5 mmol, 2 equiv) in THF (1 mL) was added via a syringe and the reaction was stirred at 23 °C for 2 h.

To a flame-dried 25 ml schlenk tube charged with stir bar, $CoBr_2$ (5.5 mg, 10 mol %), 2,2'-bipyridyl (4.0 mg, 10 mol %) were added. After evacuated and backfilled nitrogen three times, the anhydrous MeCN (0.5 mL) was added via a syringe, followed by the addition of **3a** (0.25 mmol, 1.0 equiv) and **1,1-Diphenylethylene** or **BHT** (0.25 mmol, 1.0 equiv). Then the **(^{n}Bu)$_3$Sn–ZnCl•LiCl** (0.325 mmol, 1.3 equiv) in MeCN (1 mL) was added via a syringe and the reaction was stirred at 0 °C for 1 h.

To a flame-dried 25 ml schlenk tube charged with stir bar, $CoBr_2$ (5.5 mg, 10 mol %) and 2,2'-bipyridyl (4.0 mg, 10 mol %) were added. After evacuated and backfilled nitrogen three times, the anhydrous THF (0.5 mL) was added via a syringe, followed by the addition of corresponding alkenyl acetate (0.25 mmol, 1.0 equiv). Then the **PhMe$_2$Si-ZnOPiv** (0.5 mmol, 2 equiv) in THF (1 mL) was added via a syringe and the reaction was stirred at 23 °C for 1 h.

To a flame-dried 25 ml schlenk tube charged with stir bar, $CoBr_2$ (5.5 mg, 10 mol %), 2,2'-bipyridyl (4.0 mg, 10 mol %) were added. After evacuated and backfilled nitrogen three times, the anhydrous MeCN (0.5 mL) was added via a syringe, followed by the addition of corresponding alkenyl acetate (0.25 mmol, 1.0 equiv). Then the **(^{n}Bu)$_3$Sn–ZnCl•LiCl** (0.325 mmol, 1.3 equiv) in MeCN (1 mL) was added via a syringe and the reaction was stirred at 23 °C for 1 h.

Procedure for Figure 1b:
To a flame-dried 25 ml schlenk tube charged with stir bar, $CoBr_2$ (5.5 mg, 10 mol %), 2,2'-bipyridyl (4.0 mg, 10 mol %) were added. After evacuated and backfilled nitrogen three times, the anhydrous MeCN (0.5 mL) was added via a syringe, followed by the addition of corresponding alkenyl acetates (0.25 mmol, 1.0 equiv). Then the **(^{n}Bu)$_3$Sn–ZnCl•LiCl** (0.325 mmol, 1.3 equiv) in MeCN (1 mL) was added via a syringe and the reaction was stirred at -20 °C for 15 minutes. Then solvent was evaporated in vacuo and the remaining residue was purified by column chromatography on silica gel (petroleum ether/EtOAc) to yield products **84**, **84'**, **86**.

Procedure for Figure 1c:

To a solution of $CoBr_2$ (5.5 mg, 10 mol %), 2,2'-bipyridyl (4.0 mg, 10 mol %) in anhydrous MeCN (0.5 mL) were added (*Z*)-**83** (0.25 mmol, 1.0 equiv) and (*E*)-**83** (0.25 mmol, 1.0 equiv). Then the (^{n}Bu)$_3$Sn–ZnCl•LiCl (0.25 mmol, 1.0 equiv) in MeCN (1 mL) was added and the reaction was stirred at -20 °C for 15 minutes under an atmosphere of N_2. The reaction mixture was analyzed by GC.

Procedure for Figure 1d:

To a flame-dried 25 ml schlenk tube charged with stir bar, $CoBr_2$ (5.5 mg, 10 mol %) and 2,2'-bipyridyl (4.0 mg, 10 mol %) were added. After evacuated and backfilled nitrogen three times, the anhydrous THF (0.5 mL) was added via a syringe, followed by the addition of corresponding alkenyl acetate (0.25 mmol, 1.0 equiv). Then the **PhMe$_2$Si-ZnOPiv** (0.5 mmol, 2 equiv) in THF (1 mL) was added via a syringe and the reaction was stirred at 23 °C for 1 h. Then solvent was evaporated in vacuo and the remaining residue was purified by column chromatography on silica gel (petroleum ether/EtOAc) to yield product **22**.

To a flame-dried 25 ml schlenk tube charged with stir bar, $CoBr_2$ (8.2 mg, 15 mol %), 2,2'-bipyridyl (5.9 mg, 15 mol %) were added. After evacuated and backfilled nitrogen three times, the anhydrous MeCN (0.5 mL) was added via a syringe, followed by the addition of corresponding alkenyl acetate (0.25 mmol, 1.0 equiv). Then the **(^{n}Bu)$_3$Sn–ZnCl•LiCl** (0.325 mmol, 1.3 equiv) in MeCN (1 mL) was added via a syringe and the reaction was stirred at 0 °C for 1 h. Then solvent was evaporated in vacuo and the remaining residue was purified by column chromatography on silica gel (petroleum ether/EtOAc) to yield products **65**, **90**.

Procedure for Figure 1e:

To a flame-dried 25 ml schlenk tube charged with stir bar, $CoBr_2$ (5.5 mg, 10 mol %) and 2,2'-bipyridyl (4.0 mg, 10 mol %) were added. After evacuated and backfilled nitrogen three times, the anhydrous THF (0.5 mL) was added via a syringe, followed by the addition of (*Z*)-**91** (0.25 mmol, 1.0 equiv). Then the **PhMe$_2$Si-ZnOPiv** (0.5 mmol, 2 equiv) in THF (1 mL) was added via a syringe and the reaction was stirred at 23 °C for 1 h.

To a flame-dried 25 ml schlenk tube charged with stir bar, $CoBr_2$ (5.5 mg, 10 mol %), 2,2'-

bipyridyl (4.0 mg, 10 mol %) were added. After evacuated and backfilled nitrogen three times, the anhydrous MeCN (0.5 mL) was added via a syringe, followed by the addition of (*Z*)-**91** (0.25 mmol, 1.0 equiv). Then the **(^{n}Bu)$_3$Sn–ZnCl•LiCl** (0.325 mmol, 1.3 equiv) in MeCN (1 mL) was added via a syringe and the reaction was stirred at 23 °C for 1 h. Then solvent was evaporated in vacuo and the remaining residue was purified by column chromatography on silica gel (petroleum ether/EtOAc) to yield product **93**.

To a flame-dried 25 ml schlenk tube charged with stir bar, $CoBr_2$ (5.5 mg, 10 mol %), 2,2'-bipyridyl (4.0 mg, 10 mol %) were added. After evacuated and backfilled nitrogen three times, the anhydrous THF (0.5 mL) was added via a syringe, followed by the addition of (*Z*)**-94** (0.25 mmol, 1.0 equiv). Then the **PhMe$_2$Si-ZnOPiv** (0.5 mmol, 2 equiv) in THF (1 mL) was added via a syringe and the reaction was stirred at 23 °C for 1 h.

To a flame-dried 25 ml schlenk tube charged with stir bar, $CoBr_2$ (8.2 mg, 15 mol %), 2,2'-bipyridyl (5.9 mg, 15 mol %) were added. After evacuated and backfilled nitrogen three times, the anhydrous MeCN (0.5 mL) was added via a syringe, followed by the addition of (*Z*)**-94** (0.25 mmol, 1.0 equiv). Then the **(^{n}Bu)$_3$Sn–ZnCl•LiCl** (0.325 mmol, 1.3 equiv) in MeCN (1 mL) was added via a syringe and the reaction was stirred at 0 °C for 1 h. The solvent was evaporated in vacuo and the remaining residue was purified by column chromatography on silica gel (petroleum ether/EtOAc) to yield product **96**.

To a flame-dried 25 ml schlenk tube charged with stir bar, $CoBr_2$ (5.5 mg, 10 mol %), 2,2'-bipyridyl (4.0 mg, 10 mol %) were added. After evacuated and backfilled nitrogen three times, the anhydrous THF (0.5 mL) was added via a syringe, followed by the addition of corresponding alkenyl acetate (0.25 mmol, 1.0 equiv). Then the **PhMe$_2$Si-ZnOPiv** (0.5 mmol, 2 equiv) in THF (1 mL) was added via a syringe and the reaction was stirred at 23 °C for 3 h.

To a flame-dried 25 ml schlenk tube charged with stir bar, $CoBr_2$ (5.5 mg, 10 mol %), 2,2'-bipyridyl (4.0 mg, 10 mol %) were added. After evacuated and backfilled nitrogen three times, the anhydrous MeCN (0.5 mL) was added via a syringe, followed by the addition of corresponding alkenyl acetate (0.25 mmol, 1.0 equiv). Then the **(^{n}Bu)$_3$Sn–ZnCl•LiCl** (0.325 mmol, 1.3 equiv) in MeCN (1 mL) was added via a syringe and the reaction was stirred at 23 °C for 3 h.

2.4 Representative Procedures

Typical procedure 1 (TP1) for the preparation of ketoesters:

1. Synthesis of substituted benzoylacetates from aryl ketones:[1]

S**3ac**-S**3af,** S**3ah**-S**3al** were prepared in the following manner: NaH (40 mmol, 2.0 eq, 60% in mineral oil) was added in 50 mL THF to form a suspension. And then commercial available ketone (20 mmol, 1.0 eq) was added to the mixture and stirred at rt for 10 min. Then diethyl carbonate (40 mmol, 2.0 eq) was added to the solution at rt and kept stirring at reflux conditions for 2-3 h. Upon completion as indicated by TLC, the reaction was quenched with saturated aqueous NH_4HCO_3 and followed by the addition of acetic acid (1 M) at rt. The reaction mixture was then extracted with ethyl acetate, washed with brine, dried over Na_2SO_4, filtered and concentrated to give the crude product, which was purified by column chromatography on silica gel (petroleum ether/EtOAc) to give S**3ac**-S**3af,** S**3ah**-S**3al**.

Another syhthesis method:[2]

S**3ag** was prepared in the following manner: NaH (20 mmol, 2.0 eq, 60% in mineral oil) was added in 30 mL THF to form a suspension. And diethyl carbonate (20 mmol, 2 eq) was then added to the suspension at rt and subsequently the ketone (10 mmol, 1.0 eq) was added dropwise. The reaction mixture was allowed to stir at rt until hydrogen gas evolution ceased and then heated to reflux. Upon completion as indicated by TLC, the reaction mixture was cooled to 0 °C and quenched with aqueous NH_4Cl. The reaction mixture was then extracted with ethyl acetate, washed with brine, dried over Na_2SO_4, filtered and concentrated to give the crude product, which was purified by column chromatography on silica gel (petroleum ether/EtOAc) to give S**3ag**.

According to Stubbing et al,[3] ethyl 3-(4-hydroxyphenyl)-3-oxopropanoate was prepared in the following manner: A solution of 4'-hydroxyacetophenone (10 mmol, 1.0 eq) in THF (26 mL) was added dropwise to a solution of LiHMDS (1 M in THF, 30 mmol, 3.0 eq) at -78 °C. The mixture was allowed to stir at this temperature for 2 h, then a solution of diethyl carbonate (11 mmol, 1.1 eq) in THF (10 mL) was added quickly. The mixture was allowed to slowly warm to rt and stirred overnight. The mixture was then poured onto a mixture of ice and concd HCl. The resulting solution was extracted with DCM and the combined organic extracts was washed with

water, dried over Na_2SO_4, filtered and concentrated to give the crude product, which was purified *via* flash column chromatography on silica gel (petroleum ether/EtOAc) to give ethyl 3-(4-hydroxyphenyl)-3-oxopropanoate.

2. Synthesis of cyclic ketoesters:[4]

S**3x**, S**3y** were prepared in the following manner: NaH (20 mmol, 2.0 eq, 60% in mineral oil) was added to THF (6 mL) followed by the addition of diethyl carbonate (20 mmol, 2.0 eq) to form a suspension. Then a solution of the starting material (10 mmol, 1.0 eq) in THF (20 mL) was added to the reaction mixture dropwise. The mixture was stirred at 75 ºC for 16 h, then extracted with ethyl acetate, dried over Na_2SO_4, filtered and concentrated to give the crude product, which was purified by column chromatography on silica gel (petroleum ether/EtOAc) to give the cyclic ketoesters S**3x**, S**3y**.

3. Synthesis of α-alkyl substituted ketoesters from ethyl acetoacetate:[5]

S**3c-S3u,** S**3an** and S**3ap-S3ar** were prepared in the following manner: To a suspension of *t*-BuOK (22 mmol, 1.1 eq) in THF (40 mL) was added ethyl acetoacetate or tert-butyl acetoacetate (in case of S**3ay**) (20 mmol, 1.0 eq) at 0 °C. The resulting clear solution was stirred at 0 °C for 30 min, and then alkyl bromide or alkyl iodide (24 mmol, 1.2 eq) in THF (10 mL) was added to the solution. After heating under reflux conditions for 12 h, the reaction was quenched with saturated aqueous NH_4Cl. Then the aqueous layer was extracted with ethyl acetate. The combined organic layers were washed with brine, then dried over Na_2SO_4, filtered and concentrated to give crude product, which was purified by column chromatography on silica gel (petroleum ether/EtOAc) to give α- alkyl substituted ketoesters S**3c-S3u,** S**3an** and S**3ap-S3ar**.

4. Synthesis of α-alkyl substituted benzoylacetates:[6]

S(*E*)**-82** was prepared in the following manner: A solution of ethyl 3-(4-methoxyphenyl)-3-oxopropanoate (15 mmol, 1.0 eq) in dry DMF (10 mL) was added dropwise to a suspension of NaH (18 mmol, 1.2 eq, 60% in mineral oil) in dry DMF (10 mL). The mixture was stirred for 1 h at rt. Then 4-bromobut-1-ene (16.5 mmol, 1.1 eq) in dry DMF (4 mL) was added dropwise, and the mixture was stirred for 20 h at rt. The reaction was quenched with water and neutralized with 10% aqueous HCl, extracted with ethyl actetate, dried over Na_2SO_4, filtered and concentrated to give the crude product, which was purified by column chromatography on silica gel (petroleum ether/EtOAc) to give S(*E*)**-82**.

5. Synthesis of α-aryl substituted ethyl 3-oxooctanoate:[7]

S**3v** was slightly modified by Chegaev et al. To the solution of ethyl phenylacetate (10 mmol, 1.0 eq) in THF (20 mL), 16 mL of 1.3 M LiHMDS solution in THF was added dropwise at -70 °C. Then the solution was stirred at -70 °C for 15 min. The solution of hexanoyl chloride (10 mmol, 1.0 eq) in THF (20 mL) was added dropwise and the reaction was stirred at -70 °C for 2 h. The reaction mixture was quenched with saturated aqueous NH_4Cl and then extracted with ethyl acetate, washed with water and brine, dried over Na_2SO_4, filtered and concentrated to give the crude product, which was purified by column chromatography on silica gel (petroleum ether/EtOAc) to give S**3v**.

6. Synthesis of ethyl 4-oxochromane-3-carboxylate S3z:[8]

S**3z** was slightly modified by Kenny et al. To a solution of 4-chromanone (10 mmol, 1.0 eq) in THF (20 mL) was added a solution of LiHMDS (15 mmol, 1.5 eq, 1.3 M in THF) dropwise at -78 °C and the mixture was stirred for 30 min. Ethyl cyanoformate (20 mmol, 2.0 eq) in THF (20 mL) was added dropwise and the reaction was stirred at -78 °C for 1 h. The mixture was allowed to warm to rt and quenched by the addition of aqueous NH_4Cl and water. The combined organic phases were washed with brine, dried over Na_2SO_4 and concentrated. Flash column chromatography on silica gel (petroleum ether/EtOAc) afforded S**3z**.

Typical procedure 2 (TP2) for the synthesis of esters S**3as**-S**3av:**[9]

Take S**3at** for example: To a round-bottom flask were added (S)-(+)-ibuprofen (4 mmol, 1.0 eq), dicyclohexylcarbodiimide (6 mmol, 1.5 eq), and DCM (30 mL). Then ethyl 3-(4-hydroxyphenyl)-3-oxopropanoate (4 mmol, 1.0 eq) and 4-dimethylaminopyridine (0.4 mmol, 0.1 eq) were added to the mixture. The reaction mixture was stirred at rt for 24 h and then filtered. The filtrate was concentrated and purified by flash column chromatography on silica gel (petroleum ether/EtOAc) to give S**3at**.

Typical procedure 3 (TP3) for the synthesis of alkenyl acetates 3a-3av: [10]

To a solution of ketoester (10 mmol, 1.0 eq) in *iso*-propenyl acetate (10 mL) was added *p*-TSA H_2O (1 mmol, 0.1 eq). The resulting mixture was heated under reflux conditions (130 ℃) for 18 hours (As for **3as-3av**, the resulting mixture were refluxing at 110 ℃). The mixture was allowed to cool to rt and the remaining *iso*-propenyl acetate was removed under reduced

pressure. The brown oily residue was extracted with ethyl acetate and washed with water. Afterwards the organic phase was dried over Na_2SO_4, filtered and the solvent was removed under reduced pressure. The remaining residue was purified by column chromatography on silica gel (petroleum ether /EtOAc) to yield alkenyl acetates **3a-3av**.

Alkenyl Acetates:

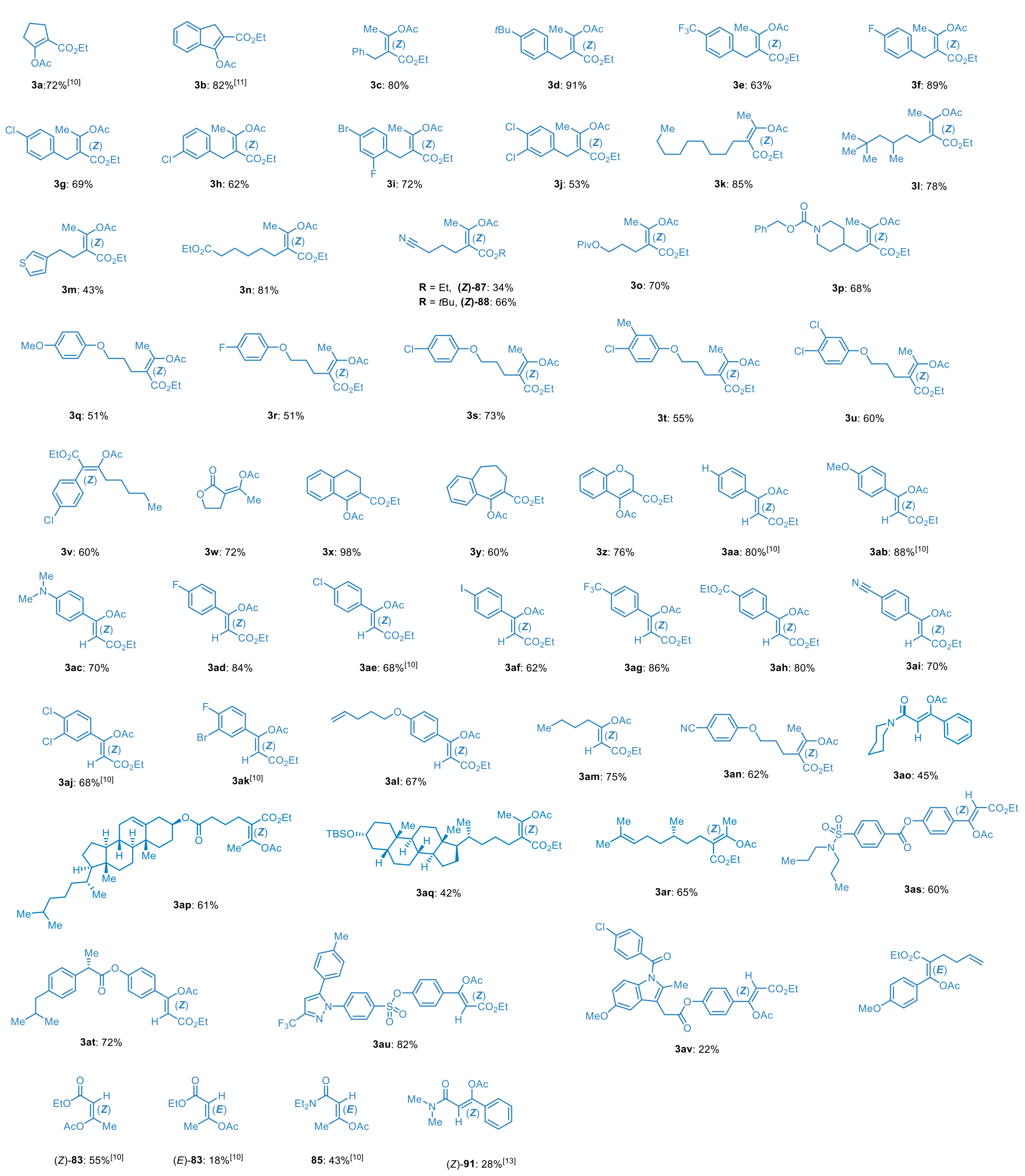

Supplementary Figure 2. **List of the prepared alkenyl acetates**.

Preparation of Zn(OPiv)2:

Pivalic acid (20.4 g, 22.6 mL, 200 mmol) was placed in a dry and argon-flushed 500 mL three-necked roundbottom flask, equipped with a magnetic stirring bar, a septum and a pressure equalizer, and was dissolved in dry THF (120 mL). The mixture was cooled to 0 °C, and a

solution of Et_2Zn (13.0 g, 10.8 mL, 105 mmol) in dry THF (120 mL) was added over a period of 30 min under vigorous stirring. Then, the ice-bath was removed and stirring was continued at 25 °C for one additional hour at which point bubbling has ceased (a thick slurry was formed). The solvent was removed in vacuo and the solid residue was dried for at least 4 h longer. $Zn(OPiv)_2$ was obtained in quantitative yield, as a puffy amorphous white solid.

Preparation of $PhMe_2Si$-ZnOPiv and (^{n}Bu)$_3$Sn–ZnCl•LiCl:

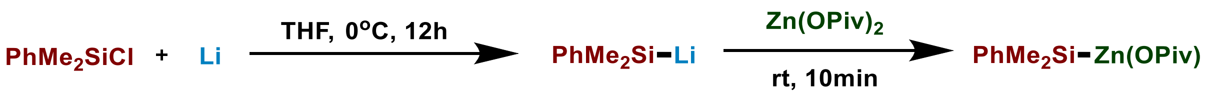

Supplementary Figure 3. Preparation of solid silylzinc pivalate.

To a 50-mL two-necked round-bottomed flask equipped with a magnetic stirring bar, lithium clippings (174 mg, 25 mmol) was added. After evacuated and backfilled nitrogen three times. Dry THF (10 ml) was added via a syringe. Then Chlorodimethylphenylsilane (10 mmol, 1.0 equiv) was added dropwise at 0 °C (use the Low temperature constant temperature stirring reaction bath) and the mixture was stirred at same temperature for 12 h. The silyllithium was titrated against iodine according to Kofron's method. Next, this solution was added via syringe into a 50-mL two-necked round-bottomed flask equipped with a magnetic stirring and $Zn(OPiv)_2$ (1.5 equiv with respect to titrated silyllithium) at rt, and the reaction mixture was stirred at rt for 10 min. The **$PhMe_2Si$-ZnOPiv (1a)** (routinely formed as an ~0.55 M solution) was titrated using Knochel's method.

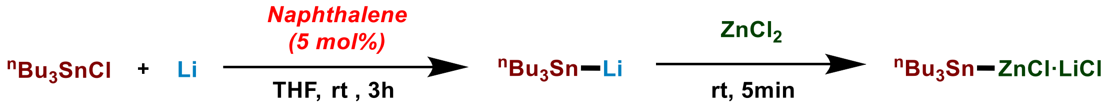

Supplementary Figure 4. Preparation of stannylzinc chloride.

To a 50-mL two-necked round-bottomed flask equipped with a magnetic stirring bar, lithium clippings (84 mg, 12 mmol) and naphthalene (26 mg, 0.2 mmol) were added. After evacuated and backfilled nitrogen three times. Dry THF (8 mL) was added via a syringe. The resulting mixture was stirred at rt for 2 minutes. Then tributyltin chloride (1.1 mL, 4 mmol) was added and the mixture was stirred at rt for 3 h. The stannyllithium was titrated against iodine according to Kofron's method (~0.25 M). A portion of 1.3 mL of the above solution (0.325 mmol) was added to a stirred solution of $ZnCl_2$ (0.5 mL, 1 M solution in THF). After the yellow reaction was stirred at rt for 5 minutes. The **(^{n}Bu)$_3$Sn–ZnCl•LiCl (2a)** (routinely formed as an ~0.25 M

solution) was titrated using Knochel's method. Then the solvent was removed in vacuo and the liquid residue (**2a**) was obtained. *CAUTION*: *The organotin reagents are toxic and volatile.These reation shoule be performed in a well-ventilated fume hood. The residual organotin reagents should be added to the saturated KF solution, then celite was added before the above solution was stirred for 1h.*

Typical procedure 4 (TP4) for the cobalt-catalyzed silylation of alkenyl acetates:

To a flame-dried 25 ml schlenk tube charged with stir bar, $CoBr_2$ (5.5 mg, 10 mol %) and 2,2'-bipyridyl (4.0 mg, 10 mol %) were added. After evacuated and backfilled nitrogen three times, the anhydrous THF (0.5 mL) was added via a syringe, followed by the addition of **alkenyl acetates 3** (0.25 mmol, 1.0 equiv). Then the **$PhMe_2Si$-ZnOPiv** (0.9 mL, 0.5 mmol, 2 equiv) in THF (1 mL) was added via a syringe and the reaction was stirred at rt for 2 h. Then solvent was evaporated in vacuo and the remaining residue was purified by column chromatography on silica gel (petroleum ether/EtOAc) to yield products **4, 21-22, 24, 26-31**.

Typical procedure 5 (TP5) for the cobalt-catalyzed silylation of alkenyl acetates:

To a flame-dried 25 ml schlenk tube charged with stir bar, $CoBr_2$·5,5'-dimethyl-2,2'-bipyridine (10 mg, 10 mol %) were added. After evacuated and backfilled nitrogen three times, the anhydrous THF (0.5 mL) was added via a syringe, followed by the addition of **alkenyl acetates 3** (0.25 mmol, 1.0 equiv). Then the **$PhMe_2Si$-ZnOPiv** (0.9 mL, 0.5 mmol, 2 equiv) in THF (1 mL) was added via a syringe and the reaction was stirred at rt for 2 h. Then solvent was evaporated in vacuo and the remaining residue was purified by column chromatography on silica gel (petroleum ether/EtOAc) to yield products **9-20, 23, 25, 32, 73-76.**

Typical procedure 6 (TP6) for the cobalt-catalyzed stannylation of alkenyl acetates:

To a flame-dried 25 ml schlenk tube charged with stir bar, $CoBr_2$ (5.5 mg, 10 mol %), 2,2'-bipyridyl (4.0 mg, 10 mol %) were added. After evacuated and backfilled nitrogen three times, the anhydrous MeCN (0.5 mL) was added via a syringe, followed by the addition of **alkenyl acetates 3** (0.25 mmol, 1.0 equiv). Then the **$(^{n}Bu)_3Sn$–ZnCl•LiCl** (1.3 mL, 0.325 mmol, 1.3 equiv) in MeCN (1 mL) was added via a syringe at -20 °C (use the Low temperature constant temperature stirring reaction bath) and the reaction was stirred at -20 °C for 15 minutes. Then solvent was evaporated in vacuo and the remaining residue was purified by column chromatography on silica gel (petroleum ether/EtOAc) to yield products **7**, **40-45, 47, 49-52,**

78-81, 84, **84’**,**86**.

Typical procedure 7 (TP7) for the cobalt-catalyzed stannylation of alkenyl acetates:

To a flame-dried 25 ml schlenk tube charged with stir bar, $CoBr_2$ (8.2 mg, 15 mol %), 2,2'-bipyridyl (5.9 mg, 15 mol %) were added. After evacuated and backfilled nitrogen three times, the anhydrous MeCN (0.5 mL) was added via a syringe, followed by the addition of **alkenyl acetates 3** (0.25 mmol, 1.0 equiv). Then the **(^{n}Bu)$_3$Sn–ZnCl•LiCl** (1.3 mL, 0.325 mmol, 1.3 equiv) in MeCN (1 mL) was added via a syringe at 0 °C (use the Low temperature constant temperature stirring reaction bath) and the reaction was stirred at 0 °C for 1 h. Then solvent was evaporated in vacuo and the remaining residue was purified by column chromatography on silica gel (petroleum ether/EtOAc) to yield products **35-39, 46, 48, 53-71, 77, 96**. *CAUTION*: *These tri- or tetraorganotin compounds should be placed in the well-ventilated fume hood.*

Typical procedure 8 (**TP8**) **for the MKS Reaction:**

To a flame-dried 25 ml schlenk tube charged with stir bar, $Pd(PPh_3)_4$ (6 mg, 5 mol %), CuI (9.5 mg, 50 mol %) were added. After evacuated and backfilled nitrogen three times, the anhydrous DMF (0.5 mL) was added via a syringe, followed by the addition of alkenyl stannanes (0.1 mmol, 1.0 equiv) and aryl or heteroaryl halides (0.12 mmol, 1.2 equiv). The reaction was stirred at 23 °C for 16 h. Then solvent was evaporated in vacuo and the remaining residue was purified by column chromatography on silica gel (petroleum ether/EtOAc) to yield products **99-108**.

Typical procedure 9 (**TP9**) **for the reduction of the ester:**

To a solution of **ester** (1.0 equiv) in THF (1.5 mL), a solution of diisobutylaluminium hydride (3.0 equiv, 1.5 M in toluene) was added at –78 °C under nitrogen. After stirring for 20 min at 0 °C, diethyl ether and water were successively added at 0 °C. After stirring for 2 h, the reaction mixture was extracted with ethyl acetate, washed with water and brine, dried over Na_2SO_4, filtered and concentrated. The remaining residue was purified by column chromatography on silica gel (petroleum ether/EtOAc) to yield product **8**.

Typical procedure 10 (**TP10**) **for the reduction of the lactones:**

To a suspended solution of **$LiAlH_4$** (1.5 equiv) in anhydrous Et_2O (1 mL) was added **lactones** (1 equiv) in Et_2O (1 mL) at 0 °C under N_2 atmosphere. After stirring for 1 h, the reaction was quenched with water at 0 °C. Then the reaction was extracted with CH_2Cl_2, washed with water

and brine, dried over Na_2SO_4, filtered and concentrated. The remaining residue was purified by column chromatography on silica gel (petroleum ether/EtOAc) to yield product **33, 72**.

Typical procedure 11 (TP11) for the C—Si bond cleavage:

To a solution of tetrasubstituted alkenyl silanes (1.0 equiv) in CCl_4 (1.5 mL) was added iodine (1.5 equiv). The reaction mixture was stirred at 70 °C for 16 h. After cooling to room temperature, saturated $Na_2S_2O_3$ solution (4 mL) was added and the resulting mixture was extracted with CH_2Cl_2 (15 ml x 3). The organic phase was washed with brine (30 mL), dried over Na_2SO_4, filtered and concentrated. The remaining residue was purified by column chromatography on silica gel (petroleum ether/EtOAc) to yield products **109-110**.

Typical procedure 12 (TP12) for the Hiyama Cross-Coupling:

To a solution of alkenyl silane (1.0 equiv) in THF (1.5 mL) was added NaOH (2 equiv) at rt, then the solution stirred for 5 min. **Ar-I** (1.5 equiv) was added and after 5 min Pd_2dba_3 (2.5 mol%) was added, then the mixture was stirred at 60 °C for 16 h. After cooling to room temperature, the reaction mixture was purified by column chromatography on silica gel (petroleum ether/EtOAc) to yield products **97-98**.

2.5 Characterization Data of Alkenyl Acetates 3

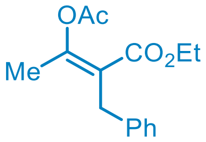

Ethyl (*Z*)-3-acetoxy-2-benzylbut-2-enoate (3c)

The general procedure **TP3** was followed using **ketoester** (3.7 mmol) for 18 h. Purification by column chromatography (petroleum ether/ EtOAc 20:1) yielded **3c** (80%, 775 mg) as a yellow oil. ^{1}H-NMR (400 MHz, $CDCl_3$): δ = 7.30 – 7.16 (m, 5H), 4.08 (q, J = 7.1 Hz, 2H), 3.71 (s, 2H), 2.20 (s, 3H), 2.06 (s, 3H), 1.15 (t, J = 7.1 Hz, 3H). ^{13}C-NMR (100 MHz, $CDCl_3$): δ = 168.9, 165.8, 154.4, 138.7, 128.6, 128.2, 126.4, 119.7, 60.7, 34.4, 21.1, 19.0, 14.2. HR-MS (EI) m/z calcd for $C_{15}H_{18}O_4$ [M+H$^+$] 263.1278, found 263.1280.

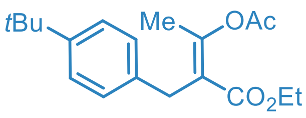

Ethyl (*Z*)-3-acetoxy-2-(4-(tert-butyl)benzyl)but-2-enoate (3d)

The general procedure **TP3** was followed using **ketoester** (3.6 mmol) for 18 h. Purification by column chromatography (petroleum ether/ EtOAc 20:1) yielded **3d** (1.04 g, 91% yield) as a colorless oil. ^{1}H-NMR (400 MHz, $CDCl_3$): δ = 7.30 (d, J = 8.3 Hz, 2H), 7.16 (d, J = 8.2 Hz, 2H), 4.09 (q, J = 7.1 Hz, 2H), 3.68 (s, 2H), 2.19 (s, 3H), 2.06 (s, 3H), 1.29 (s, 9H), 1.15 (t, J = 7.1 Hz, 3H). ^{13}C-NMR (100 MHz, $CDCl_3$): δ = 168.8, 165.8, 154.1, 149.1, 135.5, 127.7, 125.4, 119.8, 60.6, 34.4, 33.8, 31.4, 21.0, 18.9, 14.1. HR-MS (EI) m/z calcd for $C_{19}H_{26}O_4$ [M+H$^+$] 319.1904, found 319.1908.

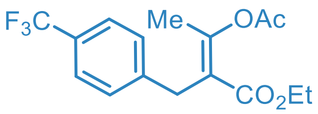

Ethyl (*Z*)-3-acetoxy-2-(4-(trifluoromethyl)benzyl)but-2-enoate (3e)

The general procedure **TP3** was followed using **ketoester** (2.2 mmol) for 18 h. Purification by column chromatography (petroleum ether/ EtOAc 20:1) yielded **3e** (63%, 457 mg) as a colorless oil. ^{1}H-NMR (400 MHz, $CDCl_3$): δ = 7.54 (d, J = 8.1 Hz, 2H), 7.35 (d, J = 8.1 Hz, 2H), 4.09 (q, J = 7.1 Hz, 2H), 3.76 (s, 2H), 2.21 (s, 3H), 2.06 (s, 3H), 1.15 (t, J = 7.1 Hz, 3H). ^{13}C-NMR (100 MHz, $CDCl_3$): δ = 168.8, 165.5, 155.3, 143.0, 128.8 (q, $^2J_{C\text{-}F}$ = 32.5 Hz), 128.4, 125.6 (q, $^3J_{C\text{-}F}$ = 3.7 Hz), 124.4 (q, $^1J_{C\text{-}F}$ = 271.7 Hz), 118.9, 60.9, 34.3, 21.1, 19.1, 14.2. ^{19}F-NMR (376 MHz, $CDCl_3$): δ = -62.41(s). HR-MS (EI) m/z calcd for $C_{16}H_{17}F_3O_4$ [M+H$^+$] 331.1152, found 331.1157.

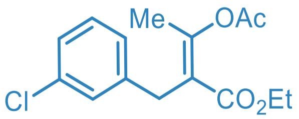

Ethyl (*Z*)-3-acetoxy-2-(3-chlorobenzyl)but-2-enoate (3h)

The general procedure **TP3** was followed using **ketoester** (3.2 mmol) for 18 h. Purification by column chromatography (petroleum ether/ EtOAc 20:1) yielded **3h** (568 mg, 60% yield) as a colorless oil. ^{1}H-NMR (400 MHz, $CDCl_3$): δ = 7.24 – 7.09 (m, 4H), 4.10 (q, J = 7.1 Hz, 2H), 3.68 (s, 2H), 2.21 (s, 3H), 2.06 (s, 3H), 1.17 (t, J = 7.1 Hz, 3H). ^{13}C-NMR (100 MHz, $CDCl_3$): δ = 168.7, 165.4, 155.0, 140.8, 134.3, 129.8, 128.3, 126.5, 126.2, 118.9, 60.7, 34.0, 20.9, 19.0, 14.1. HR-MS (EI) m/z calcd for $C_{15}H_{17}ClO_4$ [M+H$^+$] 297.0888, found 297.0891.

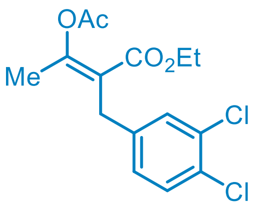

Ethyl (*Z*)-3-acetoxy-2-(3,4-dichlorobenzyl)but-2-enoate (3j)

The general procedure **TP3** was followed using **ketoester** (2 mmol) for 18 h. Purification by column chromatography (petroleum ether/ EtOAc 20:1) yielded **3j** (53%, 351 mg) as a colorless oil. ^{1}H-NMR (400 MHz, $CDCl_3$): δ = 7.34 (t, J = 5.1 Hz, 2H), 7.08 (dd, J = 8.2, 2.0 Hz, 1H), 4.10 (q, J = 7.1 Hz, 2H), 3.65 (s, 2H), 2.21 (s, 3H), 2.05 (s, 3H), 1.18 (t, J = 7.1 Hz, 3H). ^{13}C-NMR (100 MHz, $CDCl_3$): δ = 168.8, 165.4, 155.4, 139.1, 132.5, 130.5, 130.4, 130.2, 127.6, 118.8, 60.9, 33.6, 21.1, 19.2, 14.2. HR-MS (EI) m/z calcd for $C_{15}H_{16}Cl_2O_4$ [M+H$^+$] 331.0498, found 331.0501.

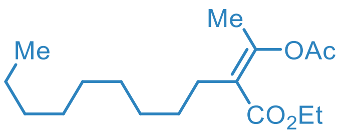

Ethyl (*Z*)-2-(1-acetoxyethylidene)undecanoate (3k)

The general procedure **TP3** was followed using **ketoester** (7.3 mmol) for 18 h. Purification by column chromatography (petroleum ether/ EtOAc 20:1) yielded **3k** (1.85 g, 85% yield) as a colorless oil. ^{1}H-NMR (400 MHz, $CDCl_3$): δ = 4.18 (q, J = 7.1 Hz, 2H), 2.35 – 2.27 (m, 2H), 2.17 (s, 3H), 2.01 (s, 3H), 1.28 (dd, J = 9.3, 4.9 Hz, 17H), 0.89 (t, J = 6.8 Hz, 3H). ^{13}C-NMR (100 MHz, $CDCl_3$): δ = 168.8, 166.5, 151.9, 121.4, 60.5, 32.0, 29.6, 29.5, 29.4, 29.4, 28.9, 28.9, 22.7, 21.0, 18.3, 14.3, 14.2. HR-MS (EI) m/z calcd for $C_{17}H_{30}O_4$ [M+H$^+$] 299.2217, found 299.2220.

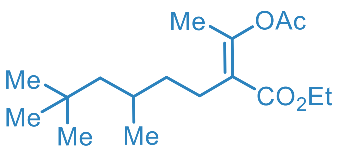

Ethyl (*Z*)-2-(1-acetoxyethylidene)-5,7,7-trimethyloctanoate (3l)

The general procedure **TP3** was followed using **ketoester** (7 mmol) for 18 h. Purification by column chromatography (petroleum ether/ EtOAc 20:1) yielded **3l** (1.62 g, 78% yield) as a colorless oil. ^{1}H-NMR (400 MHz, $CDCl_3$): δ = 4.16 (q, J = 7.1 Hz, 2H), 2.35 – 2.23 (m, 2H), 2.15 (s, 3H), 2.00 (s, 3H), 1.56 – 1.39 (m, 2H), 1.34 – 1.21 (m, 6H), 0.95 (d, J = 6.6 Hz, 3H),

0.89 (s, 9H). ^{13}C-NMR (100 MHz, CDCl$_3$): δ = 168.7, 166.2, 151.9, 121.3, 60.4, 51.0, 38.4, 31.1, 30.0, 29.3, 26.8, 22.4, 20.9, 18.2, 14.2. HR-MS (EI) m/z calcd for $C_{17}H_{30}O_4$ [M+H$^+$] 299.2217, found 299.2219.

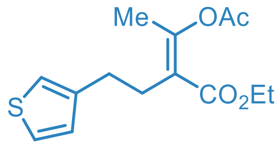

Ethyl (*Z*)-3-acetoxy-2-[2-(thiophen-3-yl)ethyl]but-2-enoate (3m)

The general procedure **TP3** was followed using **ketoester** (10.3 mmol) for 18 h. Purification by column chromatography (petroleum ether/ EtOAc 20:1) yielded **3m** (43%, 1.25 g) as a colorless oil. ^{1}H-NMR (400 MHz, CDCl$_3$): δ = 7.23 (dd, *J* = 4.8, 3.0 Hz, 1H), 6.97 (dd, *J* = 8.7, 3.6 Hz, 2H), 4.15 (q, *J* = 7.1 Hz, 2H), 2.80 (t, *J* = 7.5 Hz, 2H), 2.59 (t, *J* = 7.6 Hz, 2H), 2.16 (s, 3H), 1.85 (s, 3H), 1.27 (t, *J* = 7.1 Hz, 3H). ^{13}C-NMR (100 MHz, CDCl$_3$): δ = 168.7, 166.1, 153.4, 141.4, 128.5, 125.4, 121.0, 120.0, 60.6, 30.3, 29.4, 21.0, 18.2, 14.3. HR-MS (EI) m/z calcd for $C_{14}H_{18}O_4S$ [M+H$^+$] 283.0999, found 283.1003.

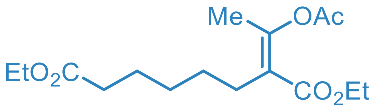

Diethyl (*Z*)-2-(1-acetoxyethylidene)octanedioate (3n)

The general procedure **TP3** was followed using **ketoester** (6.37 mmol) for 18 h. Purification by column chromatography (petroleum ether/ EtOAc 10:1) yielded **3n** (81%, 1.61 g) as a colorless oil. ^{1}H-NMR (400 MHz, CDCl$_3$): δ = 4.17 – 4.08 (m, 4H), 2.30 – 2.24 (m, 4H), 2.13 (s, 3H), 1.97 (s, 3H), 1.65 – 1.58 (m, 2H), 1.43 (dd, *J* = 15.3, 7.7 Hz, 2H), 1.37 – 1.31 (m, 2H), 1.24 (m, 6H). ^{13}C-NMR (100 MHz, CDCl$_3$): δ = 173.8, 168.8, 166.3, 152.1, 121.0, 60.5, 60.3, 34.3, 28.8, 28.7, 28.6, 24.8, 21.0, 18.3, 14.3. HR-MS (EI) m/z calcd for $C_{16}H_{26}O_6$ [M+H$^+$] 315.1802, found 315.1808.

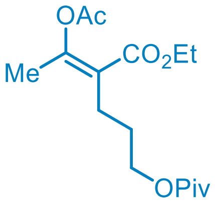

Ethyl (*Z*)-2-(1-acetoxyethylidene)-5-(pivaloyloxy)pentanoate (3o)

The general procedure **TP3** was followed using **ketoester** (5 mmol) for 18 h. Purification by column chromatography (petroleum ether/ EtOAc 20:1) yielded **3o** (70%, 1.1 g) as a pale yellow oil. ^{1}H-NMR (400 MHz, CDCl$_3$): δ = 4.15 (q, J = 7.1 Hz, 2H), 4.06 (t, J = 6.2 Hz, 2H), 2.40 – 2.33 (m, 2H), 2.14 (s, 3H), 1.99 (s, 3H), 1.77 (ddt, J = 12.4, 9.8, 6.1 Hz, 2H), 1.25 (t, J = 7.1 Hz, 3H), 1.18 (s, 9H). ^{13}C-NMR (100 MHz, CDCl$_3$): δ = 178.5, 168.7, 165.9, 153.2, 120.1, 63.6, 60.6, 38.8, 28.1, 27.3, 25.5, 21.0, 18.4, 14.3. HR-MS (EI) m/z calcd for $C_{16}H_{26}O_6$ [M+H$^+$] 315.1806, found 315.1811.

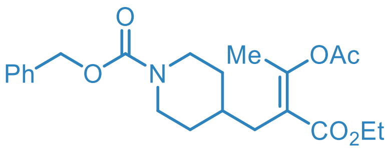

Benzyl (*Z*)-4-[3-acetoxy-2-(ethoxycarbonyl)but-2-en-1-yl]piperidine-1-carboxylate (3p)

The general procedure **TP3** was followed using **ketoester** (6.9 mmol) for 18 h. Purification by column chromatography (petroleum ether/ EtOAc 5:1) yielded **3p** (68%, 1.88 g) as an orange oil. ^{1}H-NMR (400 MHz, CDCl$_3$): δ = 7.34 (t, J = 7.3 Hz, 5H), 5.12 (s, 2H), 4.17 (dd, J = 14.2, 7.1 Hz, 4H), 2.72 (s, 2H), 2.27 (d, J = 7.1 Hz, 2H), 2.16 (s, 3H), 1.98 (s, 3H), 1.73 (d, J = 12.8 Hz, 2H), 1.60 (ddd, J = 11.3, 9.4, 5.5 Hz, 1H), 1.27 (t, J = 7.1 Hz, 3H), 1.13 (d, J = 10.5 Hz, 2H). ^{13}C-NMR (100 MHz, CDCl$_3$): δ = 168.7, 166.2, 155.2, 152.8, 136.9, 128.4, 127.9, 127.8, 119.2, 66.9, 60.6, 44.2, 36.1, 35.4, 31.8, 20.9, 18.7, 14.2. HR-MS (EI) m/z calcd for $C_{22}H_{29}NO_6$ [M+H$^+$] 404.2068, found 404.2075.

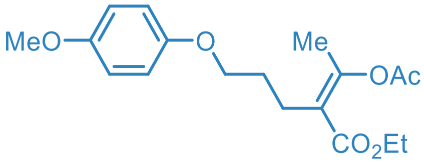

Ethyl (*Z*)-2-(1-acetoxyethylidene)-5-(4-methoxyphenoxy)pentanoate (3q)

The general procedure **TP3** was followed using **ketoester** (5.4 mmol) for 18 h. Purification by column chromatography (petroleum ether/ EtOAc 10:1) yielded **3q** (51%, 922 mg) as a yellow oil. ^{1}H-NMR (400 MHz, CDCl$_3$): δ = 6.82 (s, 4H), 4.15 (q, J = 7.1 Hz, 2H), 3.92 (t, J = 5.9 Hz, 2H), 3.75 (s, 3H), 2.52 (t, J = 7.3 Hz, 2H), 2.15 (s, 3H), 1.98 (s, 3H), 1.92 (dt, J = 13.0, 6.5 Hz, 2H), 1.25 (t, J = 7.1 Hz, 3H). ^{13}C-NMR (100 MHz, CDCl$_3$): δ = 168.8, 166.2, 153.8, 153.3, 153.1, 120.0, 115.5, 114.7, 66.9, 60.6, 55.8, 28.4, 25.3, 21.0, 18.4, 14.3. HR-MS (EI) m/z calcd for $C_{18}H_{24}O_6$ [M+H$^+$] 337.1646, found 337.1651.

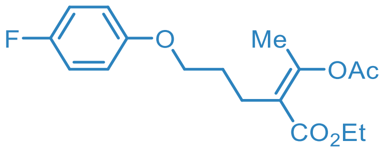

Ethyl (*Z*)-2-(1-acetoxyethylidene)-5-(4-fluorophenoxy)pentanoate (3r)

The general procedure **TP3** was followed using **ketoester** (3 mmol) for 18 h. Purification by column chromatography (petroleum ether/ EtOAc 20:1) yielded **3r** (51%, 496 mg) as a yellow oil. ^{1}H-NMR (400 MHz, $CDCl_3$): δ = 6.97 – 6.90 (m, 2H), 6.84 – 6.78 (m, 2H), 4.14 (q, J = 7.1 Hz, 2H), 3.92 (t, J = 6.0 Hz, 2H), 2.51 (t, J = 7.2 Hz, 2H), 2.14 (s, 3H), 1.96 (s, 3H), 1.91 (dd, J = 13.4, 6.9 Hz, 2H), 1.24 (t, J = 7.1 Hz, 3H). ^{13}C-NMR (100 MHz, $CDCl_3$): δ = 168.8, 166.1, 157.3 (d, $^1J_{C-F}$ = 237.8 Hz), 155.1 (d, $^4J_{C-F}$ = 2.0 Hz), 153.2, 120.0, 115.8 (d, $^2J_{C-F}$ = 22.9 Hz), 115.5 (d, $^3J_{C-F}$ = 7.9 Hz), 66.9, 60.6, 28.3, 25.2, 21.0, 18.3, 14.3. ^{19}F-NMR (376 MHz, $CDCl_3$): δ = -124.30 (s). HR-MS (EI) m/z calcd for $C_{17}H_{21}FO_5$ [M+H$^+$] 325.1446, found 325.1448.

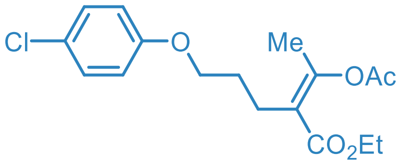

Ethyl (*Z*)-2-(1-acetoxyethylidene)-5-(4-chlorophenoxy)pentanoate (3s)

The general procedure **TP3** was followed using **ketoester** (2.9 mmol) for 18 h. Purification by column chromatography (petroleum ether/ EtOAc 10:1) yielded **3s** (73%, 720 mg) as a yellow oil. ^{1}H-NMR (400 MHz, $CDCl_3$): δ = 7.23 – 7.19 (m, 2H), 6.84 – 6.79 (m, 2H), 4.15 (q, J = 7.1 Hz, 2H), 3.94 (t, J = 5.9 Hz, 2H), 2.52 (t, J = 7.2 Hz, 2H), 2.15 (s, 3H), 1.96 (s, 3H), 1.92 (dd, J = 13.3, 7.0 Hz, 2H), 1.25 (t, J = 7.1 Hz, 3H). ^{13}C-NMR (100 MHz, $CDCl_3$): δ = 168.8, 166.1, 157.6, 153.3, 129.4, 125.5, 119.9, 115.8, 66.6, 60.7, 28.2, 25.2, 21.0, 18.4, 14.3. HR-MS (EI) m/z calcd for $C_{17}H_{21}ClO_5$ [M+H$^+$] 341.1150, found 341.1156.

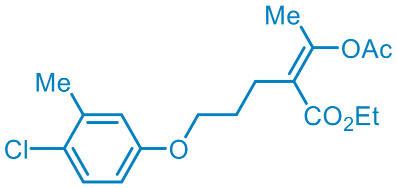

Ethyl (*Z*)-2-(1-acetoxyethylidene)-5-(4-chloro-3-methylphenoxy)pentanoate (3t)

The general procedure **TP3** was followed using **ketoester** (1.2 mmol) for 18 h. Purification by column chromatography (petroleum ether/ EtOAc 20:1) yielded **3t** (55%, 234 mg) as a colorless oil. ^{1}H-NMR (400 MHz, $CDCl_3$): δ = 7.20 (d, J = 8.7 Hz, 1H), 6.76 (d, J = 2.9 Hz, 1H), 6.66

(dd, J = 8.7, 3.0 Hz, 1H), 4.15 (q, J = 7.1 Hz, 2H), 3.93 (t, J = 5.9 Hz, 2H), 2.52 (t, J = 7.2 Hz, 2H), 2.33 (s, 3H), 2.16 (s, 3H), 1.97 (s, 3H), 1.92 (dd, J = 13.3, 6.9 Hz, 2H), 1.26 (t, J = 7.1 Hz, 3H). ^{13}C-NMR (100 MHz, $CDCl_3$): δ = 168.8, 166.1, 157.5, 153.4, 137.1, 129.7, 125.8, 120.0, 117.1, 113.2, 66.6, 60.7, 28.3, 25.3, 21.1, 20.4, 18.4, 14.4. HR-MS (EI) m/z calcd for $C_{18}H_{23}ClO_5$ [M+H$^+$] 355.1307, found 355.1311.

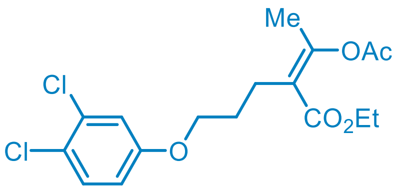

Ethyl (*Z*)-2-(1-acetoxyethylidene)-5-(3,4-dichlorophenoxy)pentanoate (3u)

The general procedure **TP3** was followed using **ketoester** (4 mmol) for 18 h. Purification by column chromatography (petroleum ether/ EtOAc 20:1) yielded **3u** (60%, 900 mg) as a colorless oil. ^{1}H-NMR (400 MHz, $CDCl_3$): δ = 7.30 (d, J = 8.9 Hz, 1H), 6.98 (d, J = 2.9 Hz, 1H), 6.74 (dd, J = 8.9, 2.9 Hz, 1H), 4.15 (q, J = 7.1 Hz, 2H), 3.94 (t, J = 5.9 Hz, 2H), 2.51 (t, J = 7.2 Hz, 2H), 2.16 (s, 3H), 1.97 (s, 3H), 1.93 (dd, J = 13.3, 6.9 Hz, 2H), 1.26 (t, J = 7.1 Hz, 3H). ^{13}C-NMR (100 MHz, $CDCl_3$): δ = 168.8, 166.1, 158.1, 153.4, 132.9, 130.8, 123.9, 119.8, 116.5, 114.5, 67.0, 60.7, 28.1, 25.2, 21.0, 18.5, 14.3. HR-MS (EI) m/z calcd for $C_{17}H_{20}Cl_2O_5$ [M+H$^+$] 375.0761, found 375.0764.

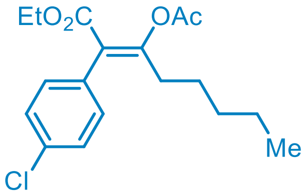

Ethyl (*Z*)-3-acetoxy-2-(4-chlorophenyl)oct-2-enoate (3v)

The general procedure **TP3** was followed using **ketoester** (8.6 mmol) for 18 h. Purification by column chromatography (petroleum ether/ EtOAc 80:1) yielded **3v** (60%, 1.76 g) as a yellow oil. ^{1}H-NMR (400 MHz, $CDCl_3$): δ = 7.36 – 7.29 (m, 2H), 7.23 – 7.16 (m, 2H), 4.11 (q, J = 7.1 Hz, 2H), 2.24 (s, 3H), 2.17 – 2.10 (m, 2H), 1.45 – 1.39 (m, 2H), 1.22 – 1.12 (m, 7H), 0.82 (t, J = 6.9 Hz, 3H). ^{13}C-NMR (100 MHz, $CDCl_3$): δ = 168.6, 165.1, 158.8, 133.9, 133.6, 131.4, 128.6, 122.1, 61.0, 32.6, 31.4, 26.1, 22.4, 21.2, 14.2, 13.9. HR-MS (EI) m/z calcd for $C_{18}H_{23}ClO_4$ [M+H$^+$] 339.1358, found 339.1364.

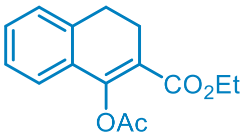

Ethyl 1-acetoxy-3,4-dihydronaphthalene-2-carboxylate (3x)

The general procedure **TP3** was followed using **ketoester** (6.8 mmol) for 18 h. Purification by column chromatography (petroleum ether/ EtOAc 30:1) yielded **3x** (1.729 g, 98% yield) as a colorless oil. ^{1}H-NMR (400 MHz, $CDCl_3$): δ = 7.35 – 7.30 (m, 1H), 7.29 – 7.26 (m, 1H), 7.25 – 7.17 (m, 2H), 4.23 (q, J = 7.1 Hz, 2H), 2.92 – 2.86 (m, 2H), 2.79 – 2.73 (m, 2H), 2.37 (s, 3H), 1.32 (t, J = 7.1 Hz, 3H). ^{13}C-NMR (100 MHz, $CDCl_3$): δ = 168.6, 165.8, 151.1, 138.3, 130.4, 130.2, 127.7, 126.8, 123.5, 116.7, 60.8, 27.4, 23.8, 21.0, 14.4. HR-MS (EI) m/z calcd for $C_{15}H_{16}O_4$ [M+H$^+$] 261.1121, found 261.1126.

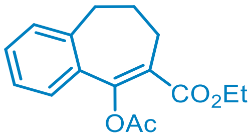

Ethyl 9-acetoxy-6,7-dihydro-5*H*-benzo[7]annulene-8-carboxylate (3y)

The general procedure **TP3** was followed using **ketoester** (4 mmol) for 18 h. Purification by column chromatography (petroleum ether/ EtOAc 70:1) yielded **3y** (657 mg, 60% yield) as a colorless oil. ^{1}H-NMR (400 MHz, $CDCl_3$): δ = 7.36 (dt, J = 10.5, 5.1 Hz, 1H), 7.30 (ddd, J = 9.9, 7.3, 1.7 Hz, 2H), 7.23 (dd, J = 7.1, 1.6 Hz, 1H), 4.25 (q, J = 7.1 Hz, 2H), 2.77 (t, J = 6.7 Hz, 2H), 2.27 – 2.19 (m, 7H), 1.33 (t, J = 7.1 Hz, 3H). ^{13}C-NMR (100 MHz, $CDCl_3$): δ = 168.9, 166.1, 152.9, 142.1, 135.8, 129.9, 129.2, 126.7, 126.4, 120.8, 60.9, 34.7, 31.7, 25.1, 21.0, 14.4. HR-MS (EI) m/z calcd for $C_{16}H_{18}O_4$ [M+H$^+$] 275.1278, found 275.1280.

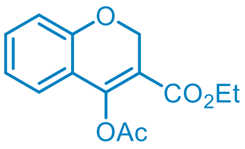

Ethyl 4-acetoxy-2*H*-chromene-3-carboxylate (3z)

The general procedure **TP3** was followed using **ketoester** (3 mmol) for 18 h. Purification by column chromatography (petroleum ether/ EtOAc 20:1) yielded **3z** (596 mg, 76% yield) as a colorless oil. ^{1}H-NMR (400 MHz, $CDCl_3$): δ = 7.31 – 7.26 (m, 1H), 7.24 (d, J = 1.5 Hz, 1H), 6.96 (td, J = 7.6, 1.1 Hz, 1H), 6.90 – 6.84 (m, 1H), 5.10 (s, 2H), 4.23 (q, J = 7.1 Hz, 2H), 2.38 (s, 3H), 1.31 (t, J = 7.1 Hz, 3H). ^{13}C-NMR (100 MHz, $CDCl_3$): δ = 167.8, 162.8, 156.9, 149.8,

132.8, 124.0, 121.8, 119.1, 116.5, 109.7, 65.4, 60.9, 20.9, 14.3. HR-MS (EI) m/z calcd for $C_{14}H_{14}O_5$ [M+H$^+$] 263.0914, found 263.0918.

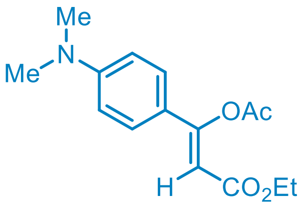

Ethyl (*Z*)-3-acetoxy-3-[4-(dimethylamino)phenyl]acrylate (3ac)

The general procedure **TP3** was followed using **ketoester** (4.5 mmol) for 18 h. Purification by column chromatography (petroleum ether/ EtOAc 10:1) yielded **3ac** (873 mg, 70% yield) as a yellow solid. ^{1}H-MR (400 MHz, $CDCl_3$): δ = 7.49 – 7.44 (m, 2H), 6.68 – 6.62 (m, 2H), 6.09 (s, 1H), 4.17 (q, J = 7.1 Hz, 2H), 3.02 (s, 6H), 2.39 (s, 3H), 1.29 (t, J = 7.1 Hz, 3H). ^{13}C-NMR (100 MHz, $CDCl_3$): δ = 168.4, 165.1, 159.1, 152.3, 127.5, 119.8, 111.8, 101.1, 60.0, 40.2, 21.2, 14.5. HR-MS (EI) m/z calcd for $C_{15}H_{19}NO_4$ [M+H$^+$] 278.1387, found 278.1391.

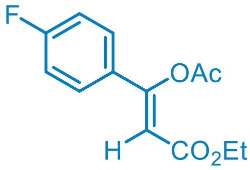

Ethyl (*Z*)-3-acetoxy-3-(4-fluorophenyl)acrylate (3ad)

The general procedure **TP3** was followed using **ketoester** (4.26 mmol) for 18 h. Purification by column chromatography (petroleum ether/ EtOAc 50:1) yielded **3ad** (84%, 898 mg) as a yellow oil. ^{1}H-NMR (400 MHz, $CDCl_3$): δ = 7.60 – 7.54 (m, 2H), 7.12 – 7.05 (m, 2H), 6.20 (s, 1H), 4.19 (q, J = 7.1 Hz, 2H), 2.38 (s, 3H), 1.29 (t, J = 7.1 Hz, 3H). ^{13}C-NMR (100 MHz, $CDCl_3$): δ = 166.9 (d, $^1J_{C-F}$ = 243.6 Hz), 164.2, 163.2, 157.2, 129.8 (d, $^4J_{C-F}$ = 3.2 Hz), 128.2 (d, $^3J_{C-F}$ = 8.7 Hz), 116.1 (d, $^2J_{C-F}$ = 22.2 Hz), 106.1, 60.5, 21.1, 14.3. ^{19}F-NMR (376 MHz, $CDCl_3$): δ = -108.62 (s). HR-MS (EI) m/z calcd for $C_{13}H_{13}FO_3$ [M+H$^+$] 253.0871, found 253.0878.

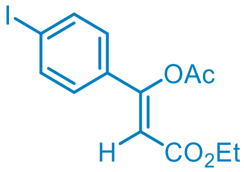

Ethyl (*Z*)-3-acetoxy-3-(4-iodophenyl)acrylate (3af)

The general procedure **TP3** was followed using **ketoester** (12 mmol) for 18 h. Purification by column chromatography (petroleum ether/ EtOAc 10:1) yielded **3af** (62%, 2.67 g) as a yellow solid. ^{1}H-NMR (400 MHz, $CDCl_3$): δ = 7.77 – 7.73 (m, 2H), 7.32 – 7.28 (m, 2H), 6.25 (s, 1H), 4.20 (q, J = 7.1 Hz, 2H), 2.38 (s, 3H), 1.30 (t, J = 7.1 Hz, 3H). ^{13}C-NMR (100 MHz, $CDCl_3$): δ = 168.0, 164.0, 157.2, 138.1, 133.0, 127.5, 106.7, 97.7, 60.5, 21.0, 14.3. HR-MS (EI) m/z calcd for $C_{13}H_{13}IO_4$ [M+H$^+$] 360.9931, found 360.9937.

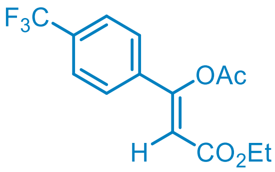

Ethyl (*Z*)-3-acetoxy-3-[4-(trifluoromethyl)phenyl]acrylate (3ag)

The general procedure TP3 was followed using ketoester (7.2 mmol) for 18 h. Purification by column chromatography (petroleum ether/ EtOAc 30:1) yielded **3ag** (86%, 1.87 g) as an orange oil. ^{1}H-NMR (400 MHz, $CDCl_3$): δ = 7.68 (q, J = 8.7 Hz, 4H), 6.32 (s, 1H), 4.22 (q, J = 7.1 Hz, 2H), 2.40 (s, 3H), 1.31 (t, J = 7.1 Hz, 3H). ^{13}C-NMR (100 MHz, $CDCl_3$): δ = 168.1, 163.9, 156.5, 137.1, 132.7 (q, $^2J_{C\text{-}F}$ = 32.8 Hz), 126.4, 126.0 (q, $^3J_{C\text{-}F}$ = 3.7 Hz), 123.8 (q, $^1J_{C\text{-}F}$ = 272.5 Hz), 108.4, 60.8, 21.0, 14.4. ^{19}F-NMR (376 MHz, $CDCl_3$): δ = -63.01 (s). HR-MS (EI) m/z calcd for $C_{14}H_{13}F_3O_4$ [M+H$^+$] 303.0839, found 303.0841.

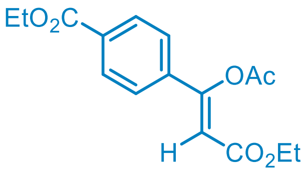

Ethyl (*Z*)-4-(1-acetoxy-3-ethoxy-3-oxoprop-1-en-1-yl)benzoate (3ah)

The general procedure **TP3** was followed using **ketoester** (3 mmol) for 18 h. Purification by column chromatography (petroleum ether/ EtOAc 15:1) yielded **3ah** (80%, 733 mg) as an white solid. ^{1}H-NMR (400 MHz, $CDCl_3$): δ = 8.07 – 8.02 (m, 2H), 7.65 – 7.60 (m, 2H), 6.32 (s, 1H), 4.37 (q, J = 7.1 Hz, 2H), 4.19 (q, J = 7.1 Hz, 2H), 2.38 (s, 3H), 1.38 (t, J = 7.1 Hz, 3H), 1.29 (t, J = 7.1 Hz, 3H). ^{13}C-NMR (100 MHz, $CDCl_3$): δ = 168.0, 165.7, 163.9, 156.8, 137.5, 132.5, 130.0, 125.9, 108.0, 61.3, 60.6, 20.9, 14.3, 14.3. HR-MS (EI) m/z calcd for $C_{16}H_{18}O_6$ [M+H$^+$] 307.1176, found 307.1179.

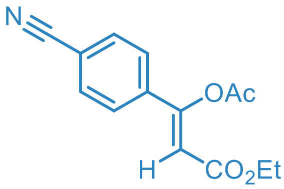

Ethyl (*Z*)-3-acetoxy-3-(4-cyanophenyl)acrylate (3ai)

The general procedure **TP3** was followed using **ketoester** (7 mmol) for 18 h. Purification by column chromatography (petroleum ether/ EtOAc 15:1) yielded **3ai** (70%, 1.27 g) as a yellow solid. ^{1}H-NMR (400 MHz, $CDCl_3$): δ = 7.72 – 7.66 (m, 4H), 6.32 (s, 1H), 4.22 (q, J = 7.1 Hz, 2H), 2.40 (s, 3H), 1.31 (t, J = 7.1 Hz, 3H). ^{13}C-NMR (100 MHz, $CDCl_3$): δ = 167.9, 163.6, 155.7, 137.8, 132.6, 126.5, 118.1, 114.3, 109.1, 60.8, 20.9, 14.2. HR-MS (EI) m/z calcd for $C_{14}H_{13}NO_4$ [M+H$^+$] 260.0917, found 260.0922.

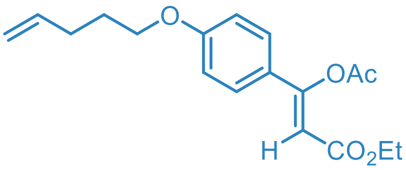

Ethyl (*Z*)-3-acetoxy-3-[4-(pent-4-en-1-yloxy)phenyl]acrylate (3al)

The general procedure **TP3** was followed using **ketoester** (2.87 mmol) for 18 h. Purification by column chromatography (petroleum ether/ EtOAc 10:1) yielded **3al** (67%, 611 mg) as a colorless oil. ^{1}H-NMR (400 MHz, $CDCl_3$): δ = 7.54 – 7.49 (m, 2H), 6.91 – 6.86 (m, 2H), 6.16 (s, 1H), 5.84 (ddt, J = 16.9, 10.2, 6.7 Hz, 1H), 5.06 (ddd, J = 17.2, 3.2, 1.6 Hz, 1H), 5.01 (dd, J = 10.2, 1.3 Hz, 1H), 4.18 (q, J = 7.1 Hz, 2H), 3.99 (t, J = 6.4 Hz, 2H), 2.39 (s, 3H), 2.23 (dd, J = 14.6, 6.8 Hz, 2H), 1.93 – 1.85 (m, 2H), 1.29 (t, J = 7.1 Hz, 3H). ^{13}C-NMR (100 MHz, $CDCl_3$): δ = 168.2, 164.6, 161.5, 158.2, 137.6, 127.7, 125.6, 115.5, 114.8, 103.9, 67.4, 60.2, 30.1, 28.3, 21.1, 14.4. HR-MS (EI) m/z calcd for $C_{18}H_{22}O_5$ [M+H$^+$] 319.1540, found 319.1547.

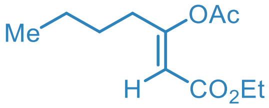

Ethyl (*Z*)-3-acetoxyhept-2-enoate (3am)

The general procedure **TP3** was followed using **ketoester** (6.7 mmol) for 18 h. Purification by column chromatography (petroleum ether/ EtOAc 20:1) yielded **3am** (75%, 1.08g) as a colorless oil. ^{1}H-NMR (400 MHz, $CDCl_3$): δ = 5.58 (s, 1H), 4.12 (q, J = 7.1 Hz, 2H), 2.26 (t, J = 7.4 Hz, 2H), 2.23 (s, 3H), 1.50 (dt, J = 15.2, 7.3 Hz, 2H), 1.36 (dd, J = 15.0, 7.4 Hz, 2H), 1.24 (t, J = 7.1 Hz, 3H), 0.91 (t, J = 7.3 Hz, 3H). ^{13}C-NMR (100 MHz, $CDCl_3$): δ = 168.1, 164.1,

163.6, 107.3, 60.1, 35.2, 28.0, 22.2, 21.1, 14.3, 13.8. HR-MS (EI) m/z calcd for $C_{11}H_{18}O_4$ [M+H$^+$] 215.1278, found 215.1281.

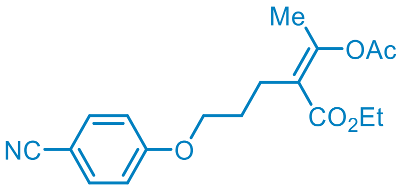

Ethyl (*Z*)-2-(1-acetoxyethylidene)-5-(4-cyanophenoxy)pentanoate (3an)

The general procedure **TP3** was followed using **ketoester** (3.3 mmol) for 18 h. Purification by column chromatography (petroleum ether/ EtOAc 10:1) yielded **3an** (62%, 677 mg) as a colorless oil. ^{1}H-NMR (400 MHz, $CDCl_3$): δ = 7.60 – 7.51 (m, 2H), 6.97 – 6.90 (m, 2H), 4.14 (q, J = 7.1 Hz, 2H), 4.03 (t, J = 6.0 Hz, 2H), 2.53 (t, J = 7.1 Hz, 2H), 2.15 (s, 3H), 2.01 – 1.95 (m, 2H), 1.95 (s, 3H), 1.25 (t, J = 7.1 Hz, 3H). ^{13}C-NMR (100 MHz, $CDCl_3$): δ = 168.8, 166.0, 162.3, 153.4, 134.1, 119.8, 119.4, 115.3, 104.0, 66.8, 60.7, 28.0, 25.2, 21.0, 18.4, 14.3. HR-MS (EI) m/z calcd for $C_{18}H_{21}NO_5$ [M+H$^+$] 332.1492, found 332.1495.

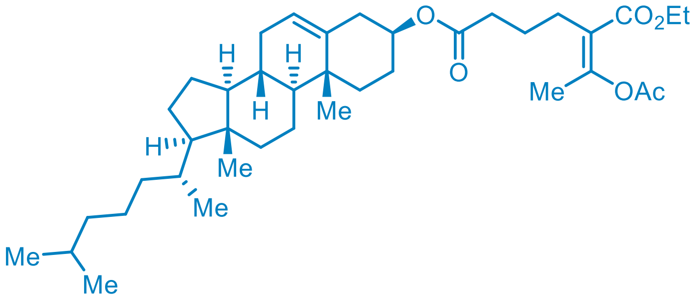

6-{(3*S*,8*S*,9*S*,10*R*,13*R*,14*S*,17*R*)-10,13-dimethyl-17-[(*R*)-6-methylheptan-2-yl]-2,3,4,7,8,9, 10,11,12,13,14,15,16,17-tetradecahydro-1*H*-cyclopenta[a]phenanthren-3-yl} 1-ethyl (*Z*)-2 -(1-acetoxyethylidene)hexanedioate (3ap)

The general procedure **TP3** was followed using **ketoester** (3.5 mmol) for 18 h. Purification by column chromatography (petroleum ether/ EtOAc 10:1) yielded **3ap** (61%, 1.34 g) as a white solid. ^{1}H-NMR (400 MHz, $CDCl_3$) δ = 5.35 (d, J = 4.1 Hz, 1H), 4.65 – 4.54 (m, 1H), 4.15 (q, J = 7.1 Hz, 2H), 2.32 (ddd, J = 11.2, 10.4, 5.8 Hz, 6H), 2.14 (s, 3H), 2.04 – 1.91 (m, 5H), 1.87 – 1.71 (m, 5H), 1.58 – 0.90 (m, 30H), 0.84 (dd, J = 6.6, 1.6 Hz, 6H), 0.66 (s, 3H). ^{13}C-NMR (100 MHz, $CDCl_3$): δ = 172.8, 168.7, 166.0, 153.2, 139.7, 122.7, 120.3, 74.0, 60.6, 56.8, 56.2, 50.1, 42.4, 39.8, 39.6, 38.2, 37.1, 36.7, 36.3, 35.9, 33.8, 32.0, 31.9, 28.3, 28.2, 28.1, 27.9, 24.4, 24.2, 23.9, 22.9, 22.7, 21.1, 21.0, 19.4, 18.8, 18.5, 14.3, 12.0. HR-MS (EI) m/z calcd for $C_{39}H_{62}O_6$ [M+H$^+$] 627.4619, found 627.4623.

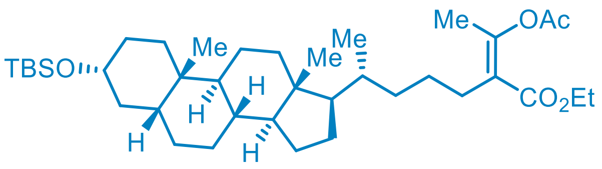

Ethyl (*R*,*Z*)-2-(1-acetoxyethylidene)-6-{(3*R*,5*R*,8*R*,9*S*,10*S*,13*R*,14*S*,17*R*)-3-[(tert-butyldimethylsilyl)oxy]-10,13-dimethylhexadecahydro-1*H*-cyclopenta[a]phenanthren-17-yl}heptanoate (3aq)

The general procedure **TP3** was followed using **ketoester** (2 mmol) for 18 h. Purification by column chromatography (petroleum ether/ EtOAc 10:1) yielded **3aq** (42%, 529 mg) as a colorless oil. ^{1}H-NMR (400 MHz, $CDCl_3$): δ = 4.17 (q, J = 7.1 Hz, 2H), 3.63 – 3.52 (m, 1H), 2.15 (s, 3H), 1.99 (s, 3H), 1.86 – 1.74 (m, 4H), 1.50 – 0.98 (m, 30H), 0.92 – 0.84 (m, 17H), 0.06 (s, 6H). ^{13}C-NMR (100 MHz, $CDCl_3$): δ = 168.9, 166.5, 151.9, 121.5, 73.0, 60.5, 56.6, 56.3, 42.8, 42.5, 40.4, 40.3, 37.1, 36.0, 35.7, 35.7, 34.7, 31.2, 29.9, 29.4, 28.4, 27.5, 26.6, 26.1, 25.5, 24.4, 23.5, 21.1, 21.0, 18.7, 18.5, 18.4, 14.4, 12.2, -4.5. HR-MS (EI) m/z calcd for $C_{38}H_{66}O_5Si$ [M+H$^+$] 631.4752, found 631.4754.

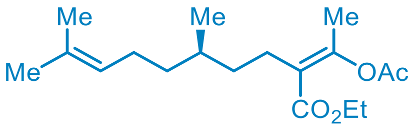

Ethyl (*R*,*Z*)-2-(1-acetoxyethylidene)-5,9-dimethyldec-8-enoate (3ar)

The general procedure **TP3** was followed using **ketoester** (4 mmol) for 18 h. Purification by column chromatography (petroleum ether/ EtOAc 20:1) yielded **3ar** (65%, 806 mg) as a colorless oil. ^{1}H-NMR (400 MHz, $CDCl_3$): δ = 5.11 – 5.03 (m, 1H), 4.15 (q, J = 7.1 Hz, 2H), 2.36 – 2.19 (m, 2H), 2.14 (s, 3H), 2.02 – 1.89 (m, 5H), 1.67 (s, 3H), 1.59 (s, 3H), 1.44 (ddd, J = 12.7, 10.6, 5.3 Hz, 2H), 1.37 – 1.24 (m, 5H), 1.20 – 1.09 (m, 1H), 0.90 (d, J = 6.4 Hz, 3H). ^{13}C-NMR (100 MHz, $CDCl_3$): δ = 168.9, 166.4, 151.9, 131.3, 124.9, 121.5, 60.5, 36.9, 36.0, 32.5, 26.6, 25.8, 25.6, 21.0, 19.5, 18.2, 17.7, 14.3. HR-MS (EI) m/z calcd for $C_{18}H_{30}O_4$ [M+H$^+$] 311.2217, found 311.2218.

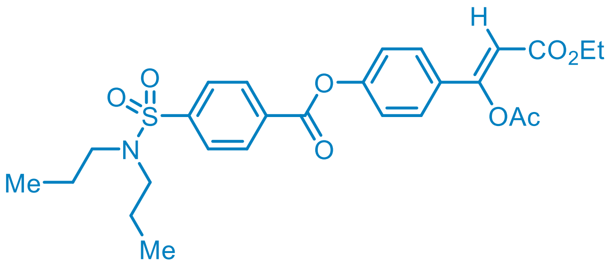

(*Z*)-4-(1-Acetoxy-3-ethoxy-3-oxoprop-1-en-1-yl)phenyl 4-(N,N dipropylsulfamoyl) benzoate (3as)

The general procedure **TP3** was followed using **ketoester** (2.44 mmol) for 18 h. Purification by column chromatography (petroleum ether/ EtOAc 5:1) yielded **3as** (60%, 757 mg) as a white solid. ^{1}H-NMR (400 MHz, $CDCl_3$): δ = 8.33 – 8.30 (m, 2H), 7.97 – 7.93 (m, 2H), 7.70 – 7.65 (m, 2H), 7.31 – 7.27 (m, 2H), 6.27 (s, 1H), 4.21 (q, J = 7.1 Hz, 2H), 3.16 – 3.11 (m, 4H), 2.41 (s, 3H), 1.62 – 1.56 (m, 4H), 1.31 (t, J = 7.1 Hz, 3H), 0.89 (t, J = 7.4 Hz, 6H). ^{13}C-NMR (100 MHz, $CDCl_3$): δ = 168.1, 164.1, 163.5, 157.1, 152.7, 145.3, 132.5, 131.6, 131.0, 127.5, 127.3, 122.1, 106.6, 60.5, 50.0, 22.0, 21.1, 14.3, 11.3. HR-MS (EI) m/z calcd for $C_{26}H_{31}NO_8S$ [$M+H^+$] 518.1843, found 518.1849.

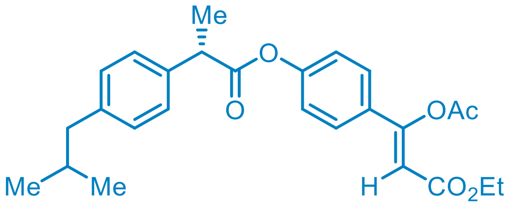

Ethyl (*S*,*Z*)-3-acetoxy-3-{4-{[2-(4-isobutylphenyl)propanoyl]oxy}phenyl}acrylate (3at)

The general procedure **TP3** was followed using **ketoester** (3.3 mmol) for 18 h. Purification by column chromatography (petroleum ether/ EtOAc 15:1) yielded **3at** (72%, 1.04 g) as an orange oil. ^{1}H-NMR (400 MHz, $CDCl_3$): δ = 7.57 – 7.53 (m, 2H), 7.29 (d, J = 8.1 Hz, 2H), 7.15 (d, J = 8.1 Hz, 2H), 7.07 – 7.03 (m, 2H), 6.20 (s, 1H), 4.19 (q, J = 7.1 Hz, 2H), 3.94 (q, J = 7.1 Hz, 1H), 2.47 (d, J = 7.2 Hz, 2H), 2.37 (s, 3H), 1.87 (m, 1H), 1.60 (d, J = 7.2 Hz, 3H), 1.29 (t, J = 7.1 Hz, 3H), 0.91 (d, J = 6.6 Hz, 6H). ^{13}C-NMR (100 MHz, $CDCl_3$): δ = 172.9, 168.1, 164.2, 157.4, 153.1, 141.1, 137.1, 131.1, 129.7, 127.3, 127.3, 122.0, 106.3, 60.5, 45.4, 45.2, 30.3, 22.5, 21.1, 18.6, 14.4. HR-MS (EI) m/z calcd for $C_{26}H_{30}O_6$ [$M+H^+$] 439.2115, found 439.2121.

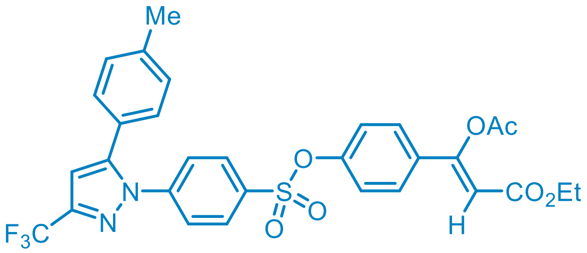

Ethyl (*Z*)-3-acetoxy-3-{4-{{{4-[5-(*p*-tolyl)-3-(trifluoromethyl)-1*H*-pyrazol-1-yl]phenyl}sulfonyl}oxy}phenyl}acrylate (3au)

The general procedure **TP3** was followed using **ketoester** (1.4 mmol) for 18 h. Purification by column chromatography (petroleum ether/ EtOAc 10:1) yielded **3au** (82%, 708 mg) as a white solid. ^{1}H-NMR (400 MHz, $CDCl_3$): δ = 7.82 – 7.78 (m, 2H), 7.54 – 7.48 (m, 4H), 7.18 (d, J = 7.9 Hz, 2H), 7.09 (d, J = 8.1 Hz, 2H), 7.06 – 7.00 (m, 2H), 6.75 (s, 1H), 6.20 (s, 1H), 4.20 (q, J = 7.1 Hz, 2H), 2.39 (s, 3H), 2.37 (s, 3H), 1.29 (t, J = 7.1 Hz, 3H). ^{13}C-NMR (100 MHz, $CDCl_3$): δ = 168.1, 164.0, 156.5, 151.1, 145.6, 144.6 (q, $^{2}J_{C\text{-}F}$ = 38.5 Hz), 144.0, 140.2, 134.3, 132.9, 131.0, 129.6, 128.9, 127.7, 125.7, 125.5, 122.9, 121.1 (q, $^{1}J_{C\text{-}F}$ = 269.3 Hz), 107.3, 106.8, 60.6, 21.5, 21.0, 14.3. ^{19}F-NMR (376 MHz, $CDCl_3$): δ = -62.52 (s). HR-MS (EI) m/z calcd for $C_{30}H_{25}F_3N_2O_7S$ [M+H$^+$] 615.1407, found 615.1423.

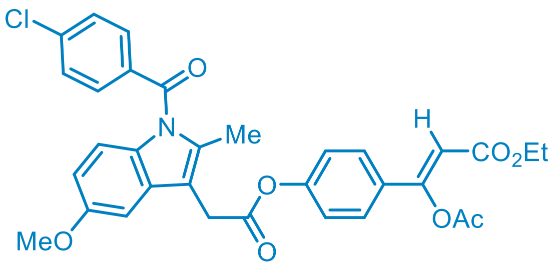

Ethyl (*Z*)-3-acetoxy-3-{4-{2-[1-(4-chlorobenzoyl)-5-methoxy-2-methyl-1*H*-indol-3-yl]acetoxy}phenyl}acrylate (3av)

The general procedure **TP3** was followed using **ketoester** (2.5 mmol) for 18 h. Purification by column chromatography (petroleum ether/ EtOAc 10:1) yielded **3av** (22%, 320 mg) as a yellow solid. ^{1}H-NMR (400 MHz, $CDCl_3$): δ = 7.69 – 7.66 (m, 2H), 7.60 – 7.56 (m, 2H), 7.50 – 7.46 (m, 2H), 7.14 – 7.10 (m, 2H), 7.04 (d, J = 2.5 Hz, 1H), 6.89 (d, J = 9.0 Hz, 1H), 6.70 (dd, J = 9.0, 2.5 Hz, 1H), 6.21 (s, 1H), 4.19 (q, J = 7.1 Hz, 2H), 3.91 (s, 2H), 3.83 (s, 3H), 2.45 (s, 3H), 2.37 (s, 3H), 1.29 (t, J = 7.1 Hz, 3H). ^{13}C-NMR (100 MHz, $CDCl_3$): δ = 169.0, 168.4, 168.1, 164.2, 157.2, 156.3, 152.8, 139.6, 136.5, 133.9, 131.3, 131.0, 130.5, 129.3, 127.4, 122.0, 115.2, 111.9, 111.8, 106.5, 101.3, 60.5, 55.9, 30.7, 21.1, 14.4, 13.6. HR-MS (EI) m/z calcd for $C_{32}H_{28}ClNO_8$ [M+H$^+$] 590.1576, found 590.1584.

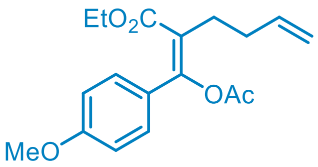

Ethyl (*E*)-2-[acetoxy(4-methoxyphenyl)methylene]hex-5-enoate

The corresponding **ketoester** (3.2 mmol, 1.0 eq) was dissolved in acetic anhydride (6.4 mL), and 4-dimethylaminopyridine (1.6 mmol, 0.5 eq) and Et_3N (24.3 mmol, 7.6 eq) were added.

The reaction mixture was stirred for 3 h at 23 °C. The solvent was evaporated and the residue was taken up in toluene and washed with a saturated solution of $NaHCO_3$, water and brine. The organic phase was dried over Na_2SO_4 and concentrated under reduced pressure. The residue was purified by flash column chromatography (petroleum ether/ EtOAc 15:1) yielded product (49%, 499 mg) as a colorless oil. ^{1}H-NMR (400 MHz, $CDCl_3$): δ = 7.31 – 7.26 (m, 2H), 6.86 – 6.80 (m, 2H), 5.84 (ddt, J = 16.9, 10.2, 6.7 Hz, 1H), 5.05 (dq, J = 17.1, 1.6 Hz, 1H), 5.01 – 4.96 (m, 1H), 4.02 (q, J = 7.1 Hz, 2H), 3.80 (s, 3H), 2.45 (dd, J = 8.9, 6.6 Hz, 2H), 2.27 – 2.19 (m, 2H), 2.16 (s, 3H), 1.01 (t, J = 7.1 Hz, 3H). ^{13}C-NMR (100 MHz, $CDCl_3$): δ = 168.4, 168.3, 160.3, 152.4, 137.7, 129.7, 128.1, 123.4, 115.3, 113.5, 60.8, 55.3, 32.4, 28.4, 21.0, 13.9. HR-MS (EI) m/z calcd for $C_{18}H_{22}O_5$ [M+H$^+$] 319.1540, found 319.1544.

2.6 Characterization Data of Products 4–96

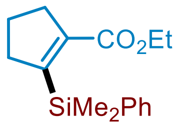

Ethyl 2-[dimethyl(phenyl)silyl]cyclopent-1-ene-1-carboxylate (4)

The general procedure **TP4** was followed using **3a** (0.25 mmol) and **1a** (0.5 mmol) at 23 °C for 2 h. Purification by column chromatography (PE) yielded **4** (55 mg, 81%) as a colorless oil. ^{1}H-NMR (400 MHz, $CDCl_3$): δ = 7.53 – 7.48 (m, 2H), 7.32 (dd, J = 4.2, 2.3 Hz, 3H), 4.00 (q, J = 7.1 Hz, 2H), 2.71 (tt, J = 8.0, 2.5 Hz, 2H), 2.55 (tt, J = 7.6, 2.5 Hz, 2H), 1.89 – 1.81 (m, 2H), 1.09 (t, J = 7.1 Hz, 3H), 0.44 (s, 6H). ^{13}C-NMR (100 MHz, $CDCl_3$): δ = 166.1, 156.0, 145.5, 138.9, 133.7, 128.7, 127.6, 60.0, 41.0, 35.8, 23.5, 14.1, -2.0. HR-MS (EI) m/z calcd for $C_{16}H_{22}O_2Si$ [M+H$^+$] 275.1462, found 275.1466.

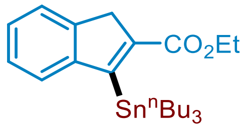

Ethyl 3-(tributylstannyl)-1*H*-indene-2-carboxylate (7)

The general procedure **TP6** was followed using **3b** (0.25 mmol) and **2a** (0.325 mmol) at -20 °C for 15 min. Purification by column chromatography (PE) yielded **7** (79 mg, 66%) as a colorless oil. ^{1}H-NMR (400 MHz, $CDCl_3$): δ = 7.64 (dd, J = 5.8, 2.7 Hz, 1H), 7.51 (dd, J = 5.3, 2.7 Hz, 1H), 7.36 – 7.27 (m, 2H), 4.30 (q, J = 7.1 Hz, 2H), 3.81 – 3.65 (m, 2H), 1.52 (ddd, J = 11.0, 8.1, 3.7 Hz, 6H), 1.39 – 1.29 (m, 9H), 1.24 – 1.10 (m, 6H), 0.87 (t, J = 7.3 Hz, 9H). ^{13}C-NMR

(100 MHz, $CDCl_3$): δ = 166.6, 161.8, 149.9, 145.8, 144.9, 127.0, 126.7, 125.2, 124.0, 60.6, 40.9, 29.3, 27.6, 14.6, 13.8, 11.5. HR-MS (EI) m/z calcd for $C_{24}H_{38}O_2Sn$ [M+H$^+$] 479.1967, found 479.1970.

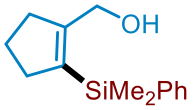

{2-[Dimethyl(phenyl)silyl]cyclopent-1-en-1-yl}methanol (8)

The general procedure **TP9** was followed using **4** (0.219 mmol) and **DIBAH** (0.66 mmol) at 0 °C for 2 h. Purification by column chromatography (petroleum ether/EtOAc 5:1) yielded **8** (38 mg, 74%) as a colorless oil. ^{1}H-NMR (400 MHz, $CDCl_3$): δ = 7.54 – 7.47 (m, 2H), 7.39 – 7.32 (m, 3H), 4.09 (s, 2H), 2.56 – 2.47 (m, 4H), 1.88 – 1.78 (m, 2H), 1.18 (s, 1H), 0.39 (s, 6H). ^{13}C-NMR (100 MHz, $CDCl_3$): δ = 154.6, 139.5, 136.9, 133.7, 129.2, 128.1, 61.8, 39.0, 36.7, 23.8, -1.4. HR-MS (EI) m/z calcd for $C_{14}H_{20}OSi$ [M+H$^+$] 233.1356, found 233.1358.

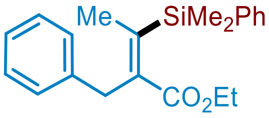

Ethyl (*Z*)-2-benzyl-3-[dimethyl(phenyl)silyl]but-2-enoate (9)

The general procedure **TP5** was followed using **3c** (0.25 mmol) and **1a** (0.5 mmol) at 23 °C for 1.5 h. Purification by column chromatography (PE) yielded **9** (48 mg, 57%) as a colorless oil. ^{1}H-NMR (400 MHz, $CDCl_3$): δ = 7.54 – 7.47 (m, 2H), 7.31 (dd, J = 6.1, 2.6 Hz, 3H), 7.28 – 7.23 (m, 2H), 7.17 (t, J = 6.5 Hz, 3H), 3.82 (s, 2H), 3.77 (q, J = 7.1 Hz, 2H), 1.90 (s, 3H), 0.98 (t, J = 7.1 Hz, 3H), 0.43 (s, 6H). ^{13}C-NMR (100 MHz, $CDCl_3$): δ = 169.2, 147.5, 142.3, 139.7, 139.4, 133.7, 128.6, 128.5, 128.4, 127.7, 126.1, 60.5, 35.9, 20.4, 14.0, -1.0. HR-MS (EI) m/z calcd for $C_{21}H_{26}O_2Si$ [M+H$^+$] 339.1775, found 339.1778.

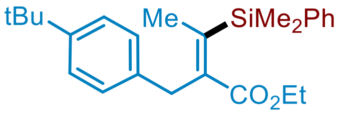

Ethyl (*Z*)-2-[4-(tert-butyl)benzyl]-3-[dimethyl(phenyl)silyl]but-2-enoate (10)

The general procedure **TP5** was followed using **3d** (0.25 mmol) and **1a** (0.5 mmol) at 23 °C for 1 h. Purification by column chromatography (PE) yielded **10** (54 mg, 55%) as a colorless oil. ^{1}H-NMR (400 MHz, $CDCl_3$): δ = 7.54 – 7.49 (m, 2H), 7.34 – 7.30 (m, 3H), 7.28 (d, J = 8.4 Hz, 2H), 7.09 (d, J = 8.4 Hz, 2H), 3.82 – 3.72 (m, 4H), 1.89 (s, 3H), 1.30 (s, 9H), 0.98 (t, J = 7.1

Hz, 3H), 0.43 (s, 6H). ^{13}C-NMR (100 MHz, $CDCl_3$): δ = 169.3, 148.8, 147.1, 142.5, 139.7, 136.1, 133.7, 128.6, 128.0, 127.7, 125.4, 60.5, 35.4, 34.5, 31.5, 20.3, 14.0, -1.0. HR-MS (EI) m/z calcd for $C_{25}H_{34}O_2Si$ [M+H$^+$] 395.2401, found 395.2403.

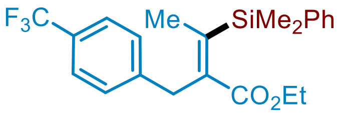

Ethyl (*Z*)-3-[dimethyl(phenyl)silyl]-2-[4-(trifluoromethyl)benzyl]but-2-enoate (11)

The general procedure **TP5** was followed using **3e** (0.25 mmol) and **1a** (0.5 mmol) at 23 °C for 1 h. Purification by column chromatography (PE) yielded **11** (68 mg, 67%) as a colorless oil. ^{1}H-NMR (400 MHz, $CDCl_3$): δ = 7.54 – 7.47 (m, 4H), 7.35 – 7.30 (m, 3H), 7.27 (d, J = 8.0 Hz, 2H), 3.86 (s, 2H), 3.77 (q, J = 7.1 Hz, 2H), 1.89 (s, 3H), 0.98 (t, J = 7.1 Hz, 3H), 0.44 (s, 6H). ^{13}C-NMR (100 MHz, $CDCl_3$): δ = 168.9, 149.0 143.6 (d, $^4J_{C\text{-}F}$ = 1.0 Hz), 141.2, 139.4, 133.7, 128.8, 128.6, 128.2 (q, $^2J_{C\text{-}F}$ = 23.5 Hz), 127.7, 125.5 (q, $^3J_{C\text{-}F}$ = 3.7 Hz), 124.5 (q, $^1J_{C\text{-}F}$ = 270.0 Hz), 60.7, 35.7, 20.6, 13.9, -1.0. ^{19}F-NMR (376 MHz, $CDCl_3$): δ = -62.33 (s). HR-MS (EI) m/z calcd for $C_{22}H_{25}F_3O_2Si$ [M+H$^+$] 407.1649, found 407.1652.

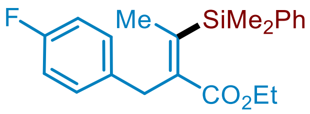

Ethyl (*Z*)-3-[dimethyl(phenyl)silyl]-2-(4-fluorobenzyl)but-2-enoate (12)

The general procedure **TP5** was followed using **3f** (0.25 mmol) and **1a** (0.5 mmol) at 23 °C for 2 h. Purification by column chromatography (PE) yielded **12** (48 mg, 54%) as a colorless oil. ^{1}H-NMR (400 MHz, $CDCl_3$): δ = 7.51 – 7.47 (m, 2H), 7.33 – 7.30 (m, 3H), 7.11 (dd, J = 8.6, 5.5 Hz, 2H), 6.98 – 6.92 (m, 2H), 3.76 (q, J = 7.1 Hz, 4H), 1.89 (s, 3H), 0.98 (t, J = 7.1 Hz, 3H), 0.42 (s, 6H). ^{13}C-NMR (100 MHz, $CDCl_3$): δ = 169.1, 161.5 (d, $^1J_{C\text{-}F}$ = 243.6 Hz), 147.5, 142.2, 139.5, 134.9 (d, $^4J_{C\text{-}F}$ = 3.2 Hz), 133.7, 129.8 (d, $^3J_{C\text{-}F}$ = 8.0 Hz), 128.7, 127.7, 115.2 (d, $^2J_{C\text{-}F}$ = 21.2 Hz), 60.6, 35.1, 20.3, 14.0, -1.0. ^{19}F-NMR (376 MHz, $CDCl_3$): δ = -117.49 (s). HR-MS (EI) m/z calcd for $C_{21}H_{25}FO_2Si$ [M+H$^+$] 357.1681, found 357.1684.

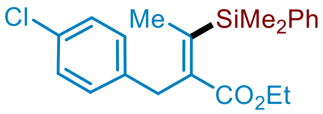

Ethyl (*Z*)-2-(4-chlorobenzyl)-3-[dimethyl(phenyl)silyl]but-2-enoate (13)

The general procedure **TP5** was followed using **3g** (0.25 mmol) and **1a** (0.5 mmol) at 23 °C for

1 h. Purification by column chromatography (PE) yielded **13** (57 mg, 61%) as a colorless oil. ^{1}H-NMR (400 MHz, $CDCl_3$): δ = 7.48 (dt, J = 7.9, 3.2 Hz, 2H), 7.34 – 7.30 (m, 3H), 7.23 (d, J = 8.4 Hz, 2H), 7.09 (d, J = 8.3 Hz, 2H), 3.77 (q, J = 7.3 Hz, 4H), 1.88 (s, 3H), 0.99 (t, J = 7.1 Hz, 3H), 0.42 (s, 6H). ^{13}C-NMR (100 MHz, $CDCl_3$): δ = 169.0, 148.1, 141.8, 139.5, 137.9, 133.7, 131.8, 129.7, 128.7, 128.6, 127.7, 60.6, 35.3, 20.4, 14.0, -1.0. HR-MS (EI) m/z calcd for $C_{21}H_{25}ClO_2Si$ [M+H$^+$] 373.1385, found 373.1388.

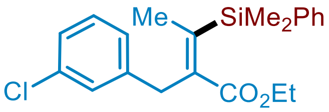

Ethyl (*Z*)-2-(3-chlorobenzyl)-3-[dimethyl(phenyl)silyl]but-2-enoate (14)

The general procedure **TP5** was followed using **3h** (0.25 mmol) and **1a** (0.5 mmol) at 23 °C for 1 h. Purification by column chromatography (PE) yielded **14** (51 mg, 55%) as a colorless oil. ^{1}H-NMR (400 MHz, $CDCl_3$): δ = 7.49 (dd, J = 6.4, 3.0 Hz, 2H), 7.34 – 7.29 (m, 3H), 7.21 – 7.13 (m, 3H), 7.04 (d, J = 7.1 Hz, 1H), 3.78 (q, J = 7.4 Hz, 4H), 1.89 (s, 3H), 0.99 (t, J = 7.1 Hz, 3H), 0.43 (s, 6H). ^{13}C-NMR (100 MHz, $CDCl_3$): δ = 168.9, 148.7, 141.5, 141.4, 139.5, 134.3, 133.6, 129.7, 128.7, 128.6, 127.7, 126.5, 126.3, 60.6, 35.5, 20.5, 14.0, -1.0. HR-MS (EI) m/z calcd for $C_{21}H_{25}ClO_2Si$ [M+H$^+$] 373.1385, found 373.1388.

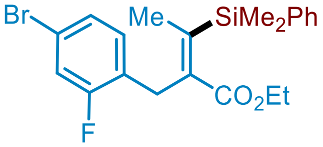

Ethyl (*Z*)-2-(4-bromo-2-fluorobenzyl)-3-[dimethyl(phenyl)silyl]but-2-enoate (15)

The general procedure **TP5** was followed using **3i** (0.25 mmol) and **1a** (0.5 mmol) at 23 °C for 2 h. Purification by column chromatography (PE) yielded **15** (55 mg, 51%) as a colorless oil. ^{1}H-NMR (400 MHz, $CDCl_3$): δ = 7.50 (dd, J = 6.5, 3.0 Hz, 2H), 7.32 (dd, J = 6.1, 2.6 Hz, 3H), 7.11 (t, J = 7.6 Hz, 1H), 7.02 (ddd, J = 15.8, 11.5, 4.7 Hz, 2H), 3.82 (s, 2H), 3.77 (q, J = 7.1 Hz, 2H), 1.88 (s, 3H), 0.98 (t, J = 7.1 Hz, 3H), 0.43 (s, 6H). ^{13}C-NMR (100 MHz, $CDCl_3$): δ = 169.0, 161.1 (d, $^1J_{C\text{-}F}$ = 245.2 Hz), 148.3, 140.8, 139.6, 133.7, 129.8 (d, $^4J_{C\text{-}F}$ = 4.5 Hz), 128.7, 127.8 (d, $^3J_{C\text{-}F}$ = 8.1 Hz), 127.7, 126.2 (d, $^2J_{C\text{-}F}$ = 15.8 Hz), 124.1 (d, $^4J_{C\text{-}F}$ = 3.6 Hz), 115.2 (d, $^2J_{C\text{-}F}$ = 22.2 Hz), 60.6, 28.8 (d, $^3J_{C\text{-}F}$ = 3.5 Hz), 20.2, 13.9, -1.0. ^{19}F-NMR (376 MHz, $CDCl_3$): δ = -117.33 (s). HR-MS (EI) m/z calcd for $C_{21}H_{24}BrFO_2Si$ [M+H$^+$] 435.0786, found 435.0789.

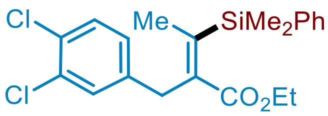

Ethyl (*Z*)-2-(3,4-dichlorobenzyl)-3-[dimethyl(phenyl)silyl]but-2-enoate (16)

The general procedure **TP5** was followed using **3j** (0.25 mmol) and **1a** (0.5 mmol) at 23 °C for 1 h. Purification by column chromatography (PE) yielded **16** (60 mg, 60%) as a colorless oil. ^{1}H-NMR (400 MHz, $CDCl_3$): δ = 7.50 – 7.46 (m, 2H), 7.35 – 7.31 (m, 4H), 7.26 (d, J = 2.0 Hz, 1H), 7.00 (dd, J = 8.2, 2.1 Hz, 1H), 3.78 (dd, J = 14.7, 7.5 Hz, 4H), 1.89 (s, 3H), 1.00 (t, J = 7.1 Hz, 3H), 0.43 (s, 6H). ^{13}C-NMR (100 MHz, $CDCl_3$): δ = 168.8, 149.3, 141.0, 139.8, 139.4, 133.6, 132.4, 130.4, 130.1, 129.7, 128.8, 127.8, 127.8, 60.7, 35.0, 20.6, 14.0, -1.0. HR-MS (EI) m/z calcd for $C_{21}H_{24}Cl_2O_2Si$ [M+H$^+$] 407.0995, found 407.0998.

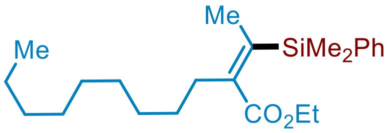

Ethyl (*Z*)-2-{1-[dimethyl(phenyl)silyl]ethylidene}undecanoate (17)

The general procedure **TP5** was followed using **3k** (0.25 mmol) and **1a** (0.5 mmol) at 23 °C for 1 h. Purification by column chromatography (PE) yielded **17** (60 mg, 64%) as a colorless oil. ^{1}H-NMR (400 MHz, $CDCl_3$): δ = 7.51 – 7.45 (m, 2H), 7.32 – 7.28 (m, 3H), 3.82 (q, J = 7.1 Hz, 2H), 2.46 – 2.34 (m, 2H), 1.83 (s, 3H), 1.43 – 1.36 (m, 2H), 1.31 – 1.24 (m, 12H), 1.09 (t, J = 7.1 Hz, 3H), 0.88 (t, J = 6.9 Hz, 3H), 0.38 (s, 6H). ^{13}C-NMR (100 MHz, $CDCl_3$): δ = 170.0, 144.8, 143.4, 139.7, 133.7, 128.6, 127.6, 60.4, 32.1, 30.5, 29.7, 29.7, 29.6, 29.5, 28.7, 22.8, 19.3, 14.3, 14.1, -1.1. HR-MS (EI) m/z calcd for $C_{23}H_{38}O_2Si$ [M+H$^+$] 375.2714, found 375.2716.

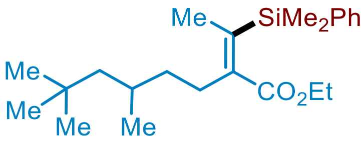

Ethyl (*Z*)-2-{1-[dimethyl(phenyl)silyl]ethylidene}-5,7,7-trimethyloctanoate (18)

The general procedure **TP5** was followed using **3l** (0.25 mmol) and **1a** (0.5 mmol) at 23 °C for 1 h. Purification by column chromatography (PE) yielded **18** (61 mg, 65%) as a colorless oil. ^{1}H-NMR (400 MHz, $CDCl_3$): δ = 7.51 – 7.45 (m, 2H), 7.30 (dd, J = 3.7, 2.6 Hz, 3H), 3.82 (q, J = 7.1 Hz, 2H), 2.46 – 2.31 (m, 2H), 1.84 (s, 3H), 1.48 (td, J = 11.7, 6.1 Hz, 1H), 1.43 – 1.33 (m, 1H), 1.27 – 1.21 (m, 2H), 1.09 (t, J = 7.1 Hz, 3H), 1.07 – 1.01 (m, 1H), 0.94 (d, J = 6.6 Hz, 3H), 0.89 (s, 9H), 0.38 (s, 6H). ^{13}C-NMR (100 MHz, $CDCl_3$): δ = 170.0, 144.8, 143.7, 139.8,

133.7, 128.6, 127.6, 60.4, 51.2, 38.2, 31.2, 30.1, 29.6, 28.4, 22.7, 19.3, 14.1, -1.0. HR-MS (EI) m/z calcd for $C_{23}H_{38}O_2Si$ [M+H$^+$] 375.2714, found 375.2717.

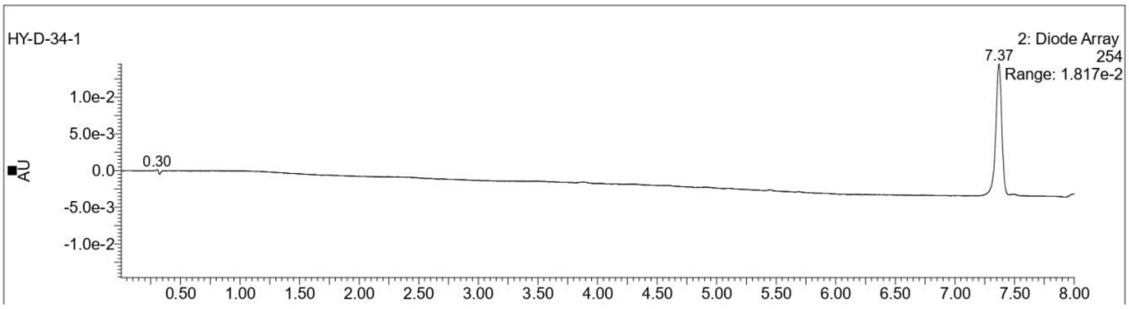

Supplementary Figure 5. HPLC data of compond **18**.

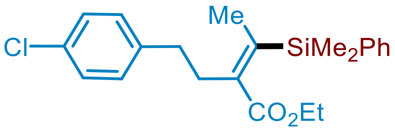

Ethyl (*Z*)-2-(4-chlorophenethyl)-3-[dimethyl(phenyl)silyl]but-2-enoate (19)

The general procedure **TP5** was followed using **corresponding alkenyl acetate** (0.25 mmol) and **1a** (0.5 mmol) at 23 °C for 2 h. Purification by column chromatography (petroleum ether/EtOAc 100:1) yielded **19** (51 mg, 52%) as a colorless oil. ^{1}H-NMR (400 MHz, $CDCl_3$): δ = 7.52 – 7.48 (m, 2H), 7.38 – 7.34 (m, 3H), 7.13 (d, J = 8.4 Hz, 2H), 6.72 (d, J = 8.4 Hz, 2H), 4.25 (q, J = 7.1 Hz, 2H), 2.51 – 2.44 (m, 2H), 2.41 – 2.35 (m, 2H), 1.92 (s, 3H), 1.34 (t, J = 7.1 Hz, 3H), 0.39 (s, 6H). ^{13}C-NMR (100 MHz, $CDCl_3$): δ = 169.8, 143.7, 139.8, 138.8, 138.5, 133.9, 131.7, 129.8, 129.4, 128.4, 128.2, 60.5, 36.3, 34.1, 20.9, 14.6, -1.1. HR-MS (EI) m/z calcd for $C_{22}H_{27}ClO_2Si$ [M+H$^+$] 387.1542, found 387.1544.

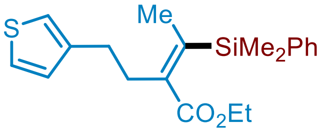

Ethyl (*Z*)-3-[dimethyl(phenyl)silyl]-2-[2-(thiophen-3-yl)ethyl]but-2-enoate (20)

The general procedure **TP5** was followed using **3m** (0.25 mmol) and **1a** (0.5 mmol) at 23 °C for 1 h. Purification by column chromatography (petroleum ether/EtOAc 100:1) yielded **20** (45 mg, 50%) as a colorless oil. ^{1}H-NMR (400 MHz, $CDCl_3$): δ = 7.46 (dt, J = 5.0, 2.7 Hz, 2H), 7.33 – 7.29 (m, 3H), 7.22 (dd, J = 4.9, 3.0 Hz, 1H), 6.94 (t, J = 3.5 Hz, 2H), 3.82 (q, J = 7.1 Hz, 2H), 2.79 – 2.69 (m, 4H), 1.71 (s, 3H), 1.09 (t, J = 7.1 Hz, 3H), 0.38 (s, 6H). ^{13}C-NMR (100 MHz, $CDCl_3$): δ = 169.6, 145.7, 143.0, 141.9, 139.6, 133.7, 128.6, 128.6, 127.6, 125.3, 120.7,

60.5, 31.4, 29.2, 19.4, 14.1, -1.1. HR-MS (EI) m/z calcd for $C_{20}H_{26}SO_2Si$ [M+H$^+$] 359.1496, found 359.1499.

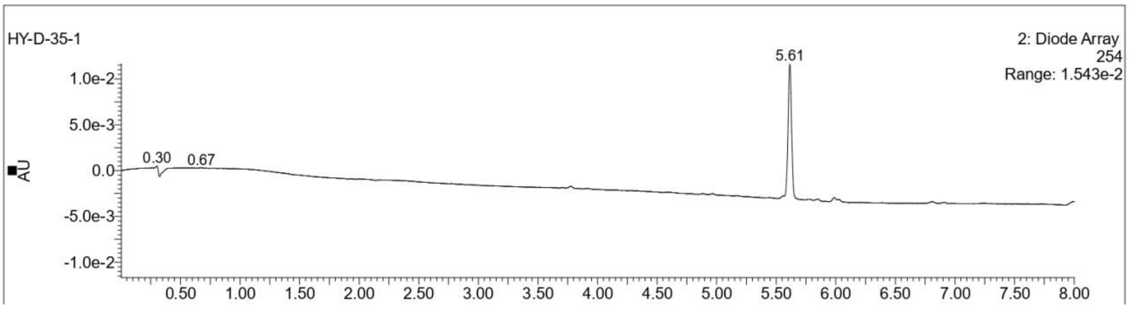

Supplementary Figure 6. HPLC data of 20.

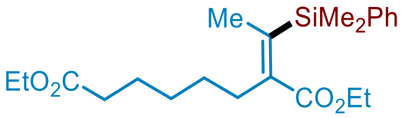

Diethyl (*Z*)-2-{1-[dimethyl(phenyl)silyl]ethylidene}octanedioate (21)

The general procedure **TP4** was followed using **3n** (0.25 mmol) and **1a** (0.5 mmol) at 23 °C for 1 h. Purification by column chromatography (petroleum ether/EtOAc 20:1) yielded **21** (53 mg, 54%) as a colorless oil. ^{1}H-NMR (400 MHz, $CDCl_3$): δ = 7.47 (dd, J = 6.6, 2.9 Hz, 2H), 7.33 – 7.28 (m, 3H), 4.12 (q, J = 7.1 Hz, 2H), 3.82 (q, J = 7.1 Hz, 2H), 2.45 – 2.36 (m, 2H), 2.29 (t, J = 7.5 Hz, 2H), 1.83 (s, 3H), 1.69 – 1.62 (m, 2H), 1.47 – 1.33 (m, 4H), 1.25 (t, J = 7.1 Hz, 3H), 1.09 (t, J = 7.1 Hz, 3H), 0.38 (s, 6H). ^{13}C-NMR (100 MHz, $CDCl_3$): δ = 173.9, 169.9, 144.3, 144.0, 139.6, 133.7, 128.6, 127.6, 60.4, 60.3, 34.4, 30.2, 29.2, 28.4, 24.9, 19.4, 14.4, 14.1, -1.1. HR-MS (EI) m/z calcd for $C_{22}H_{34}O_4Si$ [M+H$^+$] 391.2299, found 391.2302.

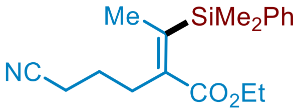

Ethyl (*Z*)-5-cyano-2-{1-[dimethyl(phenyl)silyl]ethylidene}pentanoate (22)

The general procedure **TP4** was followed using (*Z*)-**87** (0.25 mmol) and **1a** (0.5 mmol) at 23 °C for 1 h. Purification by column chromatography (petroleum ether/EtOAc 20:1) yielded **22** (55 mg, 70%) as a colorless oil. ^{1}H-NMR (400 MHz, $CDCl_3$): δ = 7.46 (dd, J = 6.4, 3.0 Hz, 2H), 7.31 (dd, J = 6.3, 2.7 Hz, 3H), 3.84 (q, J = 7.1 Hz, 2H), 2.62 – 2.52 (m, 2H), 2.36 (t, J = 7.1 Hz, 2H), 1.89 (s, 3H), 1.80 (m, 2H), 1.10 (t, J = 7.1 Hz, 3H), 0.40 (s, 6H). ^{13}C-NMR (100 MHz, $CDCl_3$): δ = 169.2, 147.5, 141.6, 139.3, 133.6, 128.8, 127.7, 119.7, 60.7, 29.0, 24.6, 19.8, 17.0, 14.1, -1.1. HR-MS (EI) m/z calcd for $C_{18}H_{25}NO_2Si$ [M+H$^+$] 316.1727, found 316.1730.

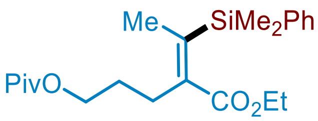

Ethyl (*Z*)-2-{1-[dimethyl(phenyl)silyl]ethylidene}-5-(pivaloyloxy)pentanoate (23)

The general procedure **TP5** was followed using **3o** (0.25 mmol) and **1a** (0.5 mmol) at 23 °C for 2 h. Purification by column chromatography (petroleum ether/EtOAc 50:1) yielded **23** (55 mg, 56%) as a colorless oil. ^{1}H-NMR (400 MHz, $CDCl_3$): δ = 7.50 – 7.44 (m, 2H), 7.33 – 7.29 (m, 3H), 4.07 (t, J = 6.3 Hz, 2H), 3.84 (q, J = 7.1 Hz, 2H), 2.53 – 2.44 (m, 2H), 1.85 (s, 3H), 1.75 (dq, J = 12.9, 6.3 Hz, 2H), 1.20 (s, 9H), 1.09 (t, J = 7.1 Hz, 3H), 0.39 (s, 6H). ^{13}C-NMR (100 MHz, $CDCl_3$): δ = 178.7, 169.5, 145.8, 143.1, 139.6, 133.7, 128.6, 127.7, 64.0, 60.5, 38.9, 28.0, 27.4, 26.8, 19.5, 14.1, -1.1. HR-MS (EI) m/z calcd for $C_{22}H_{34}O_4Si$ [M+H$^+$] 391.2299, found 391.2301.

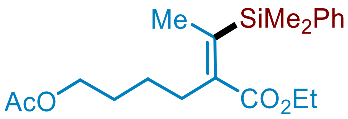

Ethyl (*Z*)-6-acetoxy-2-{1-[dimethyl(phenyl)silyl]ethylidene}hexanoate (24)

The general procedure **TP4** was followed using **corresponding alkenyl acetate** (0.25 mmol) and **1a** (0.5 mmol) at 23 °C for 1 h. Purification by column chromatography (petroleum ether/EtOAc 20:1) yielded **24** (52 mg, 58%) as a colorless oil. ^{1}H-NMR (400 MHz, $CDCl_3$): δ = 7.50 – 7.44 (m, 2H), 7.33 – 7.28 (m, 3H), 4.07 (t, J = 6.6 Hz, 2H), 3.82 (q, J = 7.1 Hz, 2H), 2.49 – 2.39 (m, 2H), 2.04 (s, 3H), 1.84 (s, 3H), 1.71 – 1.62 (m, 2H), 1.48 (tt, J = 10.1, 6.4 Hz, 2H), 1.09 (t, J = 7.1 Hz, 3H), 0.38 (s, 6H). ^{13}C-NMR (100 MHz, $CDCl_3$): δ = 171.3, 169.8, 144.6, 143.9, 139.6, 133.7, 128.6, 127.7, 64.4, 60.5, 29.9, 28.6, 25.1, 21.1, 19.5, 14.1, -1.1. HR-MS (EI) m/z calcd for $C_{20}H_{30}O_4Si$ [M+H$^+$] 363.1986, found 363.1989.

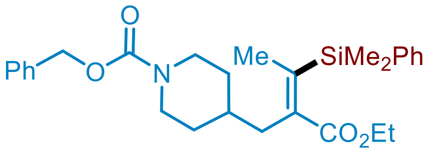

Benzyl (*Z*)-4-{3-[dimethyl(phenyl)silyl]-2-(ethoxycarbonyl)but-2-en-1-yl}piperidine-1-carboxylate (25)

The general procedure **TP5** was followed using **3p** (0.25 mmol) and **1a** (0.5 mmol) at 23 °C for 5 h. Purification by column chromatography (petroleum ether/EtOAc 15:1) yielded **25** (60 mg, 50%) as a colorless oil. ^{1}H-NMR (400 MHz, $CDCl_3$): δ = 7.46 (dd, J = 6.4, 3.0 Hz, 2H), 7.35

(d, J = 4.2 Hz, 4H), 7.34 – 7.27 (m, 4H), 5.12 (s, 2H), 4.16 (s, 2H), 3.80 (q, J = 7.1 Hz, 2H), 2.72 (s, 2H), 2.39 (d, J = 7.1 Hz, 2H), 1.82 (s, 3H), 1.72 – 1.53 (m, 4H), 1.15 (d, J = 9.5 Hz, 1H), 1.08 (t, J = 7.1 Hz, 3H), 0.38 (s, 6H). ^{13}C-NMR (100 MHz, $CDCl_3$): δ = 169.9, 155.4, 145.6, 142.3, 139.5, 137.1, 133.7, 128.7, 128.6, 128.1, 128.0, 127.7, 67.1, 60.5, 44.4, 36.8, 36.1, 32.1, 20.0, 14.1, -1.0. HR-MS (EI) m/z calcd for $C_{28}H_{37}NO_4Si$ [M+H$^+$] 480.2565, found 480.2567.

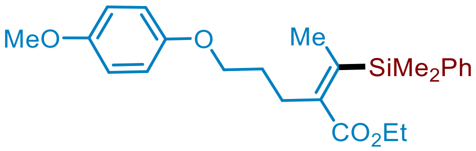

Ethyl (*Z*)-2-{1-[dimethyl(phenyl)silyl]ethylidene}-5-(4-methoxyphenoxy)pentanoate (26)

The general procedure **TP4** was followed using **3q** (0.25 mmol) and **1a** (0.5 mmol) at 23 °C for 0.5 h. Purification by column chromatography (petroleum ether/EtOAc 50:1) yielded **26** (59 mg, 57%) as a colorless oil. ^{1}H-NMR (400 MHz, $CDCl_3$): δ = 7.50 – 7.42 (m, 2H), 7.35 – 7.27 (m, 3H), 6.81 (s, 4H), 3.91 (t, J = 6.3 Hz, 2H), 3.81 (q, J = 7.1 Hz, 2H), 3.76 (s, 3H), 2.67 – 2.53 (m, 2H), 1.89 (dd, J = 8.6, 6.4 Hz, 2H), 1.85 (s, 3H), 1.07 (t, J = 7.1 Hz, 3H), 0.38 (s, 6H). ^{13}C-NMR (100 MHz, $CDCl_3$): δ = 169.7, 153.8, 153.2, 145.5, 143.3, 139.6, 133.7, 128.6, 127.7, 115.5, 114.8, 67.8, 60.5, 55.9, 28.4, 26.8, 19.5, 14.1, -1.0. HR-MS (EI) m/z calcd for $C_{24}H_{32}O_4Si$ [M+H$^+$] 413.2143, found 413.2145.

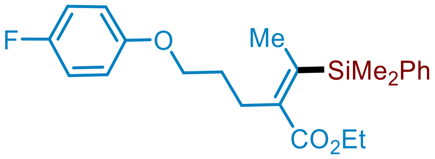

Ethyl (*Z*)-2-{1-[dimethyl(phenyl)silyl]ethylidene}-5-(4-fluorophenoxy)pentanoate (**27**)

The general procedure **TP4** was followed using **3r** (0.25 mmol) and **1a** (0.5 mmol) at 23 °C for 1 h. Purification by column chromatography (petroleum ether/EtOAc 100:1) yielded **27** (55 mg, 55%) as a colorless oil. ^{1}H-NMR (400 MHz, $CDCl_3$): δ = 7.48 – 7.43 (m, 2H), 7.32 – 7.27 (m, 3H), 6.98 – 6.91 (m, 2H), 6.84 – 6.77 (m, 2H), 3.91 (t, J = 6.2 Hz, 2H), 3.81 (q, J = 7.1 Hz, 2H), 2.67 – 2.56 (m, 2H), 1.91 (dt, J = 7.3, 6.4 Hz, 2H), 1.85 (s, 3H), 1.07 (t, J = 7.1 Hz, 3H), 0.38 (s, 6H). ^{13}C-NMR (100 MHz, $CDCl_3$): δ = 169.7, 157.3 (d, $^1J_{C\text{-}F}$ = 237.8 Hz), 155.2 (d, $^4J_{C\text{-}F}$ = 2.0 Hz), 145.6, 143.2, 139.6, 133.7, 128.6, 127.7, 115.9 (d, $^2J_{C\text{-}F}$ = 23.2 Hz), 115.5 (d, $^3J_{C\text{-}F}$ = 8.0 Hz), 67.8, 60.5, 28.3, 26.7, 19.5, 14.1, -1.1. ^{19}F-NMR (376 MHz, $CDCl_3$): δ = -124.32 (s). HR-MS (EI) m/z calcd for $C_{23}H_{29}FO_3Si$ [M+H$^+$] 401.1943 found 401.1945.

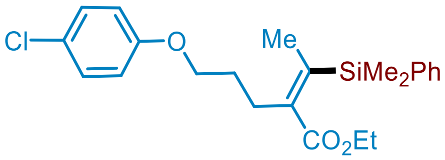

Ethyl (*Z*)-5-(4-chlorophenoxy)-2-{1-[dimethyl(phenyl)silyl]ethylidene}pentanoate (28)

The general procedure **TP4** was followed using **3s** (0.25 mmol) and **1a** (0.5 mmol) at 23 °C for 1 h. Purification by column chromatography (petroleum ether/EtOAc 50:1) yielded **28** (90 mg, 86%) as a colorless oil. ^{1}H-NMR (400 MHz, $CDCl_3$): δ = 7.46 (dd, J = 6.5, 3.0 Hz, 2H), 7.30 (dd, J = 4.8, 1.8 Hz, 3H), 7.23 – 7.18 (m, 2H), 6.83 – 6.74 (m, 2H), 3.92 (t, J = 6.2 Hz, 2H), 3.80 (q, J = 7.1 Hz, 2H), 2.61 (t, J = 7.5 Hz, 2H), 1.96 – 1.86 (m, 2H), 1.85 (s, 3H), 1.07 (t, J = 7.1 Hz, 3H), 0.38 (s, 6H). ^{13}C-NMR (100 MHz, $CDCl_3$): δ = 169.6, 157.7, 145.7, 143.1, 139.5, 133.7, 129.4, 128.6, 127.7, 125.6, 115.9, 67.5, 60.5, 28.2, 26.7, 19.5, 14.1, -1.1. HR-MS (EI) m/z calcd for $C_{23}H_{29}ClO_3Si$ [M+H$^+$] 417.1647, found 417.1649.

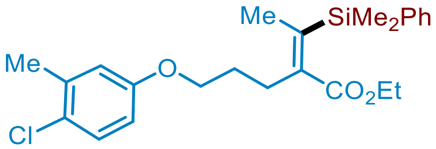

Ethyl (*Z*)-5-(4-chloro-3-methylphenoxy)-2-{1-[dimethyl(phenyl)silyl]ethylidene}pentanoate (29)

The general procedure **TP4** was followed using **3t** (0.25 mmol) and **1a** (0.5 mmol) at 23 °C for 1 h. Purification by column chromatography (petroleum ether/EtOAc 50:1) yielded **29** (77 mg, 71%) as a colorless oil. ^{1}H-NMR (400 MHz, $CDCl_3$): δ = 7.46 (dd, J = 6.4, 3.0 Hz, 2H), 7.31 – 7.29 (m, 3H), 7.19 (d, J = 8.7 Hz, 1H), 6.74 (d, J = 2.8 Hz, 1H), 6.64 (dd, J = 8.7, 2.9 Hz, 1H), 3.91 (t, J = 6.2 Hz, 2H), 3.81 (q, J = 7.1 Hz, 2H), 2.60 (t, J = 7.5 Hz, 2H), 2.32 (s, 3H), 1.93 – 1.86 (m, 2H), 1.85 (s, 3H), 1.07 (t, J = 7.1 Hz, 3H), 0.38 (s, 6H). ^{13}C-NMR (100 MHz, $CDCl_3$): δ = 169.7, 157.6, 145.7, 143.1, 139.6, 137.1, 133.7, 129.7, 128.6, 127.7, 125.8, 117.2, 113.1, 67.4, 60.5, 28.2, 26.7, 20.5, 19.6, 14.1, -1.0. HR-MS (EI) m/z calcd for $C_{24}H_{31}ClO_3Si$ [M+H$^+$] 431.1804, found 431.1808.

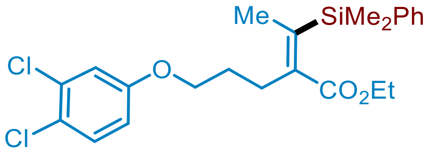

Ethyl (*Z*)-5-(3,4-dichlorophenoxy)-2-{1-[dimethyl(phenyl)silyl]ethylidene}pentanoate (30)

The general procedure **TP4** was followed using **3u** (0.25 mmol) and **1a** (0.5 mmol) at 23 °C for

1 h. Purification by column chromatography (petroleum ether/EtOAc 50:1) yielded **30** (59 mg, 52%) as a colorless oil. ^{1}H-NMR (400 MHz, $CDCl_3$): δ = 7.46 (dd, J = 6.4, 3.0 Hz, 2H), 7.30 (dd, J = 7.3, 3.7 Hz, 4H), 6.96 (d, J = 2.8 Hz, 1H), 6.72 (dd, J = 8.9, 2.8 Hz, 1H), 3.92 (t, J = 6.2 Hz, 2H), 3.81 (q, J = 7.1 Hz, 2H), 2.60 (t, J = 7.5 Hz, 2H), 1.95 – 1.86 (m, 2H), 1.85 (s, 3H), 1.07 (t, J = 7.1 Hz, 3H), 0.39 (s, 6H). ^{13}C-NMR (100 MHz, $CDCl_3$): δ = 169.6, 158.1, 145.9, 142.9, 139.5, 133.6, 132.9, 130.8, 128.7, 127.7, 123.9, 116.4, 114.7, 67.8, 60.6, 28.1, 26.6, 19.6, 14.1, -1.1. HR-MS (EI) m/z calcd for $C_{23}H_{28}Cl_2O_3Si$ [M+H$^+$] 451.1258, found 451.1260.

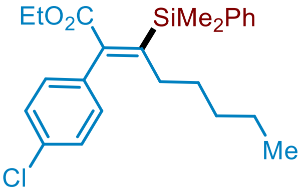

Ethyl (*Z*)-2-(4-chlorophenyl)-3-[dimethyl(phenyl)silyl]oct-2-enoate (31)

The general procedure **TP4** was followed using **3v** (0.25 mmol) and **1a** (0.5 mmol) at 23 °C for 1 h. Purification by column chromatography (petroleum ether/EtOAc 50:1) yielded **31** (64 mg, 62%) as a colorless oil. ^{1}H-NMR (400 MHz, $CDCl_3$): δ = 7.59 – 7.52 (m, 2H), 7.36 – 7.29 (m, 5H), 7.15 – 7.08 (m, 2H), 3.75 (q, J = 7.1 Hz, 2H), 2.05 (dd, J = 9.4, 6.8 Hz, 2H), 1.22 – 1.15 (m, 2H), 1.08 – 1.01 (m, 2H), 1.01 – 0.92 (m, 5H), 0.73 (t, J = 7.2 Hz, 3H), 0.50 (s, 6H). ^{13}C-NMR (100 MHz, $CDCl_3$): δ = 168.3, 153.9, 143.7, 139.5, 137.0, 133.9, 133.1, 130.6, 128.7, 128.4, 127.6, 60.9, 34.2, 31.9, 29.4, 22.2, 14.0, 13.9, -0.5. HR-MS (EI) m/z calcd for $C_{24}H_{31}ClO_2Si$ [M+H$^+$] 415.1855 found 415.1858.

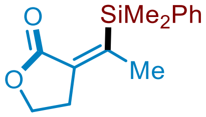

(*Z*)-3-{1-[Dimethyl(phenyl)silyl]ethylidene}dihydrofuran-2(3*H*)-one (32)

The general procedure **TP5** was followed using **3w** (0.25 mmol) and **1a** (0.5 mmol) at 23 °C for 1 h. Purification by column chromatography (petroleum ether/EtOAc 100:1) yielded **32** (50 mg, 81%) as a colorless oil. ^{1}H-NMR (400 MHz, $CDCl_3$): δ = 7.58 – 7.51 (m, 2H), 7.36 – 7.30 (m, 3H), 4.37 (t, J = 7.5 Hz, 2H), 2.98 – 2.81 (m, 2H), 1.86 (t, J = 1.9 Hz, 3H), 0.52 (s, 6H). ^{13}C-NMR (100 MHz, $CDCl_3$): δ = 170.2, 153.7, 139.0, 134.6, 134.1, 128.9, 127.7, 65.4, 28.4, 23.5, -2.0. HR-MS (EI) m/z calcd for $C_{14}H_{18}O_2Si$ [M+H$^+$] 247.1149 found 247.1151.

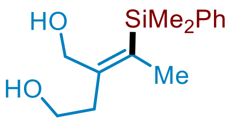

(*Z*)-2-[1-[dimethyl(phenyl)silyl]ethylidene]butane-1,4-diol (33)

The general procedure **TP10** was followed using **32** (0.56 g, 2.2 mmol) and **$LiAlH_4$** (0.125 g, 3.3 mmol) at 0 °C for 1 h. Purification by column chromatography (petroleum ether/EtOAc 2:1) yielded **33** (0.23 g, 41%) as a white solid. ^{1}H-NMR (400 MHz, $CDCl_3$): δ = 7.53 – 7.48 (m, 2H), 7.35 (dt, *J* = 4.1, 1.7 Hz, 3H), 4.01 – 3.93 (m, 2H), 3.71 (t, *J* = 5.9 Hz, 2H), 2.56 (t, *J* = 5.9 Hz, 4H), 1.82 (s, 3H), 0.42 (s, 6H). ^{13}C-NMR (100 MHz, $CDCl_3$): δ = 147.9, 140.2, 135.1, 133.6, 129.2, 128.2, 66.4, 61.6, 35.5, 18.7, -0.4. HR-MS (EI) m/z calcd for $C_{14}H_{22}O_2Si$ [$M+H^+$] 251.1462 found 251.1464.

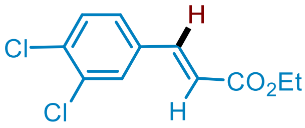

Ethyl (*E*)-3-(3,4-dichlorophenyl)acrylate (34)

The general procedure **TP4** was followed using **3aj** (0.25 mmol) and **1a** (0.5 mmol) at 23 °C for 30 min. Purification by column chromatography (petroleum ether/EtOAc 100:1) yielded **34** (47 mg, 77%) as a colorless oil. ^{1}H-NMR (400 MHz, $CDCl_3$): δ = 7.60 (d, *J* = 2.0 Hz, 1H), 7.57 (d, *J* = 16.1 Hz, 1H), 7.46 (d, *J* = 8.3 Hz, 1H), 7.34 (dd, *J* = 8.4, 2.0 Hz, 1H), 6.42 (d, *J* = 16.0 Hz, 1H), 4.27 (q, *J* = 7.1 Hz, 2H), 1.34 (t, *J* = 7.1 Hz, 3H). HR-MS (EI) m/z calcd for $C_{11}H_{10}Cl_2O_2$ [$M+H^+$] 245.0131 found 245.0133.

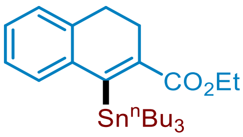

Ethyl 1-(tributylstannyl)-3,4-dihydronaphthalene-2-carboxylate (35)

The general procedure **TP7** was followed using **3x** (0.25 mmol) and **2a** (0.325 mmol) at 0 ℃ for 15 min. Purification by column chromatography (PE) yielded **35** (86 mg, 70%) as a colorless oil. ^{1}H-NMR (400 MHz, $CDCl_3$): δ = 7.20 (dt, *J* = 3.8, 2.2 Hz, 2H), 7.19 – 7.12 (m, 2H), 4.25 (q, *J* = 7.1 Hz, 2H), 2.78 – 2.62 (m, 2H), 2.59 – 2.41 (m, 2H), 1.51 – 1.41 (m, 6H), 1.34 – 1.24 (m, 9H), 1.06 – 0.93 (m, 6H), 0.85 (t, *J* = 7.3 Hz, 9H). ^{13}C-NMR (100 MHz, $CDCl_3$): δ = 168.8, 159.2, 139.9, 139.2, 137.2, 130.4, 128.0, 127.2, 126.2, 61.1, 29.4, 29.0, 27.6, 24.1, 14.5, 13.9, 13.4. HR-MS (EI) m/z calcd for $C_{25}H_{40}O_2Sn$ [$M+H^+$] 493.2123, found 493.2125.

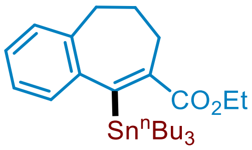

Ethyl 9-(tributylstannyl)-6,7-dihydro-5*H*-benzo[7]annulene-8-carboxylate (36)

The general procedure **TP7** was followed using **3y** (0.25 mmol) and **2a** (0.325 mmol) at 0 ℃ for 50 min. Purification by column chromatography (petroleum ether/EtOAc 50:1) yielded **36** (60 mg, 47%) as a colorless oil. ^{1}H-NMR (400 MHz, $CDCl_3$): δ = 7.22 (td, J = 7.3, 1.9 Hz, 1H), 7.17 – 7.09 (m, 2H), 6.92 (d, J = 7.4 Hz, 1H), 4.26 (q, J = 7.1 Hz, 2H), 2.45 (t, J = 6.6 Hz, 2H), 2.13 – 2.05 (m, 2H), 1.34 (m, 10H), 1.22 (dq, J = 14.5, 7.1 Hz, 7H), 0.97 – 0.84 (m, 6H), 0.82 (t, J = 7.3 Hz, 9H). ^{13}C-NMR (100 MHz, $CDCl_3$): δ = 169.3, 164.8, 143.8, 140.6, 138.4, 128.8, 128.4, 126.6, 125.6, 61.0, 34.3, 31.5, 29.3, 27.6, 26.5, 14.5, 13.8, 12.5. HR-MS (EI) m/z calcd for $C_{26}H_{42}O_2Sn$ [M+H$^+$] 507.2280, found 507.2283.

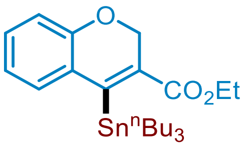

Ethyl 4-(tributylstannyl)-2*H*-chromene-3-carboxylate (37)

The general procedure **TP7** was followed using **3z** (0.25 mmol) and **2a** (0.325 mmol) at 0 ℃ for 1 h. Purification by column chromatography (PE) yielded **37** (71 mg, 58%) as a colorless oil. ^{1}H-NMR (400 MHz, $CDCl_3$): δ = 7.28 – 7.24 (m, 1H), 7.19 (td, J = 7.8, 1.5 Hz, 1H), 7.04 – 6.88 (m, 2H), 4.81 – 4.72 (m, 2H), 4.27 (q, J = 7.1 Hz, 2H), 1.54 – 1.39 (m, 6H), 1.32 (ddd, J = 19.1, 7.1, 5.6 Hz, 9H), 1.13 – 0.97 (m, 6H), 0.86 (t, J = 7.3 Hz, 9H). ^{13}C-NMR (100 MHz, $CDCl_3$): δ = 166.4, 156.2, 154.4, 133.9, 131.1, 130.5, 128.7, 121.6, 116.4, 64.6, 61.2, 29.3, 27.5, 14.5, 13.8, 13.4. HR-MS (EI) m/z calcd for $C_{24}H_{38}O_3Sn$ [M+H$^+$] 495.1916, found 495.1918.

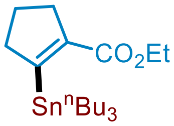

Ethyl 2-(tributylstannyl)cyclopent-1-ene-1-carboxylate (38)

The general procedure **TP7** was followed using **3a** (0.25 mmol) and **2a** (0.325 mmol) at 0 ℃ for 20 min. Purification by column chromatography (PE) yielded **38** (92 mg, 85%) as a colorless oil. ^{1}H-NMR (400 MHz, $CDCl_3$): δ = 4.19 (q, J = 7.1 Hz, 2H), 2.61 (t, J = 7.5 Hz, 4H), 1.98 – 1.82 (m, 2H), 1.60 – 1.42 (m, 6H), 1.42 – 1.18 (m, 9H), 1.00 – 0.93 (m, 6H), 0.88 (t, J = 7.3 Hz, 9H). ^{13}C-NMR (100 MHz, $CDCl_3$): δ = 166.7, 166.1, 143.9, 60.2, 41.8, 33.6, 29.4, 27.5,

24.6, 14.8, 13.8, 10.6. HR-MS (EI) m/z calcd for $C_{20}H_{38}O_2Sn$ [M+H$^+$] 431.1967, found 431.1969.

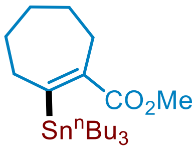

Methyl 2-(tributylstannyl)cyclohept-1-ene-1-carboxylate (39)

The general procedure **TP7** was followed using **corresponding alkenyl acetate** (0.25 mmol) and **2a** (0.325 mmol) at 0 ℃ for 30 min. Purification by column chromatography (PE) yielded **39** (67 mg, 60%) as a colorless oil. ^{1}H-NMR (400 MHz, $CDCl_3$): δ = 3.71 (s, 3H), 2.70 – 2.60 (m, 2H), 2.60 – 2.40 (m, 2H), 1.80 (dt, J = 11.9, 6.0 Hz, 2H), 1.53 – 1.37 (m, 10H), 1.29 (dd, J = 14.7, 7.3 Hz, 6H), 0.98 – 0.81 (m, 15H). ^{13}C-NMR (100 MHz, $CDCl_3$): δ = 170.1, 169.9, 144.3, 52.0, 35.3, 32.8, 29.4, 28.8, 27.6, 26.4, 25.0, 13.9, 11.7. HR-MS (EI) m/z calcd for $C_{21}H_{40}O_2Sn$ [M+H$^+$] 445.2123, found 445.2125.

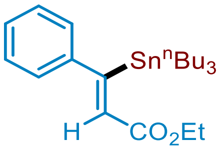

Ethyl (*Z*)-3-phenyl-3-(tributylstannyl)acrylate (40)

The general procedure **TP6** was followed using **3aa** (0.25 mmol) and **2a** (0.325 mmol) at -20 ℃ for 15 min. Purification by column chromatography (PE) yielded **40** (73 mg, 63%) as a colorless oil. ^{1}H-NMR (400 MHz, $CDCl_3$): δ = 7.38 – 7.25 (m, 2H), 7.26 – 7.18 (m, 1H), 7.09 – 7.00 (m, 2H), 6.67 – 6.30 (m, 1H), 4.24 (q, J = 7.1 Hz, 2H), 1.48 – 1.35 (m, 6H), 1.33 – 1.21 (m, 9H), 0.97 – 0.92 (m, 6H), 0.83 (t, J = 7.3 Hz, 9H). ^{13}C-NMR (100 MHz, $CDCl_3$): δ = 173.8, 167.9, 145.9, 130.8, 128.2, 126.8, 126.3, 60.6, 29.2, 27.5, 14.5, 13.8, 12.1. HR-MS (EI) m/z calcd for $C_{23}H_{38}O_2Sn$ [M+H$^+$] 467.1967, found 467.1970.

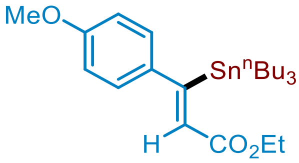

Ethyl (*Z*)-3-(4-methoxyphenyl)-3-(tributylstannyl)acrylate (41)

The general procedure **TP6** was followed using **3ab** (0.25 mmol) and **2a** (0.325 mmol) at -20 ℃ for 15 min. Purification by column chromatography (PE) yielded **41** (73 mg, 59%) as a

colorless oil. ^{1}H-NMR (400 MHz, $CDCl_3$): δ = 7.06 – 6.98 (m, 2H), 6.89 – 6.82 (m, 2H), 6.46 (s, 1H), 4.23 (q, J = 7.1 Hz, 2H), 3.81 (s, 3H), 1.51 – 1.34 (m, 6H), 1.33 – 1.20 (m, 9H), 1.04 – 0.88 (m, 6H), 0.84 (t, J = 7.3 Hz, 9H). ^{13}C-NMR (100 MHz, $CDCl_3$): δ = 173.1, 168.1, 159.1, 138.3, 129.9, 127.9, 113.7, 60.5, 55.5, 29.2, 27.5, 14.5, 13.8, 12.2. HR-MS (EI) m/z calcd for $C_{24}H_{40}O_3Sn$ [M+H$^+$] 497.2072, found 497.2075.

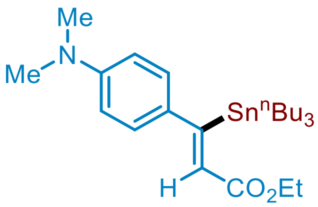

Ethyl (*Z*)-3-[4-(dimethylamino)phenyl]-3-(tributylstannyl)acrylate (42)

The general procedure **TP6** was followed using **3ac** (0.25 mmol) and **2a** (0.325 mmol) at 23 ℃ for 4.5 h. Purification by column chromatography (petroleum ether/EtOAc 50:1) yielded **42** (89 mg, 70%) as a yellow oil. ^{1}H-NMR (400 MHz, $CDCl_3$): δ = 7.10 – 7.00 (m, 2H), 6.68 (d, J = 8.9 Hz, 2H), 6.47 (s, 1H), 4.22 (q, J = 7.1 Hz, 2H), 2.96 (s, 6H), 1.52 – 1.38 (m, 6H), 1.36 – 1.20 (m, 9H), 1.00 – 0.94 (m, 6H), 0.84 (t, J = 7.3 Hz, 9H). ^{13}C-NMR (100 MHz, $CDCl_3$): δ = 173.3, 168.3, 150.1, 133.5, 128.2, 128.0, 112.1, 60.3, 40.6, 29.3, 27.5, 14.5, 13.9, 12.4. HR-MS (EI) m/z calcd for $C_{25}H_{43}NO_2Sn$ [M+H$^+$] 510.2389, found 510.2392.

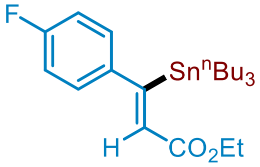

Ethyl (*Z*)-3-(4-fluorophenyl)-3-(tributylstannyl)acrylate (43)

The general procedure **TP6** was followed using **3ad** (0.25 mmol) and **2a** (0.325 mmol) at -20 ℃ for 15 min. Purification by column chromatography (PE) yielded **43** (94 mg, 78%) as a colorless oil. ^{1}H-NMR (400 MHz, $CDCl_3$): δ = 7.05 – 6.97 (m, 4H), 6.45 (s, 1H), 4.24 (q, J = 7.1 Hz, 2H), 1.51 – 1.35 (m, 6H), 1.35 – 1.16 (m, 9H), 0.98 – 0.92 (m, 6H), 0.84 (t, J = 7.3 Hz, 9H). ^{13}C-NMR (100 MHz, $CDCl_3$): δ = 172.7, 167.8, 162.1 (d, $^1J_{C\text{-}F}$ = 245.8 Hz), 141.9 (d, $^4J_{C\text{-}F}$ = 3.4 Hz), 131.0, 128.0 (d, $^3J_{C\text{-}F}$ = 7.9 Hz), 115.1 (d, $^2J_{C\text{-}F}$ = 21.4 Hz), 60.7, 29.2, 27.5, 14.5, 13.8, 12.1. ^{19}F-NMR (376 MHz, $CDCl_3$): δ = -115.94 (d, J = 6.0 Hz). HR-MS (EI) m/z calcd for $C_{23}H_{37}FO_2Sn$ [M+H$^+$] 485.1872, found 485.1875.

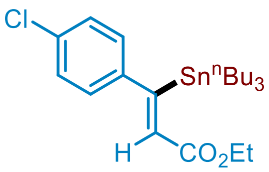

Ethyl (*Z*)-3-(4-chlorophenyl)-3-(tributylstannyl)acrylate (44)

The general procedure **TP6** was followed using **3ae** (0.25 mmol) and **2a** (0.325 mmol) at -20 ℃ for 15 min. Purification by column chromatography (PE) yielded **44** (85 mg, 68%) as a colorless oil. ^{1}H-NMR (400 MHz, $CDCl_3$): δ = 7.31 – 7.26 (m, 2H), 6.99 – 6.94 (m, 2H), 6.45 (s, 1H), 4.24 (q, J = 7.1 Hz, 2H), 1.45 – 1.34 (m, 6H), 1.33 – 1.19 (m, 9H), 0.97 – 0.92 (m, 6H), 0.84 (t, J = 7.3 Hz, 9H). ^{13}C-NMR (100 MHz, $CDCl_3$): δ = 172.5, 167.7, 144.4, 132.8, 131.2, 128.4, 127.6, 60.8, 29.2, 27.5, 14.5, 13.8, 12.1. HR-MS (EI) m/z calcd for $C_{23}H_{37}ClO_2Sn$ [M+H$^+$] 501.1577, found 501.1581.

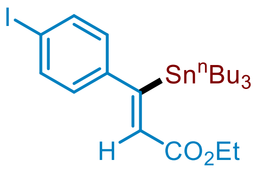

Ethyl (*Z*)-3-(4-iodophenyl)-3-(tributylstannyl)acrylate (45)

The general procedure **TP6** was followed using **3af** (0.25 mmol) and **2a** (0.275 mmol) at -20 °C for 10 min. Purification by column chromatography (PE) yielded **45** (71 mg, 49%) as a colorless oil. ^{1}H-NMR (400 MHz, $CDCl_3$): δ = 7.76 – 7.50 (m, 2H), 6.90 – 6.67 (m, 2H), 6.56 – 6.29 (m, 1H), 4.23 (q, J = 7.1 Hz, 2H), 1.44 – 1.33 (m, 6H), 1.33 – 1.18 (m, 9H), 0.96 – 0.91 (m, 6H), 0.84 (t, J = 7.3 Hz, 9H). ^{13}C-NMR (100 MHz, $CDCl_3$): δ = 172.5, 167.7, 145.6, 137.3, 131.1, 128.2, 92.2, 60.8, 29.2, 27.5, 14.5, 13.8, 12.1. HR-MS (EI) m/z calcd for $C_{23}H_{37}IO_2Sn$ [M+H$^+$] 593.0933, found 593.0938.

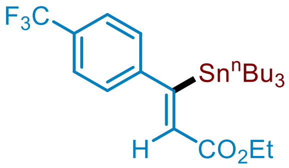

Ethyl (*Z*)-3-(tributylstannyl)-3-[4-(trifluoromethyl)phenyl]acrylate (46)

The general procedure **TP7** was followed using **3ag** (0.25 mmol) and **2a** (0.325 mmol) at -20 ℃ for 2.5 h. Purification by column chromatography (PE) yielded **46** (71 mg, 53%) as a colorless oil. ^{1}H-NMR (400 MHz, $CDCl_3$): δ = 7.57 (d, J = 8.1 Hz, 2H), 7.12 (d, J = 8.1 Hz, 2H), 6.46 (s, 1H), 4.25 (q, J = 7.1 Hz, 2H), 1.45 – 1.34 (m, 6H), 1.25 (m, 9H), 0.97 – 0.91 (m,

6H), 0.83 (t, J = 7.3 Hz, 9H). ^{13}C-NMR (100 MHz, CDCl$_3$): δ = 172.4, 167.6, 149.9, 131.7, 128.8 (q, $^2J_{C\text{-}F}$ = 32.4 Hz), 126.4, 125.2 (q, $^3J_{C\text{-}F}$ = 3.7 Hz), 124.4 (q, $^1J_{C\text{-}F}$ = 271.8 Hz), 60.9, 29.2, 27.5, 14.5, 13.8, 12.1. ^{19}F-NMR (376 MHz, CDCl$_3$): δ = -62.39 (s). HR-MS (EI) m/z calcd for $C_{24}H_{37}F_3O_2Sn$ [M+H$^+$] 535.1840, found 535.1844.

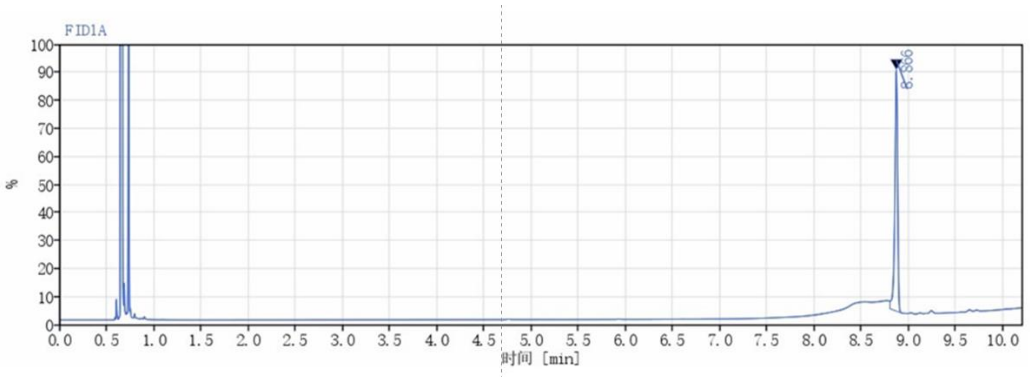

Supplementary Figure 7. GC data of compound **46**.

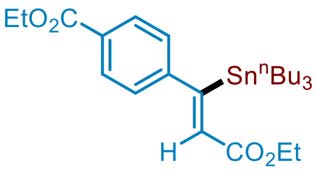

Ethyl (*Z*)-4-[3-ethoxy-3-oxo-1-(tributylstannyl)prop-1-en-1-yl]benzoate (47)

The general procedure **TP6** was followed using **3ah** (0.25 mmol) and **2a** (0.325 mmol) at -20 °C for 15 min. Purification by column chromatography (petroleum ether/EtOAc 50:1) yielded **47** (88 mg, 66%) as a colorless oil. ^{1}H-NMR (400 MHz, CDCl$_3$): δ = 8.07 – 7.96 (m, 2H), 7.10 – 7.03 (m, 2H), 6.46 (s, 1H), 4.38 (q, J = 7.1 Hz, 2H), 4.25 (q, J = 7.1 Hz, 2H), 1.43 – 1.36 (m, 6H), 1.36 – 1.16 (m, 12H), 0.97 – 0.92 (m, 6H), 0.83 (t, J = 7.3 Hz, 9H). ^{13}C-NMR (100 MHz, CDCl$_3$): δ = 172.9, 167.6, 166.6, 151.0, 131.3, 129.6, 128.7, 126.1, 61.1, 60.8, 29.2, 27.4, 14.5, 14.5, 13.8, 12.1. HR-MS (EI) m/z calcd for $C_{26}H_{42}O_4Sn$ [M+H$^+$] 539.2178, found 539.2181.

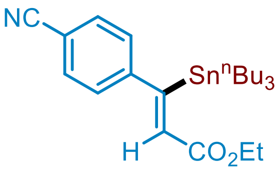

Ethyl (*Z*)-3-(4-cyanophenyl)-3-(tributylstannyl)acrylate (48)

The general procedure **TP7** was followed using **3ai** (0.25 mmol) and **2a** (0.325 mmol) at -20 °C for 15 min. Purification by column chromatography (petroleum ether/EtOAc 50:1) yielded **48** (66 mg, 54%) as a colorless oil. ^{1}H-NMR (400 MHz, CDCl$_3$): δ = 7.61 (d, J = 8.4 Hz, 2H), 7.15

– 7.06 (m, 2H), 6.44 (s, 1H), 4.25 (q, J = 7.1 Hz, 2H), 1.40 – 1.33 (m, 6H), 1.33 – 1.18 (m, 10H), 0.95 – 0.91 (m, 5H), 0.84 (t, J = 7.3 Hz, 9H). ^{13}C-NMR (100 MHz, $CDCl_3$): δ = 172.0, 167.4, 151.2, 132.1, 131.9, 126.8, 119.1, 110.2, 61.0, 29.1, 27.4, 14.4, 13.8, 12.1. HR-MS (EI) m/z calcd for $C_{24}H_{37}NO_2Sn$ [M+H$^+$] 492.1919, found 492.1921.

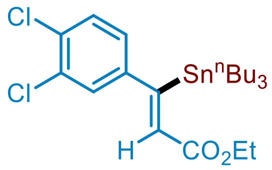

Ethyl (*Z*)-3-(3,4-dichlorophenyl)-3-(tributylstannyl)acrylate (49)

The general procedure **TP6** was followed using **3aj** (0.25 mmol) and **2a** (0.325 mmol) at -20 ℃ for 15 min. Purification by column chromatography (PE) yielded **49** (104 mg, 78%) as a colorless oil. ^{1}H-NMR (400 MHz, $CDCl_3$): δ = 7.38 (d, J = 8.2 Hz, 1H), 7.13 (d, J = 2.1 Hz, 1H), 6.91 – 6.83 (m, 1H), 6.45 (s, 1H), 4.24 (q, J = 7.1 Hz, 2H), 1.46 – 1.34 (m, 6H), 1.34 – 1.20 (m, 9H), 0.97 – 0.92 (m, 6H), 0.85 (t, J = 7.3 Hz, 9H). ^{13}C-NMR (100 MHz, $CDCl_3$): δ = 171.1, 167.5, 146.1, 132.3, 131.8, 130.7, 130.1, 128.0, 125.8, 60.9, 29.2, 27.5, 14.4, 13.8, 12.2. HR-MS (EI) m/z calcd for $C_{23}H_{36}Cl_2O_2Sn$ [M+H$^+$] 535.1187, found 535.1191.

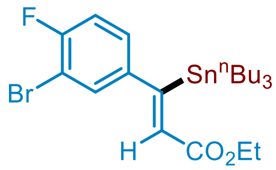

Ethyl (*Z*)-3-(3-bromo-4-fluorophenyl)-3-(tributylstannyl)acrylate (50)

The general procedure **TP6** was followed using **3ak** (0.25 mmol) and **2a** (0.325 mmol) at -20 ℃ for 15 min. Purification by column chromatography (PE) yielded **50** (93 mg, 66%) as a colorless oil. ^{1}H-NMR (400 MHz, $CDCl_3$): δ = 7.10 – 6.98 (m, 3H), 6.45 (s, 1H), 4.24 (q, J = 7.1 Hz, 2H), 1.51 – 1.35 (m, 6H), 1.35 – 1.18 (m, 9H), 0.94 (m, 6H), 0.84 (td, J = 7.3, 4.1 Hz, 9H). ^{13}C-NMR (100 MHz, $CDCl_3$): δ = 172.6, 167.8, 162.1 (d, $^1J_{C\text{-}F}$ = 245.8 Hz), 141.9 (d, $^4J_{C\text{-}F}$ = 3.0 Hz), 131.8, 131.1 (d, $^3J_{C\text{-}F}$ = 6.2 Hz), 128.0 (d, $^3J_{C\text{-}F}$ = 7.9 Hz), 116.2 (d, $^2J_{C\text{-}F}$ = 22.7 Hz), 115.1 (d, $^2J_{C\text{-}F}$ = 21.4 Hz), 60.7, 29.2, 27.5, 14.5, 13.8, 12.1. ^{19}F-NMR (376 MHz, $CDCl_3$): δ = -116.04 (s). HR-MS (EI) m/z calcd for $C_{23}H_{36}BrFO_2Sn$ [M+H$^+$] 563.0977, found 563.0979.

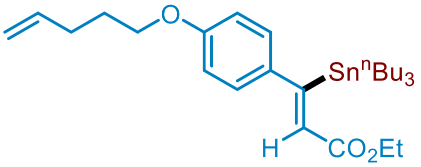

Ethyl (*Z*)-3-[4-(pent-4-en-1-yloxy)phenyl]-3-(tributylstannyl)acrylate (51)

The general procedure **TP6** was followed using **3al** (0.25 mmol) and **2a** (0.325 mmol) at -20 °C for 15 min. Purification by column chromatography (PE) yielded **51** (98 mg, 71%) as a colorless oil. ^{1}H-NMR (400 MHz, $CDCl_3$): δ = 7.05 – 6.98 (m, 2H), 6.88 – 6.82 (m, 2H), 6.46 (s, 1H), 5.85 (ddt, *J* = 16.9, 10.2, 6.6 Hz, 1H), 5.11 – 4.95 (m, 2H), 4.22 (q, *J* = 7.1 Hz, 2H), 3.97 (t, *J* = 6.5 Hz, 2H), 2.30 – 2.19 (m, 2H), 1.97 – 1.82 (m, 2H), 1.51 – 1.36 (m, 6H), 1.26 (m, 9H), 0.98 – 0.92 (m, 6H), 0.84 (t, *J* = 7.3 Hz, 9H). ^{13}C-NMR (100 MHz, $CDCl_3$): δ = 173.2, 168.1, 158.6, 138.1, 137.9, 129.8, 127.9, 115.3, 114.3, 67.4, 60.5, 30.3, 29.2, 28.6, 27.5, 14.5, 13.8, 12.2. HR-MS (EI) m/z calcd for $C_{28}H_{46}O_3Sn$ [M+H$^+$] 551.2542, found 551.2547.

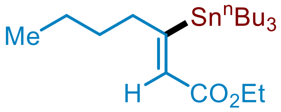

Ethyl (*Z*)-3-(tributylstannyl)hept-2-enoate (52)

The general procedure **TP6** was followed using **3am** (0.25 mmol) and **2a** (0.325 mmol) at -20 °C for 15 min. Purification by column chromatography (PE) yielded **52** (85 mg, 76%) as a colorless oil. ^{1}H-NMR (400 MHz, $CDCl_3$): δ = 6.36 (t, *J* = 1.2 Hz, 1H), 4.17 (q, *J* = 7.1 Hz, 2H), 2.39 (t, *J* = 6.7 Hz, 2H), 1.51 – 1.40 (m, 6H), 1.38 – 1.24 (m, 13H), 0.97 – 0.93 (m, 6H), 0.89 (dd, *J* = 13.6, 6.4 Hz, 12H). ^{13}C-NMR (100 MHz, $CDCl_3$): δ = 176.1, 168.1, 128.4, 60.3, 40.2, 31.5, 29.4, 27.6, 22.5, 14.5, 14.1, 13.9, 11.2. HR-MS (EI) m/z calcd for $C_{21}H_{42}O_2Sn$ [M+H$^+$] 447.2280, found 447.2284.

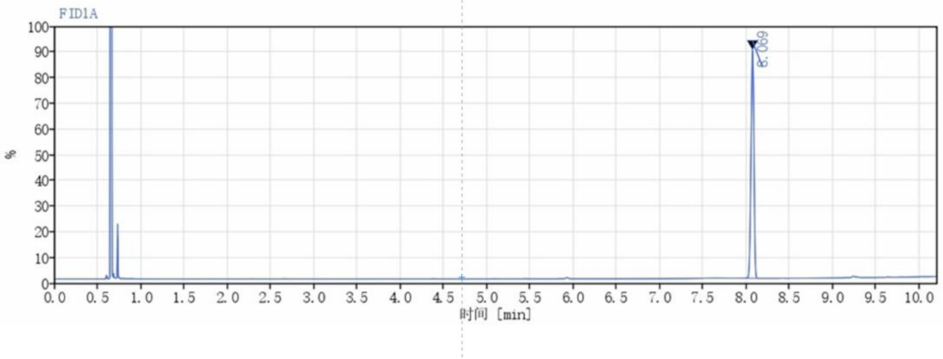

Supplementary Figure 8. GC data of compound **52**.

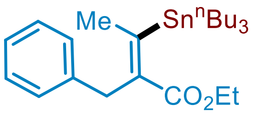

Ethyl (*Z*)-2-benzyl-3-(tributylstannyl)but-2-enoate (53)

The general procedure **TP7** was followed using **3c** (0.25 mmol) and **2a** (0.325 mmol) at 0 °C for 1 h. Purification by column chromatography (petroleum ether/EtOAc 100:1) yielded **53** (85 mg, 69%) as a colorless oil. ^{1}H-NMR (400 MHz, $CDCl_3$): δ = 7.24 (dd, J = 8.9, 5.8 Hz, 2H), 7.14 (dd, J = 13.6, 7.1 Hz, 3H), 4.11 (q, J = 7.1 Hz, 2H), 3.84 (s, 2H), 2.19 – 2.03 (m, 3H), 1.55 – 1.41 (m, 6H), 1.30 (dt, J = 14.7, 7.3 Hz, 6H), 1.16 (t, J = 7.1 Hz, 3H), 1.02 – 0.90 (m, 6H), 0.88 (t, J = 7.3 Hz, 9H). ^{13}C-NMR (100 MHz, $CDCl_3$): δ = 169.1, 165.1, 140.5, 137.9, 128.3, 128.2, 125.8, 60.9, 33.6, 29.5, 27.7, 22.9, 14.3, 14.0, 12.0. HR-MS (EI) m/z calcd for $C_{25}H_{42}O_2Sn$ [M+H$^+$] 495.2280, found 495.2283.

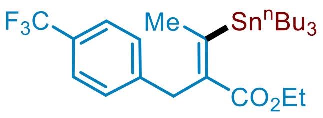

Ethyl (*Z*)-3-(tributylstannyl)-2-[4-(trifluoromethyl)benzyl]but-2-enoate (54)

The general procedure **TP7** was followed using **3e** (0.15 mmol) and **2a** (0.195 mmol) at 0 ℃ for 1 h. Purification by column chromatography (PE) yielded **54** (62 mg, 73%) as a colorless oil. ^{1}H-NMR (400 MHz, $CDCl_3$): δ = 7.49 (d, J = 8.1 Hz, 2H), 7.23 (d, J = 8.0 Hz, 2H), 4.11 (q, J = 7.1 Hz, 2H), 3.88 (s, 2H), 2.17 – 2.06 (m, 3H), 1.52 – 1.42 (m, 6H), 1.33 – 1.27 (m, 6H), 1.16 (t, J = 7.1 Hz, 3H), 0.99 – 0.90 (m, 6H), 0.88 (t, J = 7.3 Hz, 9H). ^{13}C-NMR (100 MHz, $CDCl_3$): δ = 168.7, 166.4, 144.8, 137.0 128.5, 128.2 (q, $^2J_{C\text{-}F}$ = 32.4 Hz), 125.3 (q, $^3J_{C\text{-}F}$ = 3.8 Hz), 124.5 (q, $^1J_{C\text{-}F}$ = 272.0 Hz), 61.0, 33.6, 29.5, 27.7, 23.0, 14.3, 13.9, 12.0. ^{19}F-NMR (376 MHz, $CDCl_3$): δ = -62.30 (s). HR-MS (EI) m/z calcd for $C_{26}H_{41}F_3O_2Sn$ [M+H$^+$] 563.2153, found 563.2155.

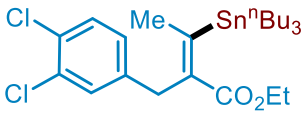

Ethyl (*Z*)-2-(3,4-dichlorobenzyl)-3-(tributylstannyl)but-2-enoate (55)

The general procedure **TP7** was followed using **3j** (0.25 mmol) and **2a** (0.325 mmol) at 0 °C for 1 h. Purification by column chromatography (PE) yielded **55** (118 mg, 84%) as a colorless oil. ^{1}H-NMR (400 MHz, $CDCl_3$): δ = 7.29 (d, J = 8.2 Hz, 1H), 7.21 (d, J = 1.8 Hz, 1H), 6.95 (dd, J = 8.3, 1.9 Hz, 1H), 4.12 (q, J = 7.1 Hz, 2H), 3.77 (s, 2H), 2.16 – 2.05 (m, 3H), 1.53 –

1.40 (m, 6H), 1.34 – 1.26 (m, 6H), 1.18 (t, *J* = 7.1 Hz, 3H), 1.01 – 0.91 (m, 6H), 0.88 (t, *J* = 7.3 Hz, 9H). ^{13}C-NMR (100 MHz, $CDCl_3$): δ = 168.6, 166.6, 140.9, 136.8, 132.3, 130.2, 130.2, 129.7, 127.6, 61.0, 32.8, 29.4, 27.6, 23.0, 14.3, 13.9, 12.0. HR-MS (EI) m/z calcd for $C_{25}H_{40}Cl_2O_2Sn$ [M+H$^+$] 563.1500, found 563.1503.

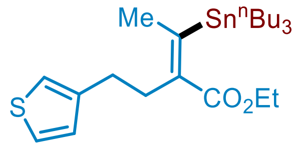

Ethyl (*Z*)-2-[2-(thiophen-3-yl)ethyl]-3-(tributylstannyl)but-2-enoate (56)

The general procedure **TP7** was followed using **3m** (0.25 mmol) and **2a** (0.325 mmol) at 0 °C for 1 h. Purification by column chromatography (PE) yielded **56** (67 mg, 52%) as a colorless oil. ^{1}H-NMR (400 MHz, $CDCl_3$): δ = 7.22 (dd, *J* = 4.9, 2.9 Hz, 1H), 6.94 (dd, *J* = 4.9, 1.2 Hz, 1H), 6.92 – 6.88 (m, 1H), 4.17 (q, *J* = 7.1 Hz, 2H), 2.76 – 2.66 (m, 4H), 1.91 (d, *J* = 22.5 Hz, 3H), 1.49 – 1.40 (m, 6H), 1.28 (td, *J* = 7.1, 3.4 Hz, 9H), 0.92 – 0.86 (m, 15H). ^{13}C-NMR (100 MHz, $CDCl_3$): δ = 169.2, 164.1, 142.5, 138.8, 128.6, 125.2, 120.4, 60.8, 30.0, 29.4, 29.3, 27.7, 22.1, 14.5, 13.9, 11.8. HR-MS (EI) m/z calcd for $C_{24}H_{42}O_2SSn$ [M+H$^+$] 515.2000, found 515.2005.

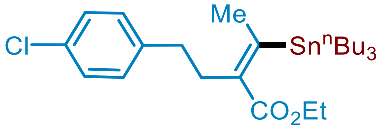

Ethyl (*Z*)-2-(4-chlorophenethyl)-3-(tributylstannyl)but-2-enoate (57)

The general procedure **TP7** was followed using corresponding alkenyl acetate (0.25 mmol) and **2a** (0.325 mmol) at 0 °C for 1 h. Purification by column chromatography (petroleum ether/EtOAc 100:1) yielded **57** (77 mg, 57%) as a colorless oil. ^{1}H-NMR (400 MHz, $CDCl_3$): δ = 7.22 (d, *J* = 8.3 Hz, 2H), 7.09 (d, *J* = 8.3 Hz, 2H), 4.16 (q, *J* = 7.1 Hz, 2H), 2.67 (m, 4H), 2.04 – 1.81 (m, 3H), 1.50 – 1.39 (m, 6H), 1.32 – 1.25 (m, 9H), 0.95 – 0.84 (m, 15H). ^{13}C-NMR (100 MHz, $CDCl_3$): δ = 169.1, 164.4, 140.6, 138.3, 131.7, 130.1, 128.4, 60.8, 35.1, 30.1, 29.4, 27.7, 22.2, 14.5, 13.9, 11.8. HR-MS (EI) m/z calcd for $C_{26}H_{43}ClO_2Sn$ [M+H$^+$] 543.2046, found 543.2049.

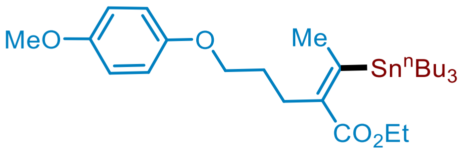

Ethyl (*Z*)-5-(4-methoxyphenoxy)-2-[1-(tributylstannyl)ethylidene]pentanoate(58)

The general procedure **TP7** was followed using **3q** (0.25 mmol) and **2a** (0.325 mmol) at 0 °C for 50 min. Purification by column chromatography (petroleum ether/EtOAc 50:1) yielded **58** (74 mg, 52%) as a colorless oil. ^{1}H-NMR (400 MHz, $CDCl_3$): δ = 6.81 (s, 4H), 4.17 (q, J = 7.1 Hz, 2H), 3.88 (t, J = 6.3 Hz, 2H), 3.76 (s, 3H), 2.62 (t, J = 7.4 Hz, 2H), 2.11 – 1.99 (m, 3H), 1.90 – 1.82 (m, 2H), 1.49 – 1.40 (m, 6H), 1.28 (m, 9H), 0.88 (m, 15H). ^{13}C-NMR (100 MHz, $CDCl_3$): δ = 169.3, 163.9, 153.8, 153.3, 138.8, 115.5, 114.8, 67.9, 60.8, 55.9, 29.4, 29.1, 27.6, 24.4, 22.3, 14.5, 13.9, 11.8. HR-MS (EI) m/z calcd for $C_{28}H_{48}O_4Sn$ [M+H$^+$] 569.2647, found 569.2650.

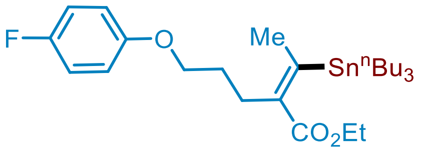

Ethyl (*Z*)-5-(4-fluorophenoxy)-2-[1-(tributylstannyl)ethylidene]pentanoate (59)

The general procedure **TP7** was followed using **3r** (0.25 mmol) and **2a** (0.325 mmol) at 0 ℃ for 1 h. Purification by column chromatography (petroleum ether/EtOAc 50:1) yielded **59** (112 mg, 81%) as a colorless oil. ^{1}H-NMR (400 MHz, $CDCl_3$): δ = 6.98 – 6.91 (m, 2H), 6.87 – 6.75 (m, 2H), 4.17 (q, J = 7.1 Hz, 2H), 3.88 (t, J = 6.3 Hz, 2H), 2.63 (t, J = 7.4 Hz, 2H), 2.11 – 1.98 (m, 3H), 1.91 – 1.82 (m, 2H), 1.50 – 1.38 (m, 6H), 1.27 (dd, J = 15.0, 7.7 Hz, 9H), 0.93 – 0.84 (m, 15H). ^{13}C-NMR (100 MHz, $CDCl_3$): δ = 169.2, 164.1, 157.3 (d, $^{1}J_{C\text{-}F}$ = 237.7 Hz), 155.3 (d, $^{4}J_{C\text{-}F}$ = 2.0 Hz), 138.7, 115.9 (d, $^{2}J_{C\text{-}F}$ = 23.0 Hz), 115.4 (d, $^{3}J_{C\text{-}F}$ = 8.0 Hz), 67.9, 60.8, 29.4, 29.0, 27.6, 24.3, 22.3, 14.5, 13.9, 11.8. ^{19}F-NMR (376 MHz, $CDCl_3$): δ = -124.43 (s). HR-MS (EI) m/z calcd for $C_{27}H_{45}FO_3Sn$ [M+H$^+$] 557.2447, found 557.2450.

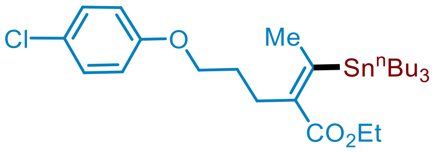

Ethyl (*Z*)-5-(4-chlorophenoxy)-2-[1-(tributylstannyl)ethylidene]pentanoate (60)

The general procedure **TP7** was followed using **3s** (0.25 mmol) and **2a** (0.325 mmol) at 0 °C for 1 h. Purification by column chromatography (petroleum ether/EtOAc 50:1) yielded **60** (89 mg, 62%) as a colorless oil. ^{1}H-NMR (400 MHz, $CDCl_3$): δ = 7.24 – 7.15 (m, 2H), 6.87 – 6.74 (m, 2H), 4.16 (q, J = 7.1 Hz, 2H), 3.89 (t, J = 6.3 Hz, 2H), 2.62 (t, J = 7.4 Hz, 2H), 2.13 – 1.97 (m, 3H), 1.94 – 1.81 (m, 2H), 1.49 – 1.39 (m, 6H), 1.32 – 1.21 (m, 9H), 0.88 (m, 15H). ^{13}C-

NMR (100 MHz, $CDCl_3$): δ = 169.2, 164.2, 157.7, 138.6, 129.4, 125.5, 115.8, 67.6, 60.8, 29.4, 28.9, 27.6, 24.3, 22.4, 14.5, 13.9, 11.8. HR-MS (EI) m/z calcd for $C_{27}H_{45}ClO_3Sn$ [M+H$^+$] 573.2152, found 573.2155.

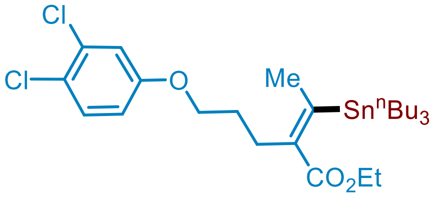

Ethyl (*Z*)-5-(3,4-dichlorophenoxy)-2-[1-(tributylstannyl)ethylidene]pentanoate (61)

The general procedure **TP7** was followed using **3u** (0.25 mmol) and **2a** (0.325 mmol) at 0 °C for 1 h. Purification by column chromatography (petroleum ether/EtOAc 50:1) yielded **61** (87 mg, 58%) as a colorless oil. ^{1}H-NMR (400 MHz, $CDCl_3$): δ = 7.30 (d, *J* = 8.9 Hz, 1H), 6.96 (d, *J* = 2.8 Hz, 1H), 6.73 (dd, *J* = 8.9, 2.8 Hz, 1H), 4.17 (q, *J* = 7.1 Hz, 2H), 3.89 (t, *J* = 6.2 Hz, 2H), 2.61 (t, *J* = 7.4 Hz, 2H), 2.10 – 1.98 (m, 3H), 1.92 – 1.83 (m, 2H), 1.49 – 1.37 (m, 6H), 1.28 (dd, *J* = 13.6, 6.7 Hz, 9H), 0.88 (m, 15H). ^{13}C-NMR (100 MHz, $CDCl_3$): δ = 169.1, 164.3, 158.2, 138.4, 133.9, 130.8, 123.9, 116.4, 114.6, 67.9, 60.9, 29.4, 28.8, 27.6, 24.3, 22.4, 14.5, 13.9, 11.8. HR-MS (EI) m/z calcd for $C_{27}H_{44}Cl_2O_3Sn$ [M+H$^+$] 607.1762, found 607.1764.

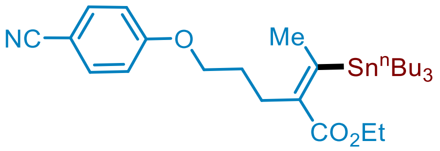

Ethyl (*Z*)-5-(4-cyanophenoxy)-2-[1-(tributylstannyl)ethylidene]pentanoate (62)

The general procedure **TP7** was followed using **3an** (0.25 mmol) and **2a** (0.325 mmol) at 0 °C for 1 h. Purification by column chromatography (petroleum ether/EtOAc 20:1) yielded **62** (66 mg, 47%) as a colorless oil. ^{1}H-NMR (400 MHz, $CDCl_3$): δ = 7.57 (d, *J* = 8.9 Hz, 2H), 6.92 (d, *J* = 8.9 Hz, 2H), 4.17 (q, *J* = 7.1 Hz, 2H), 3.98 (t, *J* = 6.2 Hz, 2H), 2.64 (t, *J* = 7.4 Hz, 2H), 2.11 – 1.98 (m, 3H), 1.96 – 1.87 (m, 2H), 1.53 – 1.37 (m, 6H), 1.27 (dd, *J* = 14.8, 7.6 Hz, 9H), 0.95 – 0.82 (m, 15H). ^{13}C-NMR (100 MHz, $CDCl_3$): δ = 169.1, 164.4, 162.4, 138.3, 134.1, 119.4, 115.2, 103.9, 67.7, 60.8, 29.4, 28.6, 27.6, 24.2, 22.3, 14.4, 13.9, 11.8. HR-MS (EI) m/z calcd for $C_{28}H_{45}NO_3Sn$ [M+H$^+$] 564.2494, found 564.2496.

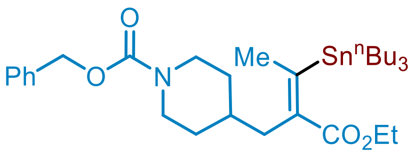

Benzyl (*Z*)-4-[2-(ethoxycarbonyl)-3-(tributylstannyl)but-2-en-1-yl]piperidine-1-carboxylate (63)

The general procedure **TP7** was followed using **3p** (0.25 mmol) and **2a** (0.325 mmol) at 0 °C for 1 h. Purification by column chromatography (petroleum ether/EtOAc 10:1) yielded **63** (89 mg, 56%) as a colorless oil. ^{1}H-NMR (400 MHz, $CDCl_3$): δ = 7.40 – 7.26 (m, 5H), 5.12 (s, 2H), 4.18 (q, J = 7.1 Hz, 4H), 2.70 (s, 2H), 2.42 (d, J = 6.8 Hz, 2H), 2.11 – 1.94 (m, 3H), 1.59 – 1.34 (m, 11H), 1.34 – 1.09 (m, 9H), 0.92 – 0.84 (m, 15H). ^{13}C-NMR (100 MHz, $CDCl_3$): δ = 169.4, 164.5, 155.4, 137.6, 137.1, 128.6, 128.0, 128.0, 67.1, 60.8, 44.5, 36.8, 34.2, 32.1, 29.4, 27.6, 23.0, 14.5, 13.9, 11.9. HR-MS (EI) m/z calcd for $C_{32}H_{53}NO_4Sn$ [M+H$^+$] 636.3069, found 636.3073.

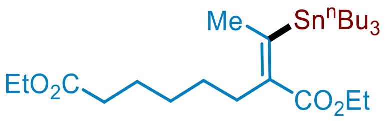

Diethyl (*Z*)-2-[1-(tributylstannyl)ethylidene]octanedioate (64)

The general procedure **TP7** was followed using **corresponding alkenyl acetate** (0.25 mmol) and **2a** (0.325 mmol) at 0 °C for 1 h. Purification by column chromatography (petroleum ether/EtOAc 50:1) yielded **64** (74 mg, 54%) as a colorless oil. ^{1}H-NMR (400 MHz, $CDCl_3$): δ = 4.18 (dd, J = 14.4, 7.3 Hz, 2H), 4.12 (dd, J = 14.4, 7.2 Hz, 2H), 2.53 – 2.34 (m, 2H), 2.29 (t, J = 7.6 Hz, 2H), 2.11 – 1.91 (m, 3H), 1.52 – 1.36 (m, 10H), 1.36 – 1.21 (m, 14H), 0.91 – 0.84 (m, 15H). ^{13}C-NMR (100 MHz, $CDCl_3$): δ = 174.0, 169.4, 162.6, 139.9, 60.7, 60.3, 34.5, 29.4, 29.2, 29.1, 27.8, 27.6, 25.0, 22.3, 14.5, 14.4, 13.9, 11.8. HR-MS (EI) m/z calcd for $C_{26}H_{50}O_4Sn$ [M+H$^+$] 547.2804, found 547.2810.

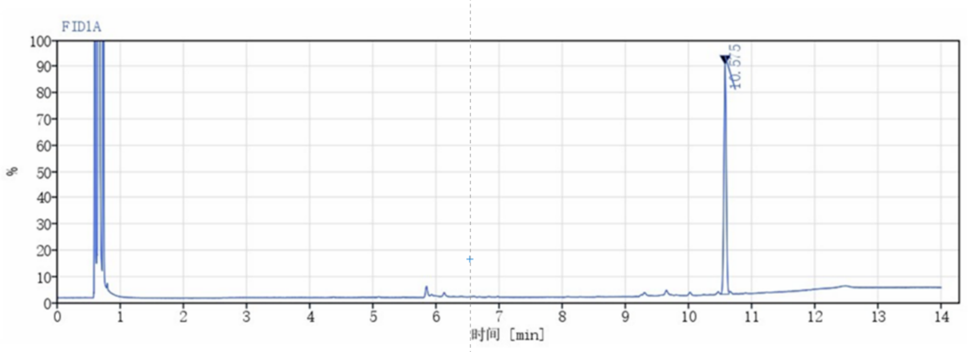

Supplementary Figure 9. GC data of compound **64**.

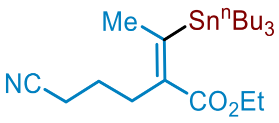

Ethyl (*Z*)-5-cyano-2-[1-(tributylstannyl)ethylidene]pentanoate (65)

The general procedure **TP7** was followed using (*Z*)-**87** (0.25 mmol) and **2a** (0.325 mmol) at 0 °C for 1 h. Purification by column chromatography (petroleum ether/EtOAc 50:1) yielded **65** (82 mg, 70%) as a colorless oil. ^{1}H-NMR (400 MHz, $CDCl_3$): δ = 4.21 (q, J = 7.1 Hz, 2H), 2.62 – 2.57 (m, 2H), 2.32 (t, J = 7.1 Hz, 2H), 2.14 – 2.03 (m, 3H), 1.80 – 1.73 (m, 2H), 1.53 – 1.36 (m, 6H), 1.33 – 1.21 (m, 9H), 0.99 – 0.81 (m, 15H). ^{13}C-NMR (100 MHz, $CDCl_3$): δ = 168.7, 165.8, 137.3, 119.8, 61.0, 29.3, 27.6, 26.8, 25.3, 22.5, 17.0, 14.4, 13.9, 11.8. HR-MS (EI) m/z calcd for $C_{22}H_{41}NO_2Sn$ [M+H$^+$] 472.2232, found 472.2236.

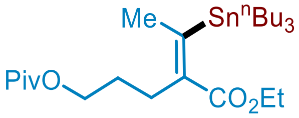

Ethyl (*Z*)-5-(pivaloyloxy)-2-[1-(tributylstannyl)ethylidene]pentanoate (66)

The general procedure **TP7** was followed using **3o** (0.25 mmol) and **2a** (0.325 mmol) at 0 °C for 1 h. Purification by column chromatography (petroleum ether/EtOAc 50:1) yielded **66** (84 mg, 62%) as a colorless oil. ^{1}H-NMR (400 MHz, $CDCl_3$): δ = 4.19 (q, J = 7.1 Hz, 2H), 4.06 (t, J = 6.3 Hz, 2H), 2.58 – 2.45 (m, 2H), 2.12 – 1.99 (m, 3H), 1.78 – 1.66 (m, 2H), 1.51 – 1.39 (m, 6H), 1.32 – 1.25 (m, 9H), 1.24 – 1.19 (m, 9H), 0.93 – 0.83 (m, 15H). ^{13}C-NMR (100 MHz, $CDCl_3$): δ = 178.7, 169.1, 164.1, 138.7, 64.2, 60.8, 38.9, 29.4, 28.7, 27.6, 27.4, 24.5, 22.2, 14.5, 13.9, 11.8. HR-MS (EI) m/z calcd for $C_{26}H_{50}O_4Sn$ [M+H$^+$] 547.2804, found 547.2808.

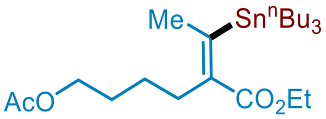

Ethyl (*Z*)-6-acetoxy-2-(1-(tributylstannyl)ethylidene)hexanoate (67)

The general procedure **TP7** was followed using corresponding alkenyl acetate (0.25 mmol) and **2a** (0.325 mmol) at 0 °C for 1 h. Purification by column chromatography (petroleum ether/EtOAc 50:1) yielded **67** (82 mg, 63%) as a colorless oil. ^{1}H-NMR (400 MHz, $CDCl_3$): δ = 4.19 (q, J = 7.1 Hz, 2H), 4.07 (t, J = 6.7 Hz, 2H), 2.51 – 2.41 (m, 2H), 2.11 – 1.99 (m, 6H), 1.68 – 1.60 (m, 2H), 1.50 – 1.39 (m, 8H), 1.28 (dd, J = 14.3, 7.1 Hz, 9H), 0.88 (m, 15H). ^{13}C-NMR (100 MHz, $CDCl_3$): δ = 171.3, 169.3, 163.2, 139.4, 64.6, 60.8, 29.4, 28.6, 27.6, 27.4, 25.8, 22.3, 21.1, 14.5, 13.9, 11.8. HR-MS (EI) m/z calcd for $C_{24}H_{46}O_4Sn$ [M+H$^+$] 519.2491,

found 519.2493.

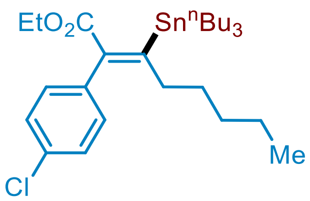

Ethyl (*Z*)-2-(4-chlorophenyl)-3-(tributylstannyl)oct-2-enoate (68)

The general procedure **TP7** was followed using **3v** (0.25 mmol) and **2a** (0.325 mmol) at 23 °C for 6 h. Purification by column chromatography (PE) yielded **68** (77 mg, 54%) as a colorless oil. ^{1}H-NMR (400 MHz, $CDCl_3$): δ = 7.36 – 7.26 (m, 2H), 7.05 – 6.92 (m, 2H), 4.11 (q, J = 7.1 Hz, 2H), 2.26 – 2.05 (m, 2H), 1.55 – 1.43 (m, 6H), 1.32 (dd, J = 14.7, 7.3 Hz, 6H), 1.21 – 1.07 (m, 9H), 0.99 (m, 6H), 0.90 (t, J = 7.3 Hz, 9H), 0.79 (t, J = 7.0 Hz, 3H). ^{13}C-NMR (100 MHz, $CDCl_3$): δ = 172.6, 168.4, 139.6, 137.1, 132.6, 131.1, 128.1, 61.1, 36.3, 31.9, 29.5, 29.3, 27.7, 22.5, 14.4, 14.0, 13.9, 12.1. HR-MS (EI) m/z calcd for $C_{28}H_{47}ClO_2Sn$ [M+H$^+$] 571.2359, found 571.2363.

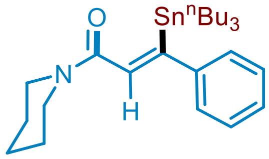

(*Z*)-3-Phenyl-1-(piperidin-1-yl)-3-(tributylstannyl)prop-2-en-1-one (69)

The general procedure **TP7** was followed using **3ao** (0.25 mmol) and **2a** (0.325 mmol) at 23 °C for 40 min. Purification by column chromatography (petroleum ether/EtOAc 50:1) yielded **69** (70 mg, 56%) as a yellow oil. ^{1}H-NMR (400 MHz, $CDCl_3$): δ = 7.30 (t, J = 7.4 Hz, 2H), 7.25 – 7.16 (m, 1H), 7.11 – 7.03 (m, 2H), 7.03 – 6.77 (m, 1H), 3.71 – 3.46 (m, 4H), 1.65 (dd, J = 7.0, 3.5 Hz, 2H), 1.60 – 1.55 (m, 4H), 1.45 – 1.32 (m, 6H), 1.22 (dt, J = 14.3, 7.2 Hz, 6H), 1.00 – 0.86 (m, 6H), 0.82 (t, J = 7.3 Hz, 9H). ^{13}C-NMR (100 MHz, $CDCl_3$): δ = 168.8, 166.3, 147.0, 130.4, 128.1, 126.5, 126.4, 46.8, 43.6, 29.4, 27.6, 26.9, 25.8, 24.8, 13.9, 12.6. HR-MS (EI) m/z calcd for $C_{26}H_{43}NOSn$ [M+H$^+$] 506.2439, found 506.2444.

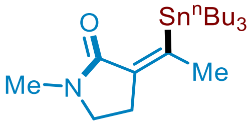

(*Z*)-1-Methyl-3-(1-(tributylstannyl)ethylidene)pyrrolidin-2-one (70)

The general procedure **TP7** was followed using corresponding alkenyl acetate (0.25 mmol) and **2a** (0.325 mmol) at 23 °C for 40 min. Purification by column chromatography (petroleum

ether/EtOAc 50:1) yielded **70** (45 mg, 43%) as a colorless oil. ^{1}H-NMR (400 MHz, $CDCl_3$): δ = 3.45 – 3.34 (m, 2H), 2.92 (s, 3H), 2.76 – 2.66 (m, 2H), 2.06 – 1.92 (m, 3H), 1.52 – 1.37 (m, 6H), 1.29 (dd, J = 14.7, 7.3 Hz, 6H), 1.03 – 0.90 (m, 6H), 0.87 (t, J = 7.3 Hz, 9H). ^{13}C-NMR (100 MHz, $CDCl_3$): δ = 169.2, 149.6, 137.6, 46.5, 30.4, 29.5, 27.6, 23.7, 22.1, 13.9, 11.5. HR-MS (EI) m/z calcd for $C_{19}H_{37}NOSn$ [M+H$^+$] 416.1970, found 416.1974.

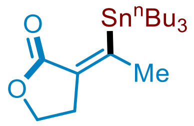

(*Z*)-3-[1-(tributylstannyl)ethylidene]dihydrofuran-2(3*H*)-one (71)

The general procedure **TP7** was followed using **3w** (0.25 mmol) and **2a** (0.325 mmol) at 0 °C for 40 min. Purification by column chromatography (petroleum ether/EtOAc 100:1) yielded **71** (68 mg, 68%) as a colorless oil. ^{1}H-NMR (400 MHz, $CDCl_3$): δ = 4.32 (t, J = 7.6 Hz, 2H), 2.85 (td, J = 7.6, 1.8 Hz, 2H), 2.07 – 1.96 (m, 3H), 1.44 – 1.30 (m, 6H), 1.22 (dd, J = 14.7, 7.3 Hz, 6H), 0.95 – 0.84 (m, 6H), 0.84 – 0.77 (m, 9H). ^{13}C-NMR (100 MHz, $CDCl_3$): δ = 172.2, 163.0, 131.7, 65.5, 29.3, 27.5, 26.8, 24.0, 13.8, 11.0. HR-MS (EI) m/z calcd for $C_{18}H_{34}O_2Sn$ [M+H$^+$] 403.1654, found 403.1656.

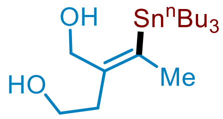

(*Z*)-2-[1-(Tributylstannyl)ethylidene]butane-1,4-diol (72)

The general procedure **TP10** was followed using **71** (0.5 mmol) and **$LiAlH_4$** (0.75 mmol) at 0 °C for 1 h. Purification by column chromatography (petroleum ether/EtOAc 3:1) yielded **72** (120 mg, 59%) as a colorless oil. ^{1}H-NMR (400 MHz, $CDCl_3$): δ = 3.99 (d, J = 4.6 Hz, 2H), 3.71 (q, J = 5.6 Hz, 2H), 2.77 (t, J = 5.0 Hz, 1H), 2.58 (t, J = 5.9 Hz, 2H), 2.53 (t, J = 4.8 Hz, 1H), 1.95 – 1.83 (m, 3H), 1.54 – 1.40 (m, 6H), 1.31 (dd, J = 14.6, 7.3 Hz, 6H), 0.91 (dt, J = 14.6, 7.8 Hz, 15H). ^{13}C-NMR (100 MHz, $CDCl_3$): δ = 144.9, 141.3, 70.4, 61.9, 33.9, 29.3, 27.5, 21.1, 13.8, 10.9. HR-MS (EI) m/z calcd for $C_{18}H_{38}O_2Sn$ [M+H$^+$] 407.1967, found 407.1969.

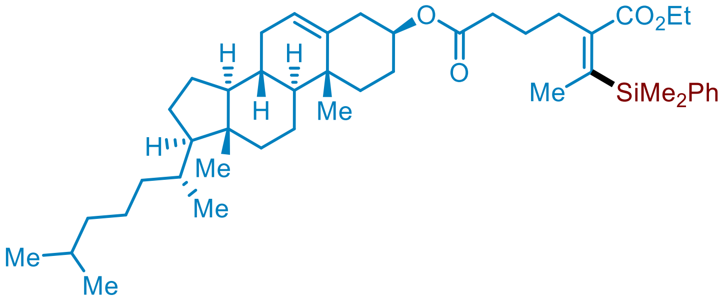

6-{(3*S*,8*S*,9*S*,10*R*,13*R*,14*S*,17*R*)-10,13-Dimethyl-17-[(*R*)-6-methylheptan-2-yl]-2,3,4,7,8,9,10,11,12,13,14,15,16,17-tetradecahydro-1*H*-cyclopenta[a]phenanthren-3-yl} 1-ethyl (*Z*)-2-{1-[dimethyl(phenyl)silyl]ethylidene}hexanedioate (73)

The general procedure **TP5** was followed using **3ap** (0.25 mmol) and **1a** (0.5 mmol) at 23 °C for 2.5 h. Purification by column chromatography (petroleum ether/EtOAc 50:1) yielded **73** (98 mg, 55%) as a colorless oil. ^{1}H-NMR (400 MHz, $CDCl_3$): δ = 7.50 – 7.44 (m, 2H), 7.33 – 7.27 (m, 3H), 5.37 (d, *J* = 4.0 Hz, 1H), 4.69 – 4.57 (m, 1H), 3.83 (q, *J* = 7.1 Hz, 2H), 2.51 – 2.41 (m, 2H), 2.31 (t, *J* = 7.2 Hz, 4H), 2.05 – 1.93 (m, 2H), 1.90 – 1.81 (m, 6H), 1.75 (dd, *J* = 15.4, 7.6 Hz, 2H), 1.59 – 1.44 (m, 6H), 1.42 – 1.18 (m, 6H), 1.17 – 1.11 (m, 4H), 1.09 (t, *J* = 7.1 Hz, 5H), 1.02 (s, 4H), 0.96 (dd, *J* = 12.3, 4.7 Hz, 2H), 0.92 (d, *J* = 6.5 Hz, 3H), 0.86 (dd, *J* = 6.6, 1.7 Hz, 6H), 0.68 (s, 3H), 0.38 (s, 6H). ^{13}C-NMR (100 MHz, $CDCl_3$): δ = 172.9, 169.6, 145.6, 143.3, 139.8, 139.6, 133.6, 128.6, 127.6, 122.8, 74.0, 60.5, 56.8, 56.3, 50.2, 42.4, 39.9, 39.7, 38.3, 37.1, 36.7, 36.3, 35.9, 34.4, 32.0, 32.0, 29.6, 28.4, 28.1, 27.9, 24.4, 24.1, 24.0, 23.0, 22.7, 21.2, 19.6, 19.5, 18.9, 14.1, 12.0, -1.0. HR-MS (EI) m/z calcd for $C_{45}H_{70}O_4Si$ [M+H$^+$] 703.5116, found 703.5119.

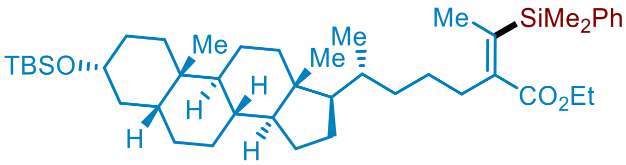

Ethyl (*R*,*Z*)-6-{(3*R*,5*R*,8*R*,9*S*,10*S*,13*R*,14*S*,17*R*)-3-[(tert-butyldimethylsilyl)oxy]-10,13-dimethylhexadecahydro-1*H*-cyclopenta[a]phenanthren-17-yl}-2-{1-[dimethyl(phenyl)silyl]ethylidene}heptanoate (74)

The general procedure **TP5** was followed using **3aq** (0.25 mmol) and **1a** (0.5 mmol) at 23 °C for 1 h. Purification by column chromatography (petroleum ether/EtOAc 100:1) yielded **74** (80 mg, 45%) as a colorless oil. ^{1}H-NMR (400 MHz, $CDCl_3$): δ = 7.51 – 7.46 (m, 2H), 7.33 – 7.28 (m, 3H), 3.83 (q, *J* = 7.1 Hz, 2H), 3.63 – 3.52 (m, 1H), 2.45 – 2.29 (m, 2H), 1.94 (d, *J* = 11.8 Hz, 1H), 1.85 – 1.72 (m, 7H), 1.53 (s, 2H), 1.38 (dt, *J* = 23.6, 15.1 Hz, 10H), 1.27 – 1.14 (m,

5H), 1.09 (t, J = 7.1 Hz, 5H), 1.04 (d, J = 8.7 Hz, 3H), 0.89 (s, 16H), 0.63 (s, 3H), 0.38 (s, 6H), 0.06 (s, 6H). ^{13}C-NMR (100 MHz, $CDCl_3$): δ = 170.1, 144.8, 143.4, 139.7, 133.7, 128.6, 127.6, 73.0, 60.4, 56.6, 56.4, 42.8, 42.5, 40.4, 40.3, 37.1, 36.0, 35.8, 35.8, 34.8, 31.2, 30.8, 28.4, 27.5, 26.6, 26.1, 25.2, 24.4, 23.6, 21.0, 19.4, 18.8, 18.5, 14.1, 12.2, -1.0, -4.4. HR-MS (EI) m/z calcd for $C_{44}H_{74}O_3Si_2$ [M+H$^+$] 707.5249, found 707.5253.

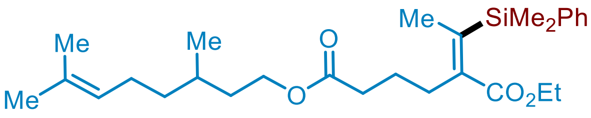

6-(3,7-Dimethyloct-6-en-1-yl) 1-ethyl (*Z*)-2-{1-[dimethyl(phenyl)silyl]ethylidene}hexanedioate (75)

The general procedure **TP5** was followed using corresponding alkenyl acetate (0.25 mmol) and **1a** (0.5 mmol) at 23 °C for 1.5 h. Purification by column chromatography (petroleum ether/EtOAc 50:1) yielded **75** (48 mg, 41%) as a colorless oil. ^{1}H-NMR (400 MHz, $CDCl_3$): δ = 7.49 – 7.45 (m, 2H), 7.32 – 7.29 (m, 3H), 5.08 (ddd, J = 7.1, 5.8, 1.3 Hz, 1H), 4.14 – 4.07 (m, 2H), 3.82 (q, J = 7.1 Hz, 2H), 2.48 – 2.43 (m, 2H), 2.33 (t, J = 7.4 Hz, 2H), 1.98 (dt, J = 17.1, 7.3 Hz, 2H), 1.85 (s, 3H), 1.76 (dd, J = 15.4, 7.7 Hz, 2H), 1.68 (s, 3H), 1.60 (s, 3H), 1.49 (ddd, J = 20.8, 13.3, 7.0 Hz, 2H), 1.40 – 1.22 (m, 2H), 1.22 – 1.14 (m, 1H), 1.08 (t, J = 7.1 Hz, 3H), 0.91 (d, J = 6.6 Hz, 3H), 0.38 (s, 6H). ^{13}C-NMR (100 MHz, $CDCl_3$): δ = 173.7, 169.6, 145.7, 143.3, 139.6, 133.6, 131.5, 128.6, 127.6, 124.7, 63.1, 60.5, 37.1, 35.6, 34.0, 29.6, 25.8, 25.5, 24.0, 19.6, 19.5, 17.8, 14.1, -1.0. HR-MS (EI) m/z calcd for $C_{28}H_{44}O_4Si$ [M+H$^+$] 473.3082, found 473.3085.

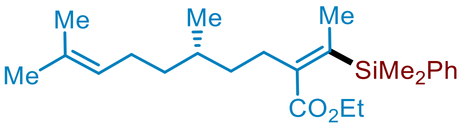

Ethyl (*S*,*Z*)-2-{1-[dimethyl(phenyl)silyl]ethylidene}-5,9-dimethyldec-8-enoate (76)

The general procedure **TP5** was followed using **3ar** (0.25 mmol) and **1a** (0.5 mmol) at 23 °C for 1 h. Purification by column chromatography (PE) yielded **76** (49 mg, 50%) as a colorless oil. ^{1}H-NMR (400 MHz, $CDCl_3$): δ = 7.47 (dd, J = 6.1, 3.0 Hz, 2H), 7.35 – 7.27 (m, 3H), 5.10 (t, J = 7.0 Hz, 1H), 3.83 (q, J = 7.1 Hz, 2H), 2.49 – 2.32 (m, 2H), 1.98 (dd, J = 16.4, 10.2 Hz, 2H), 1.83 (s, 3H), 1.68 (s, 3H), 1.60 (s, 3H), 1.41 (m, 3H), 1.26 – 1.13 (m, 2H), 1.09 (t, J = 7.1 Hz, 3H), 0.91 (d, J = 6.4 Hz, 3H), 0.38 (s, 6H). ^{13}C-NMR (100 MHz, $CDCl_3$): δ = 169.9, 144.9, 143.6, 139.8, 133.7, 131.2, 128.6, 127.6, 125.0, 60.4, 37.0, 35.7, 32.8, 28.1, 25.9, 25.7, 19.6,

19.3, 17.8, 14.1, -1.0. HR-MS (EI) m/z calcd for $C_{24}H_{38}O_2Si$ [M+H$^+$] 387.2714, found 387.2716.

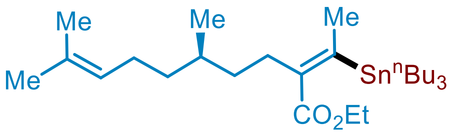

Ethyl (*R*,*Z*)-5,9-dimethyl-2-[1-(tributylstannyl)ethylidene]dec-8-enoate (77)

The general procedure **TP7** was followed using **3ar** (0.25 mmol) and **2a** (0.325 mmol) at 0 °C for 1 h. Purification by column chromatography (PE) yielded **77** (80 mg, 59%) as a colorless oil. ^{1}H-NMR (400 MHz, $CDCl_3$): δ = 5.22 – 5.04 (m, 1H), 4.18 (q, J = 7.1 Hz, 2H), 2.48 – 2.34 (m, 2H), 2.11 – 1.95 (m, 5H), 1.69 (s, 3H), 1.61 (s, 3H), 1.42 (dddd, J = 17.3, 13.2, 7.7, 5.4 Hz, 9H), 1.31 – 1.25 (m, 9H), 1.21 – 1.12 (m, 2H), 0.89 (dt, J = 14.6, 6.9 Hz, 18H). ^{13}C-NMR (100 MHz, $CDCl_3$): δ = 169.5, 162.0, 140.4, 131.2, 125.1, 60.7, 37.0, 36.5, 32.9, 29.4, 27.6, 25.9, 25.7, 25.6, 22.1, 19.7, 17.8, 14.5, 13.9, 11.8. HR-MS (EI) m/z calcd for $C_{28}H_{54}O_2Sn$ [M+H$^+$] 543.3219, found 543.3222.

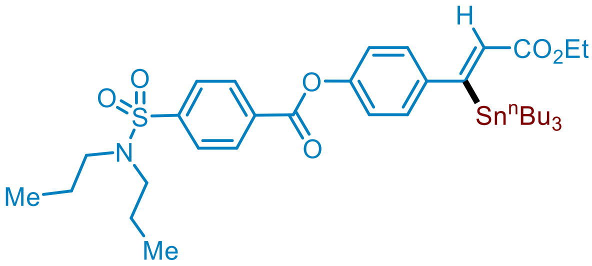

(*Z*)-4-[3-Ethoxy-3-oxo-1-(tributylstannyl)prop-1-en-1-yl]phenyl 4-(*N*,*N*-dipropylsulfamoyl)benzoate (78)

The general procedure **TP6** was followed using **3as** (0.25 mmol) and **2a** (0.325 mmol) at -20 °C for 15 min. Purification by column chromatography (petroleum ether/EtOAc 50:1) yielded **78** (133 mg, 71%) as a colorless oil. ^{1}H-NMR (400 MHz, $CDCl_3$): δ = 8.33 (d, J = 8.4 Hz, 2H), 7.95 (d, J = 8.4 Hz, 2H), 7.19 (d, J = 8.6 Hz, 2H), 7.15 – 7.09 (m, 2H), 6.76 – 6.25 (m, 1H), 4.25 (q, J = 7.1 Hz, 2H), 3.17 – 3.11 (m, 4H), 1.57 (dd, J = 15.1, 7.5 Hz, 4H), 1.43 (ddd, J = 12.5, 8.5, 6.1 Hz, 6H), 1.34 – 1.22 (m, 9H), 1.04 – 0.94 (m, 6H), 0.87 (dt, J = 14.5, 7.3 Hz, 15H). ^{13}C-NMR (100 MHz, $CDCl_3$): δ = 172.5, 167.8, 163.9, 149.7, 145.0, 144.0, 133.0, 131.3, 130.9, 127.5, 127.3, 121.2, 60.7, 50.0, 29.2, 27.4, 22.1, 14.4, 13.8, 12.1, 11.3. HR-MS (EI) m/z calcd for $C_{36}H_{55}NO_6SSn$ [M+H$^+$] 750.2845, found 750.2851.

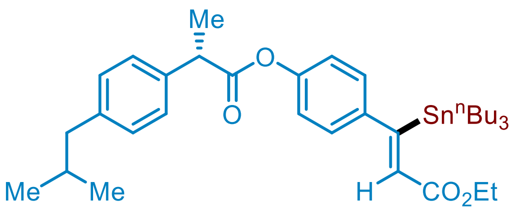

Ethyl (*S*,*Z*)-3-{4-{[2-(4-isobutylphenyl)propanoyl]oxy}phenyl}-3-(tributylstannyl)acrylate (79)

The general procedure **TP6** was followed using **3at** (0.25 mmol) and **2a** (0.325 mmol) at -20 °C for 15 min. Purification by column chromatography (petroleum ether/EtOAc 50:1) yielded **79** (91 mg, 54%) as a colorless oil. ^{1}H-NMR (400 MHz, CDCl$_3$): δ = 7.30 (d, J = 8.1 Hz, 2H), 7.14 (d, J = 8.1 Hz, 2H), 7.07 – 6.98 (m, 2H), 6.98 – 6.91 (m, 2H), 6.60 – 6.27 (m, 1H), 4.22 (q, J = 7.1 Hz, 2H), 3.93 (q, J = 7.2 Hz, 1H), 2.47 (d, J = 7.2 Hz, 2H), 1.87 (dt, J = 13.5, 6.8 Hz, 1H), 1.60 (d, J = 7.2 Hz, 3H), 1.43 – 1.33 (m, 6H), 1.31 – 1.20 (m, 9H), 0.96 – 0.87 (m, 12H), 0.83 (t, J = 7.3 Hz, 9H). ^{13}C-NMR (100 MHz, CDCl$_3$): δ = 173.3, 172.7, 167.8, 150.0, 143.4, 141.0, 137.4, 131.1, 129.7, 127.4, 127.3, 121.2, 60.7, 45.4, 45.2, 30.3, 29.2, 27.5, 22.5, 18.7, 14.5, 13.8, 12.1. HR-MS (EI) m/z calcd for $C_{36}H_{54}O_4Sn$ [M+H$^+$] 671.3117, found 671.3121.

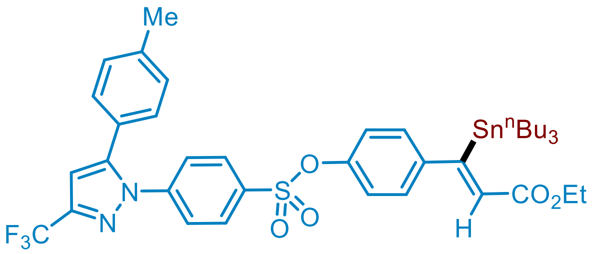

Ethyl (*Z*)-3-{4-{{{4-[5-(*p*-tolyl)-3-(trifluoromethyl)-1*H*-pyrazol-1-yl]phenyl}sulfonyl}oxy}phenyl}-3-(tributylstannyl)acrylate (80)

The general procedure **TP6** was followed using **3au** (0.25 mmol) and **2a** (0.325 mmol) at -20 °C for 15 min. Purification by column chromatography (petroleum ether/EtOAc 50:1) yielded **80** (100 mg, 47%) (64% at 23 °C) as a colorless oil. ^{1}H-NMR (400 MHz, CDCl$_3$): δ = 7.85 – 7.72 (m, 2H), 7.52 – 7.44 (m, 2H), 7.19 (d, J = 8.0 Hz, 2H), 7.09 (d, J = 8.1 Hz, 2H), 6.94 (ddd, J = 14.0, 7.8, 2.3 Hz, 4H), 6.75 (s, 1H), 6.57 – 6.30 (m, 1H), 4.23 (q, J = 7.1 Hz, 2H), 2.39 (s, 3H), 1.38 – 1.31 (m, 6H), 1.31 – 1.17 (m, 9H), 0.91 (dd, J = 9.5, 6.6 Hz, 6H), 0.82 (t, J = 7.3 Hz, 9H). ^{13}C-NMR (100 MHz, CDCl$_3$): δ = 172.1, 167.6, 148.2, 145.5, 145.4, 144.5 (q, $^2J_{C\text{-}F}$ = 39.0 Hz), 143.8, 140.1, 134.6, 131.6, 129.9, 129.7, 128.9, 127.5, 125.7, 125.5, 122.1, 118.4 (dd, $^1J_{C\text{-}F}$ = 269.4 Hz), 106.8, 60.8, 29.1, 27.4, 21.5, 14.4, 13.8, 12.1. ^{19}F-NMR (376 MHz, CDCl$_3$): δ = -62.52 (s). HR-MS (EI) m/z calcd for $C_{40}H_{49}F_3N_2O_5SSn$ [M+H$^+$] 847.2409, found

847.2415.

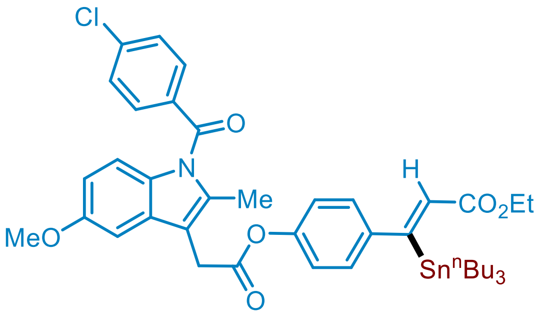

Ethyl (*Z*)-3-{4-{2-[1-(4-chlorobenzoyl)-5-methoxy-2-methyl-1*H*-indol-3-yl]acetoxy}phenyl}-3-(tributylstannyl)acrylate (81)

The general procedure **TP6** was followed using **3av** (0.25 mmol) and **2a** (0.325 mmol) at -20 °C for 15 min. Purification by column chromatography (petroleum ether/EtOAc 10:1) yielded **81** (115 mg, 56%) as a colorless oil. ^{1}H-NMR (400 MHz, $CDCl_3$): δ = 7.68 (d, J = 7.9 Hz, 2H), 7.47 (d, J = 7.9 Hz, 2H), 7.09 – 7.00 (m, 5H), 6.90 (d, J = 9.0 Hz, 1H), 6.70 (dd, J = 9.0, 2.0 Hz, 1H), 6.59 – 6.29 (m, 1H), 4.23 (q, J = 7.0 Hz, 2H), 3.90 (s, 2H), 3.84 (d, J = 0.6 Hz, 3H), 2.46 (s, 3H), 1.39 (dt, J = 8.0, 6.3 Hz, 6H), 1.33 – 1.18 (m, 9H), 0.96 – 0.91 (m, 6H), 0.83 (t, J = 7.3 Hz, 9H). ^{13}C-NMR (100 MHz, $CDCl_3$): δ = 172.6, 169.4, 168.4, 167.8, 156.3, 149.8, 143.7, 139.5, 136.3, 134.0, 131.3, 131.2, 131.0, 130.6, 129.3, 127.4, 121.1, 115.2, 112.1, 112.0, 101.3, 60.7, 55.8, 30.7, 29.2, 27.4, 14.5, 13.8, 13.6, 12.1. HR-MS (EI) m/z calcd for $C_{42}H_{52}ClNO_6Sn$ [M+H$^+$] 822.2578, found 822.2582.

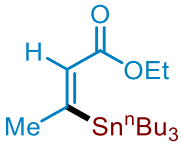

Ethyl (*Z*)-3-(tributylstannyl)but-2-enoate (84)

The general procedure **TP6** was followed using (*Z*)-**83** (0.25 mmol) and **2a** (0.325 mmol) at -20 °C for 15 min. Purification by column chromatography (PE) yielded **84** (87 mg, 86%) as a colorless oil. ^{1}H-NMR (400 MHz, $CDCl_3$): δ = 6.58 – 6.24 (m, 1H), 4.17 (q, J = 7.1 Hz, 2H), 2.18 – 2.08 (m, 3H), 1.53 – 1.41 (m, 6H), 1.39 – 1.23 (m, 9H), 0.99 – 0.94 (m, 6H), 0.88 (t, J = 7.3 Hz, 9H). ^{13}C-NMR (100 MHz, $CDCl_3$): δ = 171.7, 167.9, 129.4, 60.2, 29.4, 27.6, 27.5, 14.5, 13.9, 11.0. HR-MS (EI) m/z calcd for $C_{18}H_{36}O_2Sn$ [M+H$^+$] 405.1810, found 405.1815.

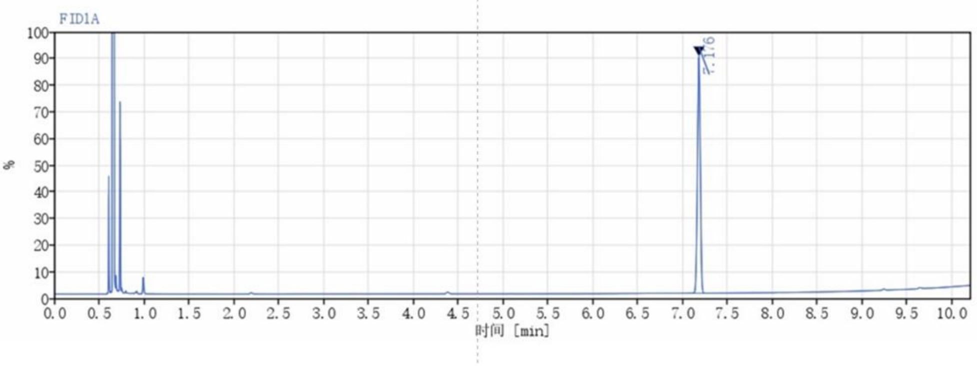

Supplementary Figure 10. GC data of compound **84**.

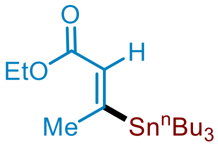

Ethyl (*E*)-3-(tributylstannyl)but-2-enoate (84')

The general procedure **TP6** was followed using (*E*)-**83** (0.25 mmol) and **2a** (0.325 mmol) at -20 °C for 15 min. Purification by column chromatography (PE) yielded **84'** (27 mg, 27%) as a colorless oil. ^{1}H-NMR (400 MHz, CDCl$_3$): δ = 6.10 – 5.83 (m, 1H), 4.16 (q, J = 7.1 Hz, 2H), 2.48 – 2.31 (m, 3H), 1.54 – 1.45 (m, 6H), 1.35 – 1.26 (m, 9H), 0.96 (dd, J = 9.5, 6.7 Hz, 6H), 0.89 (dd, J = 7.3, 5.1 Hz, 9H). ^{13}C-NMR (100 MHz, CDCl$_3$): δ = 169.3, 164.6, 128.3, 59.7, 29.1, 27.5, 22.5, 14.5, 13.8, 9.6. HR-MS (EI) m/z calcd for $C_{18}H_{36}O_2Sn$ [M+H$^+$] 405.1810, found 405.1815.

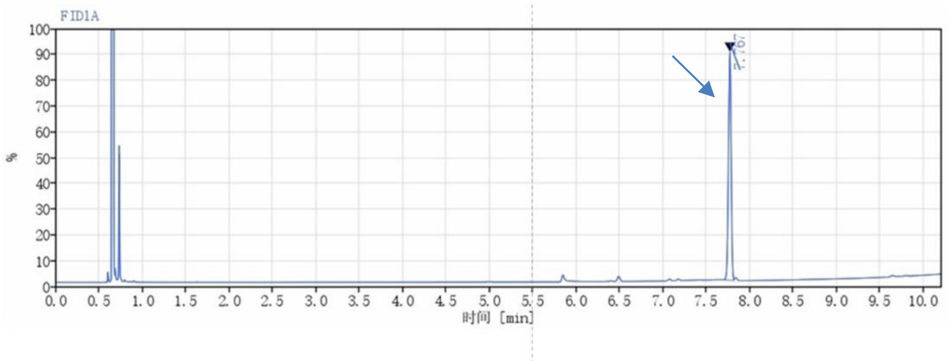

Supplementary Figure 11. GC data of compound **84'**.

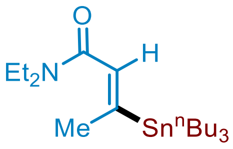

(*E*)-*N*,*N*-Diethyl-3-(tributylstannyl)but-2-enamide (86)

The general procedure **TP6** was followed using **85** (0.25 mmol) and **2a** (0.325 mmol) at -20 °C for 15 min. Purification by column chromatography (petroleum ether/EtOAc 50:1) yielded **86** (8 mg, 7%) as a colorless oil. ^{1}H-NMR (400 MHz, CDCl$_3$): δ = 6.77 (d, J = 1.6 Hz, 1H), 3.39 (m, 4H), 2.26 – 2.06 (m, 3H), 1.49 – 1.42 (m, 6H), 1.28 (m, 6H), 1.20 (t, J = 7.1 Hz, 3H), 1.11 (t, J = 7.1 Hz, 3H), 0.89 (dd, J = 7.0, 4.9 Hz, 15H). ^{13}C-NMR (100 MHz, CDCl$_3$): δ = 166.8, 166.5, 128.7, 42.0, 41.0, 29.5, 27.7, 27.3, 15.0, 14.0, 13.4, 11.7. HR-MS (EI) m/z calcd for $C_{20}H_{41}NOSn$ [M+H$^+$] 432.2283, found 432.2285.

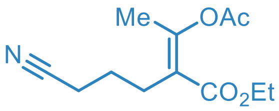

Ethyl (*Z*)-2-(1-acetoxyethylidene)-5-cyanopentanoate [(*Z*)-87]

The general procedure **TP3** was followed using **ketoester** (10.7 mmol) for 18 h. Purification by column chromatography (petroleum ether/ EtOAc 5:1) yielded (*Z*)-**87** (34%, 871 mg) as a yellow oil. ^{1}H-NMR (400 MHz, CDCl$_3$): δ = 4.18 (q, J = 7.1 Hz, 2H), 2.49 (t, J = 7.4 Hz, 2H), 2.40 (t, J = 7.0 Hz, 2H), 2.17 (s, 3H), 2.05 (s, 3H), 1.88 – 1.80 (m, 2H), 1.28 (t, J = 7.1 Hz, 3H). ^{13}C-NMR (100 MHz, CDCl$_3$): δ = 168.7, 165.7, 154.2, 119.6, 119.1, 60.9, 27.7, 24.8, 21.0, 18.8, 16.6, 14.4. HR-MS (EI) m/z calcd for $C_{12}H_{17}NO_4$ [M+H$^+$] 240.1230, found 240.1237.

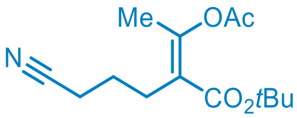

***tert*-Butyl (*Z*)-2-(1-acetoxyethylidene)-5-cyanopentanoate [(*Z*)-88]**

The general procedure **TP3** was followed using **ketoester** (5 mmol) for 18 h. Purification by column chromatography (petroleum ether/ EtOAc 50:1) yielded (*Z*)-**88** (66%, 887 mg) as a colorless oil. ^{1}H-NMR (400 MHz, CDCl$_3$): δ = 2.43 – 2.39 (m, 2H), 2.38 (t, J = 5.9 Hz, 2H), 2.14 (s, 3H), 1.98 (s, 3H), 1.85 – 1.76 (m, 2H), 1.44 (s, 9H). ^{13}C-NMR (100 MHz, CDCl$_3$): δ = 168.6, 165.3, 151.6, 120.6, 119.6, 81.4, 28.2, 27.8, 24.6, 21.0, 18.1, 16.4. HR-MS (EI) m/z calcd for $C_{14}H_{21}NO_4$ [M+H$^+$] 268.1543, found 268.1551.

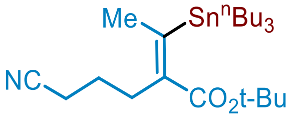

***tert*-Butyl (*Z*)-5-cyano-2-[1-(tributylstannyl)ethylidene]pentanoate (90)**

The general procedure **TP7** was followed using (*Z*)-**88** (0.25 mmol) and **2a** (0.325 mmol) at

0 °C for 2 h. Purification by column chromatography (petroleum ether/EtOAc 50:1) yielded **90** (39 mg, 31%) as a colorless oil. ^{1}H-NMR (400 MHz, $CDCl_3$): δ = 2.62 – 2.48 (m, 2H), 2.31 (t, J = 7.1 Hz, 2H), 2.13 – 1.97 (m, 3H), 1.81 – 1.68 (m, 2H), 1.48 (s, 9H), 1.48 – 1.36 (m, 6H), 1.27 (dd, J = 14.6, 7.3 Hz, 6H), 0.93 – 0.83 (m, 15H). ^{13}C-NMR (100 MHz, $CDCl_3$): δ = 167.8, 164.7, 138.1, 119.9, 80.7, 29.4, 28.2, 27.6, 26.9, 25.4, 22.3, 17.0, 13.9, 12.1. HR-MS (EI) m/z calcd for $C_{24}H_{45}NO_2Sn$ [M+H$^+$] 500.2545, found 500.2549.

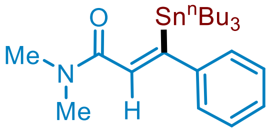

(*Z*)-*N*,*N*-Dimethyl-3-phenyl-3-(tributylstannyl)acrylamide (93)

The general procedure **TP7** was followed using (*Z*)-**91** (0.25 mmol) and **2a** (0.325 mmol) at 23 °C for 1 h. Purification by column chromatography (petroleum ether/EtOAc 50:1) yielded **93** (50 mg, 43%) as a colorless oil. ^{1}H-NMR (400 MHz, $CDCl_3$): δ = 7.35 – 7.26 (m, 2H), 7.24 – 7.18 (m, 1H), 7.05 (dt, J = 8.0, 1.7 Hz, 2H), 7.02 – 6.74 (m, 1H), 3.13 (s, 3H), 3.03 (s, 3H), 1.45 – 1.34 (m, 6H), 1.23 (m, 6H), 0.90 (m, 6H), 0.82 (t, J = 7.3 Hz, 9H). ^{13}C-NMR (100 MHz, $CDCl_3$): δ = 169.6, 167.5, 147.0, 129.9, 128.1, 126.4, 126.4, 37.4, 36.2, 29.3, 27.6, 13.9, 12.6. HR-MS (EI) m/z calcd for $C_{23}H_{39}NOSn$ [M+H$^+$] 466.2126, found 466.2130.

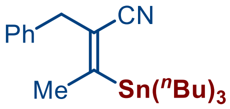

(*Z*)-2-Benzyl-3-(tributylstannyl)but-2-enenitrile (96)

The general procedure **TP7** was followed using (*Z*)-**94** (0.25 mmol) and **2a** (0.325 mmol) at 0 °C for 1 h. Purification by column chromatography (petroleum ether/EtOAc 20:1) yielded **96** (28 mg, 25%) as a colorless oil. ^{1}H-NMR (400 MHz, $CDCl_3$): δ = 7.33 – 7.29 (m, 2H), 7.25 – 7.19 (m, 3H), 3.65 (s, 2H), 2.18 – 2.02 (m, 3H), 1.56 – 1.46 (m, 6H), 1.32 (dd, J = 14.8, 7.3 Hz, 6H), 1.16 – 1.03 (m, 6H), 0.89 (t, J = 7.3 Hz, 9H). ^{13}C-NMR (100 MHz, $CDCl_3$): δ = 166.2, 137.7, 128.8, 128.5, 126.8, 122.8, 121.0, 36.5, 29.2, 27.4, 22.1, 13.8, 10.4. HR-MS (EI) m/z calcd for $C_{23}H_{37}NSn$ [M+H$^+$] 448.2021, found 448.2025.

2.7 Late-Stage Modifications of Alkenyl Silanes and Alkenyl Stannanes:

Figure S8: Synthetic Applicability of Alkenyl Silanes by Hiyama coupling and Alkenyl Stannanes by MKS Reaction.[14]

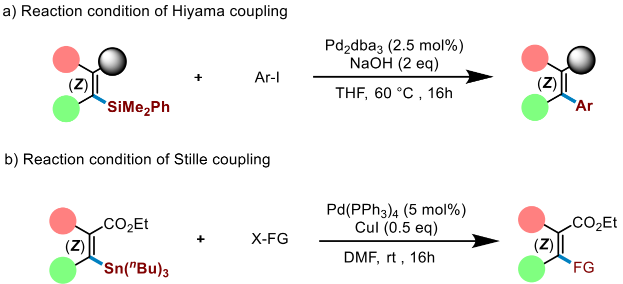

Supplementary Figure 12. Reaction conditions for Hiyama and Stille cross-coupling.

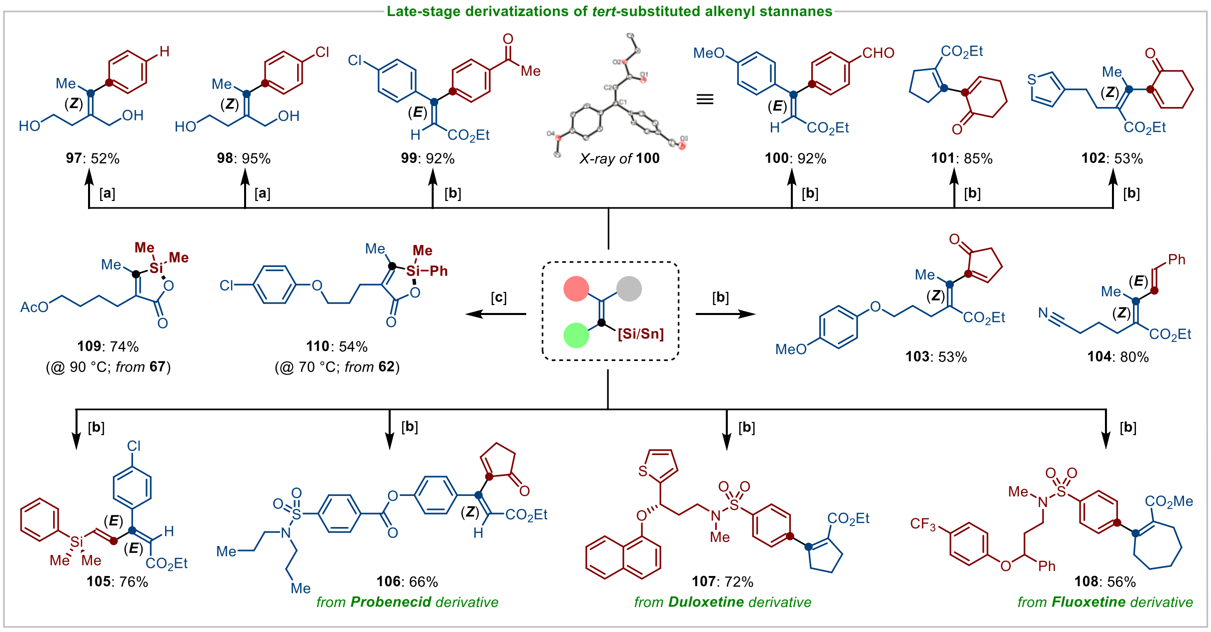

Supplementary Figure 13. Late-Stage Modifications of Alkenyl Silanes and Alkenyl Stannanes.

2.8 Characterization Data of Products 97–110

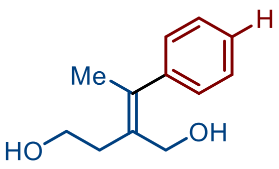

(*Z*)-2-(1-phenylethylidene)butane-1,4-diol (97)

The general procedure **TP12** was followed using **33** (0.1 mmol) and **iodobenzene** (0.15 mmol) at 60 °C for 16 h. Purification by column chromatography (petroleum ether/EtOAc 2:1) yielded **97** (10 mg, 52%) as a colorless oil. ^{1}H-NMR (400 MHz, $CDCl_3$): δ = 7.33 (t, J = 7.3 Hz, 2H), 7.26 (s, 1H), 7.21 – 7.14 (m, 2H), 3.94 (s, 2H), 3.84 (t, J = 5.8 Hz, 2H), 2.61 (t, J = 5.7 Hz, 2H), 2.29 (s, 2H), 2.03 (s, 3H). ^{13}C-NMR (100 MHz, $CDCl_3$): δ = 143.6, 138.4, 132.3, 128.3, 128.0, 126.8, 64.0, 62.0, 35.0, 21.2. HR-MS (EI) m/z calcd for $C_{12}H_{16}O_2$ [M+H$^+$] 193.1223, found 193.1225.

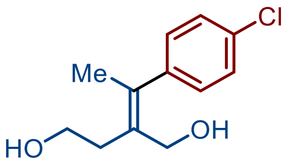

(*Z*)-2-[1-(4-chlorophenyl)ethylidene]butane-1,4-diol (98)

The general procedure **TP12** was followed using **33** (0.1 mmol) and **1-chloro-4-iodobenzene** (0.15 mmol) at 60 °C for 16 h. Purification by column chromatography (petroleum ether/EtOAc 2:1) yielded **98** (19 mg, 56%) as a colorless oil. ^{1}H-NMR (400 MHz, $CDCl_3$): δ = 7.33 – 7.26 (m, 2H), 7.16 – 7.09 (m, 2H), 3.91 (s, 2H), 3.82 (t, J = 5.7 Hz, 2H), 2.76 (s, 2H), 2.59 (t, J = 5.7 Hz, 2H), 2.00 (s, 3H). ^{13}C-NMR (100 MHz, $CDCl_3$): δ = 142.0, 137.1, 133.0, 132.7, 129.5, 128.5, 63.9, 61.8, 35.0, 21.1. HR-MS (EI) m/z calcd for $C_{12}H_{15}ClO_2$ [M+H$^+$] 227.0833, found 227.0835.

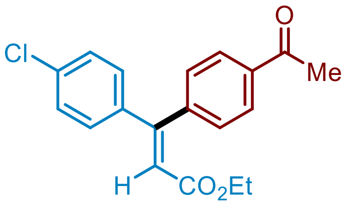

Ethyl (*E*)-3-(4-acetylphenyl)-3-(4-chlorophenyl)acrylate (99)

The general procedure **TP8** was followed using **44** (0.1 mmol) and **1-(4-iodophenyl)ethan-1-one** (0.12 mmol) at 23 °C for 16 h. Purification by column chromatography (petroleum ether/EtOAc 20:1) yielded **99** (30 mg, 92%) as a colorless oil. ^{1}H-NMR (400 MHz, $CDCl_3$): δ = 8.07 – 7.85 (m, 2H), 7.37 – 7.26 (m, 4H), 7.24 – 7.15 (m, 2H), 6.41 (s, 1H), 4.06 (q, J = 7.1 Hz, 2H), 2.64 (s, 3H), 1.14 (t, J = 7.1 Hz, 3H). ^{13}C-NMR (100 MHz, $CDCl_3$): δ = 197.7, 165.6, 154.3, 143.7, 138.5, 136.8, 136.1, 129.5, 129.4, 129.0, 128.2, 118.5, 60.5, 26.8, 14.1. HR-MS (EI) m/z calcd for $C_{19}H_{17}ClO_3$ [M+H$^+$] 329.0939, found 329.0943.

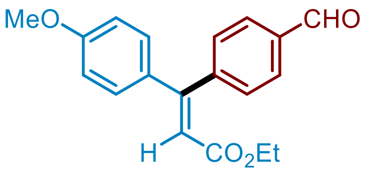

Ethyl (*E*)-3-(4-formylphenyl)-3-(4-methoxyphenyl)acrylate (100)

The general procedure **TP8** was followed using **41** (0.147 mmol) and **4-iodobenzaldehyde** (0.18 mmol) at 23 °C for 16 h. Purification by column chromatography (petroleum ether/EtOAc 20:1) yielded **100** (43 mg, 95%) as a yellow solid. ^{1}H-NMR (400 MHz, $CDCl_3$): δ = 10.06 (s, 1H), 7.92 (d, J = 8.3 Hz, 2H), 7.38 (d, J = 8.1 Hz, 2H), 7.25 – 7.16 (m, 2H), 6.93 – 6.77 (m, 2H), 6.39 (s, 1H), 4.04 (q, J = 7.1 Hz, 2H), 3.81 (s, 3H), 1.12 (t, J = 7.1 Hz, 3H). ^{13}C-NMR (100 MHz, $CDCl_3$): δ = 192.0, 165.9, 161.2, 155.1, 146.1, 135.8, 132.0, 129.8, 129.7, 129.5, 116.0, 114.1, 60.2, 55.5, 14.1. HR-MS (EI) m/z calcd for $C_{19}H_{18}O_4$ [M+H$^+$] 311.1278, found 311.1282.

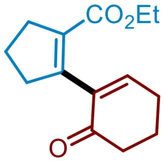

Ethyl 2-(6-oxocyclohex-1-en-1-yl)cyclopent-1-ene-1-carboxylate (101)

The general procedure **TP8** was followed using **38** (0.1 mmol) and **2-iodocyclohex-2-en-1-one** (0.12 mmol) at 23 °C for 16 h. Purification by column chromatography (petroleum ether/EtOAc 20:1) yielded **101** (20 mg, 85%) as a colorless oil. ^{1}H-NMR (400 MHz, $CDCl_3$): δ = 6.77 (t, J = 4.2 Hz, 1H), 4.09 (q, J = 7.1 Hz, 2H), 2.74 (ddd, J = 7.9, 4.9, 2.4 Hz, 2H), 2.62 (tt, J = 8.0, 2.4 Hz, 2H), 2.57 – 2.48 (m, 2H), 2.47 – 2.38 (m, 2H), 2.05 (dt, J = 12.4, 6.1 Hz, 2H), 1.99 – 1.84 (m, 2H), 1.22 (t, J = 7.1 Hz, 3H). ^{13}C-NMR (100 MHz, $CDCl_3$): δ = 197.0, 165.7, 149.6, 145.7, 138.1, 131.7, 60.0, 39.6, 38.8, 34.1, 26.1, 22.9, 21.9, 14.4. HR-MS (EI) m/z calcd for $C_{14}H_{18}O_3$ [M+H$^+$] 235.1329, found 235.1333.

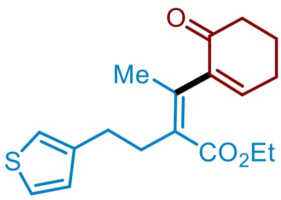

Ethyl (*Z*)-3-(6-oxocyclohex-1-en-1-yl)-2-[2-(thiophen-3-yl)ethyl]but-2-enoate (102)

The general procedure **TP8** was followed using **56** (0.1 mmol) and **2-iodocyclohex-2-en-1-one** (0.12 mmol) at 23 °C for 16 h. Purification by column chromatography (petroleum ether/EtOAc

20:1) yielded **102** (17 mg, 53%) as a colorless oil. ^{1}H-NMR (400 MHz, $CDCl_3$): δ = 7.23 (dd, J = 4.7, 3.1 Hz, 1H), 6.98 (d, J = 4.9 Hz, 2H), 6.53 (t, J = 4.2 Hz, 1H), 4.05 (q, J = 7.1 Hz, 2H), 2.79 (t, J = 7.6 Hz, 2H), 2.67 (t, J = 7.6 Hz, 2H), 2.49 (dd, J = 8.6, 4.9 Hz, 2H), 2.37 (dd, J = 10.3, 6.0 Hz, 2H), 2.08 – 1.97 (m, 2H), 1.76 (s, 3H), 1.22 (t, J = 7.1 Hz, 3H). ^{13}C-NMR (100 MHz, $CDCl_3$): δ = 197.5, 168.7, 144.1, 143.1, 142.6, 141.9, 130.6, 128.7, 125.2, 120.8, 60.3, 38.8, 31.0, 29.4, 26.0, 22.9, 21.1, 14.4. HR-MS (EI) m/z calcd for $C_{18}H_{22}O_3S$ [M+H$^+$] 319.1362, found 319.1368.

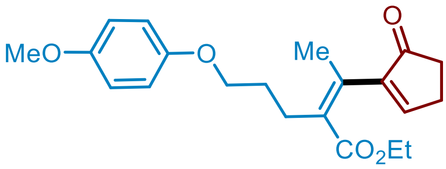

Ethyl (*Z*)-5-(4-methoxyphenoxy)-2-[1-(5-oxocyclopent-1-en-1-yl)ethylidene]pentanoate (103)

The general procedure **TP8** was followed using **58** (0.09 mmol) and **2-iodocyclopent-2-en-1-one** (0.108 mmol) at 23 °C for 16 h. Purification by column chromatography (petroleum ether/EtOAc 5:1) yielded **103** (17 mg, 53%) as a colorless oil. ^{1}H-NMR (400 MHz, $CDCl_3$): δ = 7.34 (t, J = 2.8 Hz, 1H), 6.83 (s, 4H), 4.06 (q, J = 7.1 Hz, 2H), 3.93 (t, J = 6.1 Hz, 2H), 3.76 (s, 3H), 2.71 – 2.59 (m, 4H), 2.53 – 2.37 (m, 2H), 1.99 – 1.83 (m, 5H), 1.20 (t, J = 7.1 Hz, 3H). ^{13}C-NMR (100 MHz, $CDCl_3$): δ = 206.5, 168.8, 156.9, 153.8, 153.2, 148.7, 135.5, 132.2, 115.5, 114.8, 67.5, 60.5, 55.9, 34.8, 28.5, 26.7, 26.5, 20.6, 14.3. HR-MS (EI) m/z calcd for $C_{21}H_{26}O_5$ [M+H$^+$] 359.1853, found 359.1857.

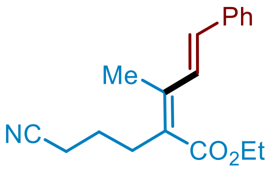

Ethyl (2*Z*,4*E*)-2-(3-cyanopropyl)-3-methyl-5-phenylpenta-2,4-dienoate (104)

The general procedure **TP8** was followed using **65** (0.127 mmol) and **(*E*)-(2-bromovinyl)benzene** (0.16 mmol) at 23 °C for 16 h. Purification by column chromatography (petroleum ether/EtOAc 20:1) yielded **104** (29 mg, 80%) as a colorless oil. ^{1}H-NMR (400 MHz, $CDCl_3$): δ = 7.62 (d, J = 16.1 Hz, 1H), 7.49 – 7.41 (m, 2H), 7.33 (t, J = 7.5 Hz, 2H), 7.27 – 7.24 (m, 1H), 6.79 (d, J = 16.1 Hz, 1H), 4.30 (q, J = 7.1 Hz, 2H), 2.73 – 2.56 (m, 2H), 2.40 (t, J = 7.0 Hz, 2H), 2.09 (s, 3H), 1.85 (t, J = 7.6 Hz, 2H), 1.36 (t, J = 7.1 Hz, 3H). ^{13}C-NMR (100 MHz, $CDCl_3$): δ = 169.2, 141.1, 137.3, 132.3, 129.6, 128.8, 128.2, 128.2, 127.0, 119.6, 60.9, 29.7,

24.9, 16.9, 15.3, 14.5. HR-MS (EI) m/z calcd for $C_{18}H_{21}NO_2$ [M+H$^+$] 284.1645, found 284.1649.

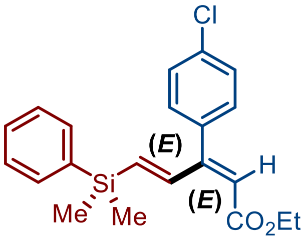

Ethyl (2*E*,4*E*)-3-(4-chlorophenyl)-5-[dimethyl(phenyl)silyl]penta-2,4-dienoate (105)

The general procedure **TP8** was followed using **44** (0.128 mmol) and **(*E*)-(2-iodovinyl)dimethyl(phenyl)silane** (0.15 mmol) at 23 °C for 16 h. Purification by column chromatography (petroleum ether/EtOAc 50:1) yielded **105** (36 mg, 76%) as a colorless oil. ^{1}H-NMR (400 MHz, $CDCl_3$): δ = 8.09 (d, J = 19.2 Hz, 1H), 7.46 – 7.38 (m, 2H), 7.32 – 7.20 (m, 5H), 7.13 (d, J = 8.4 Hz, 2H), 6.07 (d, J = 19.2 Hz, 1H), 5.73 (s, 1H), 4.14 (q, J = 7.1 Hz, 2H), 1.22 (t, J = 7.1 Hz, 3H), 0.33 (s, 6H). ^{13}C-NMR (100 MHz, $CDCl_3$): δ = 166.0, 154.8, 141.4, 141.1, 138.2, 138.0, 134.6, 133.9, 130.5, 129.3, 128.6, 128.0, 118.8, 60.4, 14.4, -2.7. HR-MS (EI) m/z calcd for $C_{21}H_{23}ClO_2Si$ [M+H$^+$] 371.1229, found 371.1231.

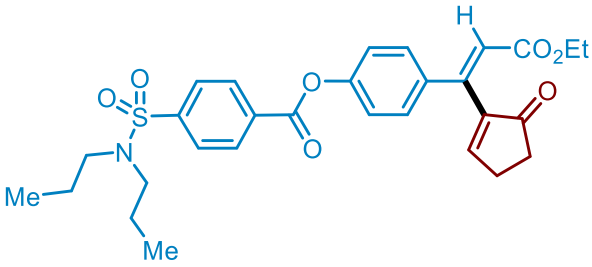

(*Z*)-4-[3-Ethoxy-3-oxo-1-(5-oxocyclopent-1-en-1-yl)prop-1-en-1-yl]phenyl 4-(*N*,*N*-dipropy lsulfamoyl)benzoate (106)

The general procedure **TP8** was followed using **78** (0.185 mmol) and **2-iodocyclopent-2-en-1-one** (0.23 mmol) at 23 °C for 16 h. Purification by column chromatography (petroleum ether/EtOAc 5:1) yielded **106** (66 mg, 66%) as a colorless oil. ^{1}H-NMR (400 MHz, $CDCl_3$): δ = 8.31 (d, J = 8.6 Hz, 2H), 7.95 (d, J = 8.5 Hz, 2H), 7.52 – 7.41 (m, 3H), 7.25 – 7.18 (m, 2H), 6.39 (s, 1H), 4.16 (q, J = 7.1 Hz, 2H), 3.18 – 3.09 (m, 4H), 2.81 (td, J = 4.7, 2.6 Hz, 2H), 2.67 – 2.57 (m, 2H), 1.58 (dt, J = 15.1, 7.5 Hz, 4H), 1.28 (t, J = 7.1 Hz, 3H), 0.89 (t, J = 7.4 Hz, 6H). ^{13}C-NMR (100 MHz, $CDCl_3$): δ = 205.8, 165.7, 163.7, 160.8, 151.7, 145.9, 145.3, 145.2, 137.3, 132.6, 131.0, 128.9, 127.3, 121.9, 120.2, 60.5, 50.1, 34.7, 27.4, 22.1, 14.3, 11.3. HR-MS (EI) m/z calcd for $C_{29}H_{33}NO_7S$ [M+H$^+$] 540.2050, found 540.2054.

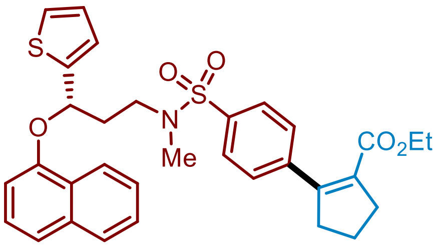

Ethyl (*S*)-2-{4-{N-methyl-N-[3-(naphthalen-1-yloxy)-3-(thiophen-2-yl)propyl]sulfamoyl}phenyl}cyclopent-1-ene-1-carboxylate (107)

The general procedure **TP8** was followed using **38** (0.1 mmol) and **(*S*)-4-iodo-N-methyl-N-[3-(naphthalen-1-yloxy)-3-(thiophen-2-yl)propyl]benzenesulfonamide** (0.12 mmol) at 23 °C for 16 h. Purification by column chromatography (petroleum ether/EtOAc 10:1) yielded **107** (42 mg, 72%) as a colorless oil. ^{1}H-NMR (400 MHz, $CDCl_3$): δ = 8.30 (dd, J = 6.2, 3.5 Hz, 1H), 7.77 (dd, J = 6.0, 3.4 Hz, 1H), 7.71 – 7.67 (m, 2H), 7.50 – 7.45 (m, 2H), 7.43 – 7.38 (m, 3H), 7.30 – 7.25 (m, 1H), 7.20 (dd, J = 5.0, 1.1 Hz, 1H), 7.13 – 7.10 (m, 1H), 6.93 (dd, J = 5.0, 3.5 Hz, 1H), 6.88 (d, J = 7.6 Hz, 1H), 5.79 (dd, J = 7.8, 5.0 Hz, 1H), 4.04 (q, J = 7.1 Hz, 2H), 3.38 – 3.19 (m, 2H), 2.87 – 2.80 (m, 4H), 2.77 (s, 3H), 2.51 (dq, J = 7.6, 5.9 Hz, 1H), 2.41 – 2.26 (m, 1H), 2.00 (dt, J = 15.2, 7.7 Hz, 2H), 1.06 (t, J = 7.1 Hz, 3H). ^{13}C-NMR (100 MHz, $CDCl_3$): δ = 165.7, 153.1, 151.2, 144.5, 142.0, 136.3, 134.7, 131.7, 128.6, 127.7, 127.0, 126.8, 126.4, 126.2, 125.9, 125.4, 125.3, 125.1, 122.1, 121.0, 107.3, 73.8, 60.3, 47.3, 40.3, 37.8, 35.9, 35.3, 22.1, 14.1. HR-MS (EI) m/z calcd for $C_{32}H_{33}NO_5S_2$ [M+H$^+$] 576.1873, found 576.1875.

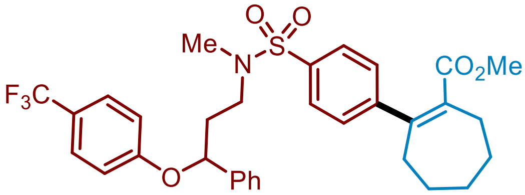

Methyl 2-{4-{N-methyl-N-{3-phenyl-3-[4-(trifluoromethyl)phenoxy]propyl}sulfamoyl}phenyl}cyclohept-1-ene-1-carboxylate (108)

The general procedure **TP8** was followed using **39** (0.149 mmol) and 4-iodo-N-methyl-N-{3-phenyl-3-[4-(trifluoromethyl)phenoxy]propyl}benzenesulfonamide (0.179 mmol) at 23 ℃ for 16 h. Purification by column chromatography (petroleum ether/EtOAc 10:1) yielded **108** (50 mg, 56%) as a colorless oil. ^{1}H-NMR (400 MHz, $CDCl_3$): δ = 7.68 (d, J = 8.4 Hz, 2H), 7.43 (d, J = 8.6 Hz, 2H), 7.34 (d, J = 4.4 Hz, 4H), 7.29 (dd, J = 8.3, 4.2 Hz, 1H), 7.25 (d, J = 8.5 Hz, 2H), 6.91 (d, J = 8.6 Hz, 2H), 5.31 (dd, J = 8.6, 4.1 Hz, 1H), 3.34 (s, 3H), 3.29 (dd, J = 14.0, 7.0 Hz, 1H), 3.18 – 3.09 (m, 1H), 2.75 (s, 3H), 2.59 (dd, J = 10.5, 4.6 Hz, 4H), 2.22 (qd, J = 7.3, 5.3 Hz, 1H), 2.10 (dtd, J = 11.6, 7.4, 4.2 Hz, 1H), 1.87 (dt, J = 11.7, 5.9 Hz, 2H), 1.70 –

1.63 (m, 4H). ^{13}C-NMR (100 MHz, $CDCl_3$): δ = 170.7, 160.4, 149.5, 149.5, 140.5, 135.5, 135.3, 129.0, 128.2, 127.4, 127.4, 126.9 (q, $^3J_{C\text{-}F}$ = 3.6 Hz), 126.0, 124.5 (q, $^1J_{C\text{-}F}$ = 270.8 Hz), 123.2 (q, $^2J_{C\text{-}F}$ = 32.6 Hz), 116.0, 51.5, 47.3, 37.4, 37.0, 35.7, 32.4, 31.0, 26.3, 25.8. ^{19}F-NMR (376 MHz, $CDCl_3$): δ = -61.58 (s). HR-MS (EI) m/z calcd for $C_{32}H_{34}F_3NO_5S$ [M+H$^+$] 602.2183, found 602.2185.

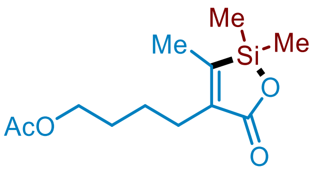

4-(2,2,3-Trimethyl-5-oxo-2,5-dihydro-1,2-oxasilol-4-yl)butyl acetate (109)

The general procedure **TP11** was followed using **24** (0.16 mmol) and **I$_2$** (0.24 mmol) at 90 °C overnight. Purification by column chromatography (petroleum ether/EtOAc 10:1) yielded **109** (30 mg, 74%) as a colorless oil. ^{1}H-NMR (400 MHz, $CDCl_3$): δ = 4.07 (t, J = 6.5 Hz, 2H), 2.45 – 2.36 (m, 2H), 2.04 (s, 3H), 1.99 (s, 3H), 1.70 – 1.62 (m, 2H), 1.52 (m, 2H), 0.40 (s, 6H). ^{13}C-NMR (100 MHz, $CDCl_3$): δ = 171.3, 169.7, 155.2, 145.8, 64.3, 28.6, 25.7, 25.0, 21.1, 13.9, -2.7. HR-MS (EI) m/z calcd for $C_{12}H_{20}O_4Si$ [M+H$^+$] 257.1204, found 257.1207.

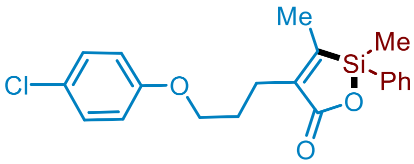

4-[3-(4-Chlorophenoxy)propyl]-2,3-dimethyl-2-phenyl-1,2-oxasilol-5(2*H*)-one (110)

The general procedure **TP11** was followed using **28** (0.2 mmol) and **I$_2$** (0.3 mmol) at 70 °C overnight. Purification by column chromatography (petroleum ether/EtOAc 10:1) yielded **110** (40 mg, 54%) as a colorless oil. ^{1}H-NMR (400 MHz, $CDCl_3$): δ = 7.58 – 7.46 (m, 3H), 7.45 – 7.36 (m, 2H), 7.21 (d, J = 8.5 Hz, 2H), 6.79 (d, J = 8.5 Hz, 2H), 3.92 (t, J = 6.0 Hz, 2H), 2.65 (t, J = 7.3 Hz, 2H), 2.10 – 2.00 (m, 2H), 1.98 (s, 3H), 0.70 (s, 3H). ^{13}C-NMR (100 MHz, $CDCl_3$): δ = 169.8, 157.6, 154.5, 145.9, 134.1, 131.7, 130.4, 129.5, 128.6, 125.7, 115.8, 67.4, 27.8, 22.8, 14.1, -5.1. HR-MS (EI) m/z calcd for $C_{20}H_{21}ClO_3Si$ [M+H$^+$] 373.1021, found 373.1023.

Supplementary Notes

3.1 NMR Spectra

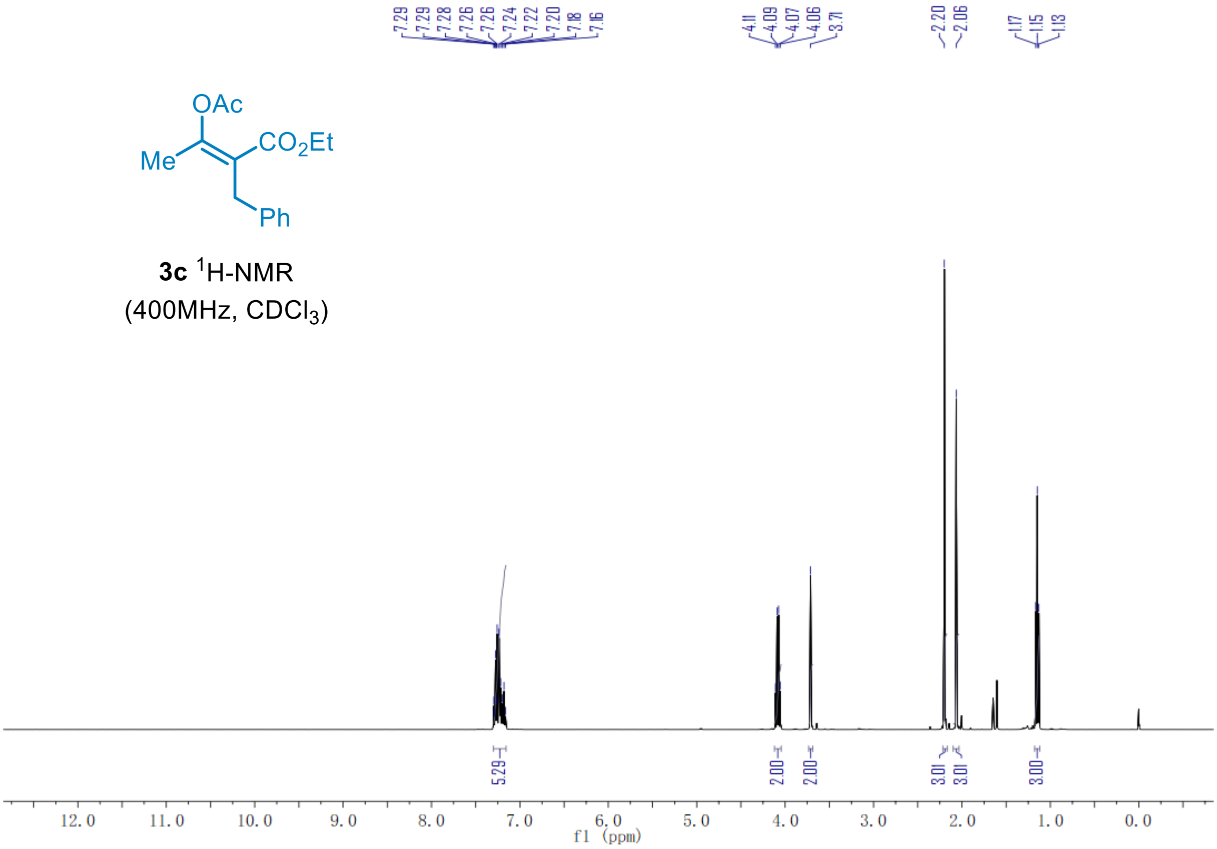

Supplementary Figure 14. **^{1}H-NMR** (400 MHz, $CDCl_3$, 298K) of **3c**

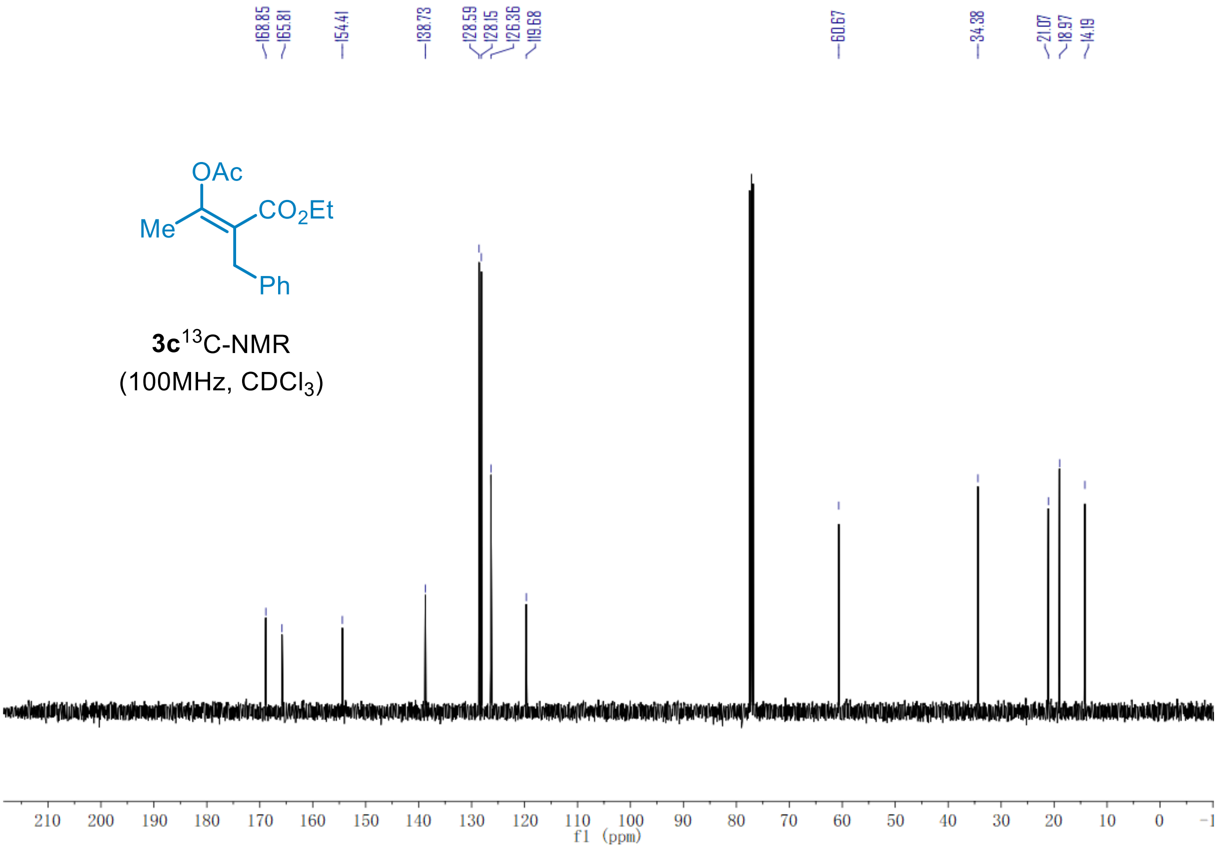

Supplementary Figure 15. **^{13}C-NMR** (100 MHz, $CDCl_3$, 298K) of **3c**

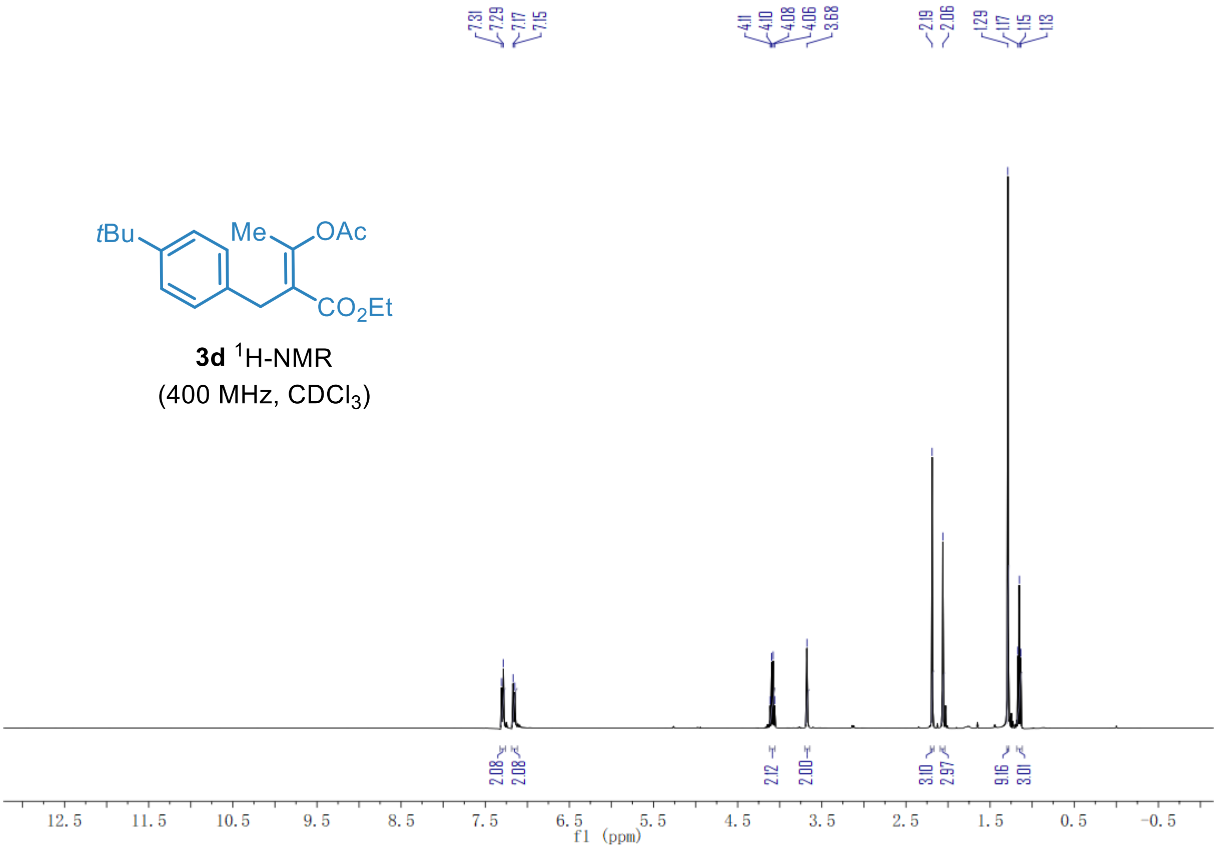

Supplementary Figure 16. **^{1}H-NMR** (400 MHz, $CDCl_3$, 298K) of **3d**

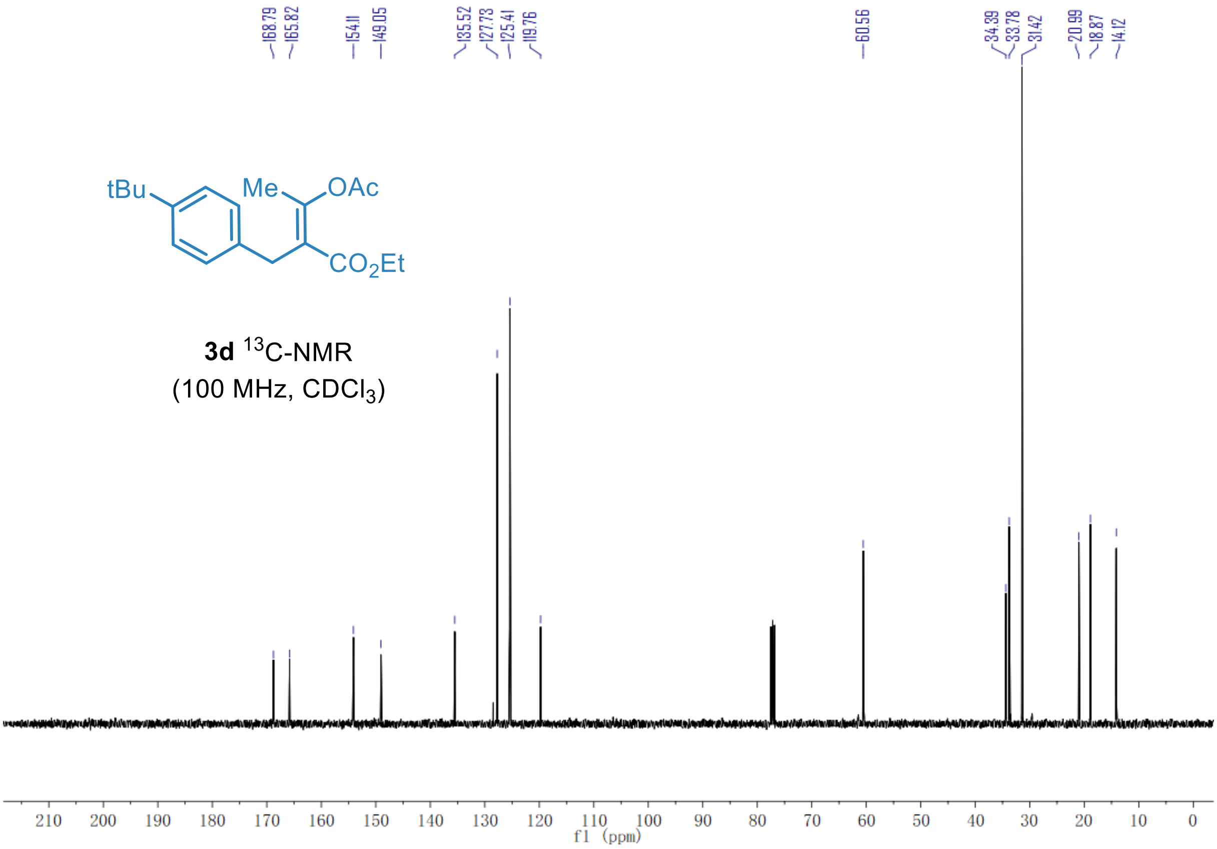

Supplementary Figure 17. **^{13}C-NMR** (100 MHz, $CDCl_3$, 298K) of **3d**

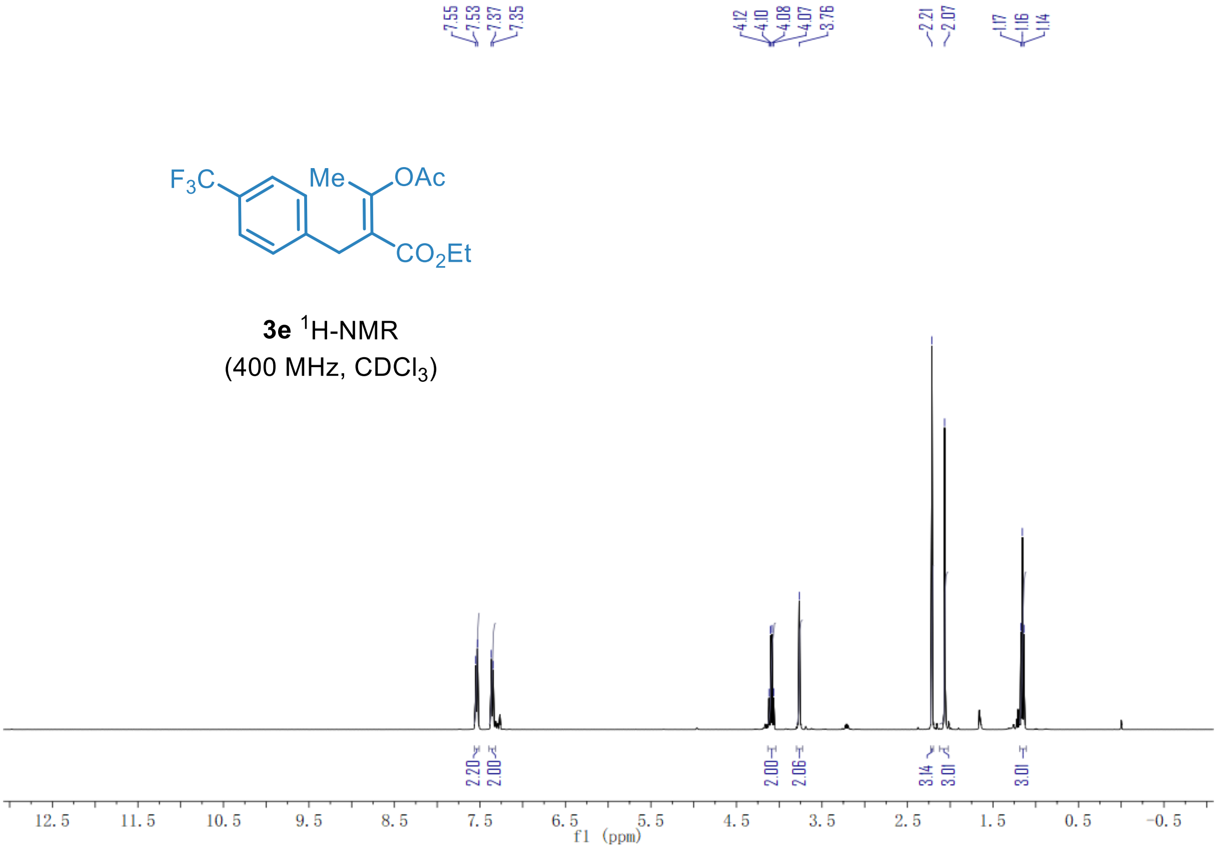

Supplementary Figure 18. **^{1}H-NMR** (400 MHz, $CDCl_3$, 298K) of **3e**

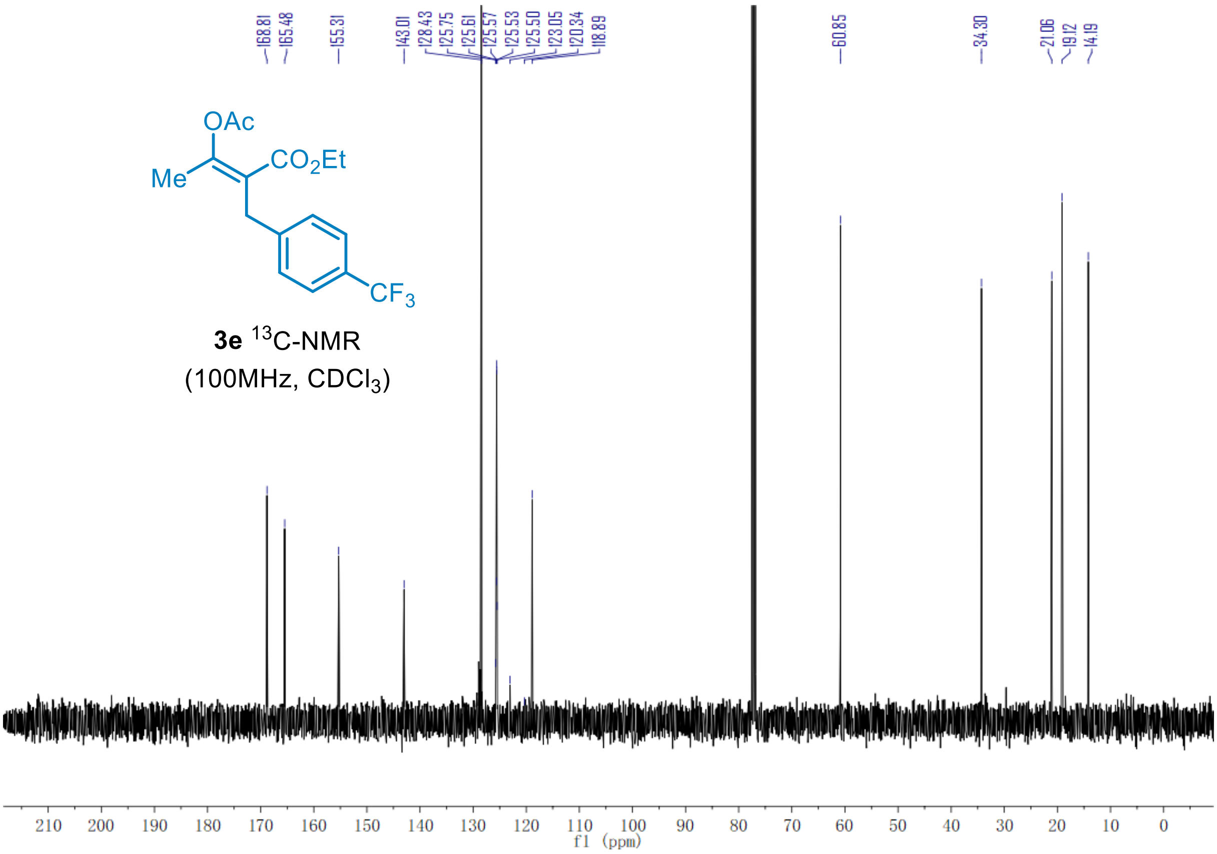

Supplementary Figure 19. **^{13}C-NMR** (100 MHz, $CDCl_3$, 298K) of **3e**

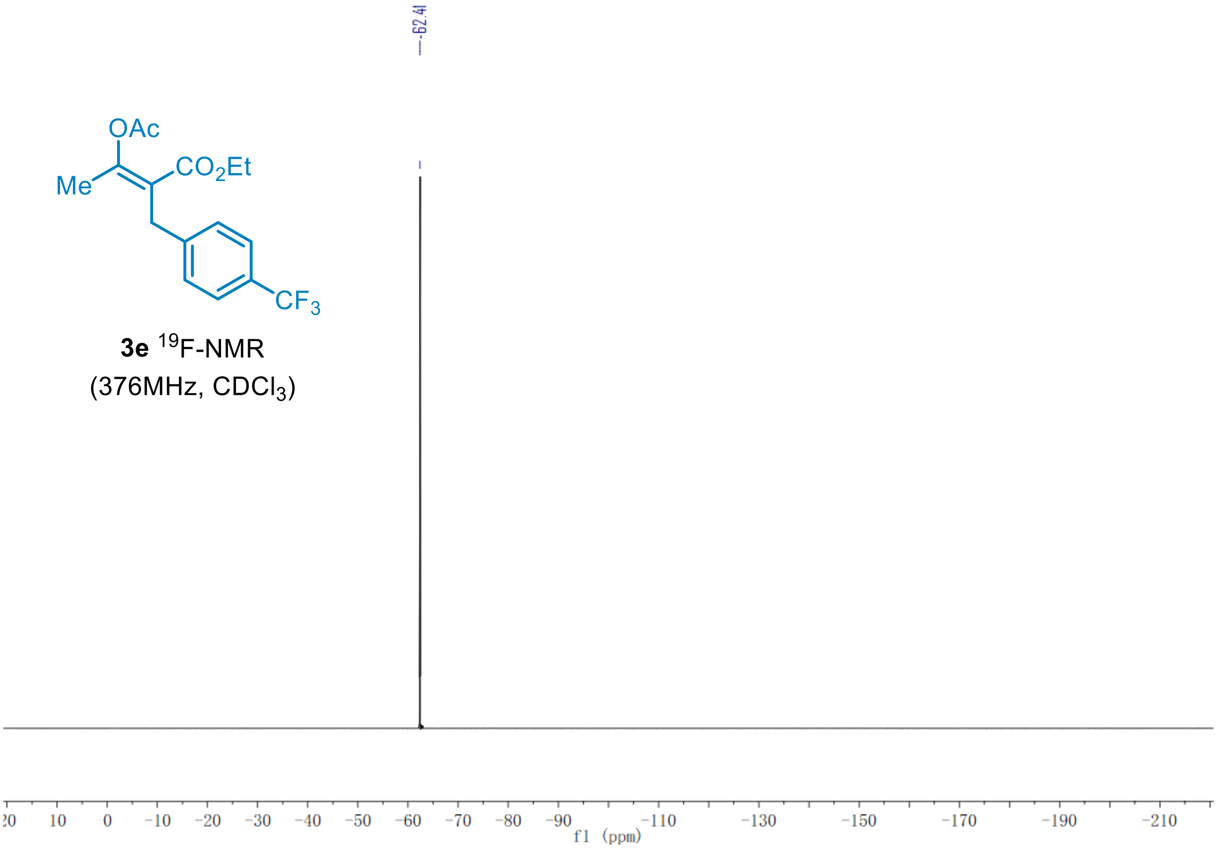

Supplementary Figure 20. **^{19}F-NMR** (376 MHz, $CDCl_3$, 298K) of **3e**

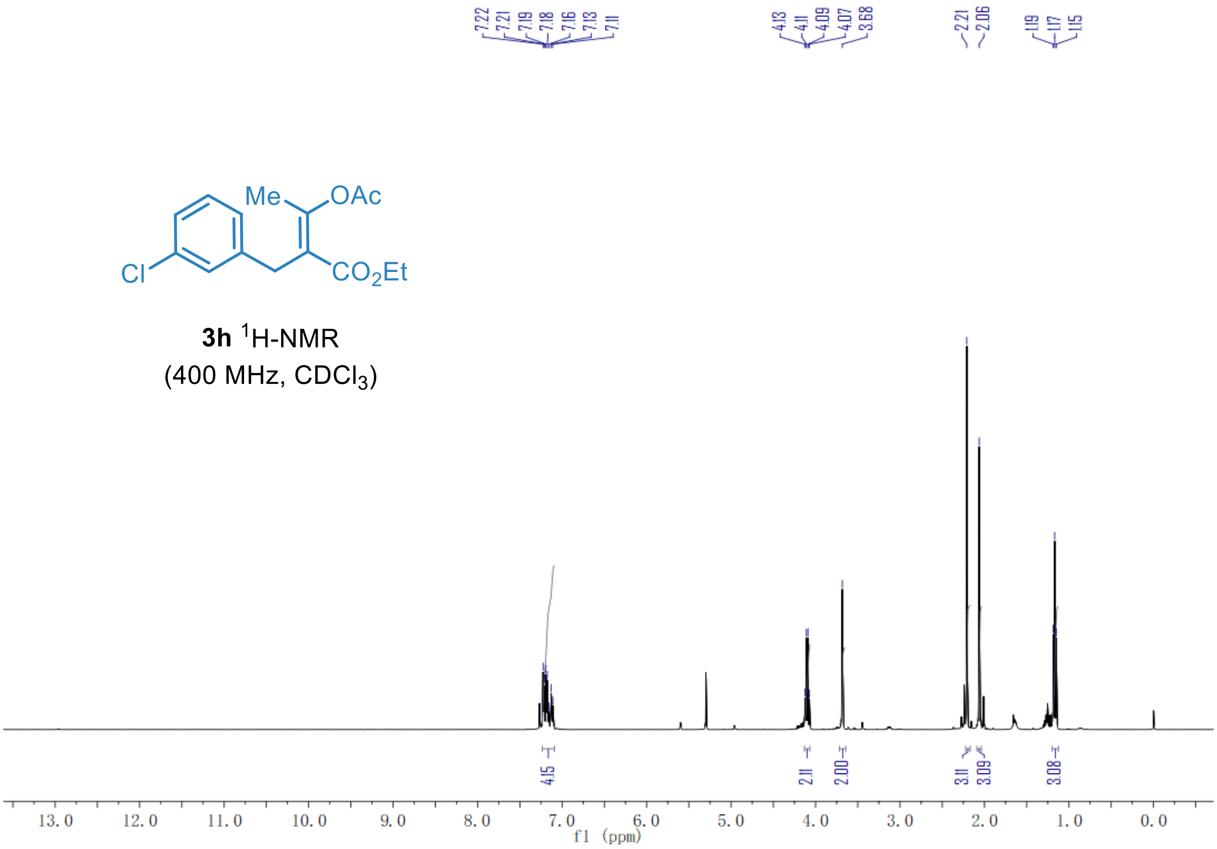

Supplementary Figure 21. **^{1}H-NMR** (400 MHz, $CDCl_3$, 298K) of **3h**

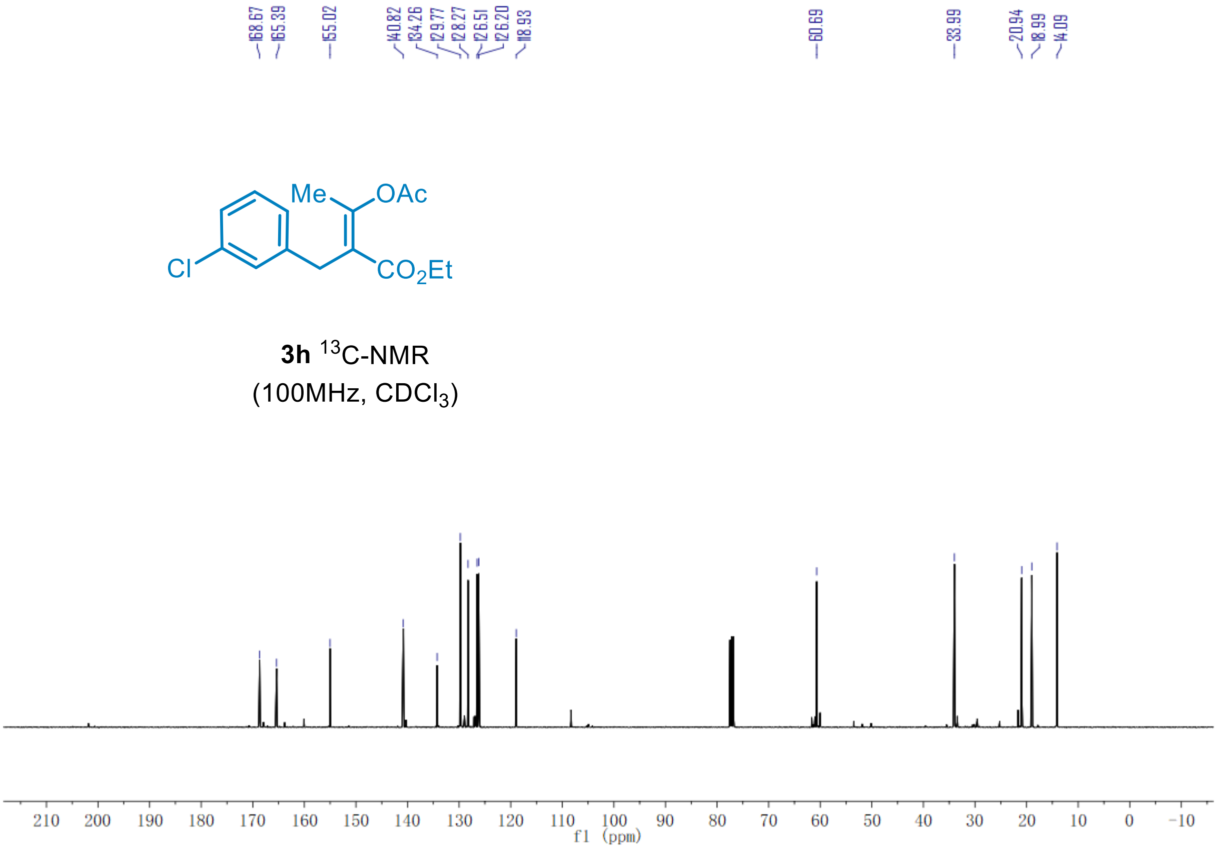

Supplementary Figure 22. **^{13}C-NMR** (100 MHz, $CDCl_3$, 298K) of **3h**

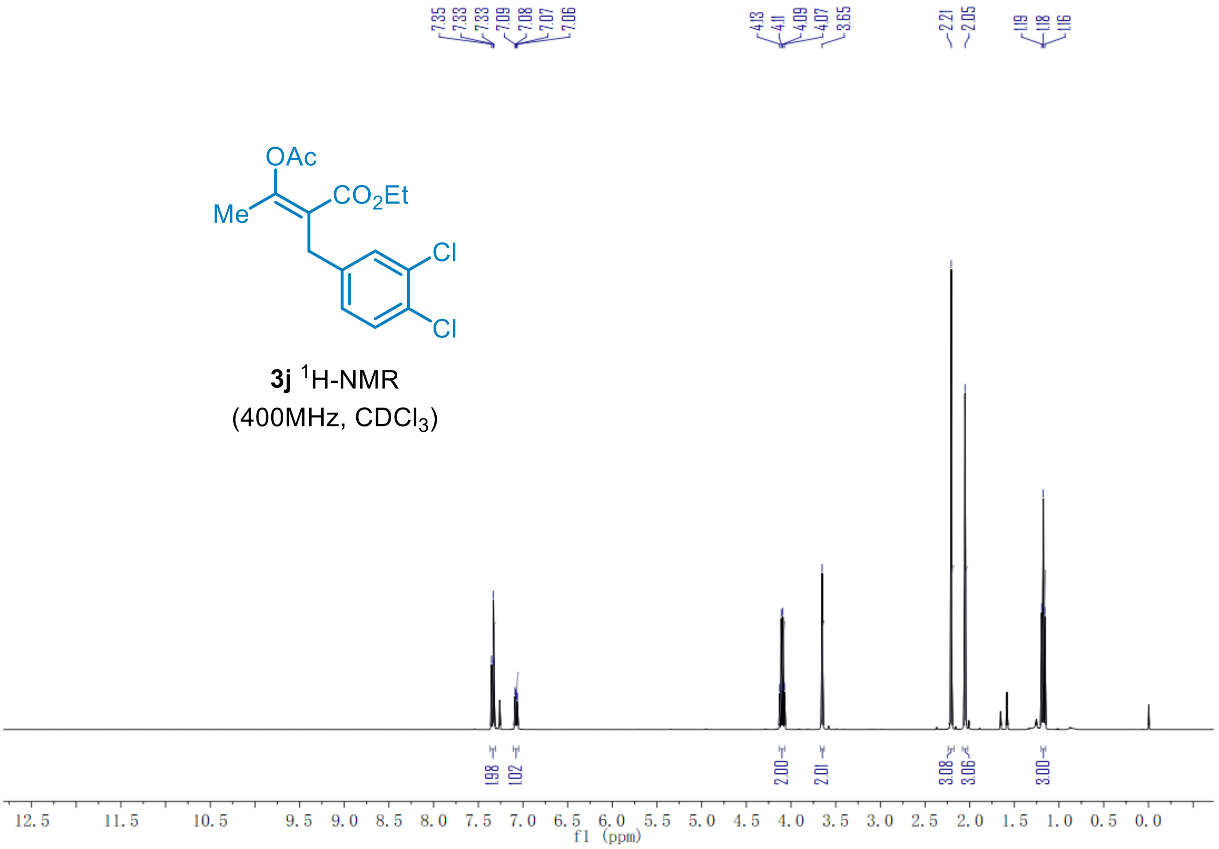

Supplementary Figure 23. **^{1}H-NMR** (400 MHz, $CDCl_3$, 298K) of **3j**

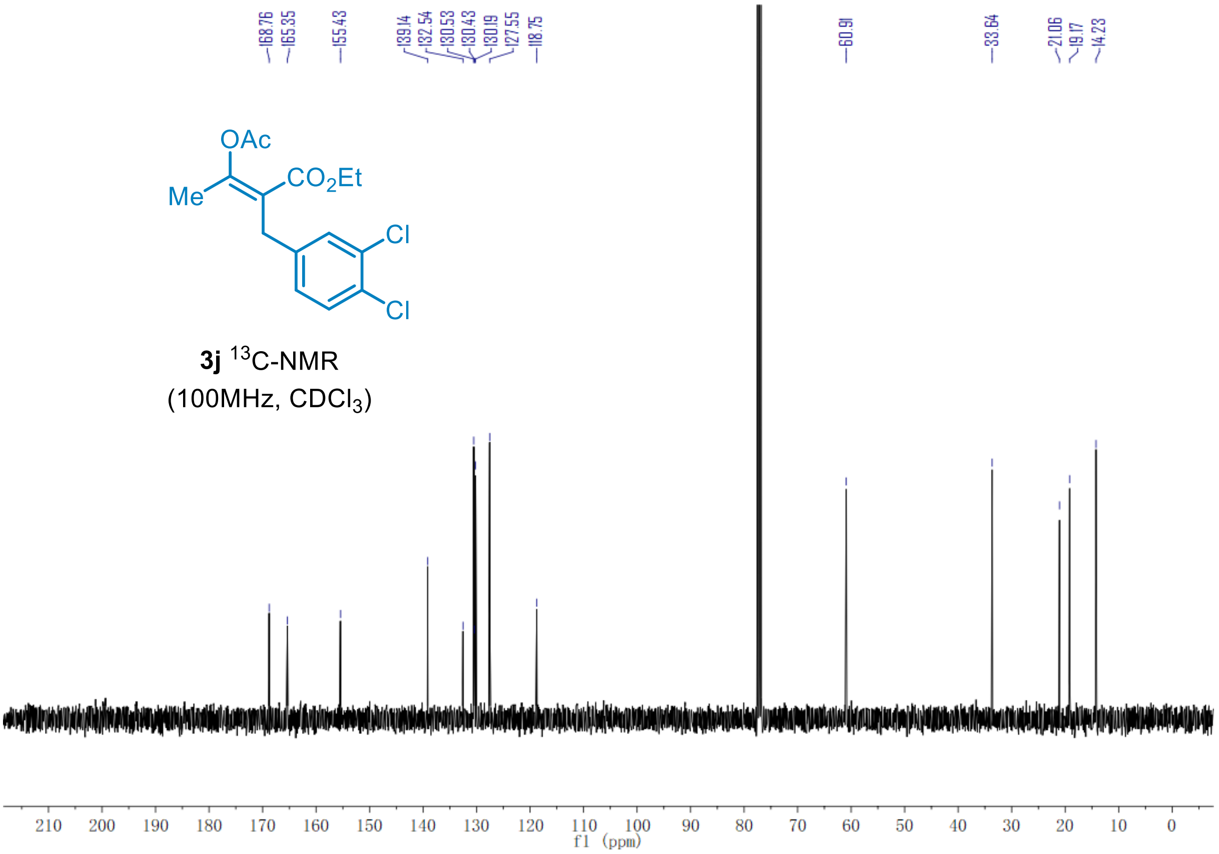

Supplementary Figure 24. **^{13}C-NMR** (100 MHz, $CDCl_3$, 298K) of **3j**

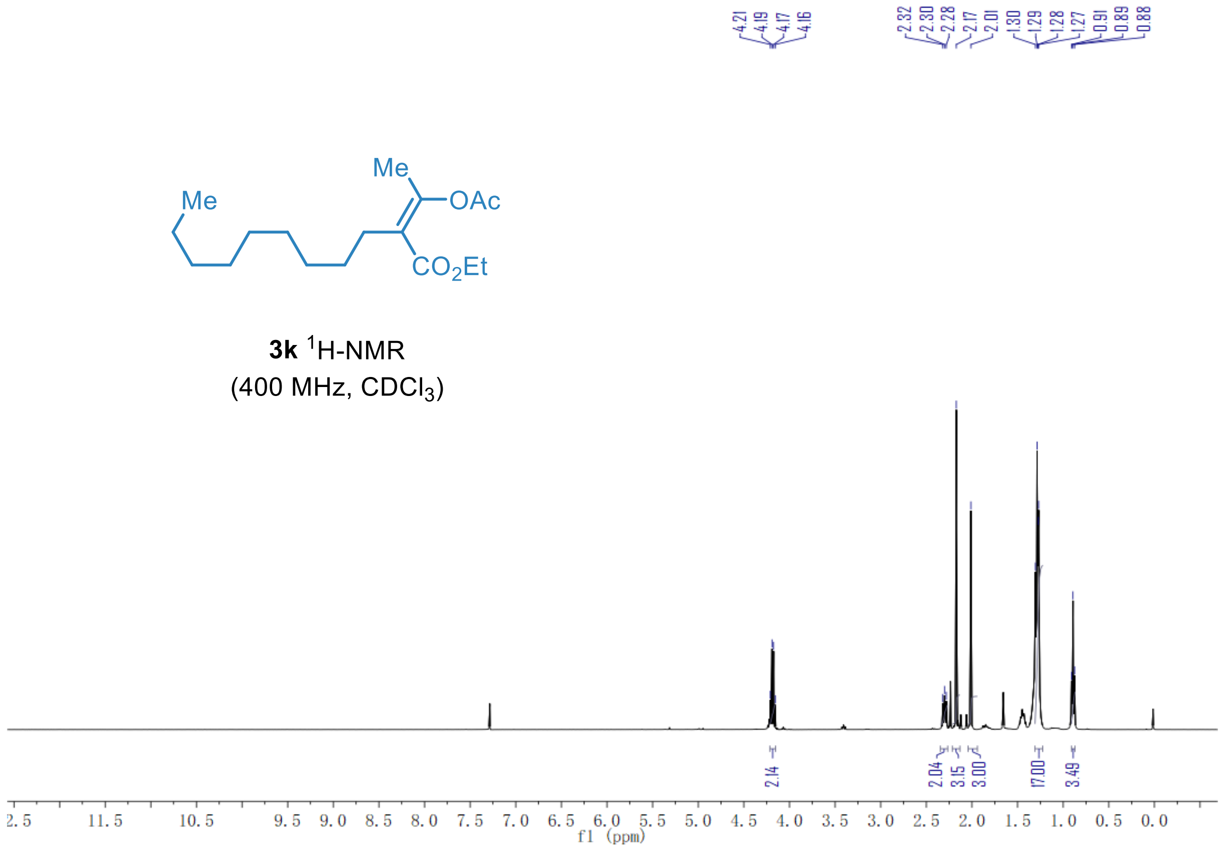

Supplementary Figure 25. **^{1}H-NMR** (400 MHz, $CDCl_3$, 298K) of **3k**

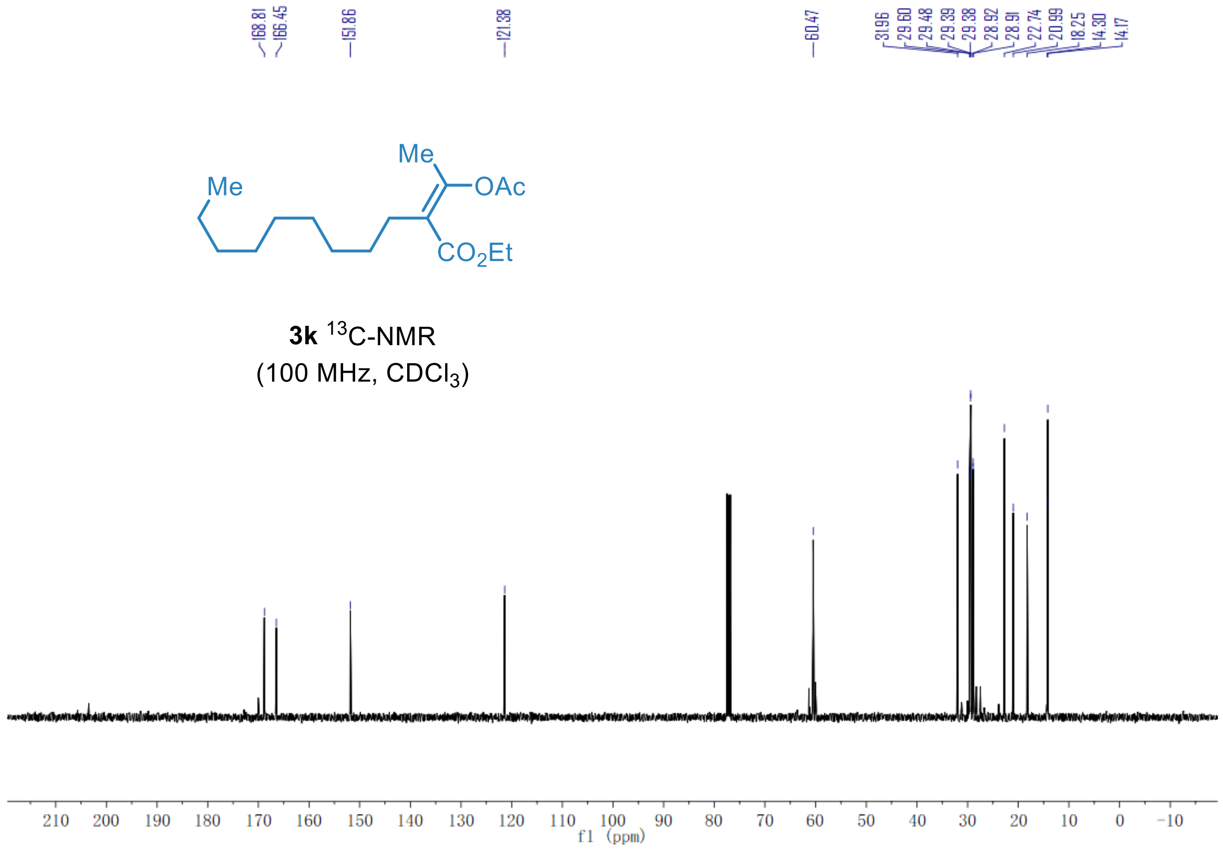

Supplementary Figure 26. **^{13}C-NMR** (100 MHz, $CDCl_3$, 298K) of **3k**

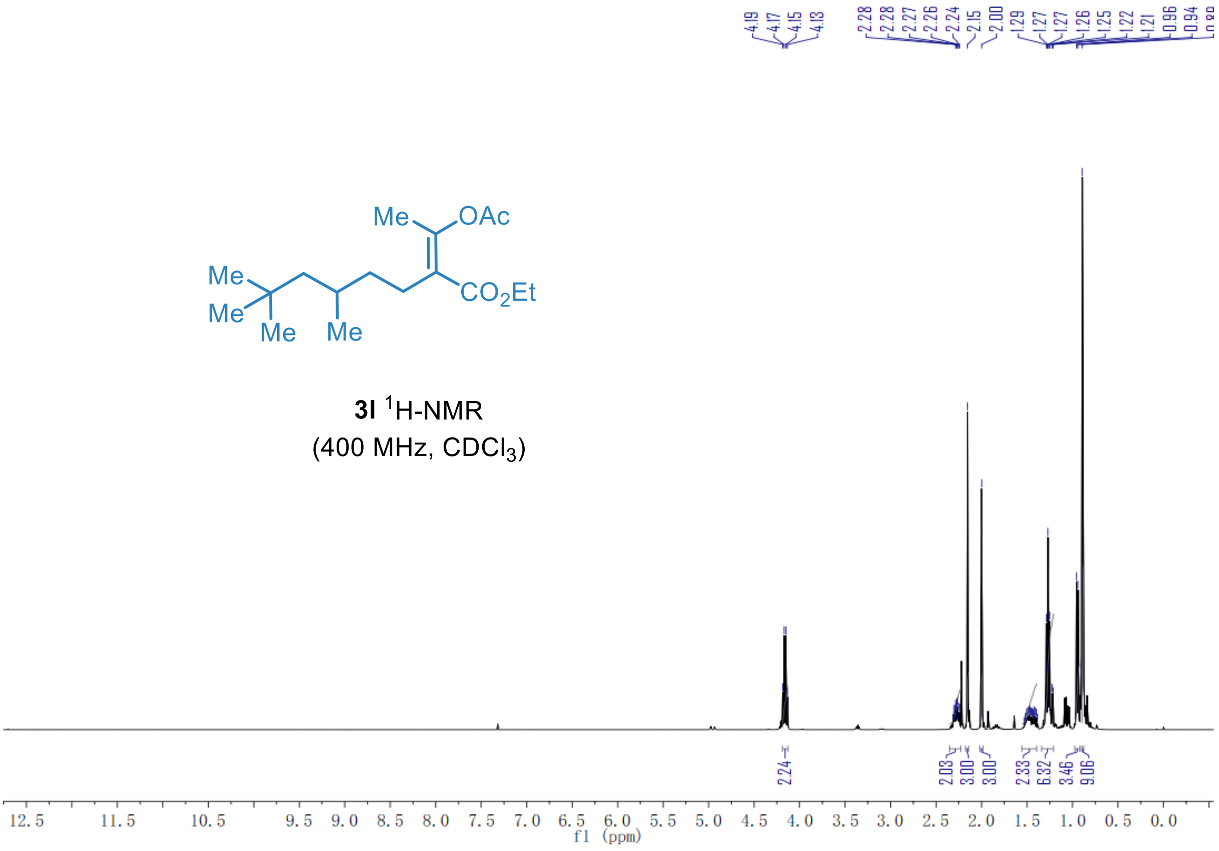

Supplementary Figure 27. **^{1}H-NMR** (400 MHz, $CDCl_3$, 298K) of **3l**

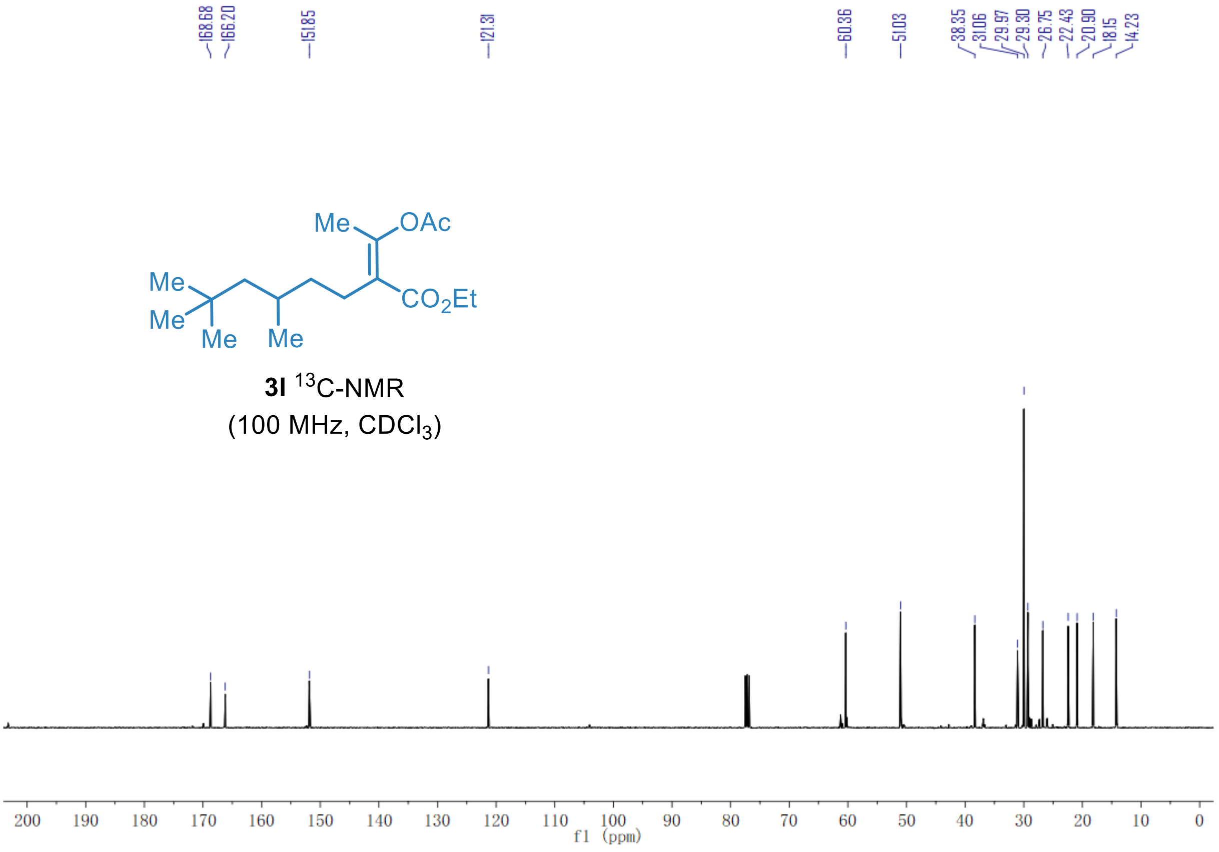

Supplementary Figure 28. **^{13}C-NMR** (100 MHz, $CDCl_3$, 298K) of **3l**

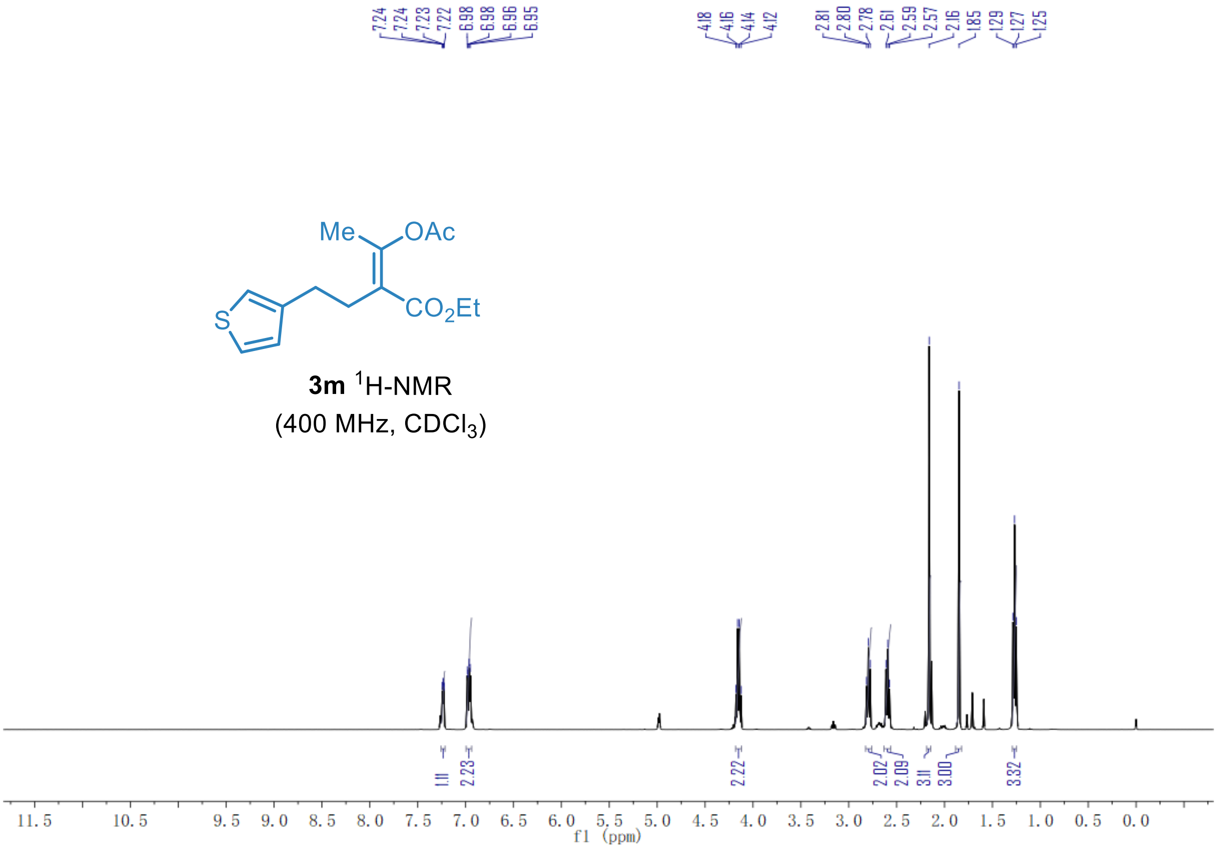

Supplementary Figure 29. **^{1}H-NMR** (400 MHz, $CDCl_3$, 298K) of **3m**

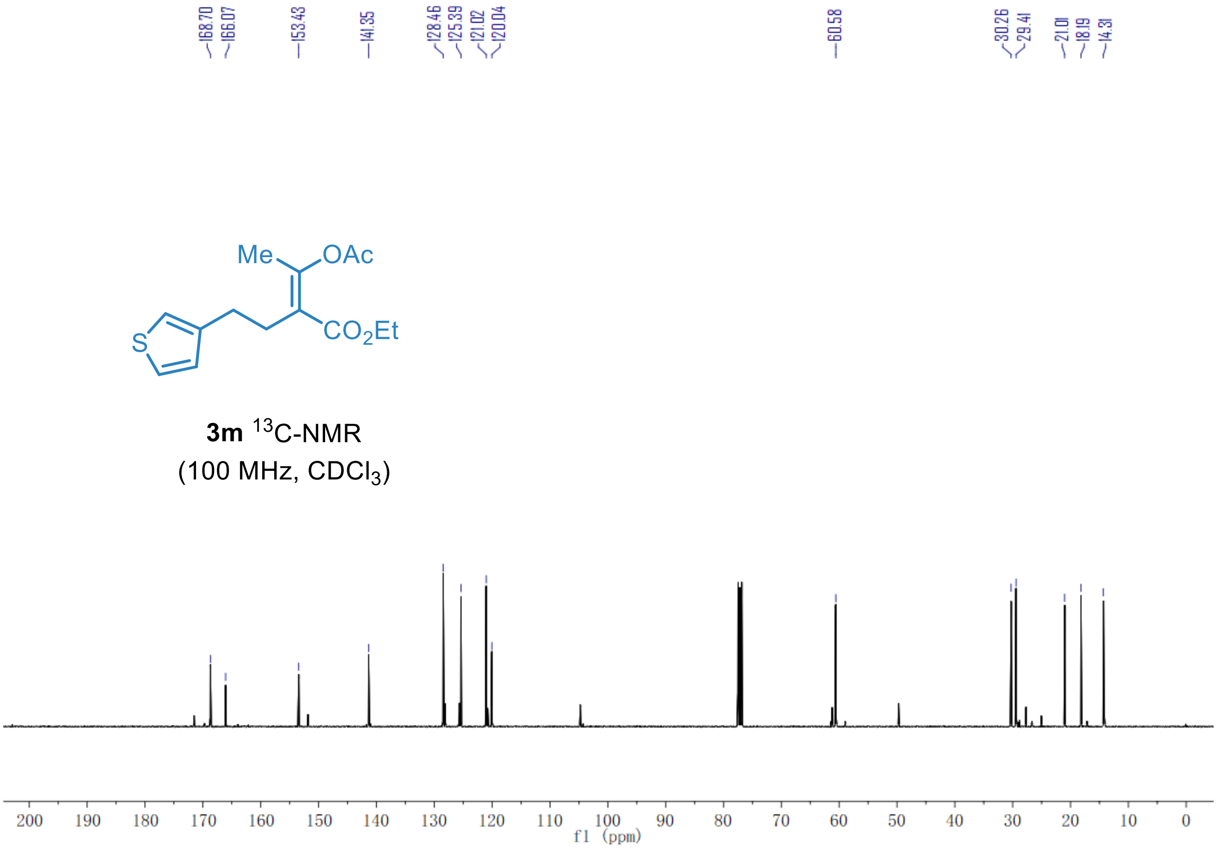

Supplementary Figure 30. **^{13}C-NMR** (100 MHz, $CDCl_3$, 298K) of **3m**

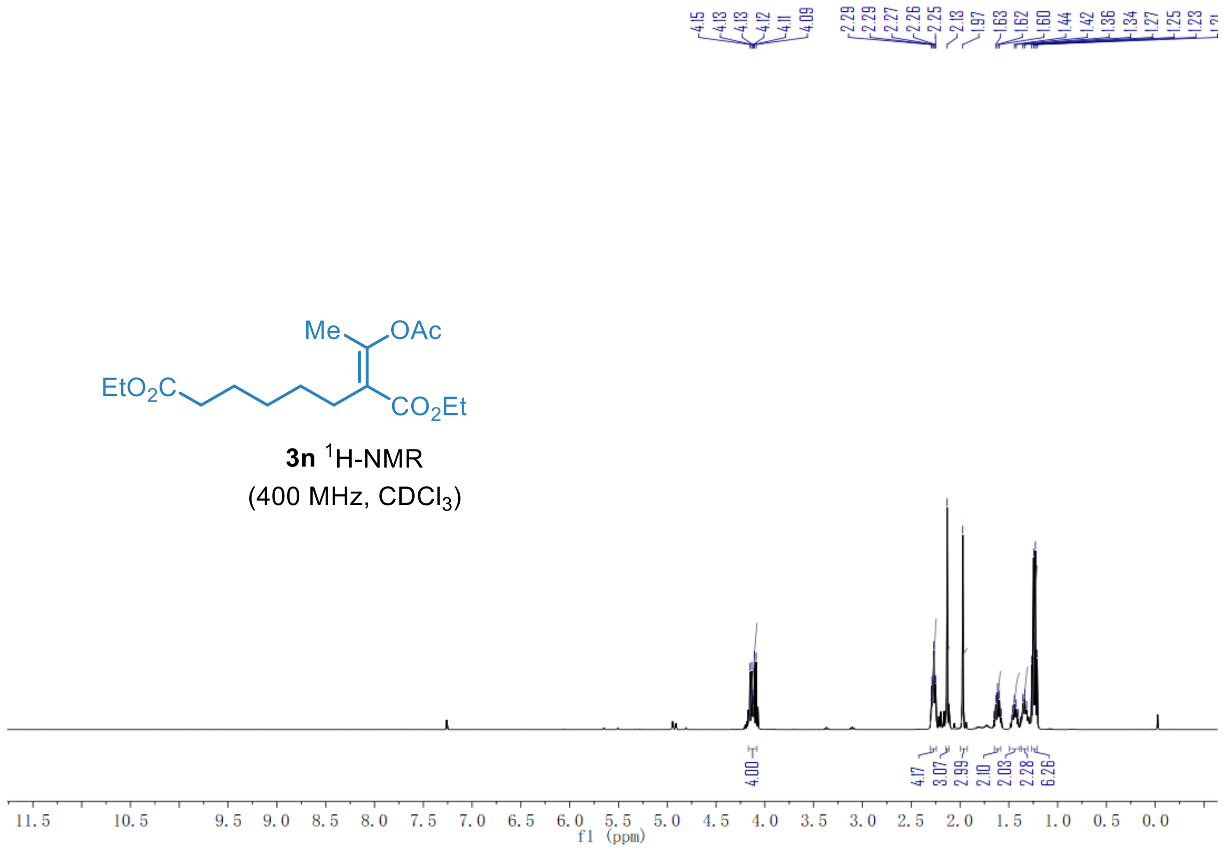

Supplementary Figure 31. **^{1}H-NMR** (400 MHz, $CDCl_3$, 298K) of **3n**

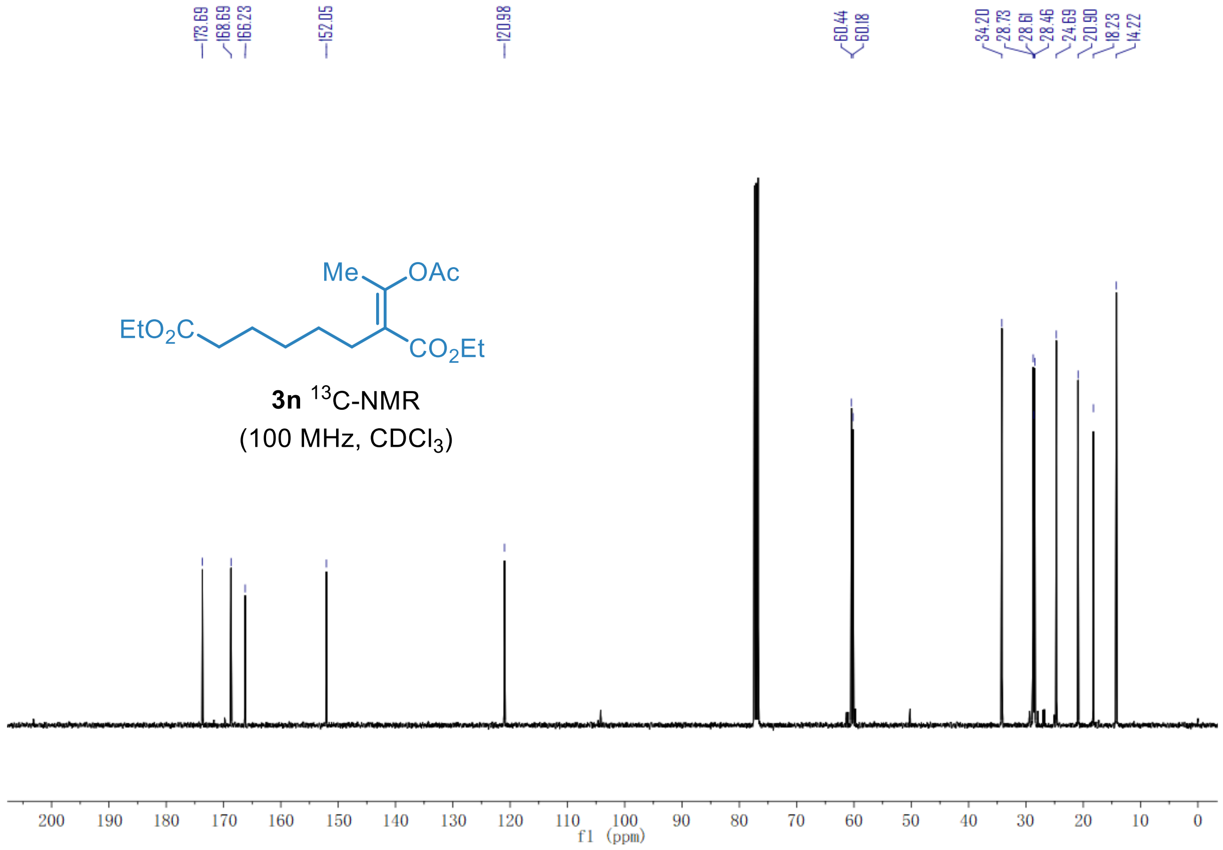

Supplementary Figure 32. **^{13}C-NMR** (100 MHz, $CDCl_3$, 298K) of **3n**

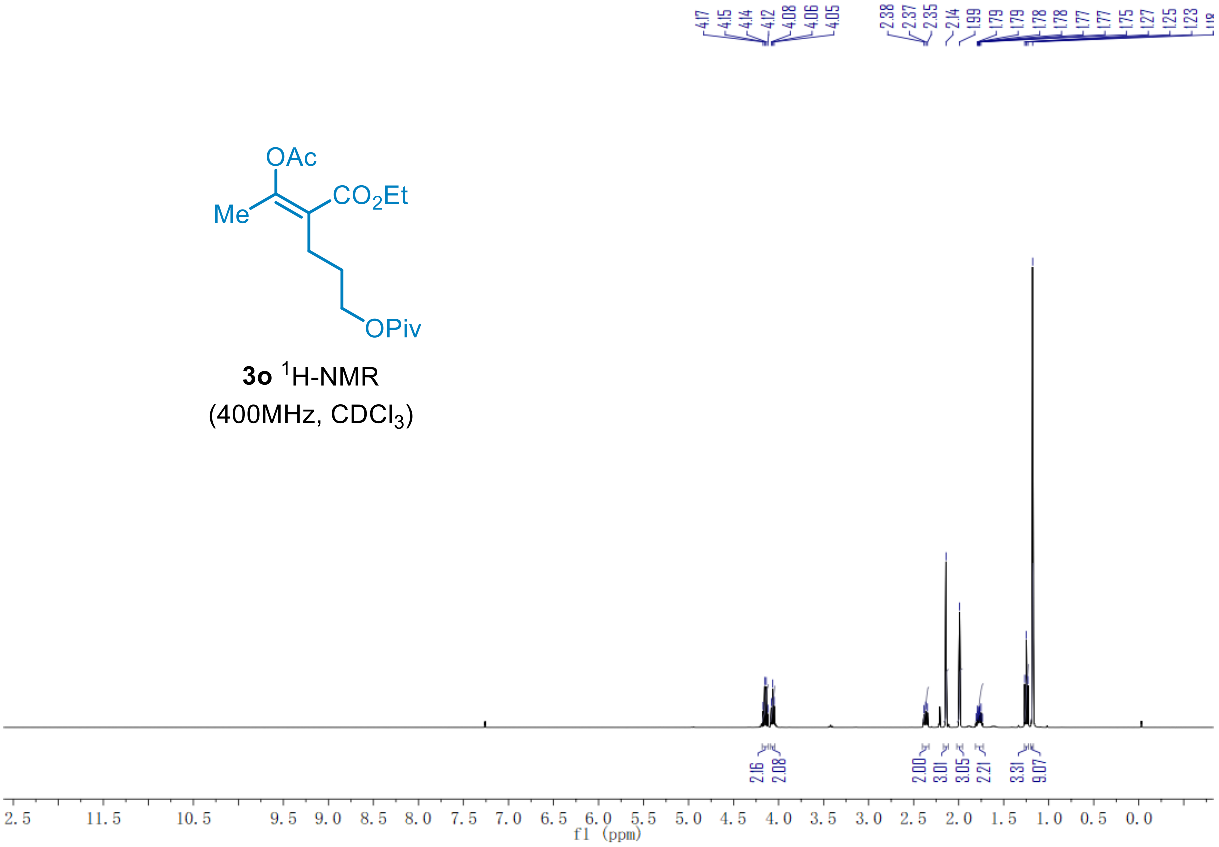

Supplementary Figure 33. **^{1}H-NMR** (400 MHz, $CDCl_3$, 298K) of **3o**

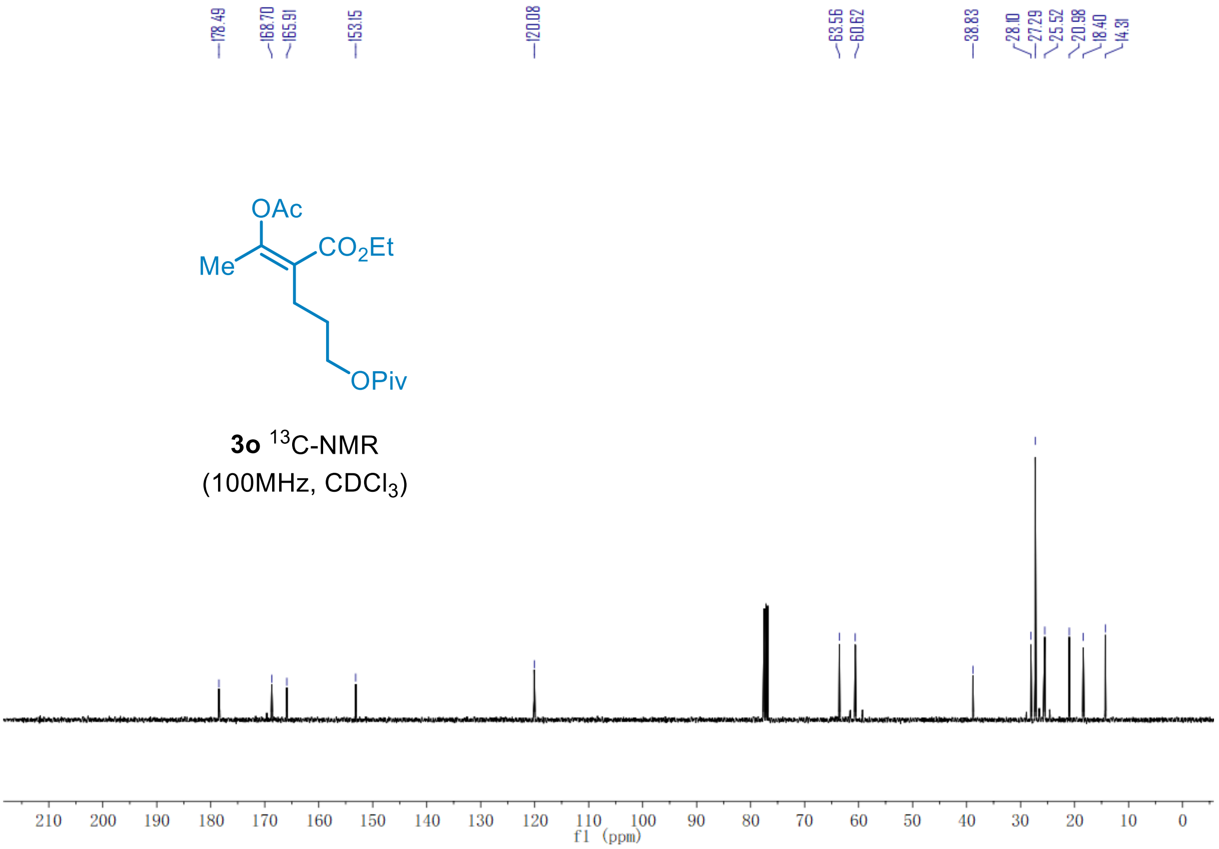

Supplementary Figure 34. **^{13}C-NMR** (100 MHz, $CDCl_3$, 298K) of **3o**

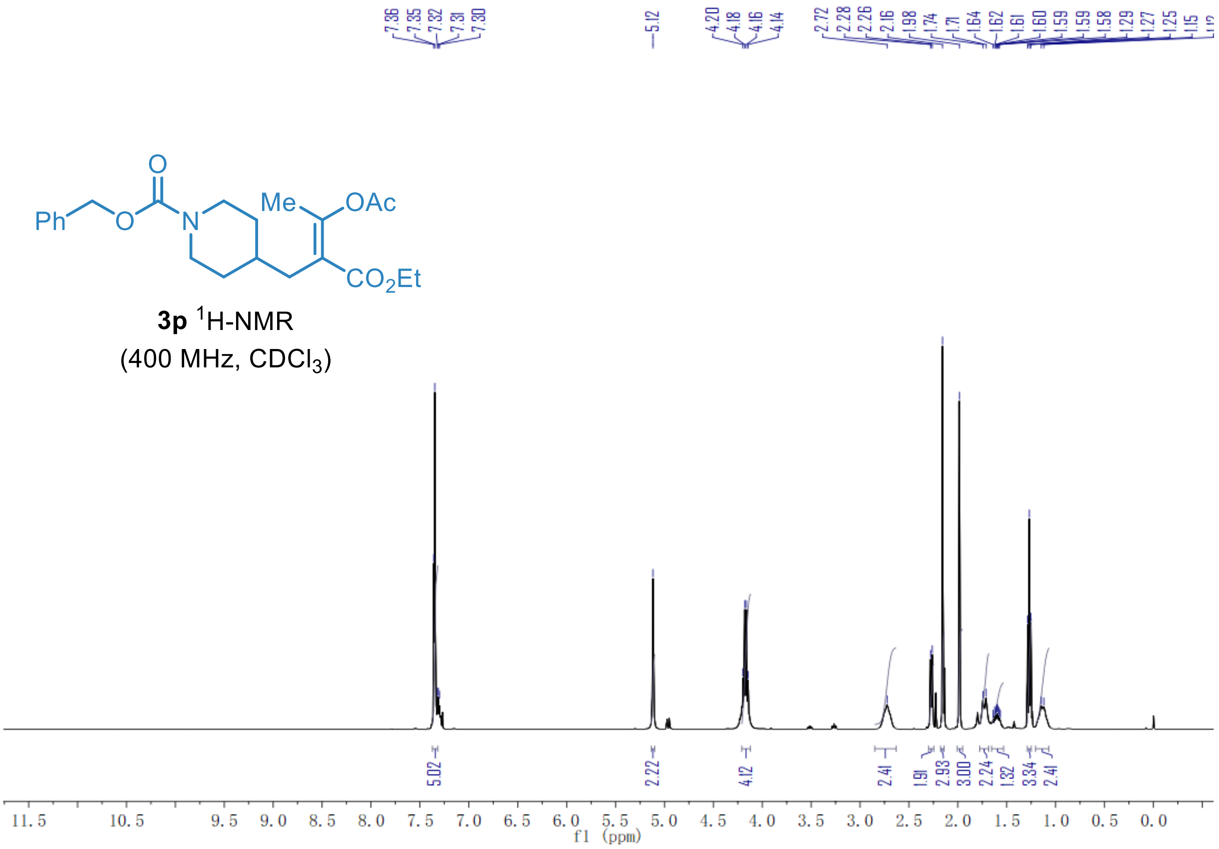

Supplementary Figure 35. **^{1}H-NMR** (400 MHz, $CDCl_3$, 298K) of **3p**

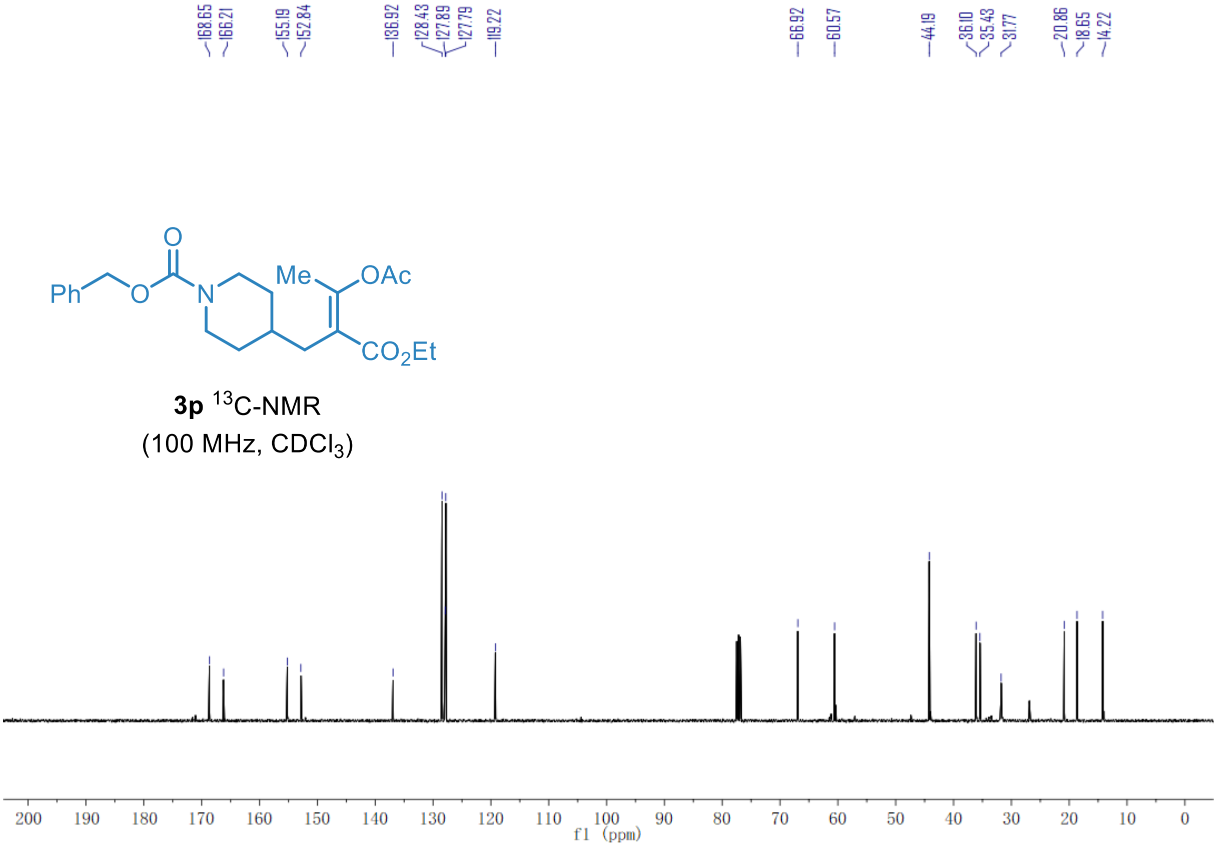

Supplementary Figure 36. **^{13}C-NMR** (100 MHz, $CDCl_3$, 298K) of **3p**

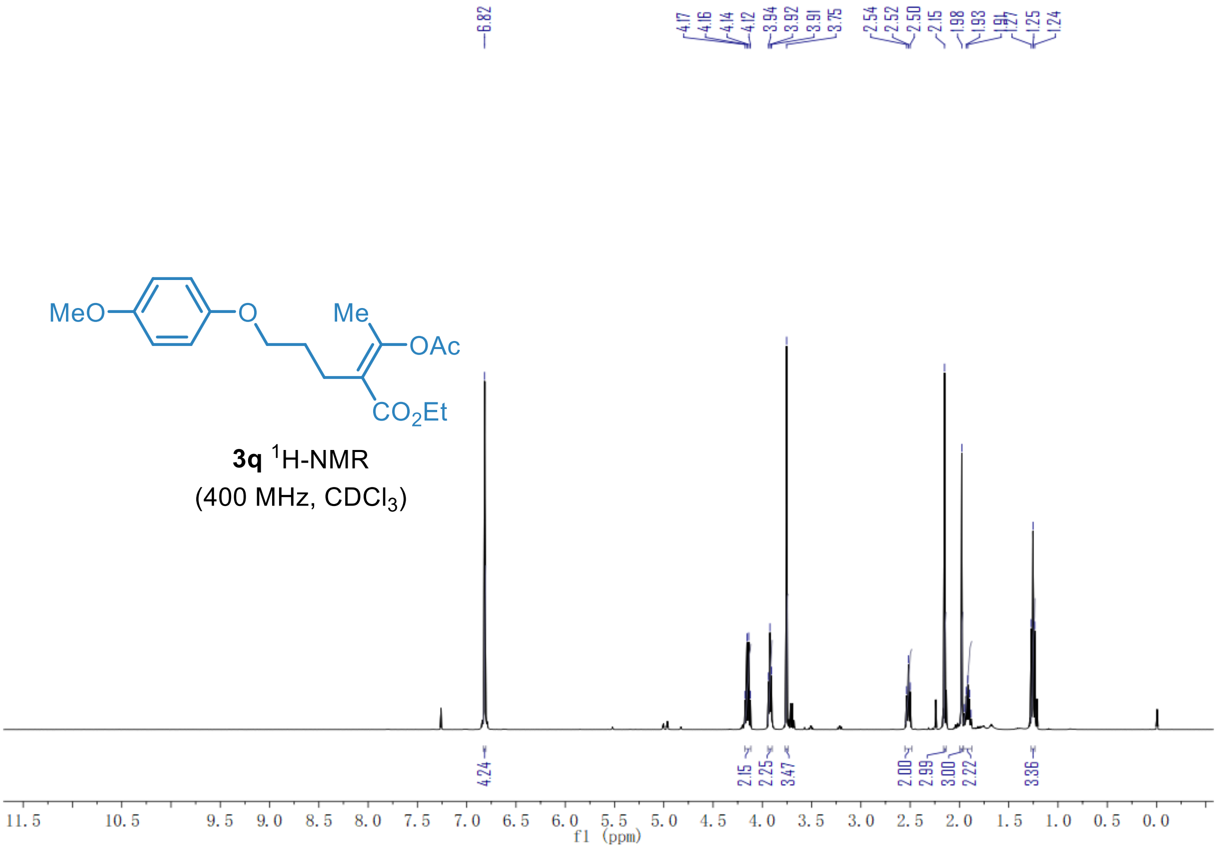

Supplementary Figure 37. **^{1}H-NMR** (400 MHz, $CDCl_3$, 298K) of **3q**

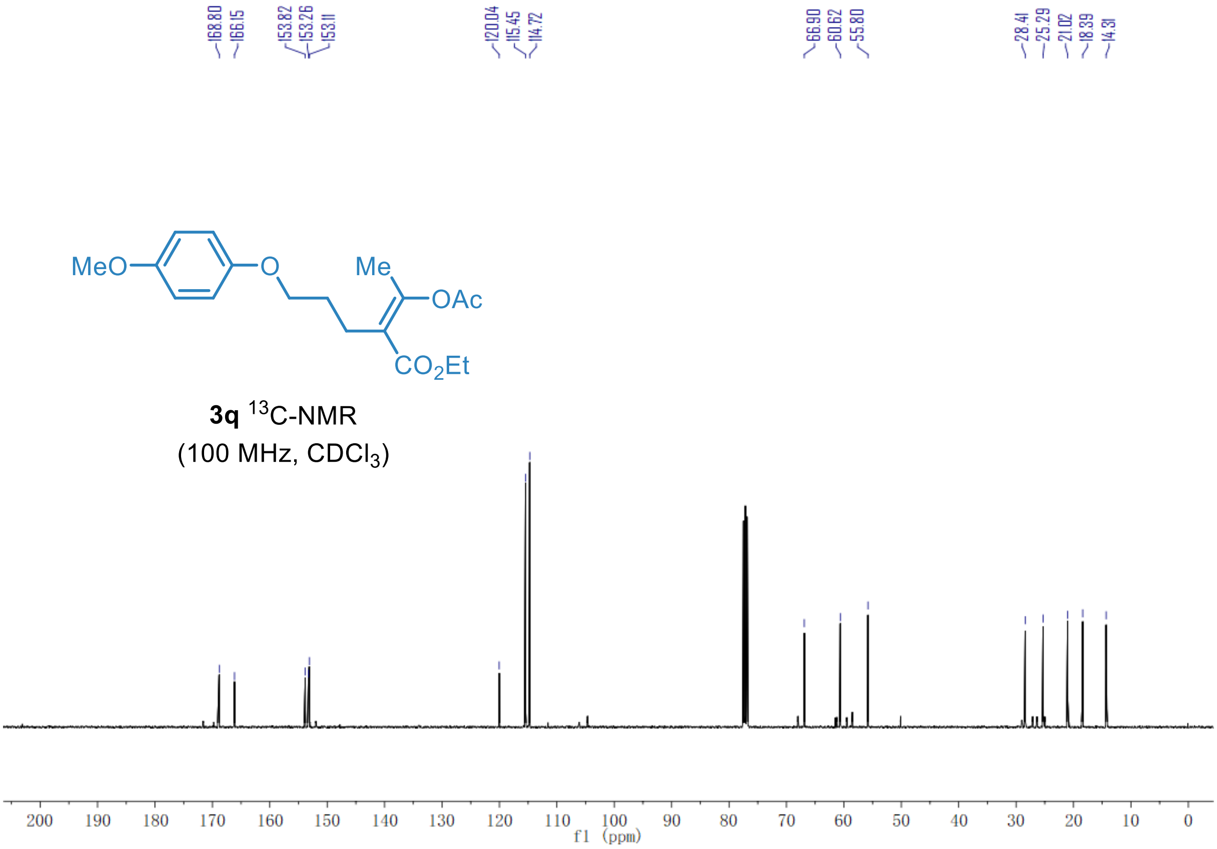

Supplementary Figure 38. **^{13}C-NMR** (100 MHz, $CDCl_3$, 298K) of **3q**

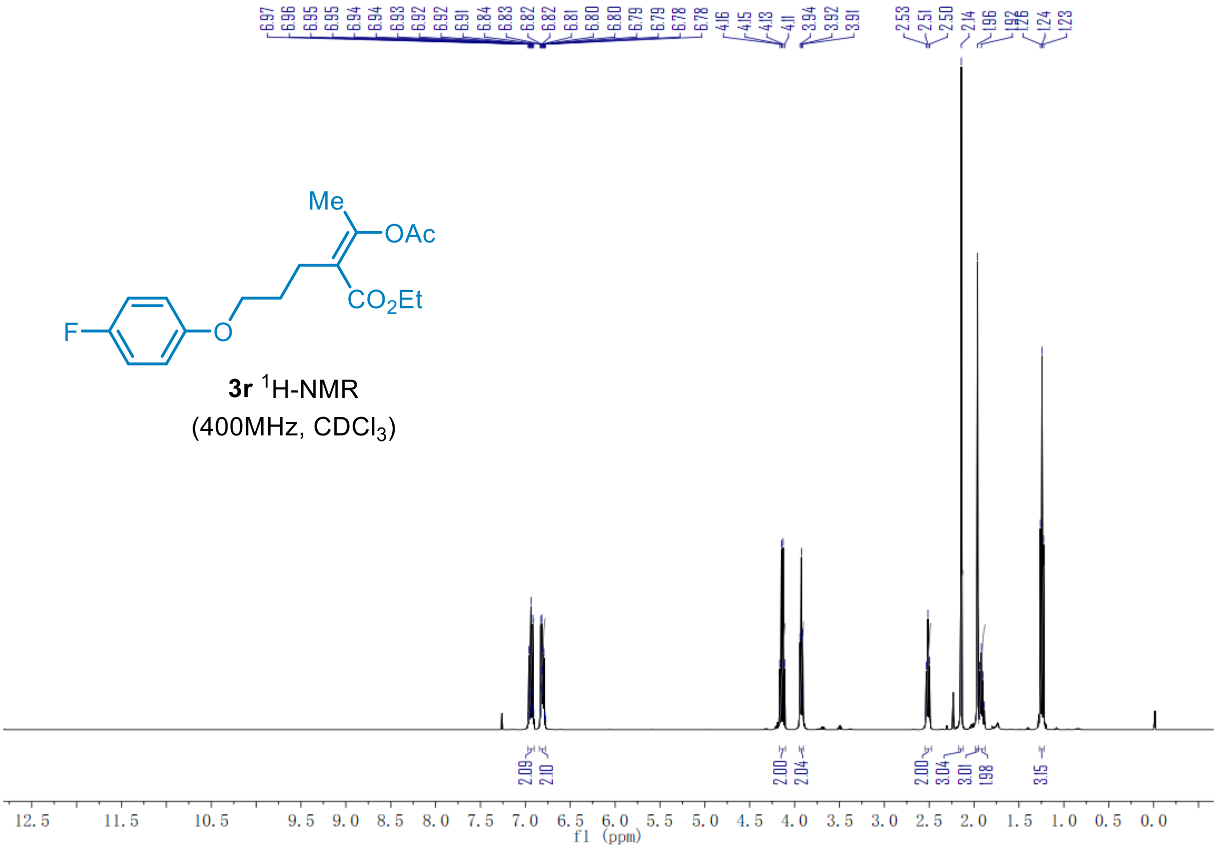

Supplementary Figure 39. **^{1}H-NMR** (400 MHz, $CDCl_3$, 298K) of **3r**

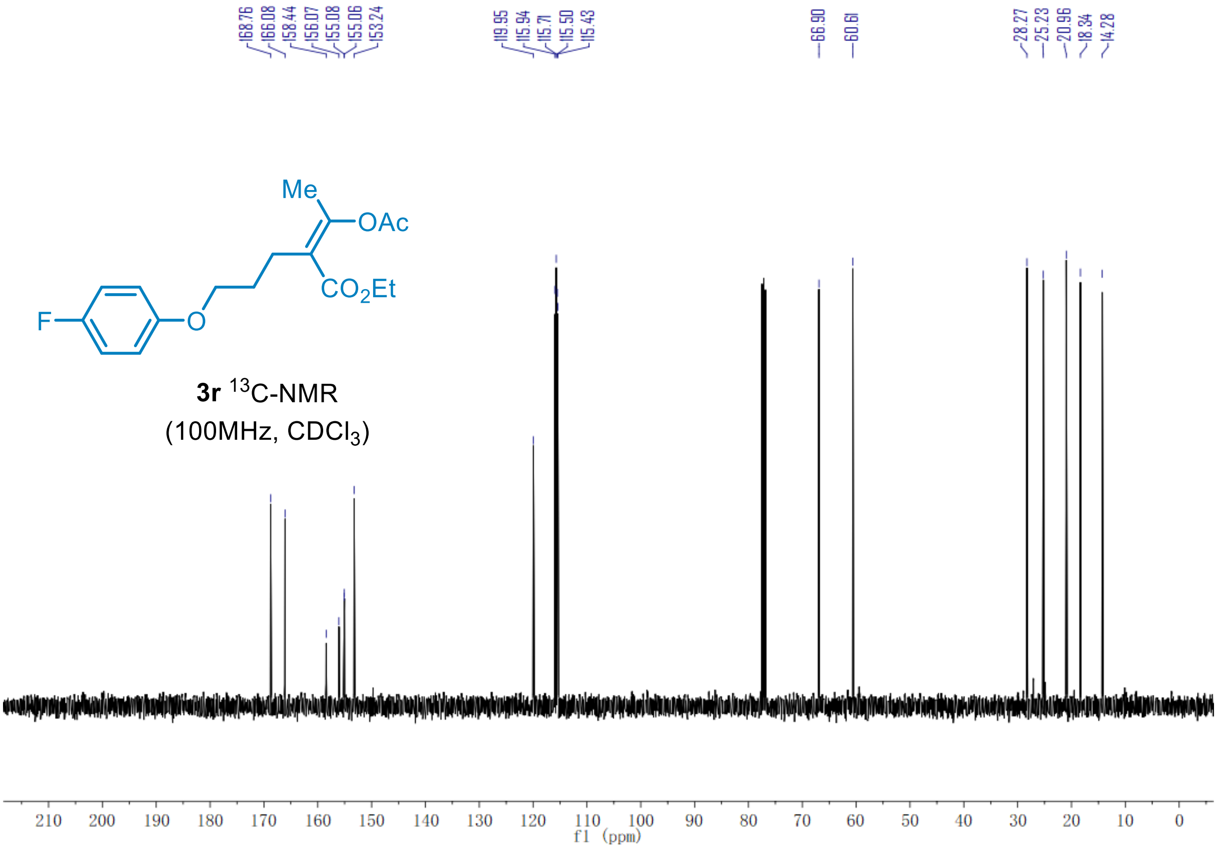

Supplementary Figure 40. **^{13}C-NMR** (100 MHz, $CDCl_3$, 298K) of **3r**

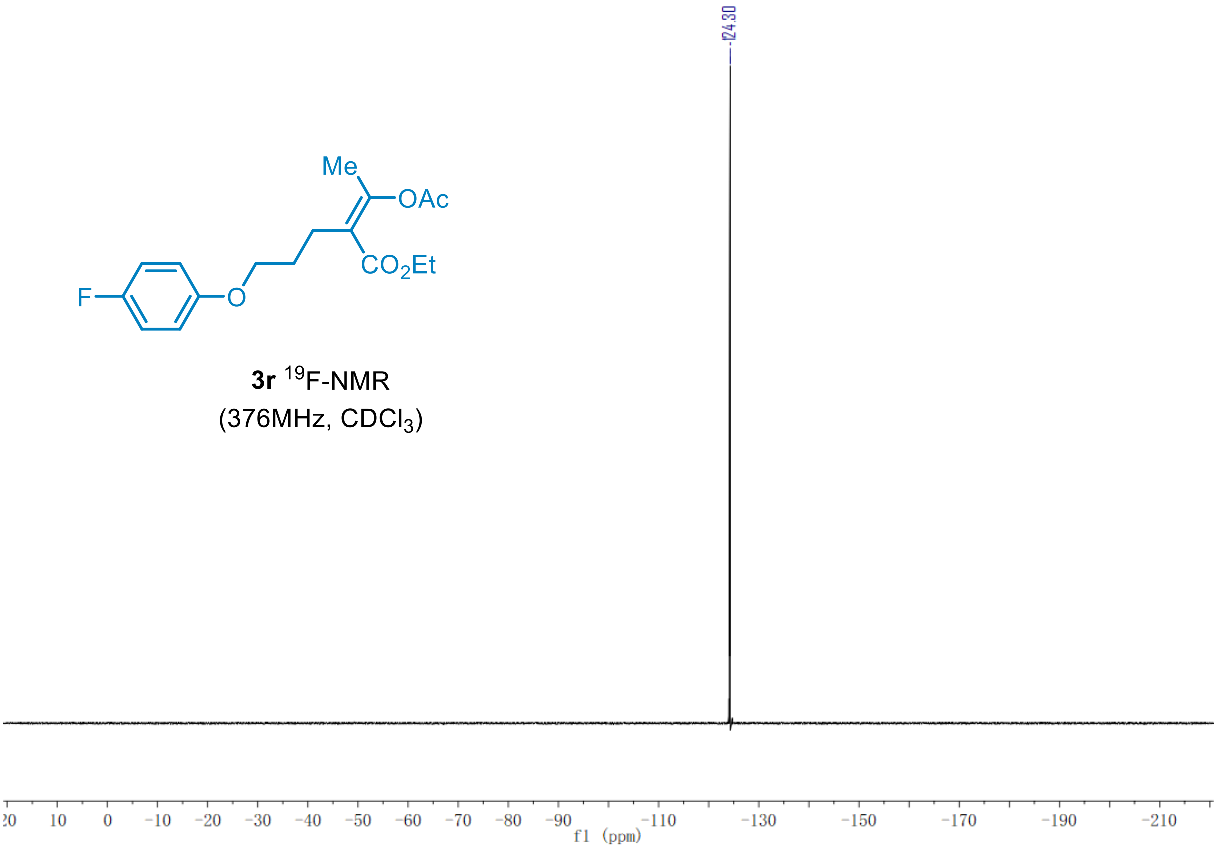

Supplementary Figure 41. **^{19}F-NMR** (376 MHz, $CDCl_3$, 298K) of **3r**

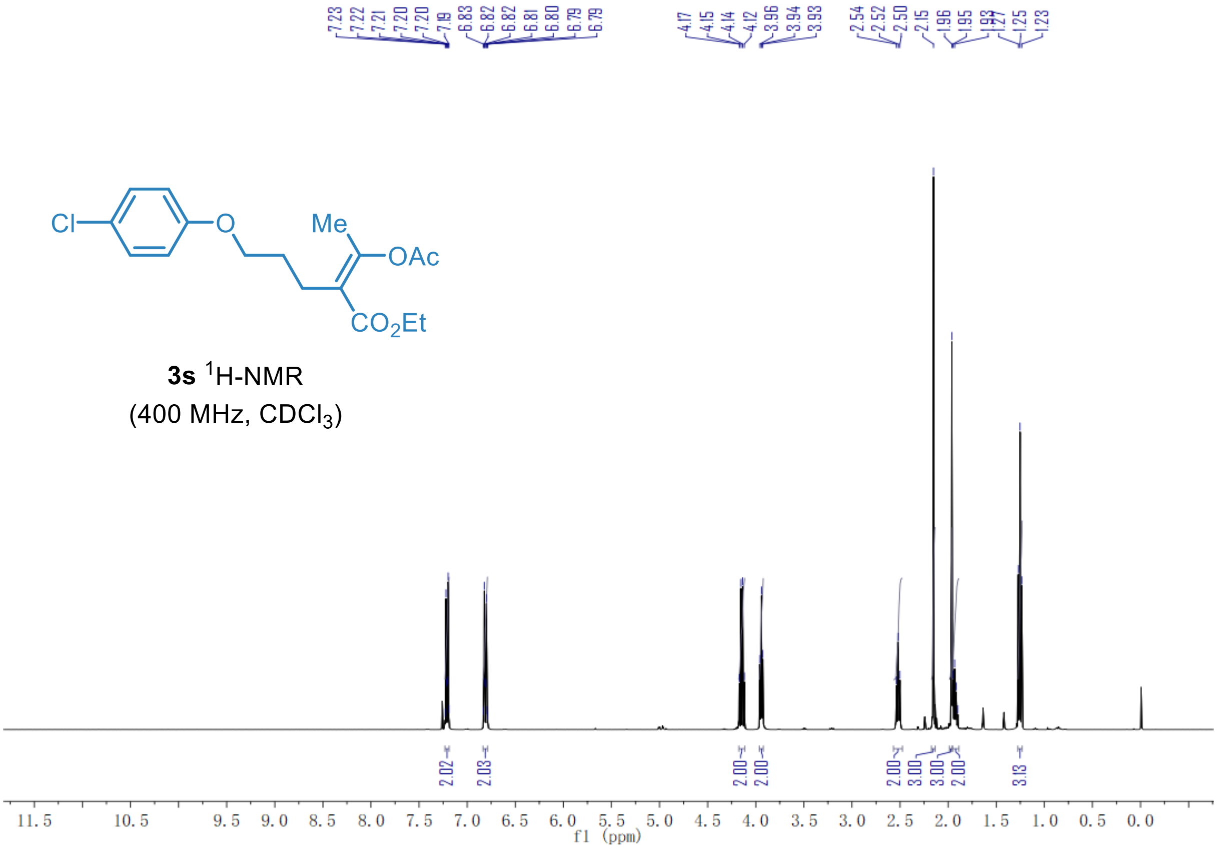

Supplementary Figure 42. **^{1}H-NMR** (400 MHz, $CDCl_3$, 298K) of **3s**

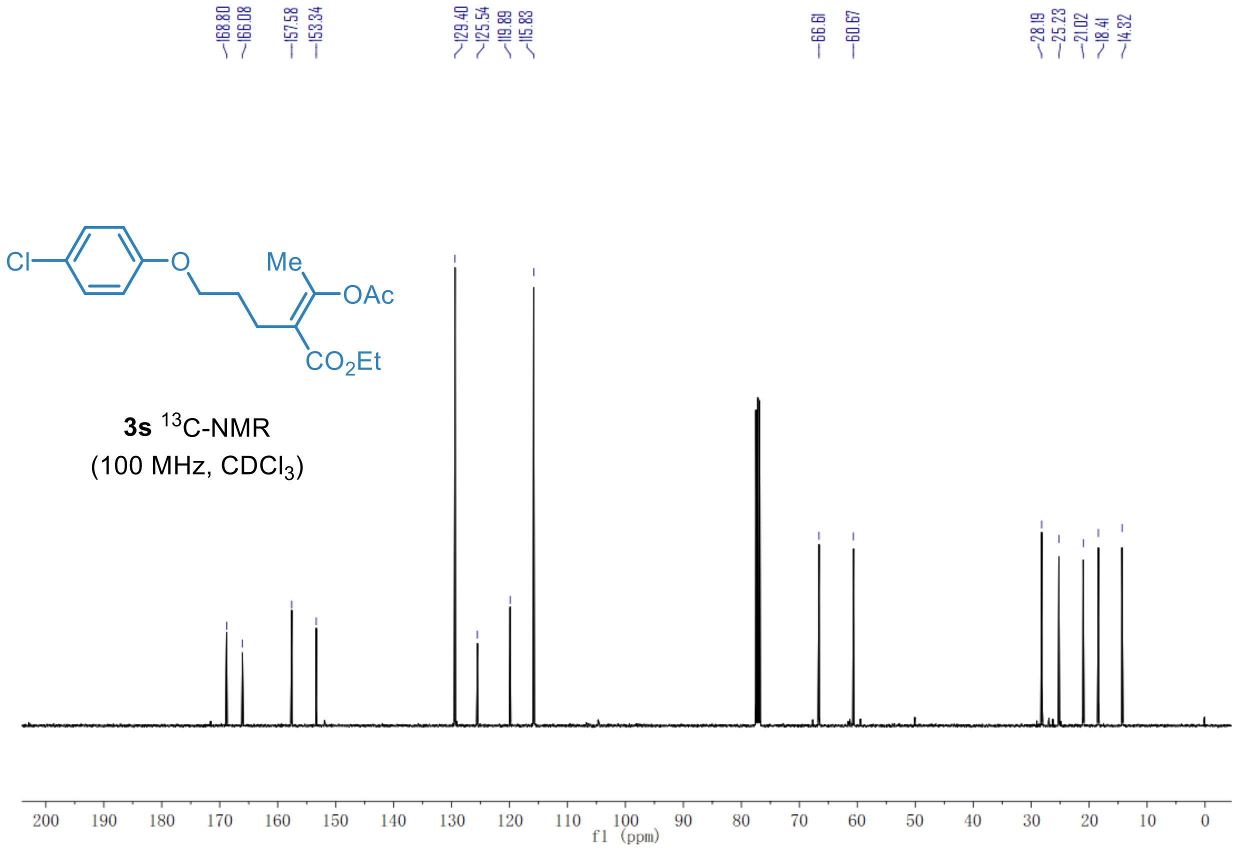

Supplementary Figure 43. **^{13}C-NMR** (100 MHz, $CDCl_3$, 298K) of **3s**

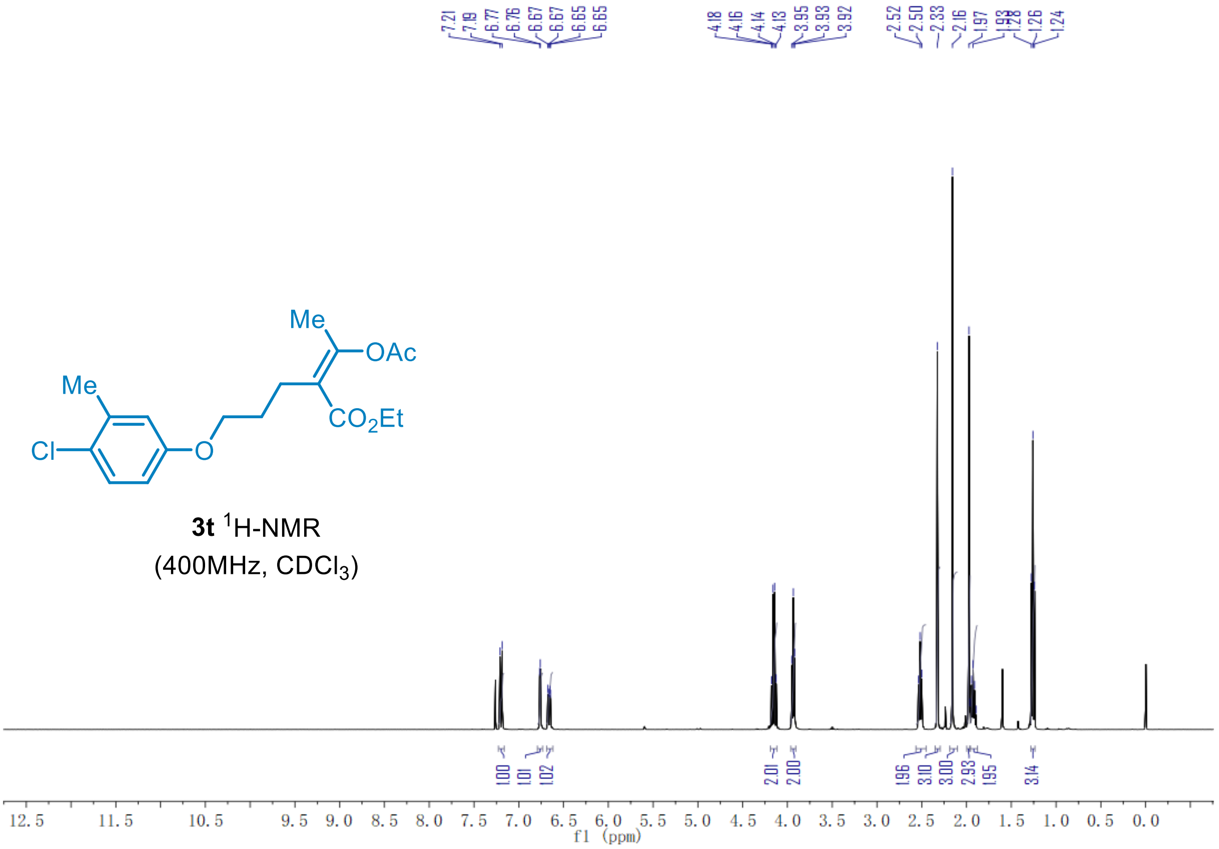

Supplementary Figure 44. **^{1}H-NMR** (400 MHz, $CDCl_3$, 298K) of **3t**

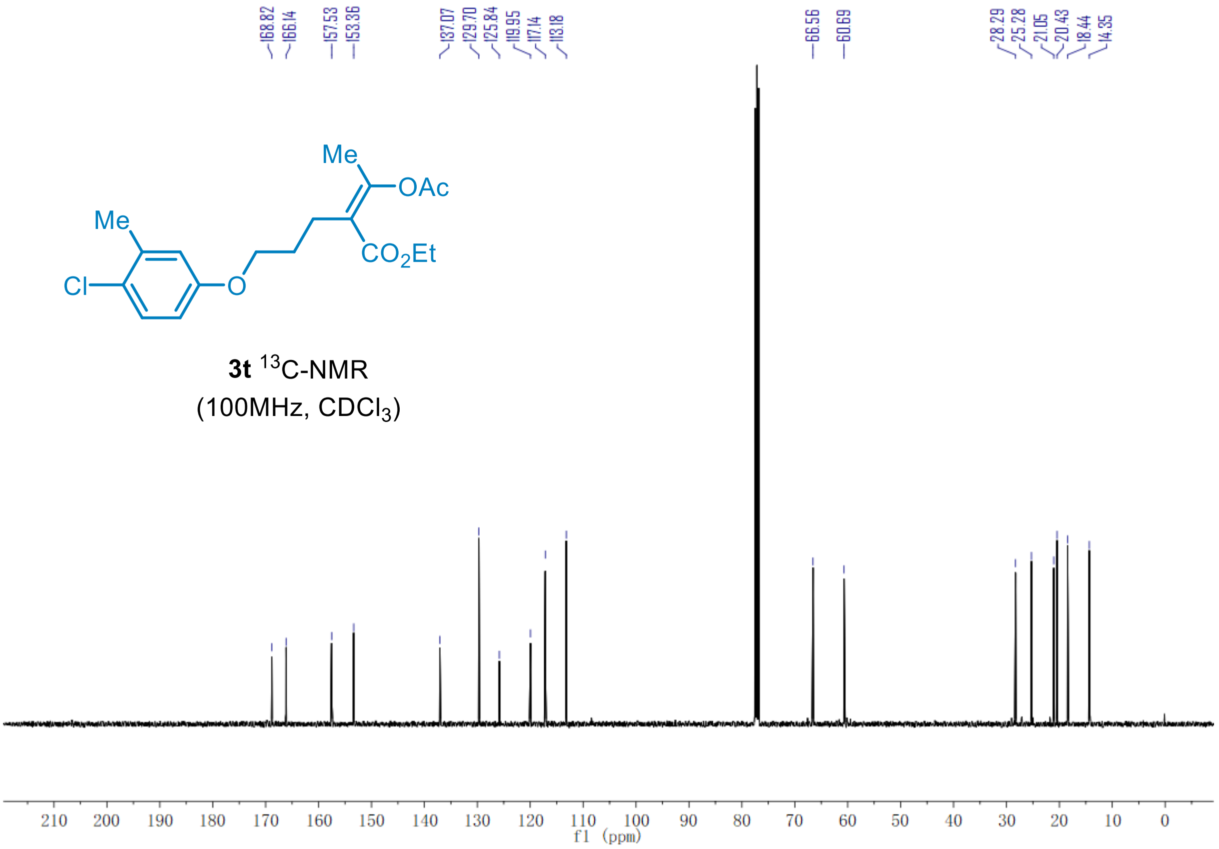

Supplementary Figure 45. **^{13}C-NMR** (100 MHz, CDCl$_3$, 298K) of **3t**

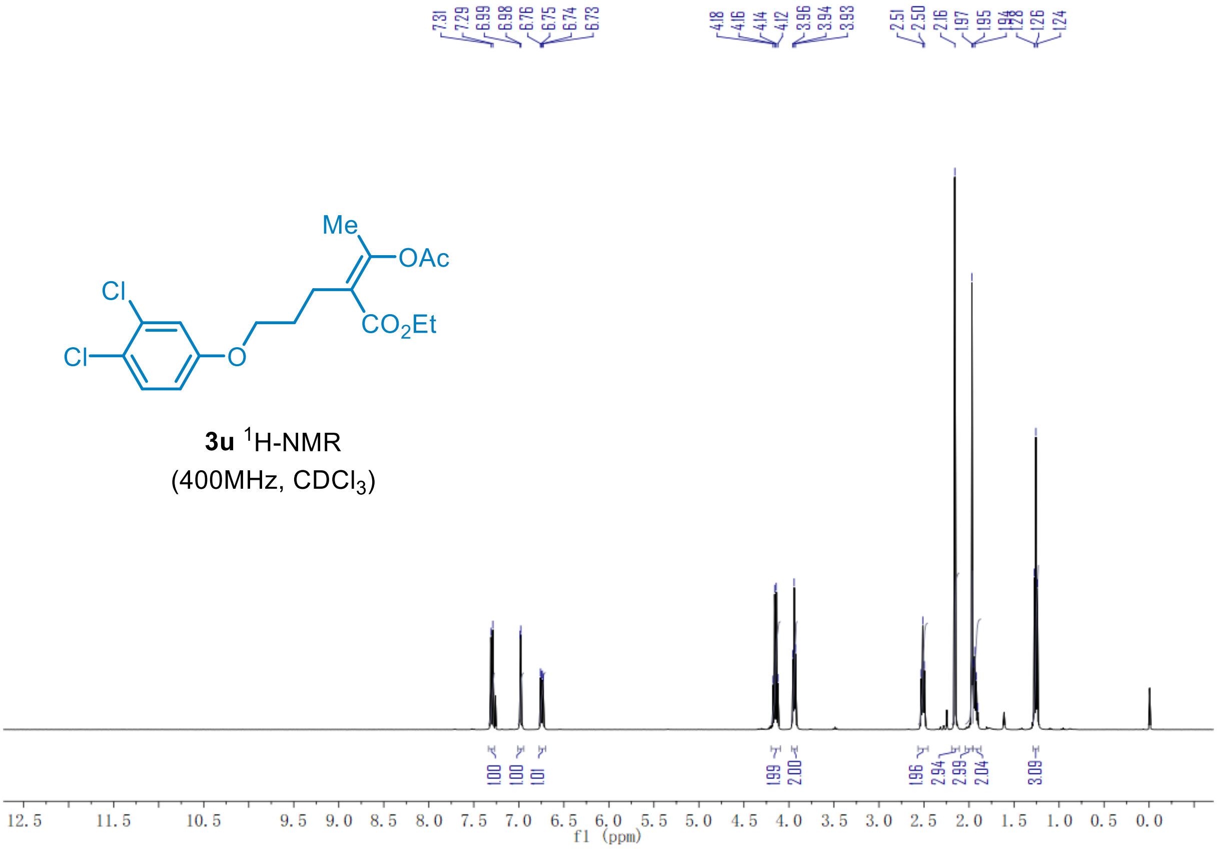

Supplementary Figure 46. **^{1}H-NMR** (400 MHz, CDCl$_3$, 298K) of **3u**

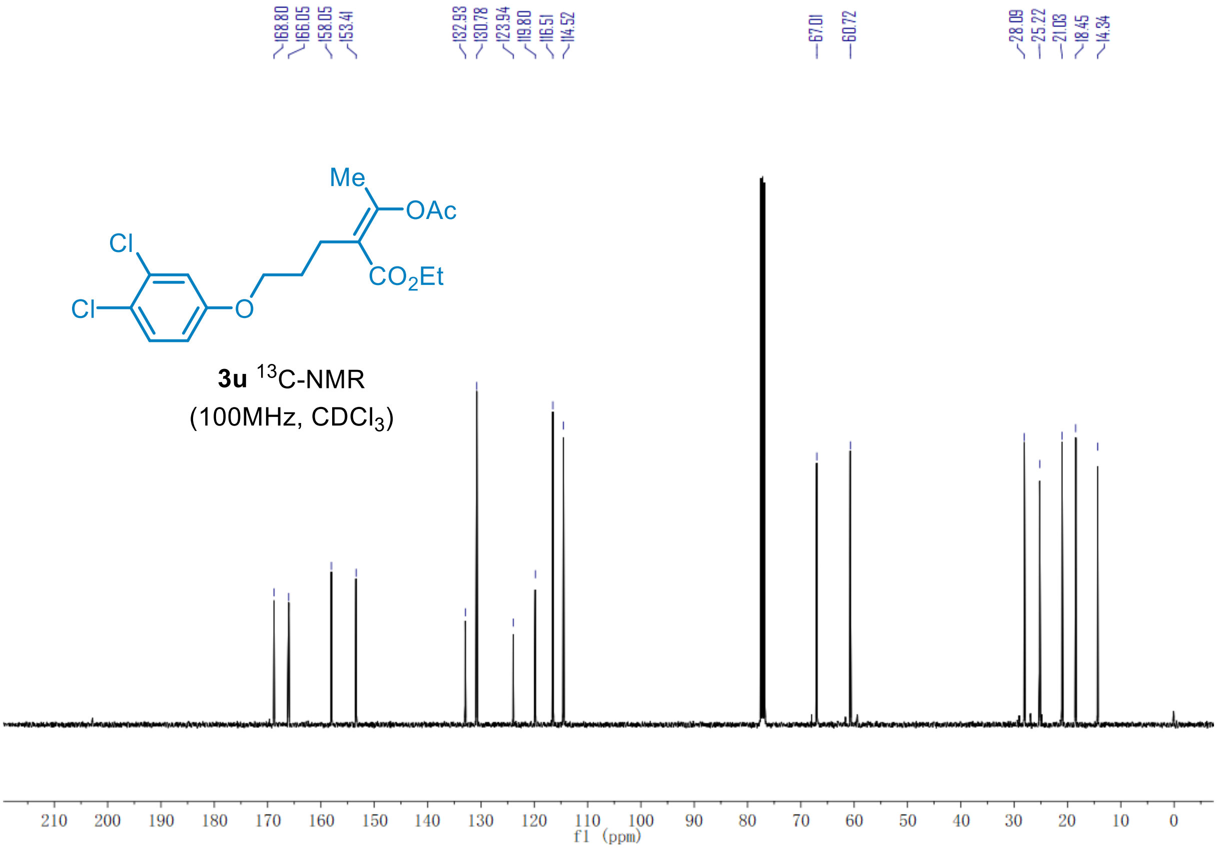

Supplementary Figure 47. **^{13}C-NMR** (100 MHz, $CDCl_3$, 298K) of **3u**

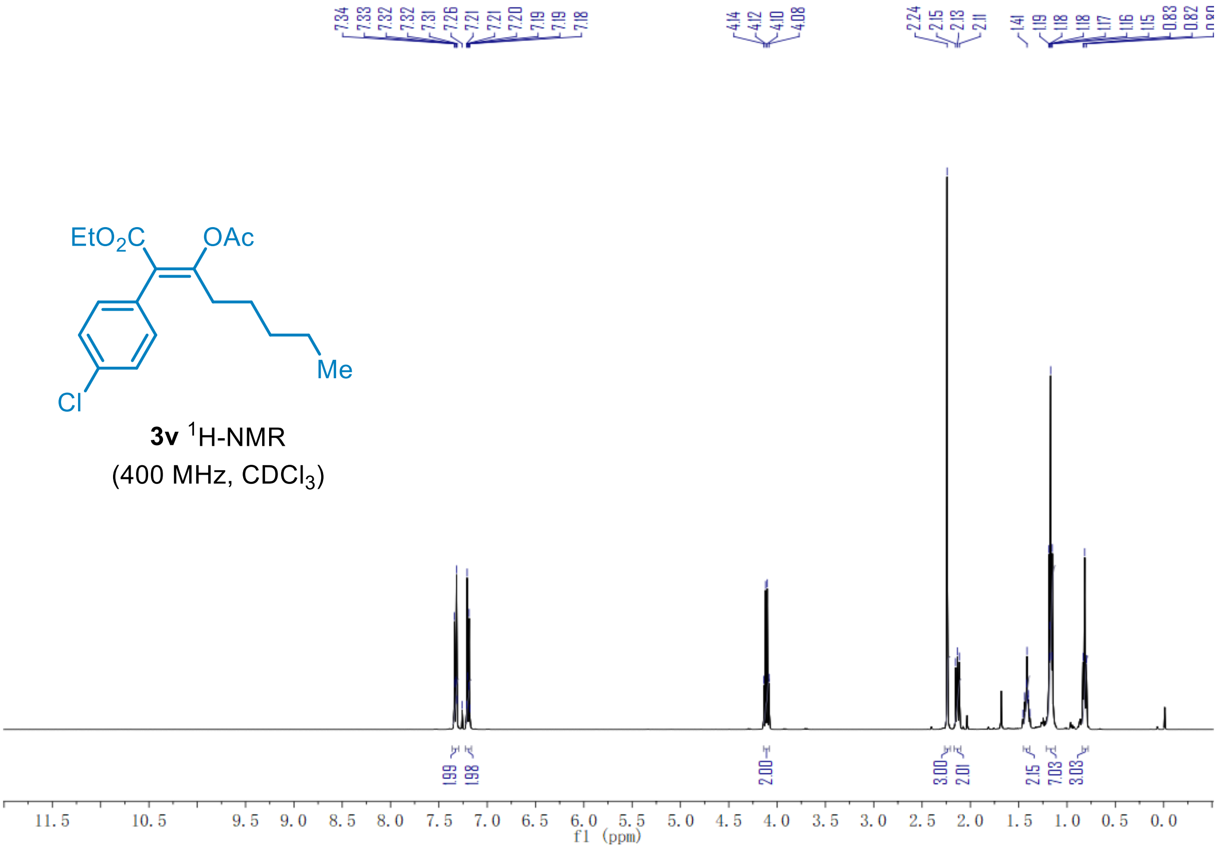

Supplementary Figure 48. **^{1}H-NMR** (400 MHz, $CDCl_3$, 298K) of **3v**

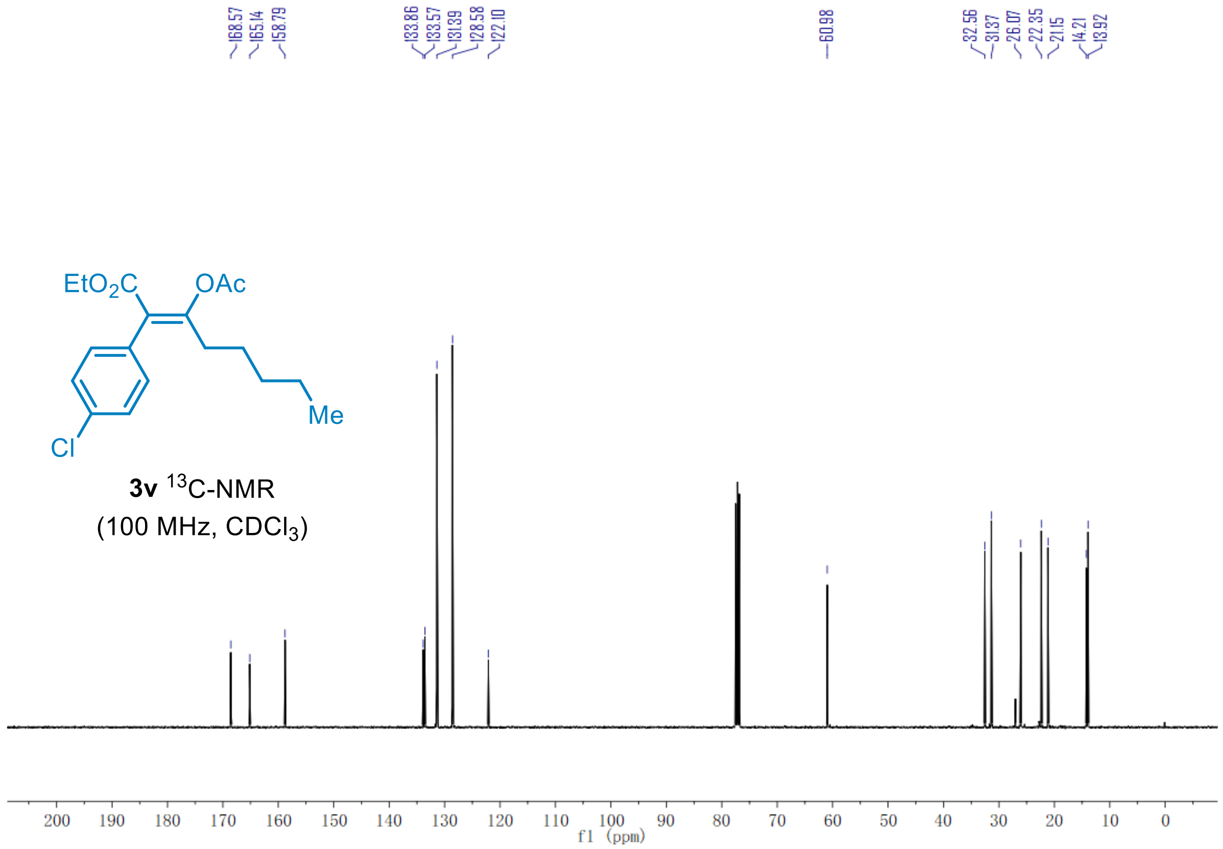

Supplementary Figure 49. **^{13}C-NMR** (100 MHz, $CDCl_3$, 298K) of **3v**

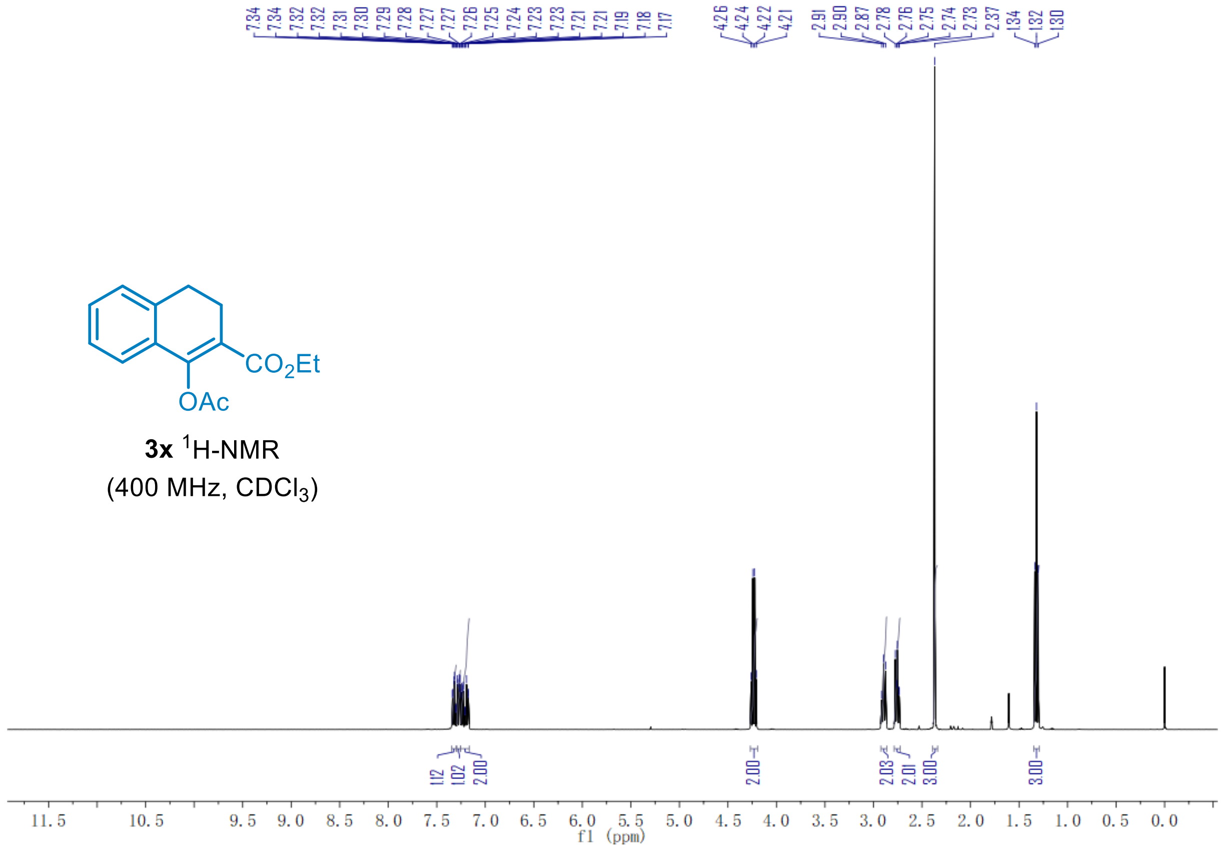

Supplementary Figure 50. **^{1}H-NMR** (400 MHz, $CDCl_3$, 298K) of **3x**

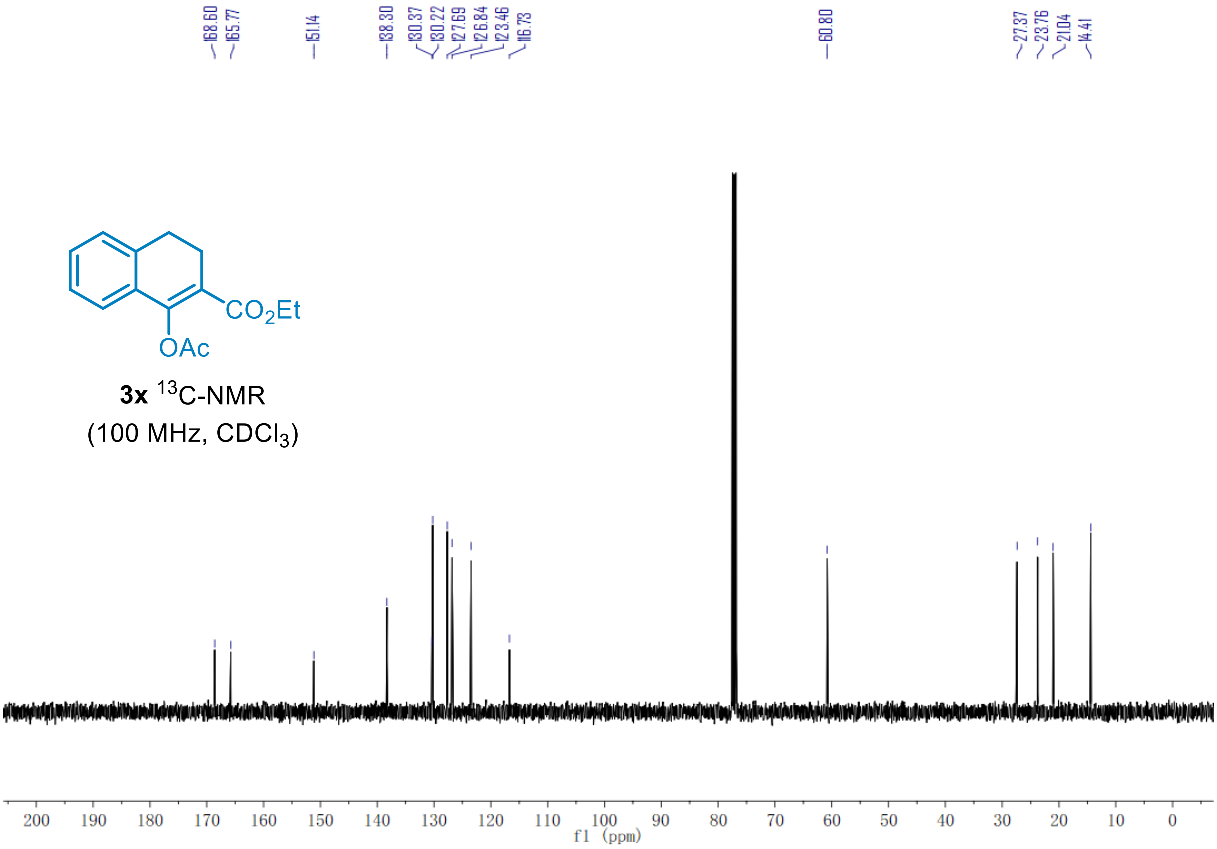

Supplementary Figure 51. **^{13}C-NMR** (100 MHz, $CDCl_3$, 298K) of **3x**

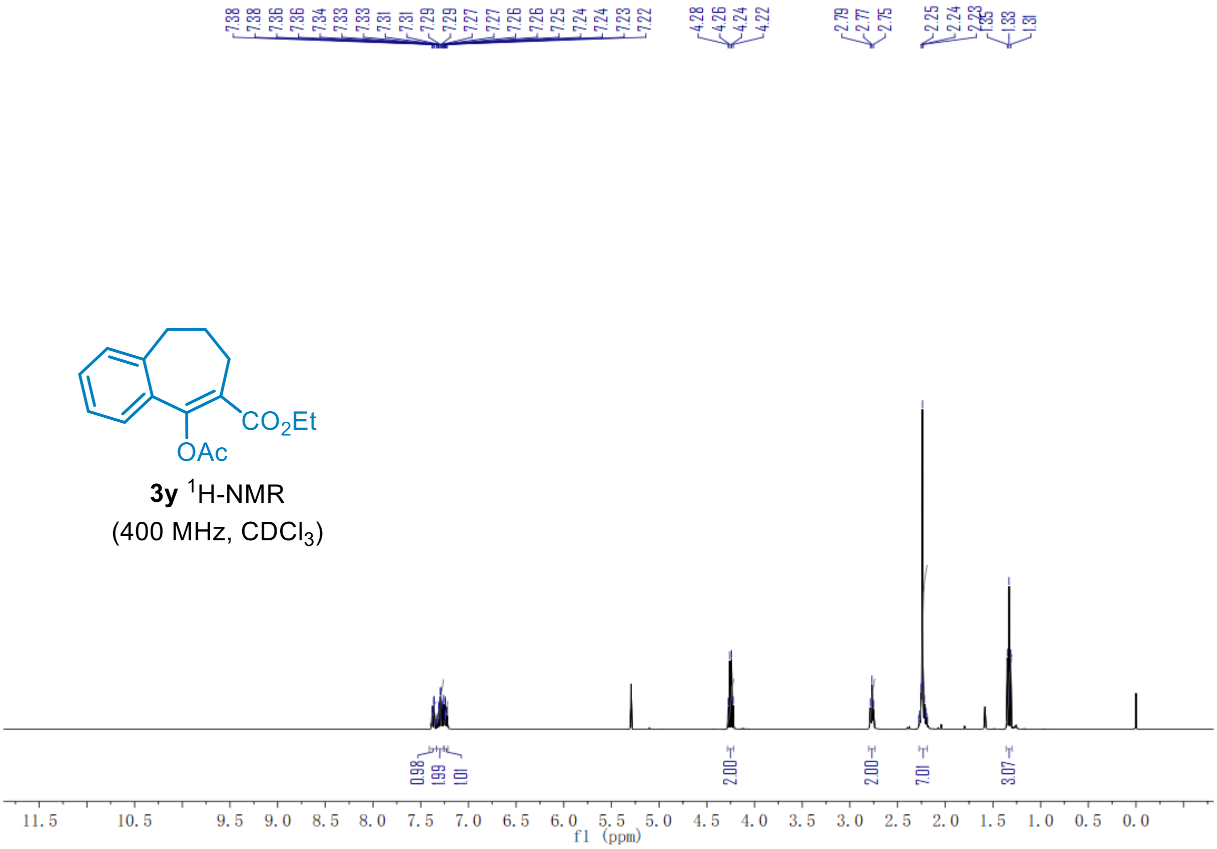

Supplementary Figure 52. **^{1}H-NMR** (400 MHz, $CDCl_3$, 298K) of **3y**

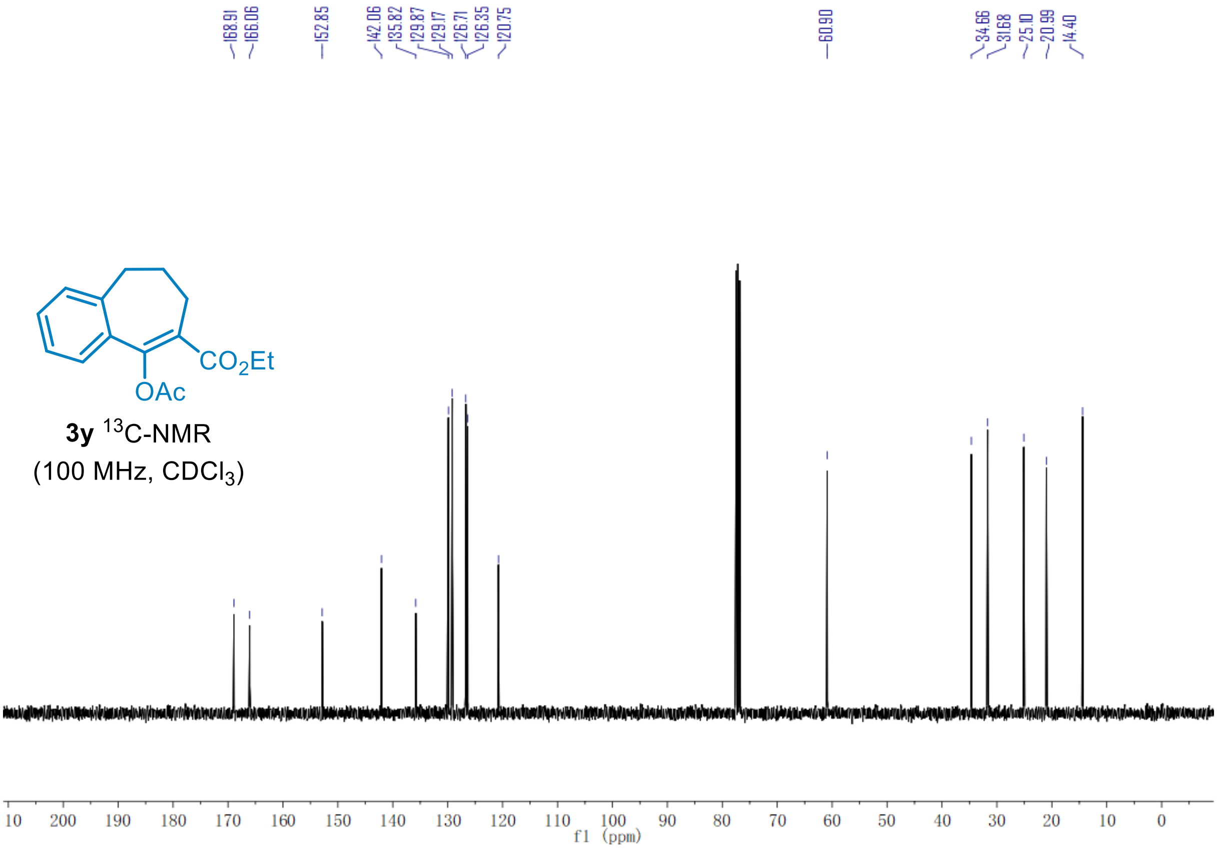

Supplementary Figure 53. **^{13}C-NMR** (100 MHz, $CDCl_3$, 298K) of **3y**

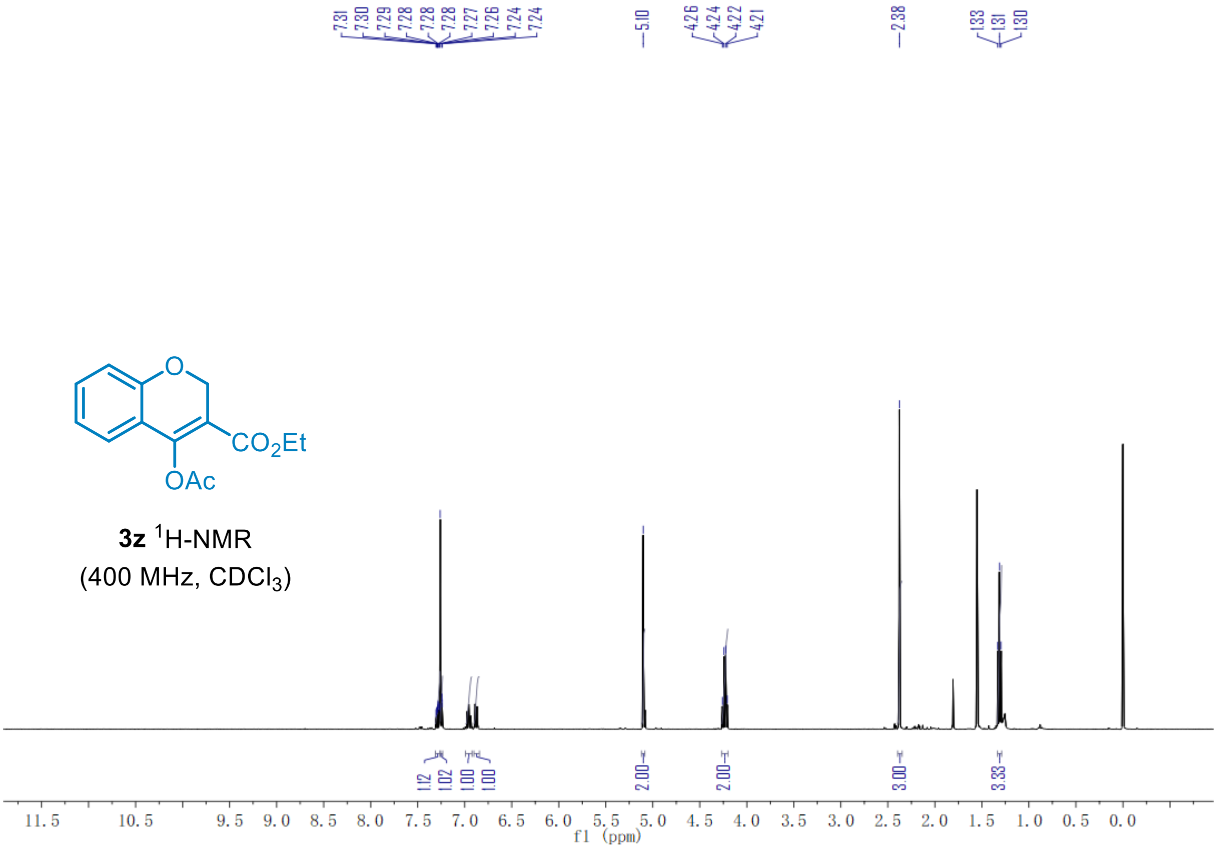

Supplementary Figure 54. **^{1}H-NMR** (400 MHz, $CDCl_3$, 298K) of **3z**

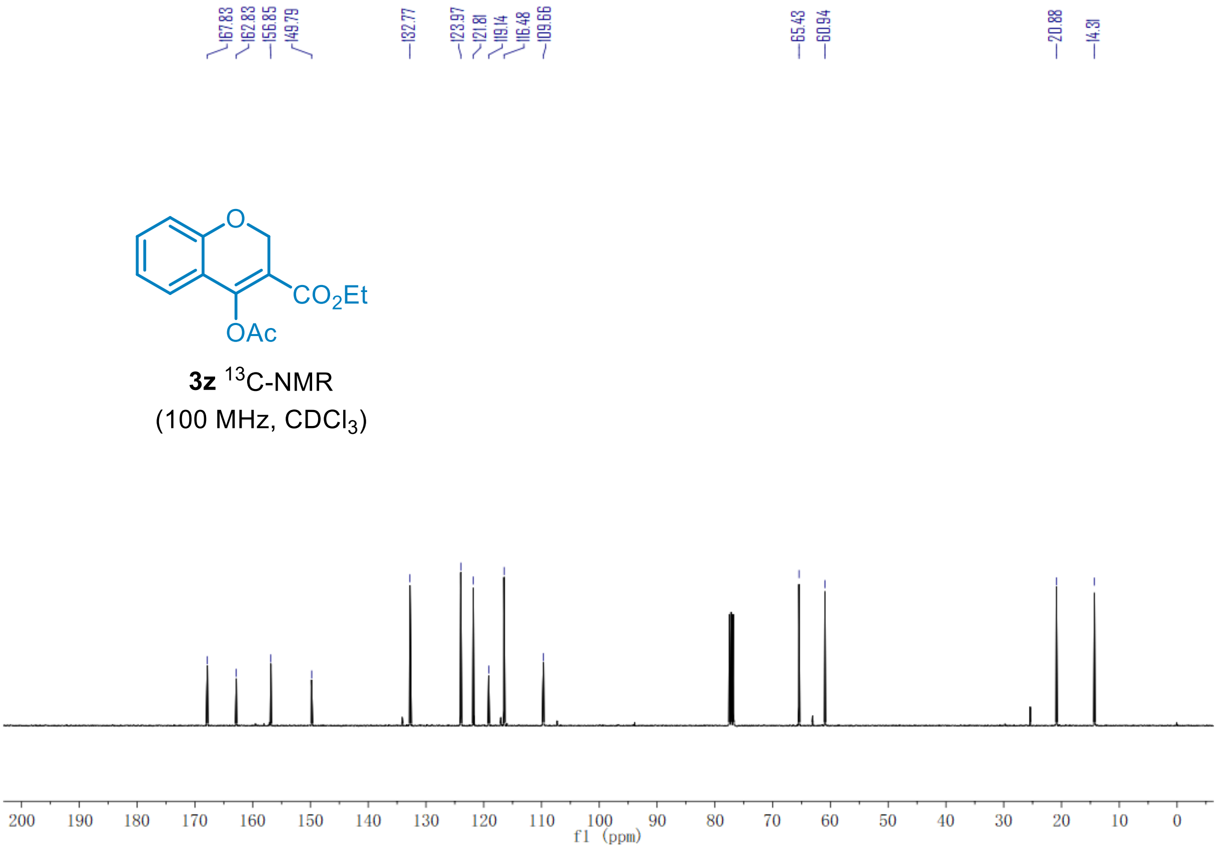

Supplementary Figure 55. **^{13}C-NMR** (100 MHz, $CDCl_3$, 298K) of **3z**

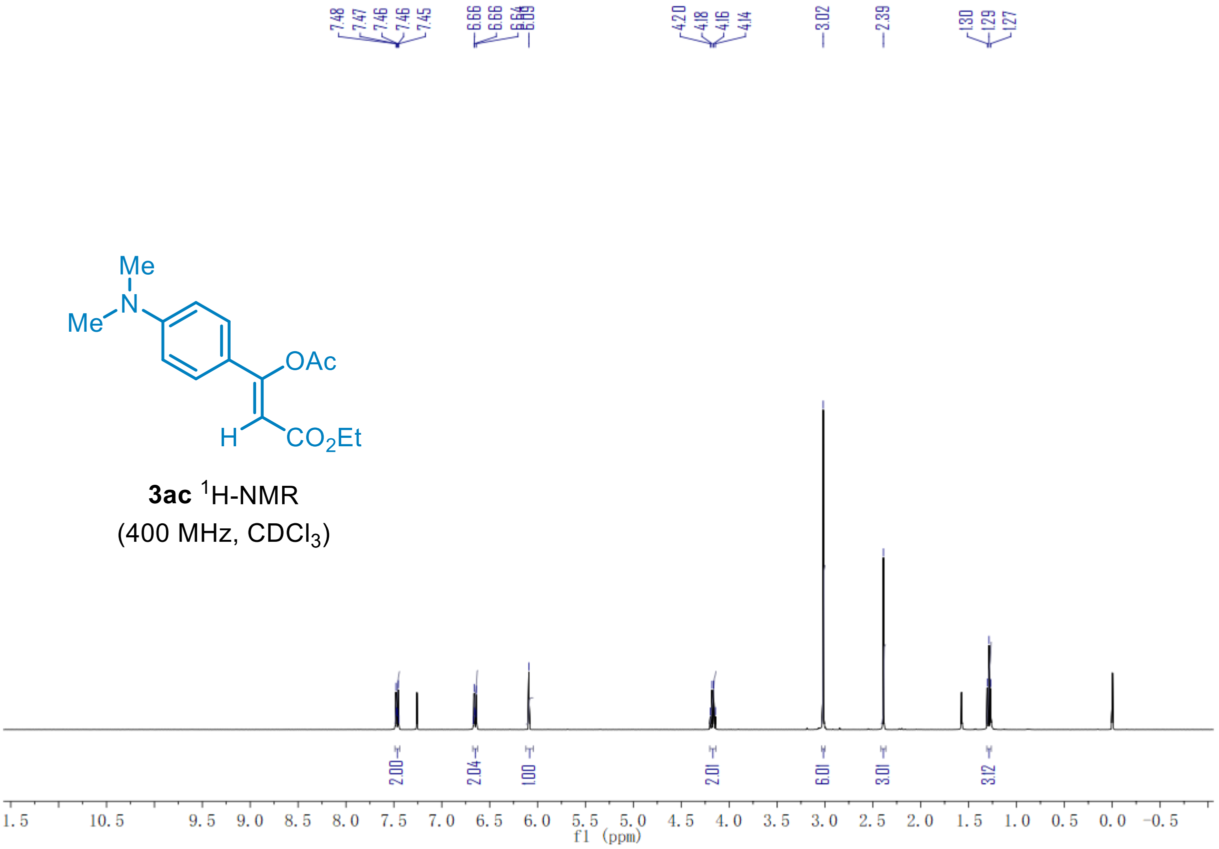

Supplementary Figure 56. **^{1}H-NMR** (400 MHz, $CDCl_3$, 298K) of **3ac**

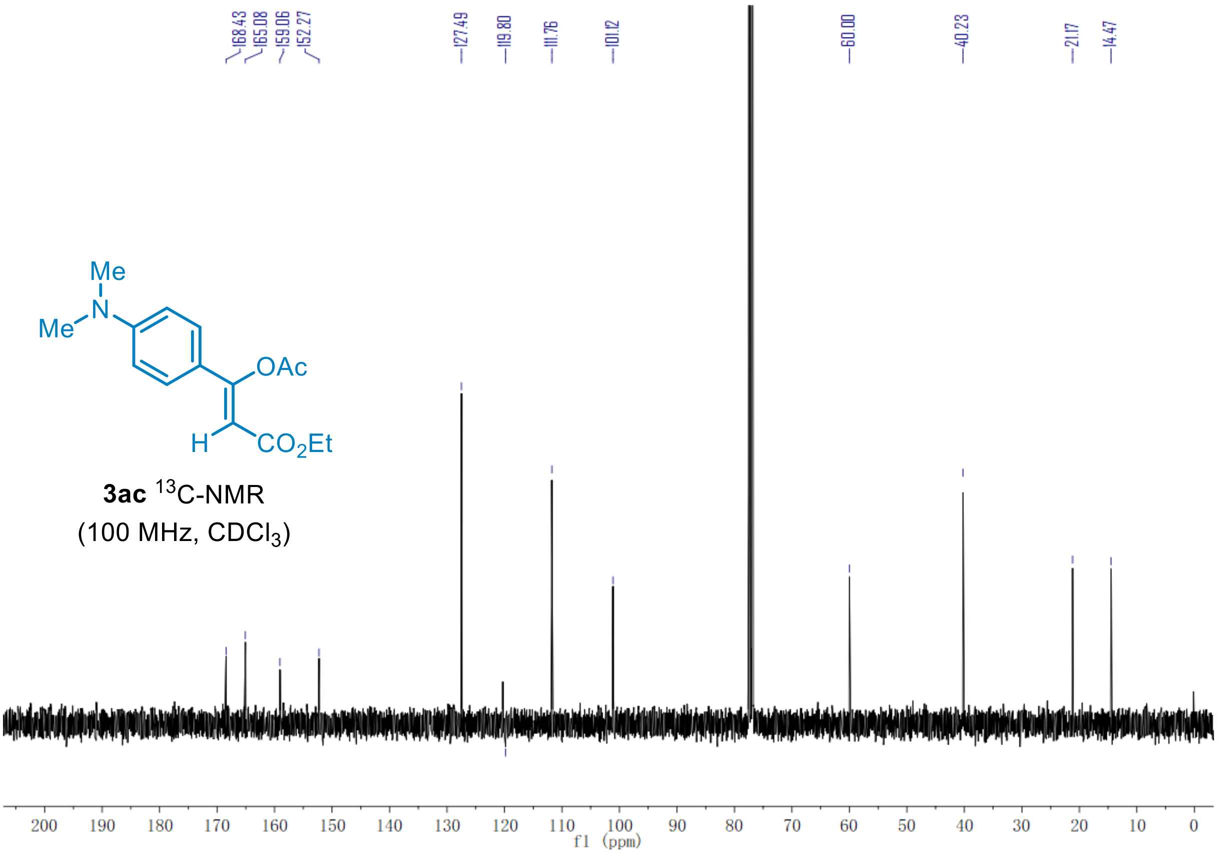

Supplementary Figure 57. **^{13}C-NMR** (100 MHz, $CDCl_3$, 298K) of **3ac**

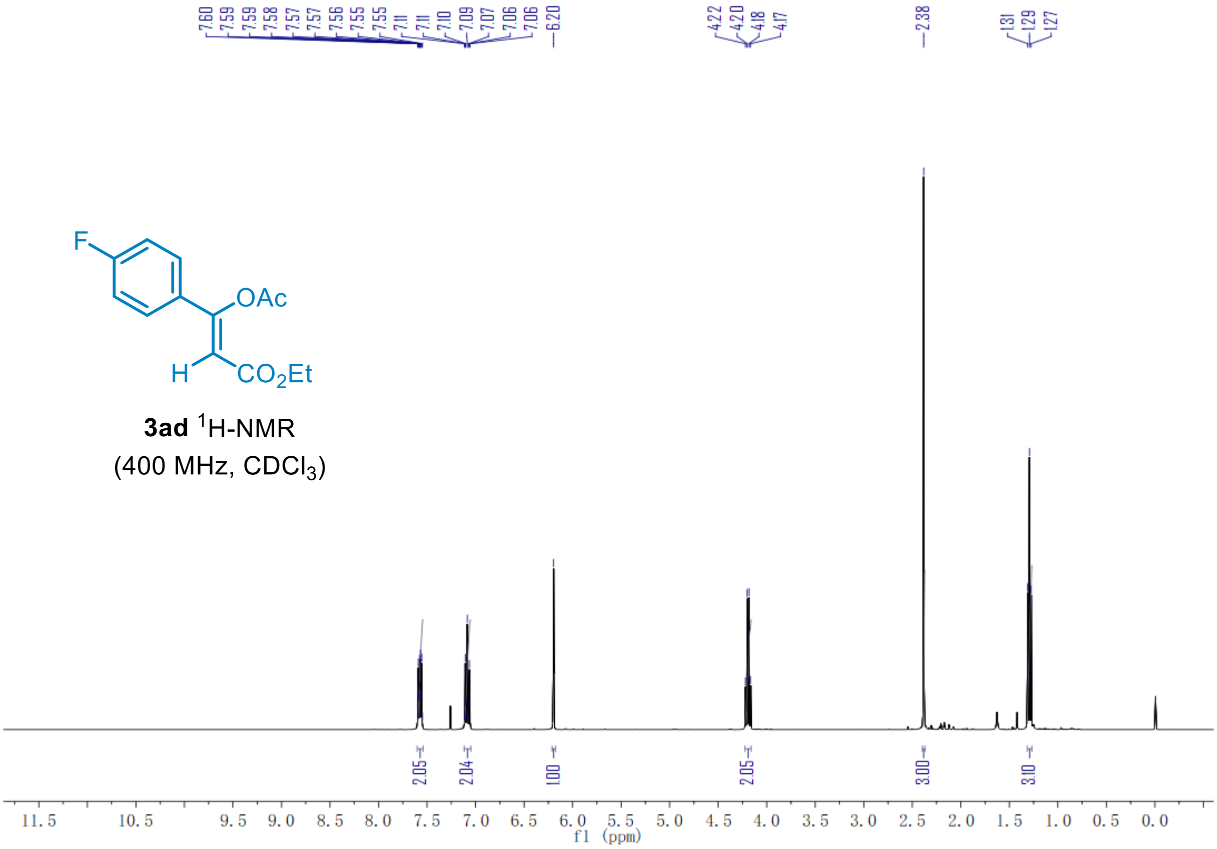

Supplementary Figure 58. **^{1}H-NMR** (400 MHz, $CDCl_3$, 298K) of **3ad**

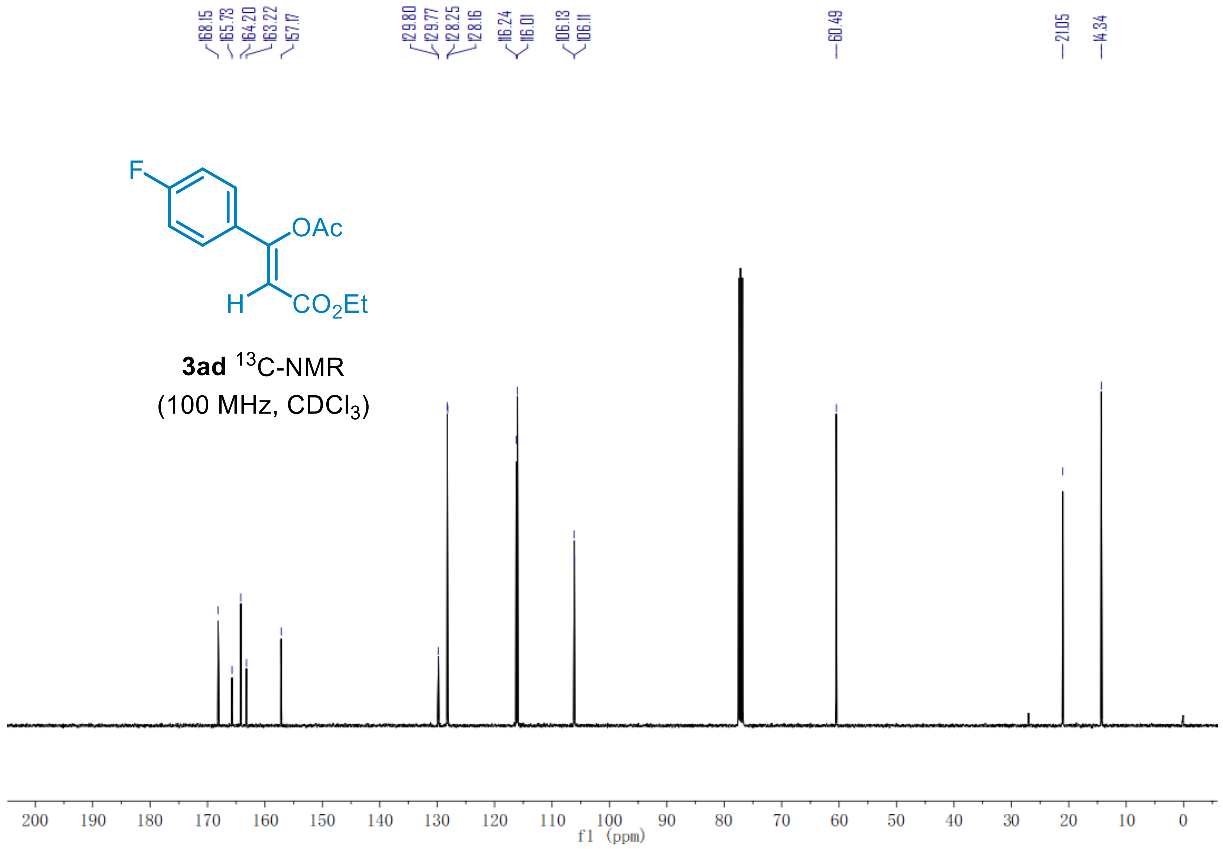

Supplementary Figure 59. **^{13}C-NMR** (100 MHz, $CDCl_3$, 298K) of **3ad**

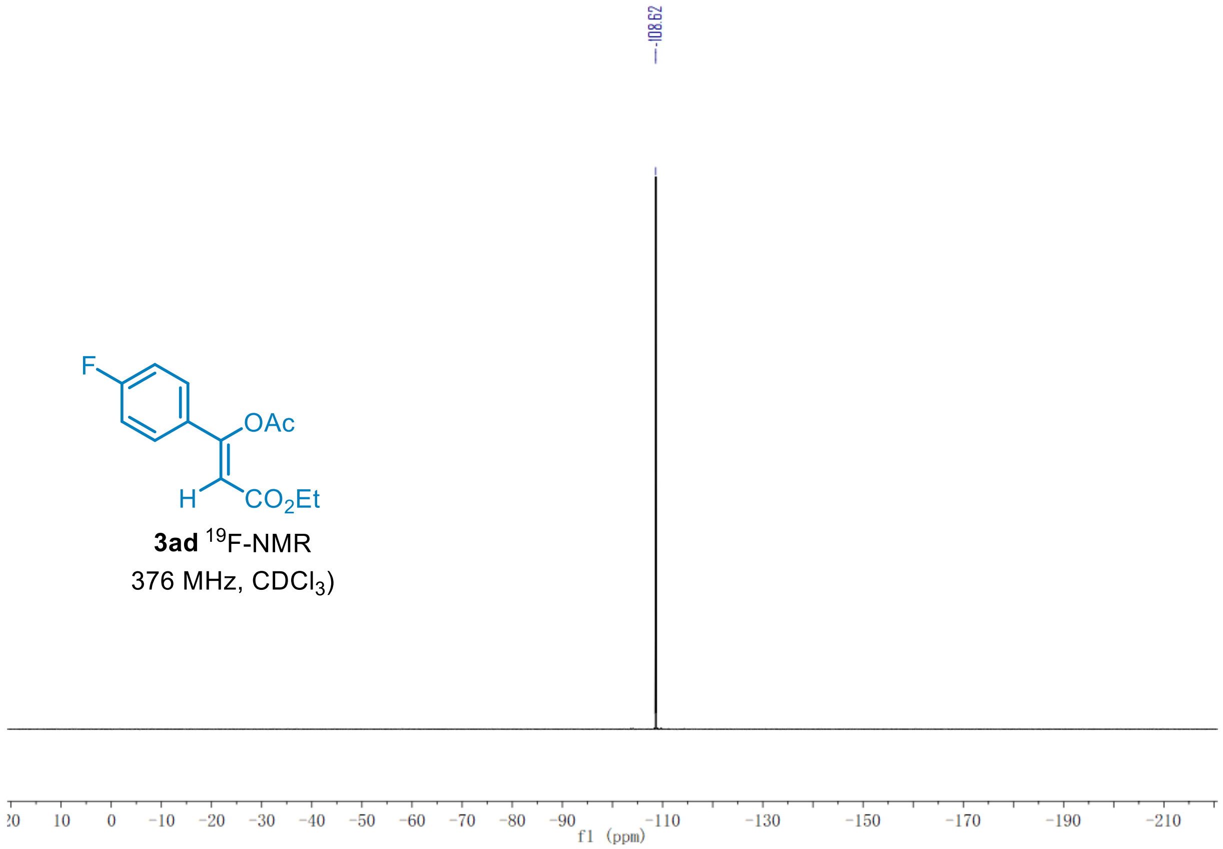

Supplementary Figure 60. **^{19}F-NMR** (376 MHz, $CDCl_3$, 298K) of **3ad**

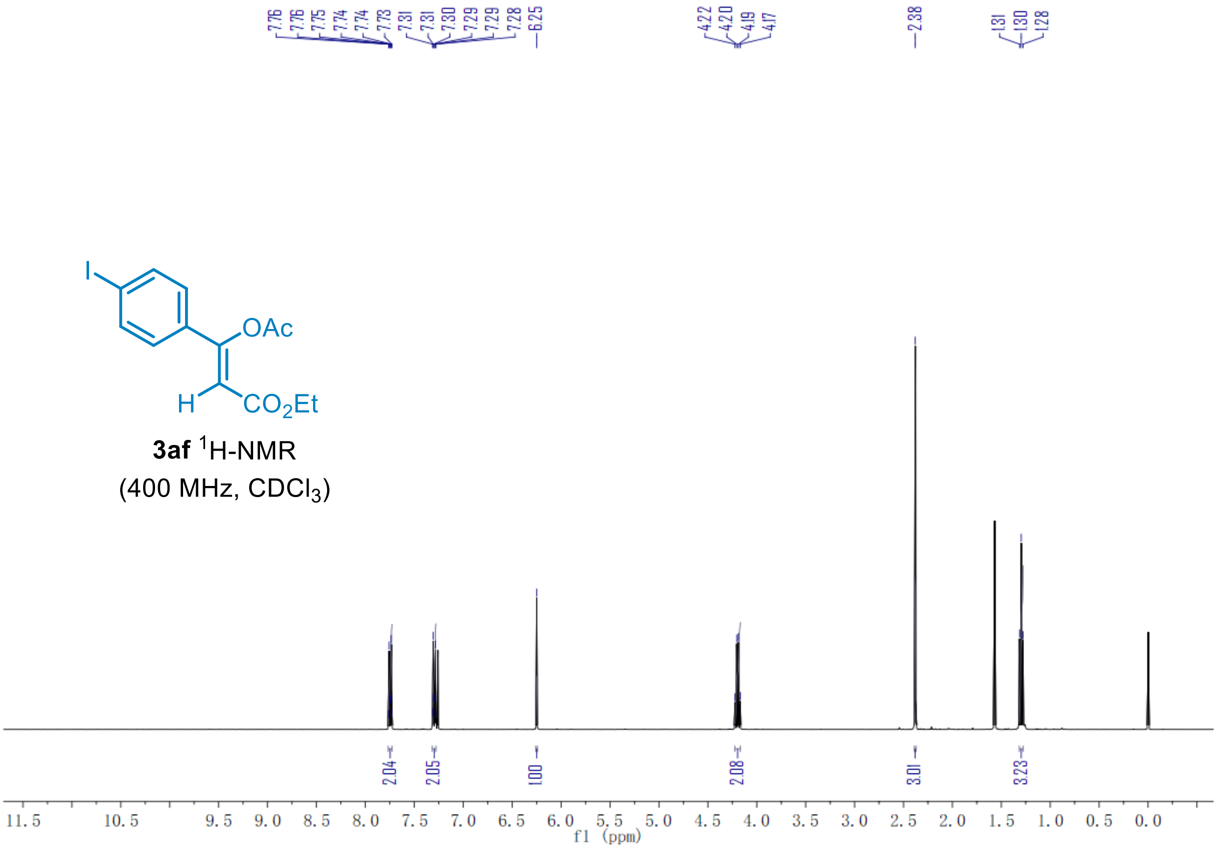

Supplementary Figure 61. **^{1}H-NMR** (400 MHz, $CDCl_3$, 298K) of **3af**

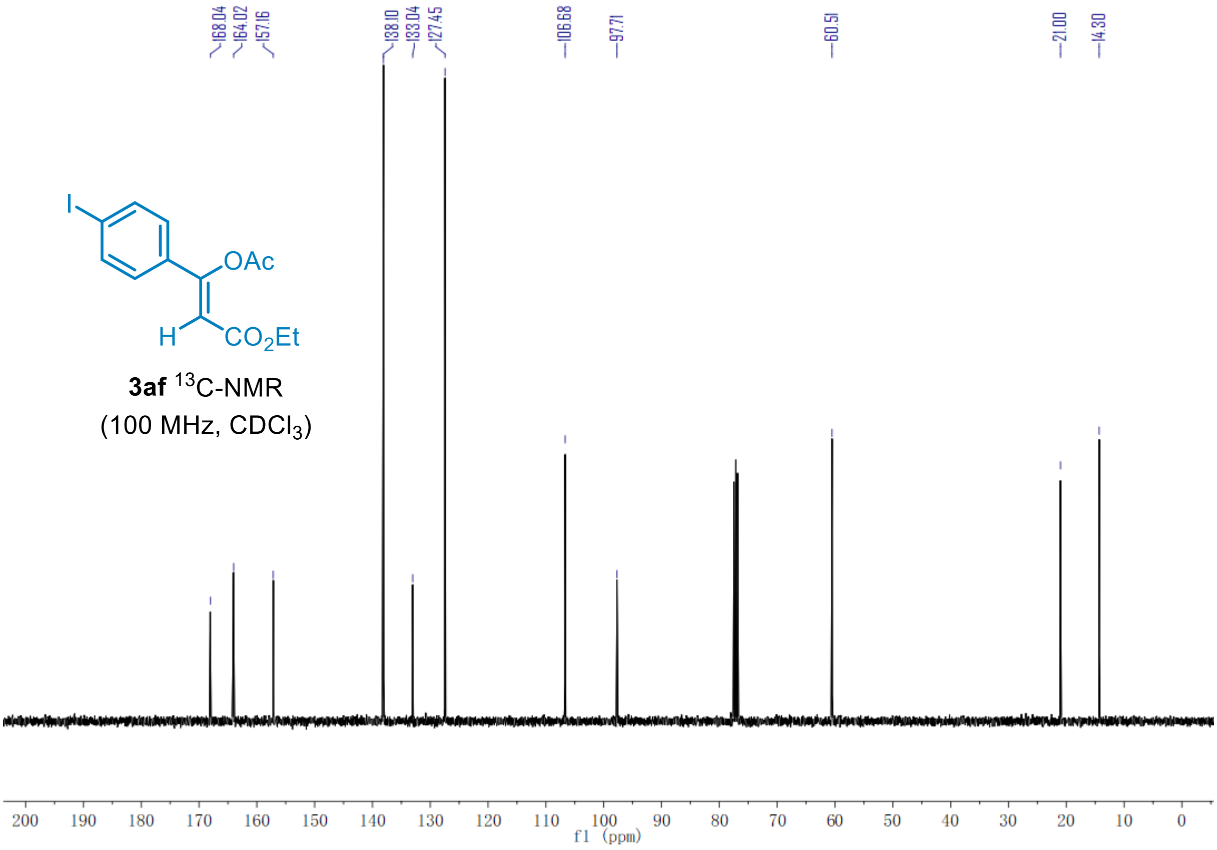

Supplementary Figure 62. **^{13}C-NMR** (100 MHz, $CDCl_3$, 298K) of **3af**

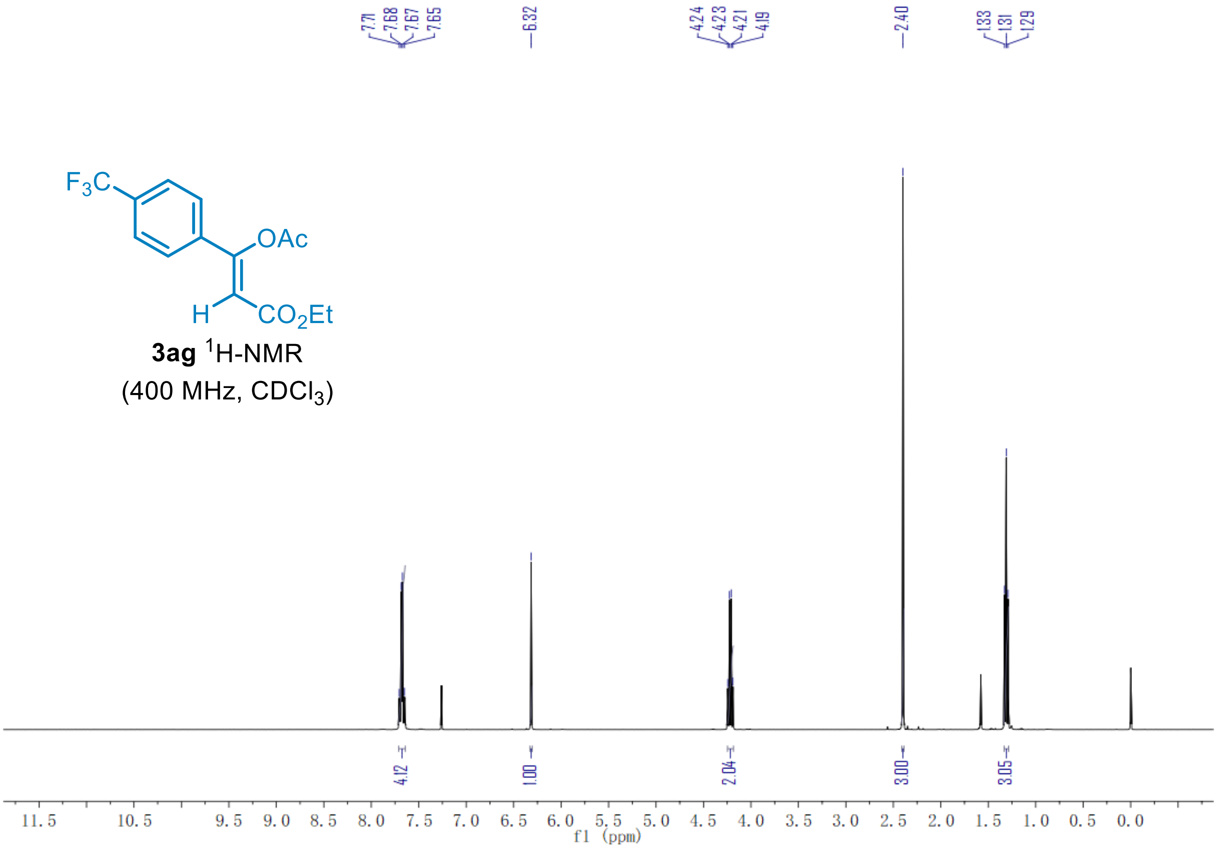

Supplementary Figure 63. **^{1}H-NMR** (400 MHz, $CDCl_3$, 298K) of **3ag**

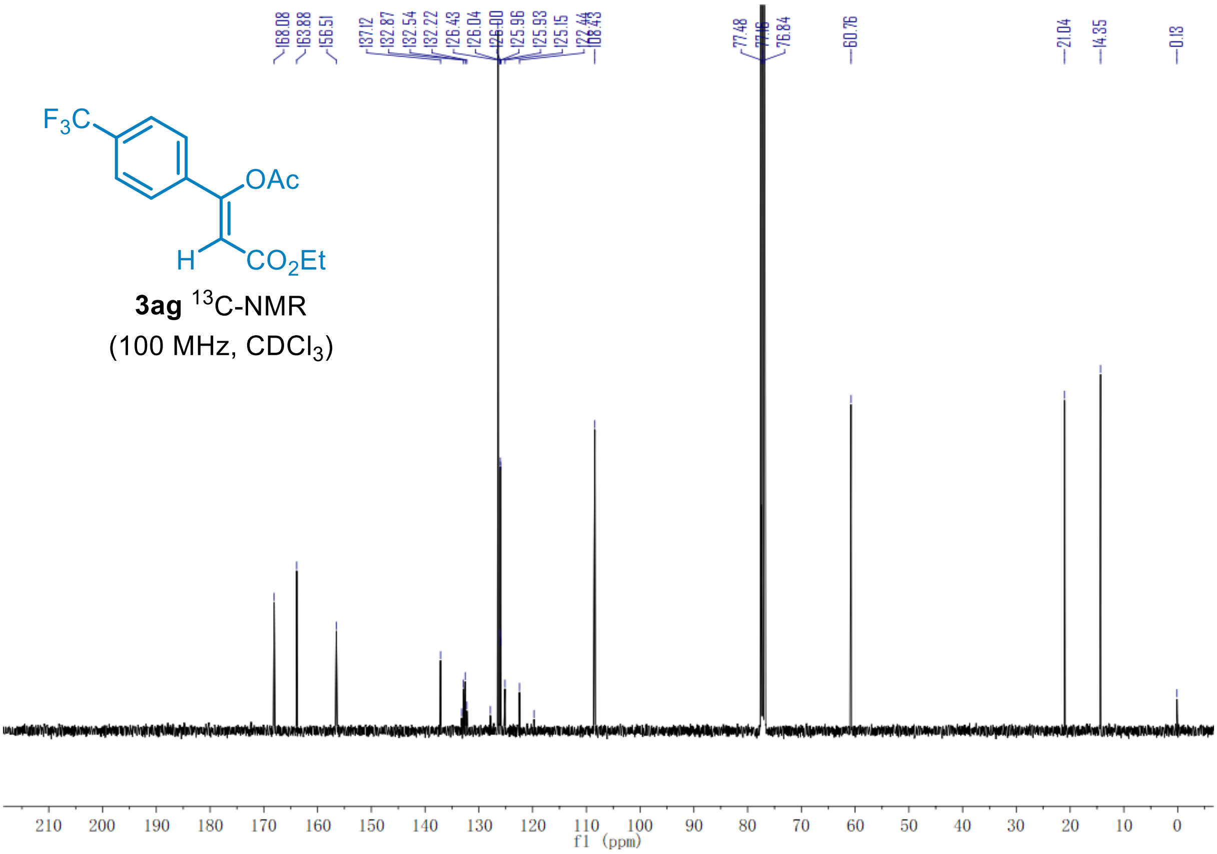

Supplementary Figure 64. **^{13}C-NMR** (100 MHz, $CDCl_3$, 298K) of **3ag**

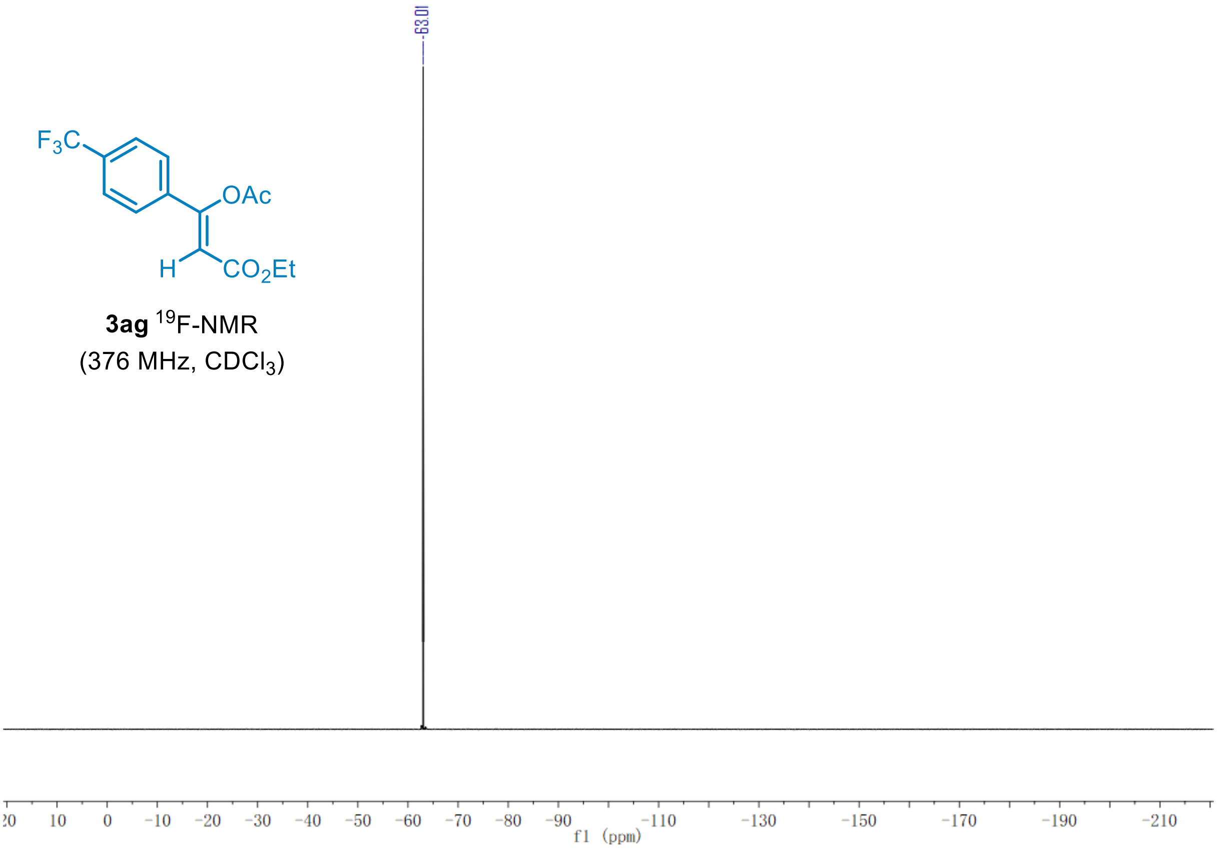

Supplementary Figure 65. **^{19}F-NMR** (376 MHz, $CDCl_3$, 298K) of **3ag**

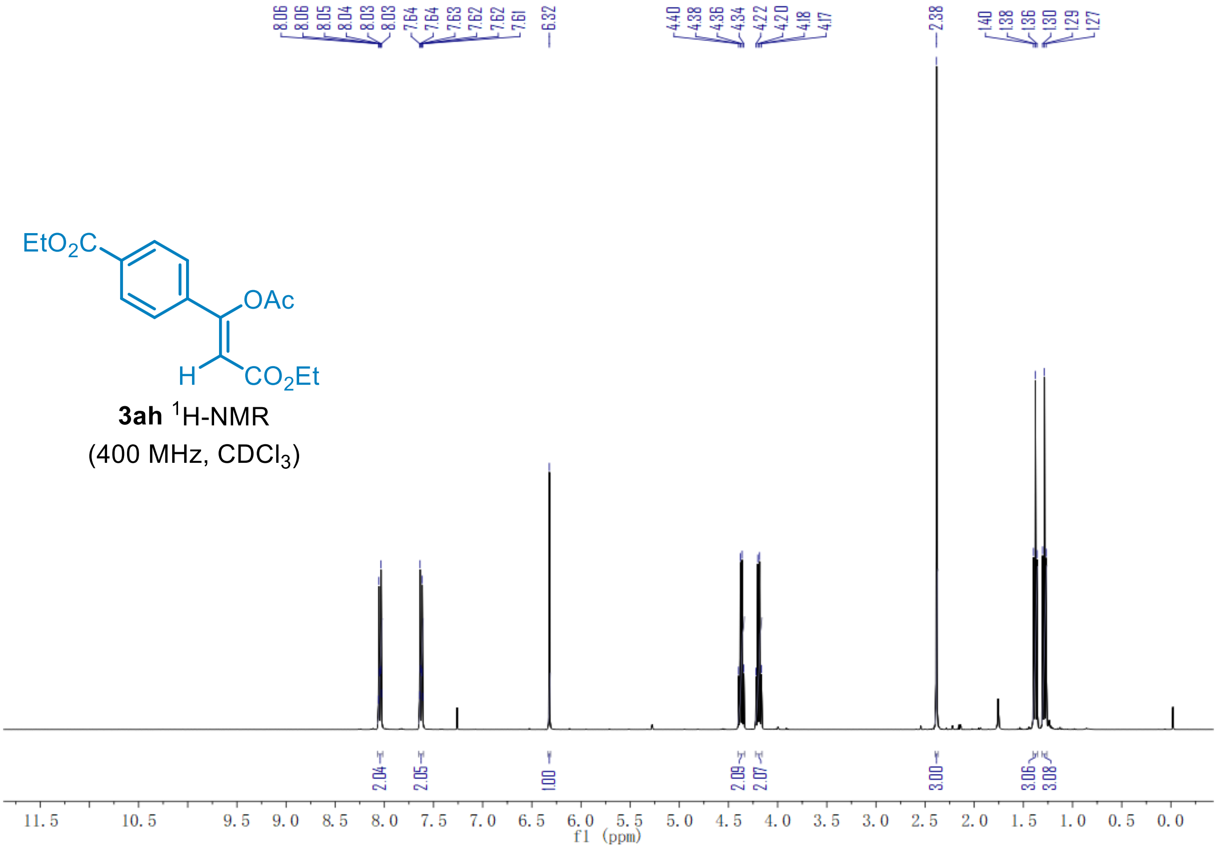

Supplementary Figure 66. **^{1}H-NMR** (400 MHz, $CDCl_3$, 298K) of **3ah**

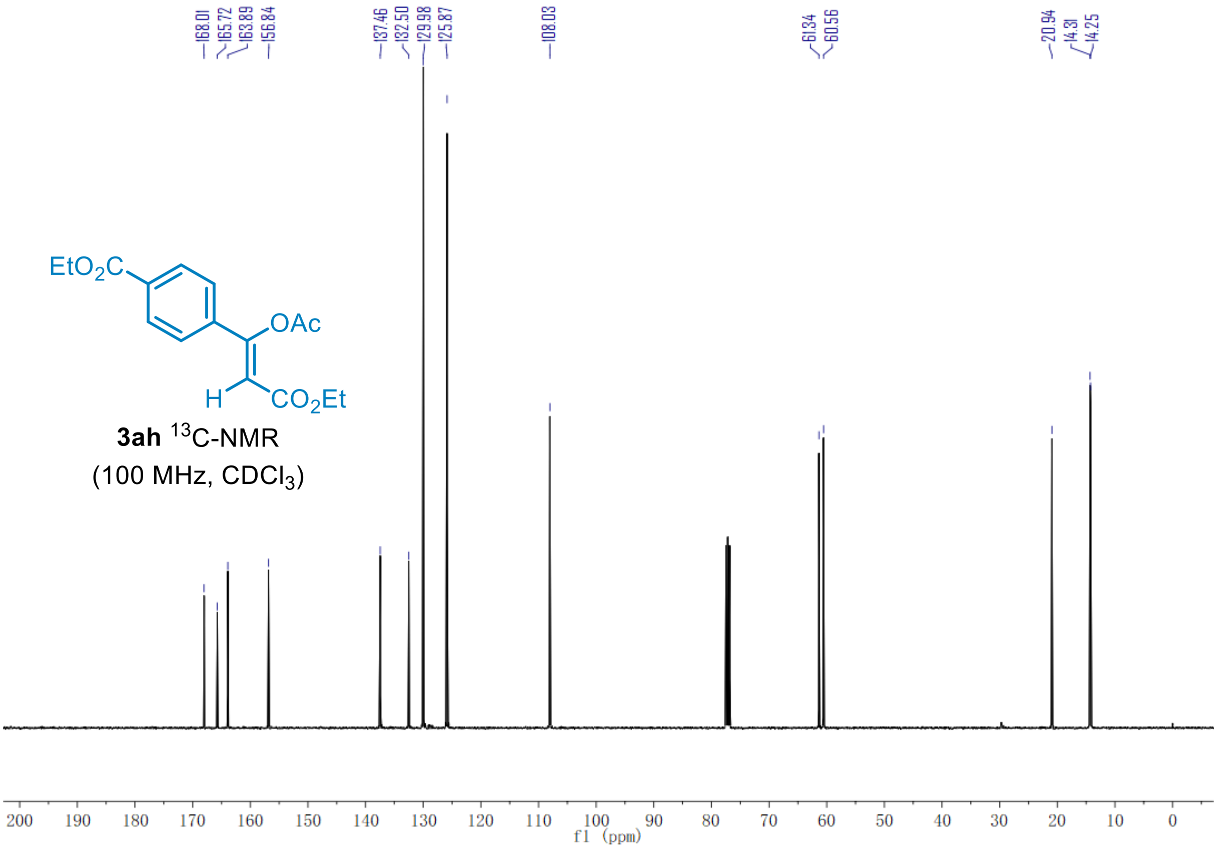

Supplementary Figure 67. **^{13}C-NMR** (100 MHz, $CDCl_3$, 298K) of **3ah**

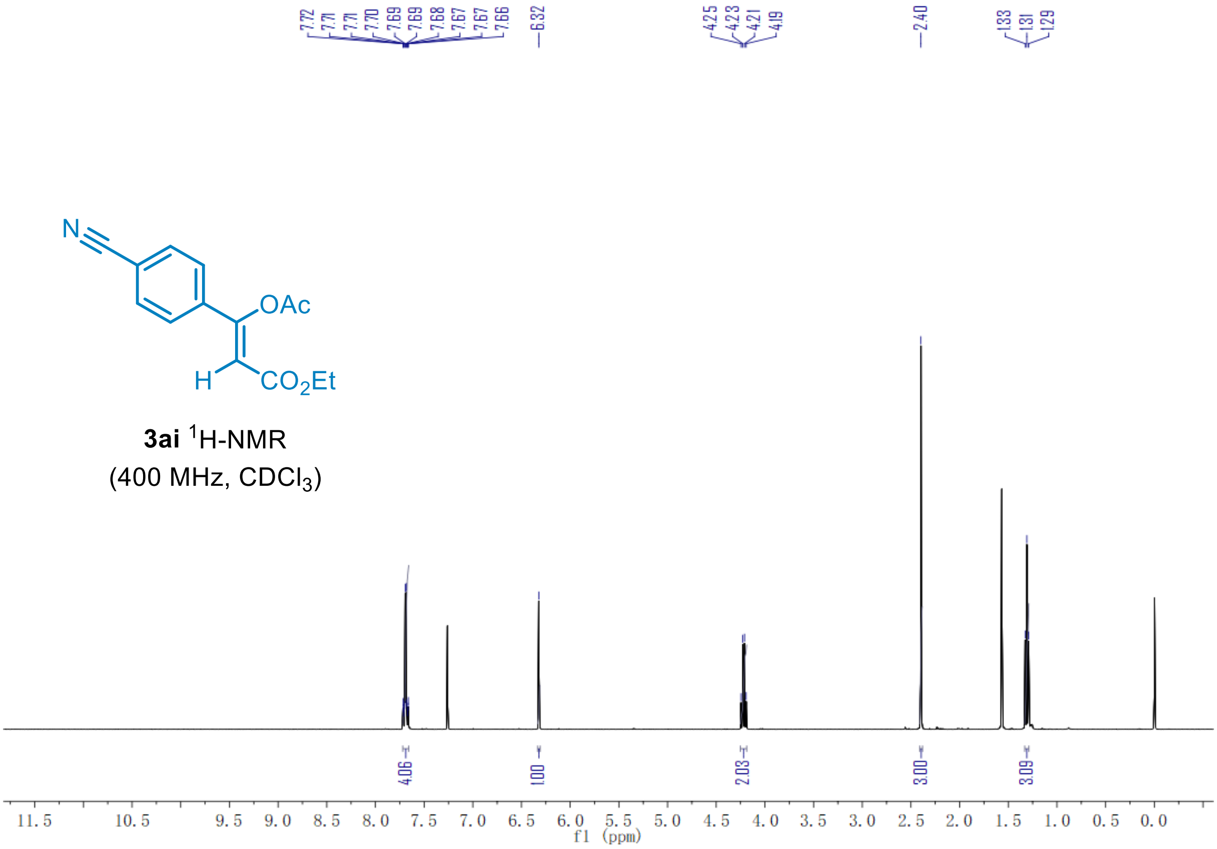

Supplementary Figure 68. **^{1}H-NMR** (400 MHz, $CDCl_3$, 298K) of **3ai**

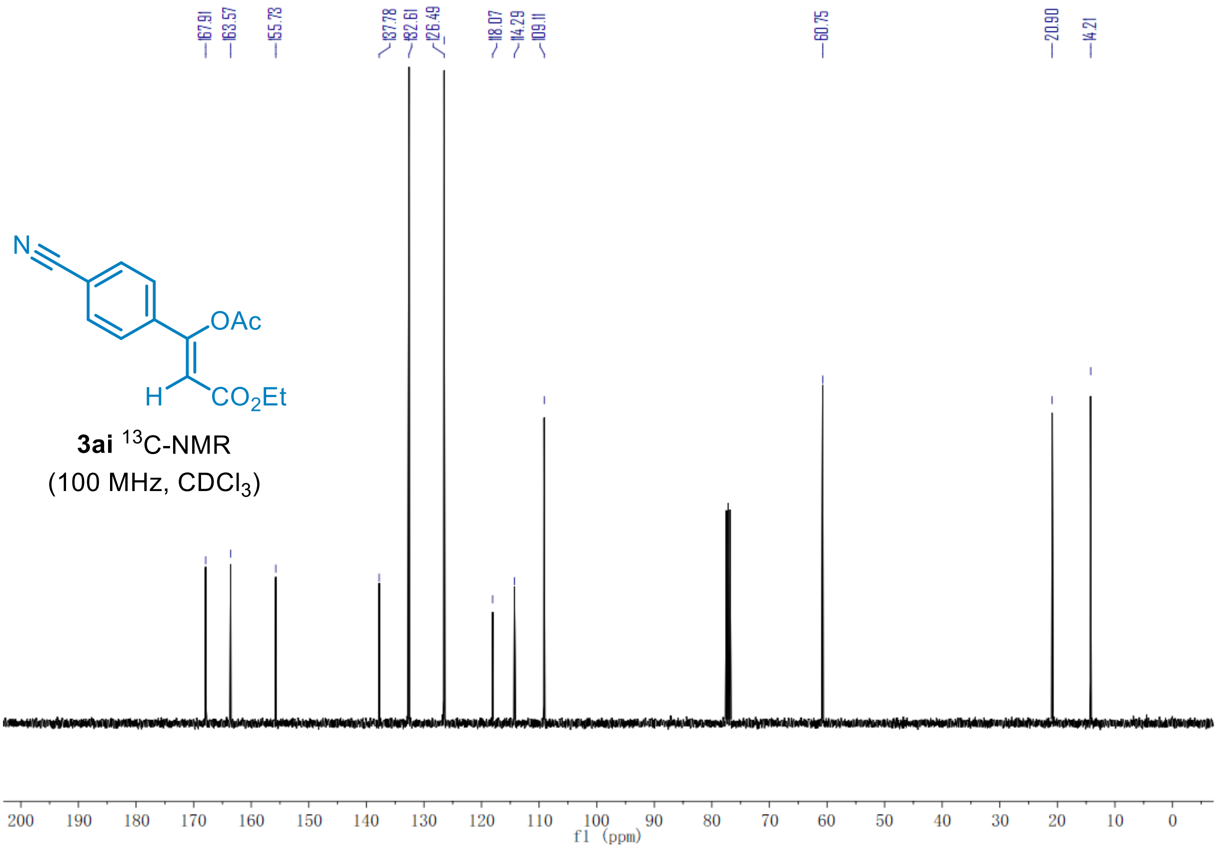

Supplementary Figure 69. **^{13}C-NMR** (100 MHz, $CDCl_3$, 298K) of **3ai**

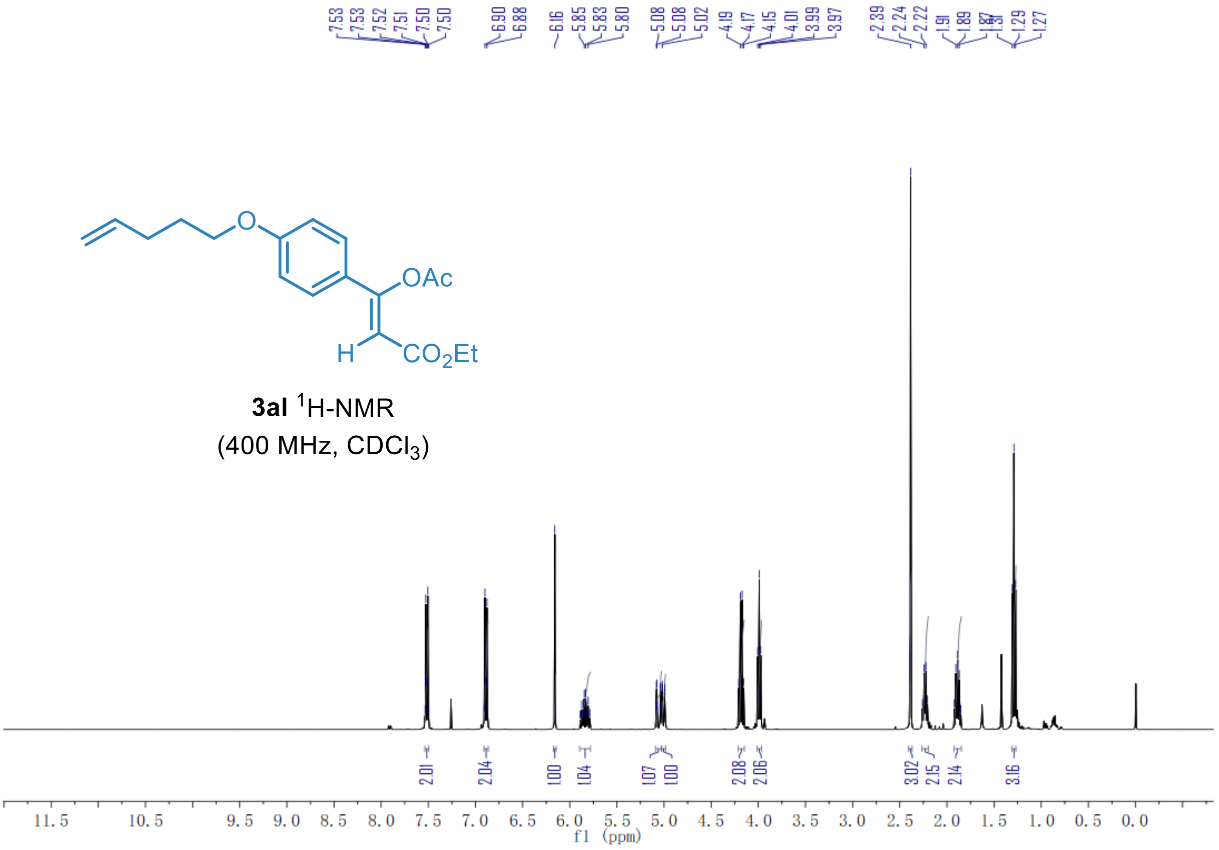

Supplementary Figure 70. **^{1}H-NMR** (400 MHz, $CDCl_3$, 298K) of **3al**

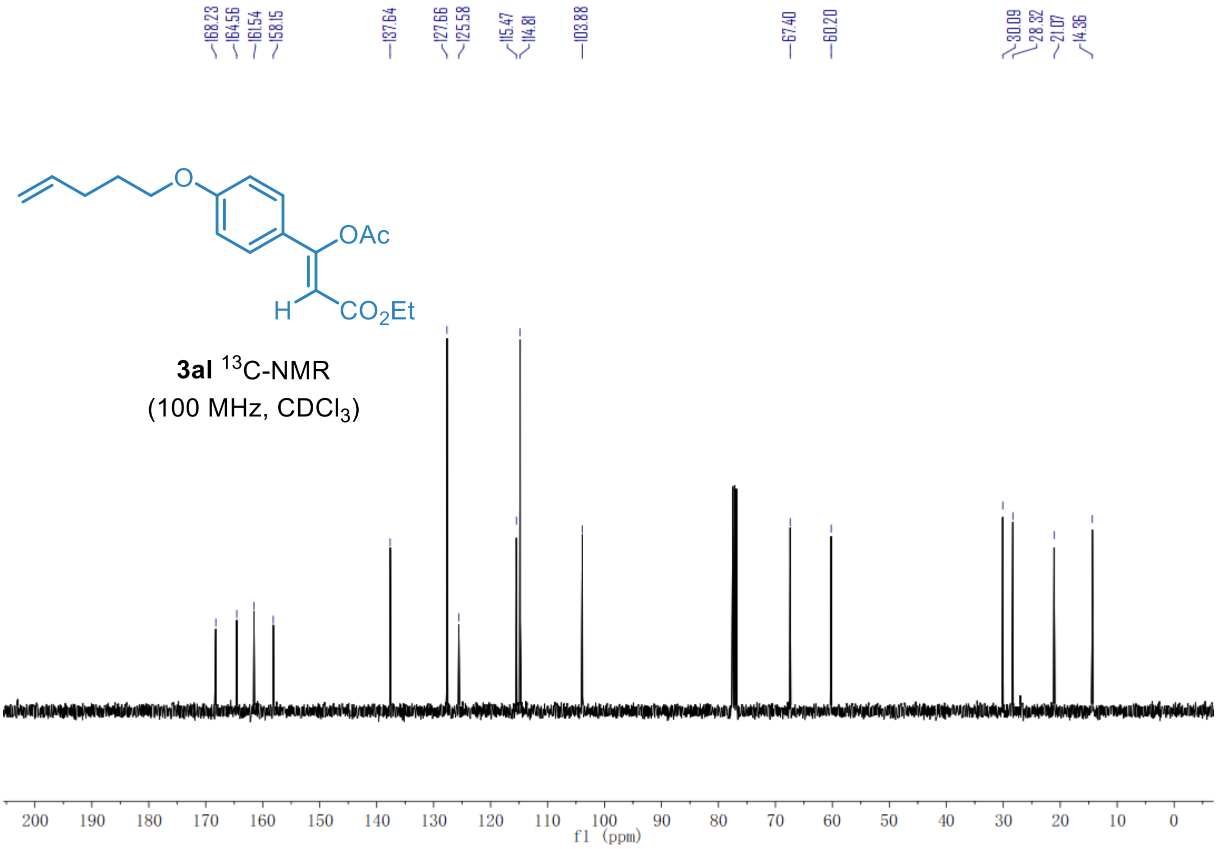

Supplementary Figure 71. **^{13}C-NMR** (100 MHz, $CDCl_3$, 298K) of **3al**

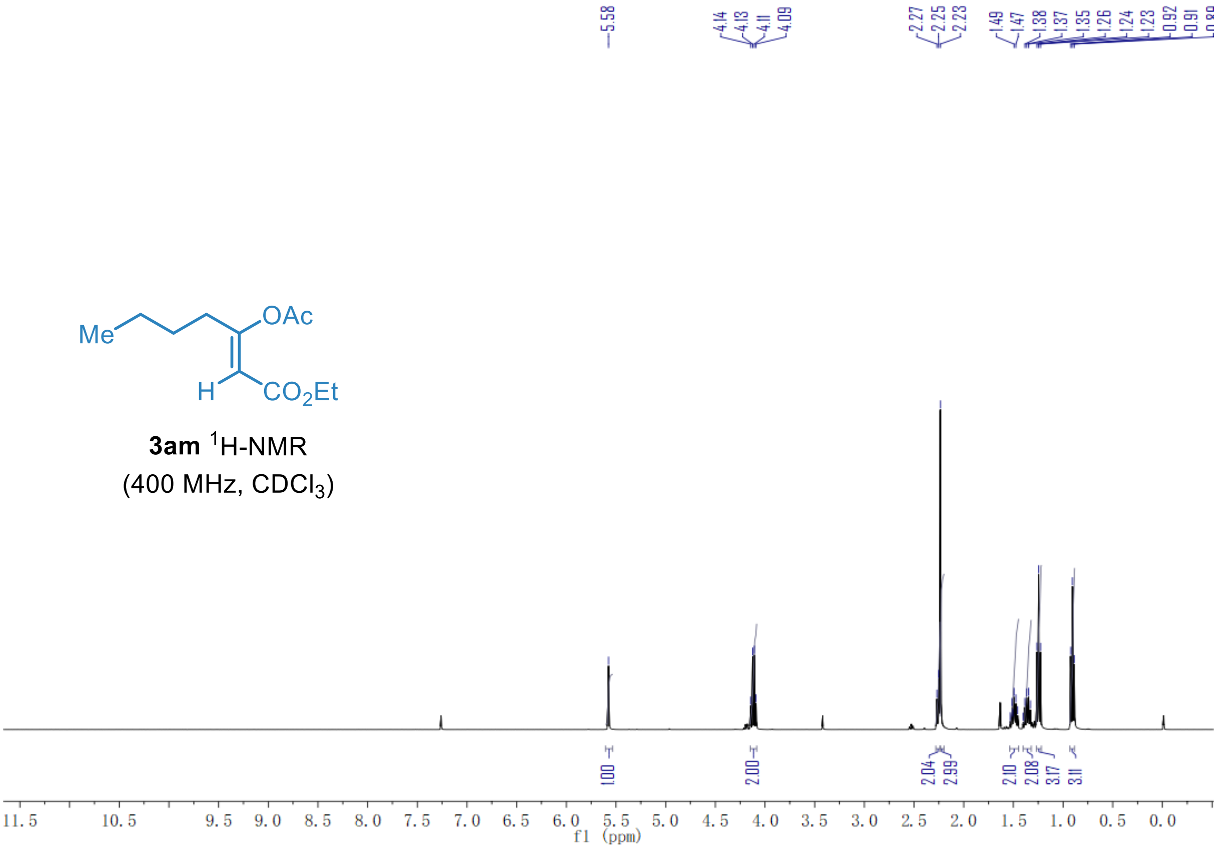

Supplementary Figure 72. **^{1}H-NMR** (400 MHz, $CDCl_3$, 298K) of **3am**

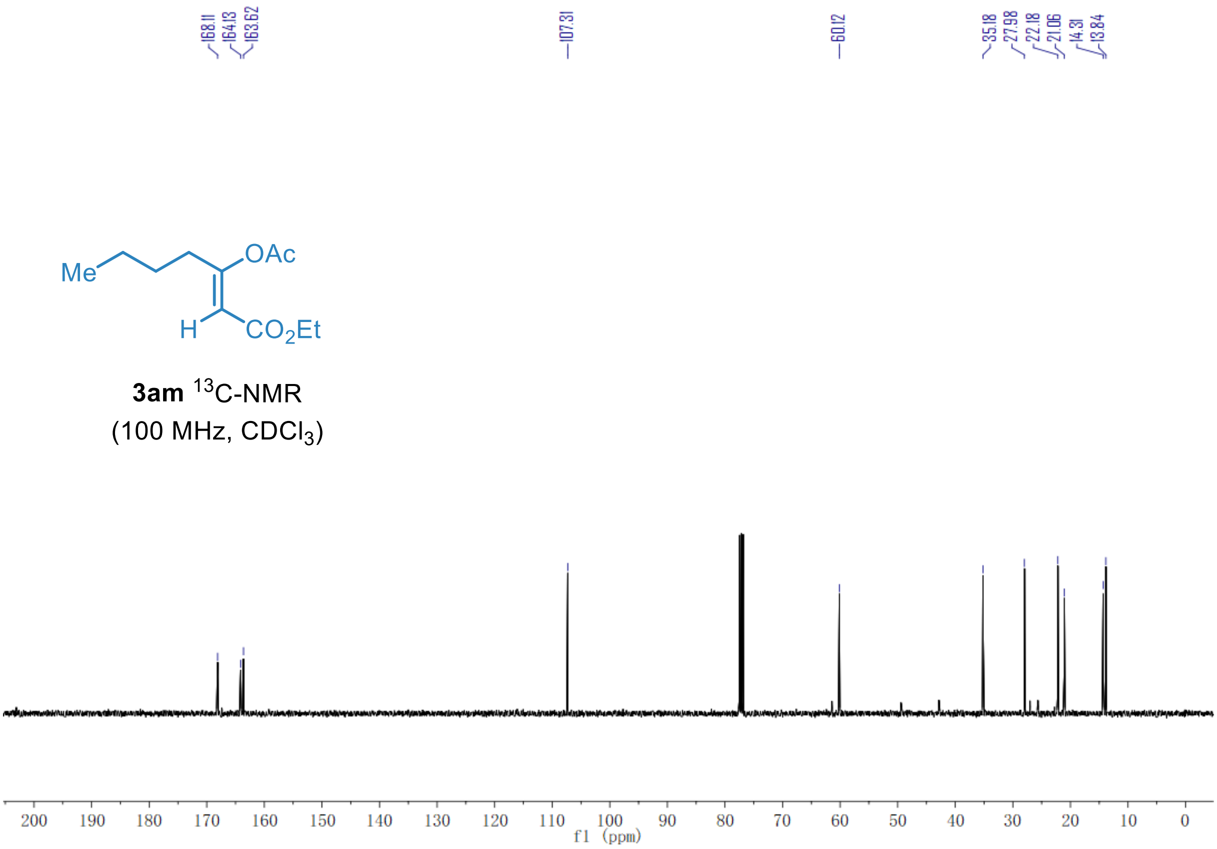

Supplementary Figure 73. **^{13}C-NMR** (100 MHz, $CDCl_3$, 298K) of **3am**

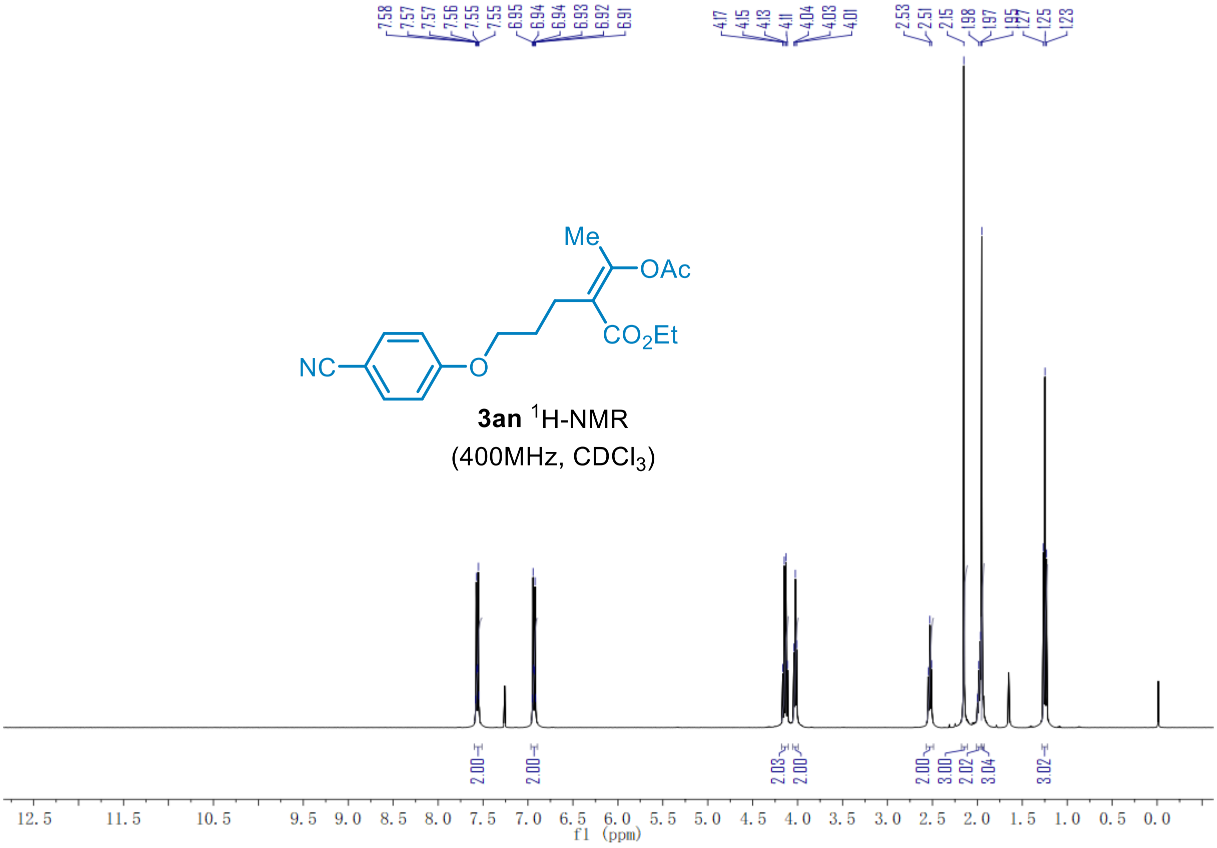

Supplementary Figure 74. **^{1}H-NMR** (400 MHz, $CDCl_3$, 298K) of **3an**

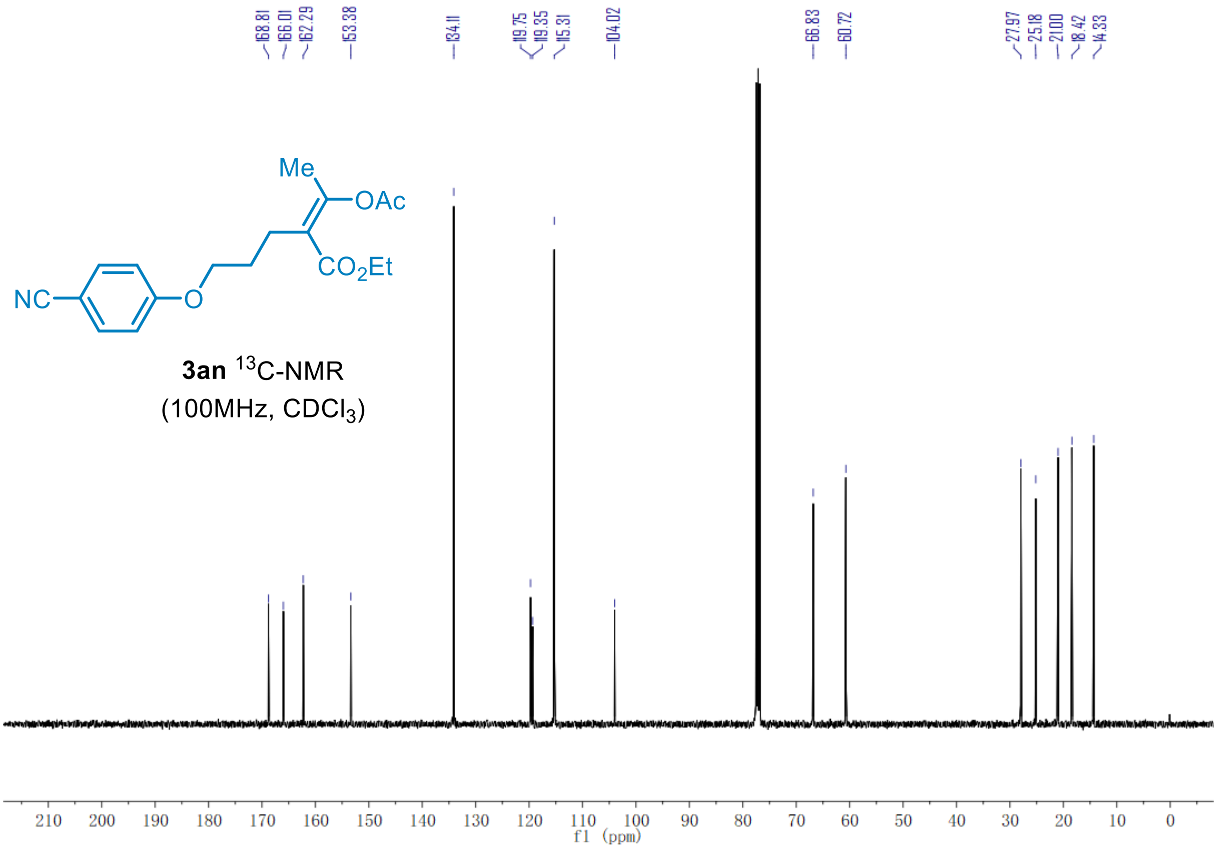

Supplementary Figure 75. **^{13}C-NMR** (100 MHz, $CDCl_3$, 298K) of **3an**

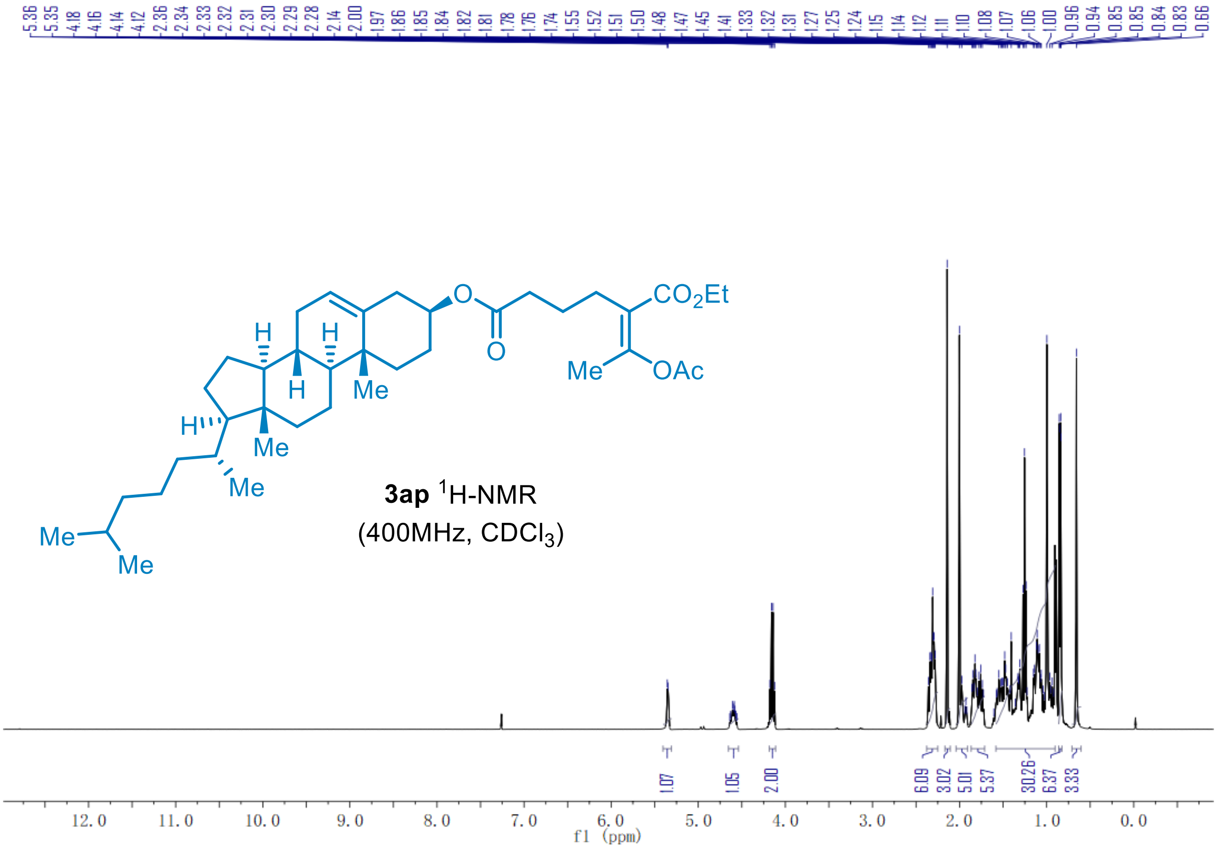

Supplementary Figure 76. **^{1}H-NMR** (400 MHz, $CDCl_3$, 298K) of **3ap**

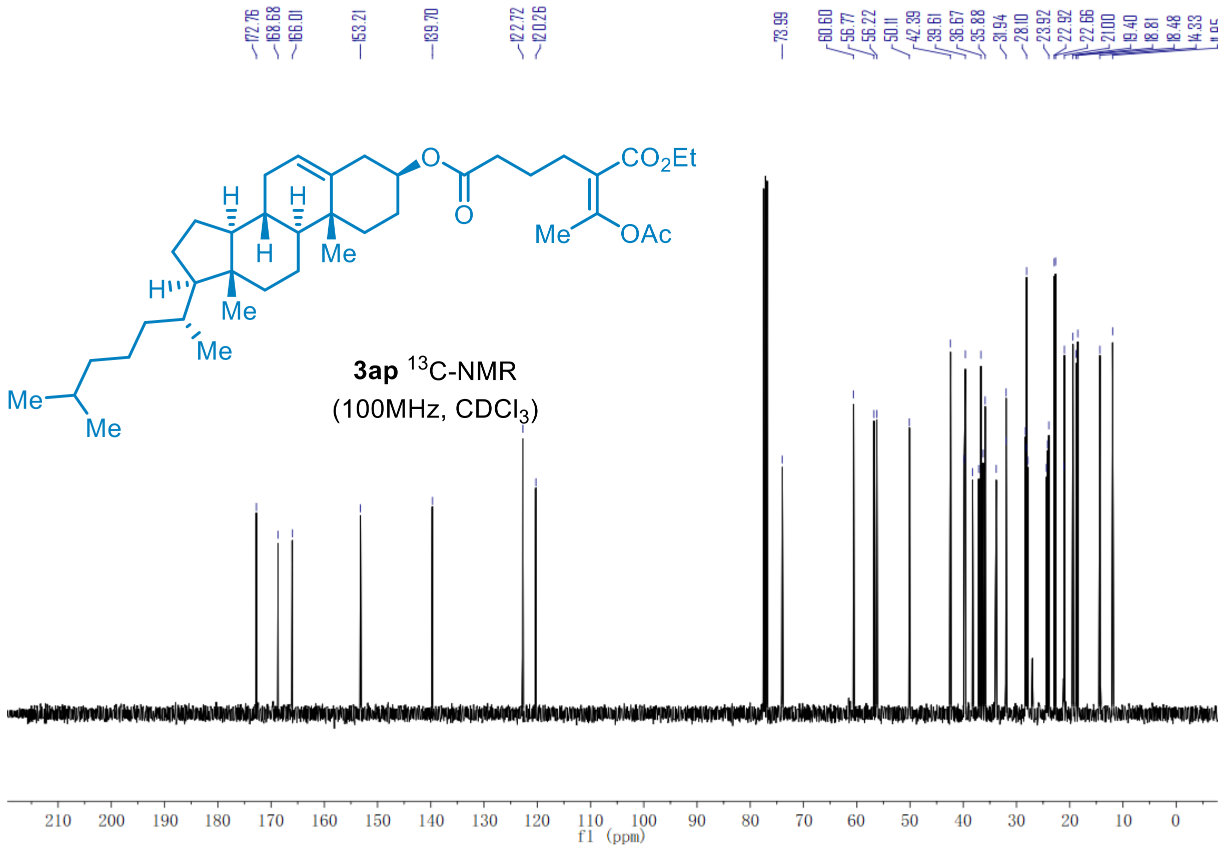

Supplementary Figure 77. **^{13}C-NMR** (100 MHz, $CDCl_3$, 298K) of **3ap**

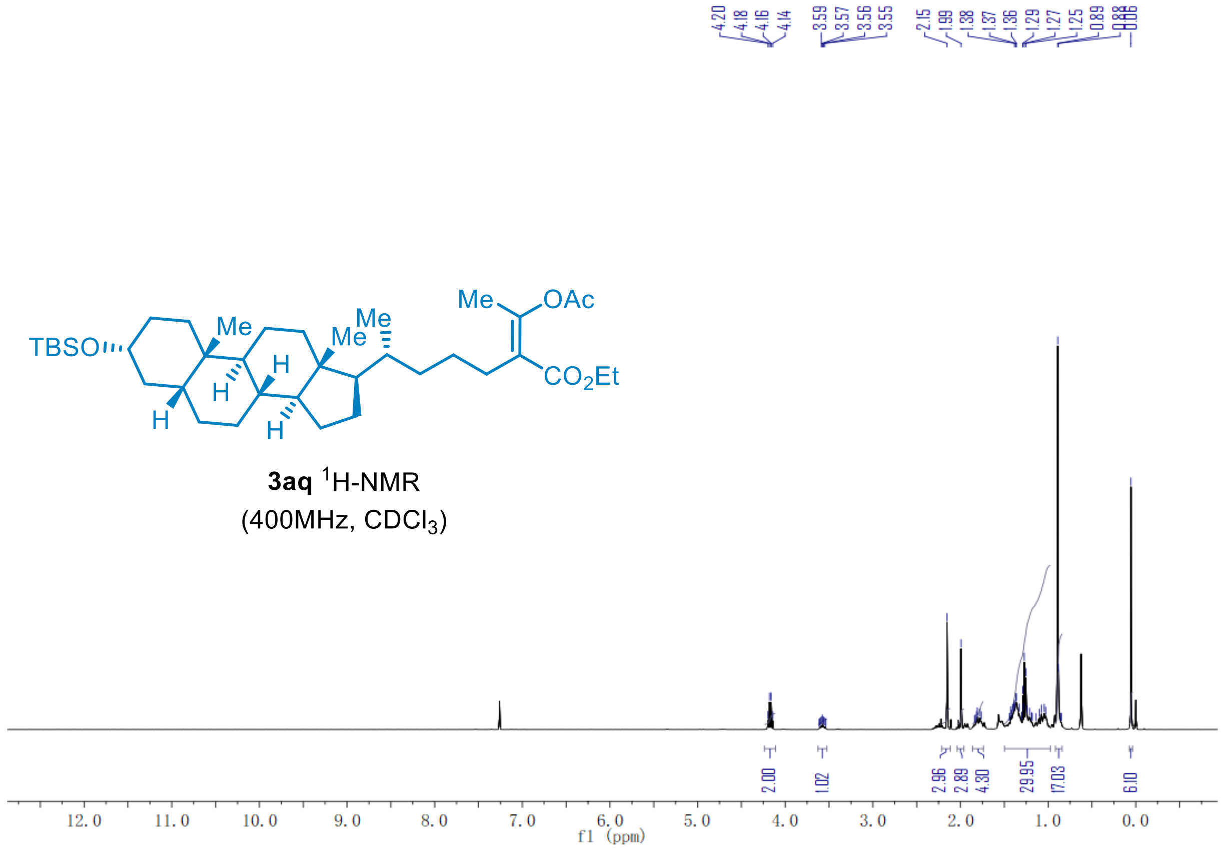

Supplementary Figure 78. **^{1}H-NMR** (400 MHz, $CDCl_3$, 298K) of **3aq**

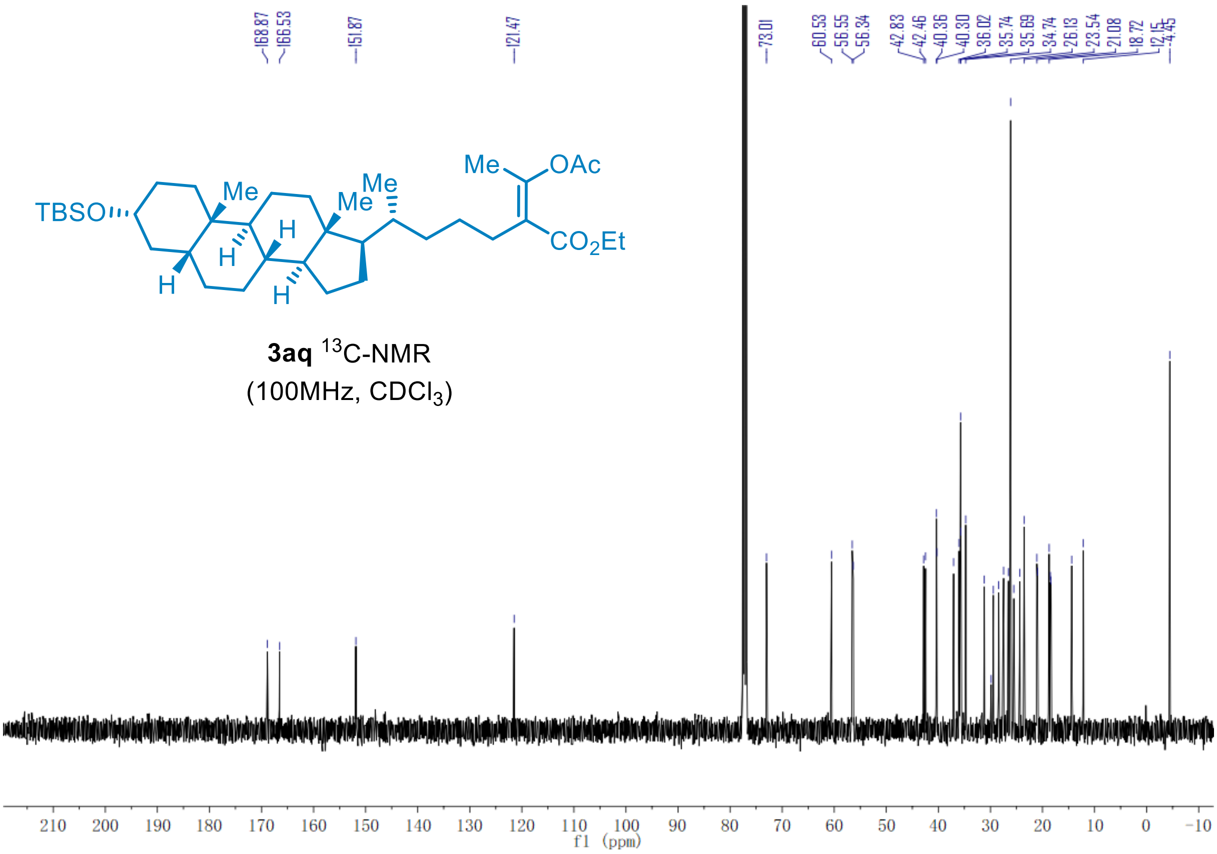

Supplementary Figure 79. **^{13}C-NMR** (100 MHz, $CDCl_3$, 298K) of **3aq**

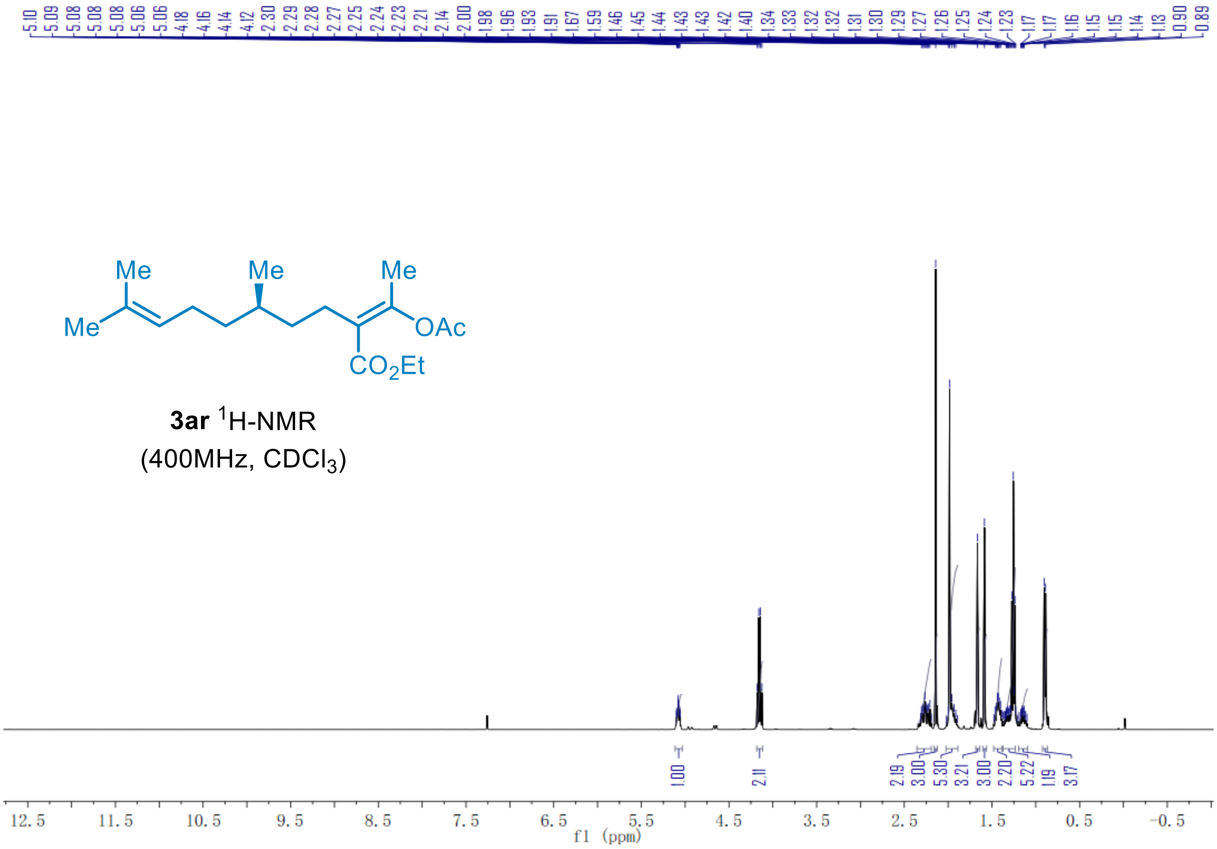

Supplementary Figure 80. **^{1}H-NMR** (400 MHz, $CDCl_3$, 298K) of **3ar**

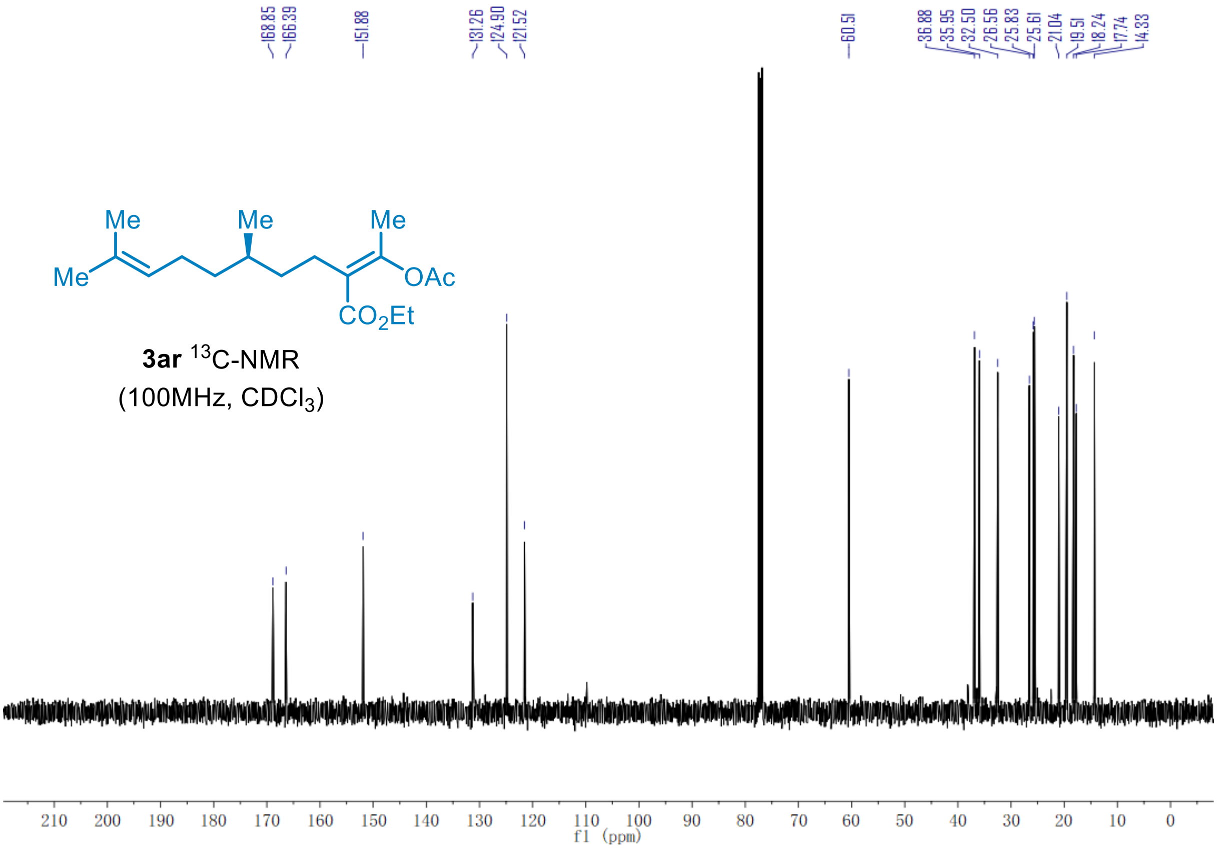

Supplementary Figure 81. **^{13}C-NMR** (100 MHz, $CDCl_3$, 298K) of **3ar**

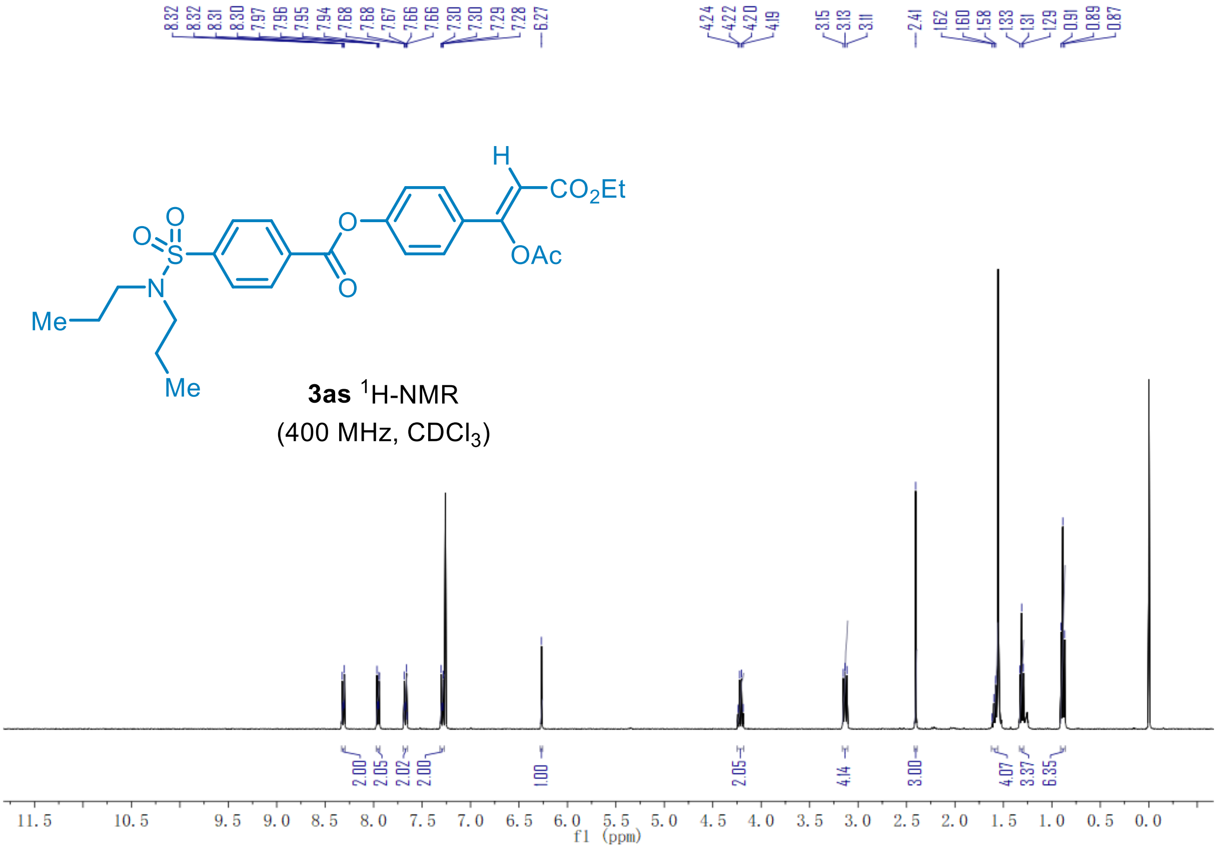

Supplementary Figure 82. **^{1}H-NMR** (400 MHz, $CDCl_3$, 298K) of **3as**

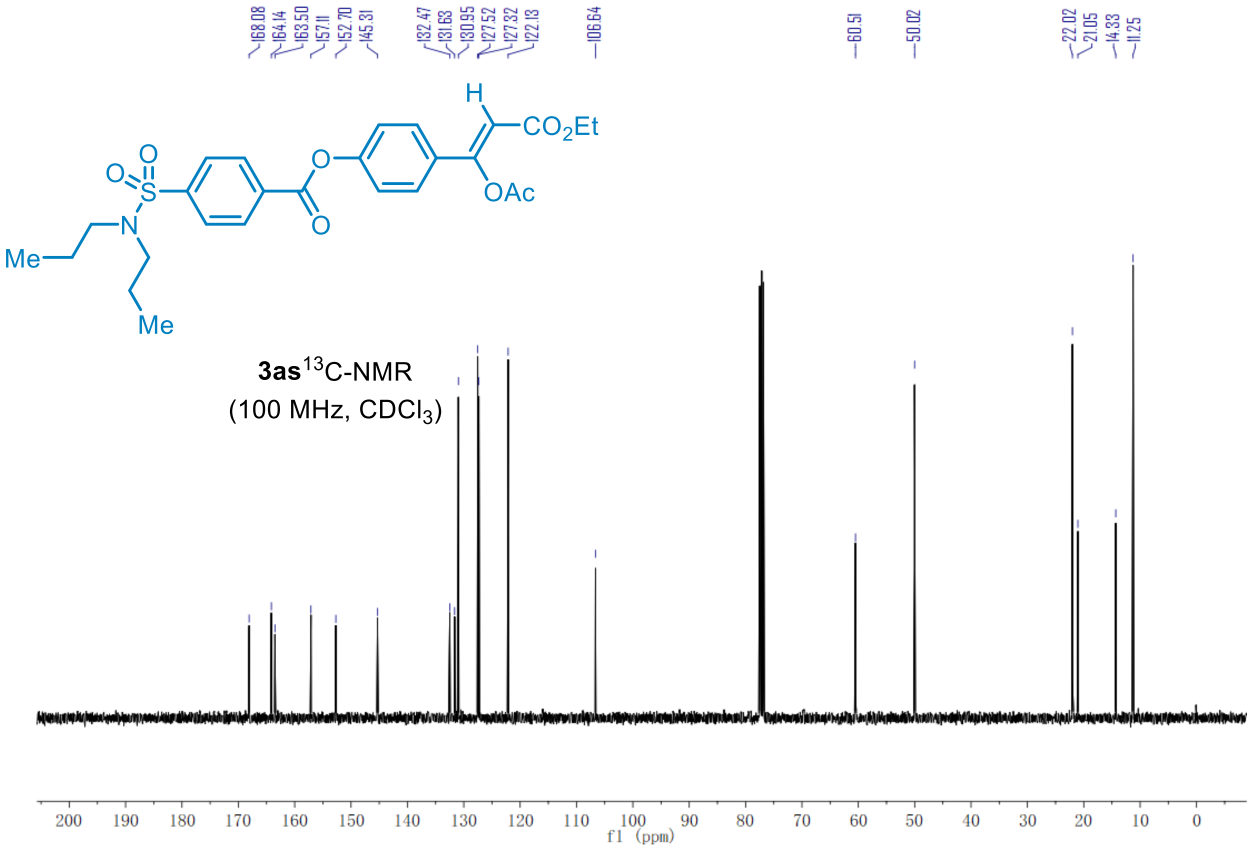

Supplementary Figure 83. **^{13}C-NMR** (100 MHz, $CDCl_3$, 298K) of **3as**

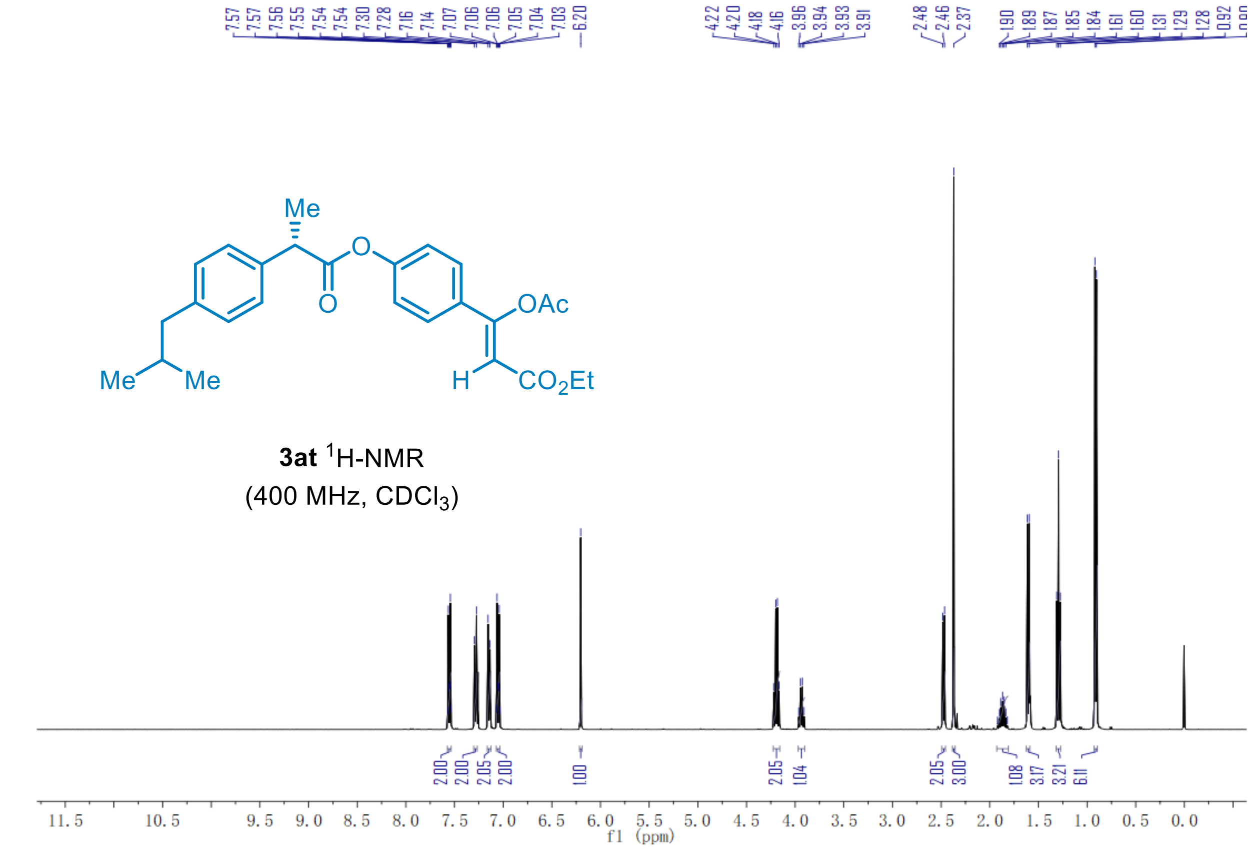

Supplementary Figure 84. **^{1}H-NMR** (400 MHz, $CDCl_3$, 298K) of **3at**

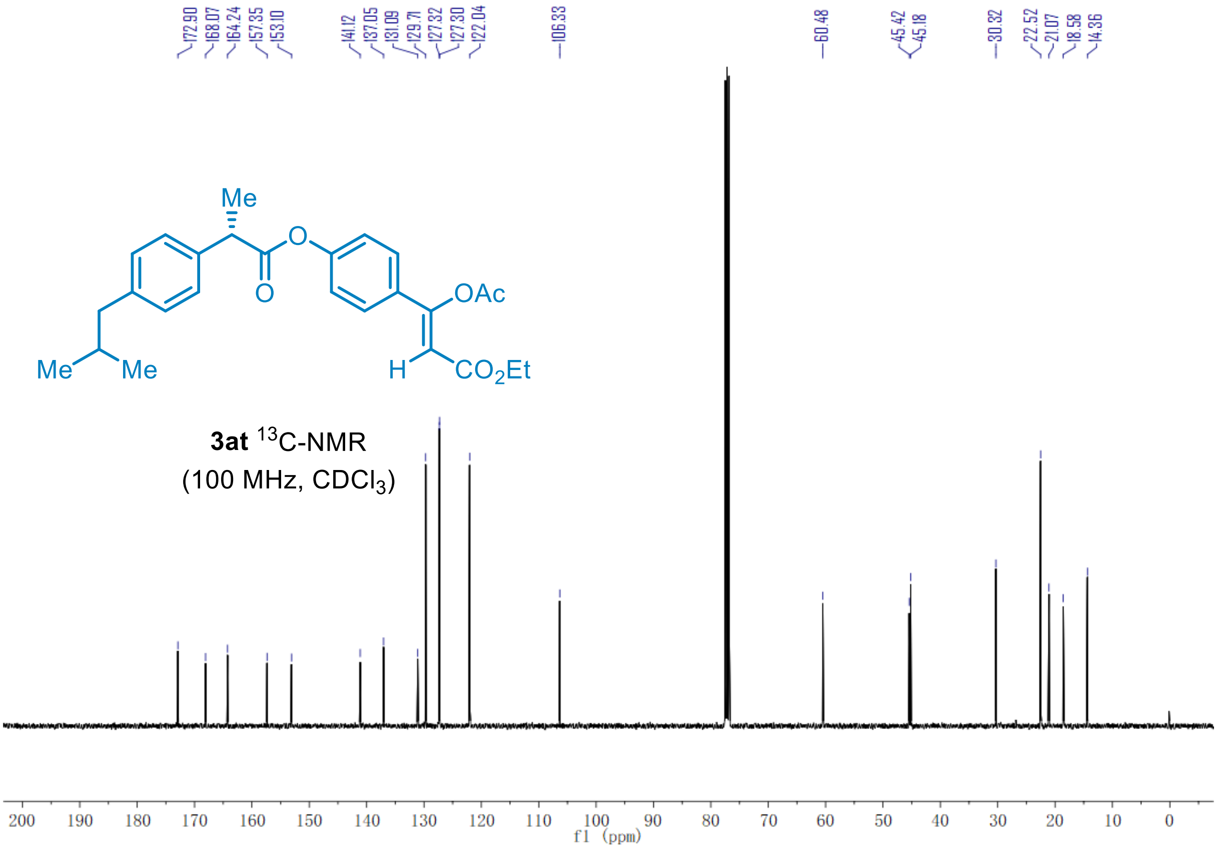

Supplementary Figure 85. **^{13}C-NMR** (100 MHz, $CDCl_3$, 298K) of **3at**

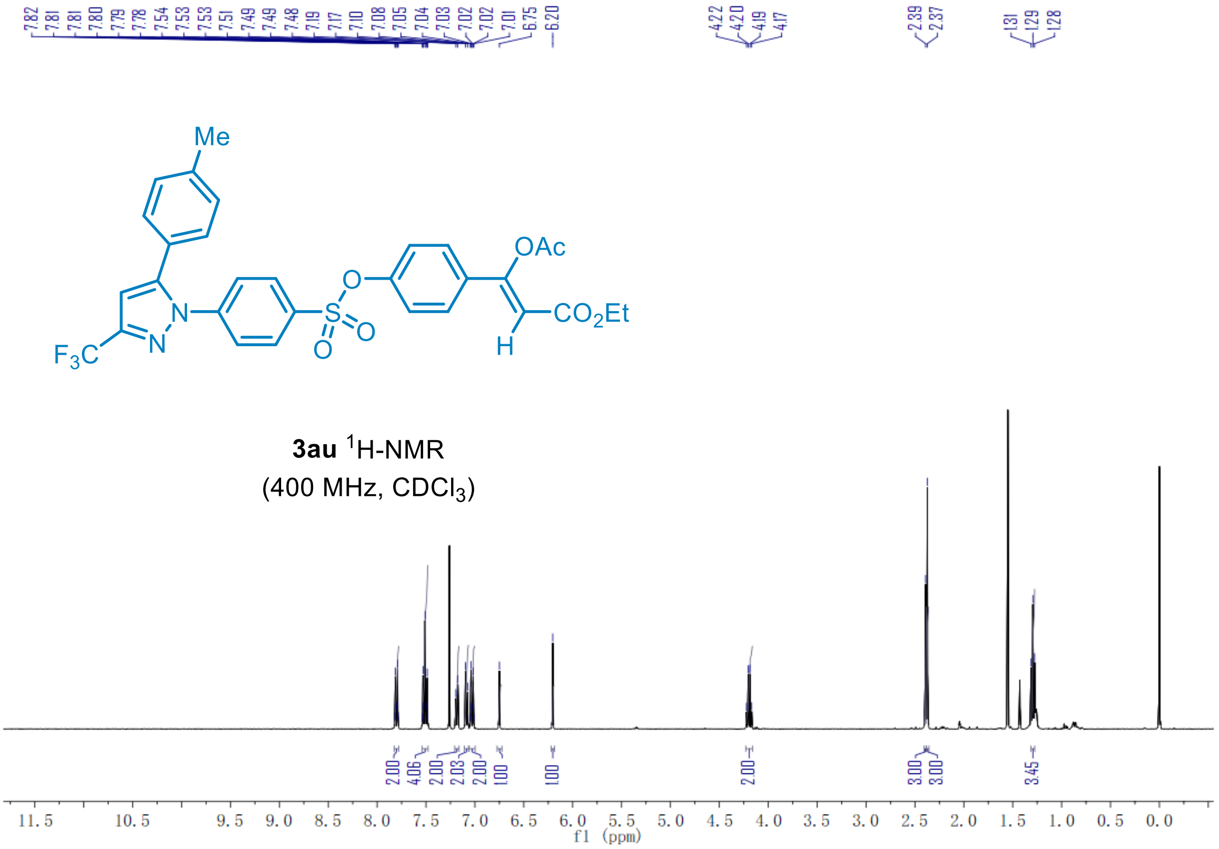

Supplementary Figure 86. **^{1}H-NMR** (400 MHz, $CDCl_3$, 298K) of **3au**

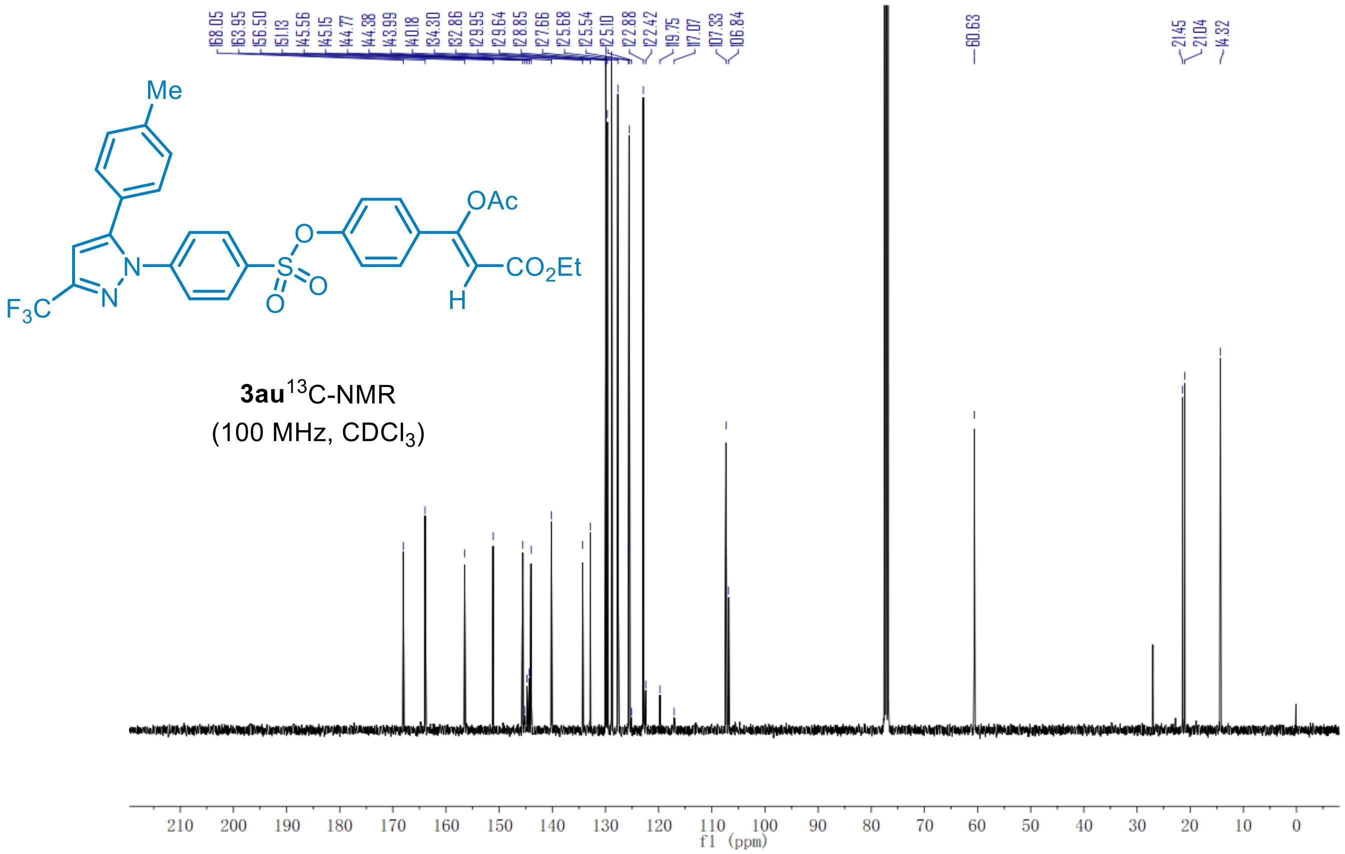

Supplementary Figure 87. **^{13}C-NMR** (100 MHz, $CDCl_3$, 298K) of **3au**

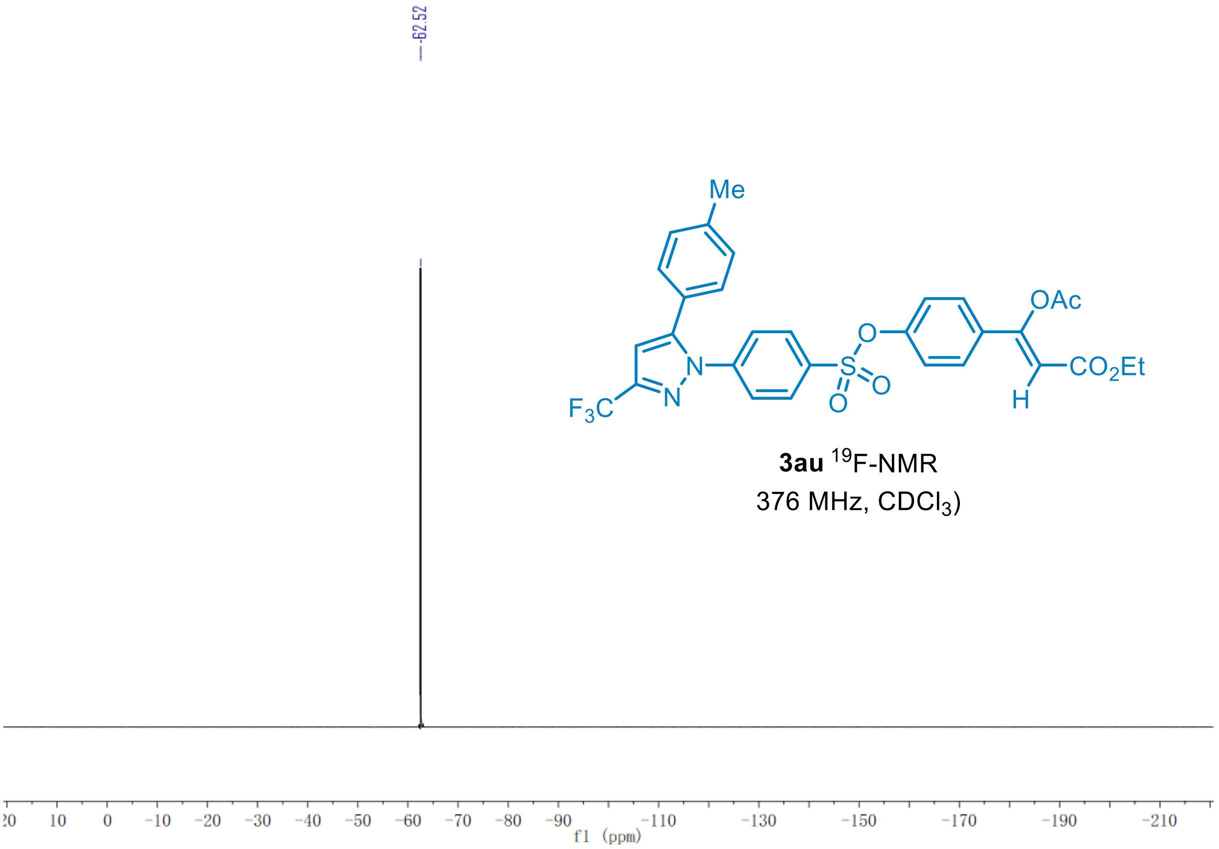

Supplementary Figure 88. **^{19}F-NMR** (376 MHz, $CDCl_3$, 298K) of **3au**

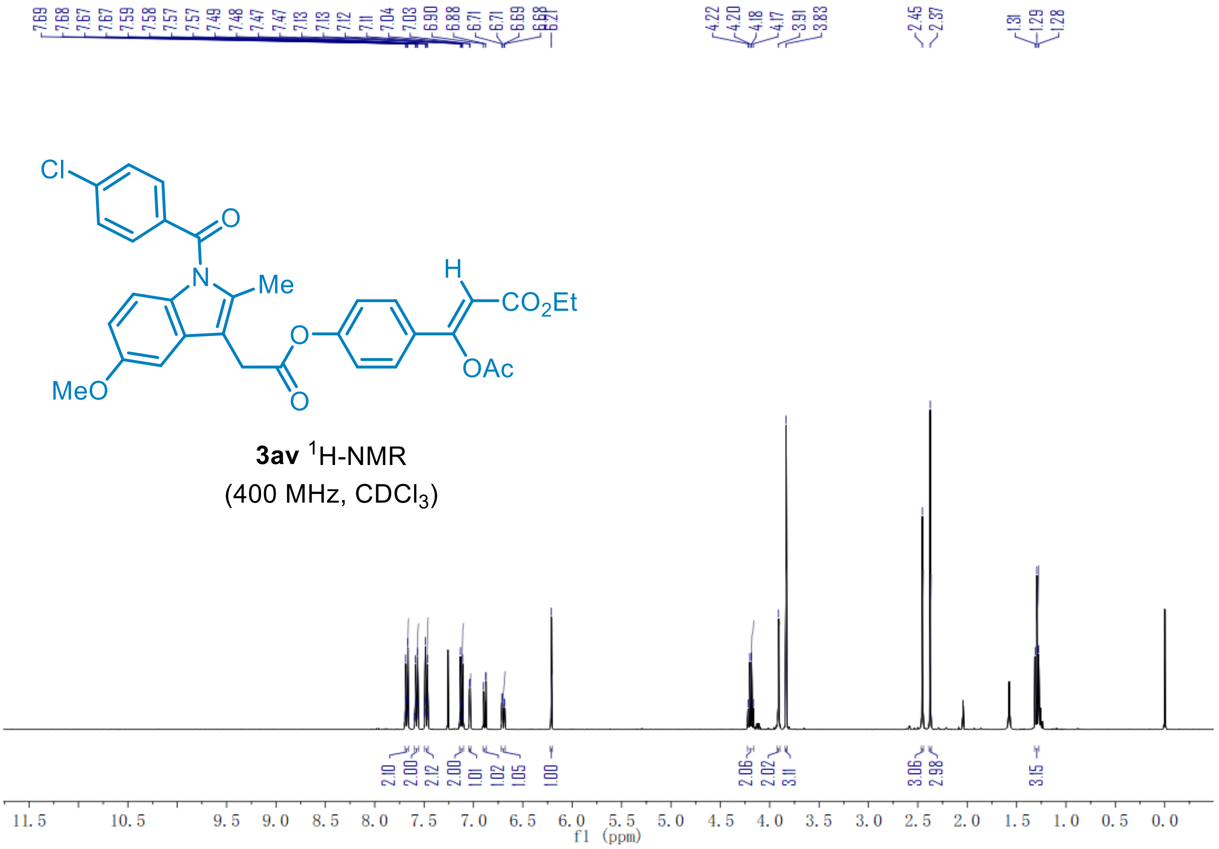

Supplementary Figure 89. **^{1}H-NMR** (400 MHz, $CDCl_3$, 298K) of **3av**

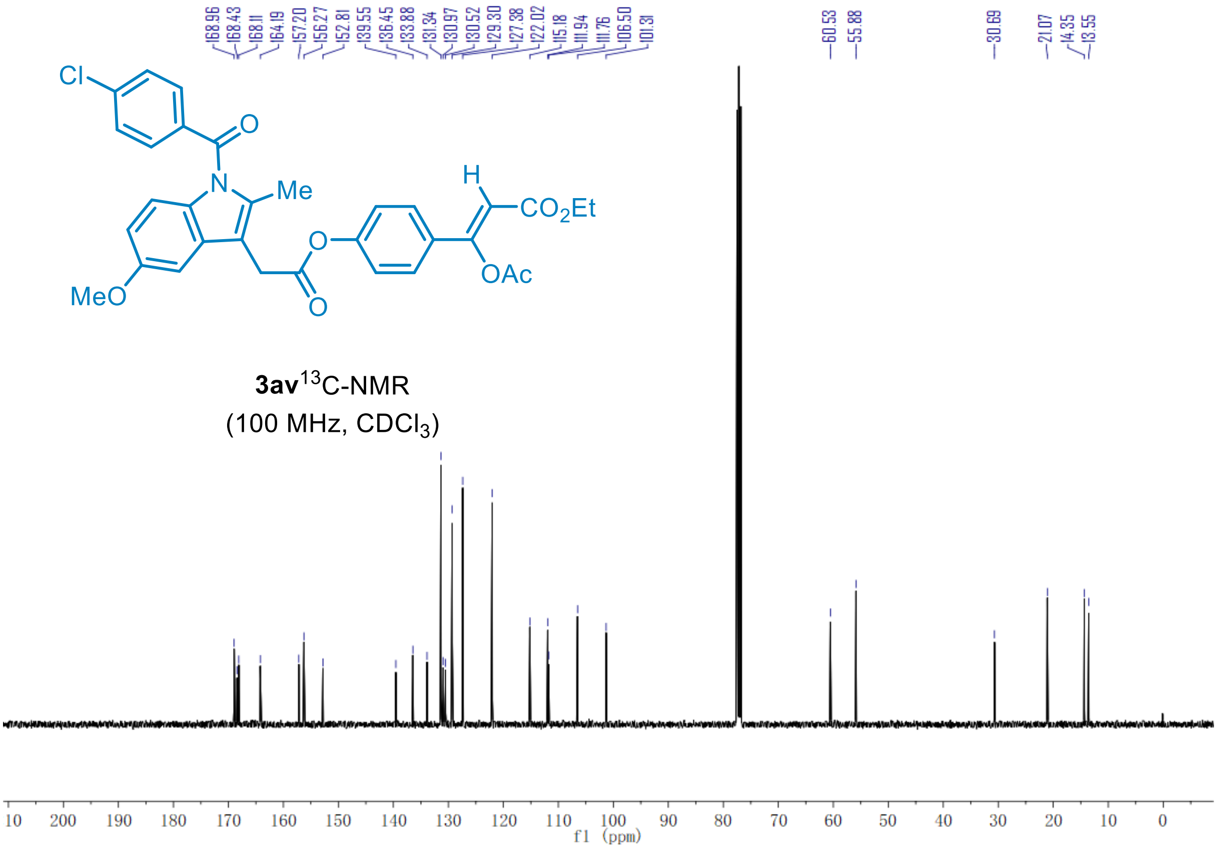

Supplementary Figure 90. **^{13}C-NMR** (100 MHz, $CDCl_3$, 298K) of **3av**

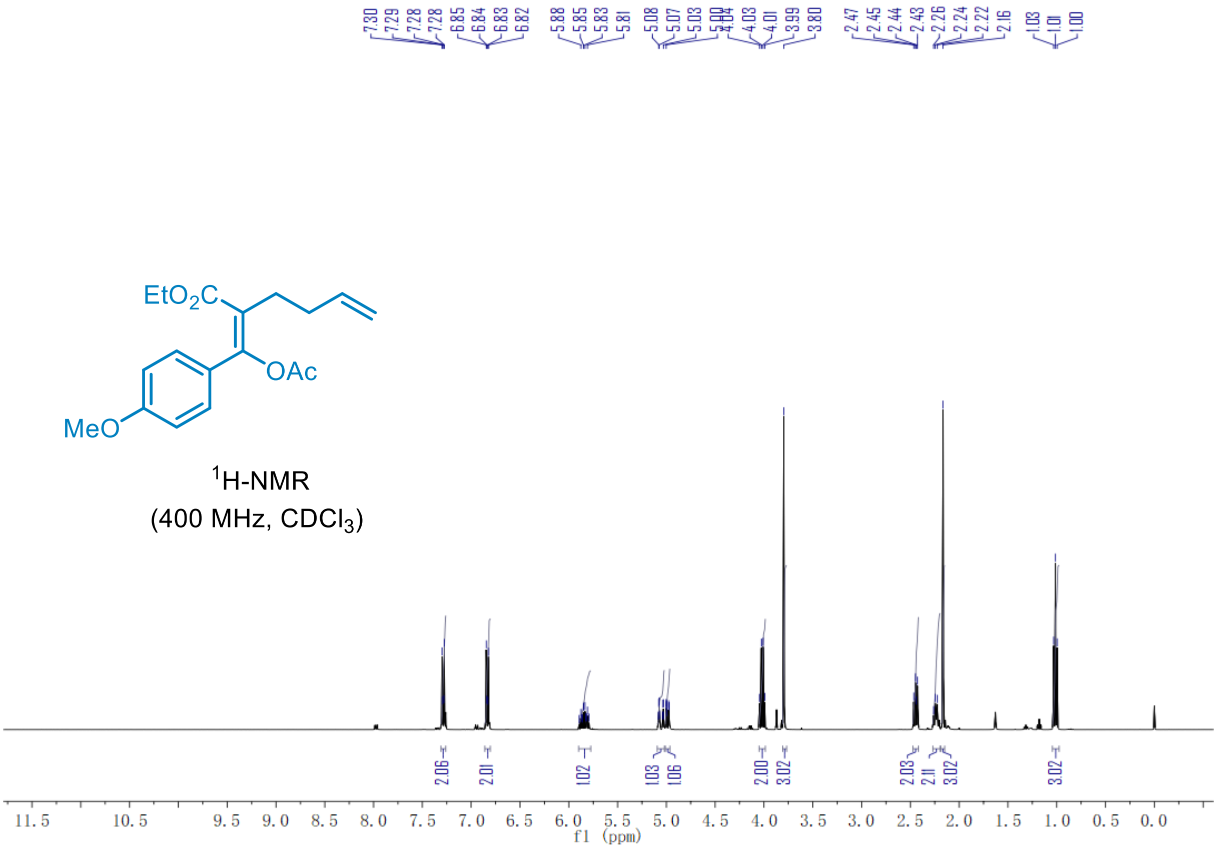

Supplementary Figure 91. **^{1}H-NMR** (400 MHz, $CDCl_3$, 298K)

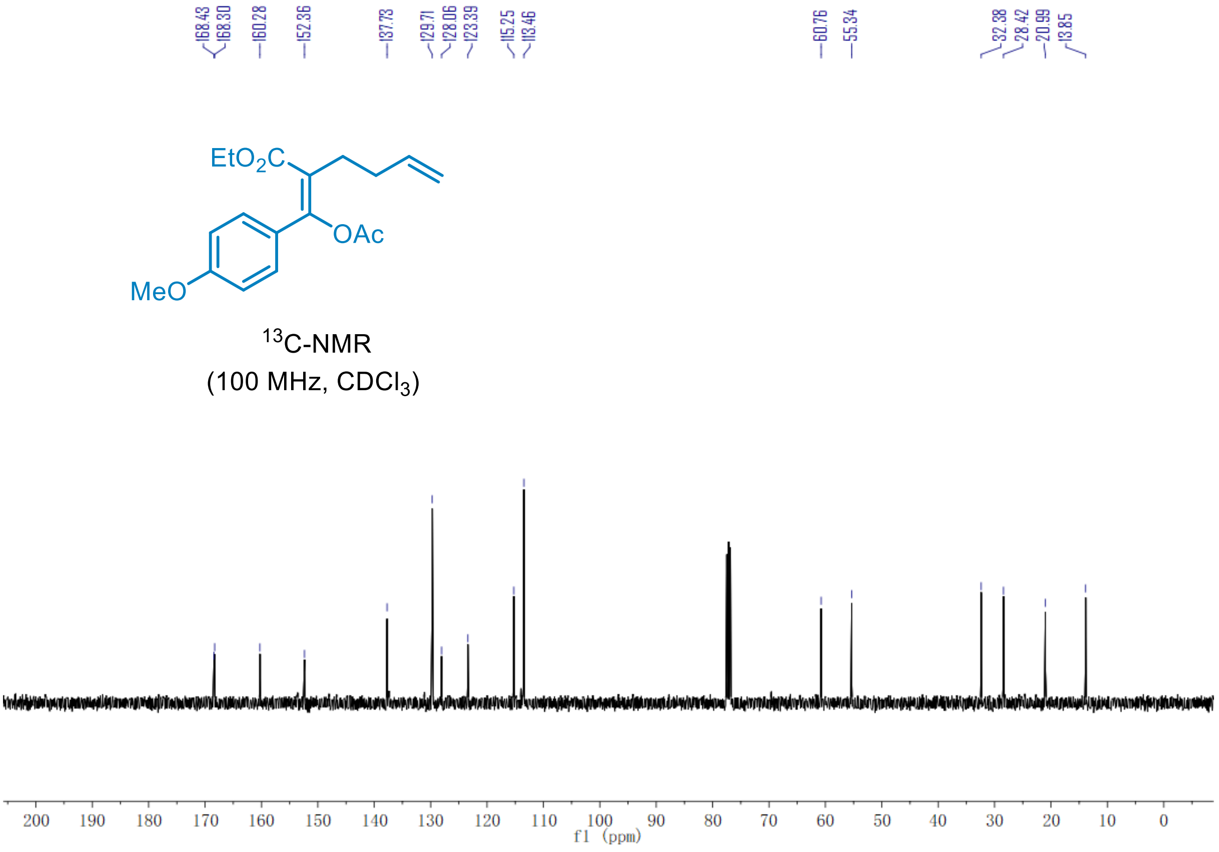

Supplementary Figure 92. **^{13}C-NMR** (100 MHz, $CDCl_3$, 298K)

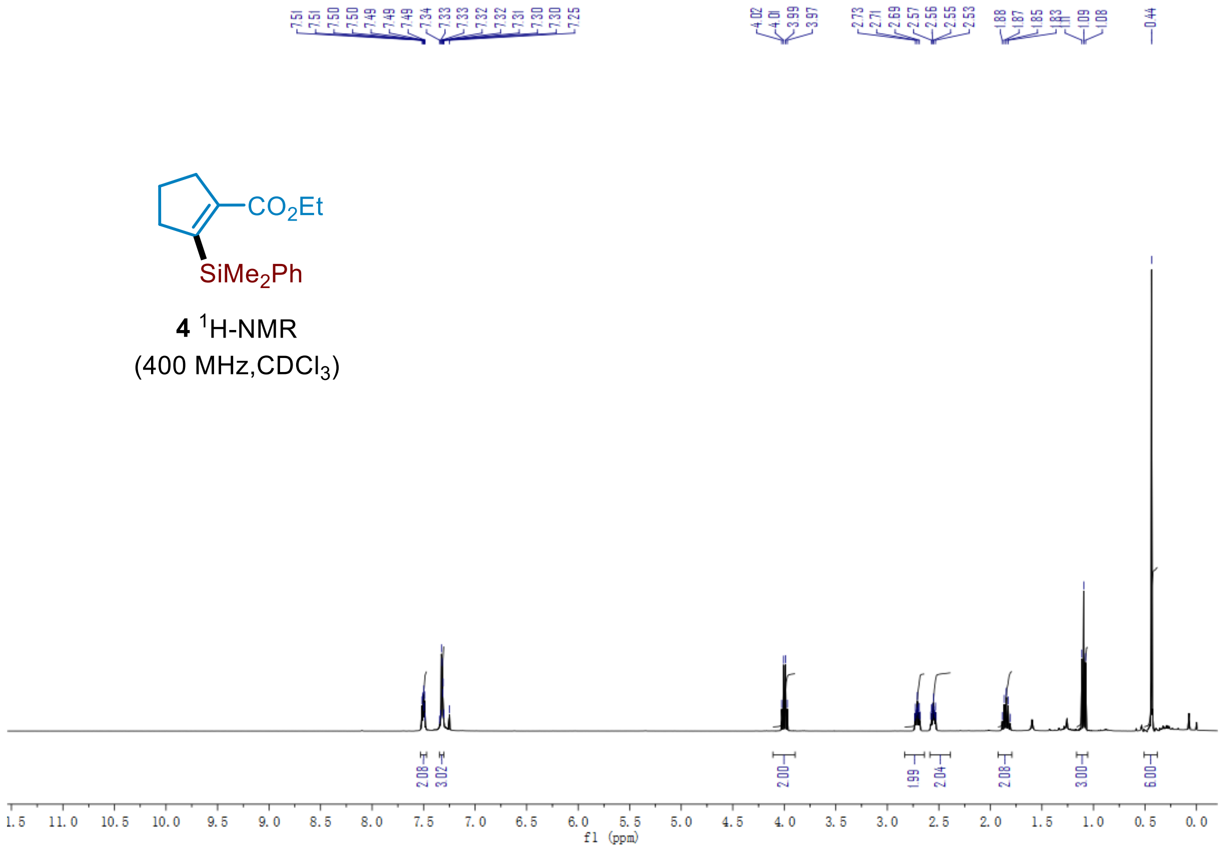

Supplementary Figure 93. **^{1}H-NMR** (400 MHz, $CDCl_3$, 298K) of **4**

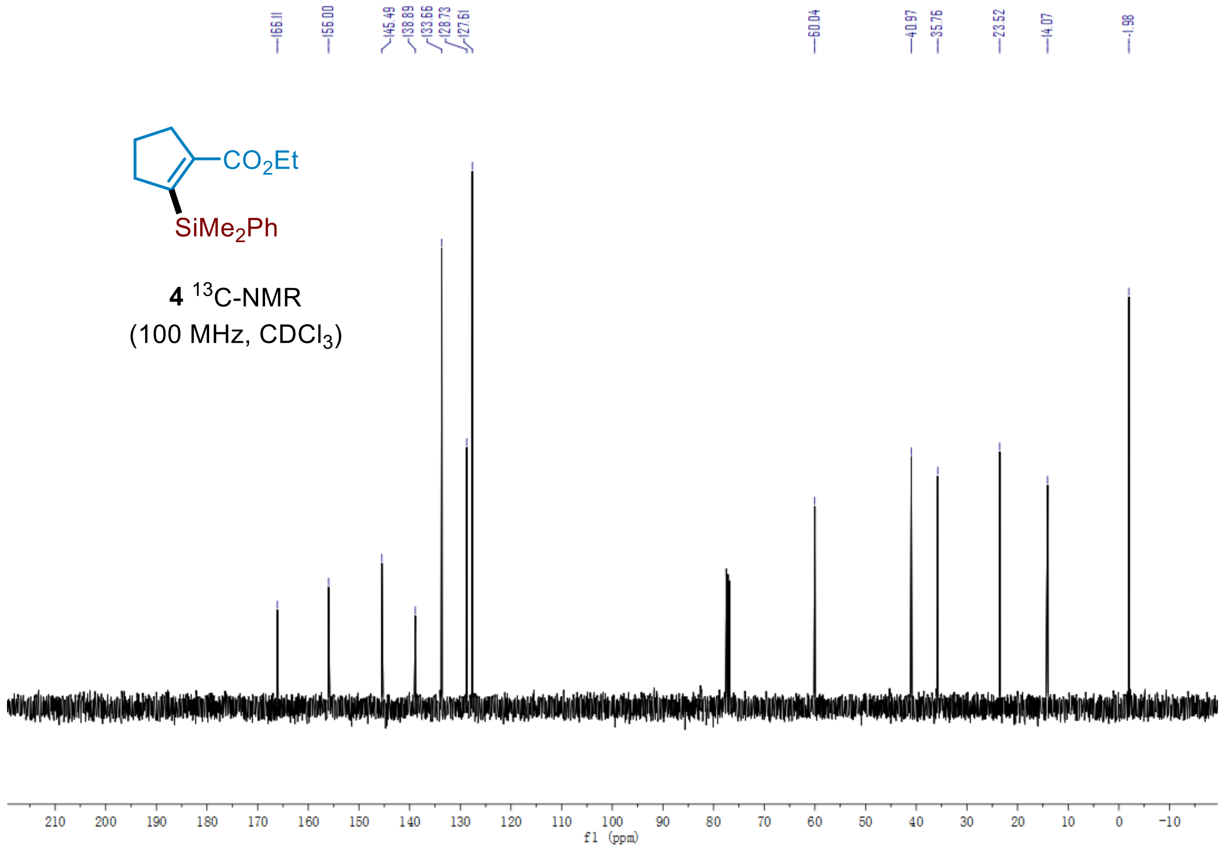

Supplementary Figure 94. **^{13}C-NMR** (100 MHz, $CDCl_3$, 298K) of **4**

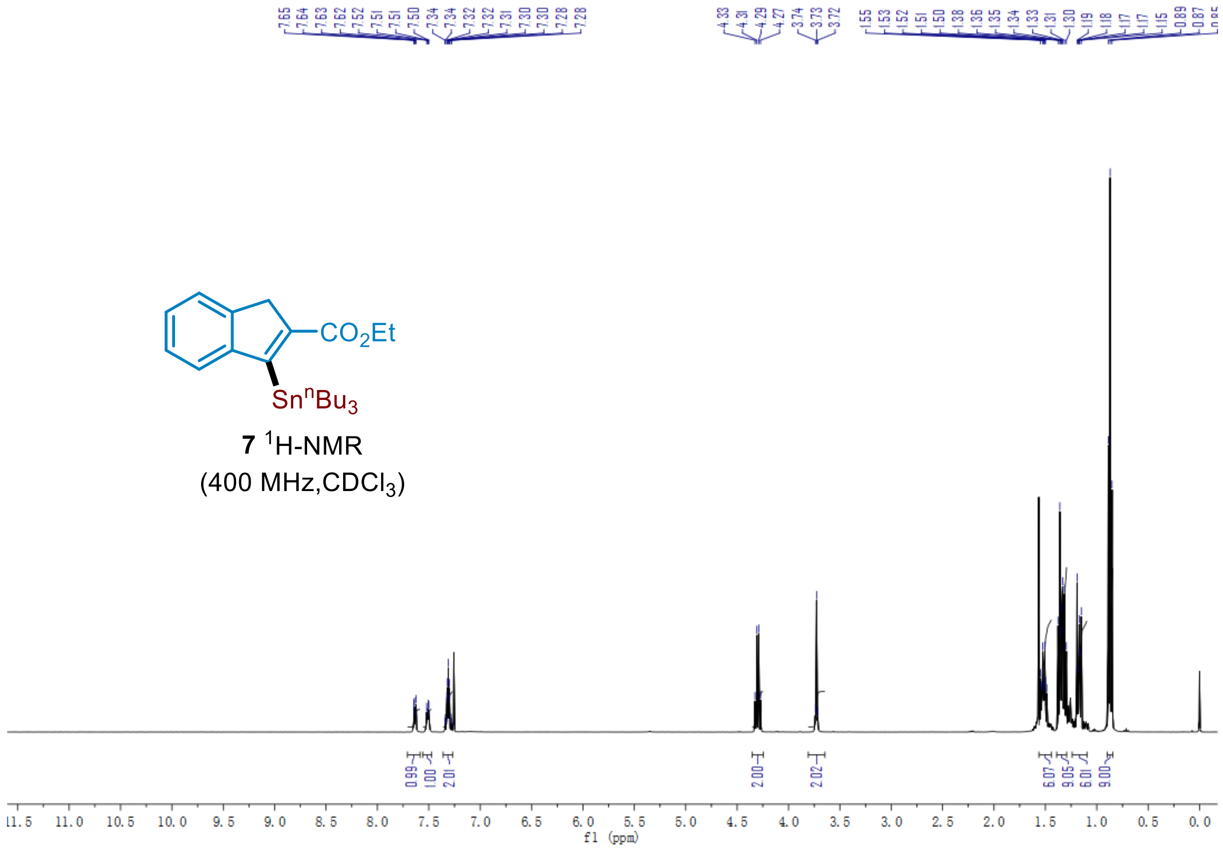

Supplementary Figure 95. **^{1}H-NMR** (400 MHz, $CDCl_3$, 298K) of **7**

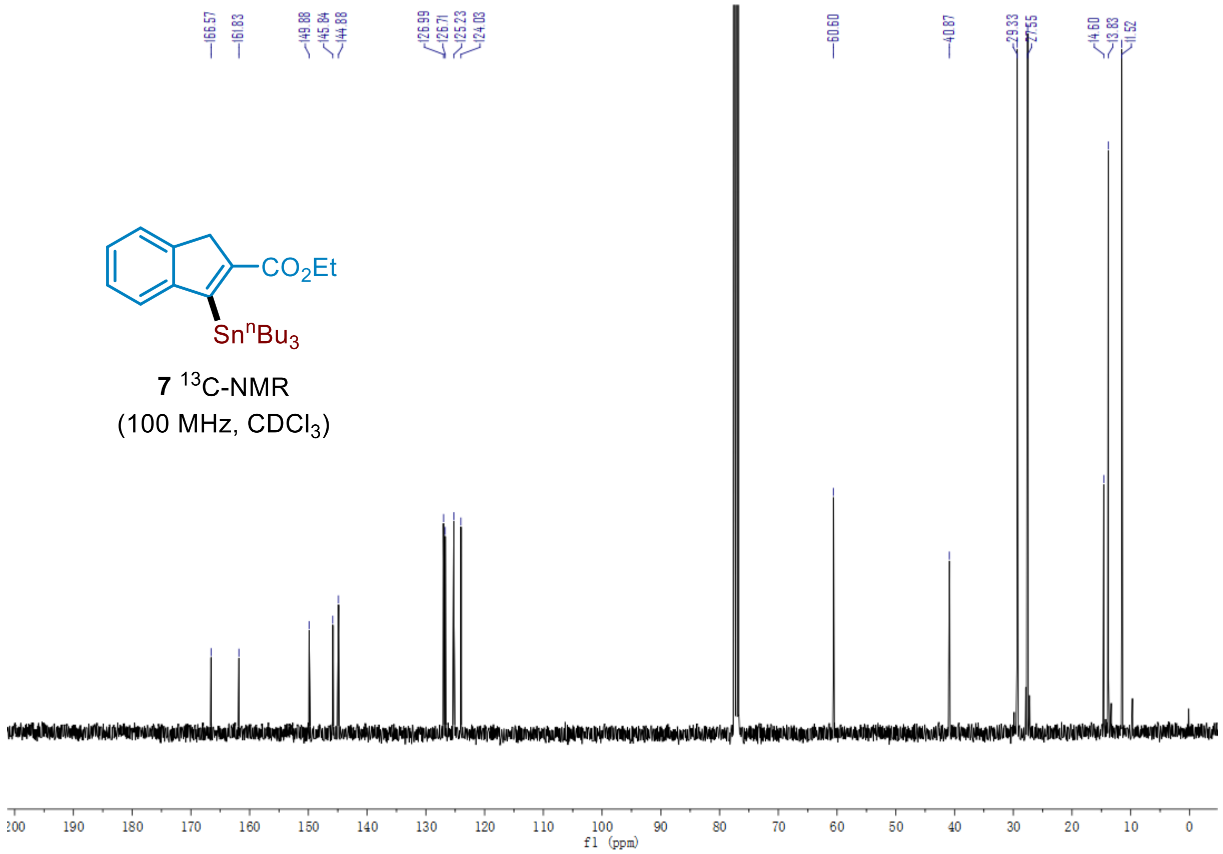

Supplementary Figure 96. **^{13}C-NMR** (100 MHz, $CDCl_3$, 298K) of **7**

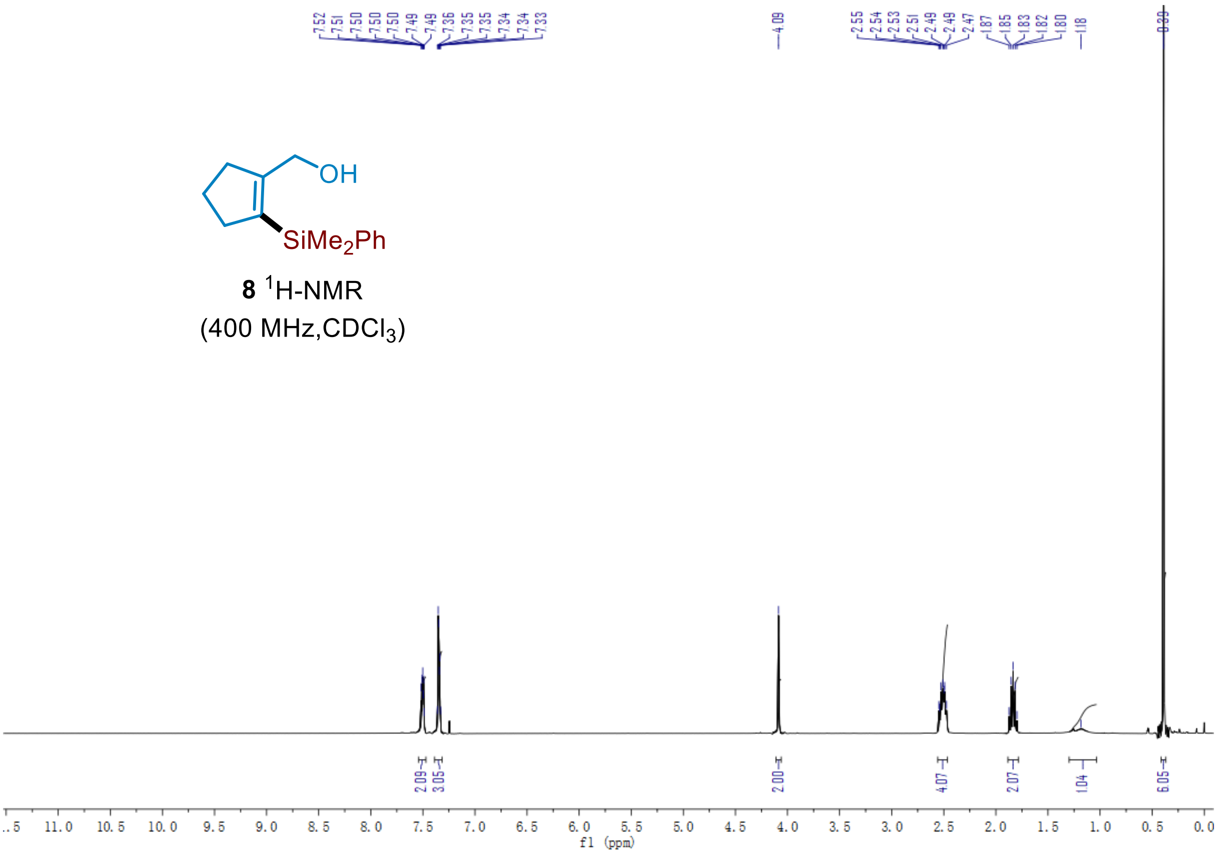

Supplementary Figure 97. **^{1}H-NMR** (400 MHz, $CDCl_3$, 298K) of **8**

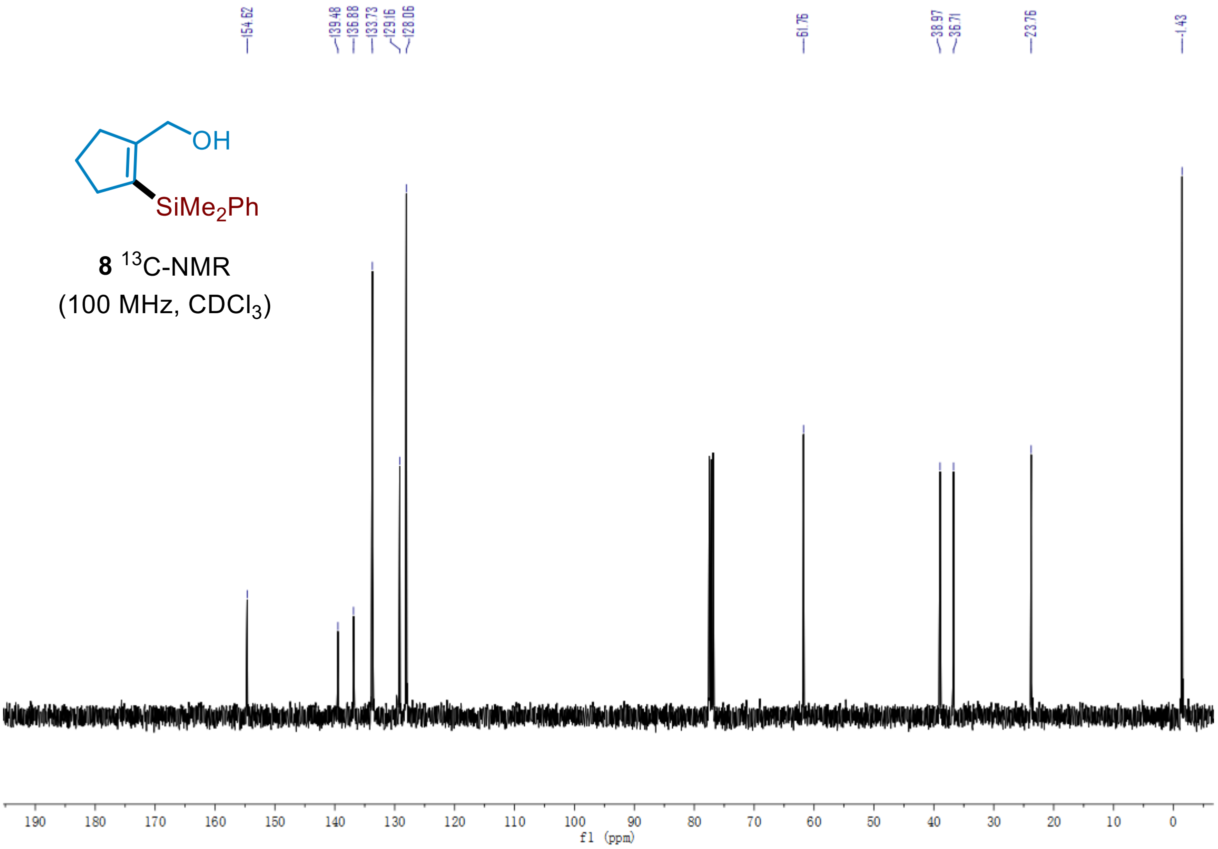

Supplementary Figure 98. **^{13}C-NMR** (100 MHz, $CDCl_3$, 298K) of **8**

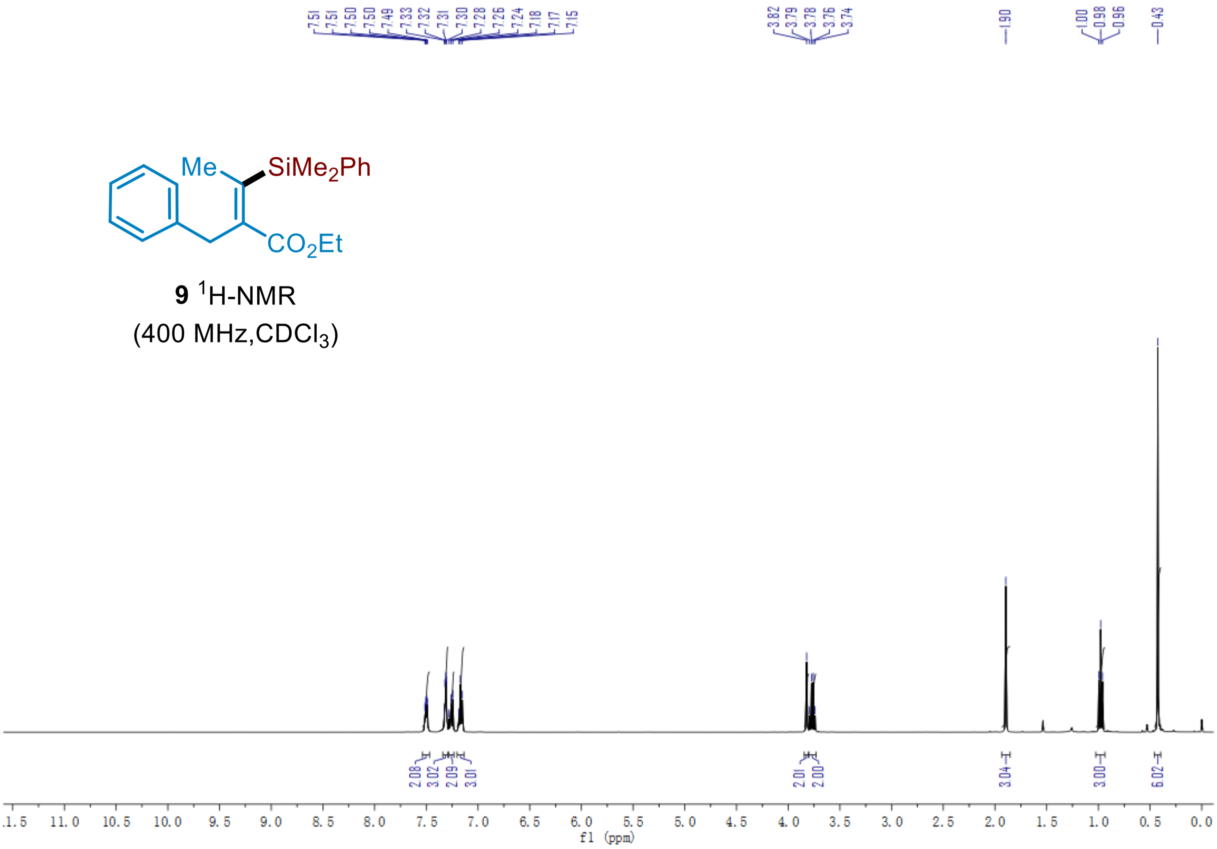

Supplementary Figure 99. **^{1}H-NMR** (400 MHz, $CDCl_3$, 298K) of **9**

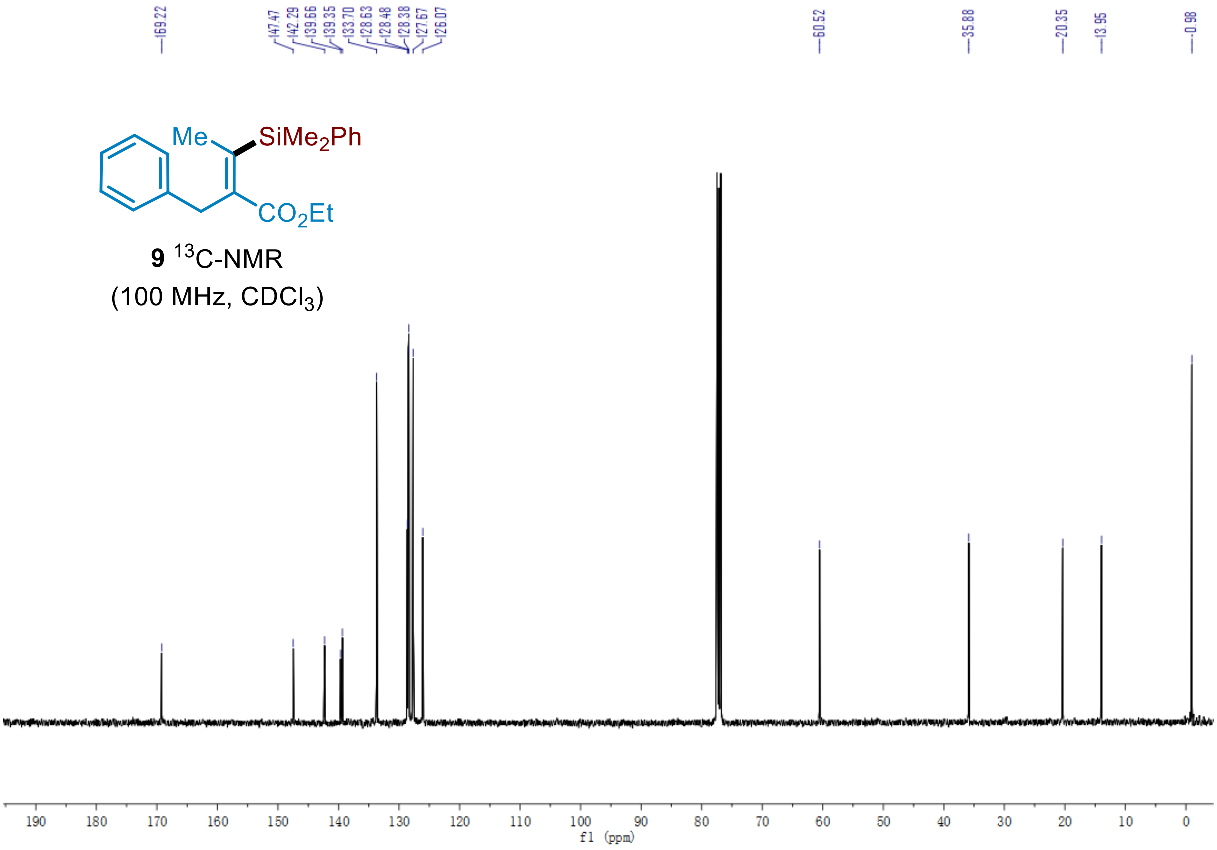

Supplementary Figure 100. **^{13}C-NMR** (100 MHz, $CDCl_3$, 298K) of **9**

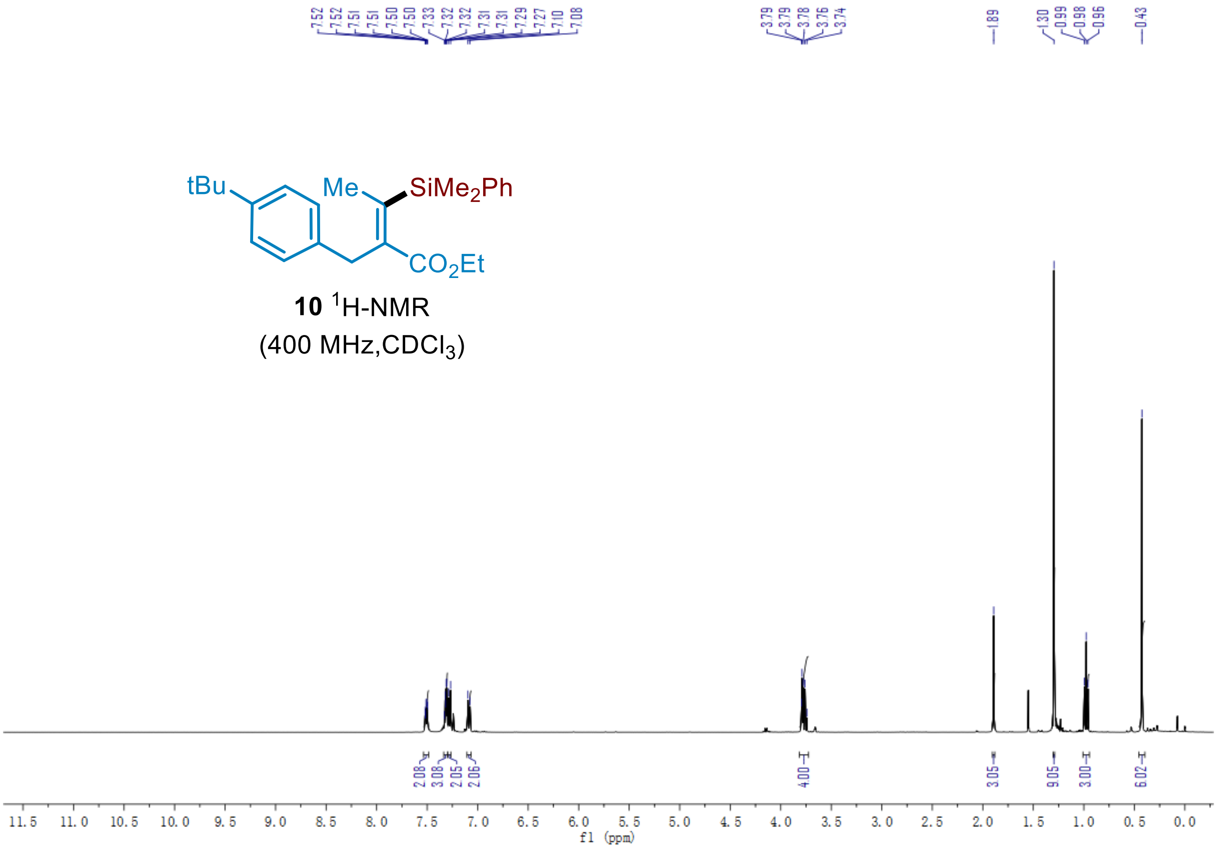

Supplementary Figure 101. **^{1}H-NMR** (400 MHz, $CDCl_3$, 298K) of **10**

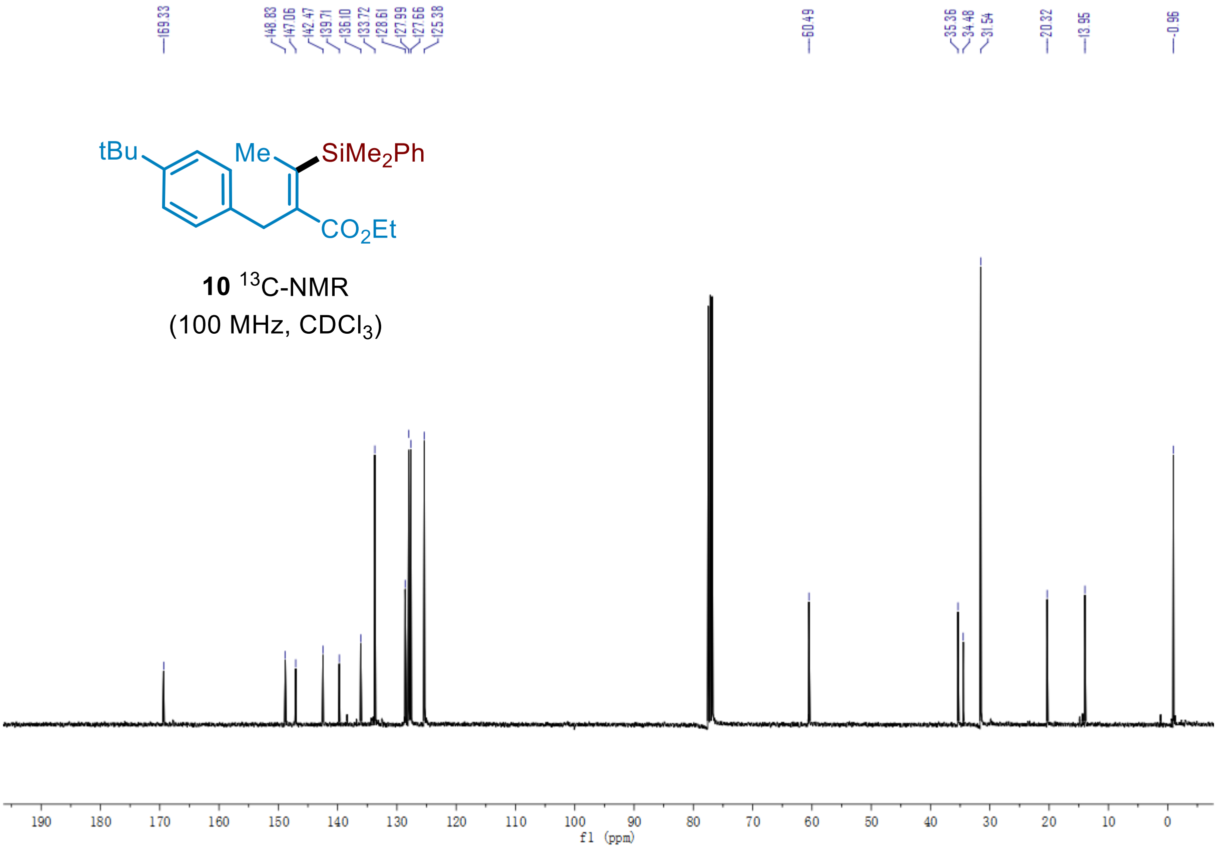

Supplementary Figure 102. **^{13}C-NMR** (100 MHz, $CDCl_3$, 298K) of **10**

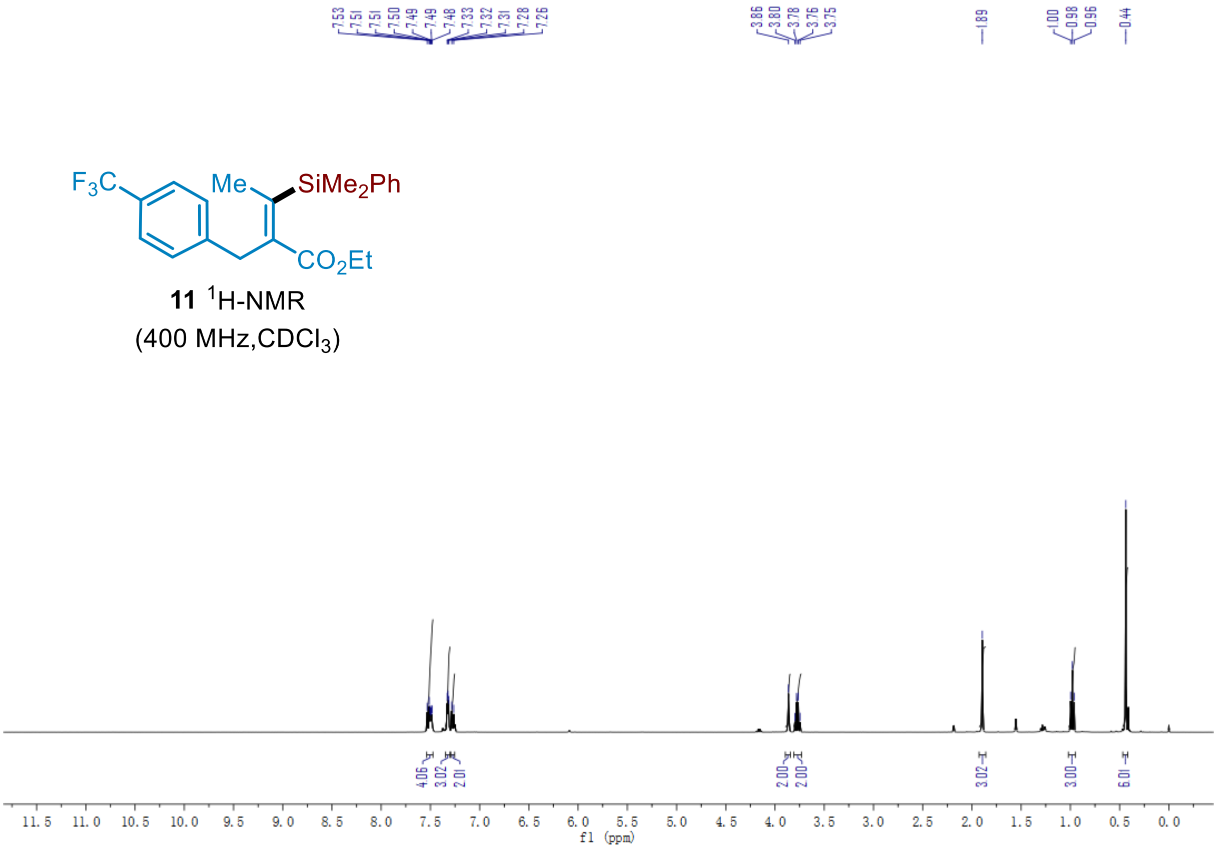

Supplementary Figure 103. **^{1}H-NMR** (400 MHz, $CDCl_3$, 298K) of **11**

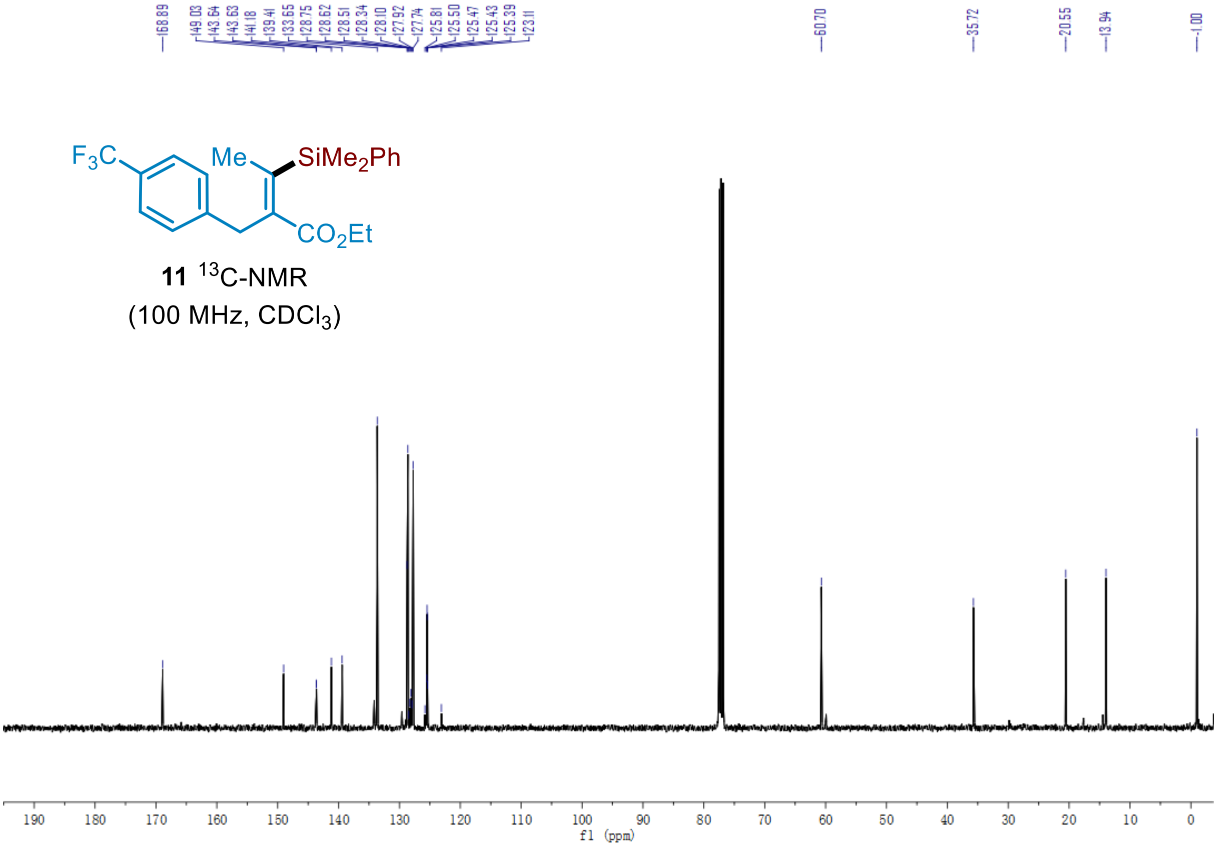

Supplementary Figure 104. **^{13}C-NMR** (100 MHz, $CDCl_3$, 298K) of **11**

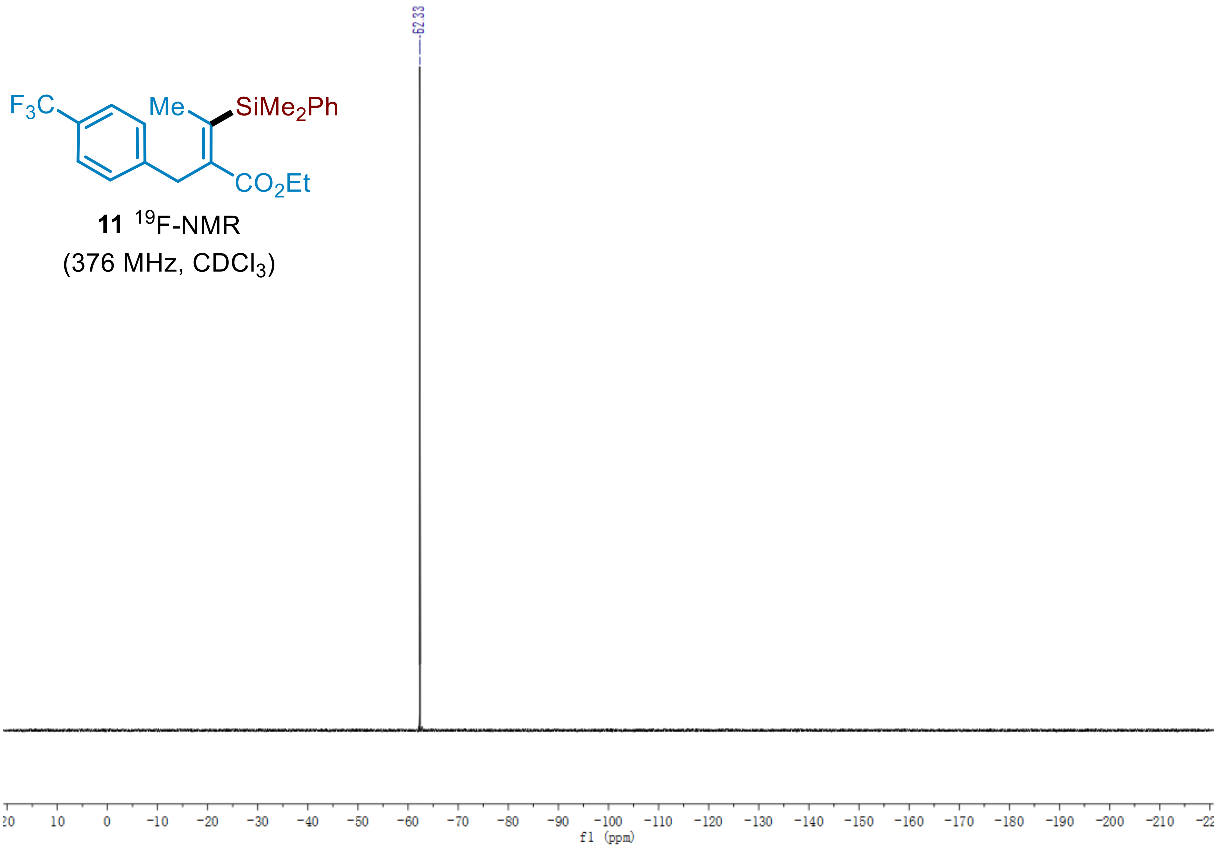

Supplementary Figure 105. **^{19}F-NMR** (376 MHz, $CDCl_3$, 298K) of **11**

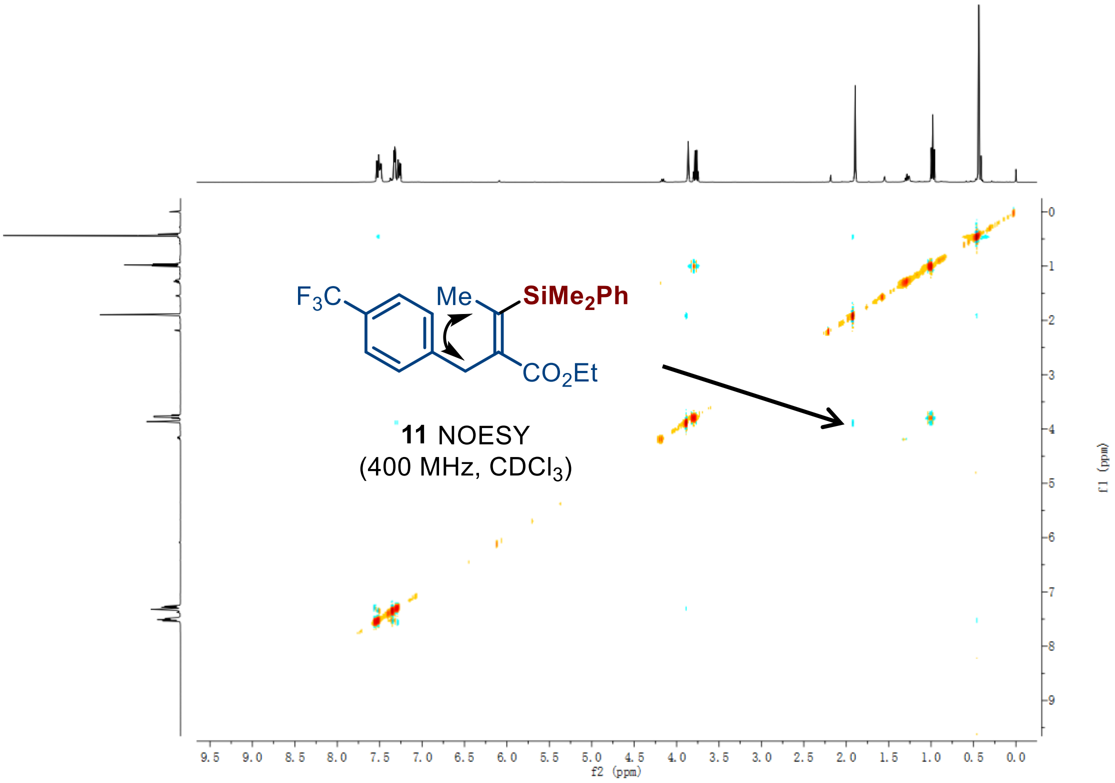

Supplementary Figure 106. NOESY of **11**

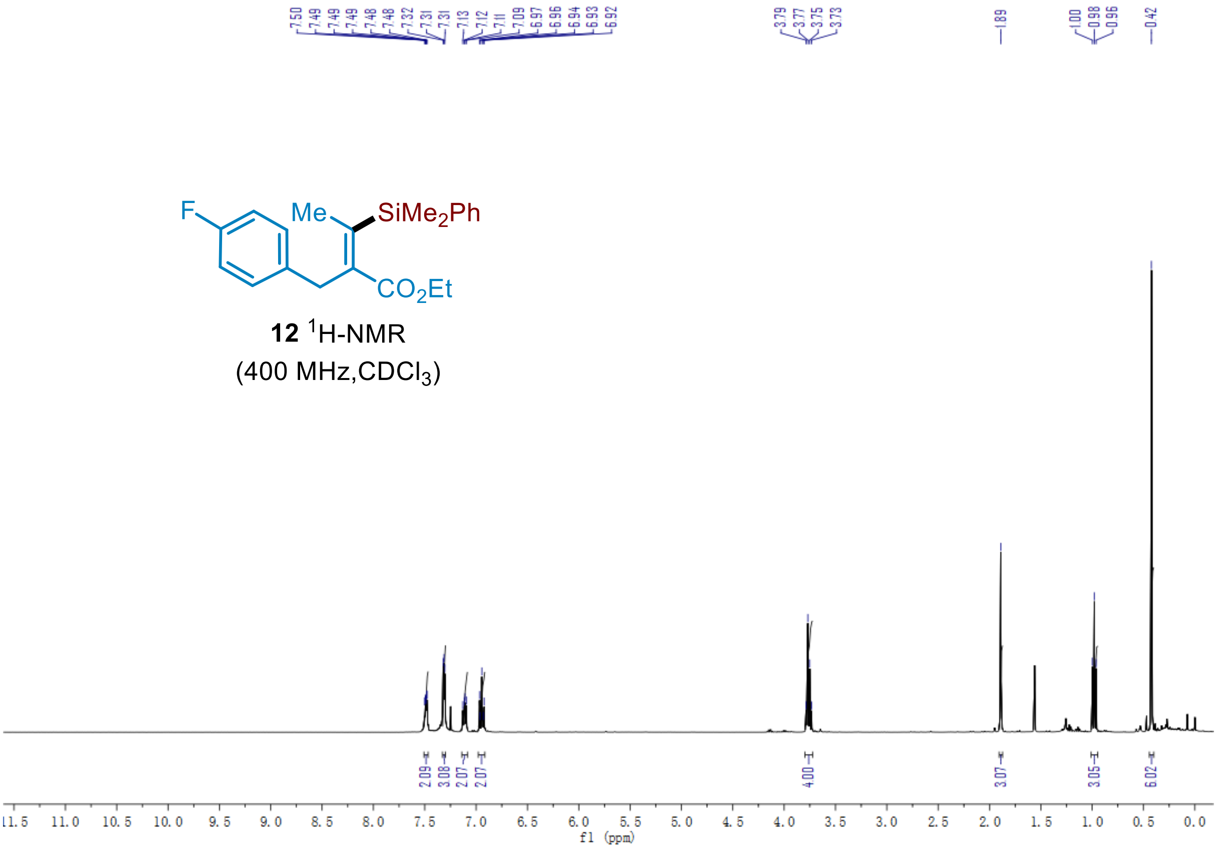

Supplementary Figure 107. **^{1}H-NMR** (400 MHz, $CDCl_3$, 298K) of **12**

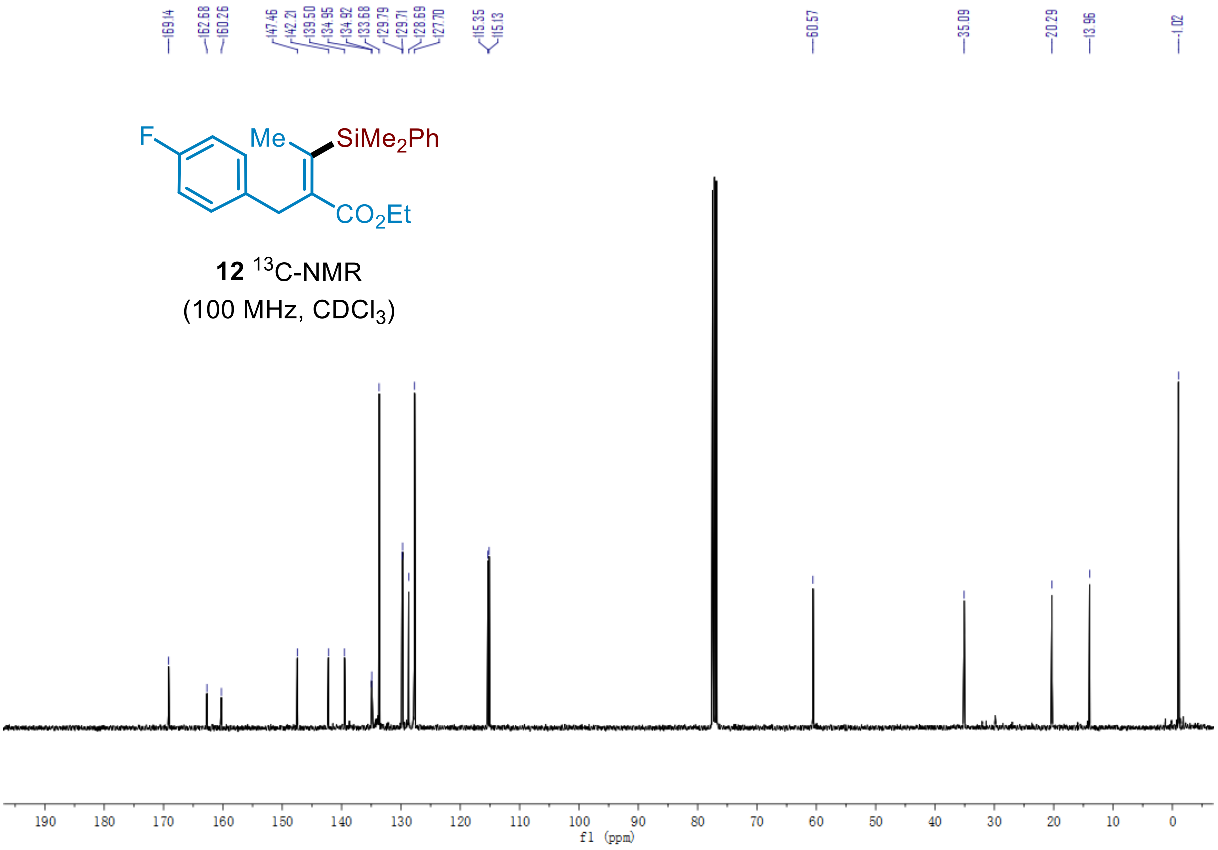

Supplementary Figure 108. **^{13}C-NMR** (100 MHz, $CDCl_3$, 298K) of **12**

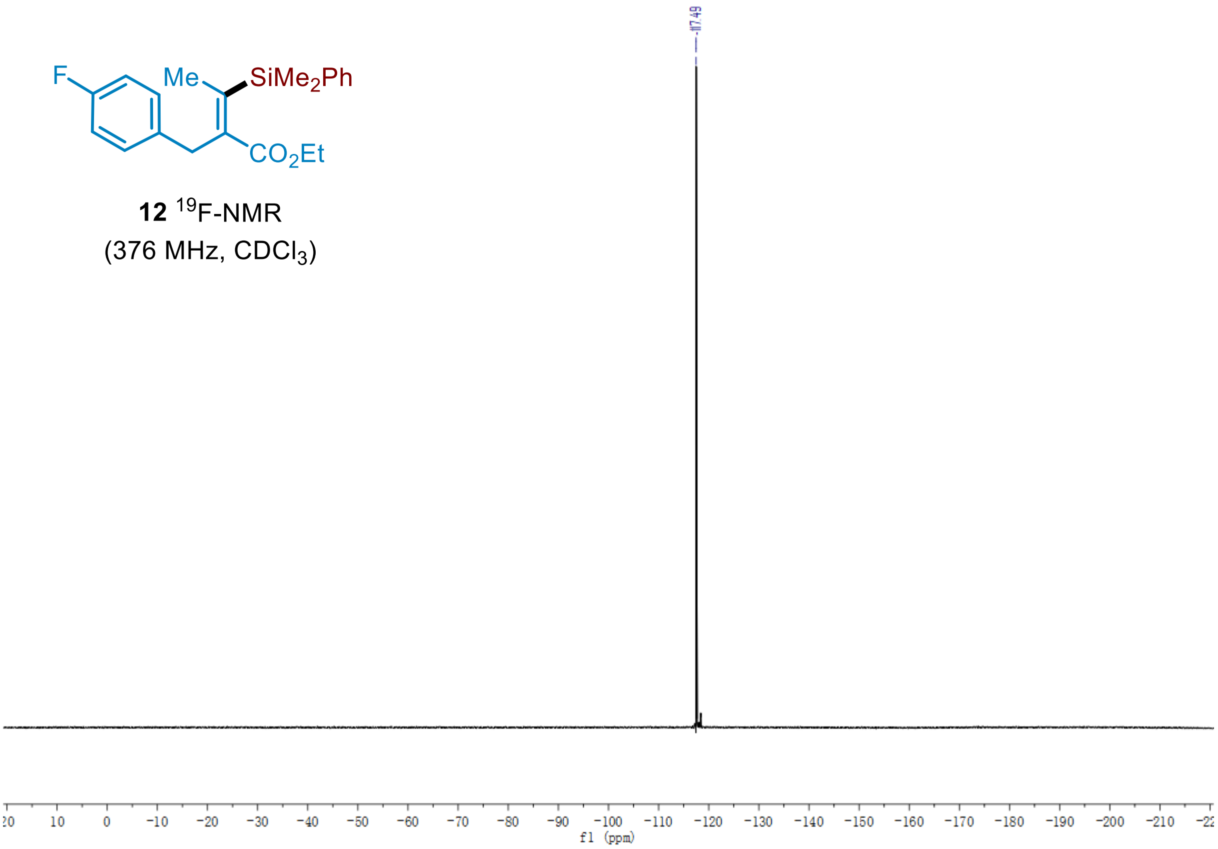

Supplementary Figure 109. **^{19}F-NMR** (376 MHz, $CDCl_3$, 298K) of **12**

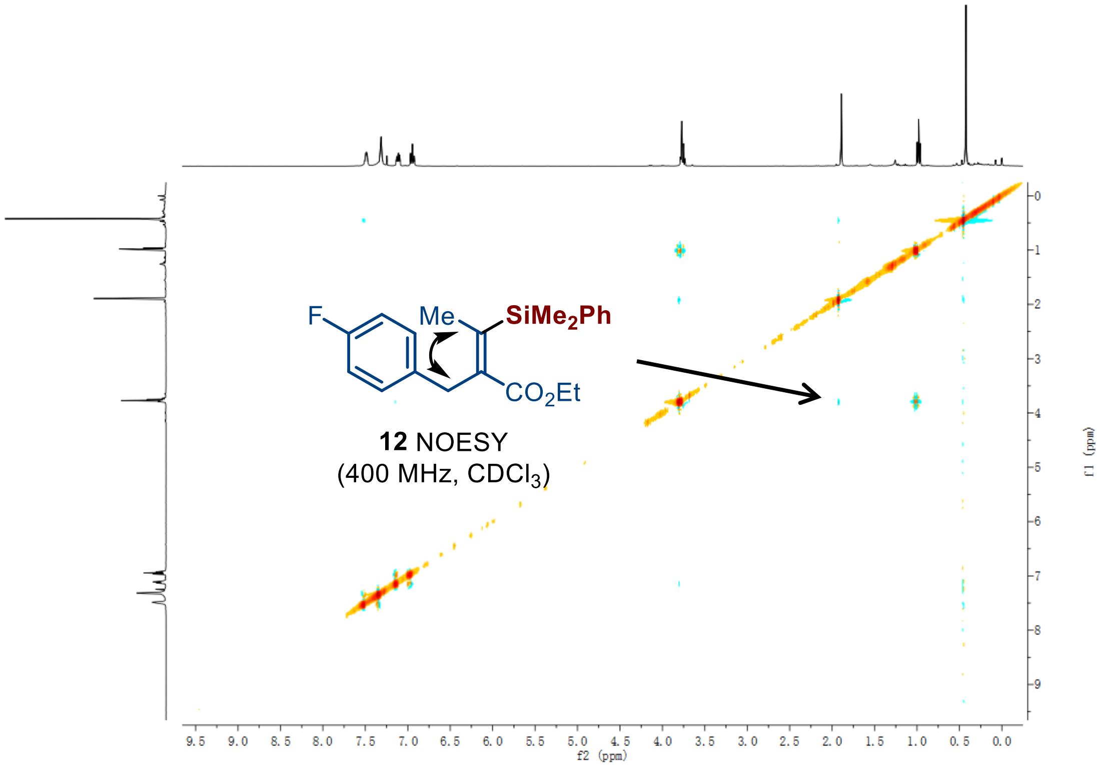

Supplementary Figure 110. NOESY of **12**

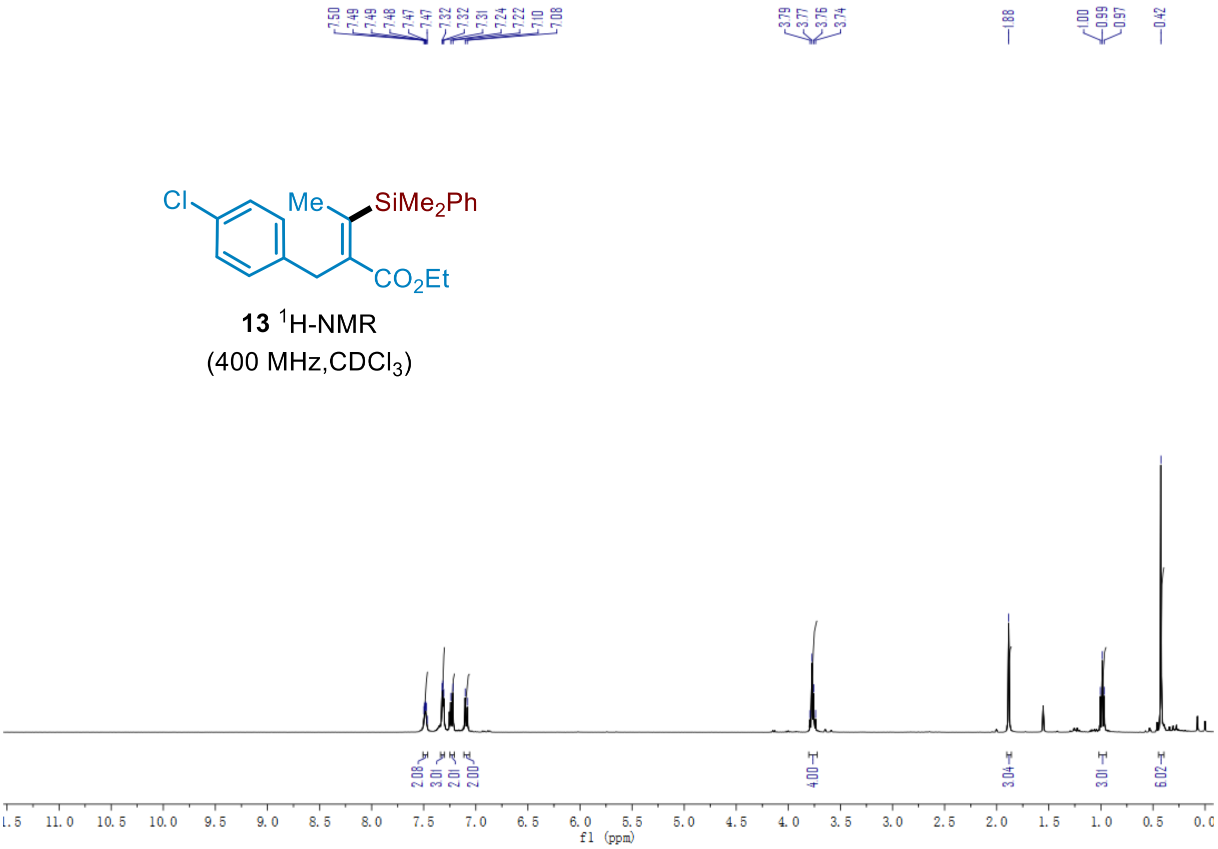

Supplementary Figure 111. **^{1}H-NMR** (400 MHz, $CDCl_3$, 298K) of **13**

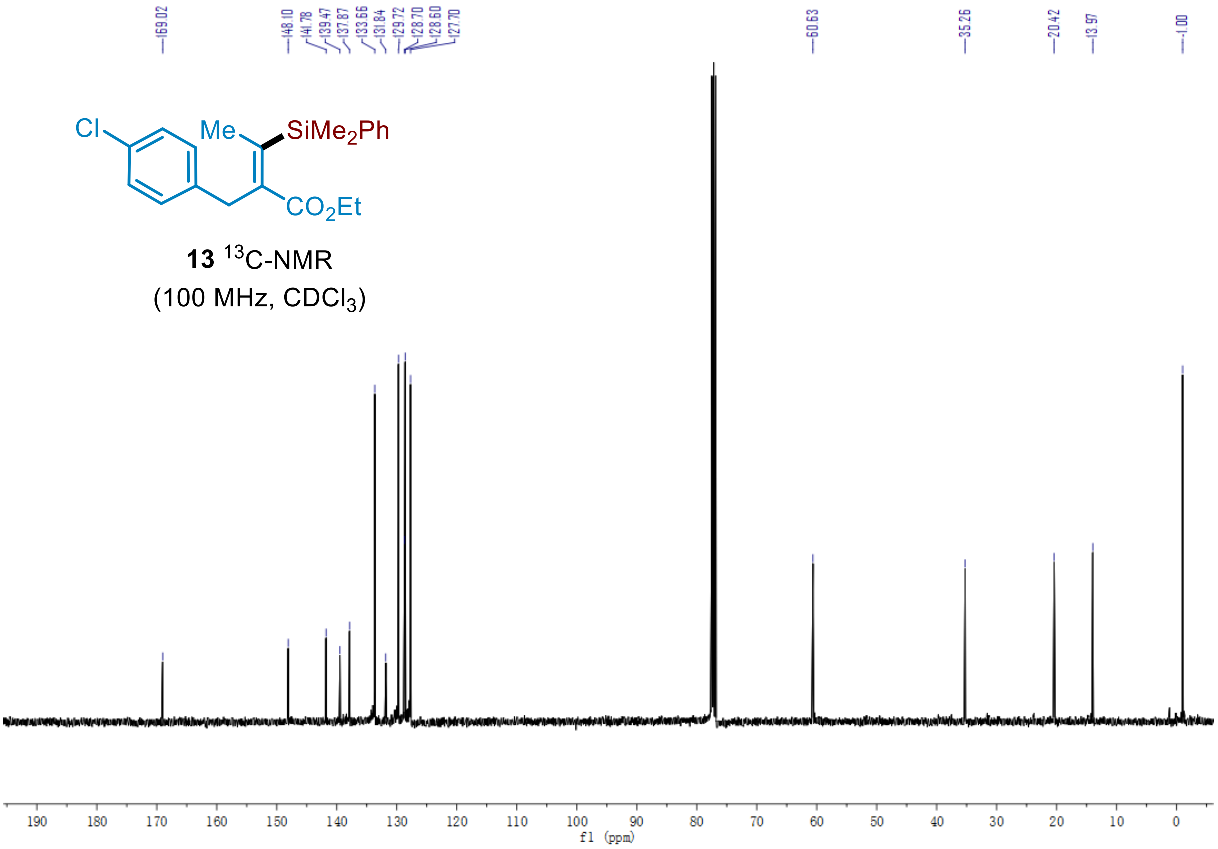

Supplementary Figure 112. **^{13}C-NMR** (100 MHz, $CDCl_3$, 298K) of **13**

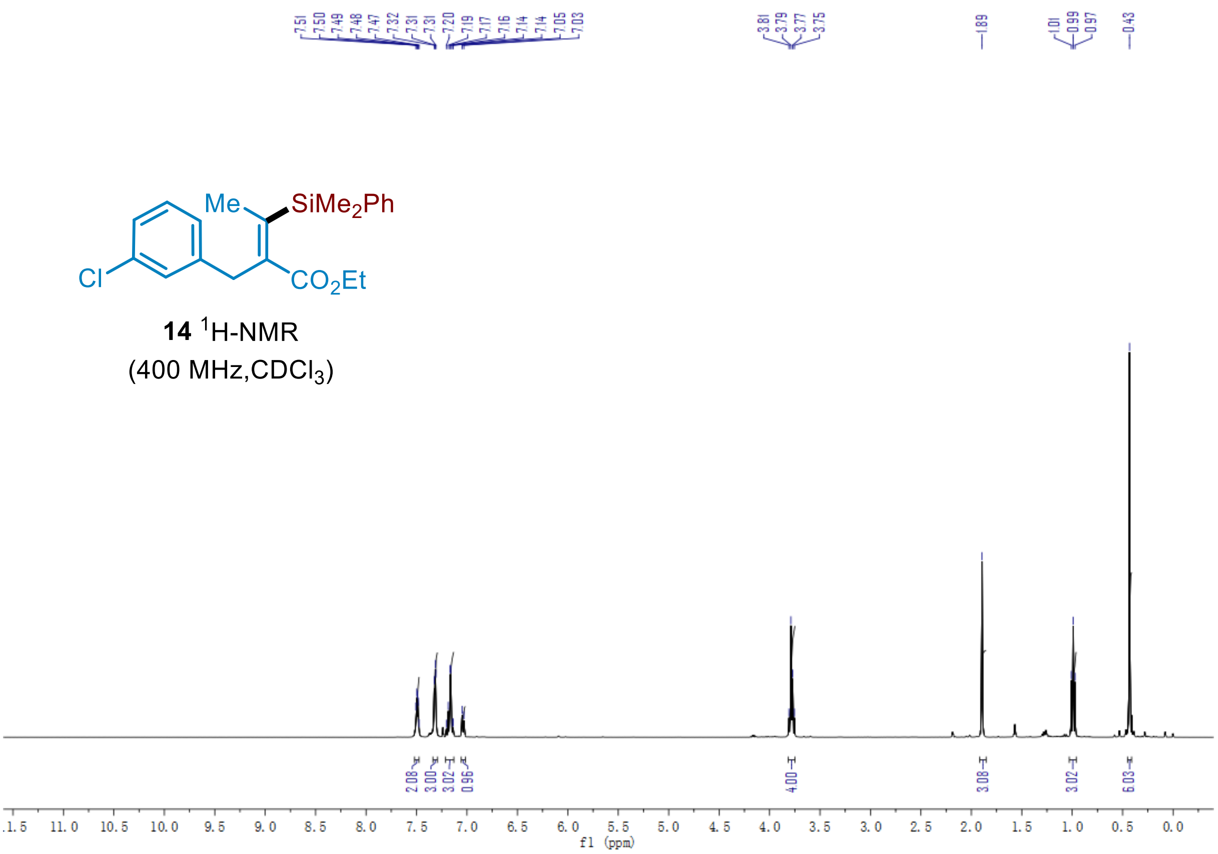

Supplementary Figure 113. **^{1}H-NMR** (400 MHz, $CDCl_3$, 298K) of **14**

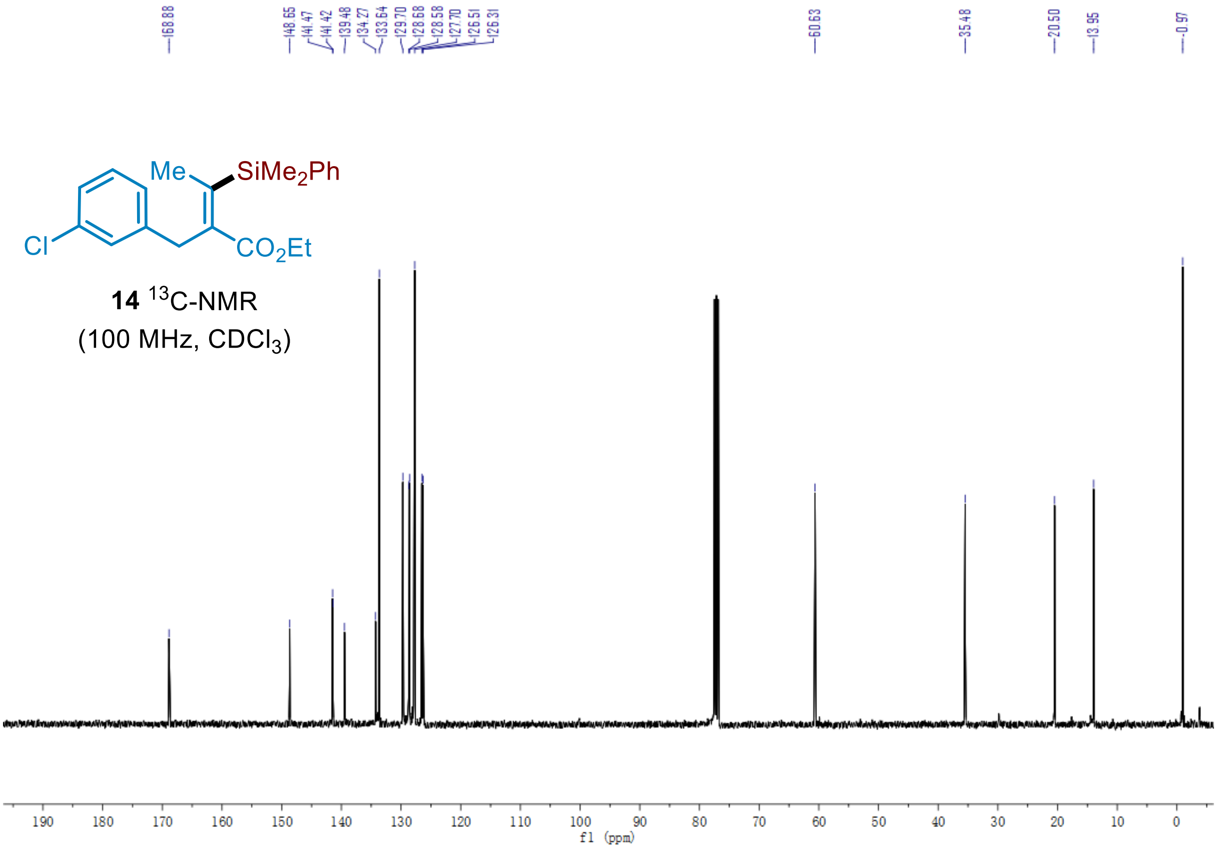

Supplementary Figure 114. **^{13}C-NMR** (100 MHz, $CDCl_3$, 298K) of **14**

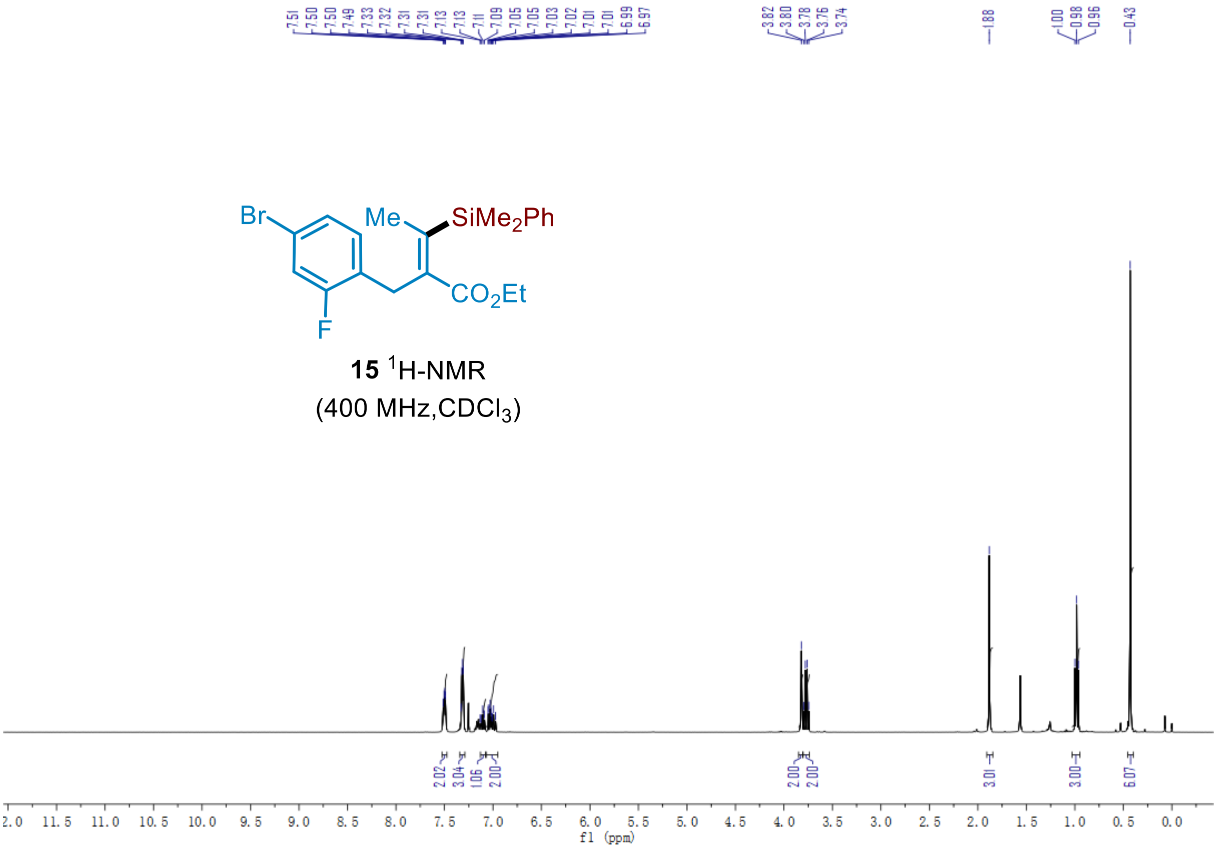

Supplementary Figure 115. **^{1}H-NMR** (400 MHz, $CDCl_3$, 298K) of **15**

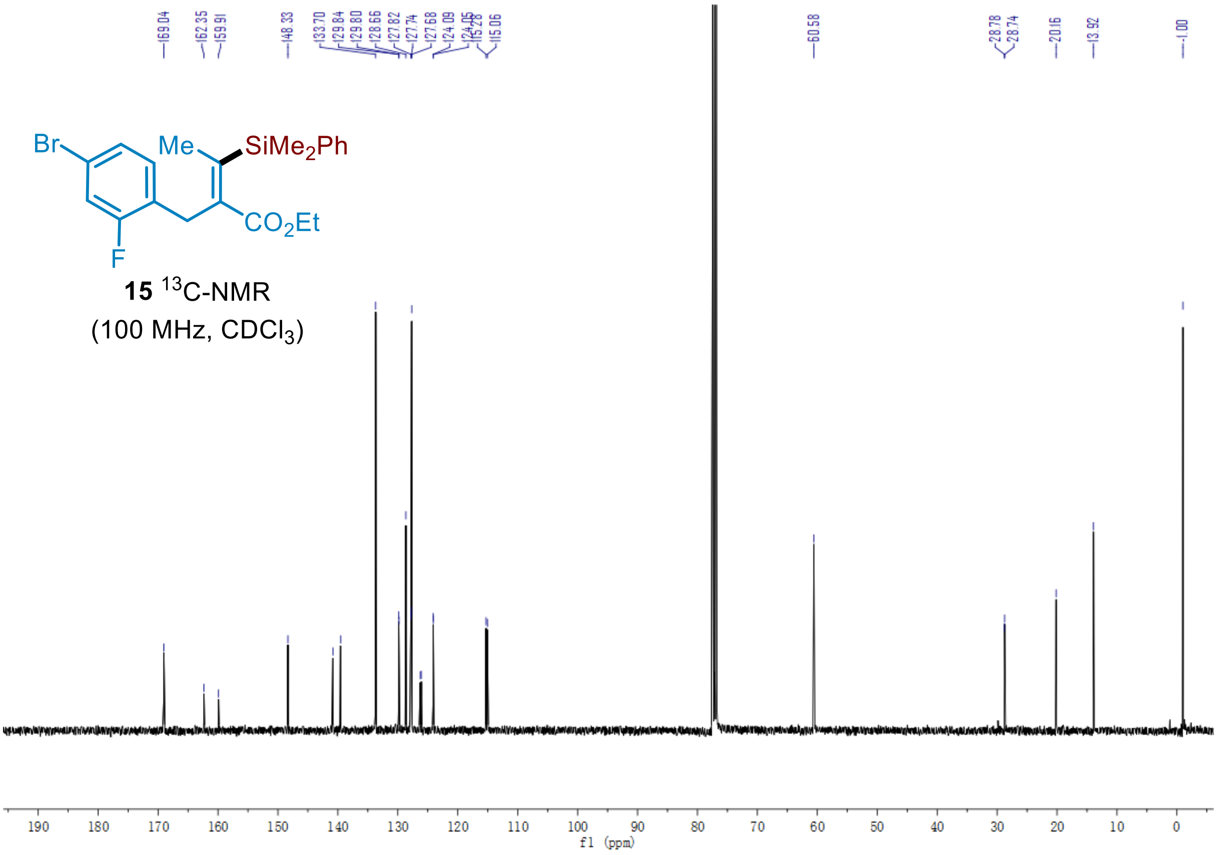

Supplementary Figure 116. **^{13}C-NMR** (100 MHz, $CDCl_3$, 298K) of **15**

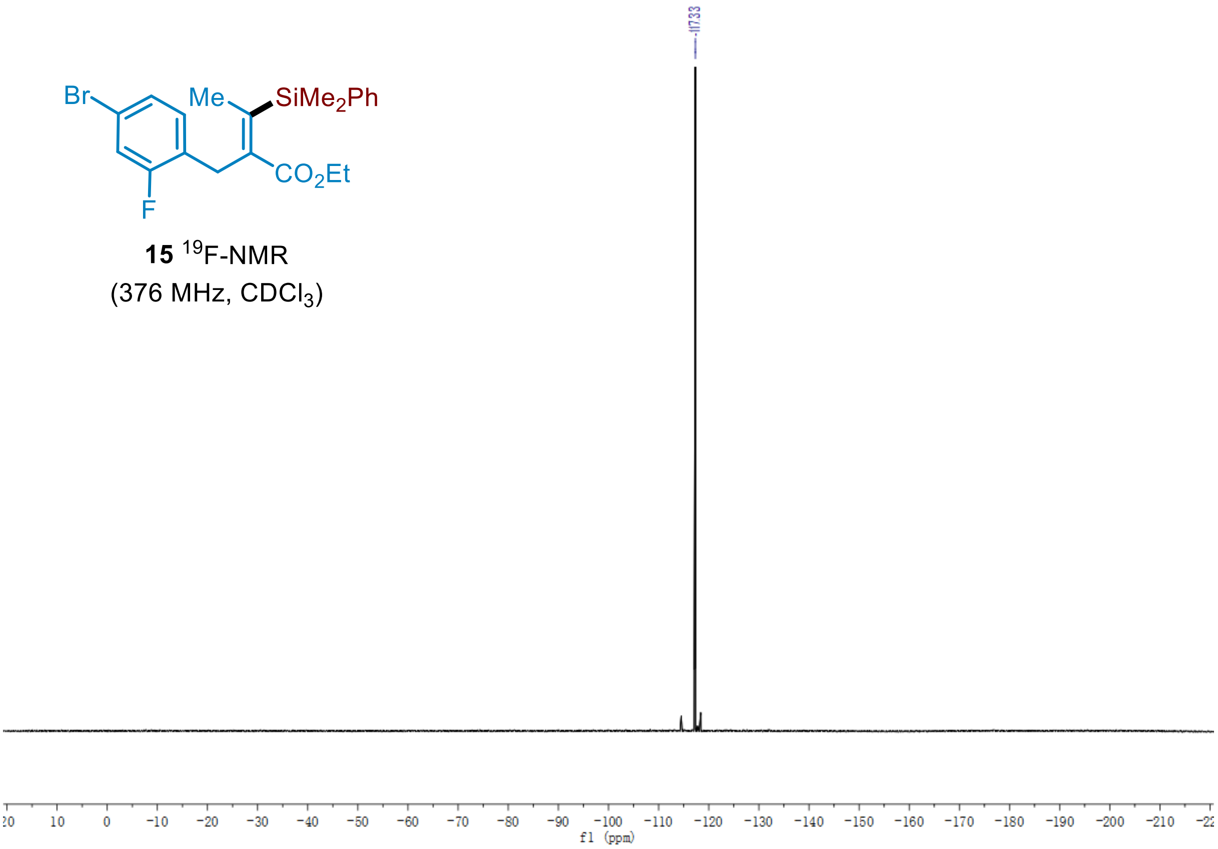

Supplementary Figure 117. **^{19}F-NMR** (376 MHz, $CDCl_3$, 298K) of **15**

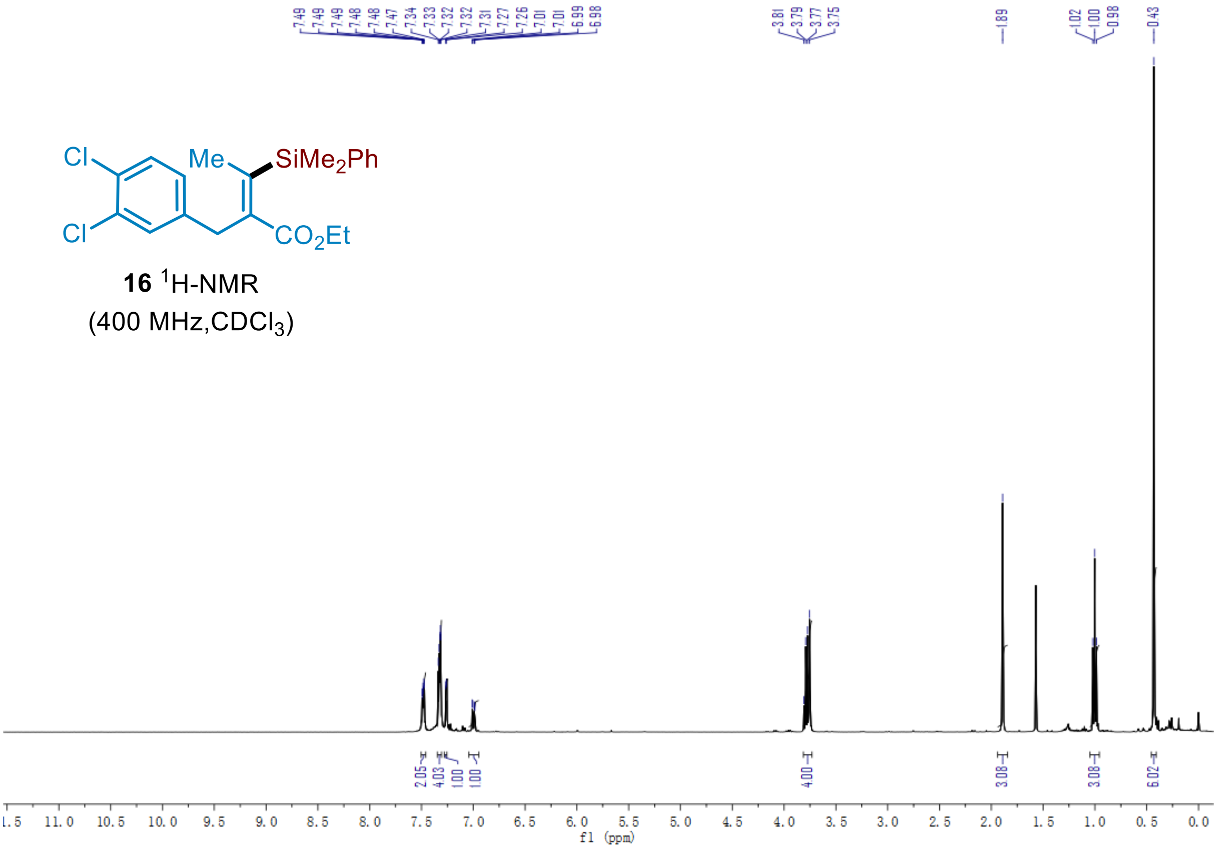

Supplementary Figure 118. **^{1}H-NMR** (400 MHz, $CDCl_3$, 298K) of **16**

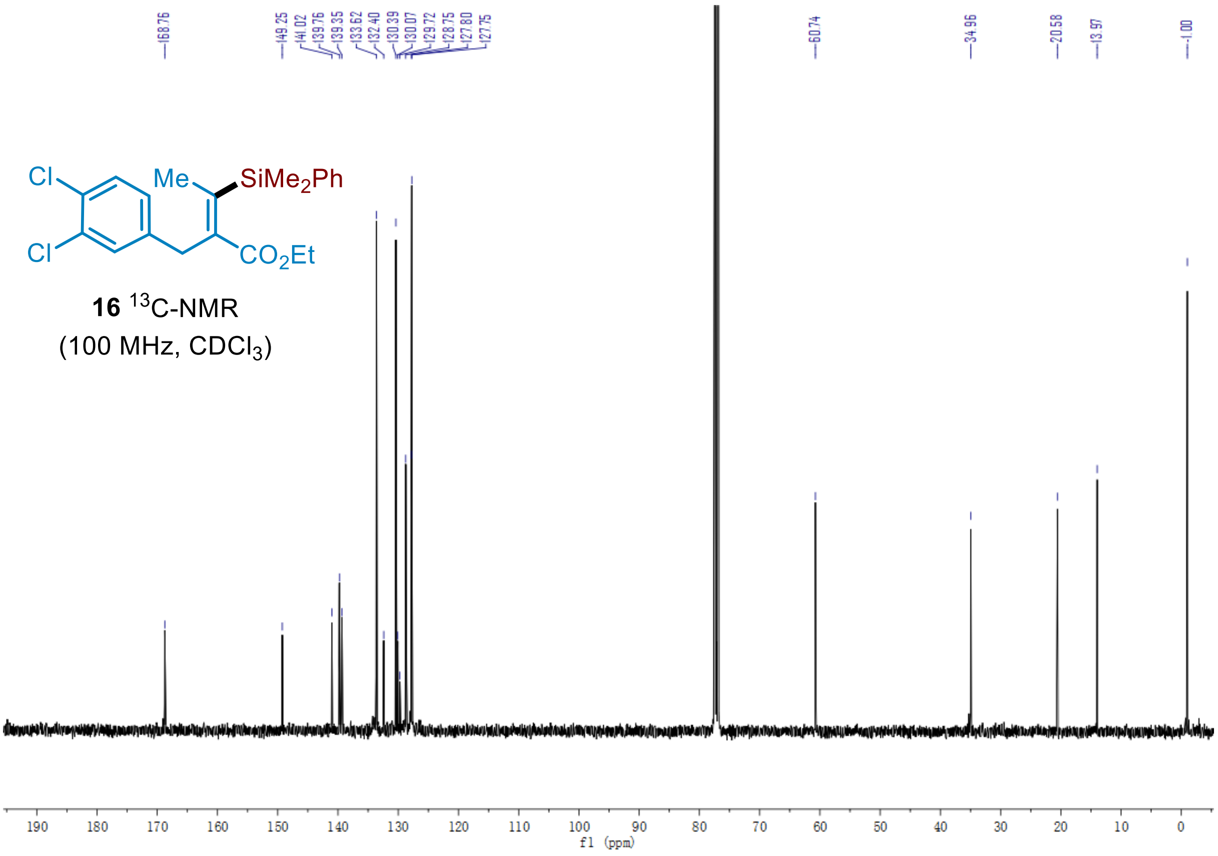

Supplementary Figure 119. **^{13}C-NMR** (100 MHz, $CDCl_3$, 298K) of **16**

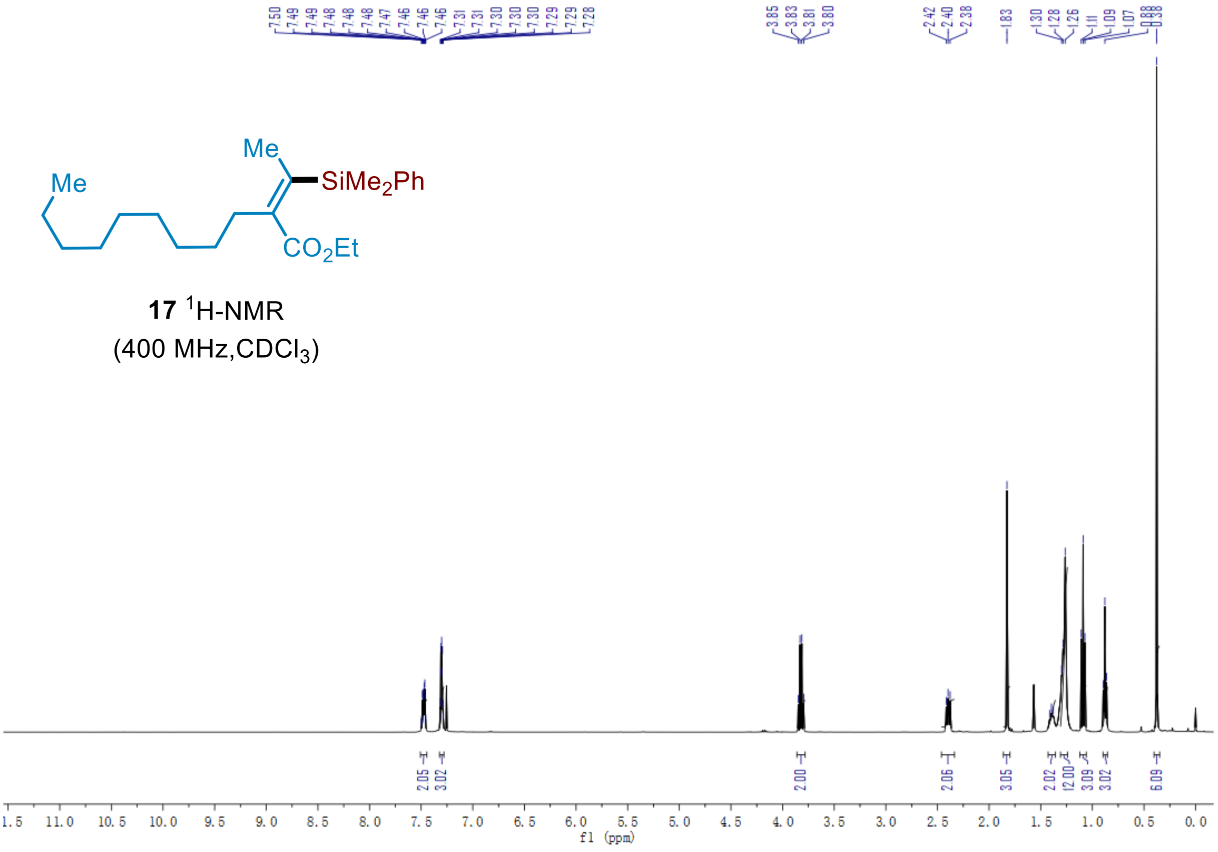

Supplementary Figure 120. **^{1}H-NMR** (400 MHz, $CDCl_3$, 298K) of **17**

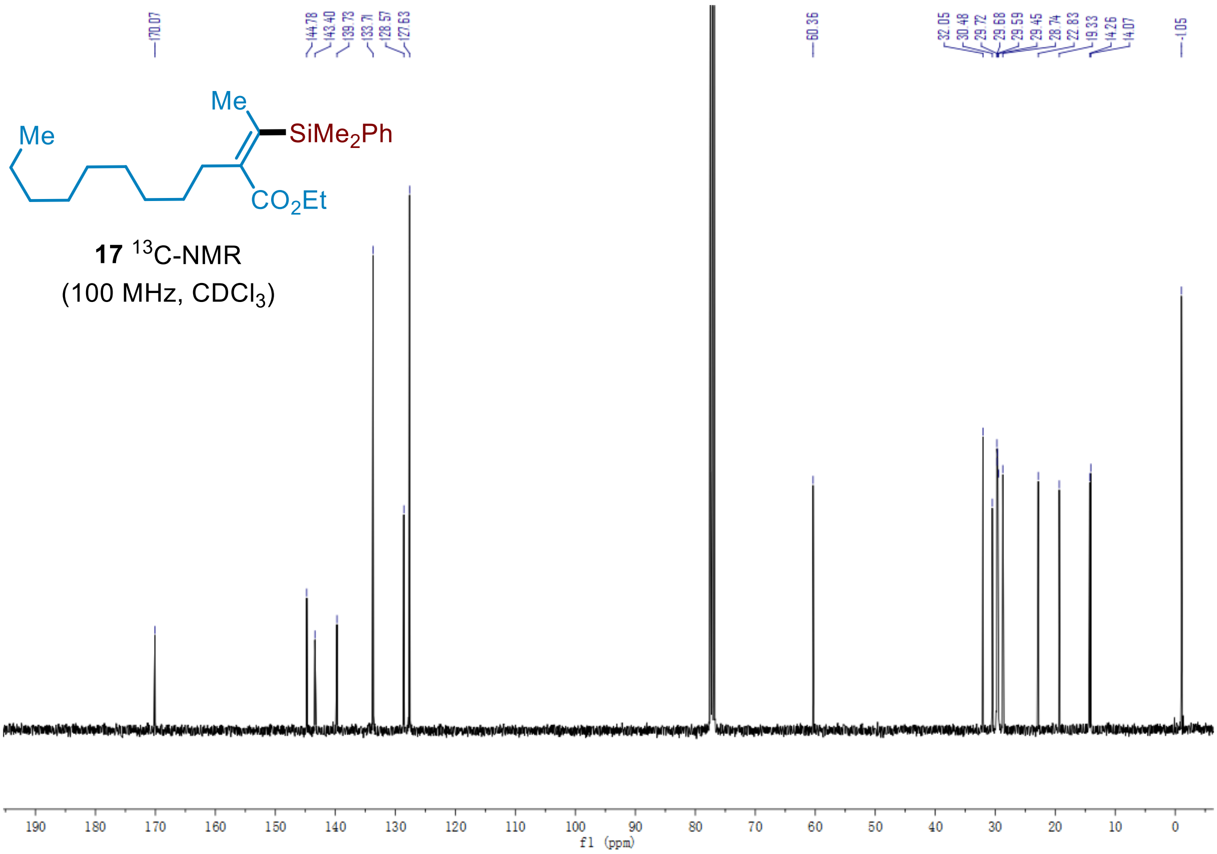

Supplementary Figure 121. **^{13}C-NMR** (100 MHz, $CDCl_3$, 298K) of **17**

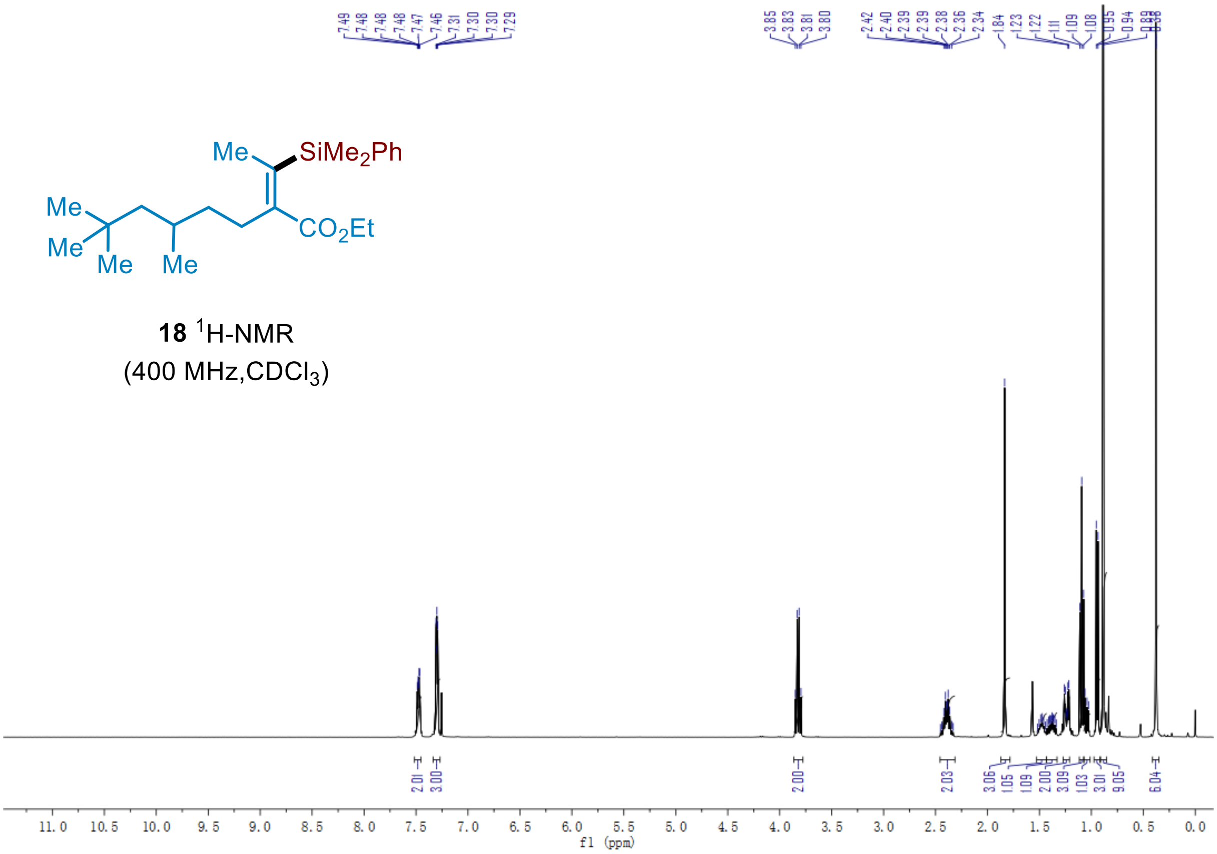

Supplementary Figure 122. **^{1}H-NMR** (400 MHz, $CDCl_3$, 298K) of **18**

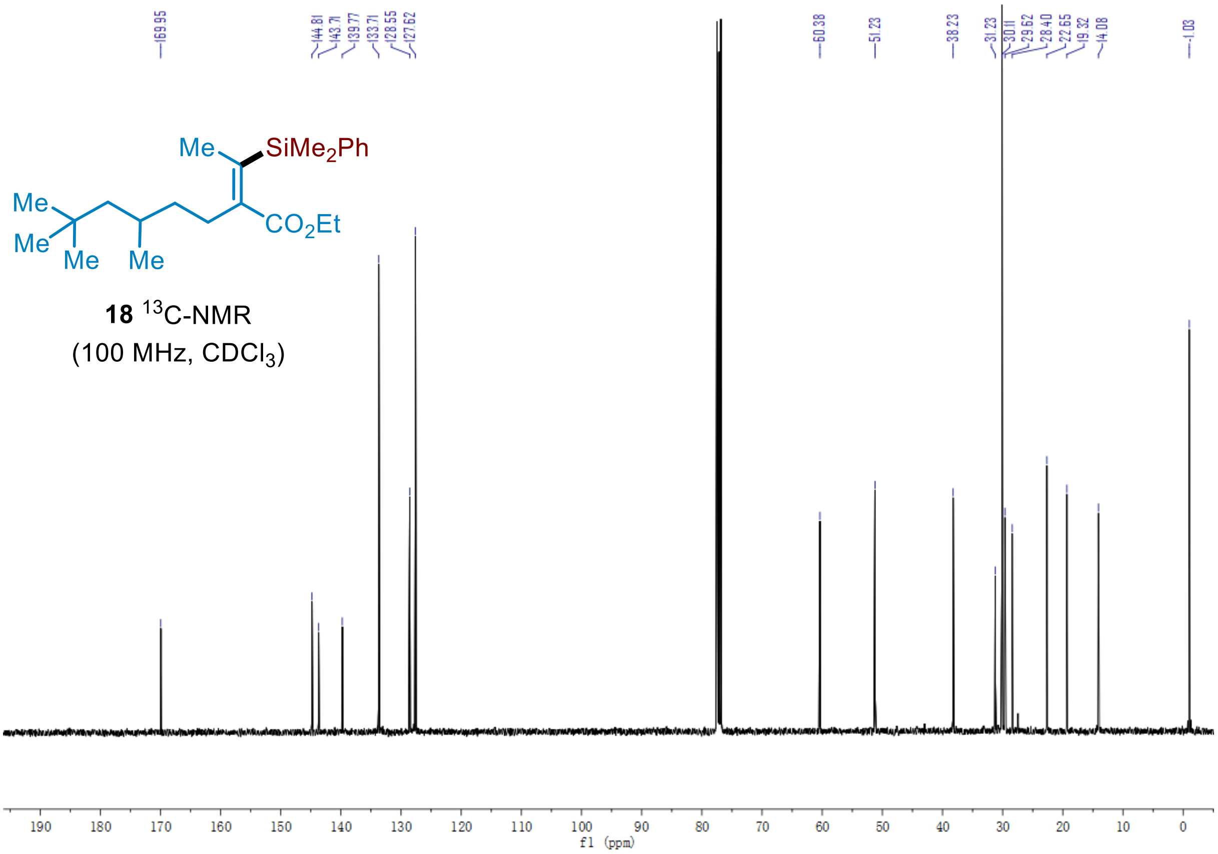

Supplementary Figure 123. **^{13}C-NMR** (100 MHz, $CDCl_3$, 298K) of **18**

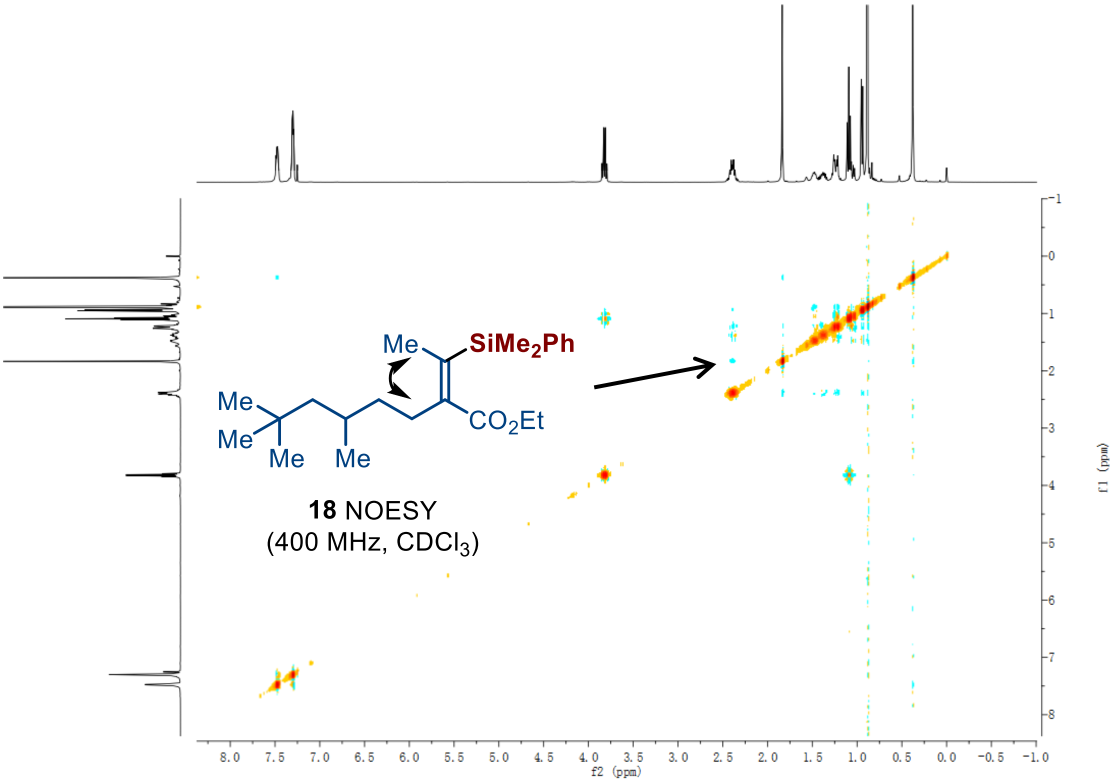

Supplementary Figure 124. NOESY of **18**

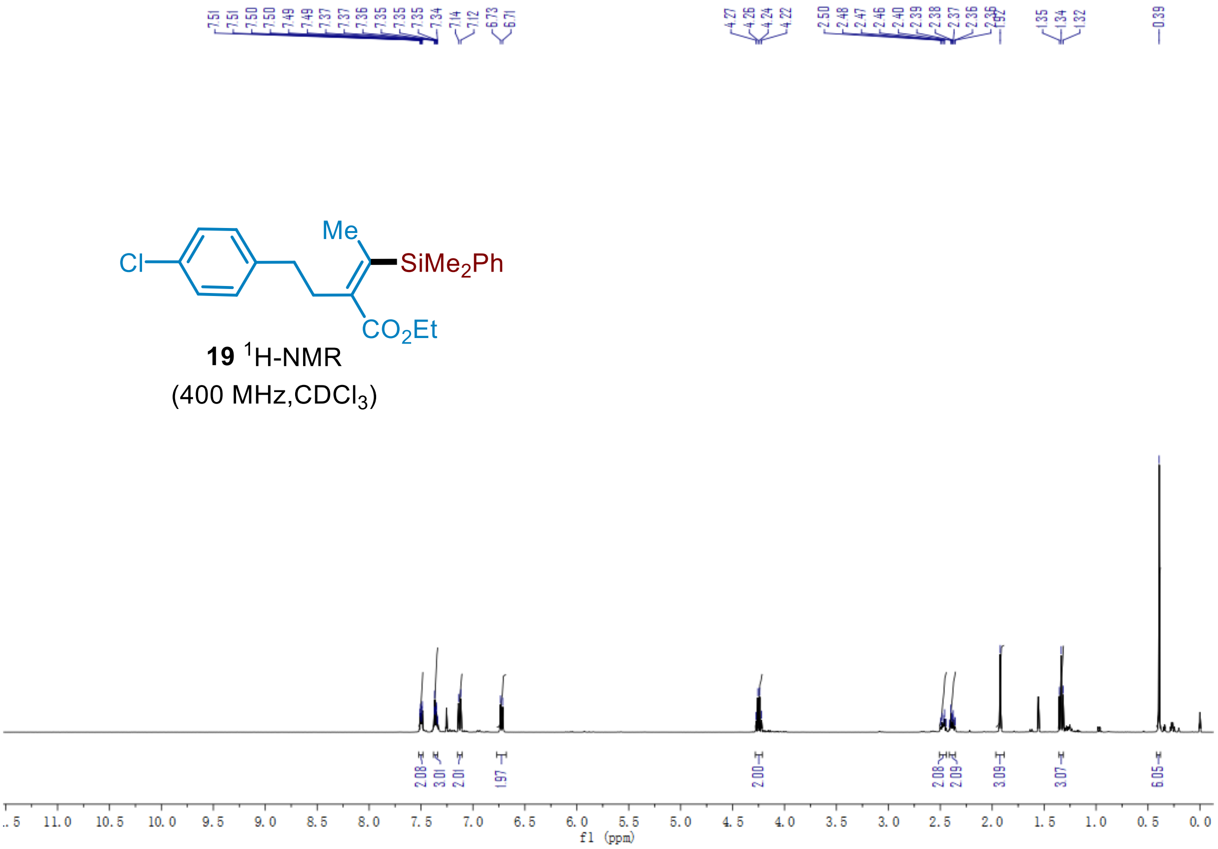

Supplementary Figure 125. ^{1}**H-NMR** (400 MHz, $CDCl_3$, 298K) of **19**

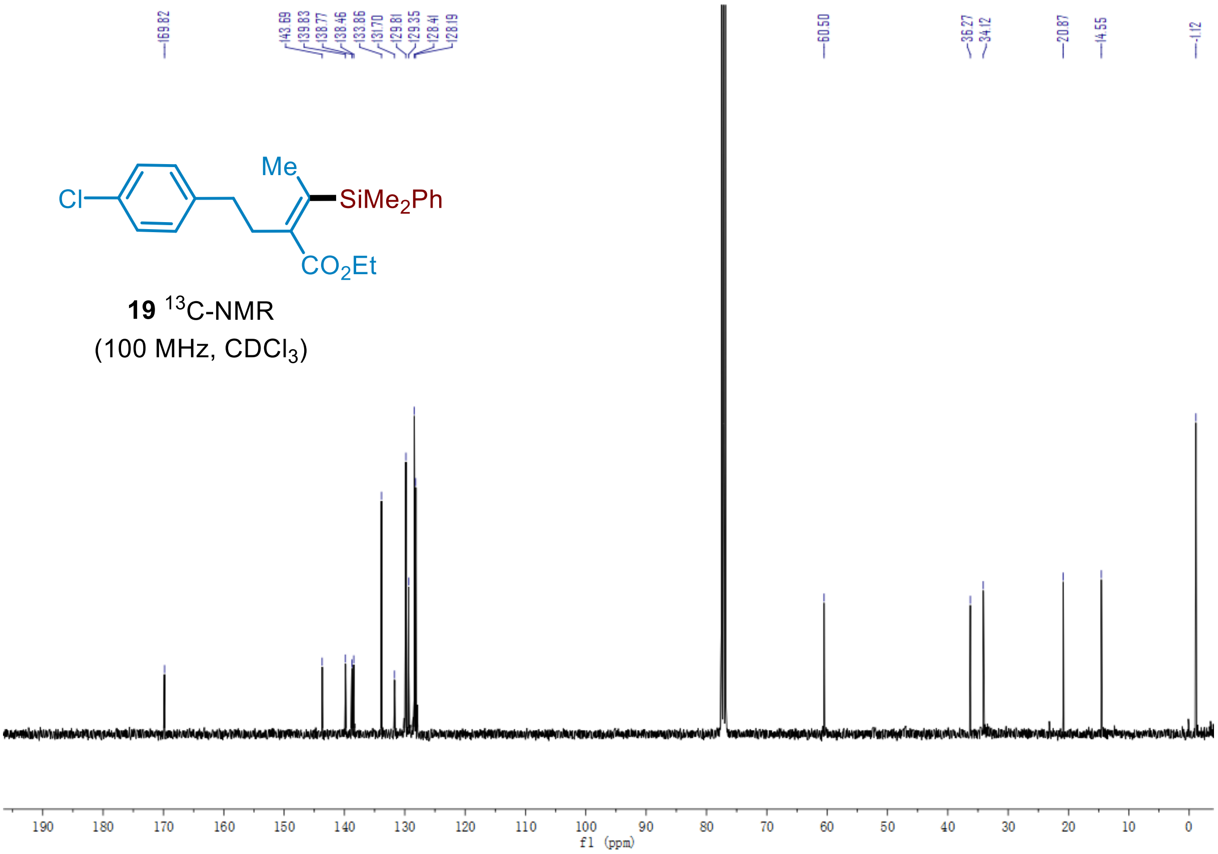

Supplementary Figure 126. **^{13}C-NMR** (100 MHz, $CDCl_3$, 298K) of **19**

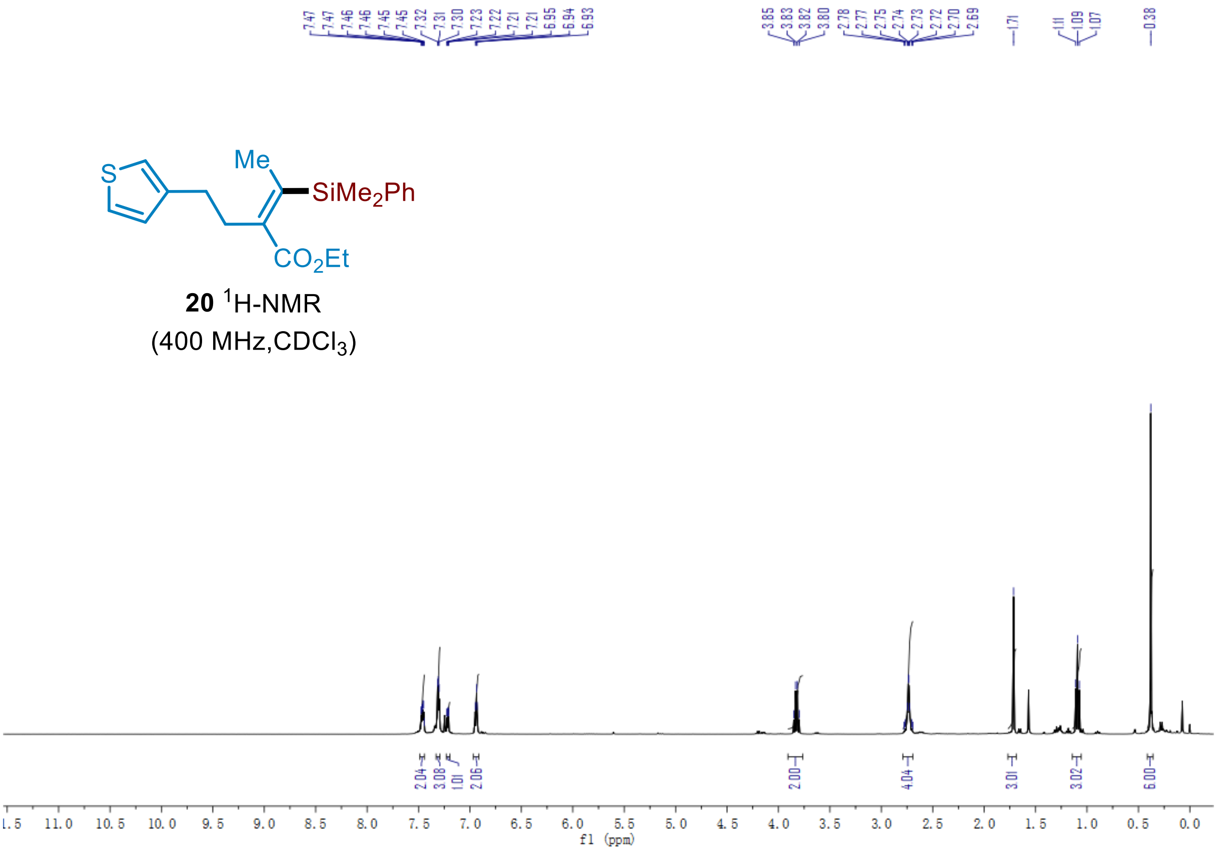

Supplementary Figure 127. **^{1}H-NMR** (400 MHz, $CDCl_3$, 298K) of **20**

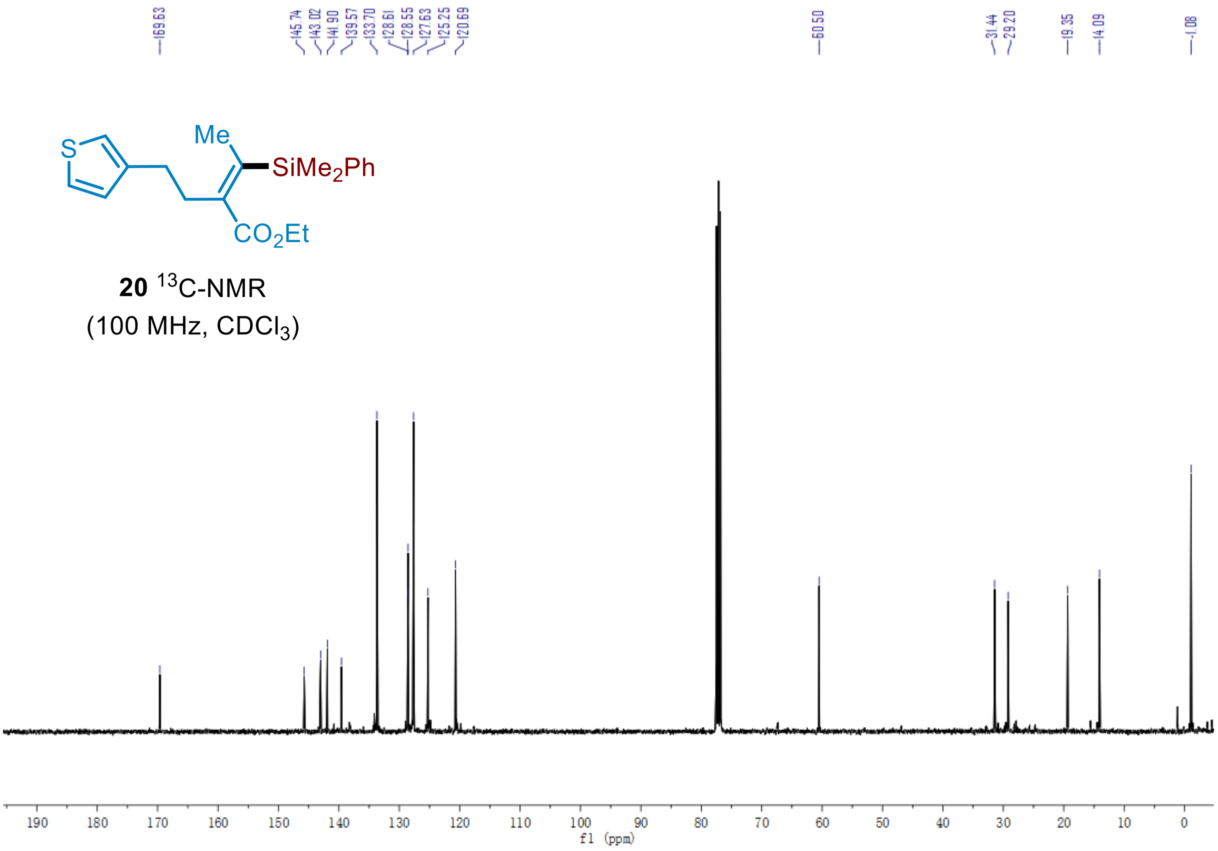

Supplementary Figure 128. **^{13}C-NMR** (100 MHz, $CDCl_3$, 298K) of **20**

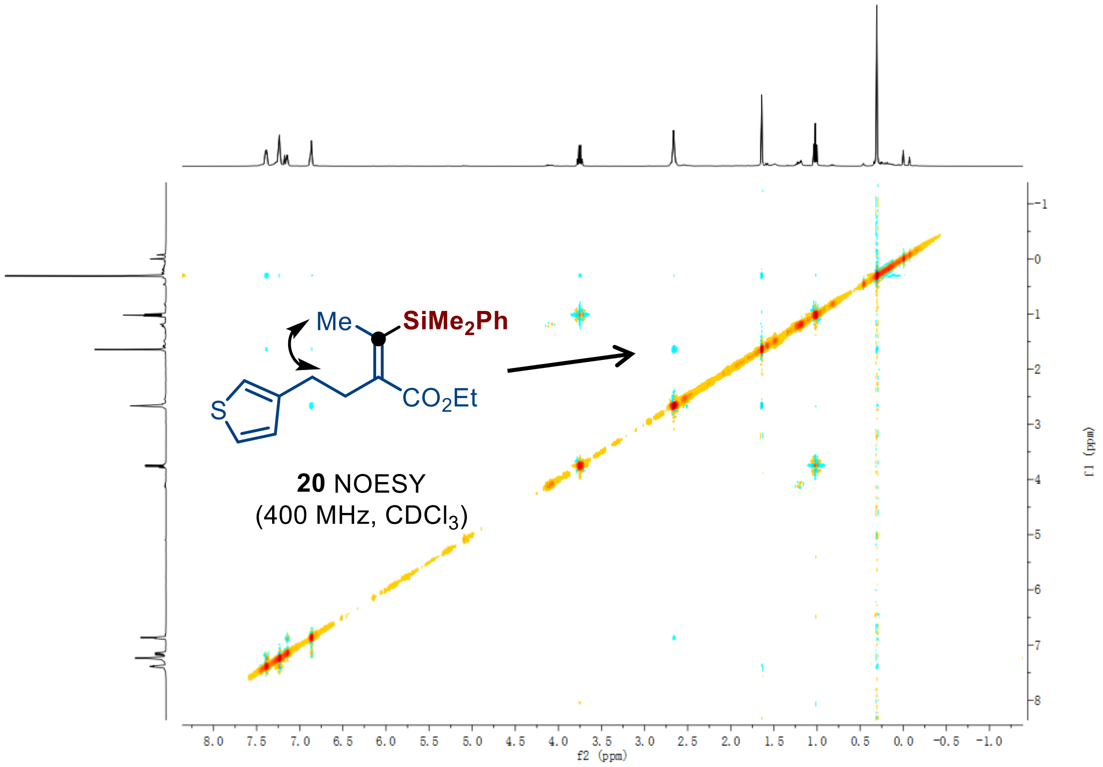

Supplementary Figure 129.**NOESY** of **20**

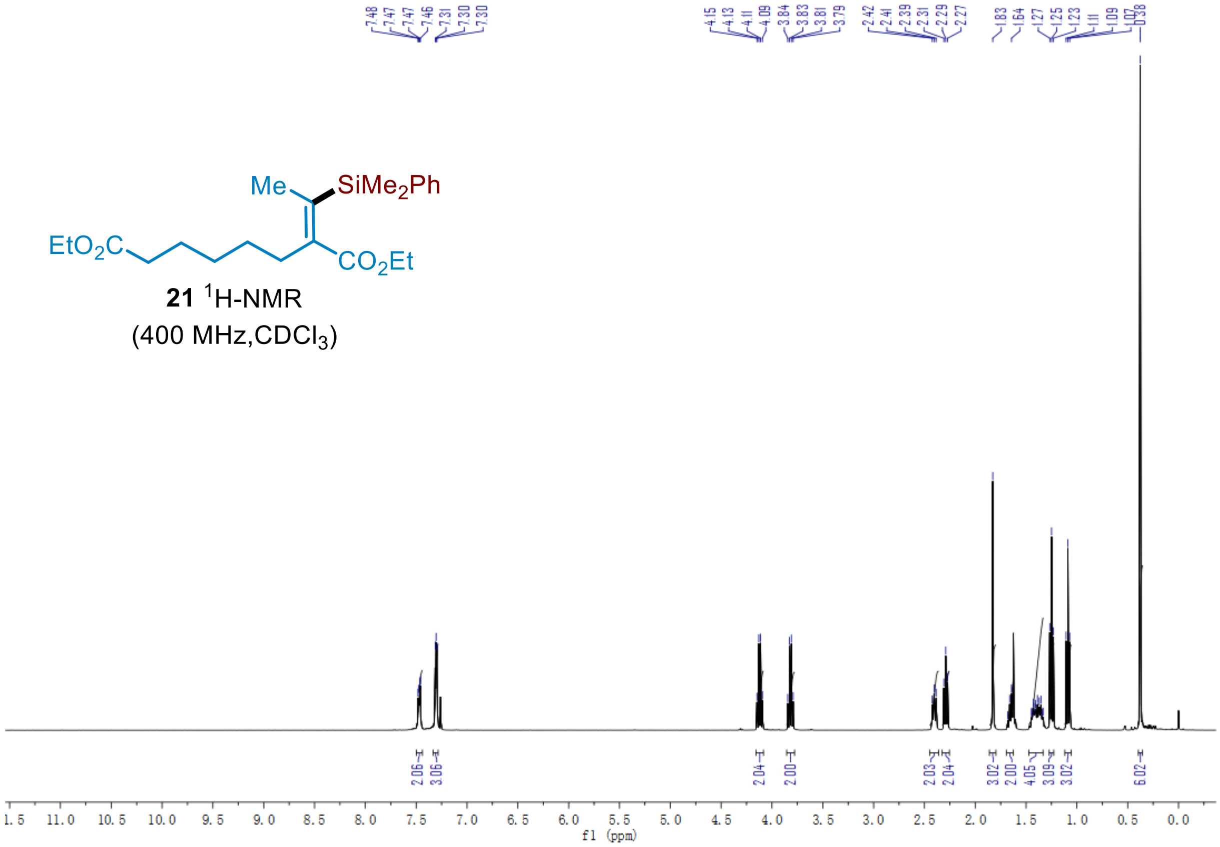

Supplementary Figure 130. **^{1}H-NMR** (400 MHz, $CDCl_3$, 298K) of **21**

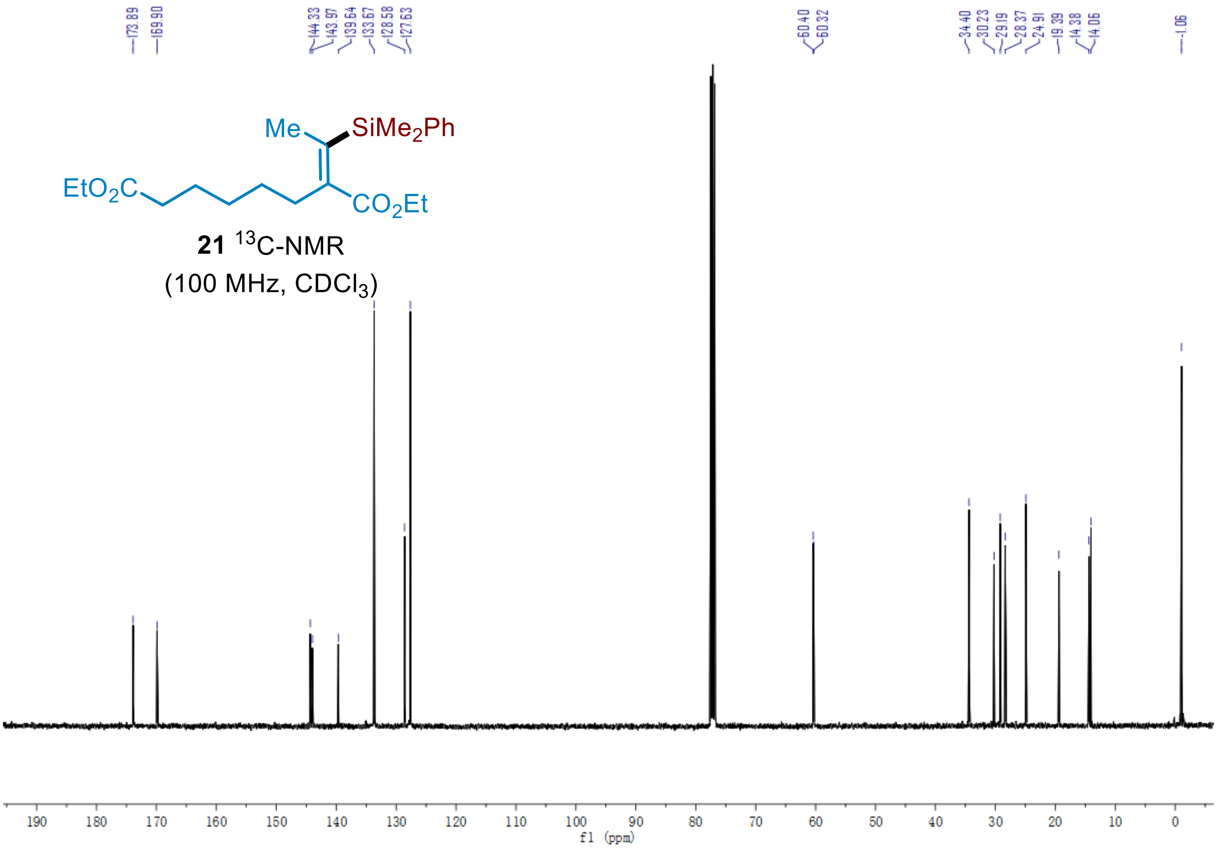

Supplementary Figure 131. **^{13}C-NMR** (100 MHz, $CDCl_3$, 298K) of **21**

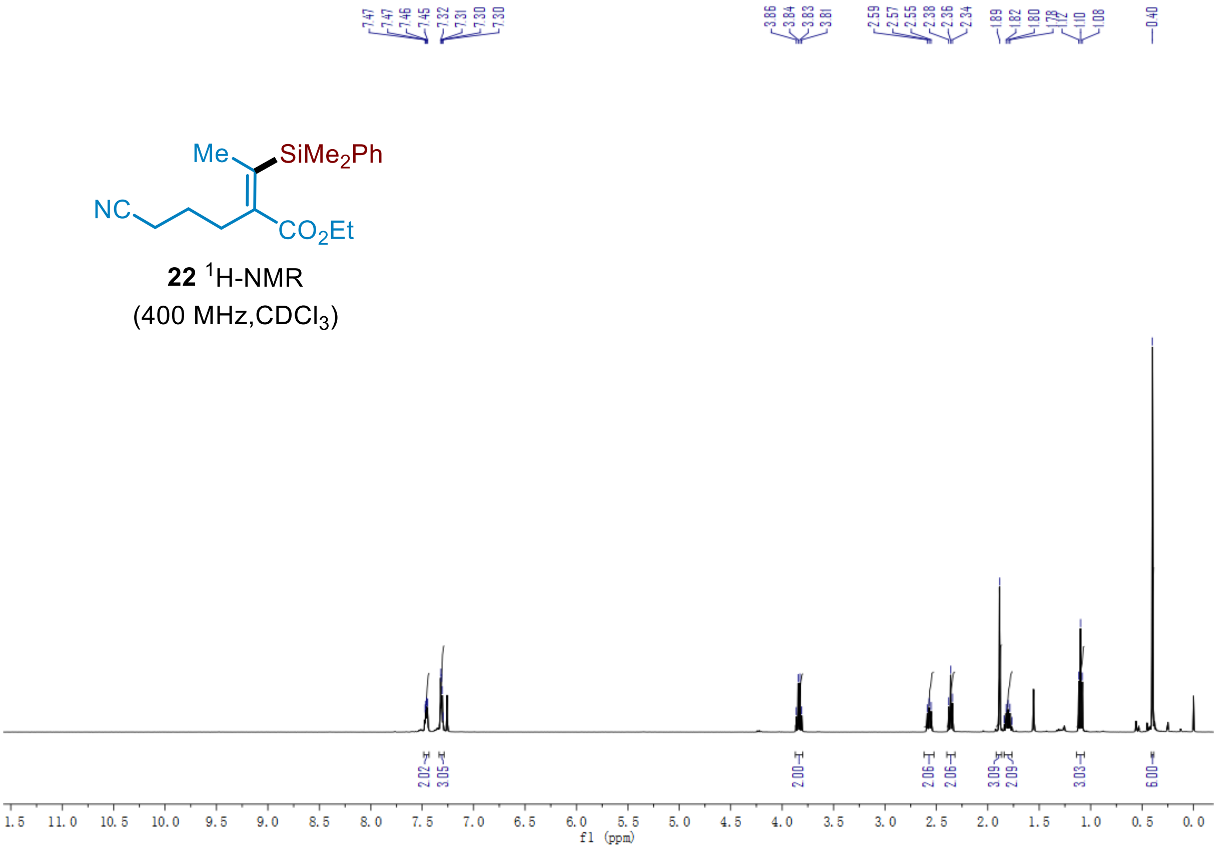

Supplementary Figure 132. **^{1}H-NMR** (400 MHz, $CDCl_3$, 298K) of **22**

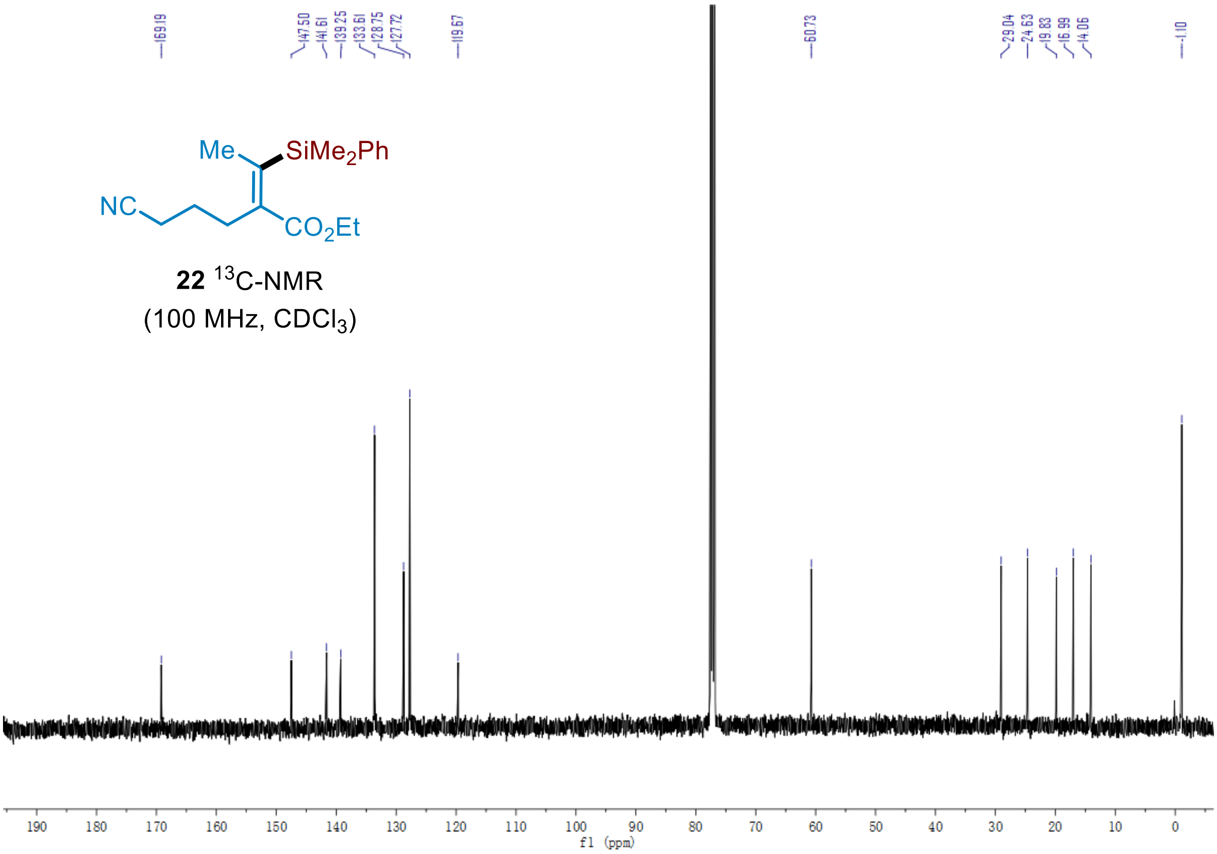

Supplementary Figure 133. **^{13}C-NMR** (100 MHz, $CDCl_3$, 298K) of **22**

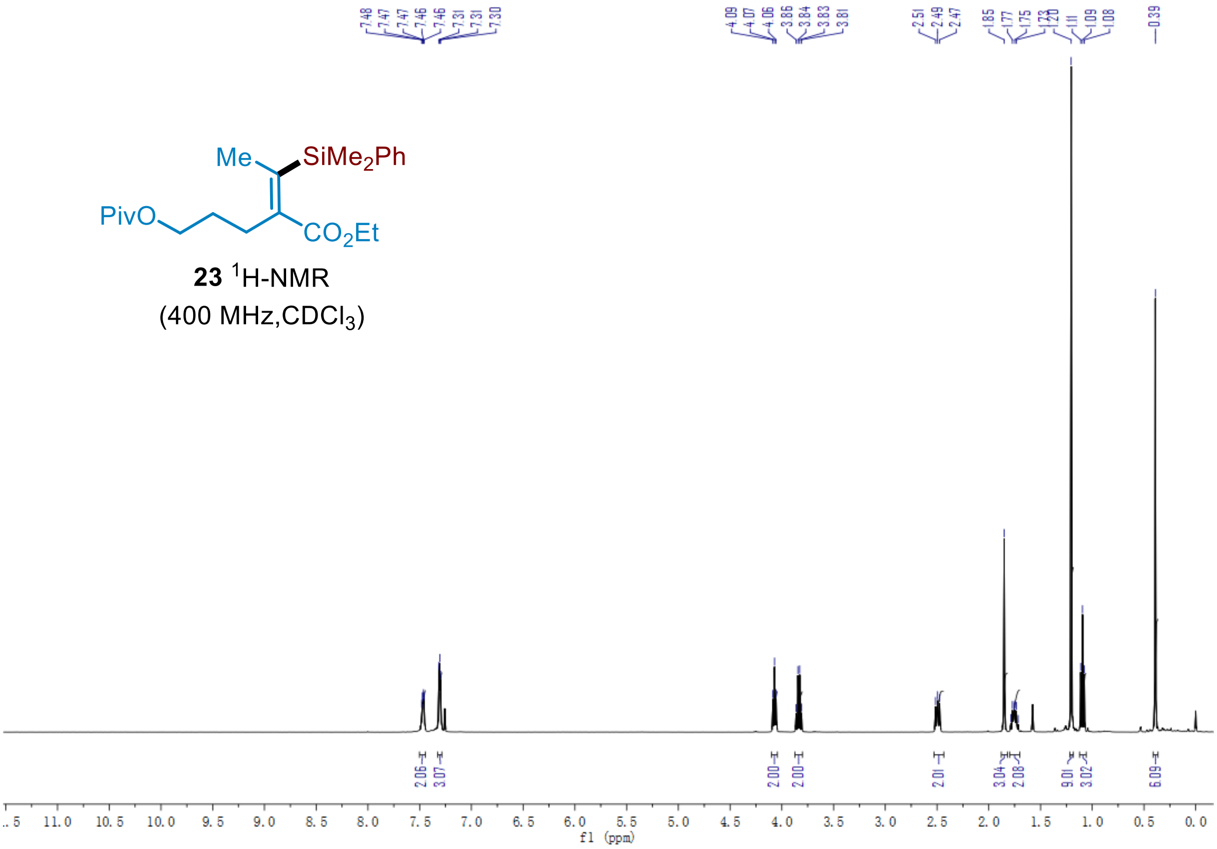

Supplementary Figure 134. **^{1}H-NMR** (400 MHz, $CDCl_3$, 298K) of **23**

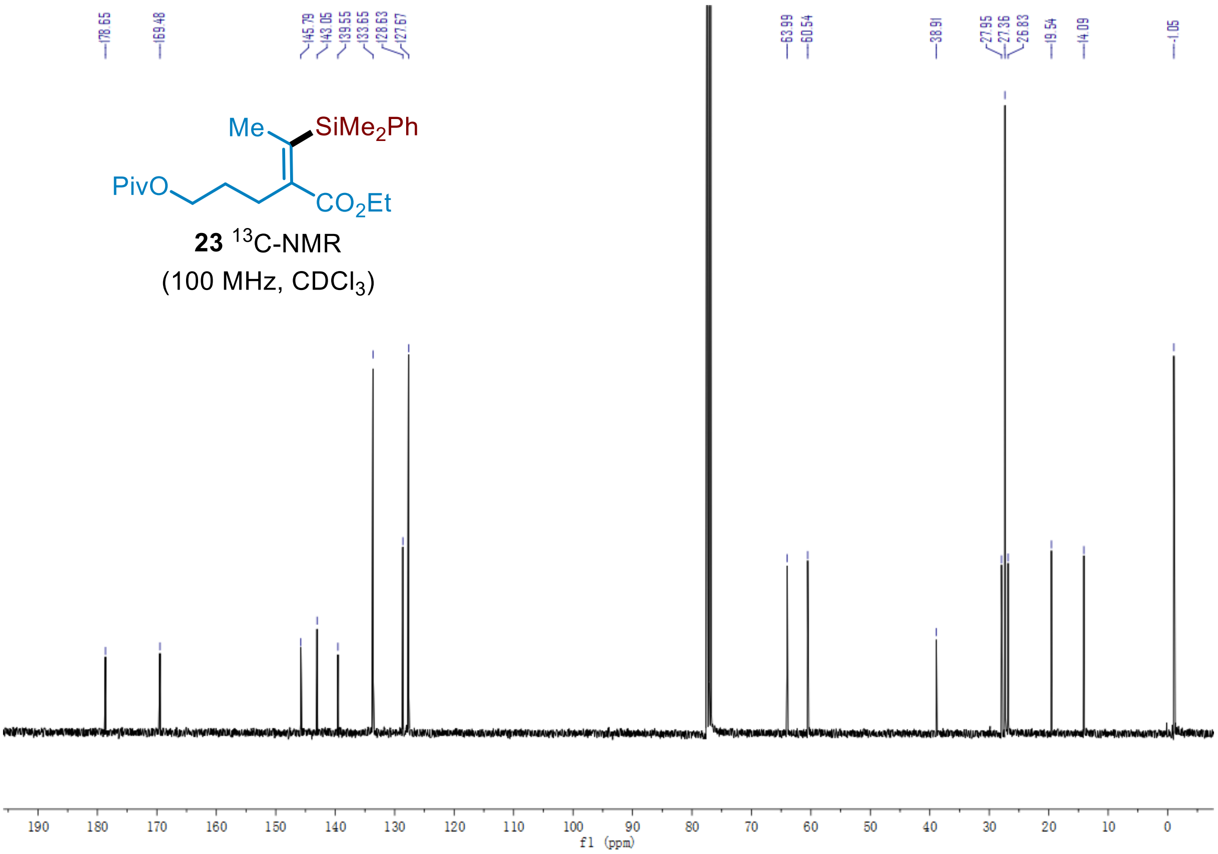

Supplementary Figure 135. **^{13}C-NMR** (100 MHz, $CDCl_3$, 298K) of **23**

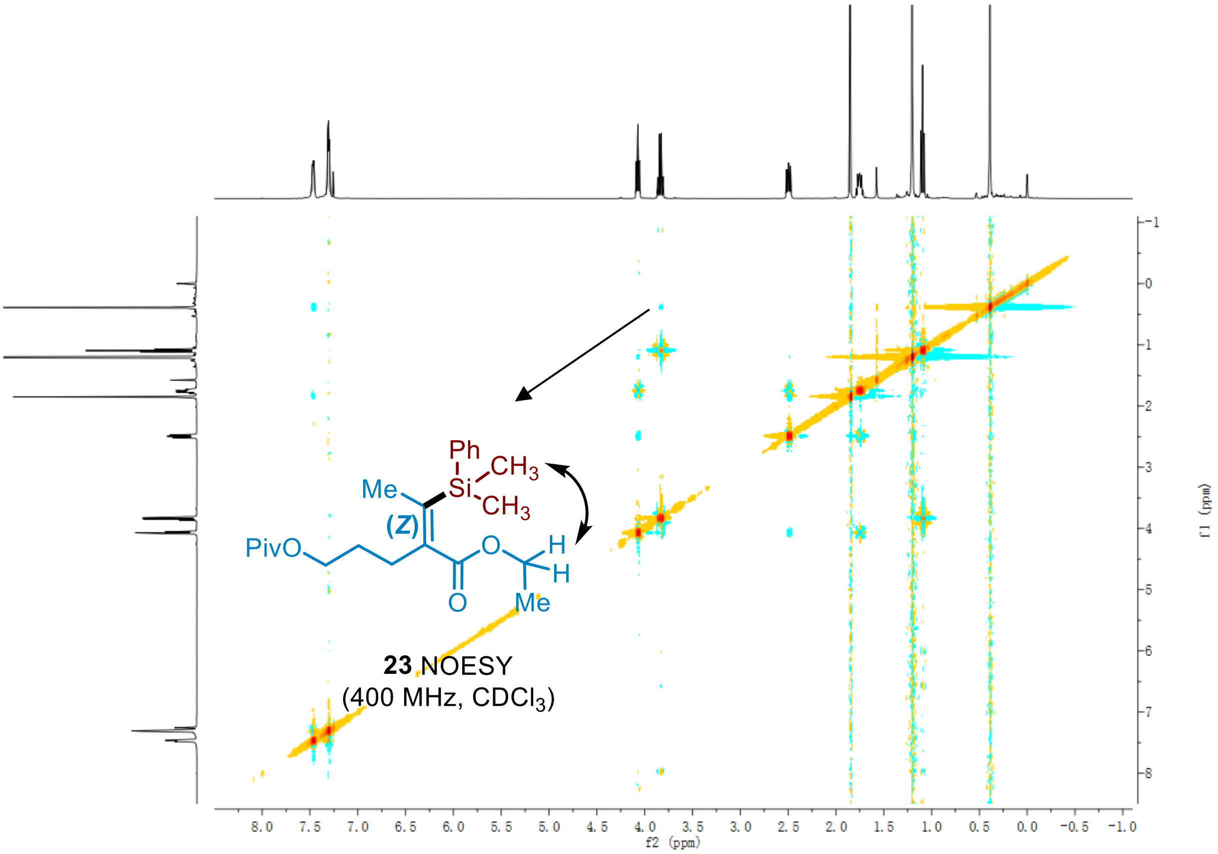

Supplementary Figure 136. **NOESY** of **23**

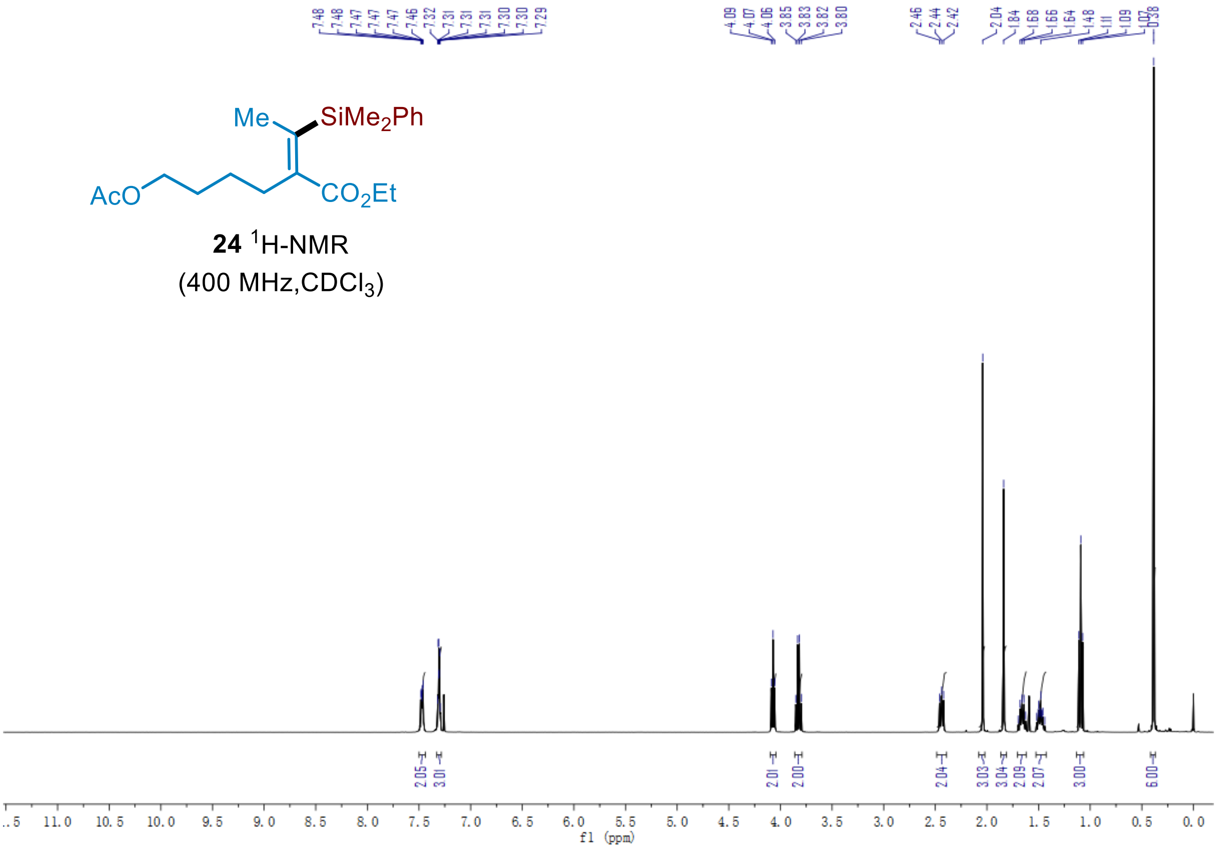

Supplementary Figure 137. **^{1}H-NMR** (400 MHz, $CDCl_3$, 298K) of **24**

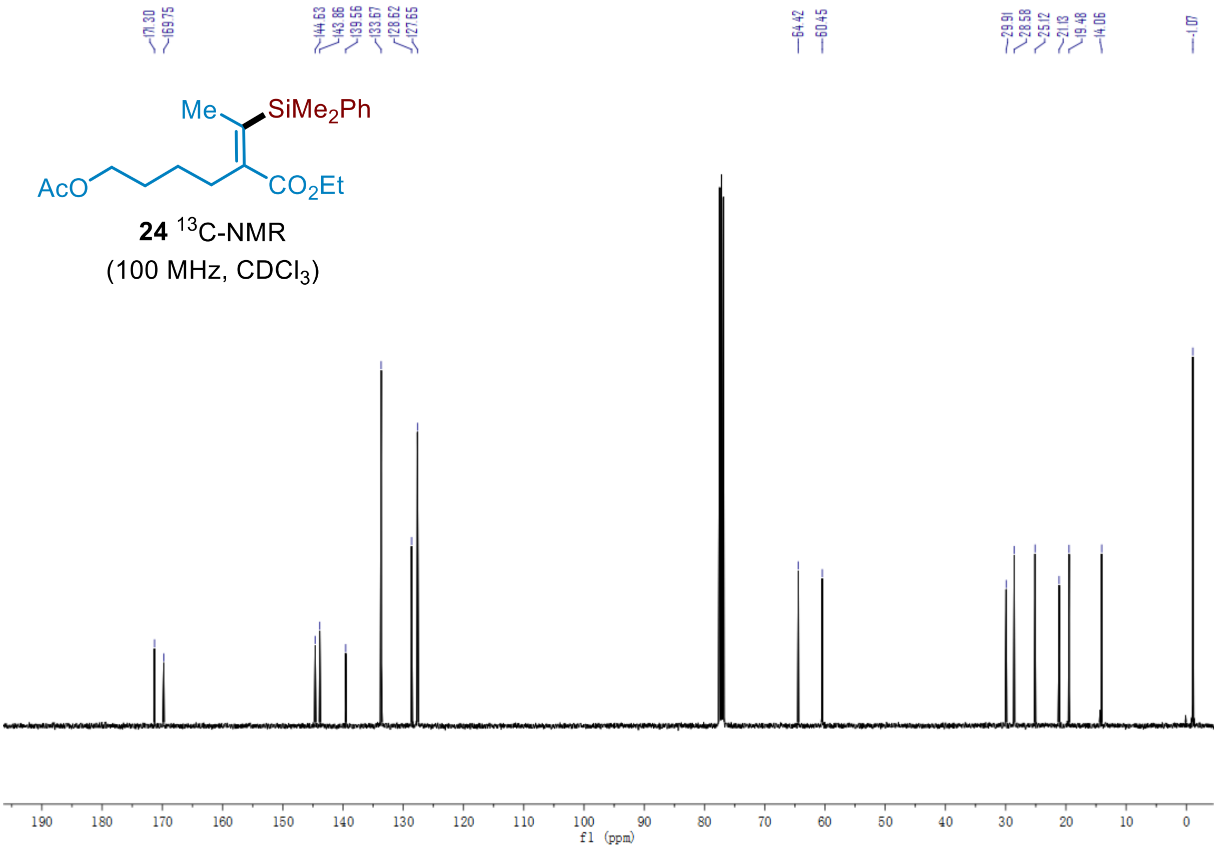

Supplementary Figure 138. **^{13}C-NMR** (100 MHz, $CDCl_3$, 298K) of **24**

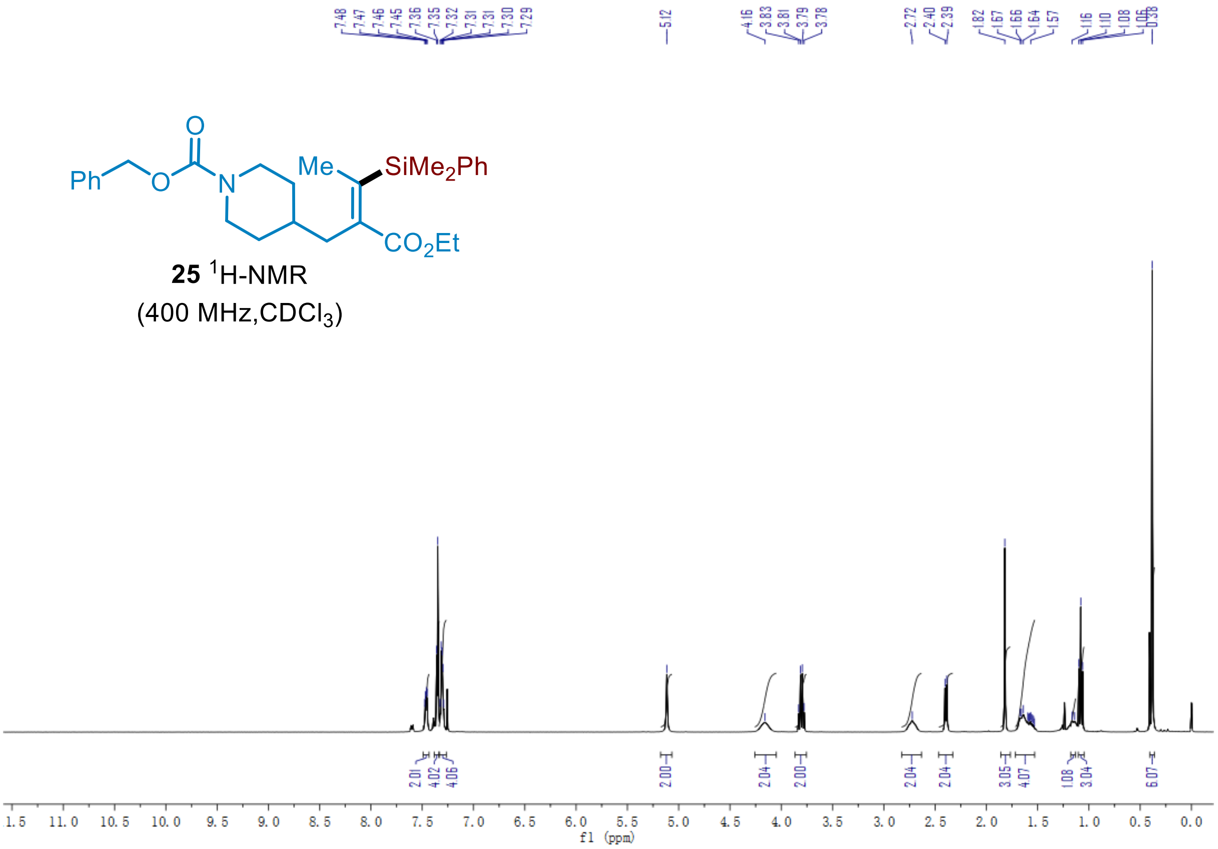

Supplementary Figure 139. **^{1}H-NMR** (400 MHz, $CDCl_3$, 298K) of **25**

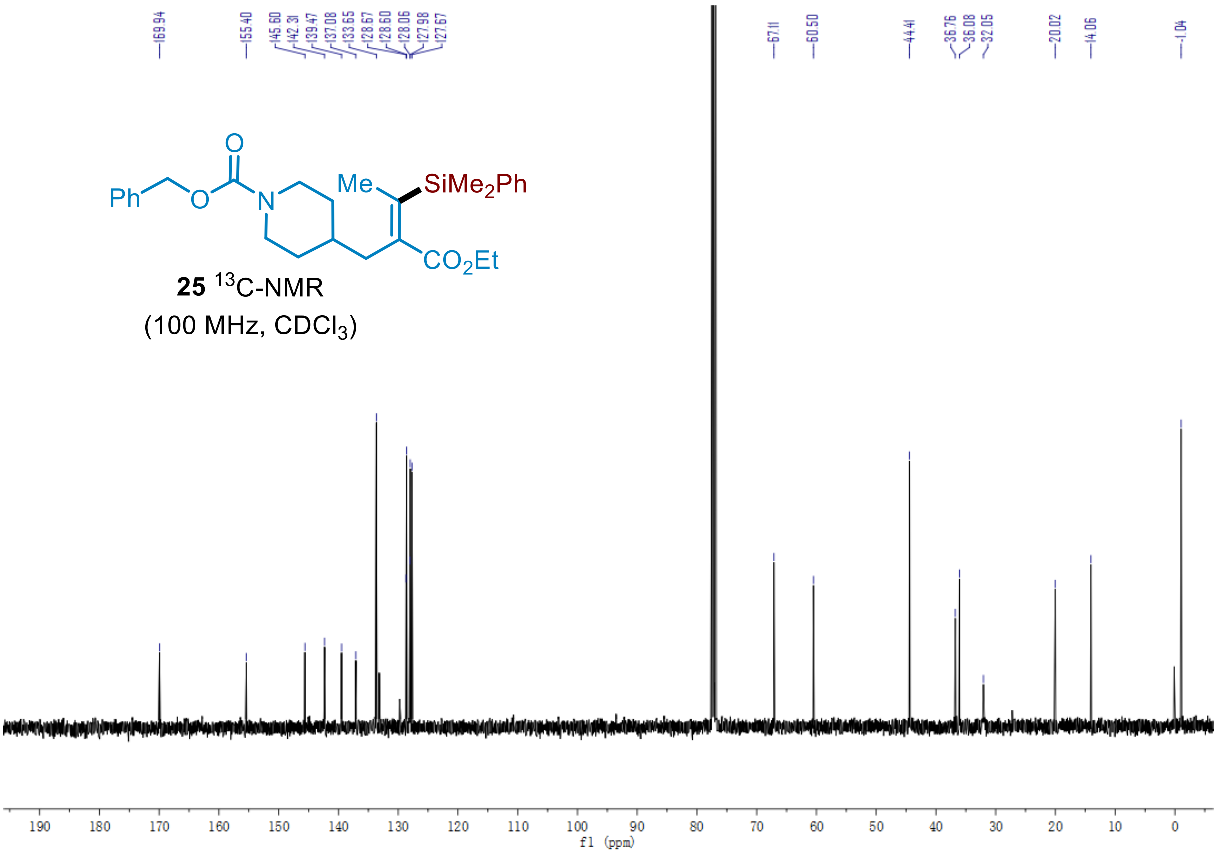

Supplementary Figure 140. **^{13}C-NMR** (100 MHz, $CDCl_3$, 298K) of **25**

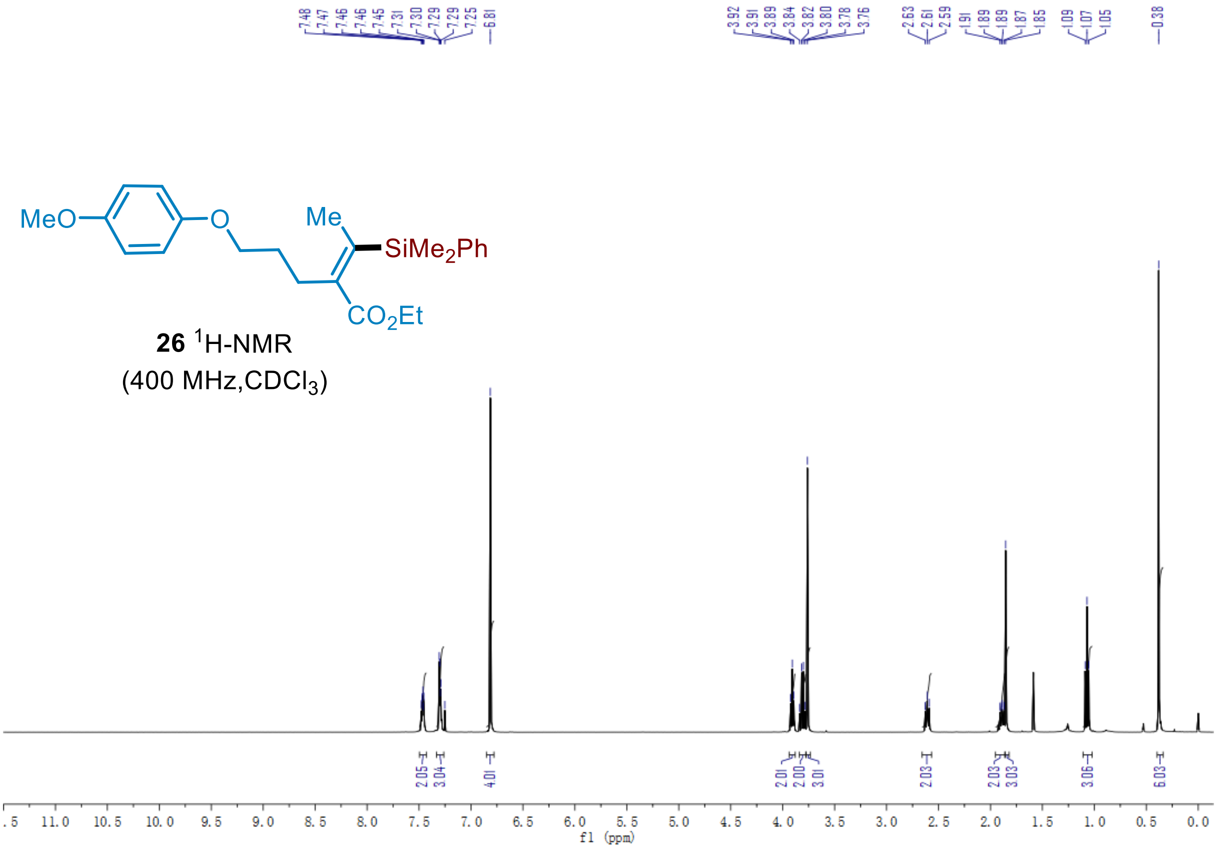

Supplementary Figure 141. **^{1}H-NMR** (400 MHz, $CDCl_3$, 298K) of **26**

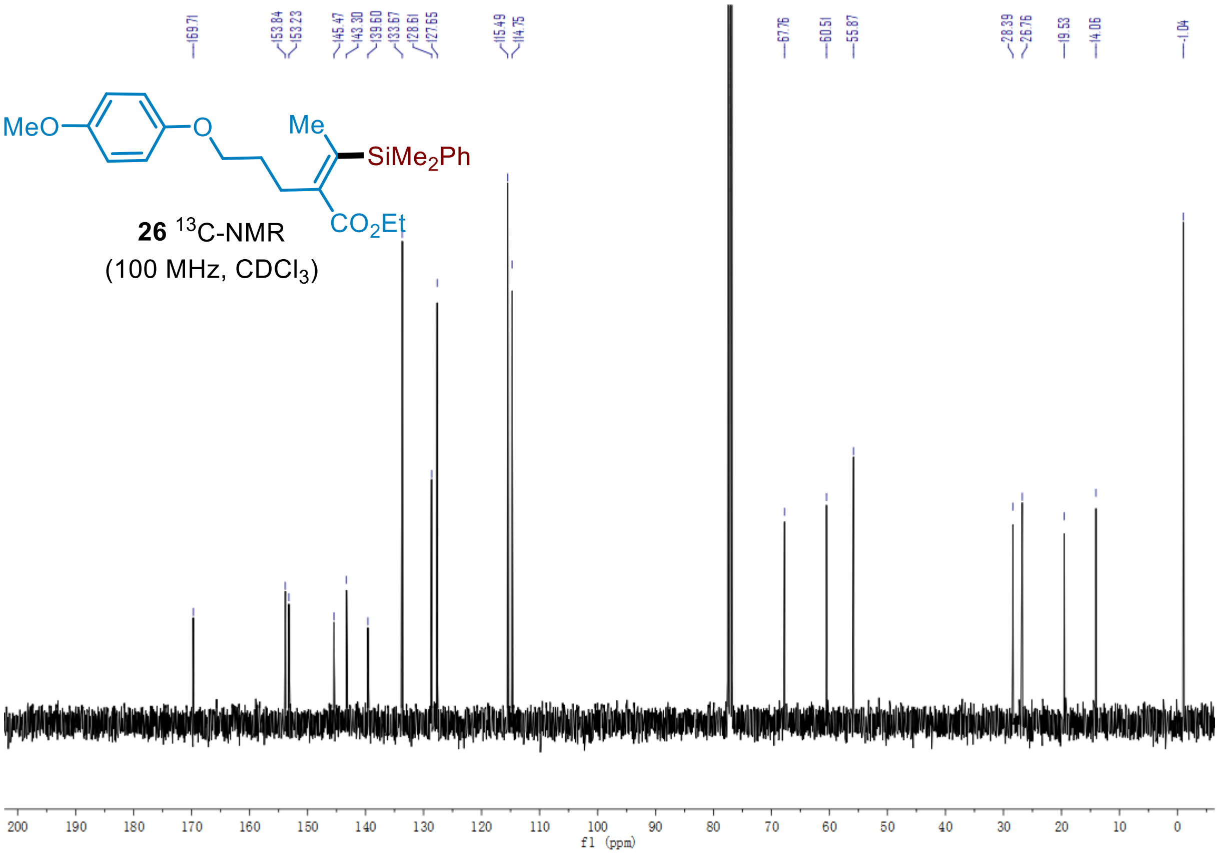

Supplementary Figure 142. **^{13}C-NMR** (100 MHz, $CDCl_3$, 298K) of **26**

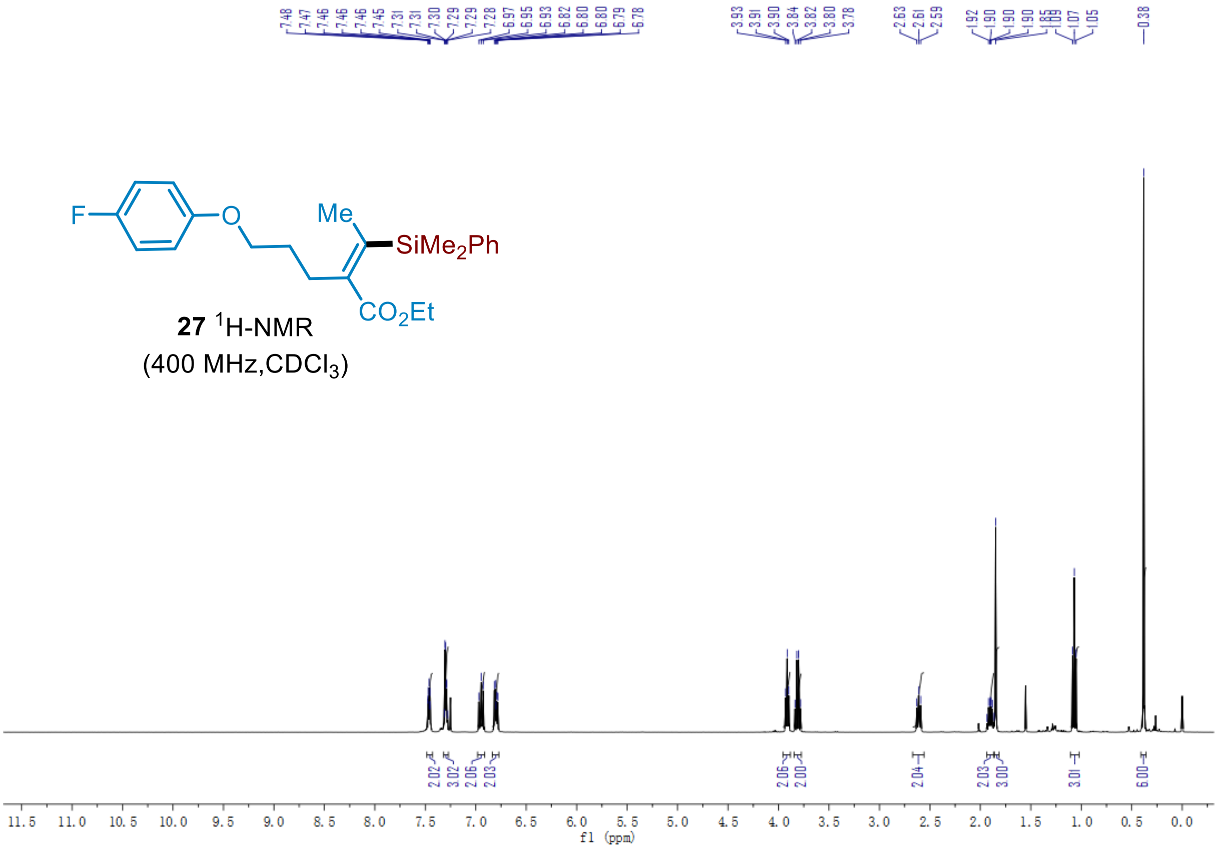

Supplementary Figure 143. **^{1}H-NMR** (400 MHz, $CDCl_3$, 298K) of **27**

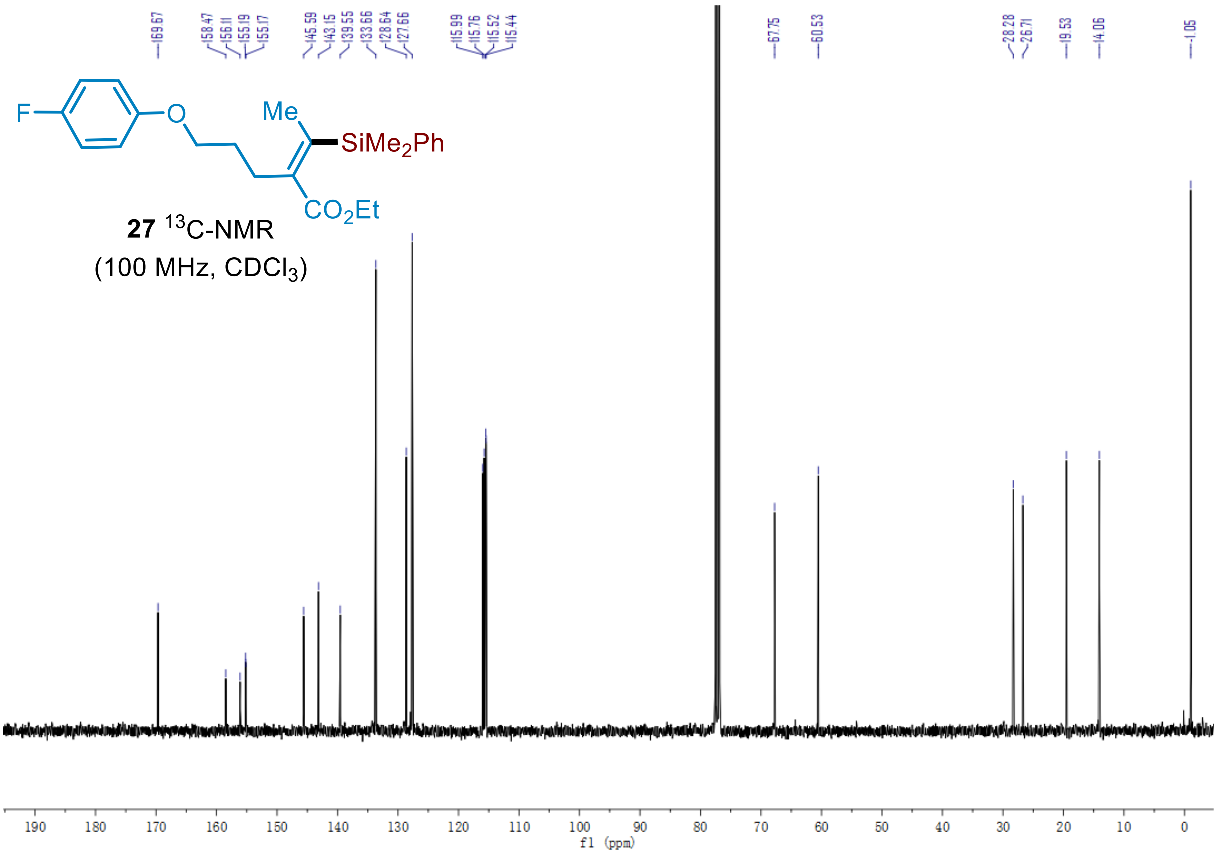

Supplementary Figure 144. **^{13}C-NMR** (100 MHz, $CDCl_3$, 298K) of **27**

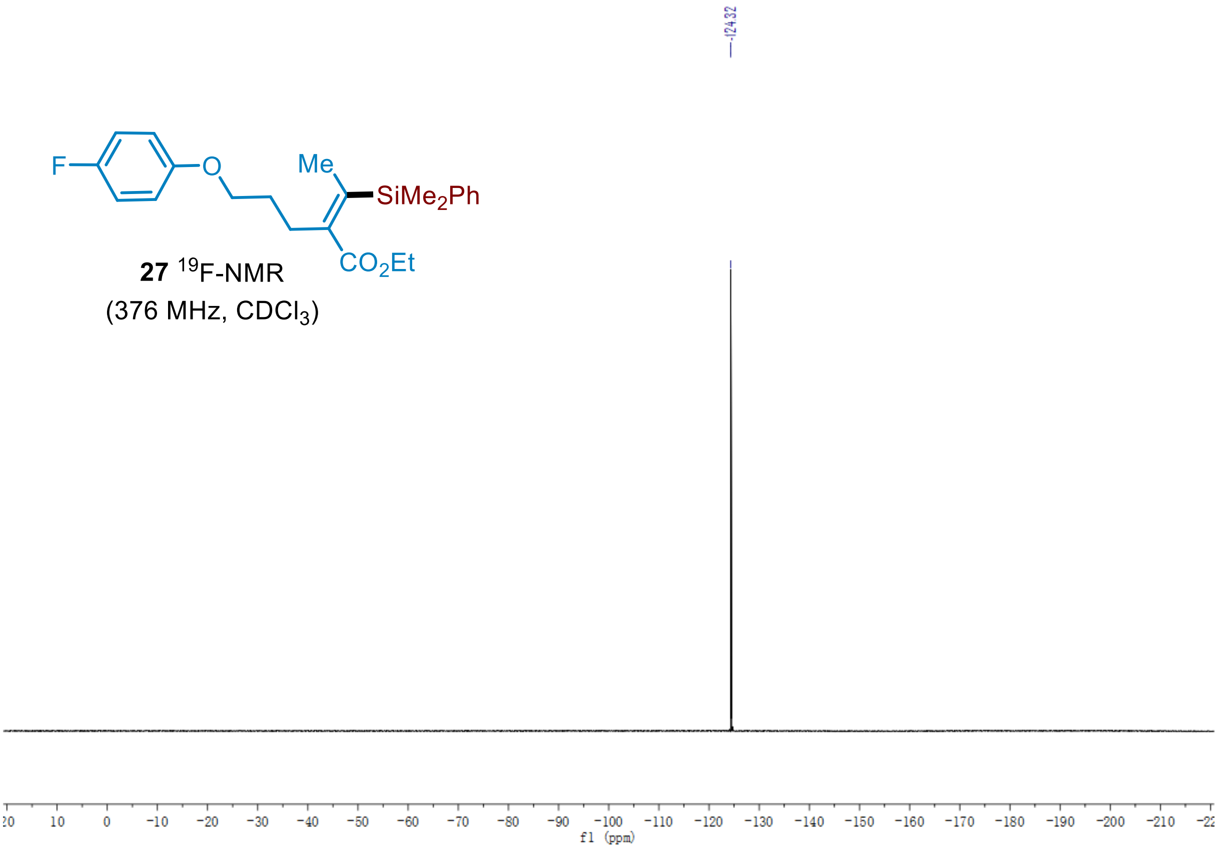

Supplementary Figure 145. **^{19}F-NMR** (376 MHz, $CDCl_3$, 298K) of **27**

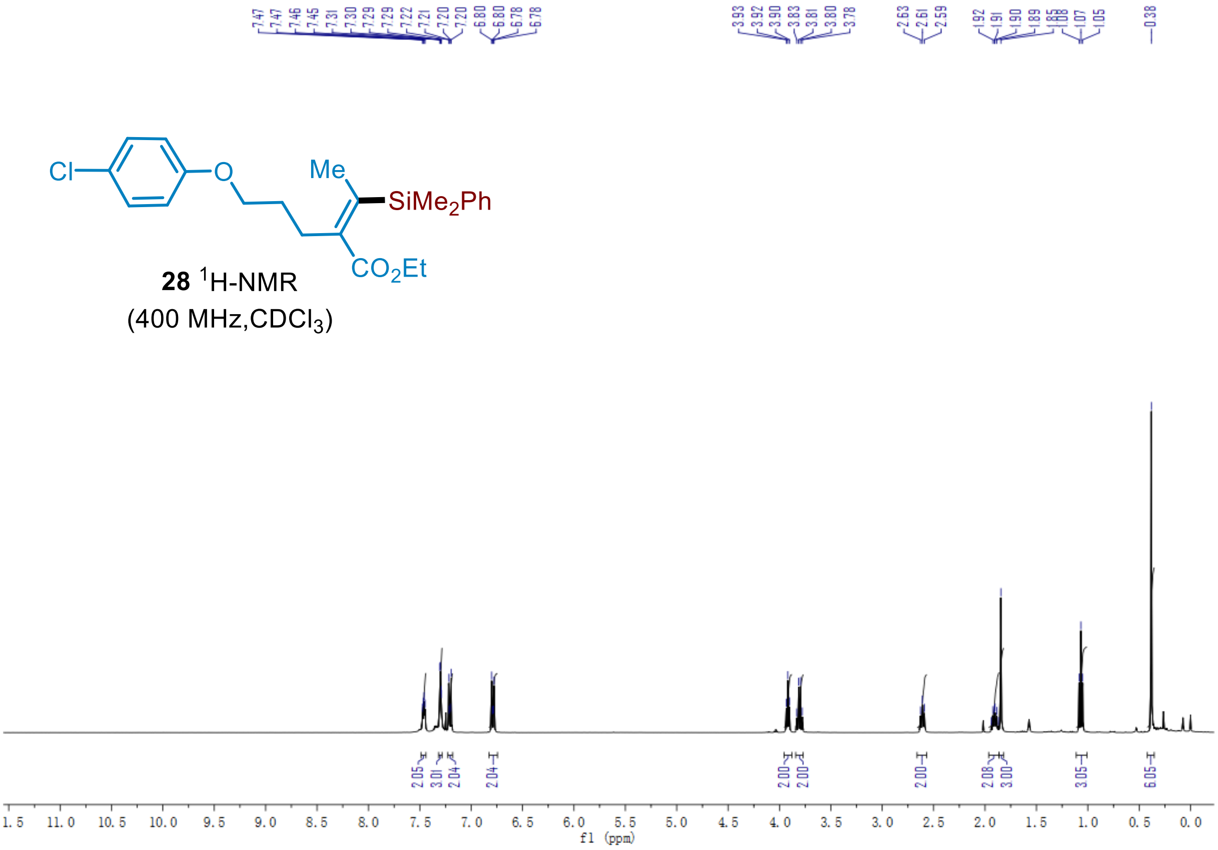

Supplementary Figure 146. **^{1}H-NMR** (400 MHz, $CDCl_3$, 298K) of **28**

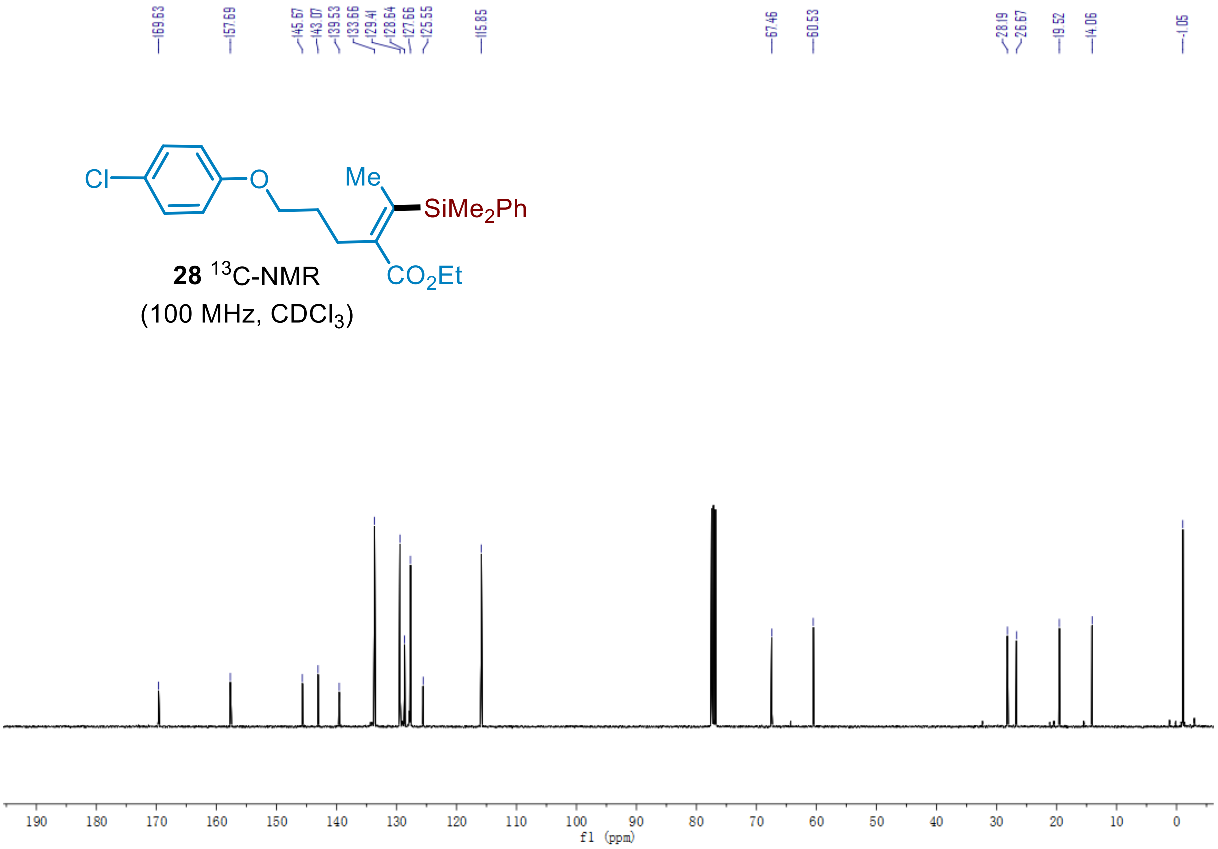

Supplementary Figure 147. **^{13}C-NMR** (100 MHz, $CDCl_3$, 298K) of **28**

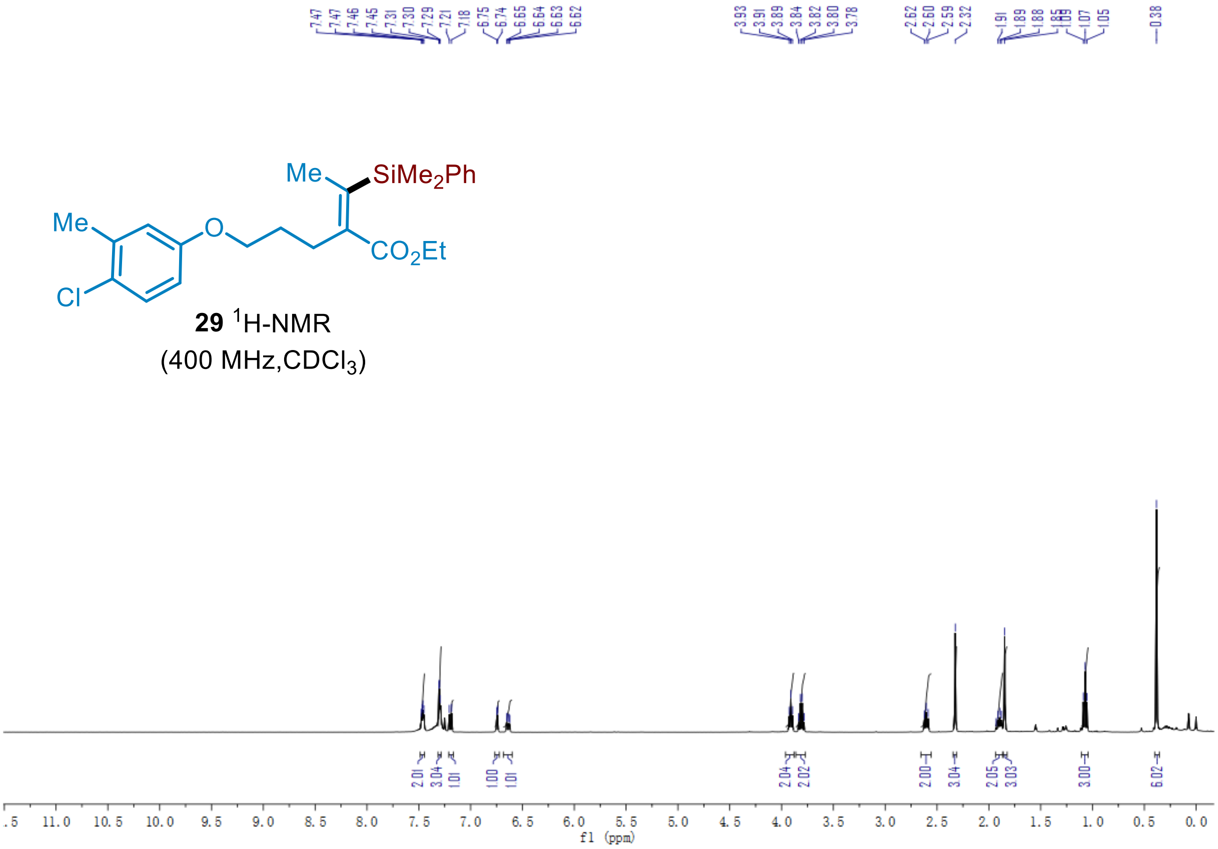

Supplementary Figure 148. **^{1}H-NMR** (400 MHz, $CDCl_3$, 298K) of **29**

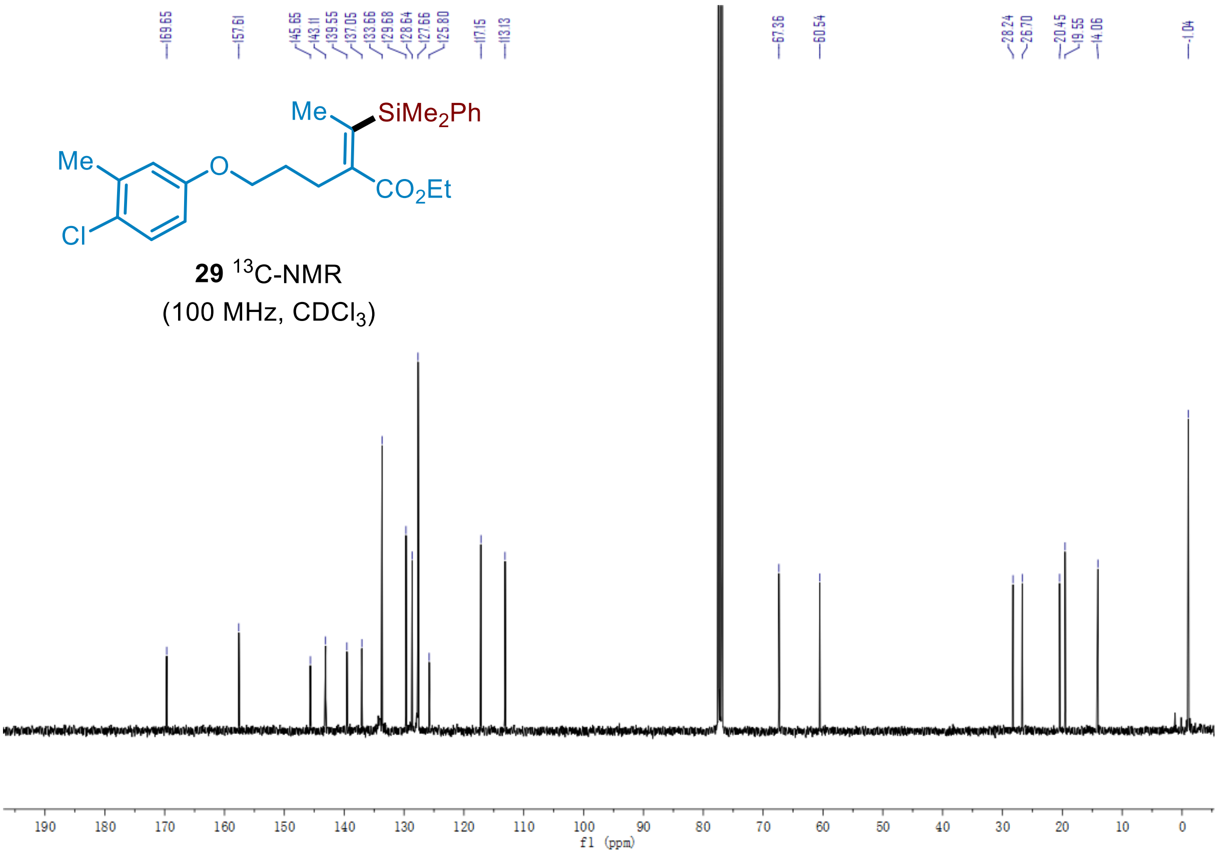

Supplementary Figure 149. **^{13}C-NMR** (100 MHz, $CDCl_3$, 298K) of **29**

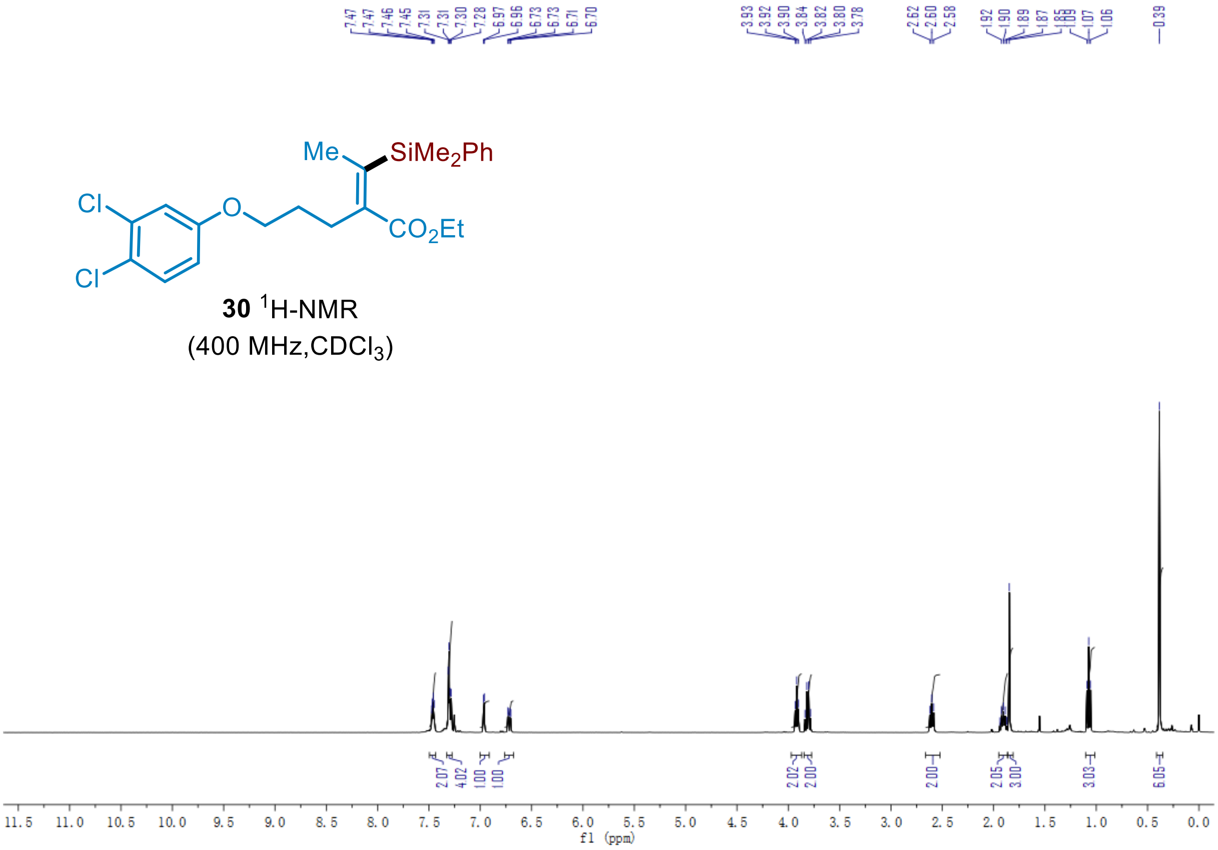

Supplementary Figure 150. **^{1}H-NMR** (400 MHz, $CDCl_3$, 298K) of **30**

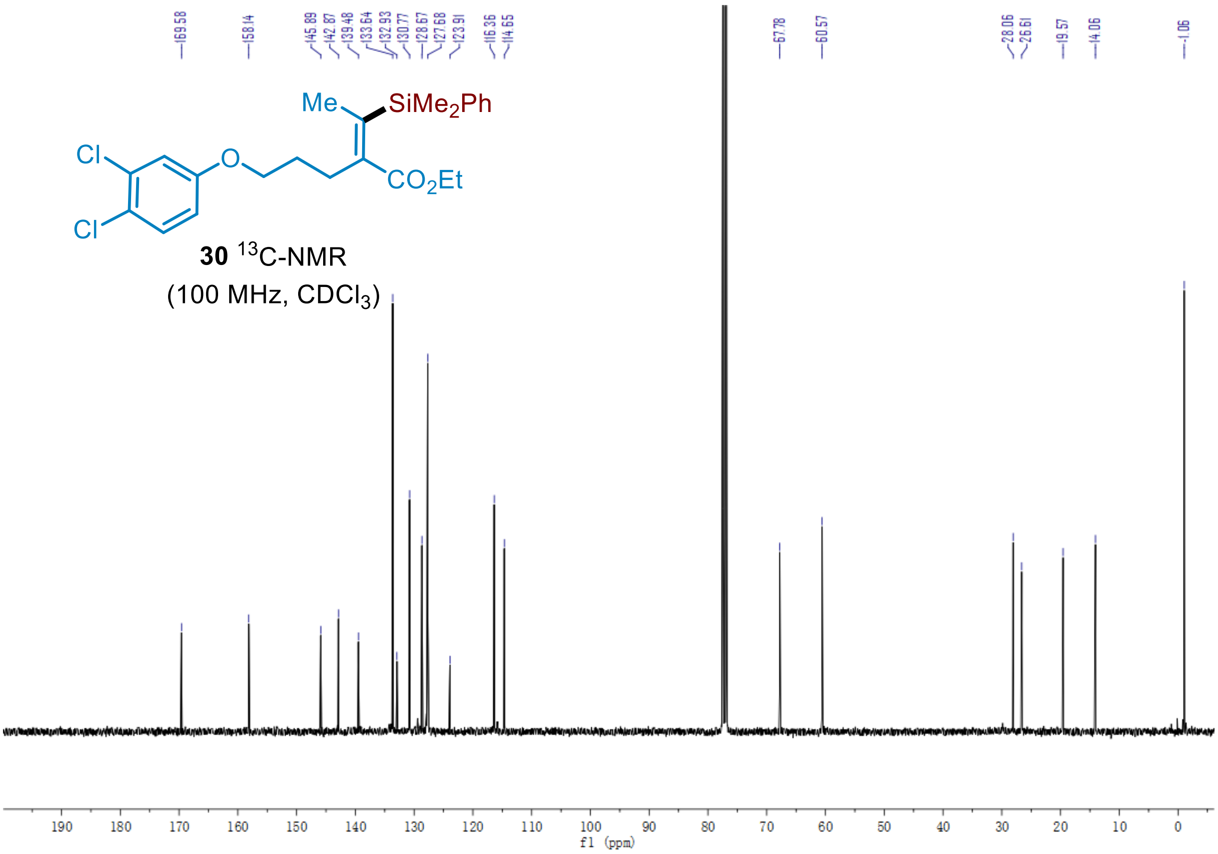

Supplementary Figure 151. **^{13}C-NMR** (100 MHz, $CDCl_3$, 298K) of **30**

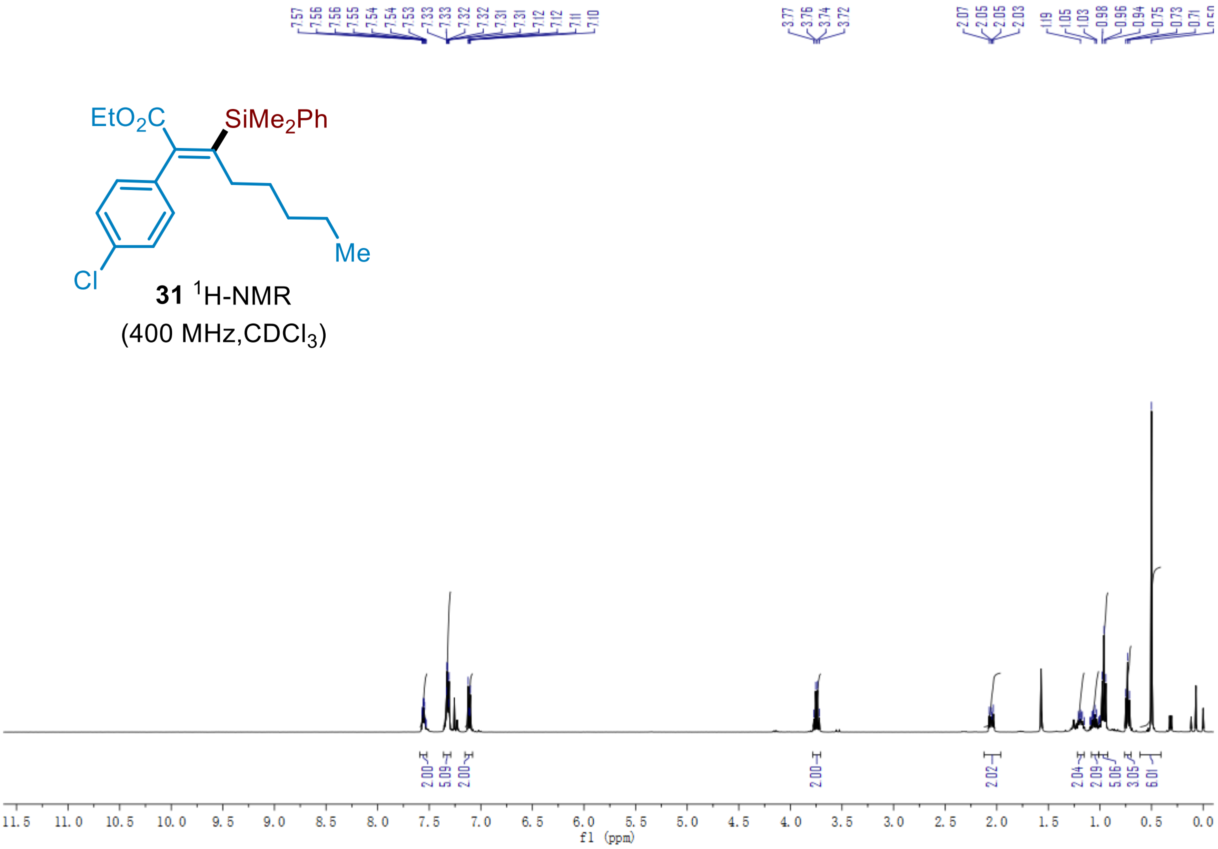

Supplementary Figure 152. **^{1}H-NMR** (400 MHz, $CDCl_3$, 298K) of **31**

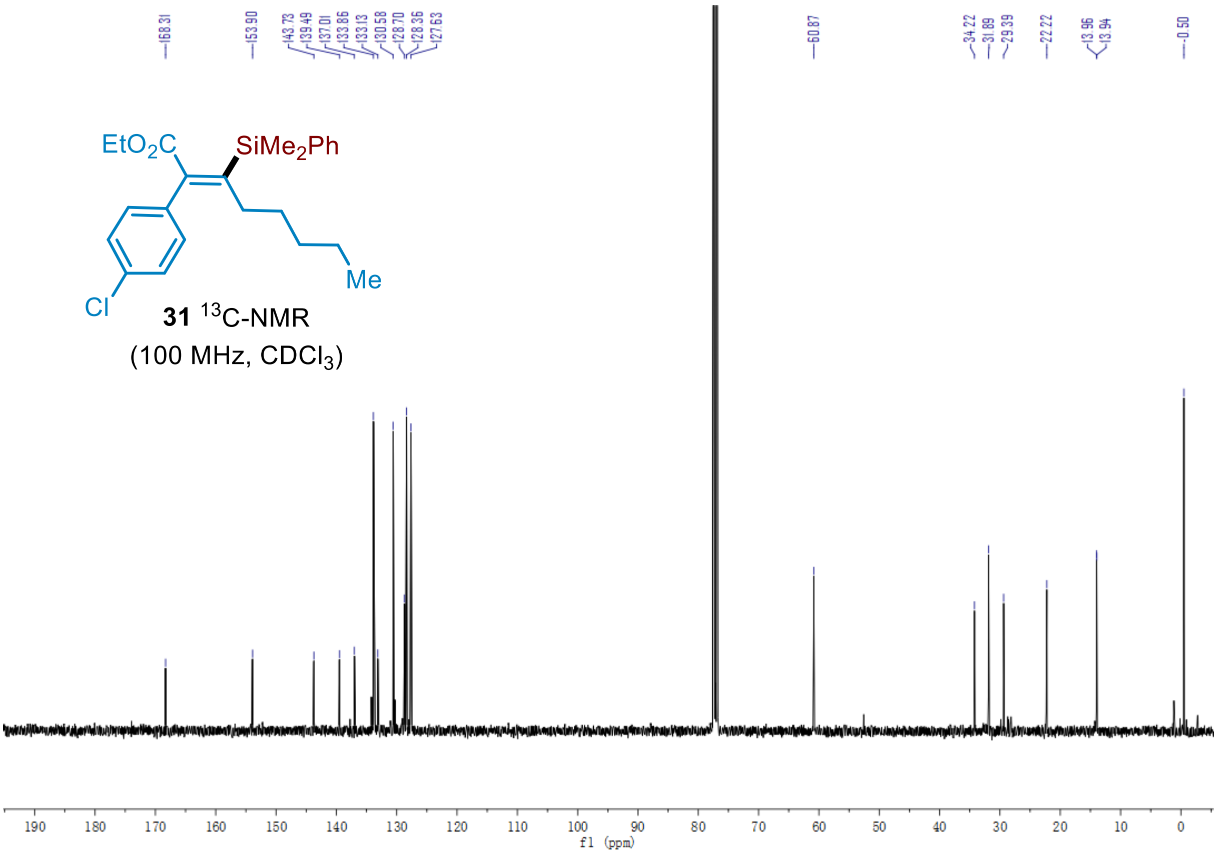

Supplementary Figure 153. **^{13}C-NMR** (100 MHz, $CDCl_3$, 298K) of **31**

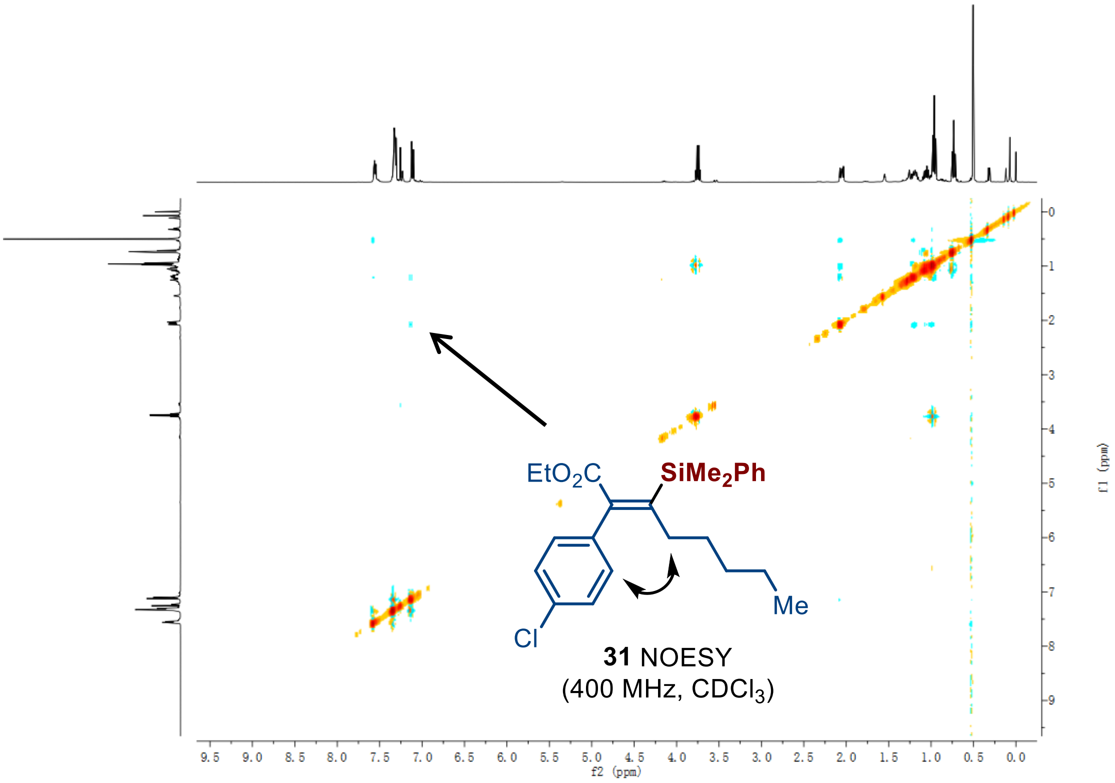

Supplementary Figure 154.NOESY of **31**

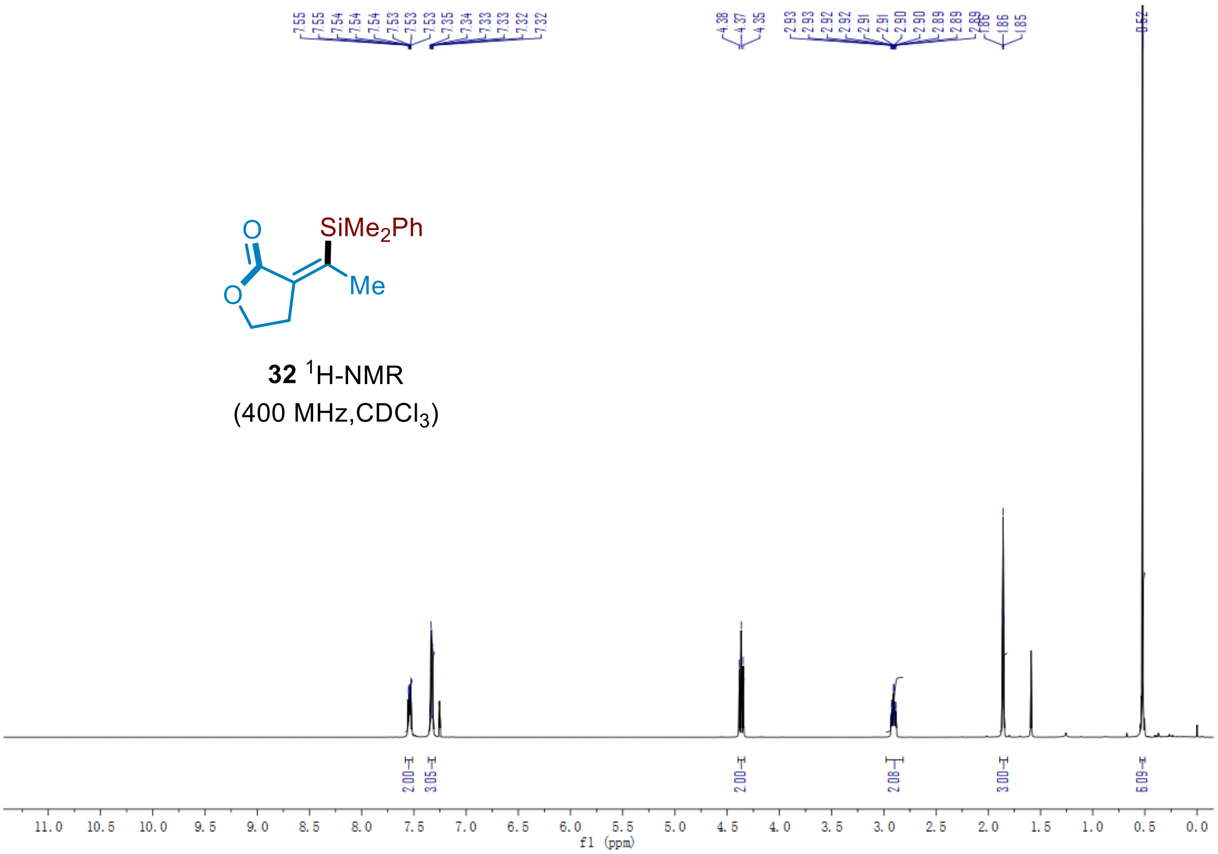

Supplementary Figure 155. **^{1}H-NMR** (400 MHz, $CDCl_3$, 298K) of **32**

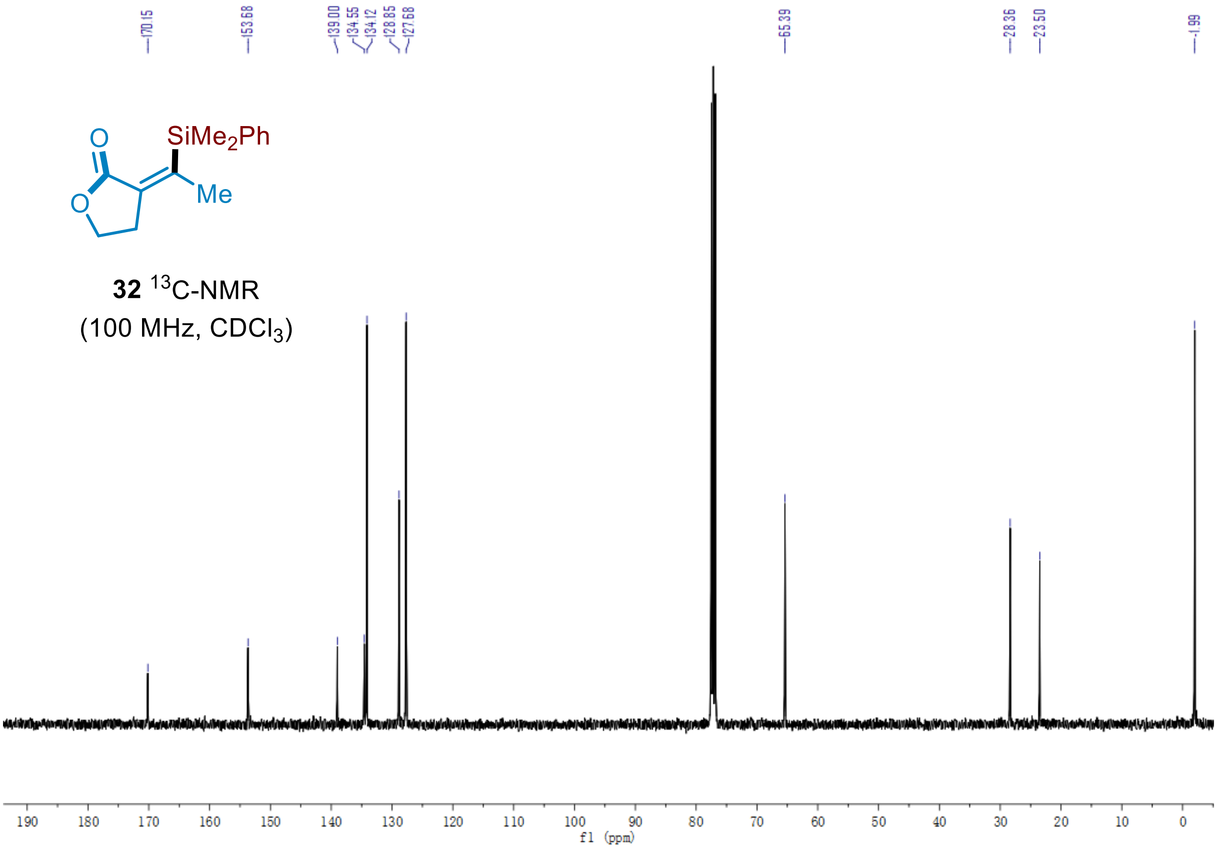

Supplementary Figure 156. **^{13}C-NMR** (100 MHz, $CDCl_3$, 298K) of **32**

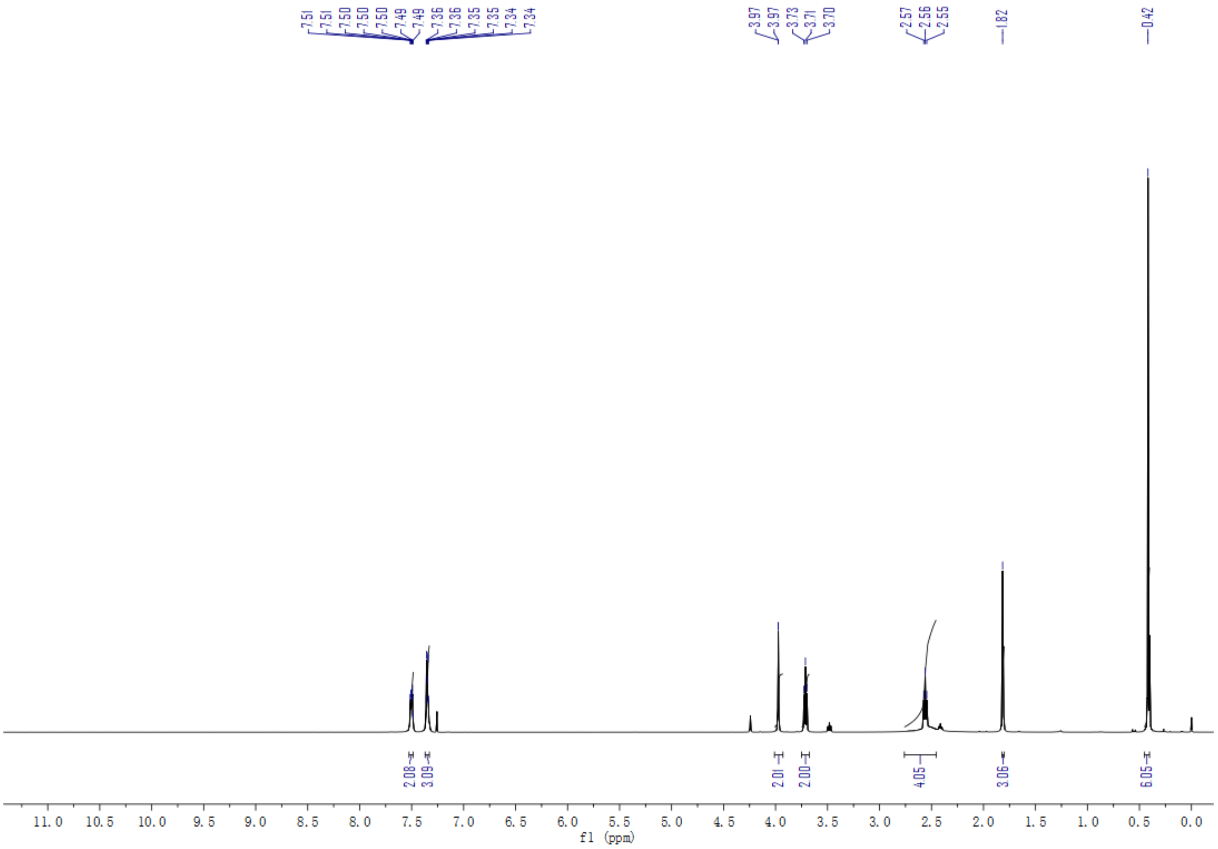

Supplementary Figure 157. **^{1}H-NMR** (400 MHz, $CDCl_3$, 298K) of **33**

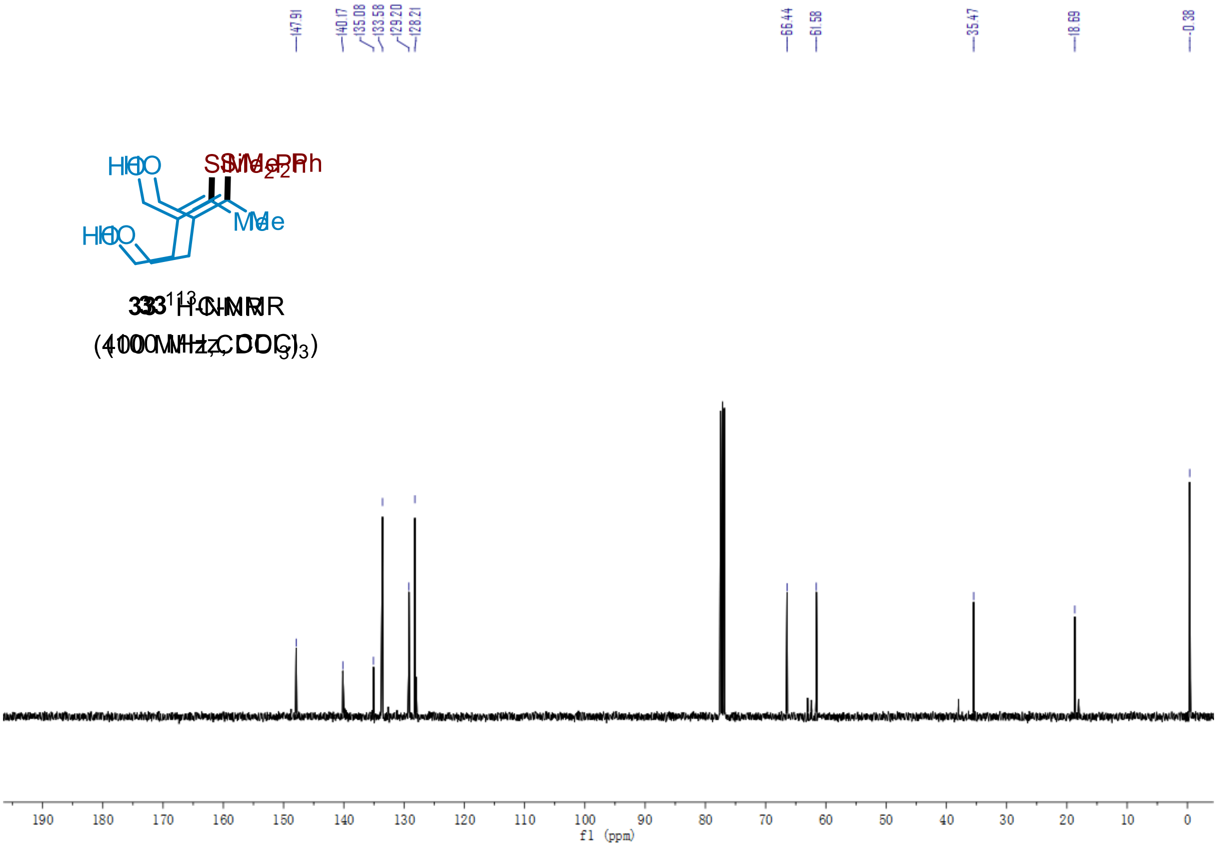

Supplementary Figure 158. **^{13}C-NMR** (100 MHz, $CDCl_3$, 298K) of **33**

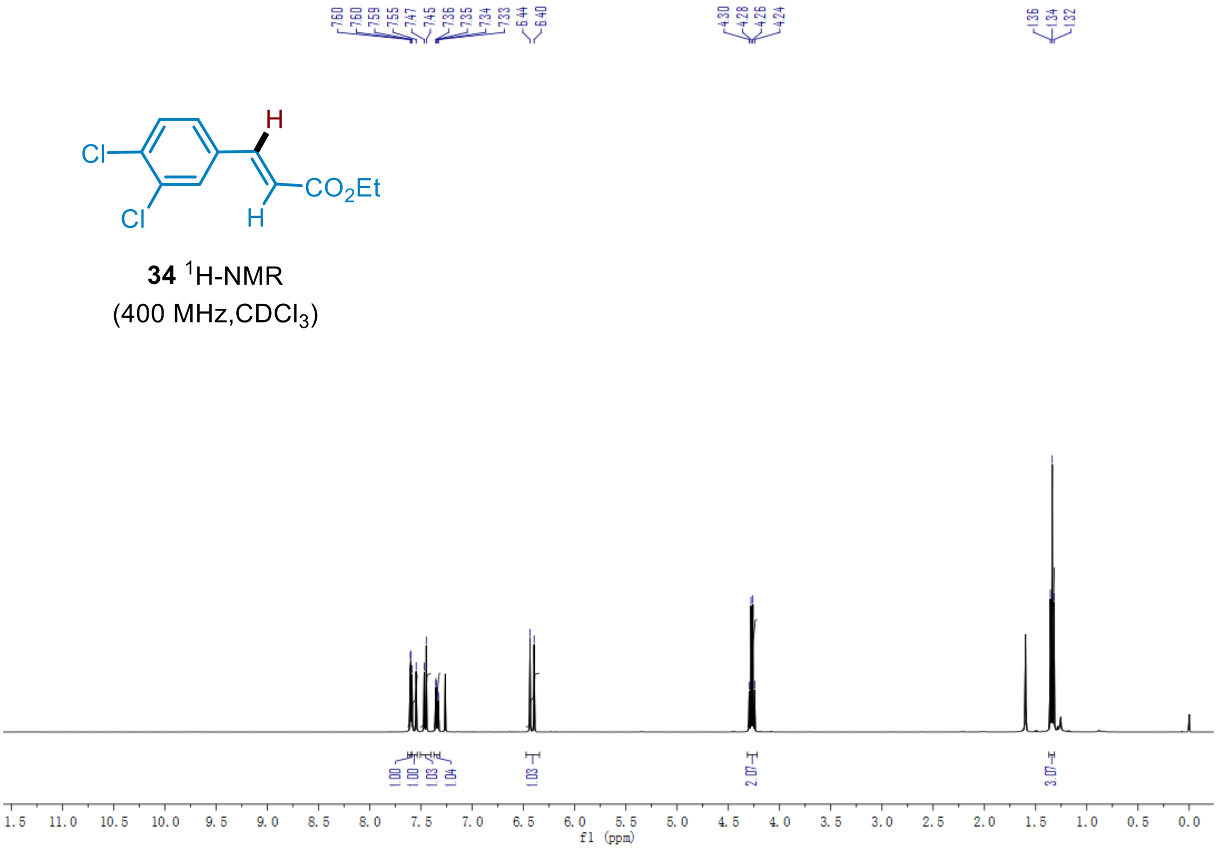

Supplementary Figure 159. **^{1}H-NMR** (400 MHz, $CDCl_3$, 298K) of **34**

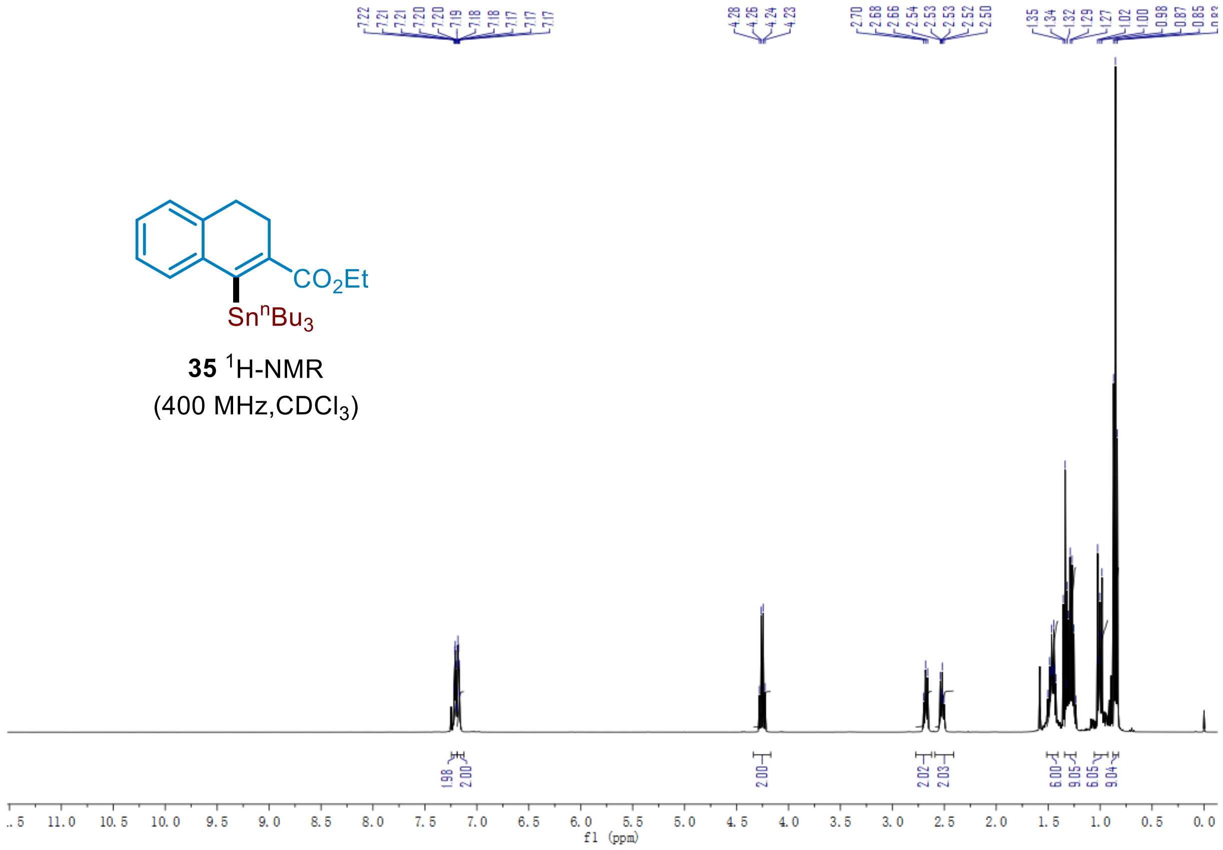

Supplementary Figure 160. **^{1}H-NMR** (400 MHz, $CDCl_3$, 298K) of **35**

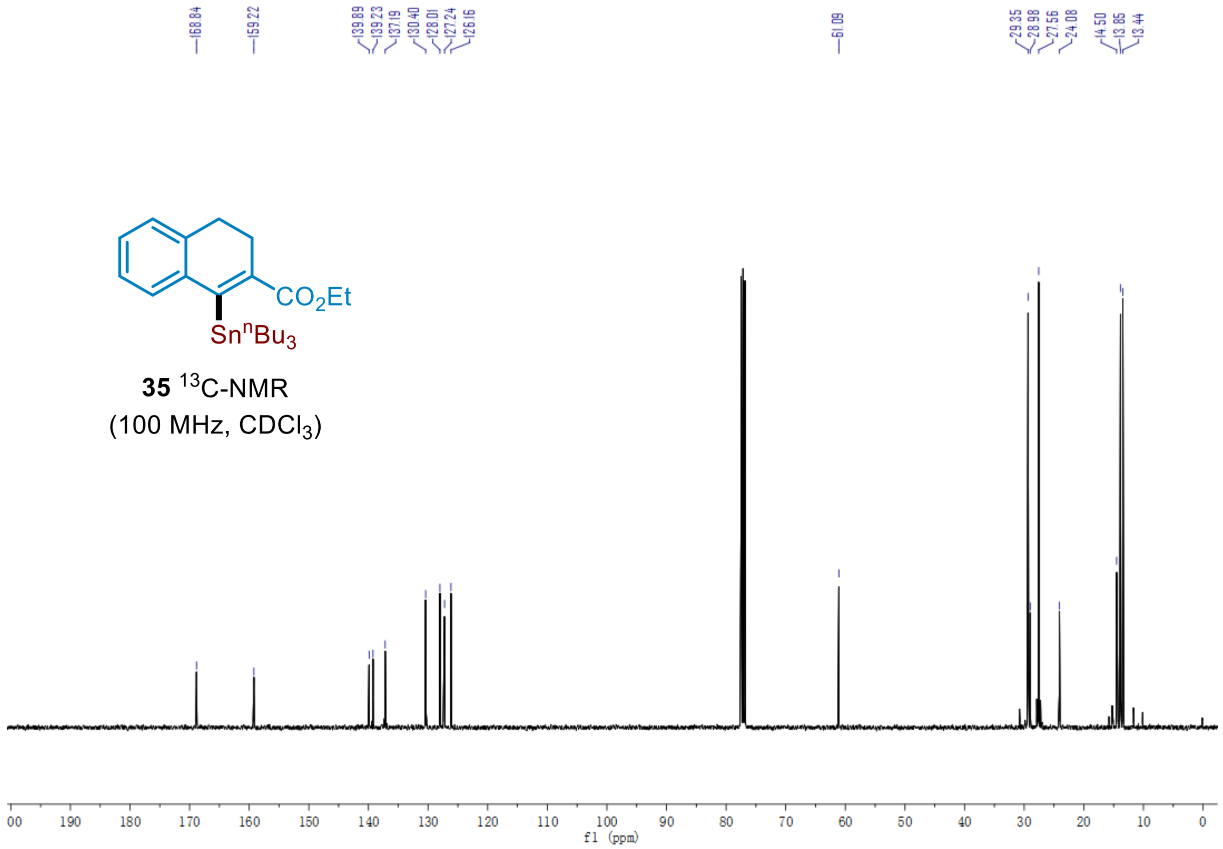

Supplementary Figure 161. **^{13}C-NMR** (100 MHz, $CDCl_3$, 298K) of **35**

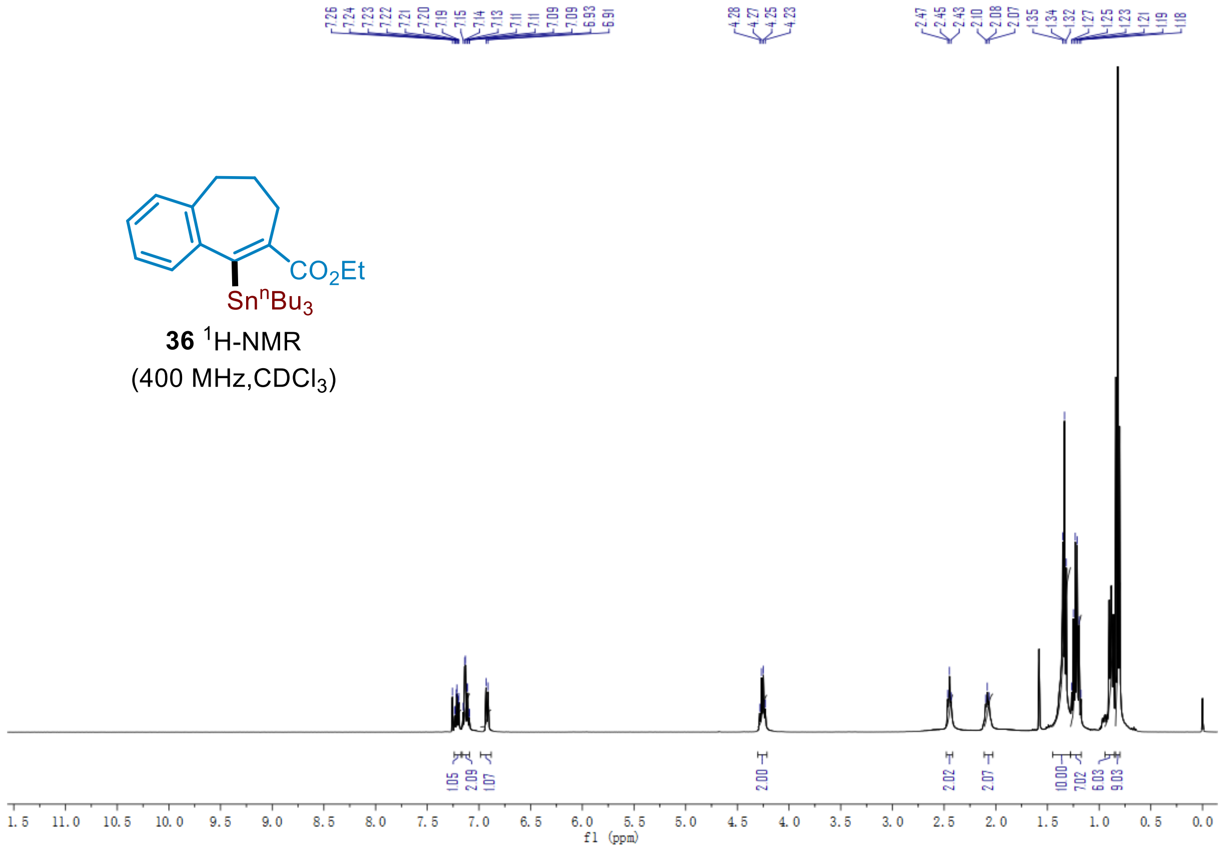

Supplementary Figure 162. **^{1}H-NMR** (400 MHz, $CDCl_3$, 298K) of **36**

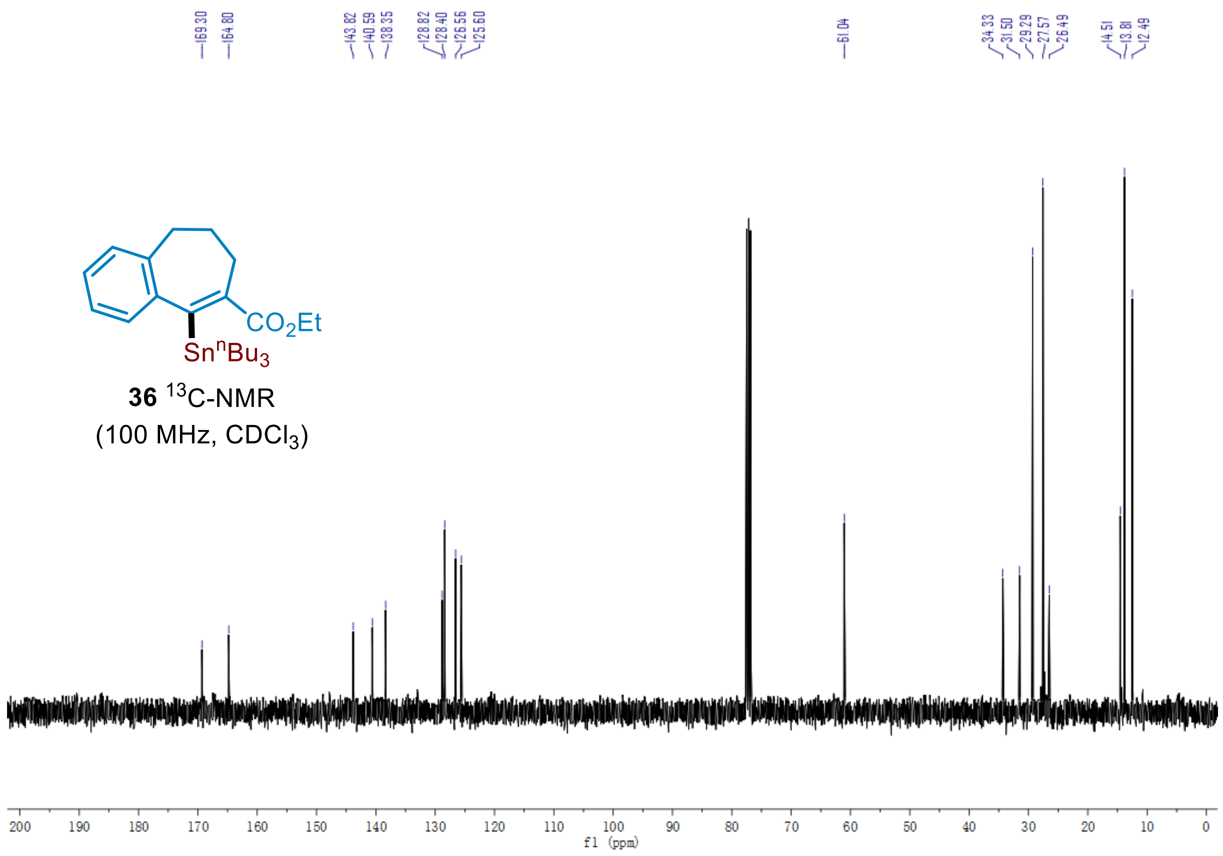

Supplementary Figure 163. **^{13}C-NMR** (100 MHz, $CDCl_3$, 298K) of **36**

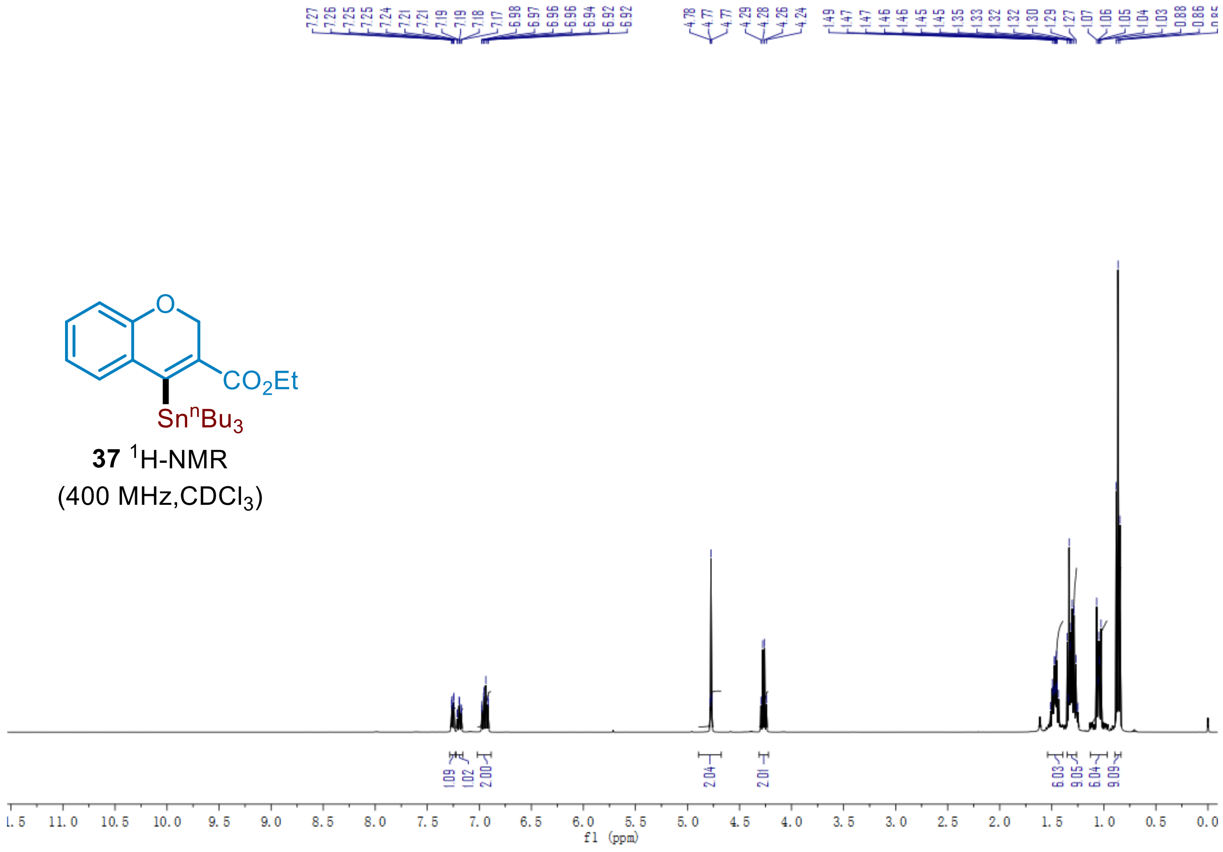

Supplementary Figure 164. **^{1}H-NMR** (400 MHz, $CDCl_3$, 298K) of **37**

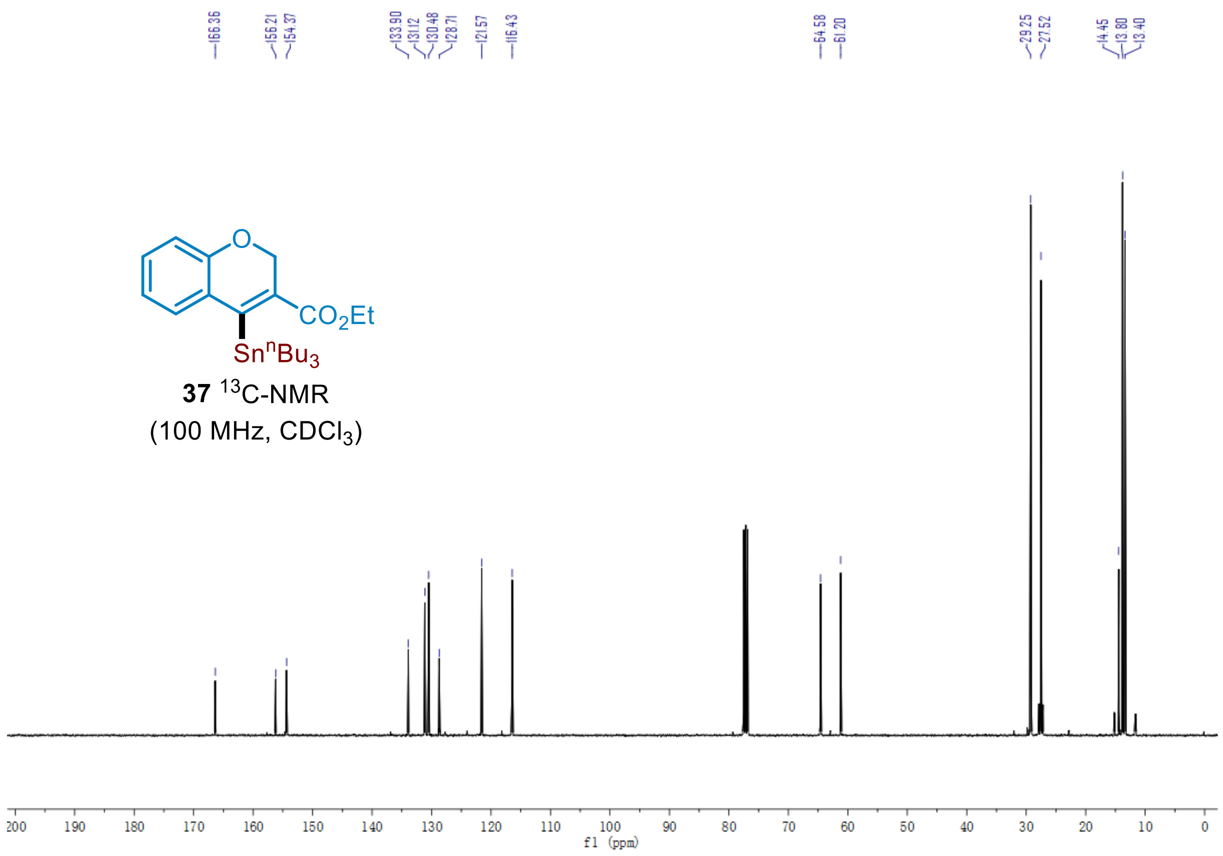

Supplementary Figure 165. **^{13}C-NMR** (100 MHz, $CDCl_3$, 298K) of **37**

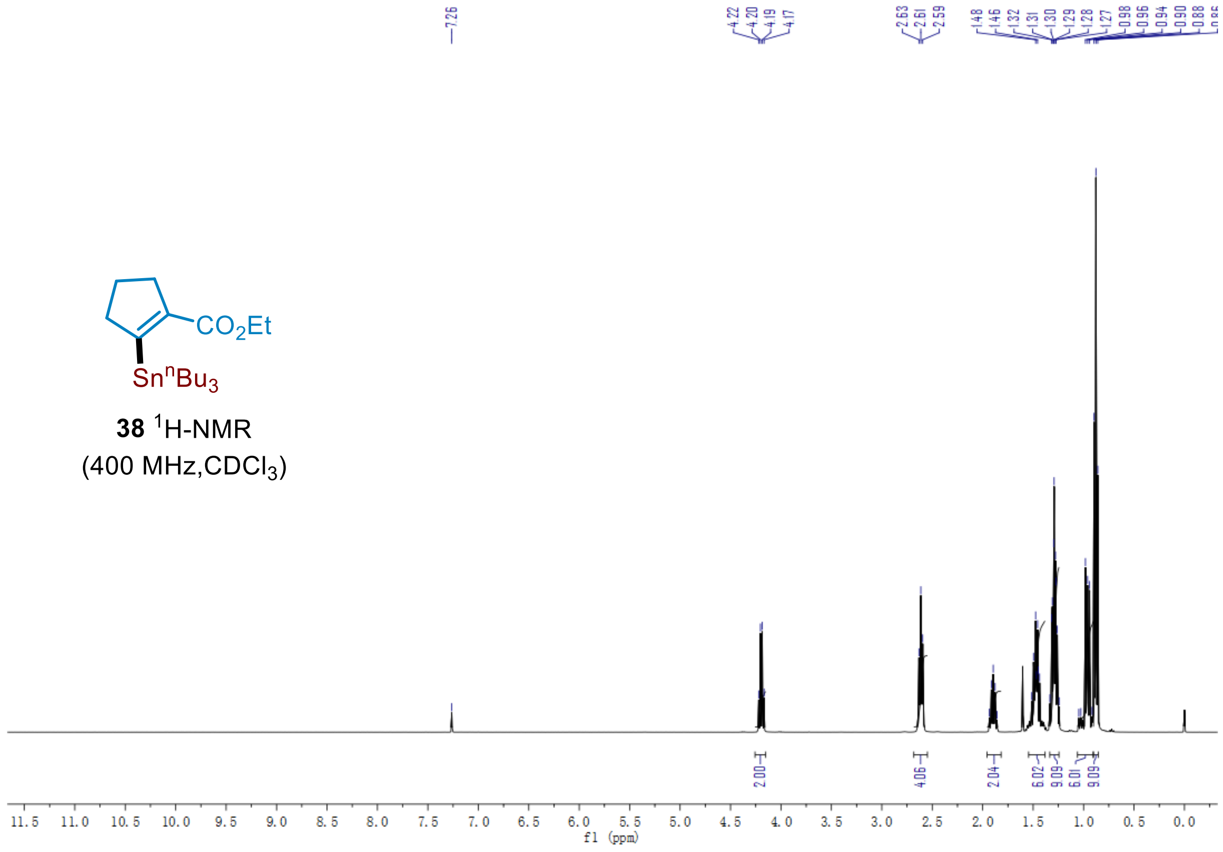

Supplementary Figure 166. **^{1}H-NMR** (400 MHz, $CDCl_3$, 298K) of **38**

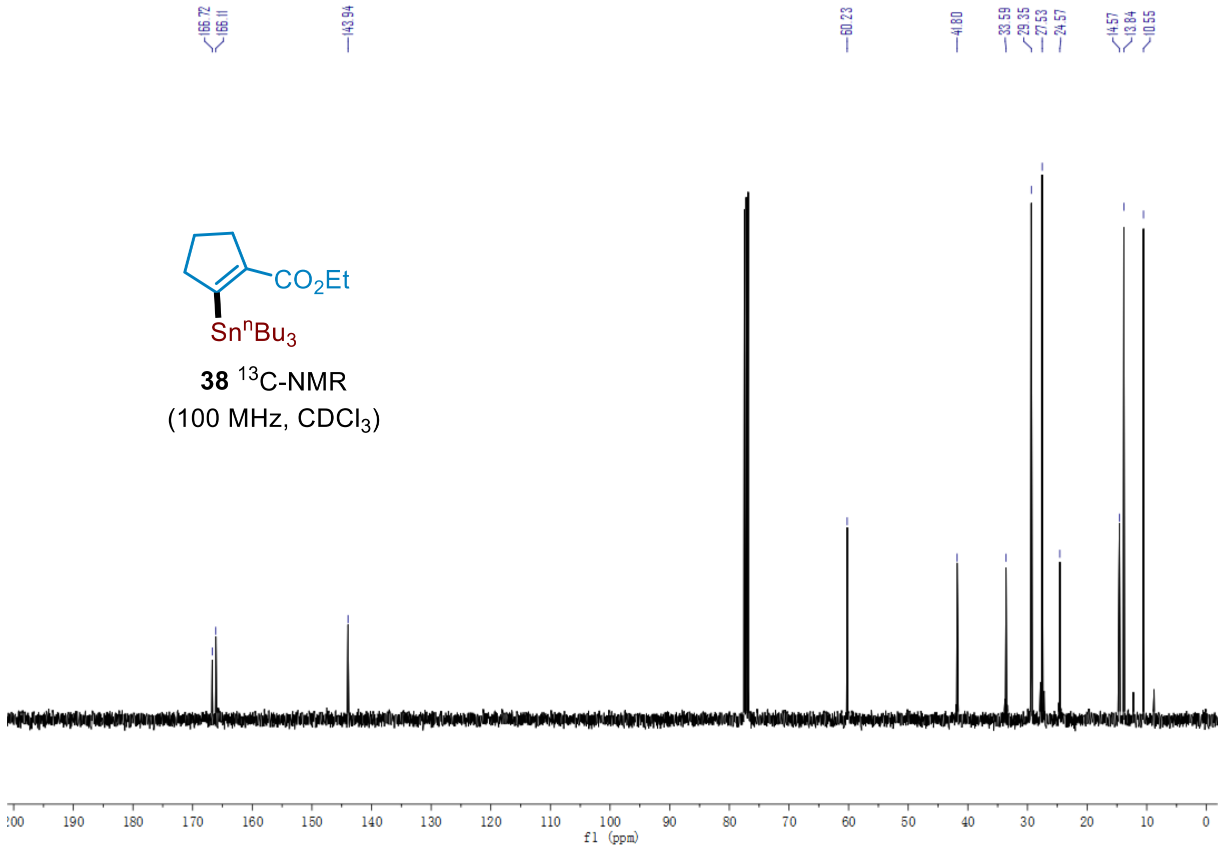

Supplementary Figure 167. **^{13}C-NMR** (100 MHz, $CDCl_3$, 298K) of **38**

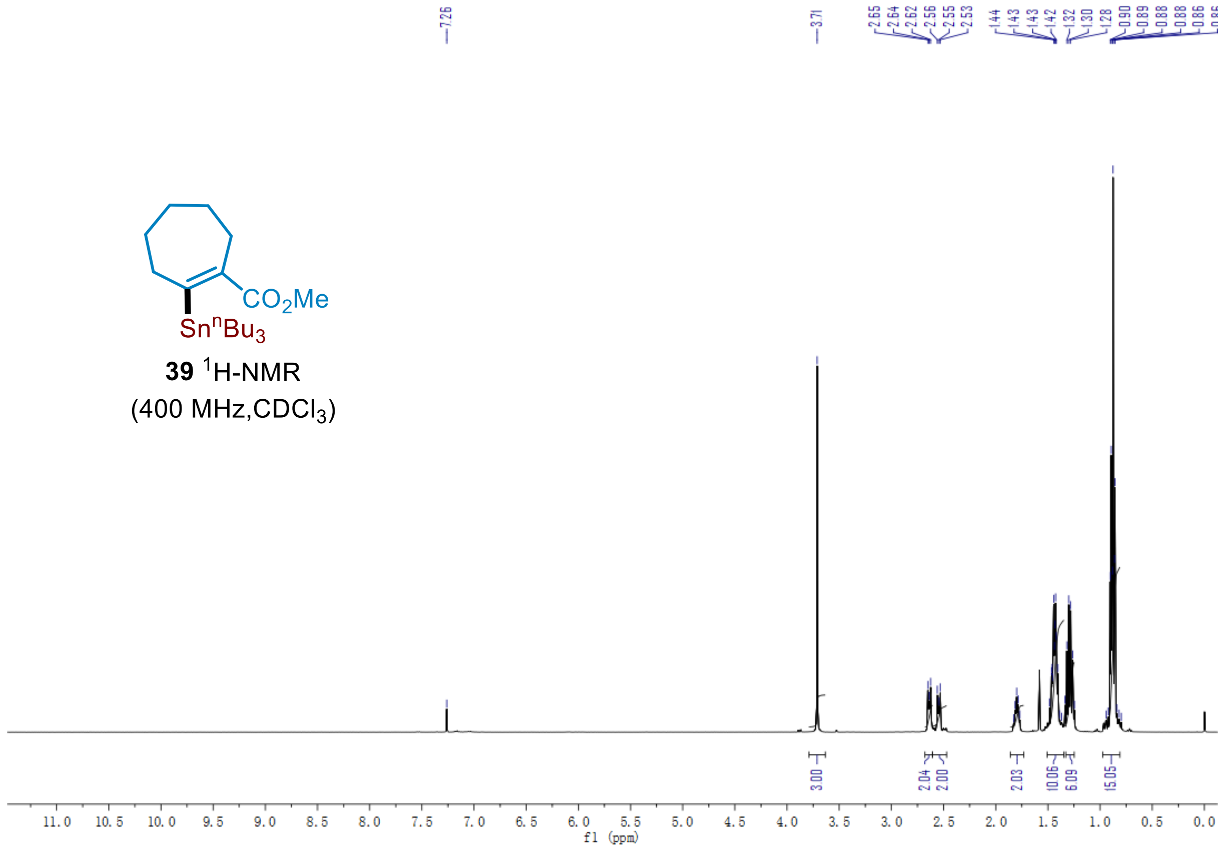

Supplementary Figure 168. **^{1}H-NMR** (400 MHz, $CDCl_3$, 298K) of **39**

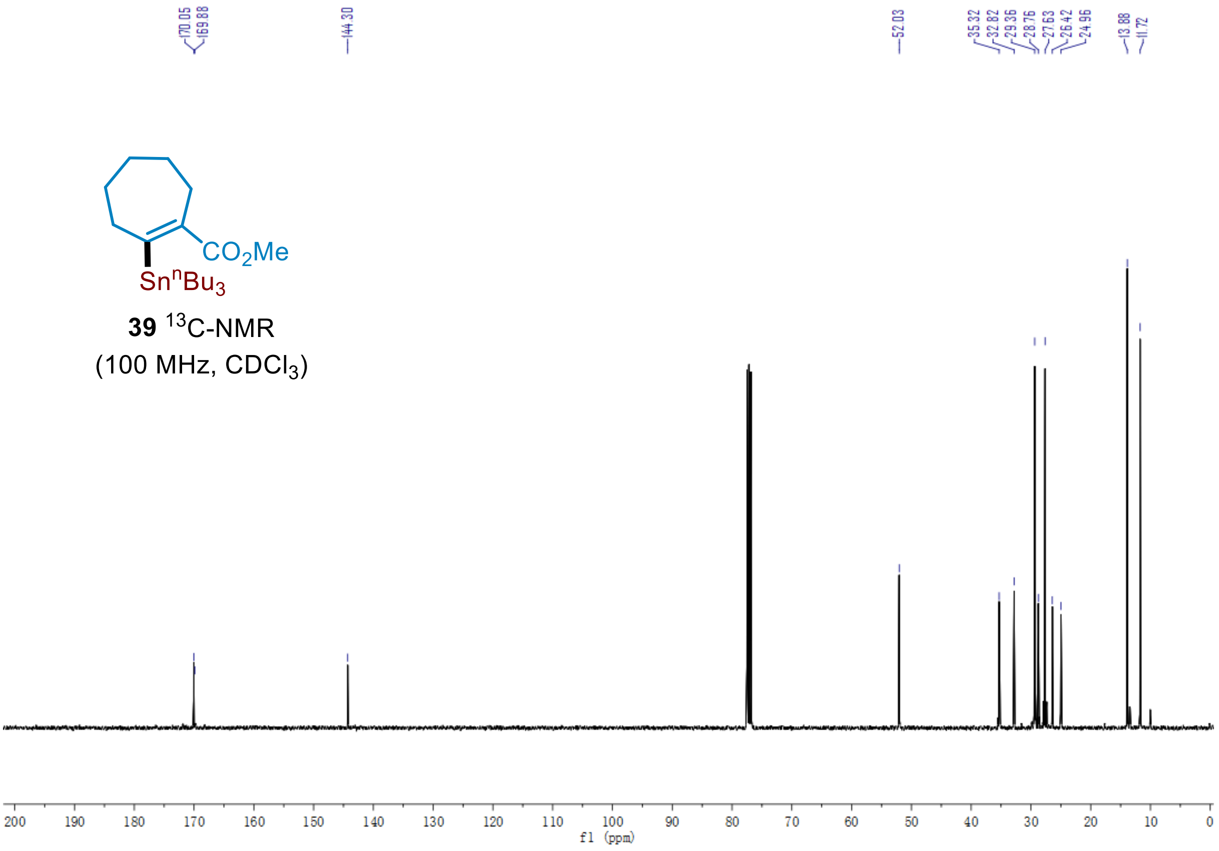

Supplementary Figure 169. **^{13}C-NMR** (100 MHz, $CDCl_3$, 298K) of **39**

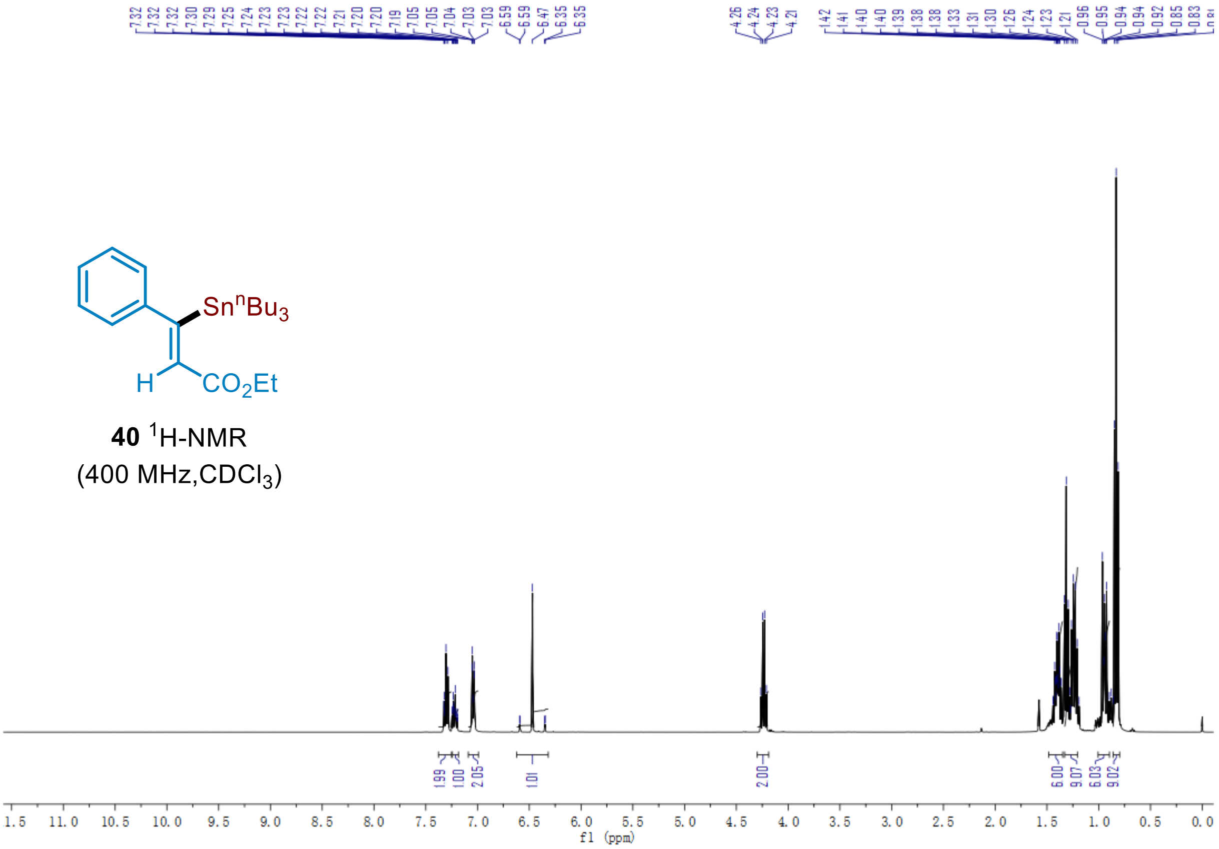

Supplementary Figure 170. **^{1}H-NMR** (400 MHz, $CDCl_3$, 298K) of **40**

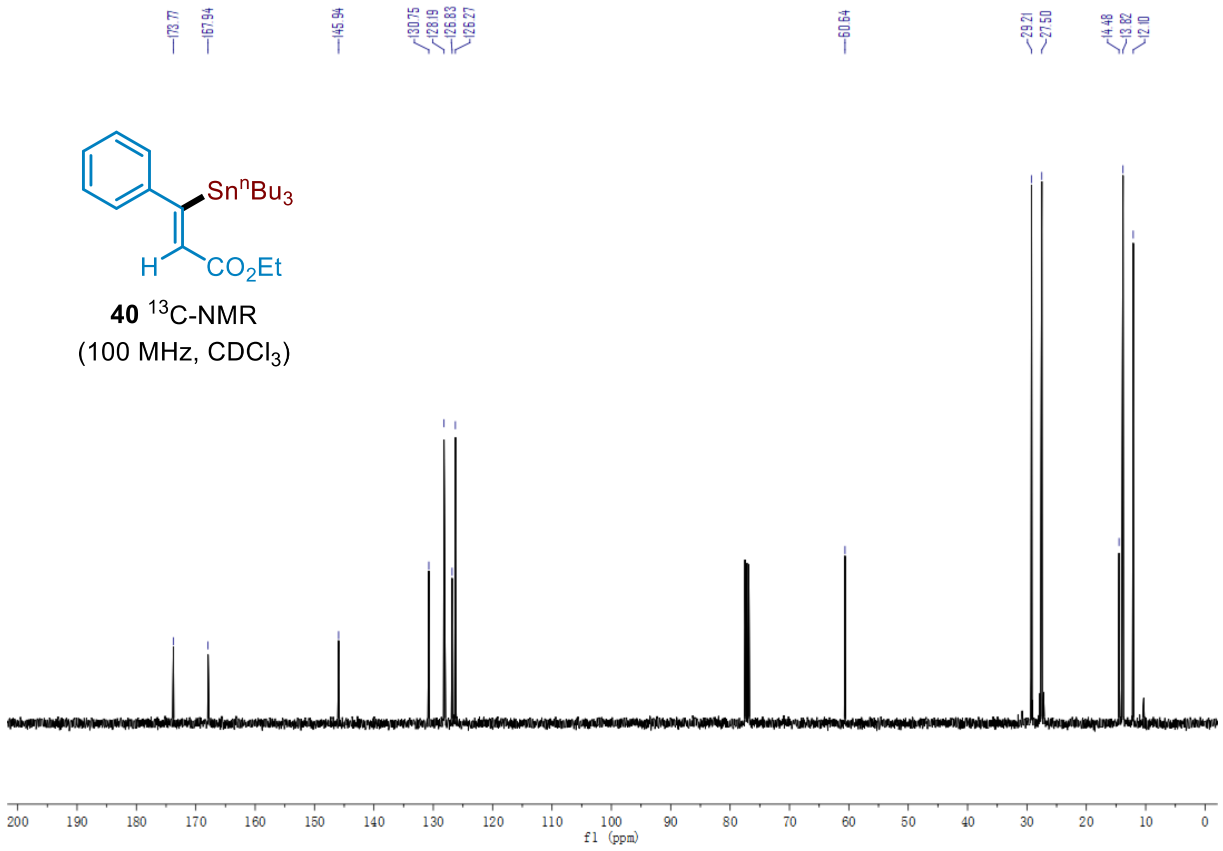

Supplementary Figure 171. **^{13}C-NMR** (100 MHz, CDCl$_3$, 298K) of **40**

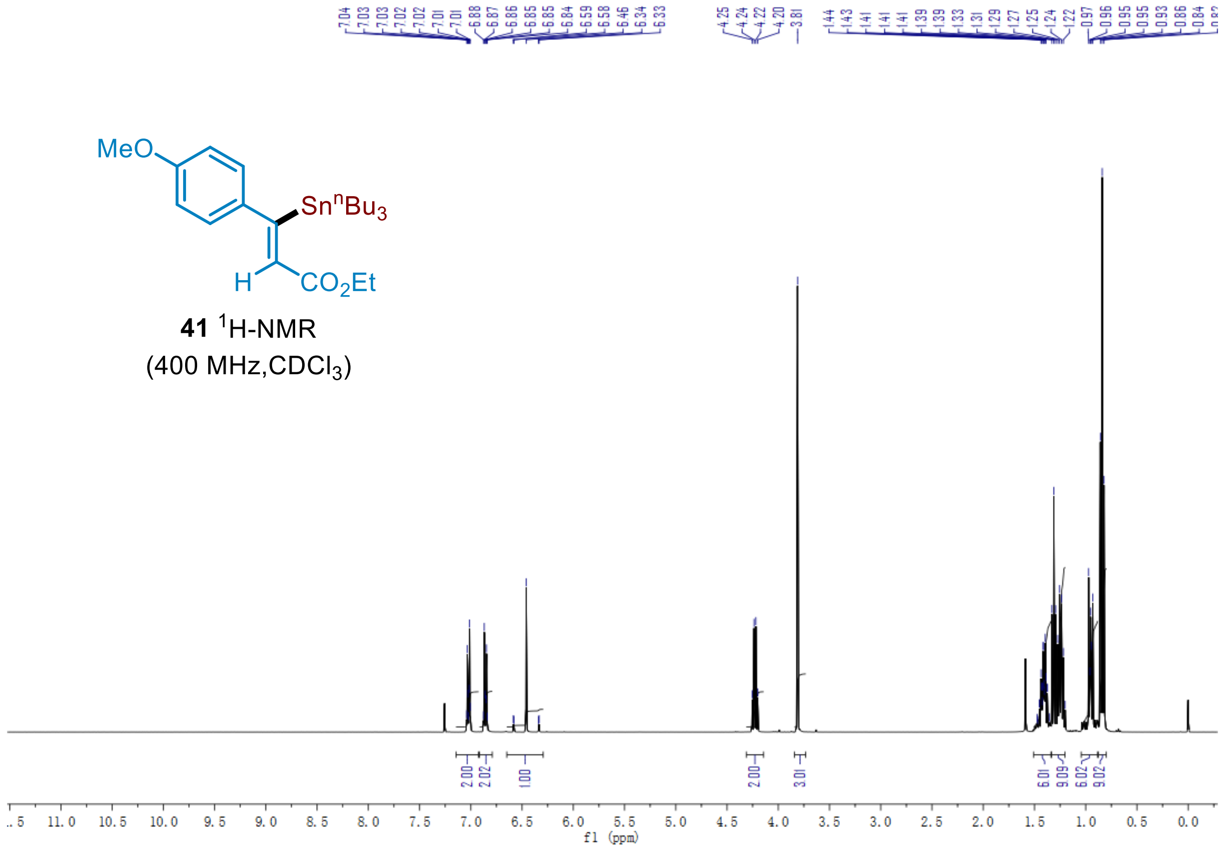

Supplementary Figure 172. **^{1}H-NMR** (400 MHz, CDCl$_3$, 298K) of **41**

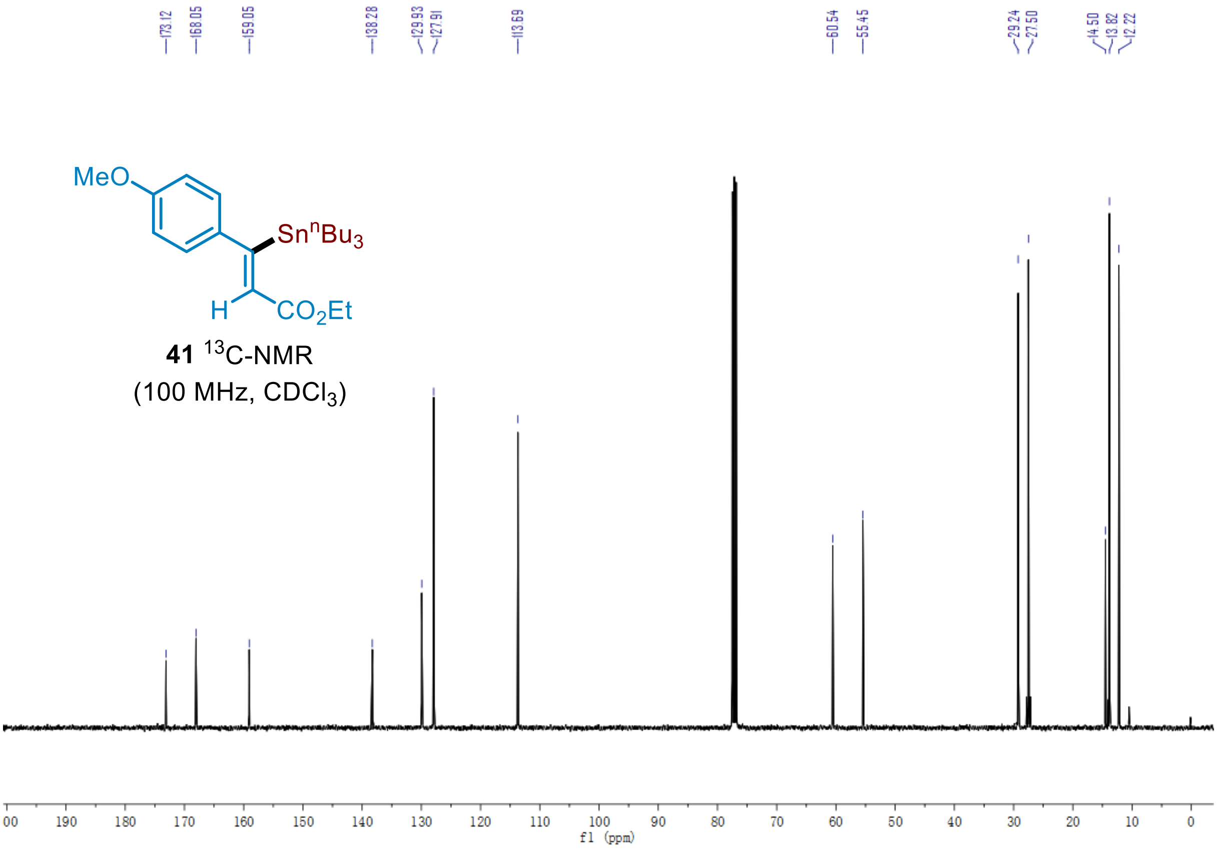

Supplementary Figure 173. **^{13}C-NMR** (100 MHz, $CDCl_3$, 298K) of **41**

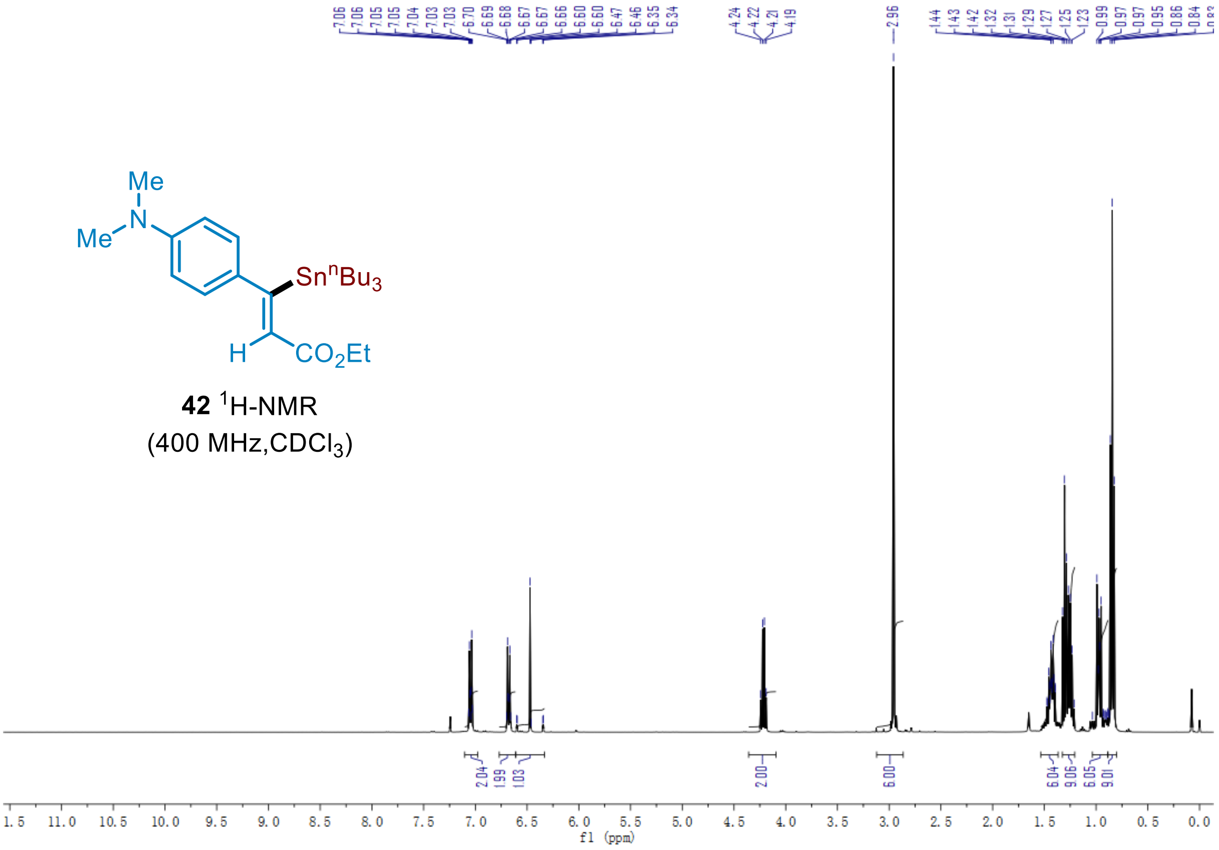

Supplementary Figure 174. **^{1}H-NMR** (400 MHz, $CDCl_3$, 298K) of **42**

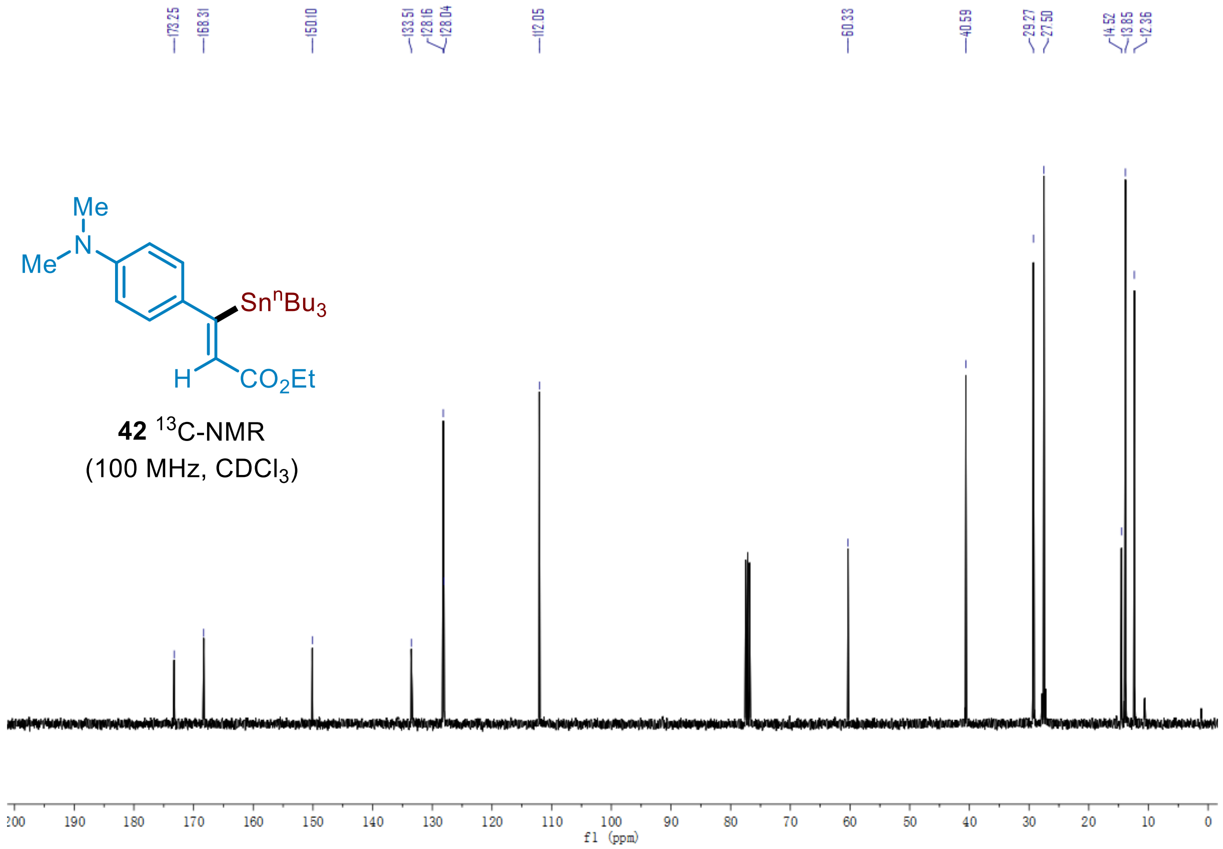

Supplementary Figure 175. **^{13}C-NMR** (100 MHz, $CDCl_3$, 298K) of **42**

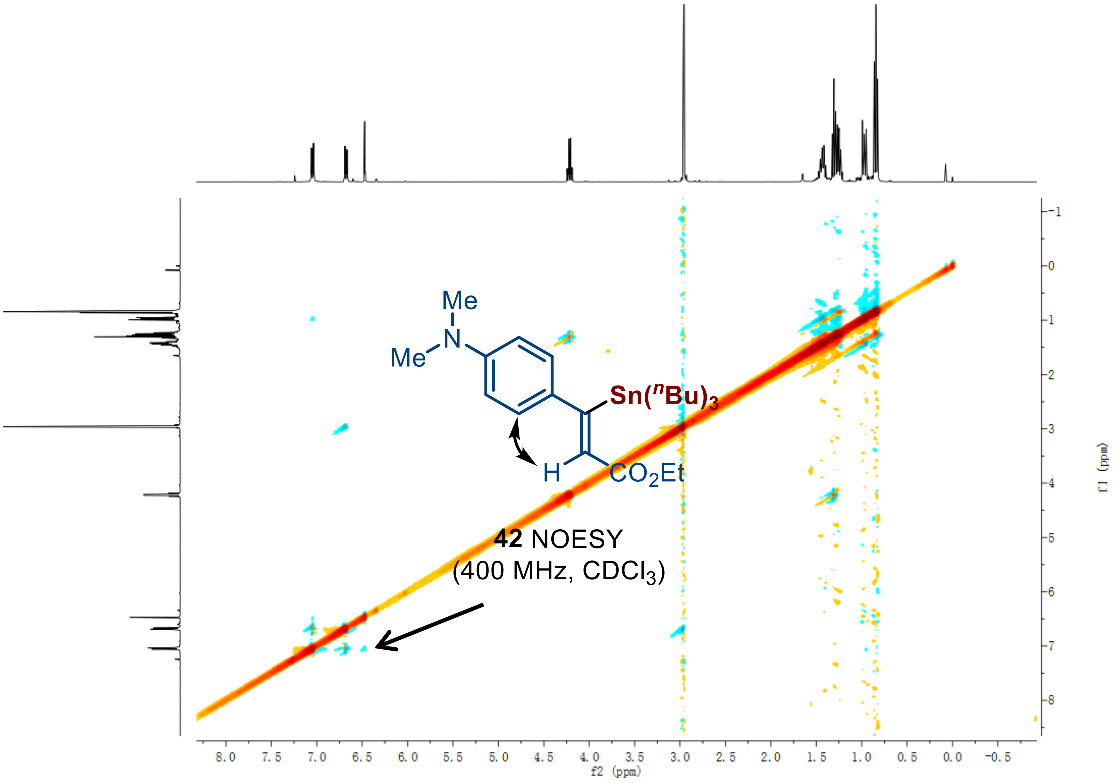

Supplementary Figure 176. NOESY of **42**

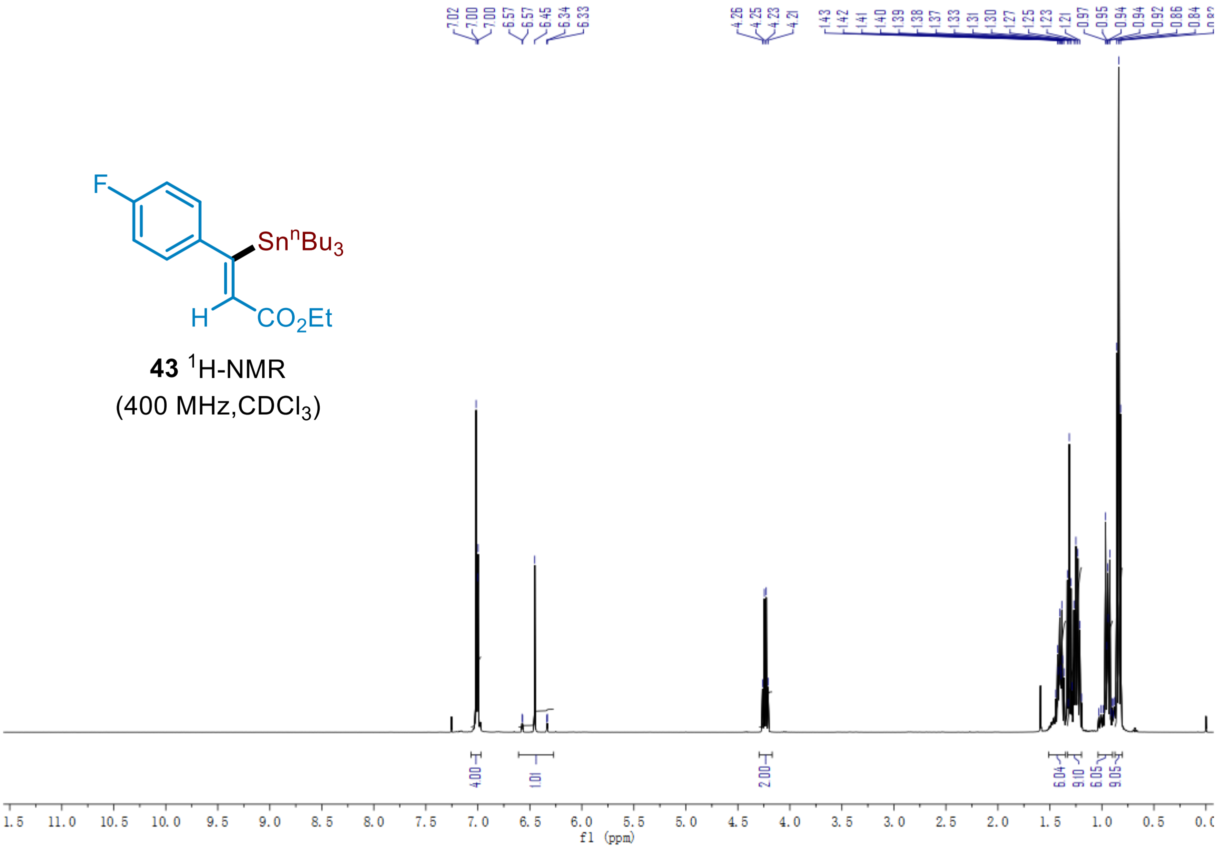

Supplementary Figure 177. **^{1}H-NMR** (400 MHz, $CDCl_3$, 298K) of **43**

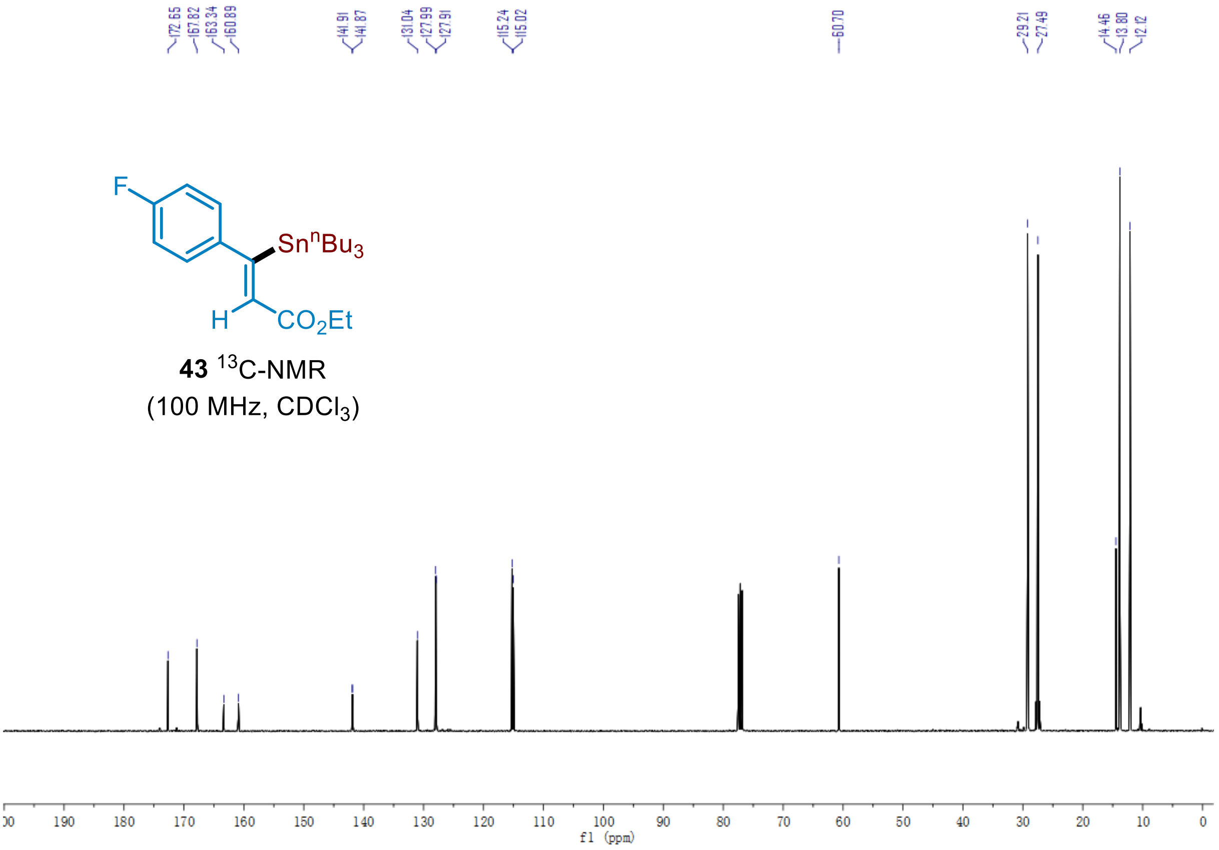

Supplementary Figure 178. **^{13}C-NMR** (100 MHz, $CDCl_3$, 298K) of **43**

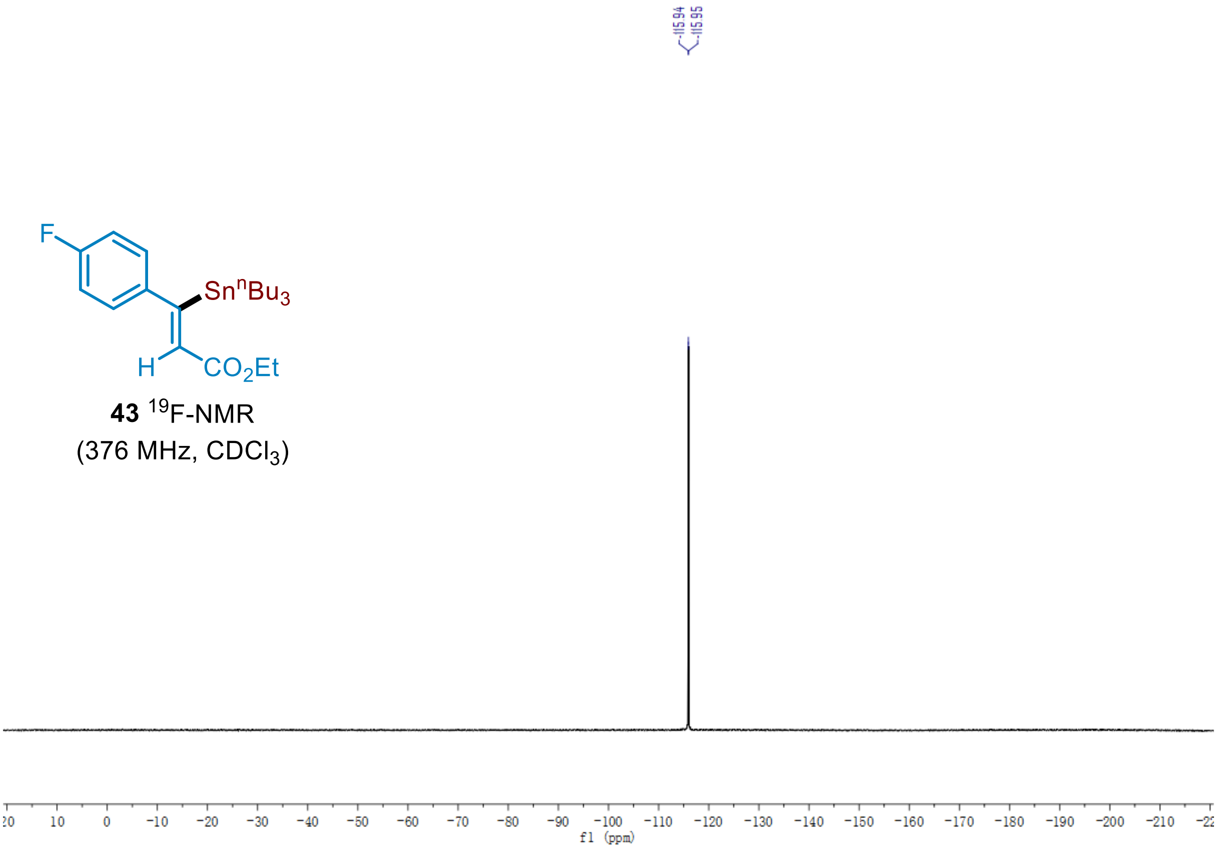

Supplementary Figure 179. **^{19}F-NMR** (376 MHz, $CDCl_3$, 298K) of **43**

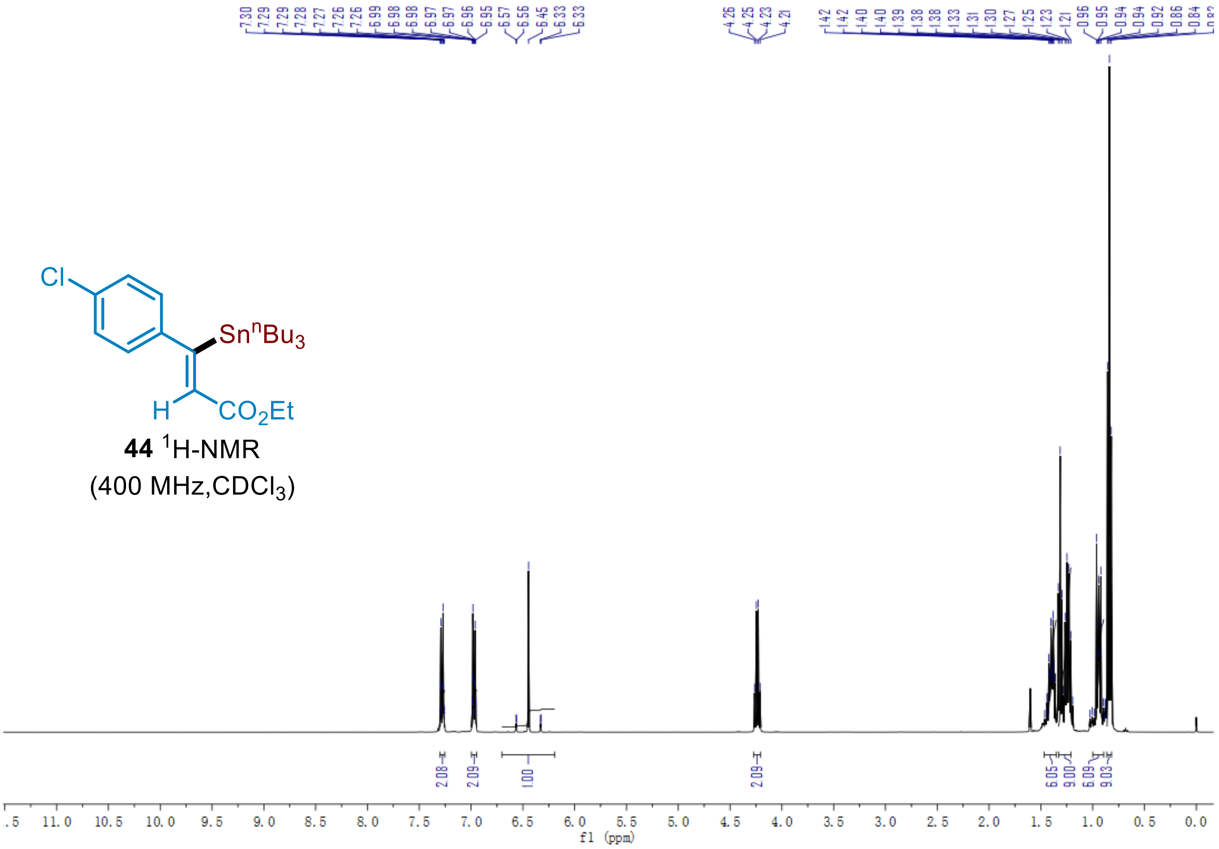

Supplementary Figure 180. **^{1}H-NMR** (400 MHz, $CDCl_3$, 298K) of **44**

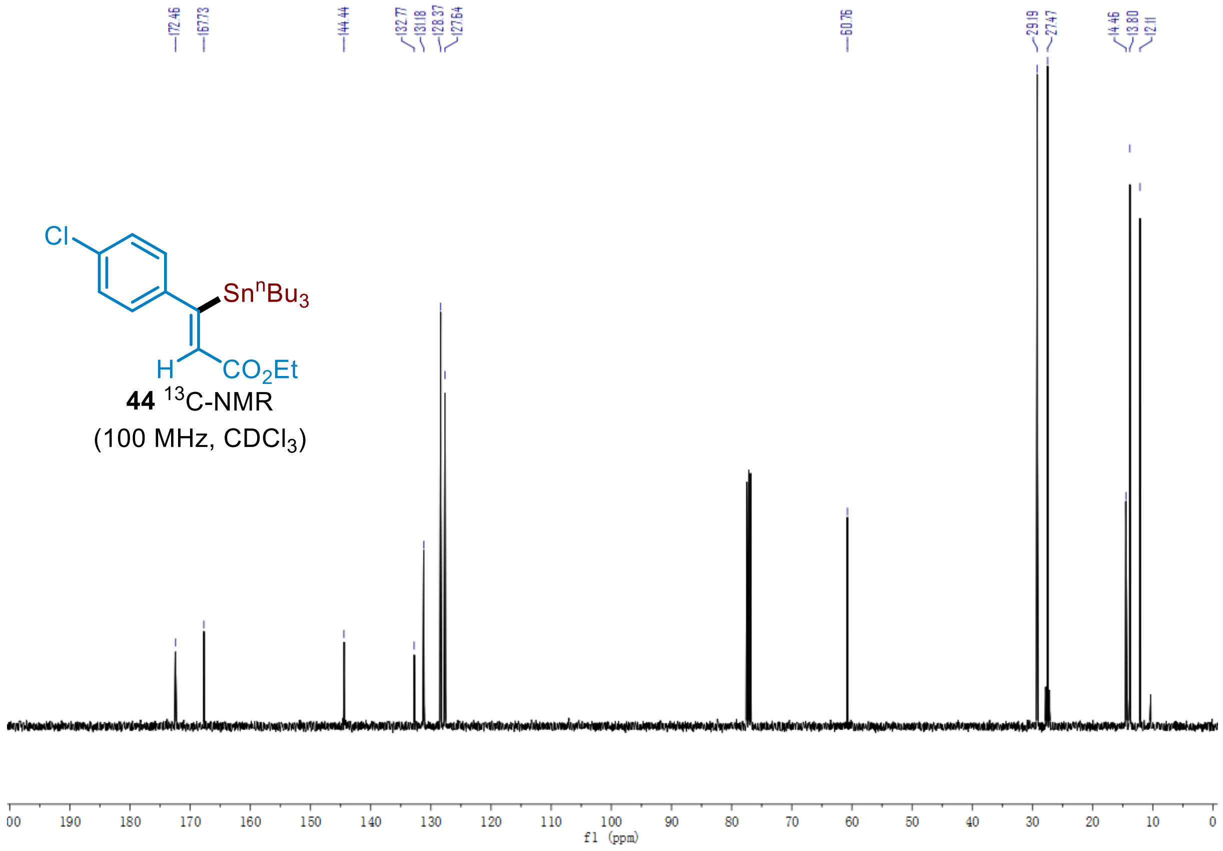

Supplementary Figure 181. **^{13}C-NMR** (100 MHz, $CDCl_3$, 298K) of **44**

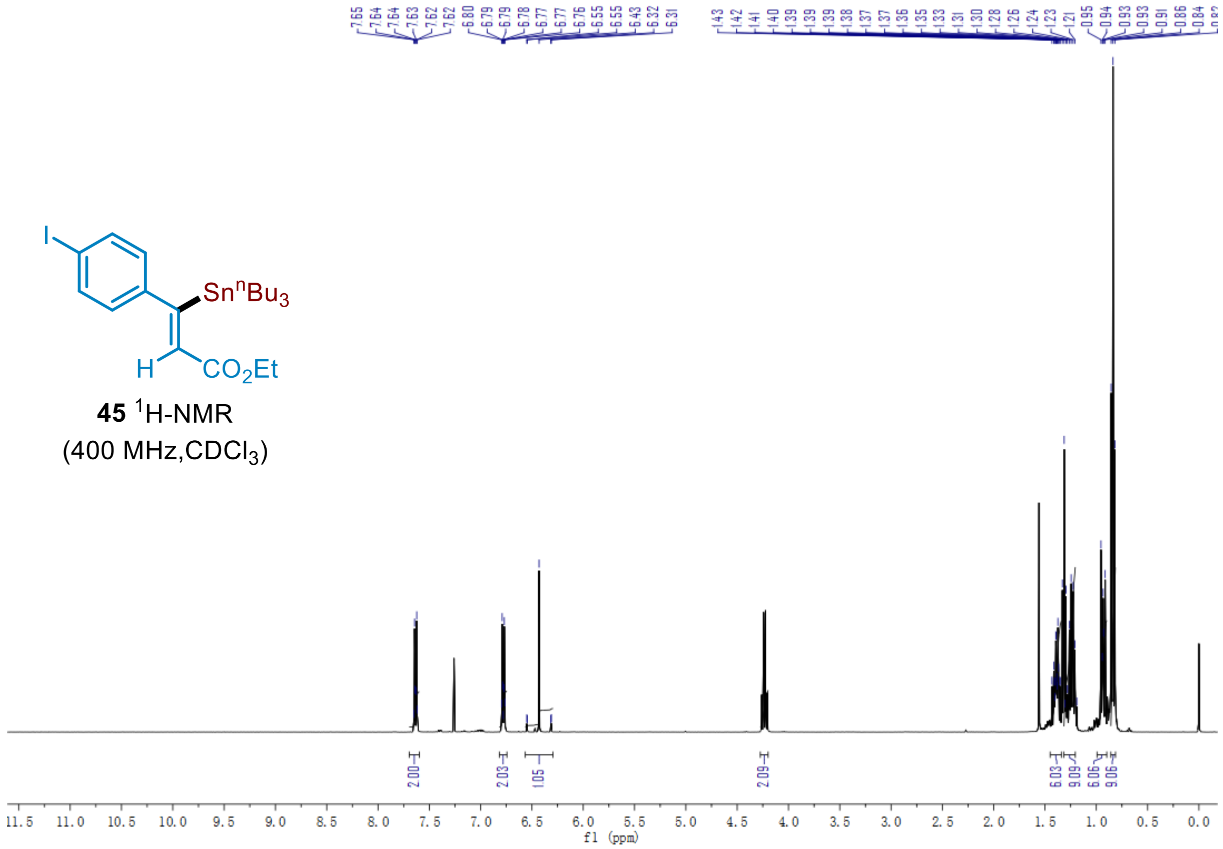

Supplementary Figure 182. **^{1}H-NMR** (400 MHz, $CDCl_3$, 298K) of **45**

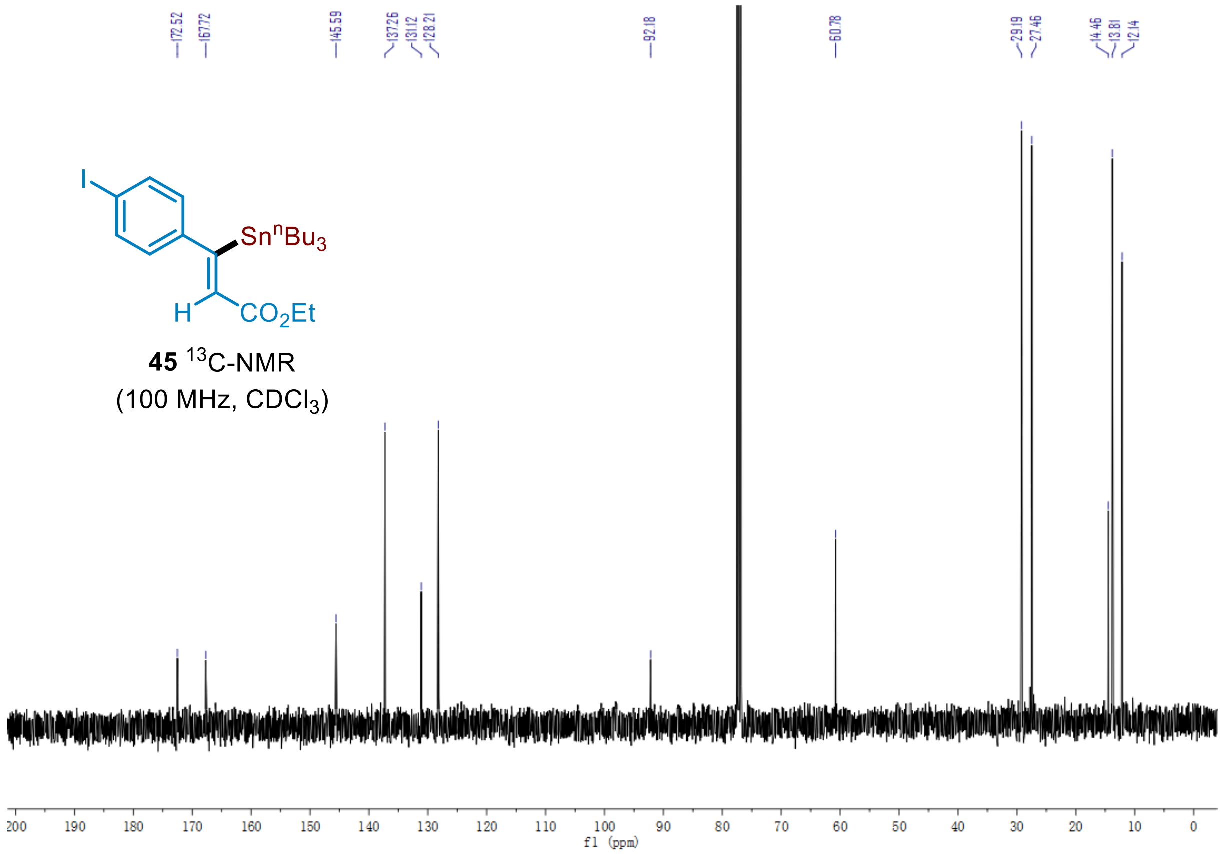

Supplementary Figure 183. **^{13}C-NMR** (100 MHz, $CDCl_3$, 298K) of **45**

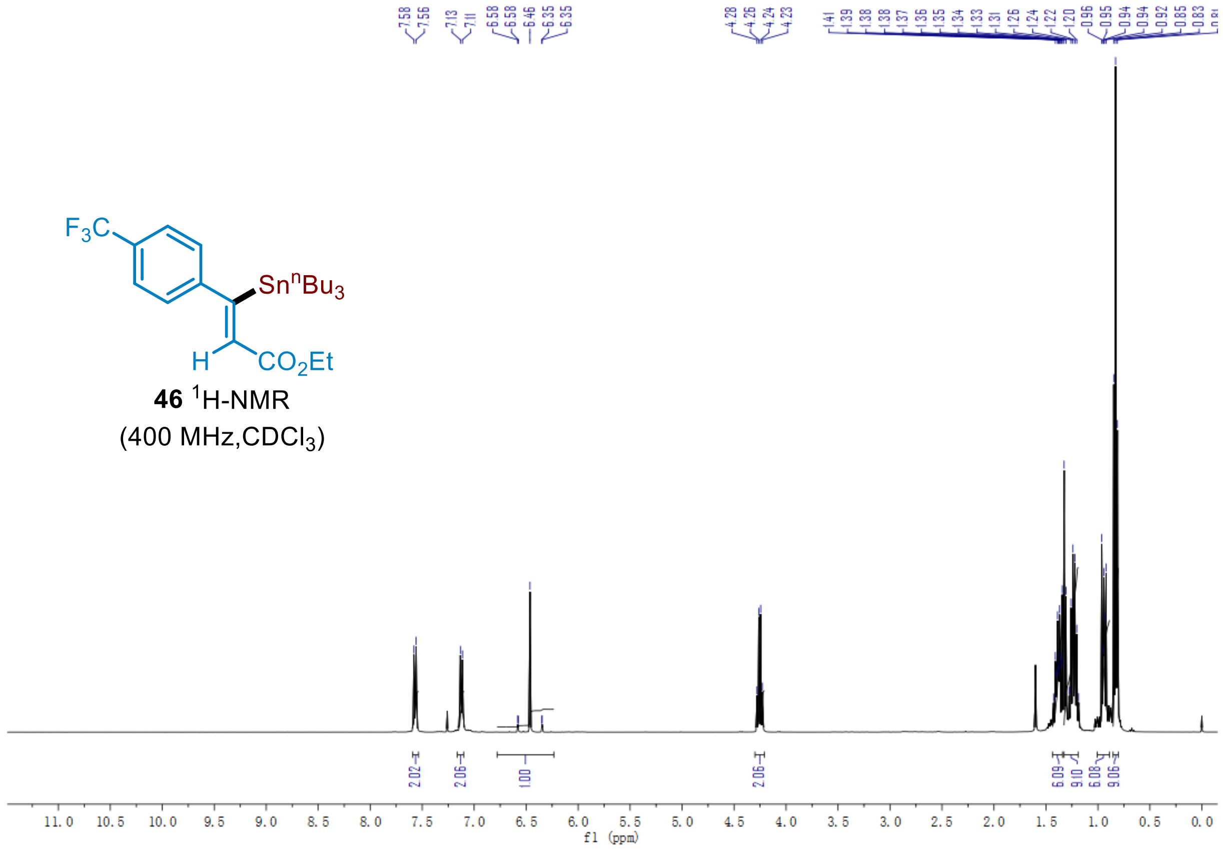

Supplementary Figure 184. **^{1}H-NMR** (400 MHz, $CDCl_3$, 298K) of **46**

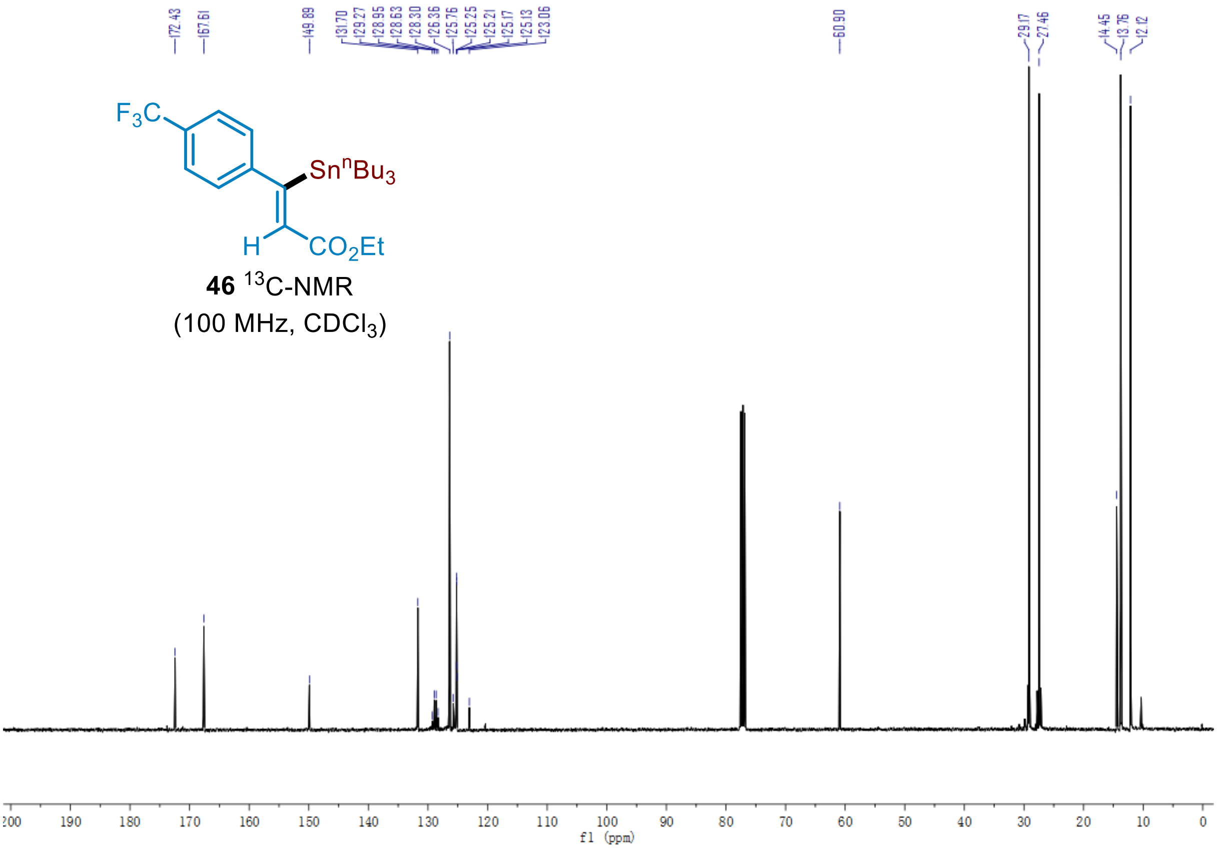

Supplementary Figure 185. **^{13}C-NMR** (100 MHz, $CDCl_3$, 298K) of **46**

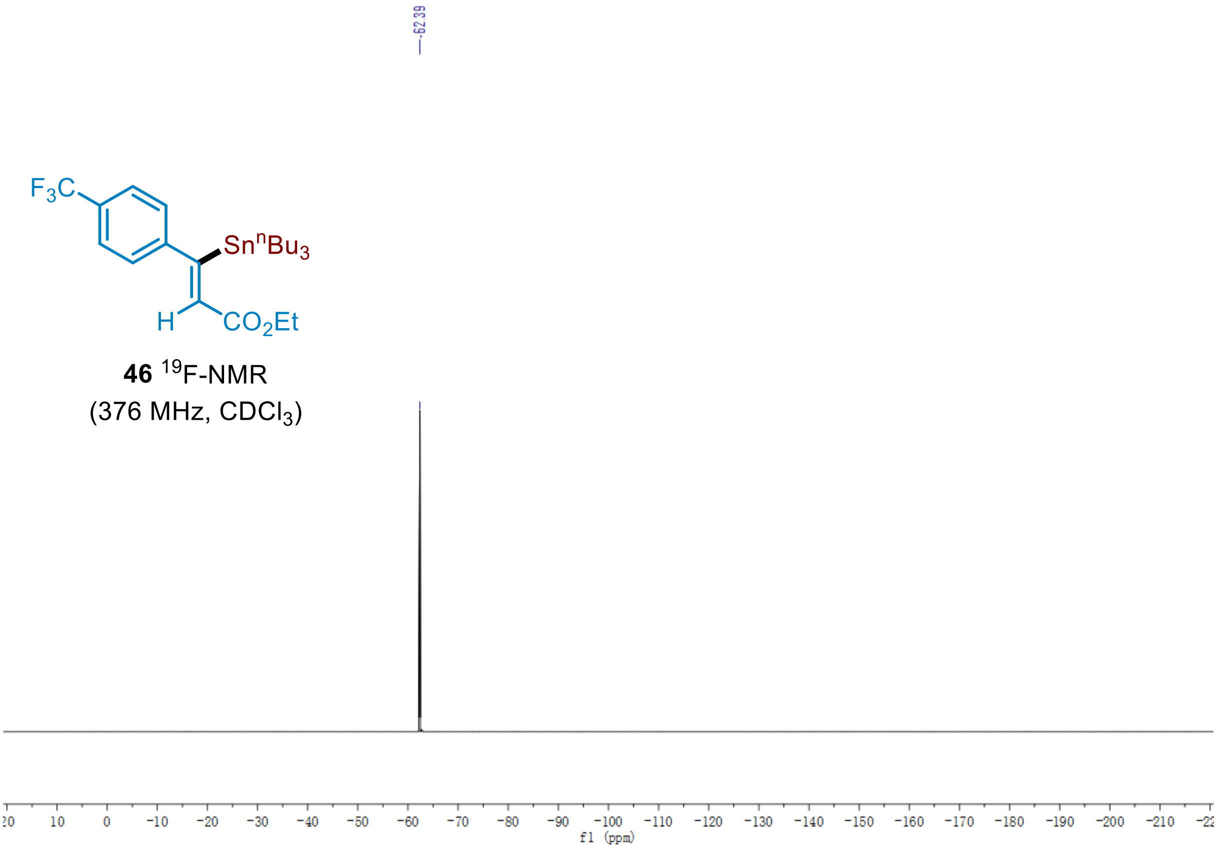

Supplementary Figure 186. **^{19}F-NMR** (376 MHz, $CDCl_3$, 298K) of **46**

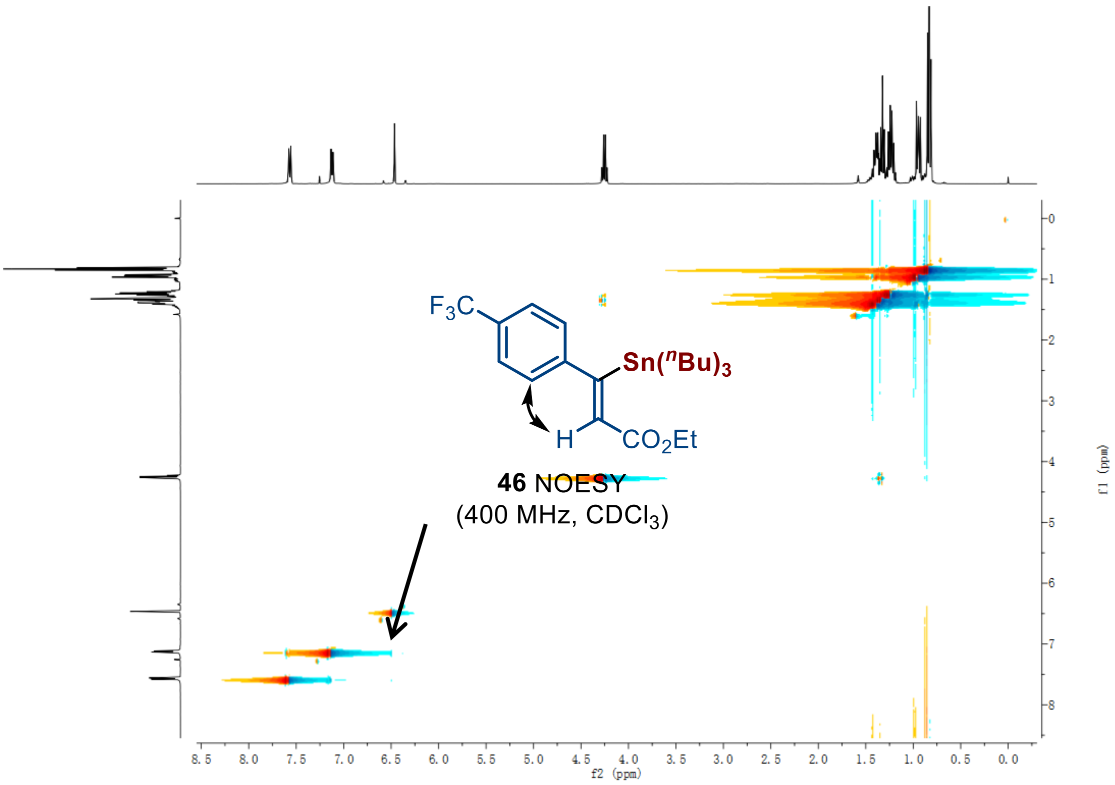

Supplementary Figure 187. **NOESY** of **46**

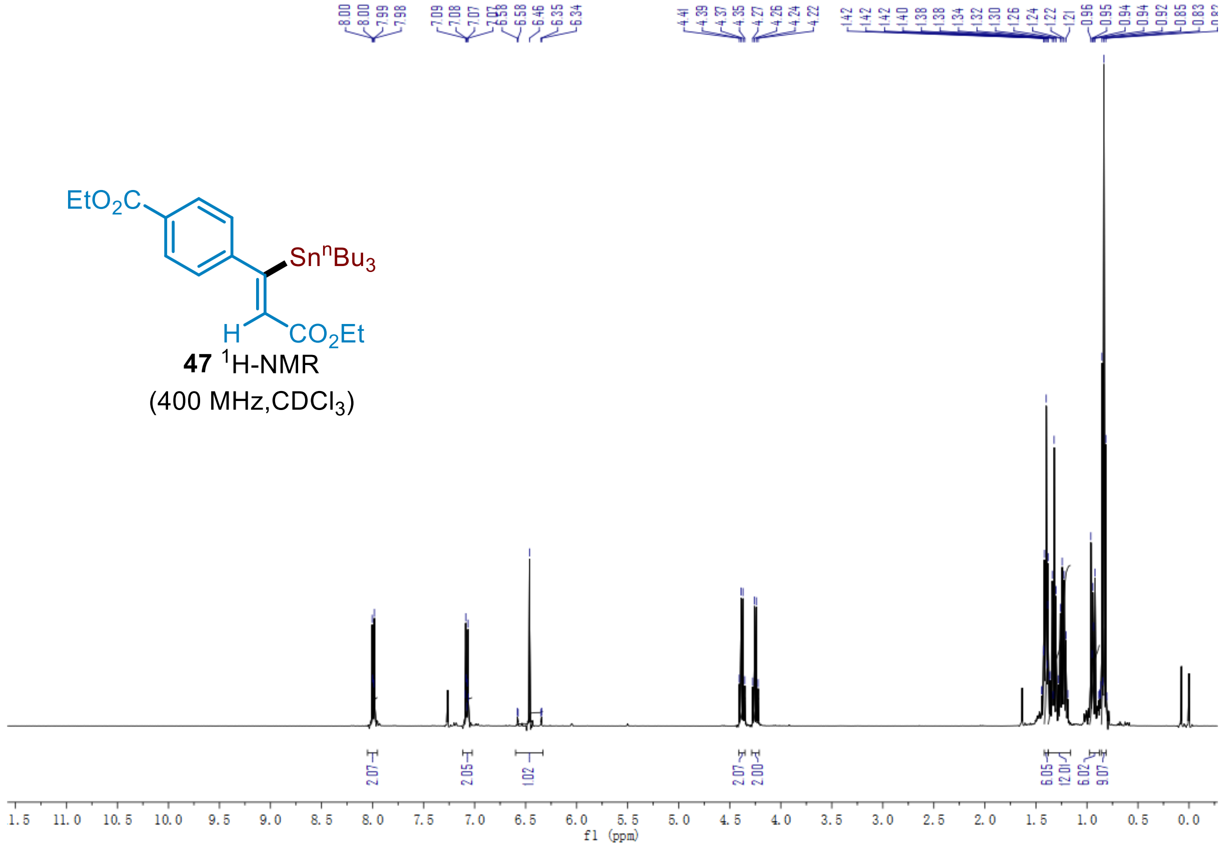

Supplementary Figure 188. **^{1}H-NMR** (400 MHz, $CDCl_3$, 298K) of **47**

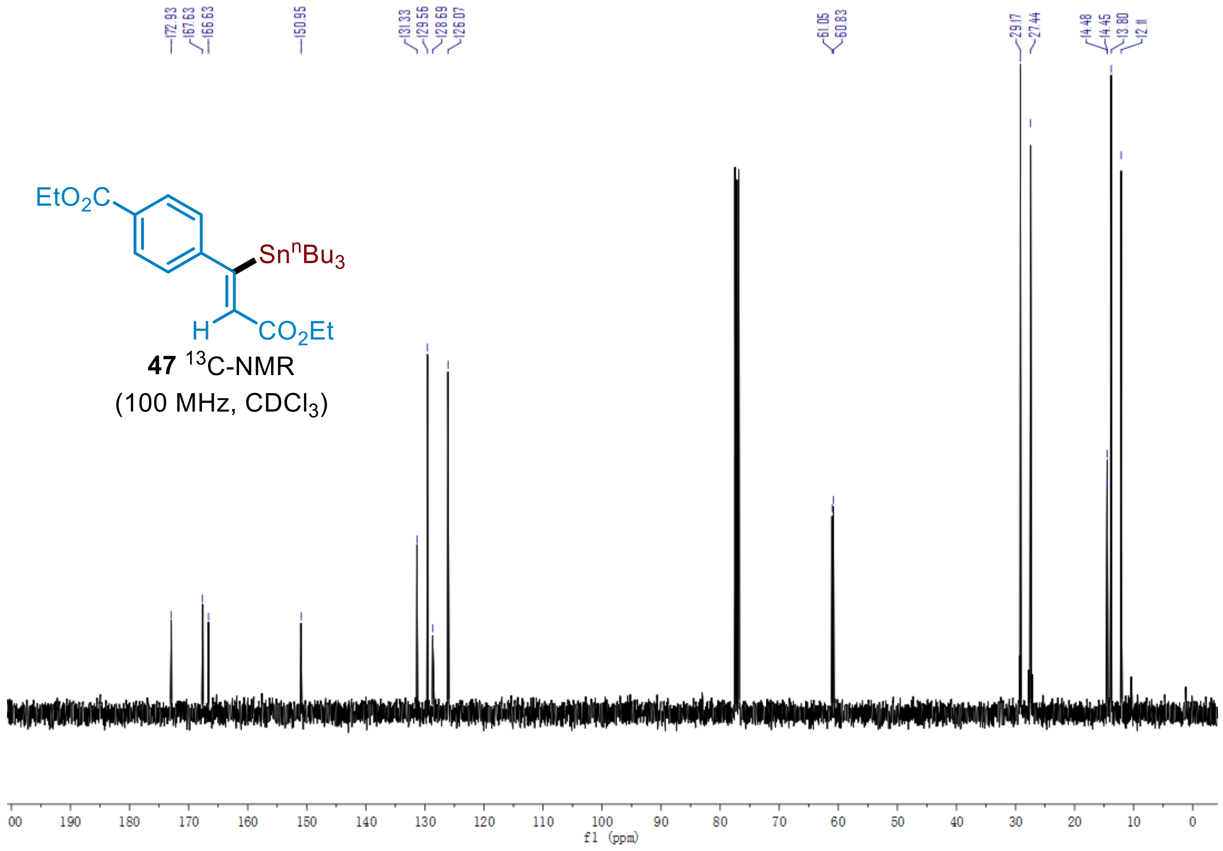

Supplementary Figure 189. **^{13}C-NMR** (100 MHz, $CDCl_3$, 298K) of **47**

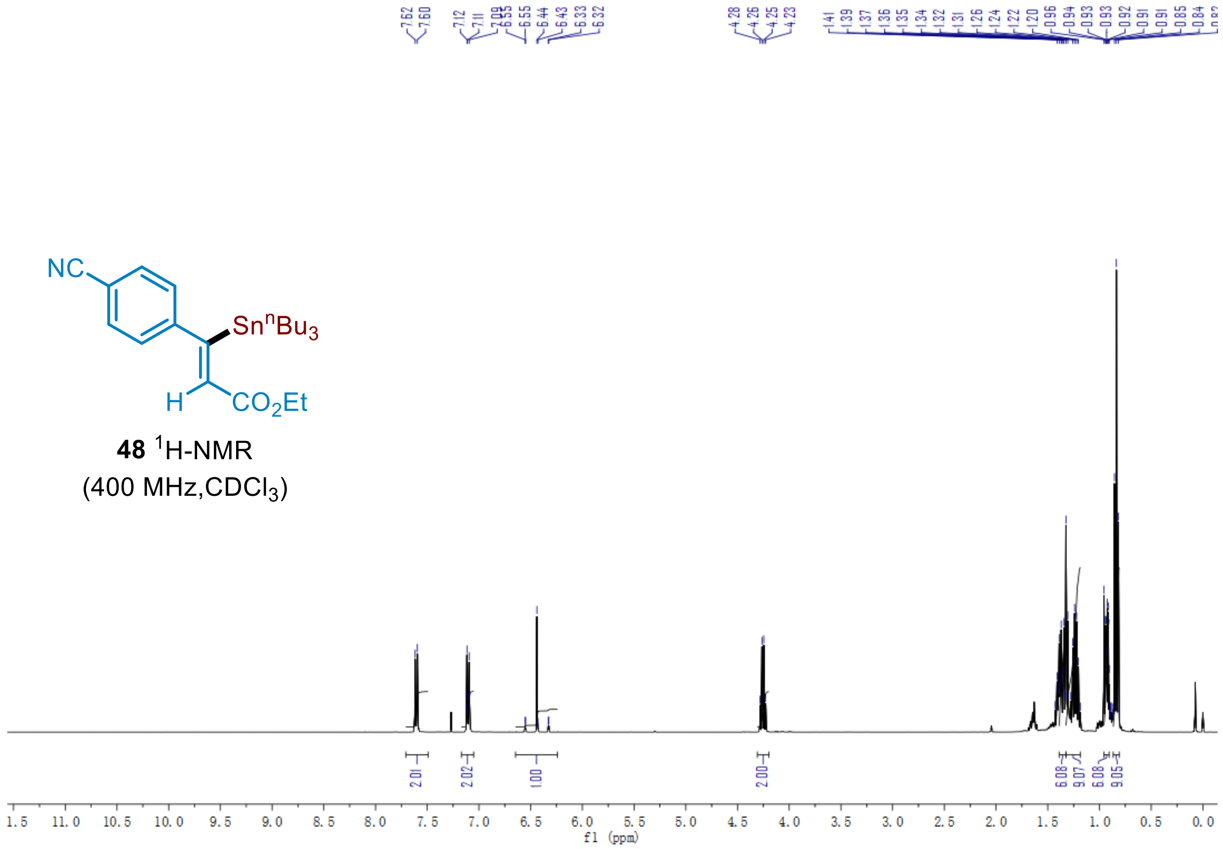

Supplementary Figure 190. **^{1}H-NMR** (400 MHz, $CDCl_3$, 298K) of **48**

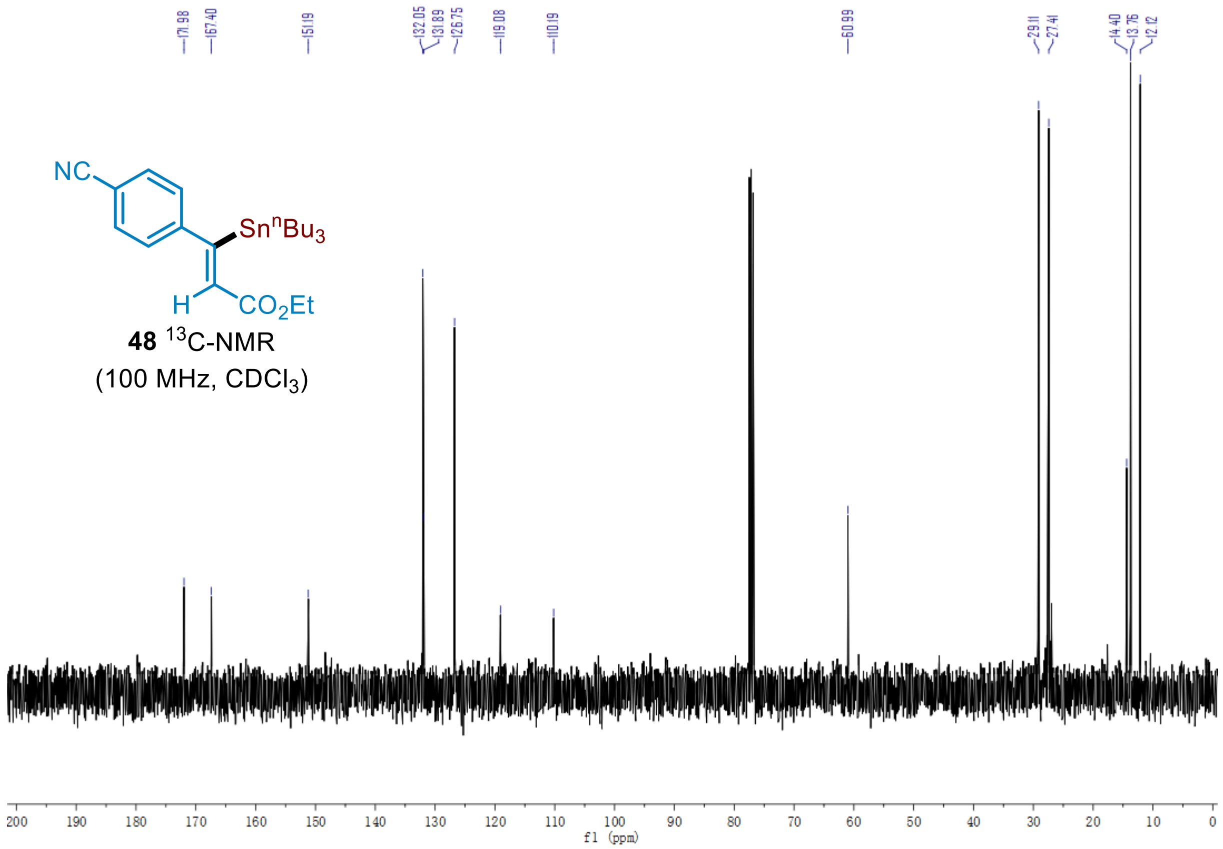

Supplementary Figure 191. **^{13}C-NMR** (100 MHz, $CDCl_3$, 298K) of **48**

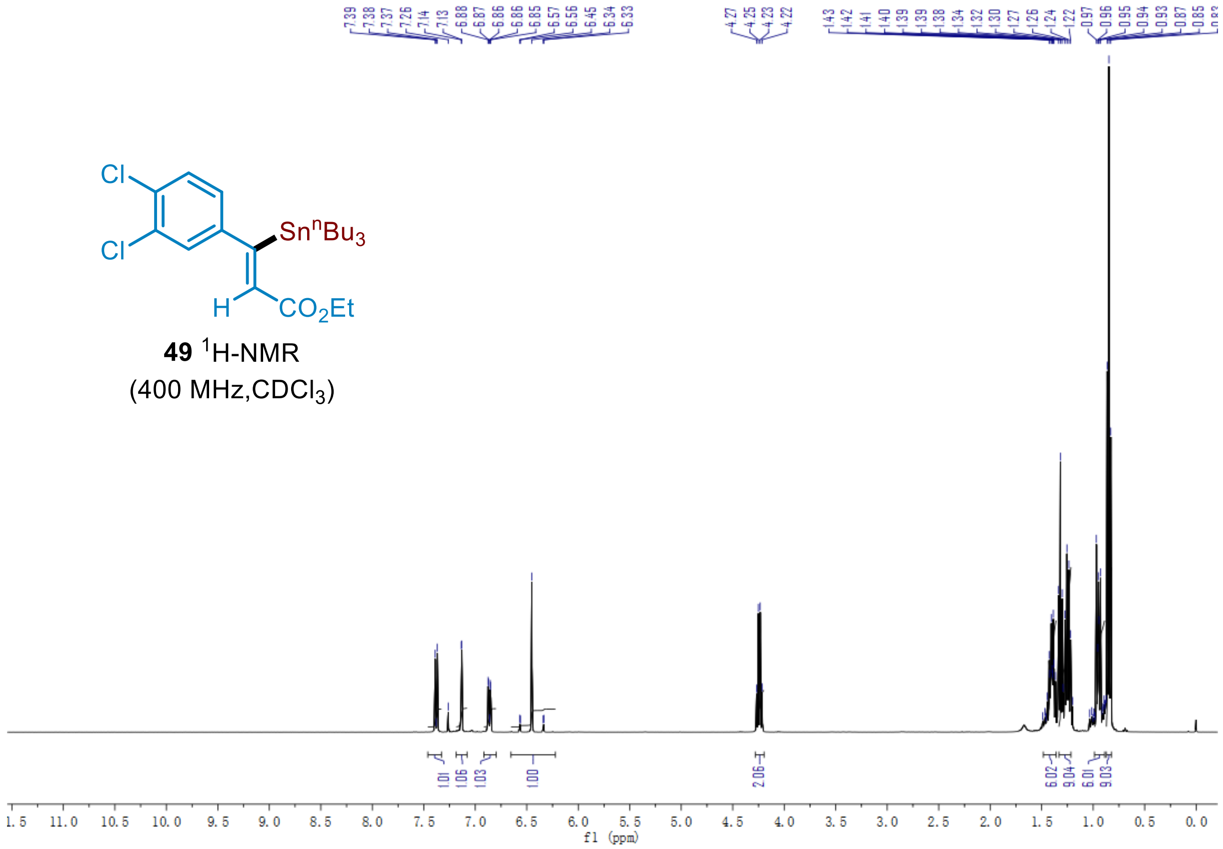

Supplementary Figure 192. **^{1}H-NMR** (400 MHz, $CDCl_3$, 298K) of **49**

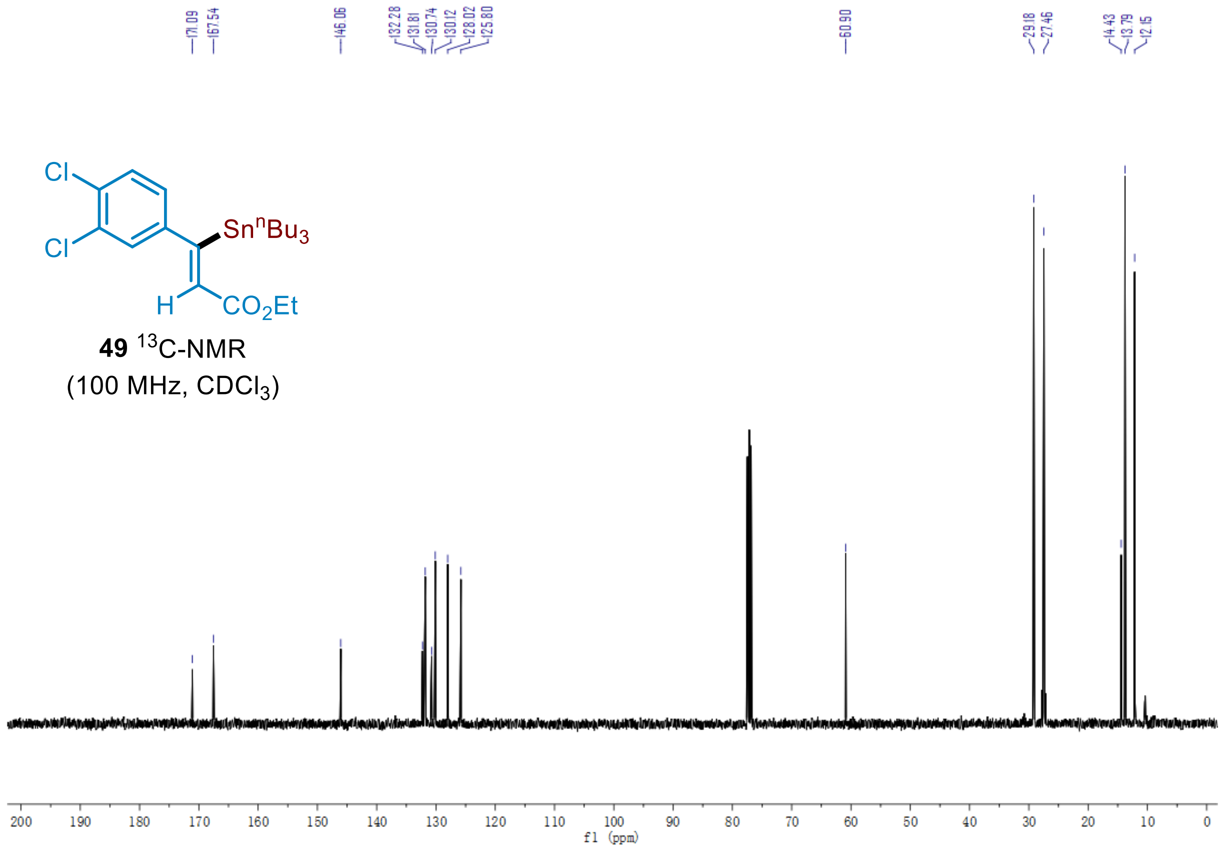

Supplementary Figure 193. **^{13}C-NMR** (100 MHz, $CDCl_3$, 298K) of **49**

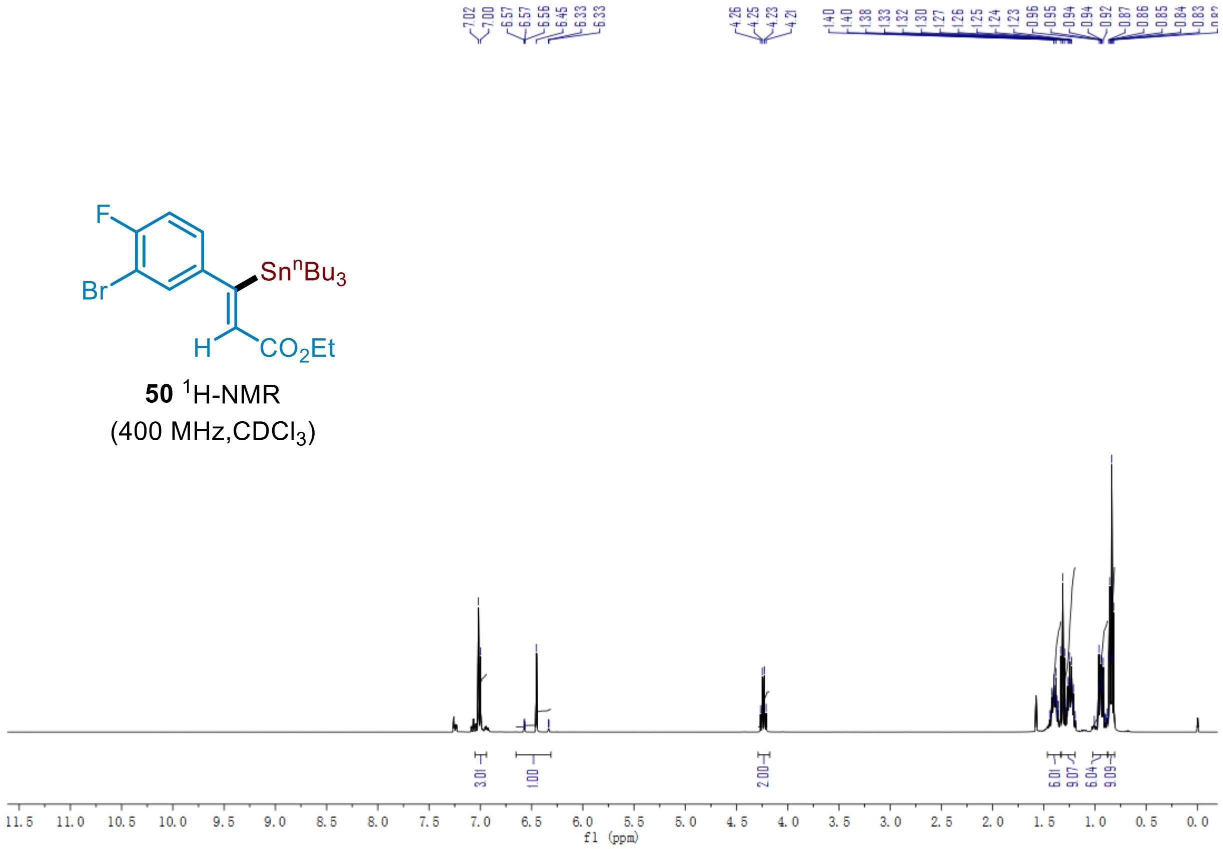

Supplementary Figure 194. **^{1}H-NMR** (400 MHz, $CDCl_3$, 298K) of **50**

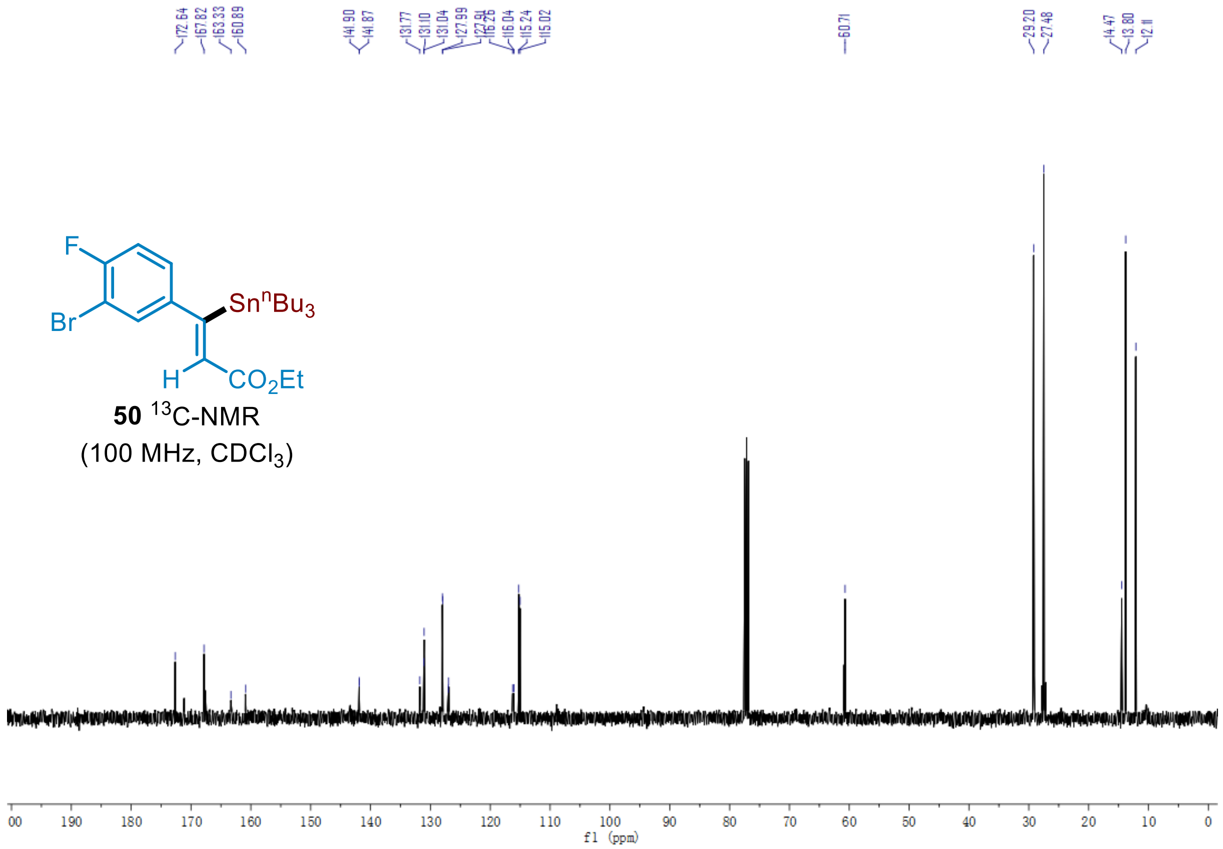

Supplementary Figure 195. **^{13}C-NMR** (100 MHz, $CDCl_3$, 298K) of **50**

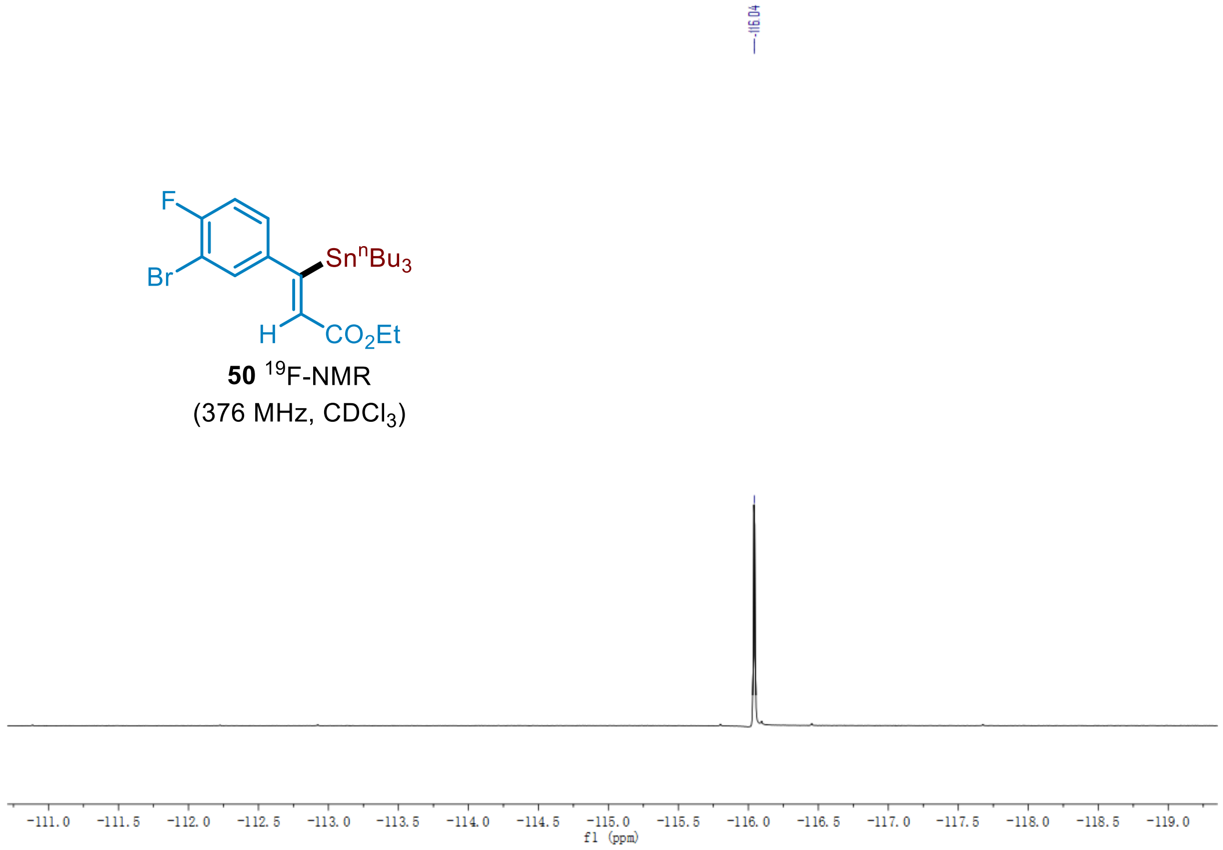

Supplementary Figure 196. **^{19}F-NMR** (376 MHz, $CDCl_3$, 298K) of **50**

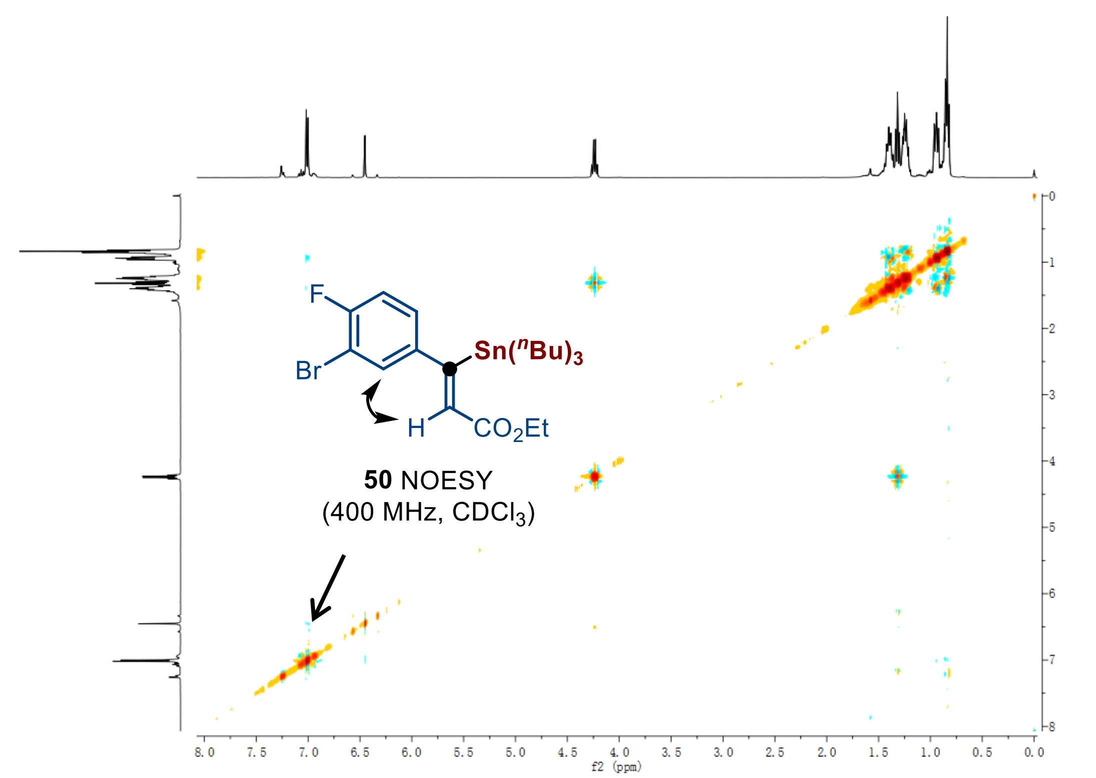

Supplementary Figure 197. **NOESY** of **50**

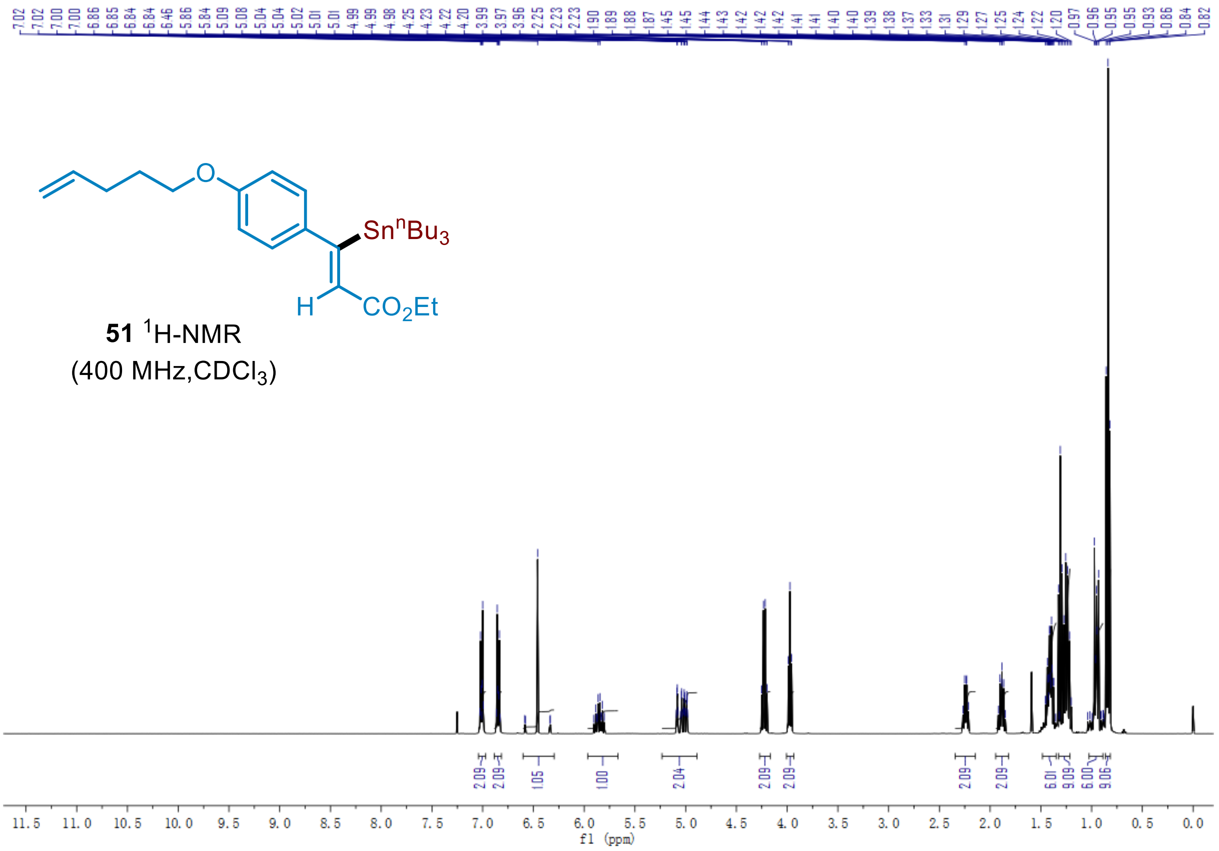

Supplementary Figure 198. **^{1}H-NMR** (400 MHz, $CDCl_3$, 298K) of **51**

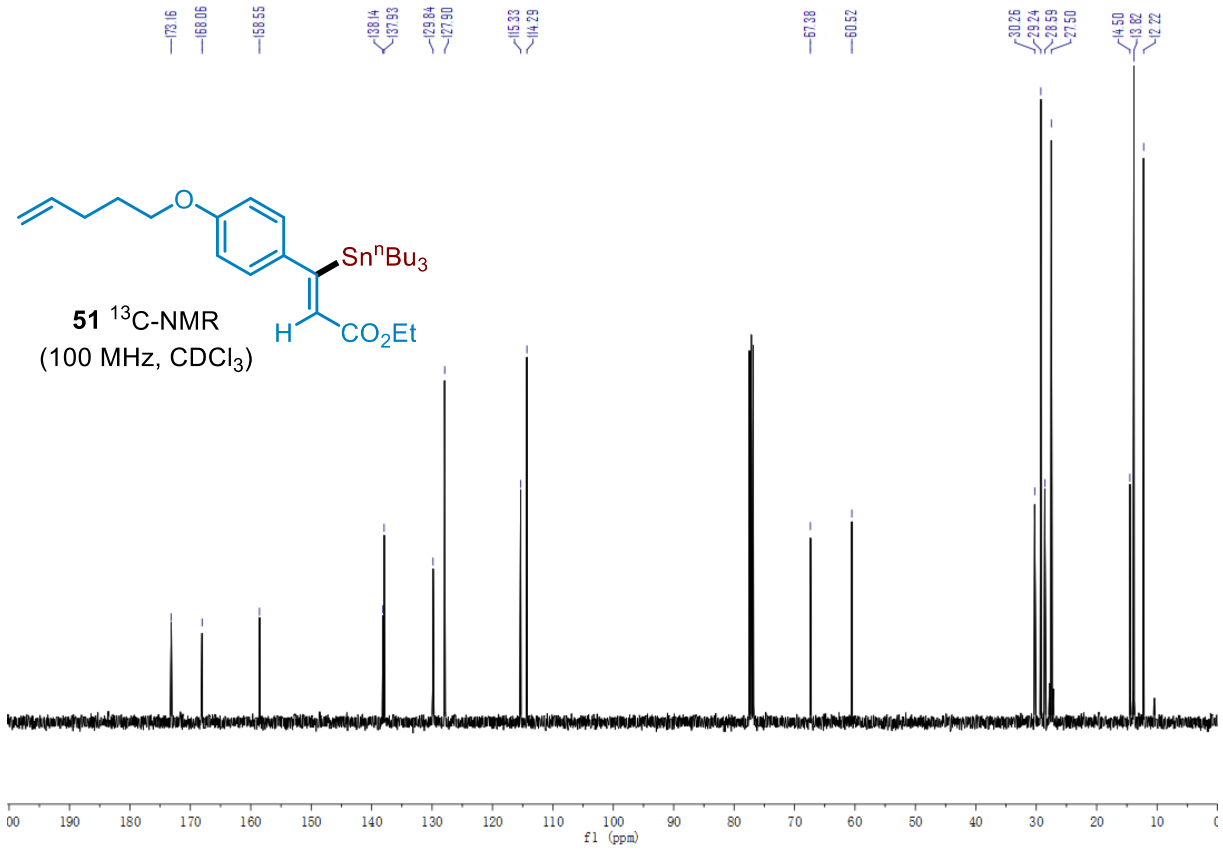

Supplementary Figure 199. **^{13}C-NMR** (100 MHz, $CDCl_3$, 298K) of **51**

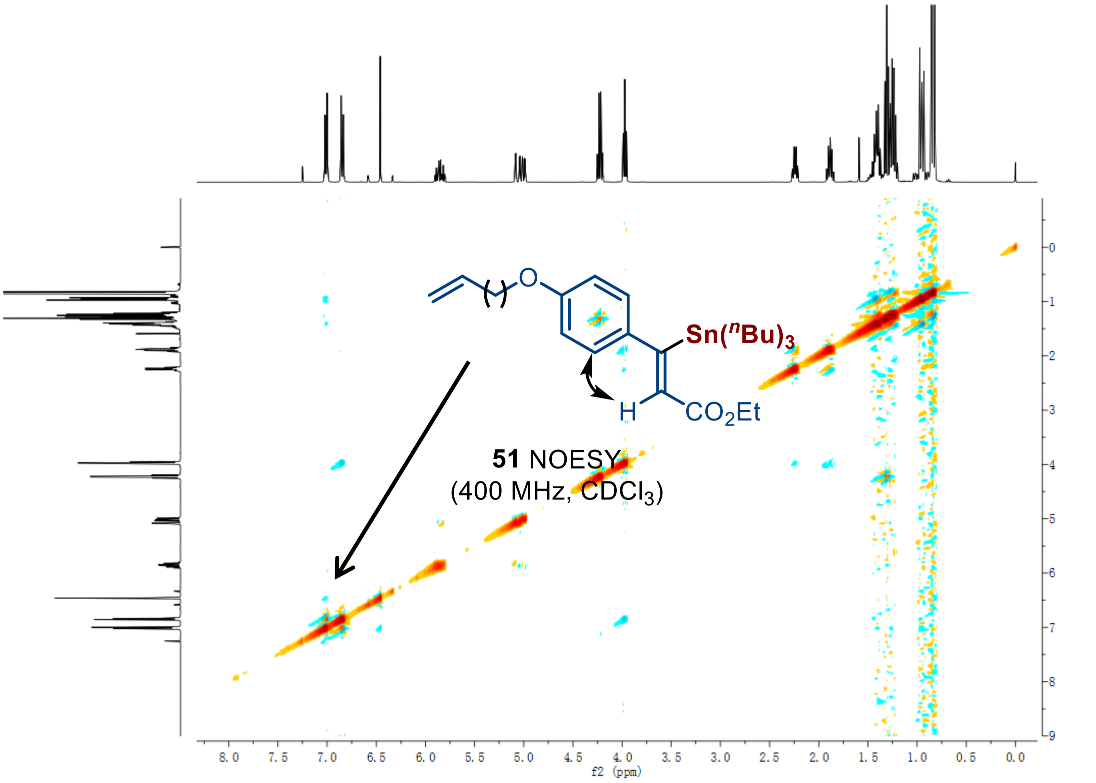

Supplementary Figure 200. **NOESY** of **51**

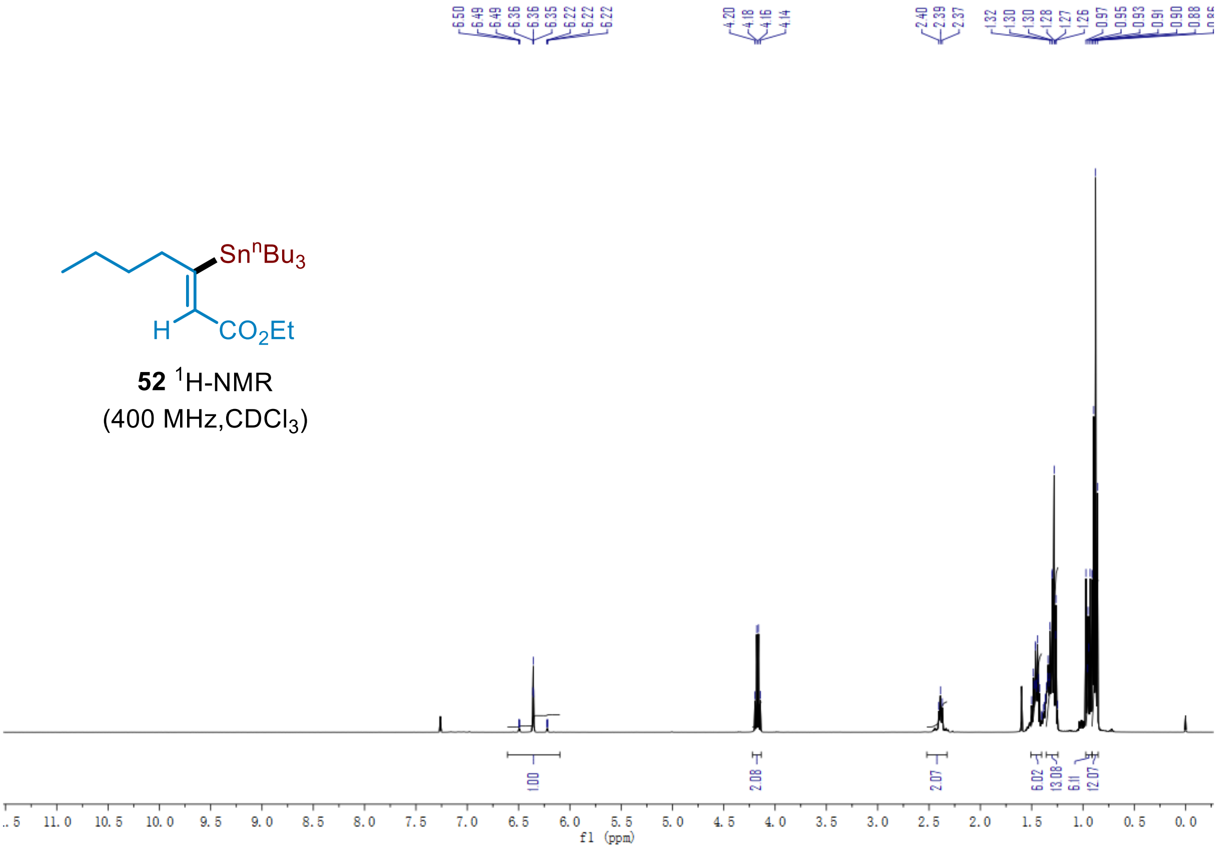

Supplementary Figure 201. **^{1}H-NMR** (400 MHz, $CDCl_3$, 298K) of **52**

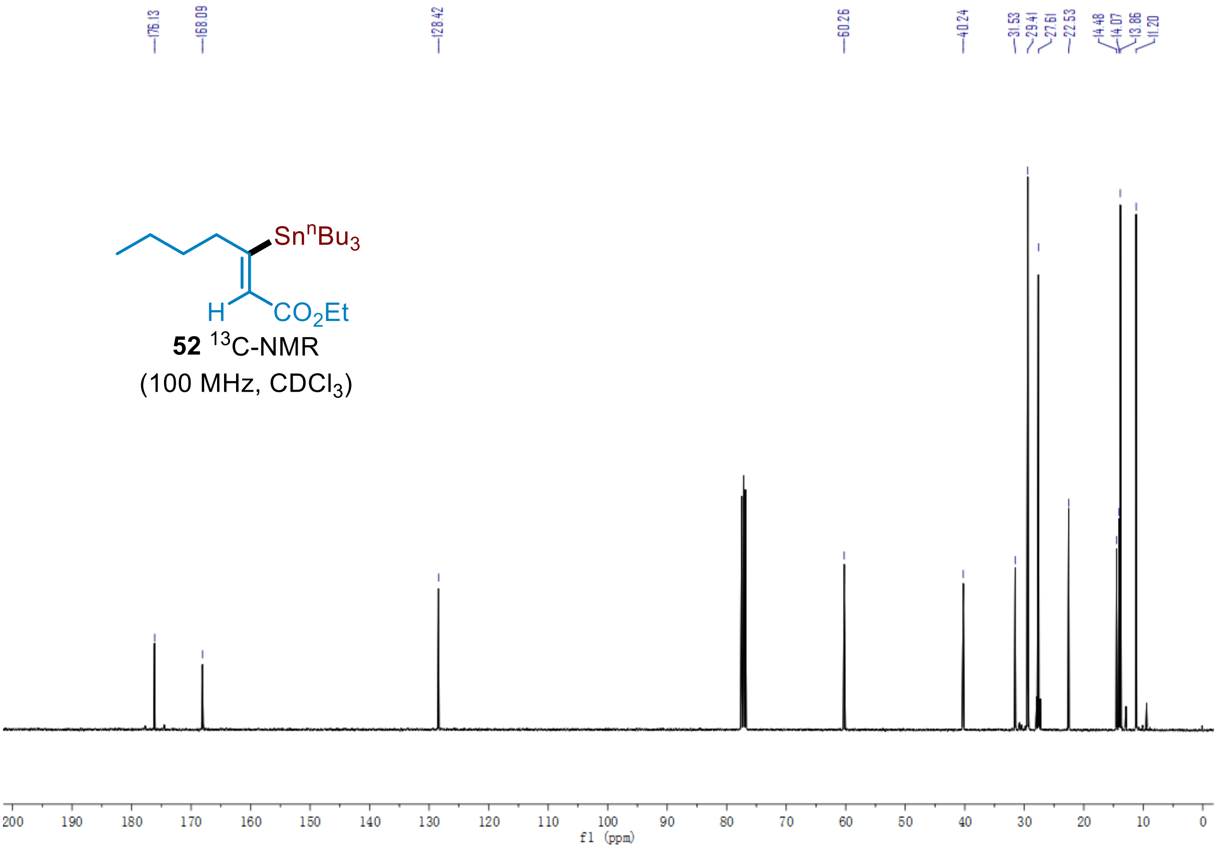

Supplementary Figure 202. **^{13}C-NMR** (100 MHz, $CDCl_3$, 298K) of **52**

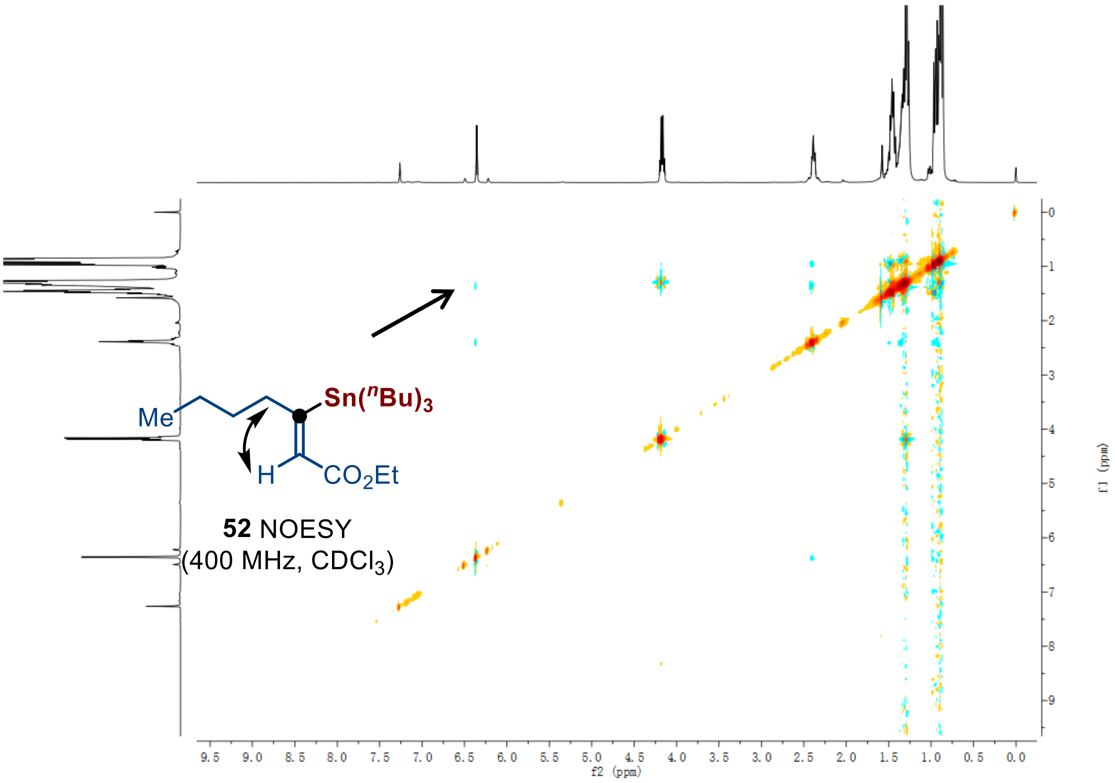

Supplementary Figure 203. **NOESY** of **52**

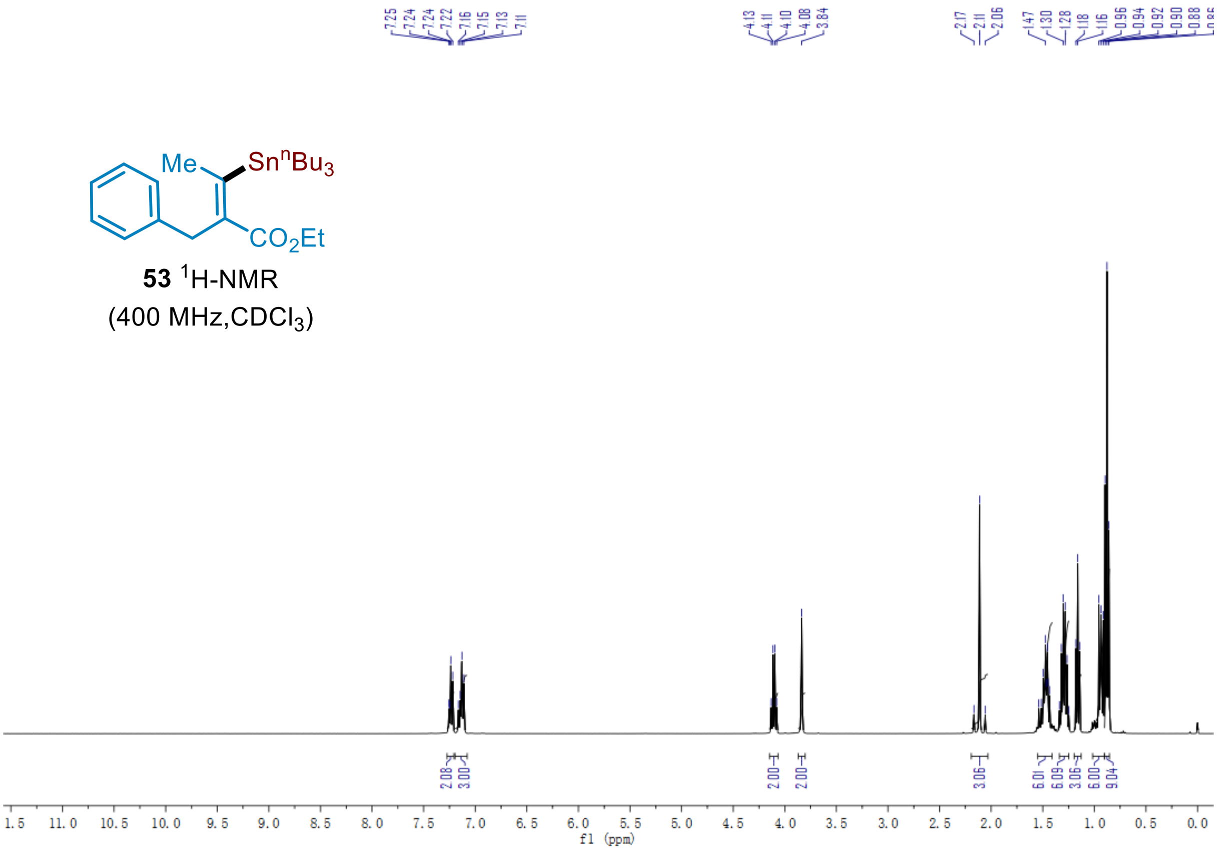

Supplementary Figure 204. **^{1}H-NMR** (400 MHz, $CDCl_3$, 298K) of **53**

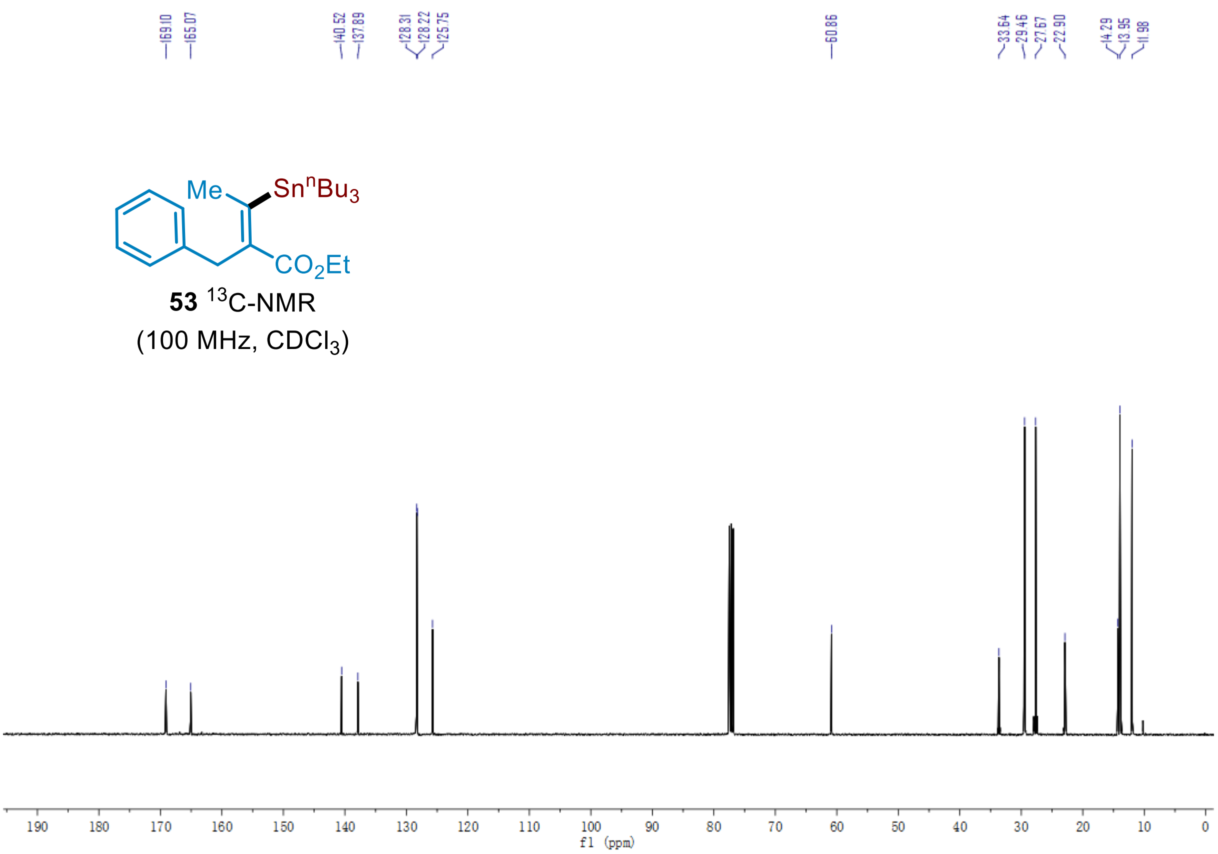

Supplementary Figure 205. **^{13}C-NMR** (100 MHz, $CDCl_3$, 298K) of **53**

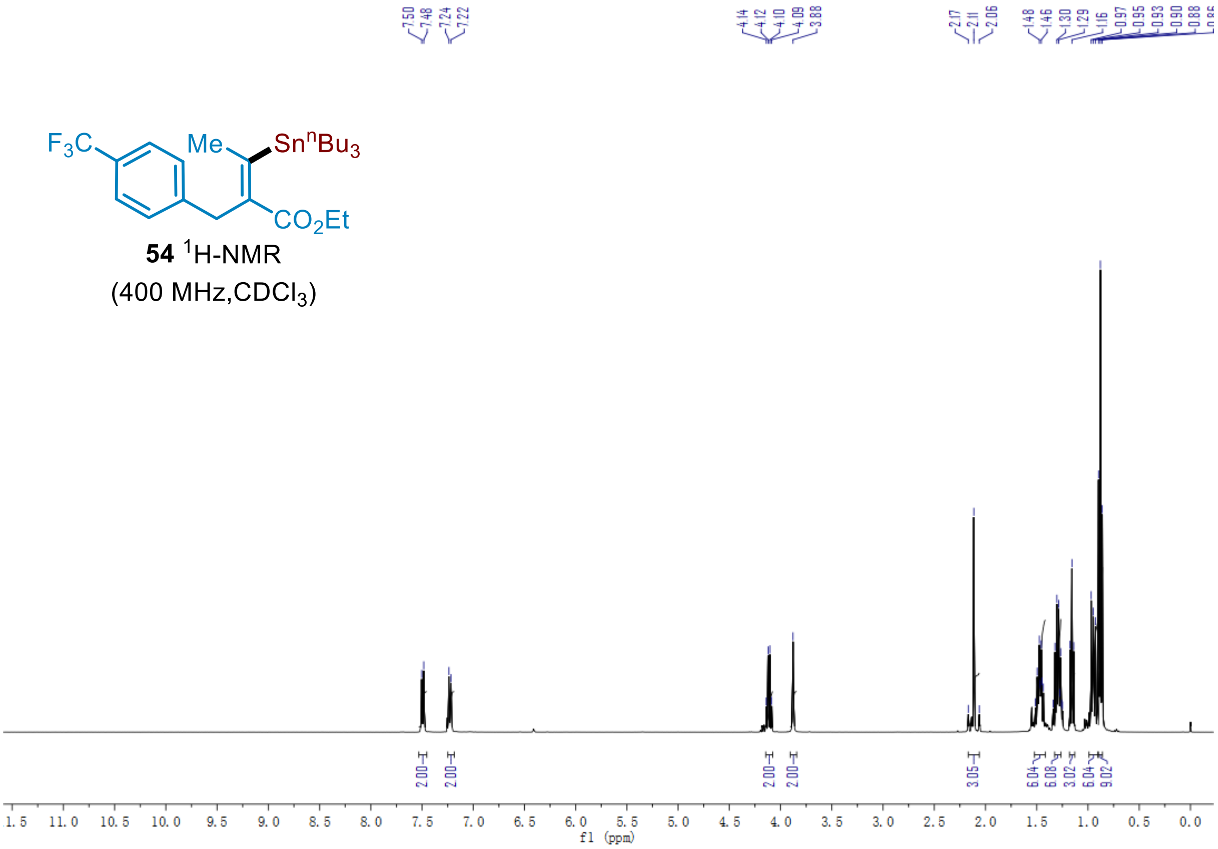

Supplementary Figure 206. **^{1}H-NMR** (400 MHz, $CDCl_3$, 298K) of **54**

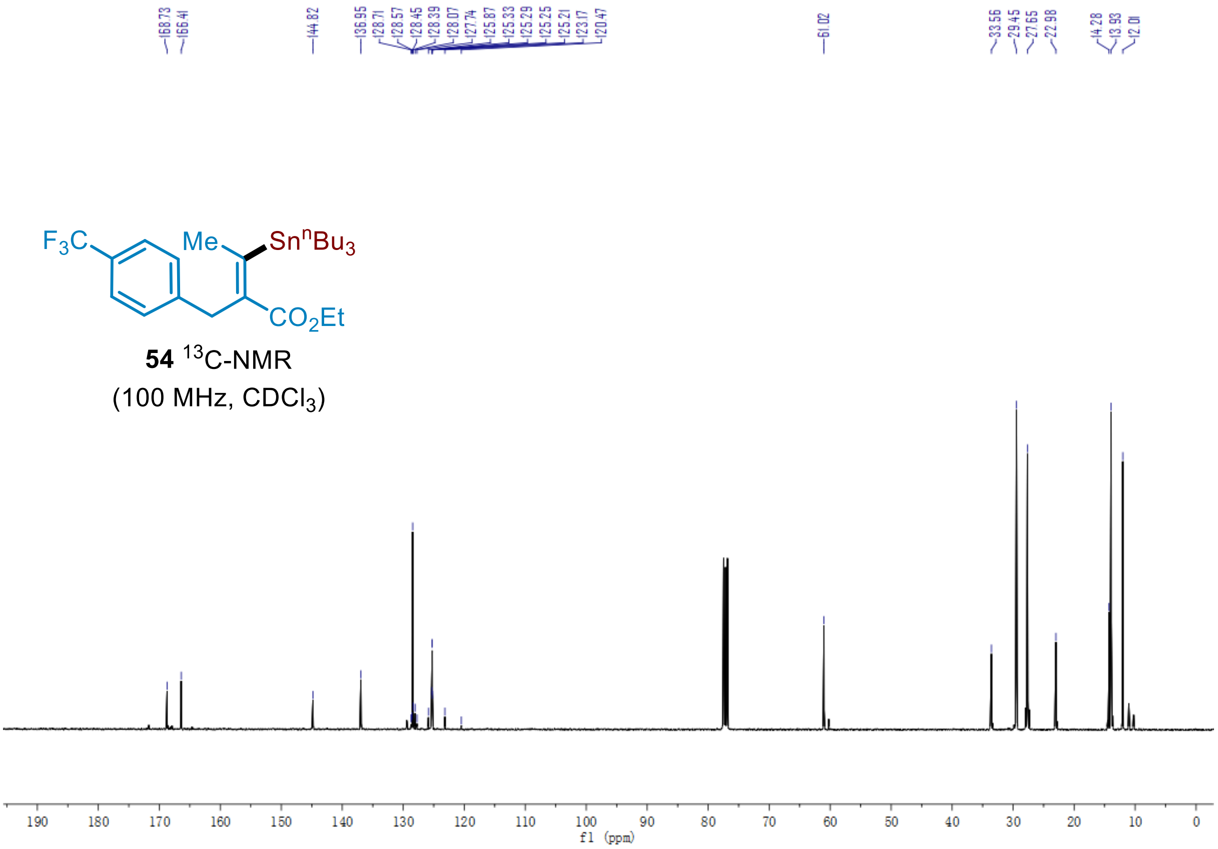

Supplementary Figure 207. **^{13}C-NMR** (100 MHz, $CDCl_3$, 298K) of **54**

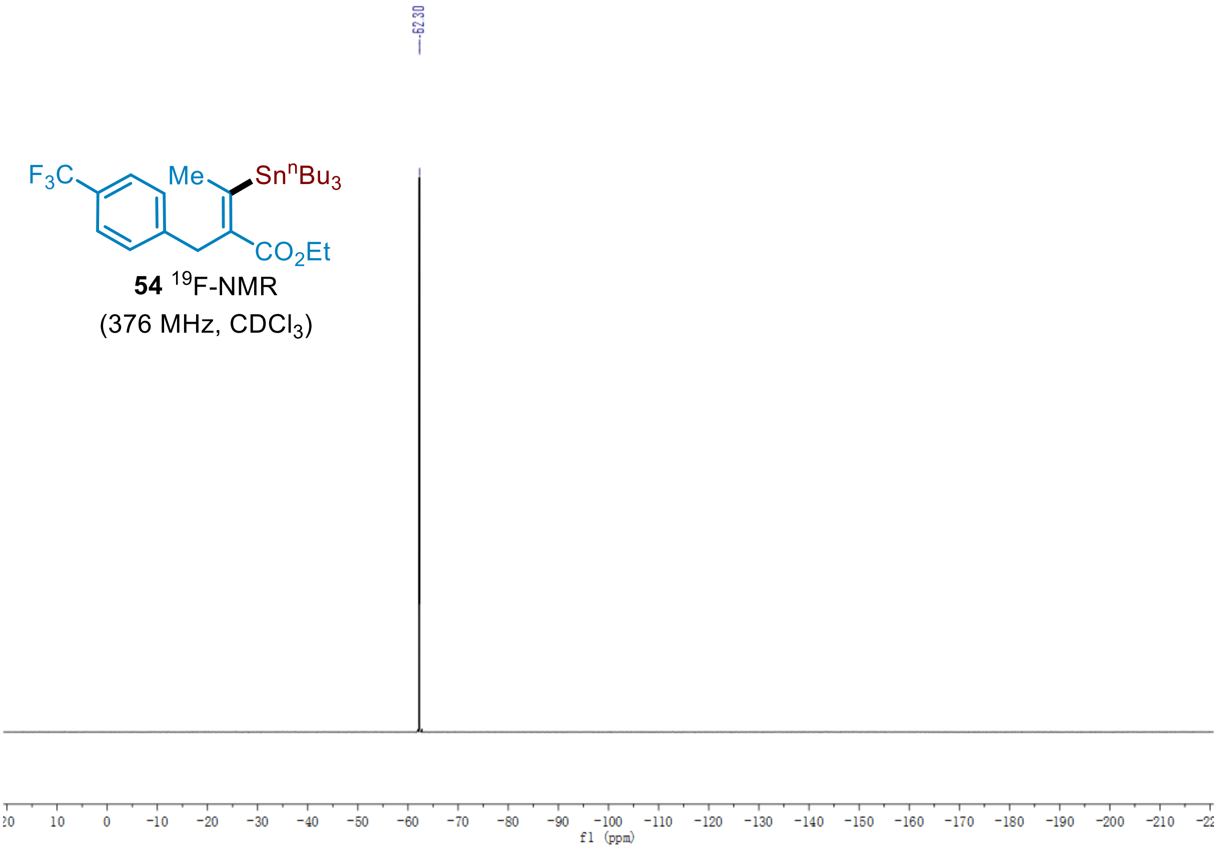

Supplementary Figure 208. **^{19}F-NMR** (376 MHz, $CDCl_3$, 298K) of **54**

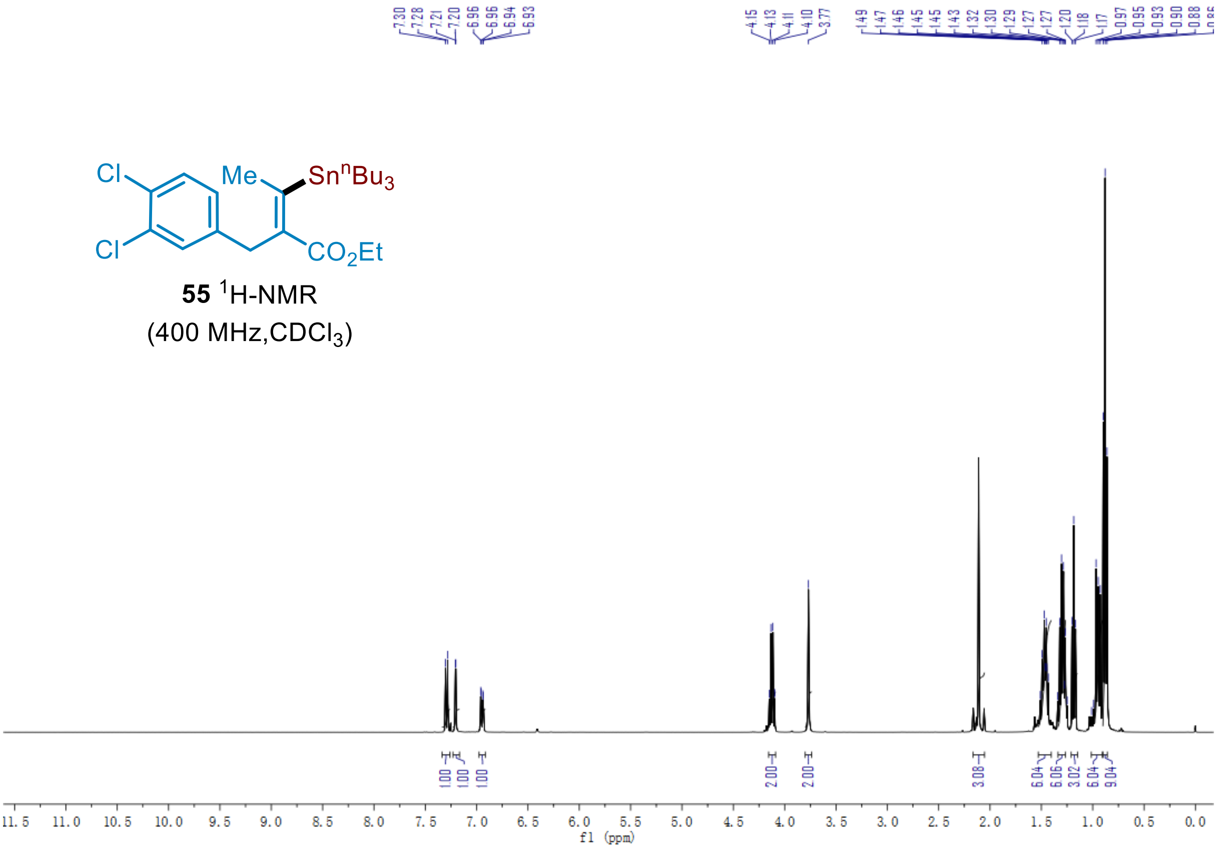

Supplementary Figure 209. **^{1}H-NMR** (400 MHz, $CDCl_3$, 298K) of **55**

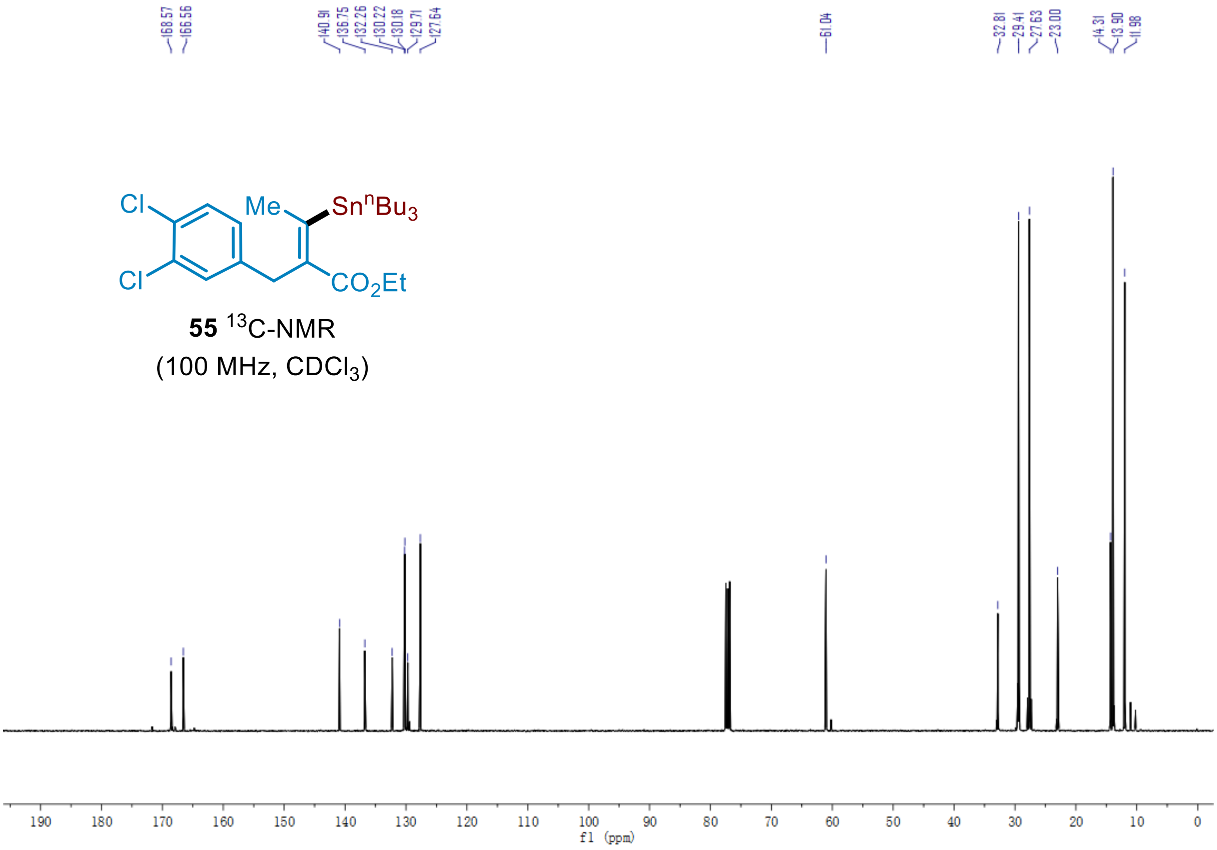

Supplementary Figure 210. **^{13}C-NMR** (100 MHz, $CDCl_3$, 298K) of **55**

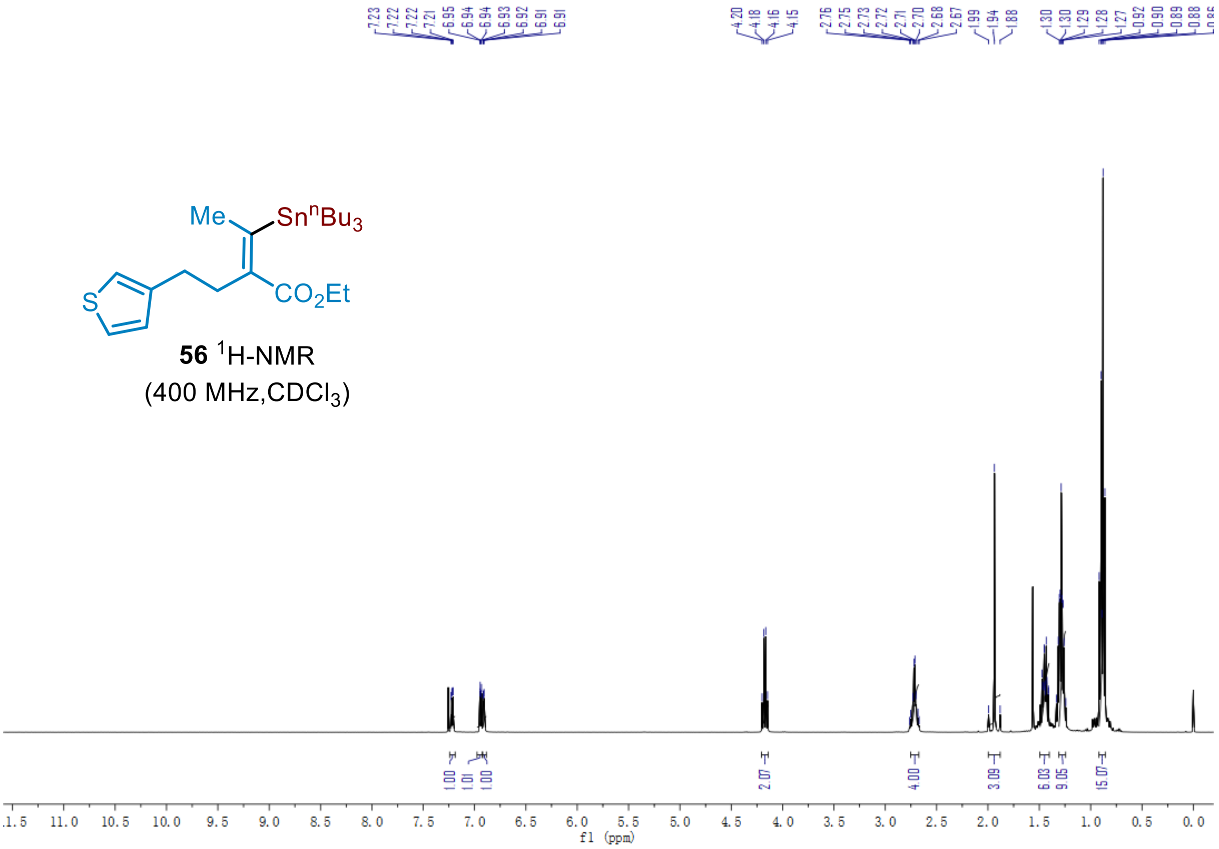

Supplementary Figure 211. **^{1}H-NMR** (400 MHz, $CDCl_3$, 298K) of **56**

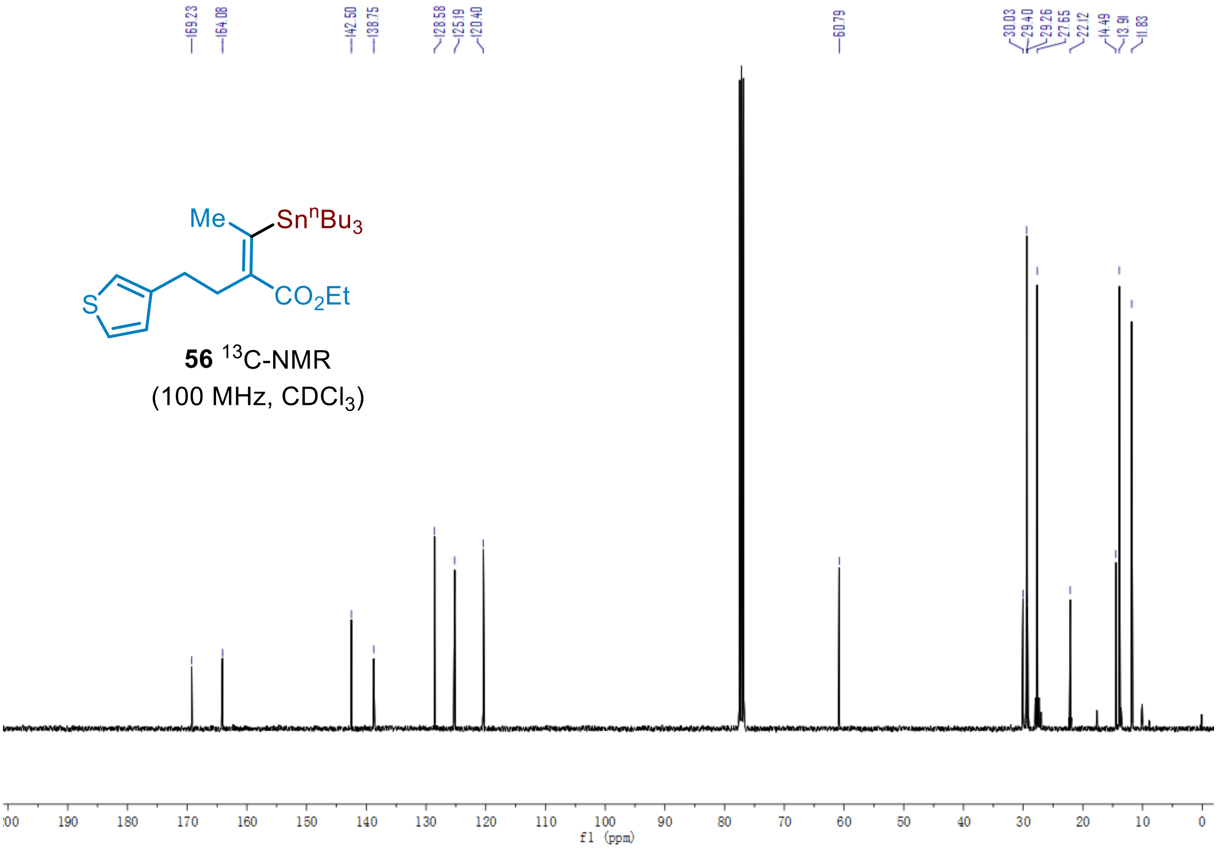

Supplementary Figure 212. **^{13}C-NMR** (100 MHz, $CDCl_3$, 298K) of **56**

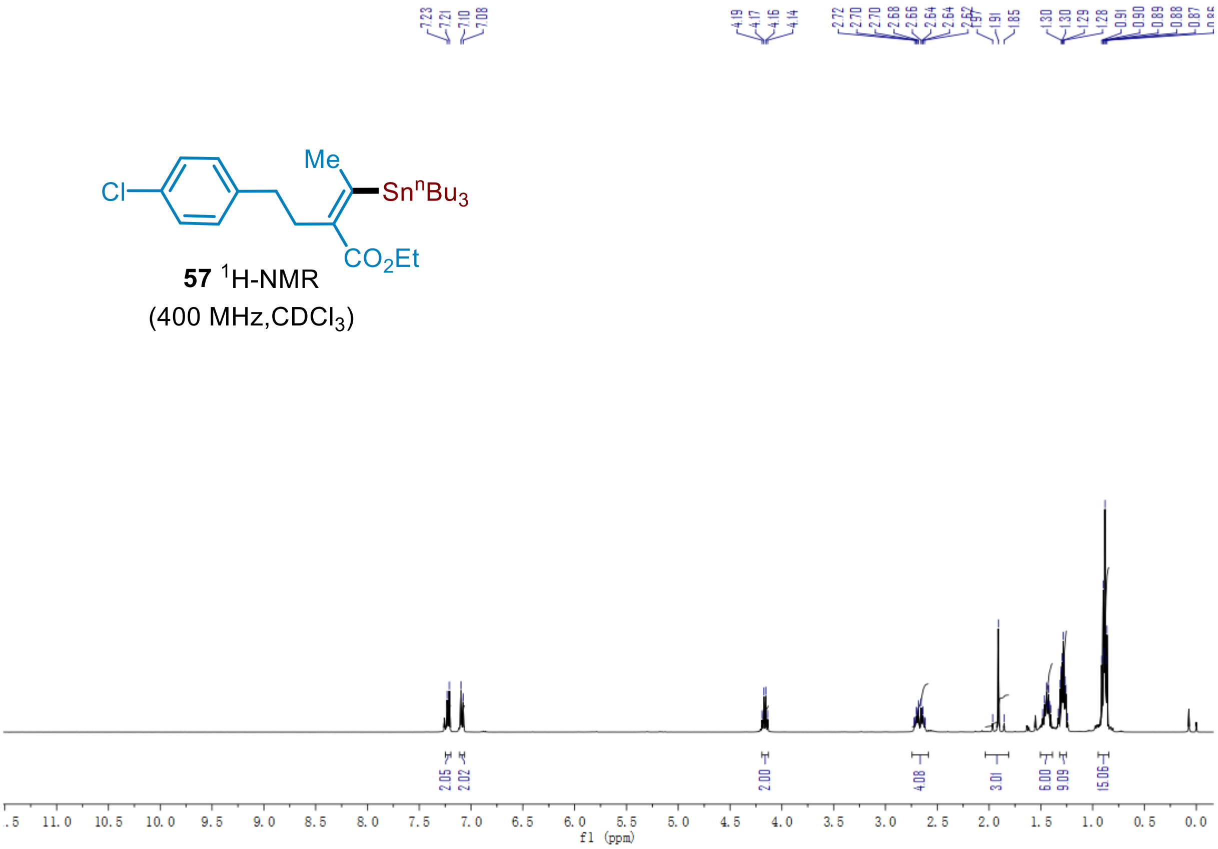

Supplementary Figure 213. **^{1}H-NMR** (400 MHz, CDCl$_3$, 298K) of **57**

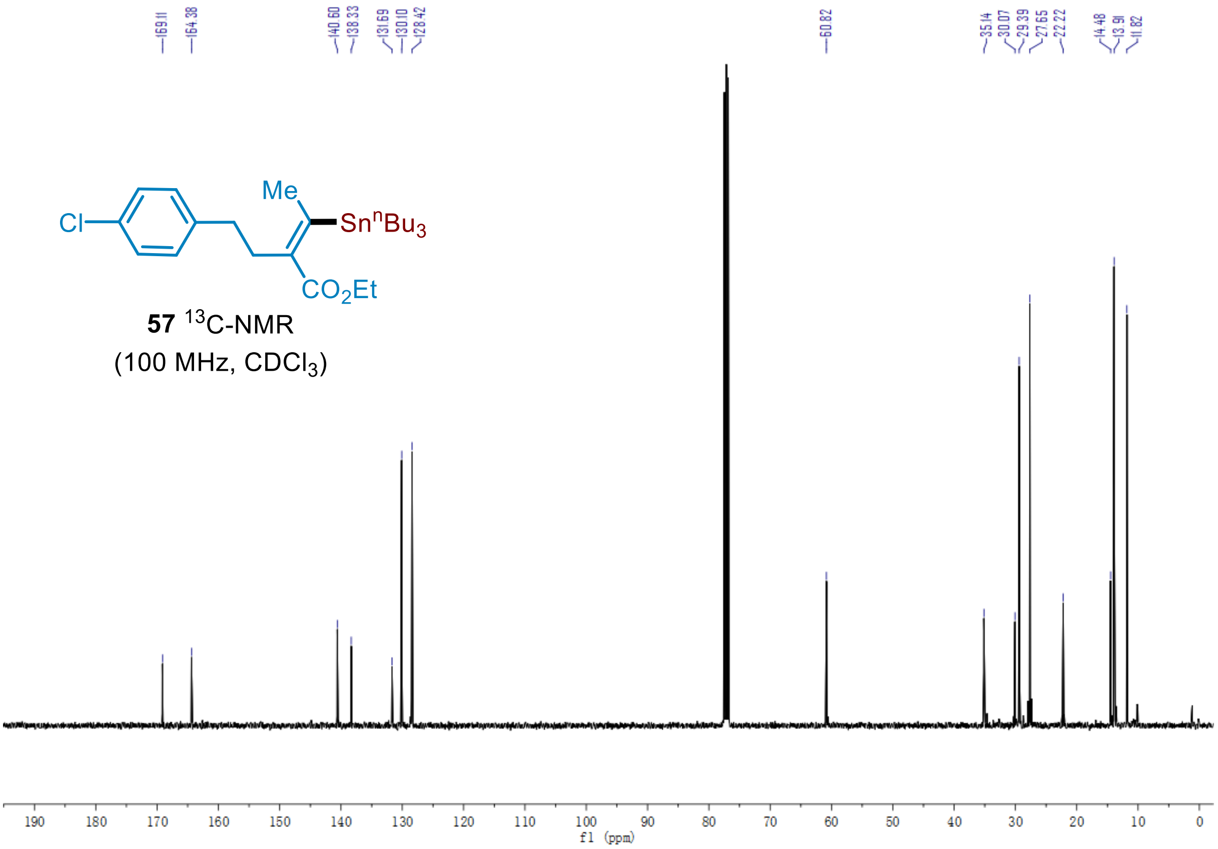

Supplementary Figure 214. **^{13}C-NMR** (100 MHz, CDCl$_3$, 298K) of **57**

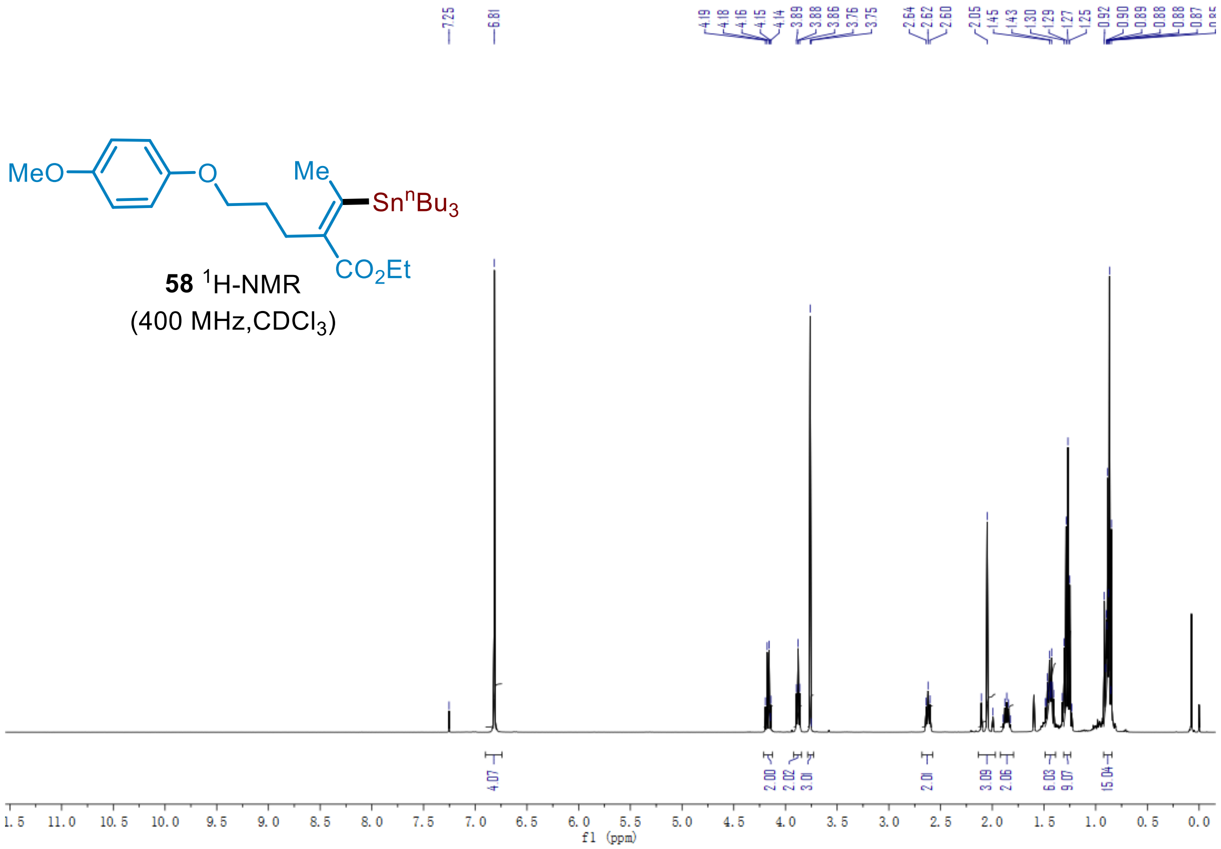

Supplementary Figure 215. **^{1}H-NMR** (400 MHz, $CDCl_3$, 298K) of **58**

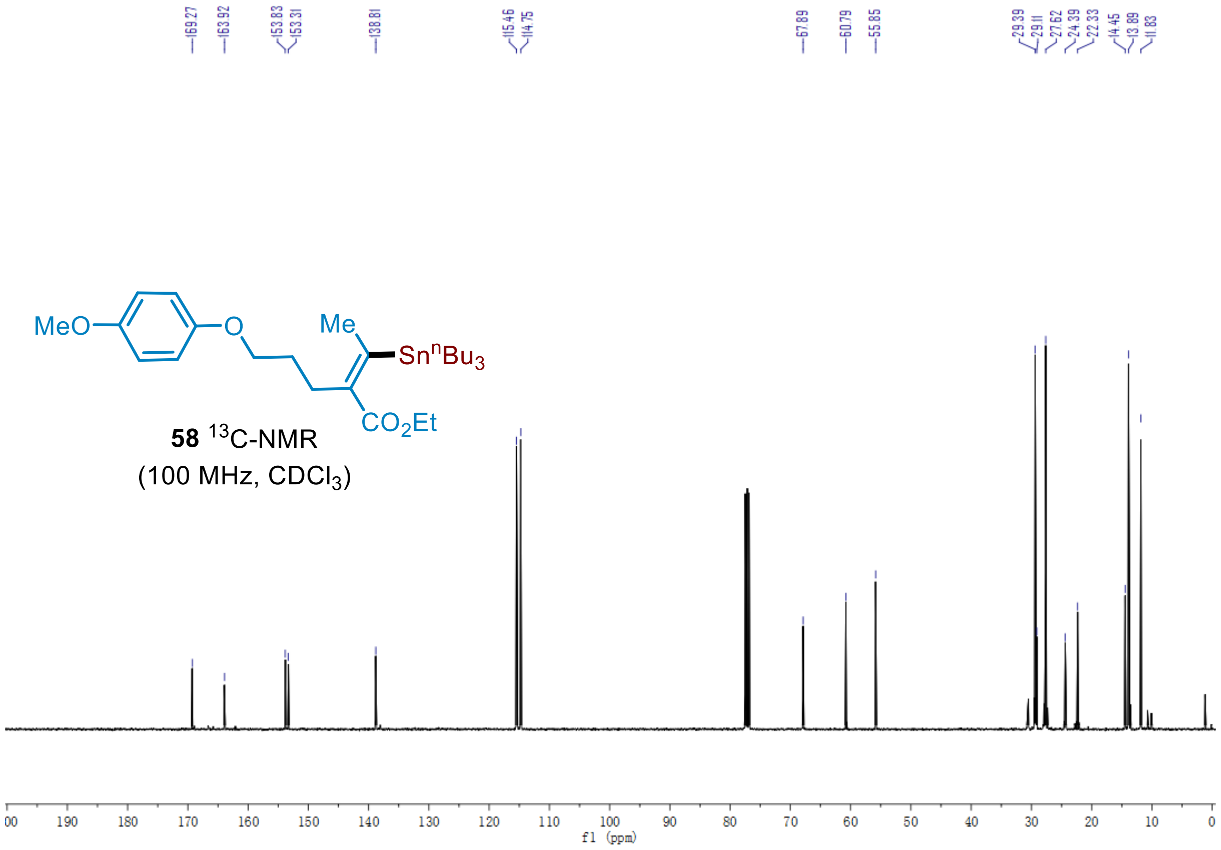

Supplementary Figure 216. **^{13}C-NMR** (100 MHz, $CDCl_3$, 298K) of **58**

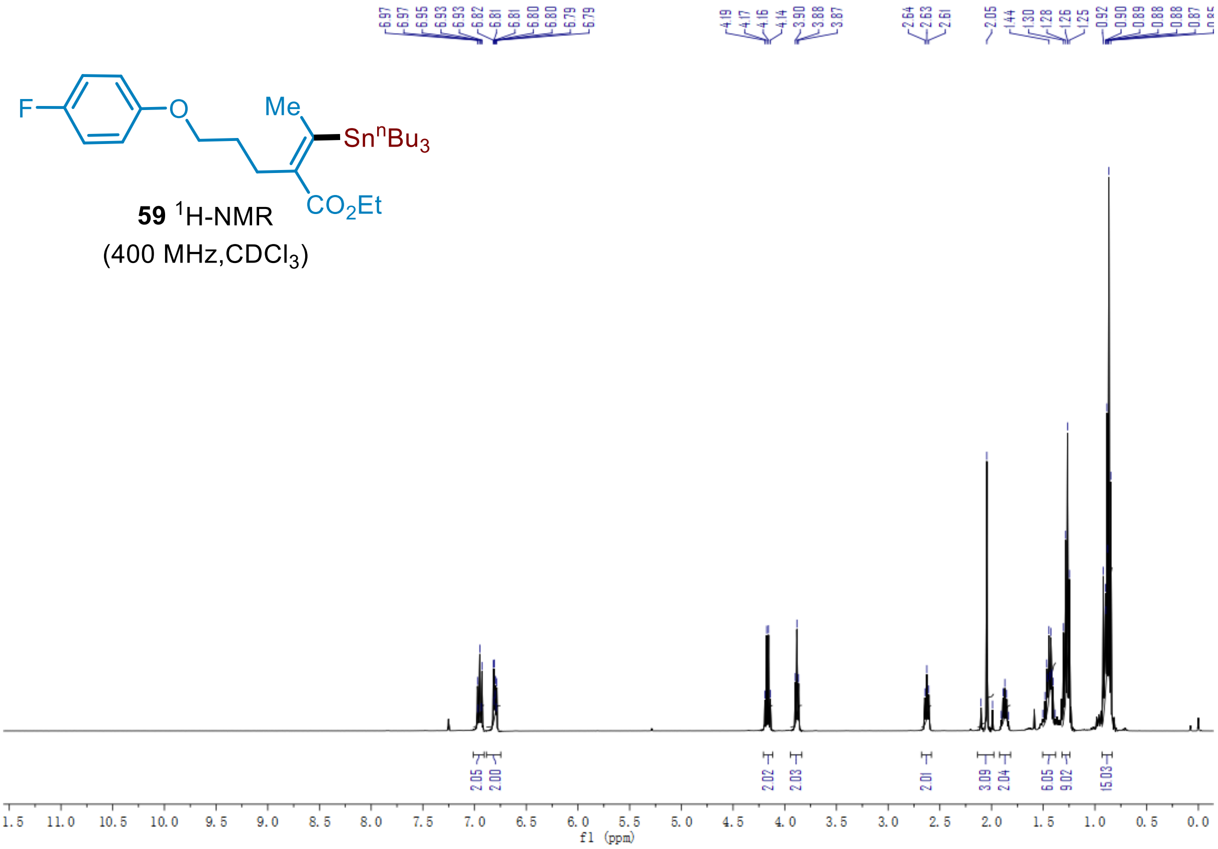

Supplementary Figure 217. **^{1}H-NMR** (400 MHz, $CDCl_3$, 298K) of **59**

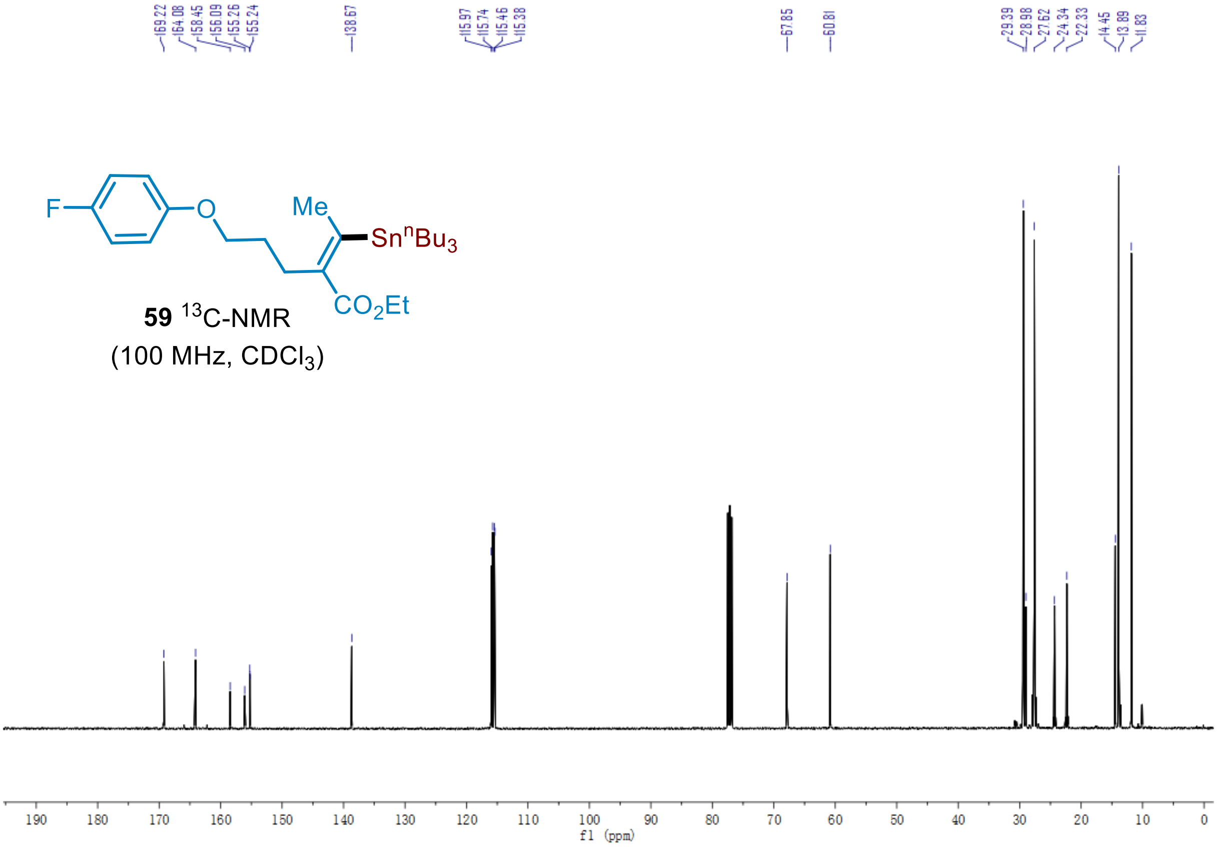

Supplementary Figure 218. **^{13}C-NMR** (100 MHz, $CDCl_3$, 298K) of **59**

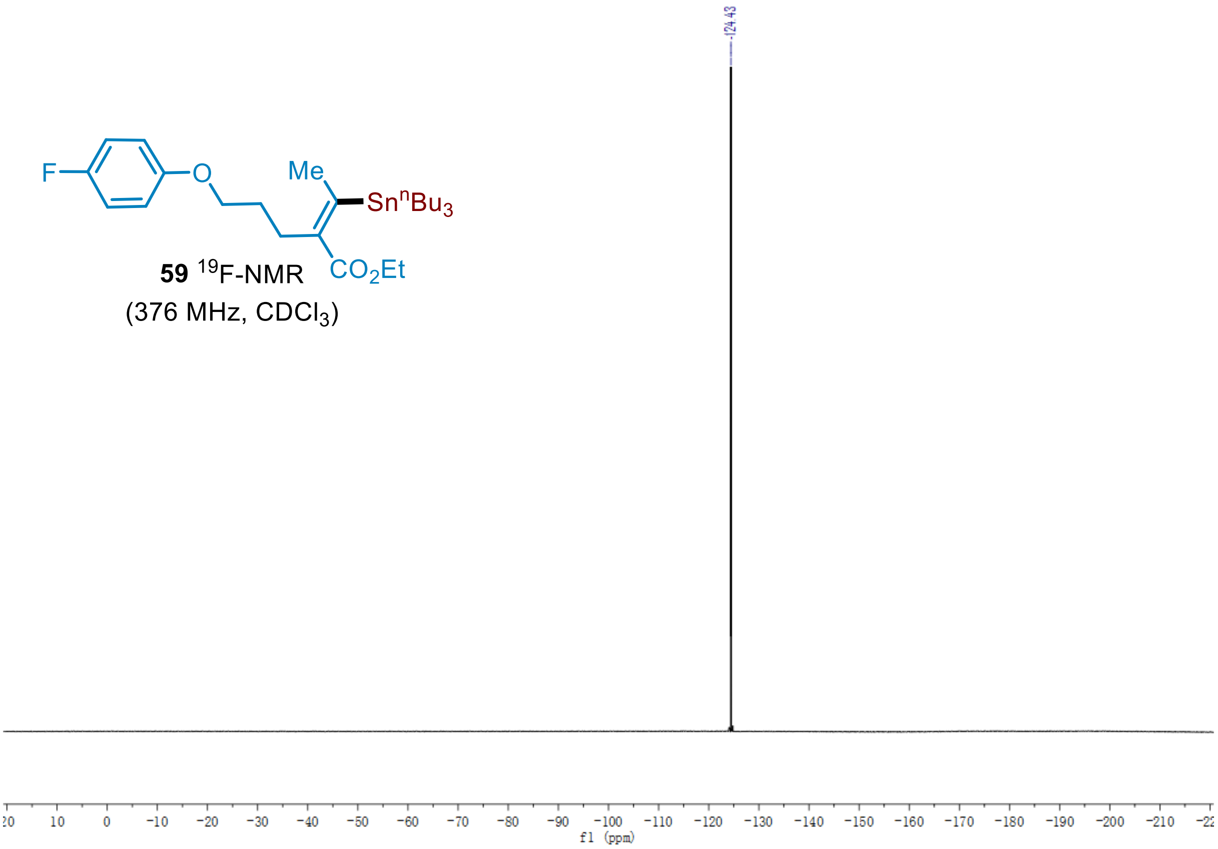

Supplementary Figure 219. **^{19}F-NMR** (376 MHz, $CDCl_3$, 298K) of **59**

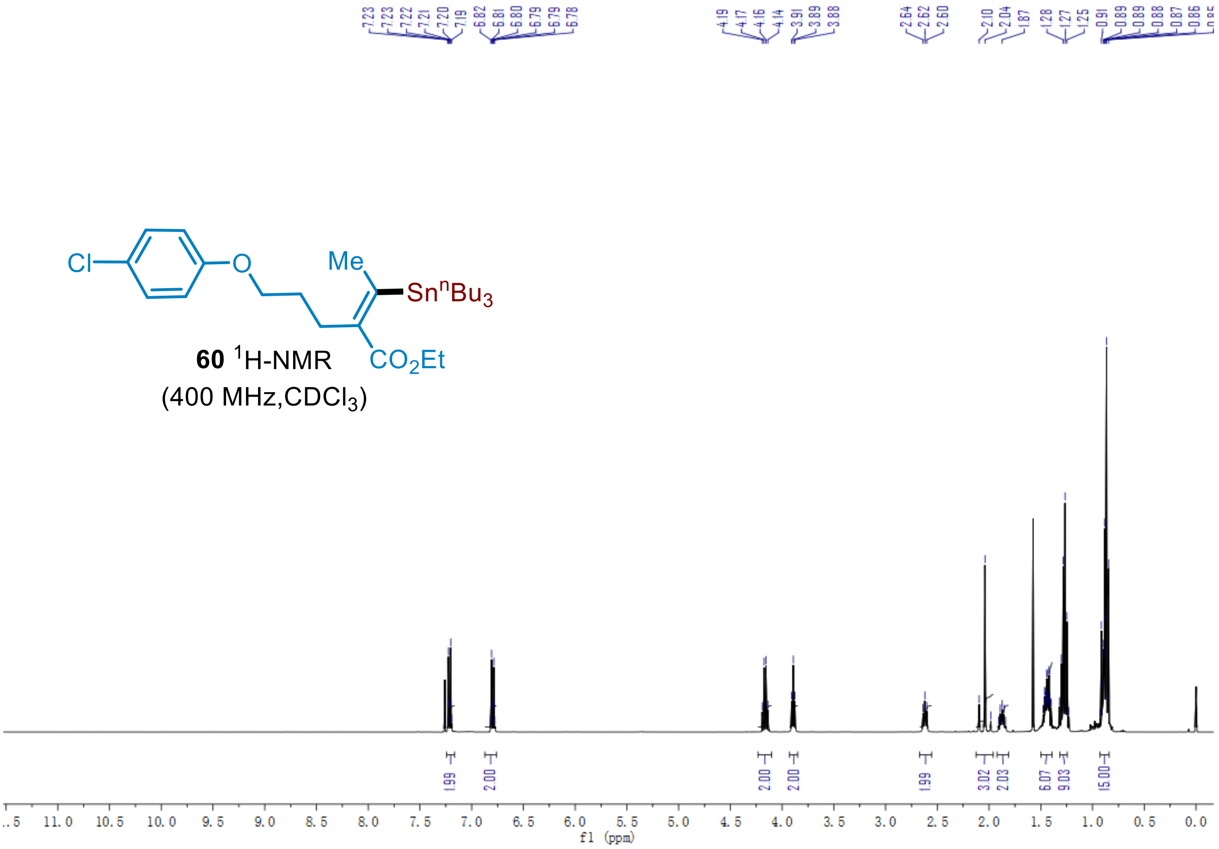

Supplementary Figure 220. **^{1}H-NMR** (400 MHz, $CDCl_3$, 298K) of **60**

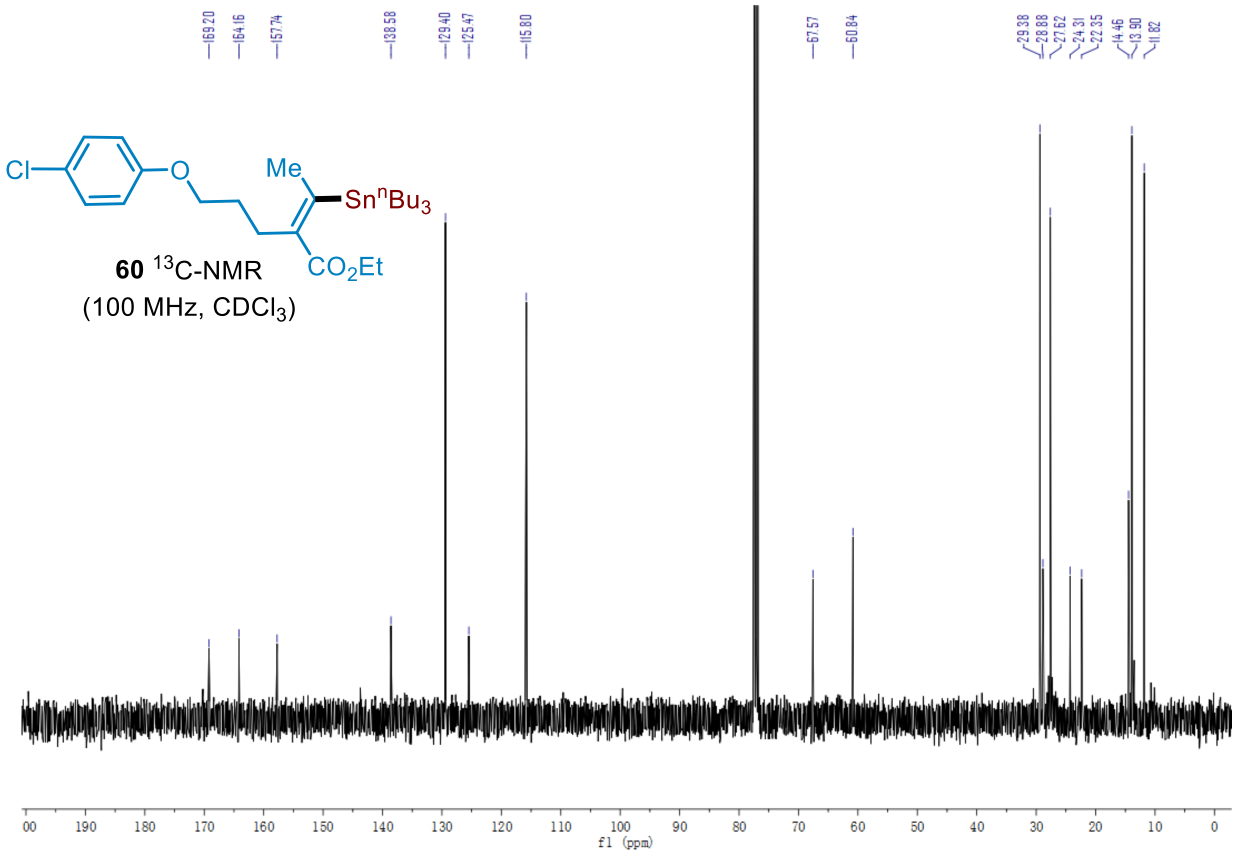

Supplementary Figure 221. **^{13}C-NMR** (100 MHz, $CDCl_3$, 298K) of **60**

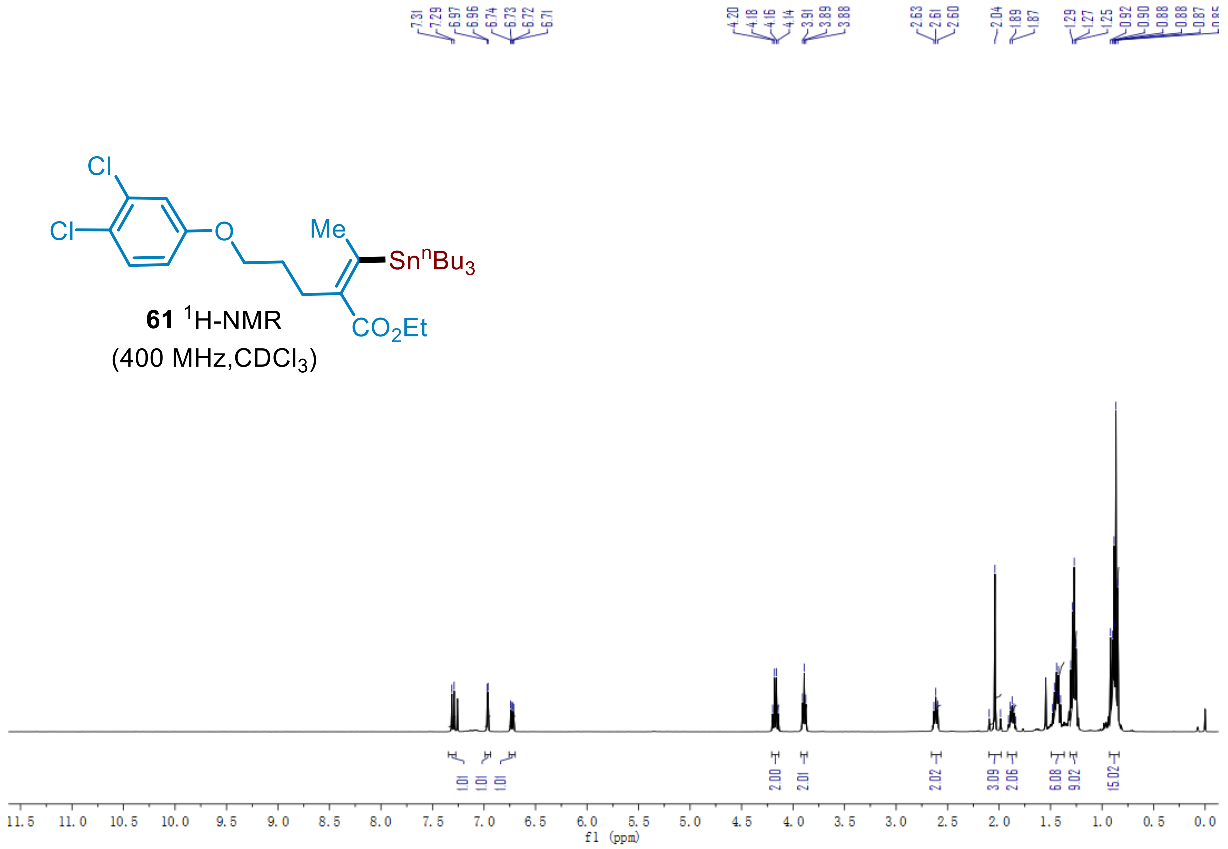

Supplementary Figure 222. **^{1}H-NMR** (400 MHz, $CDCl_3$, 298K) of **61**

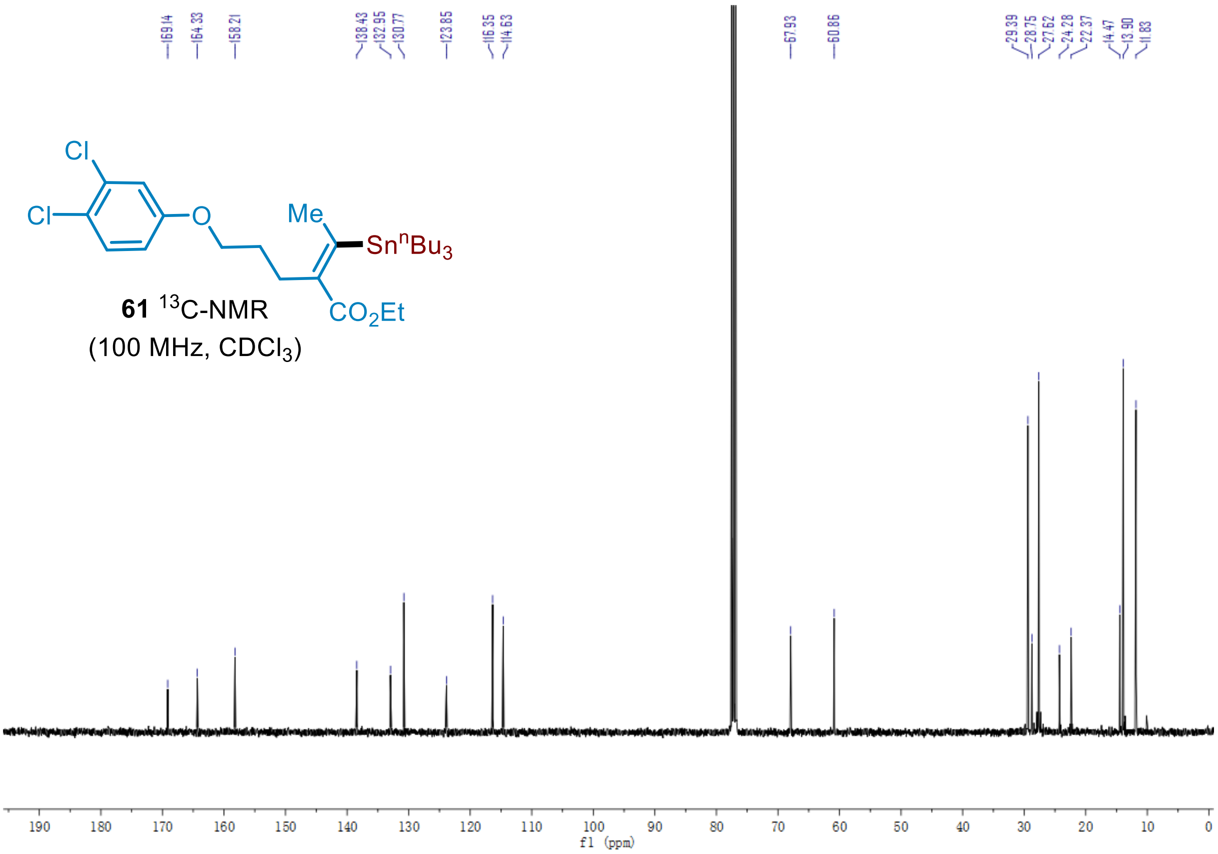

Supplementary Figure 223. **^{13}C-NMR** (100 MHz, $CDCl_3$, 298K) of **61**

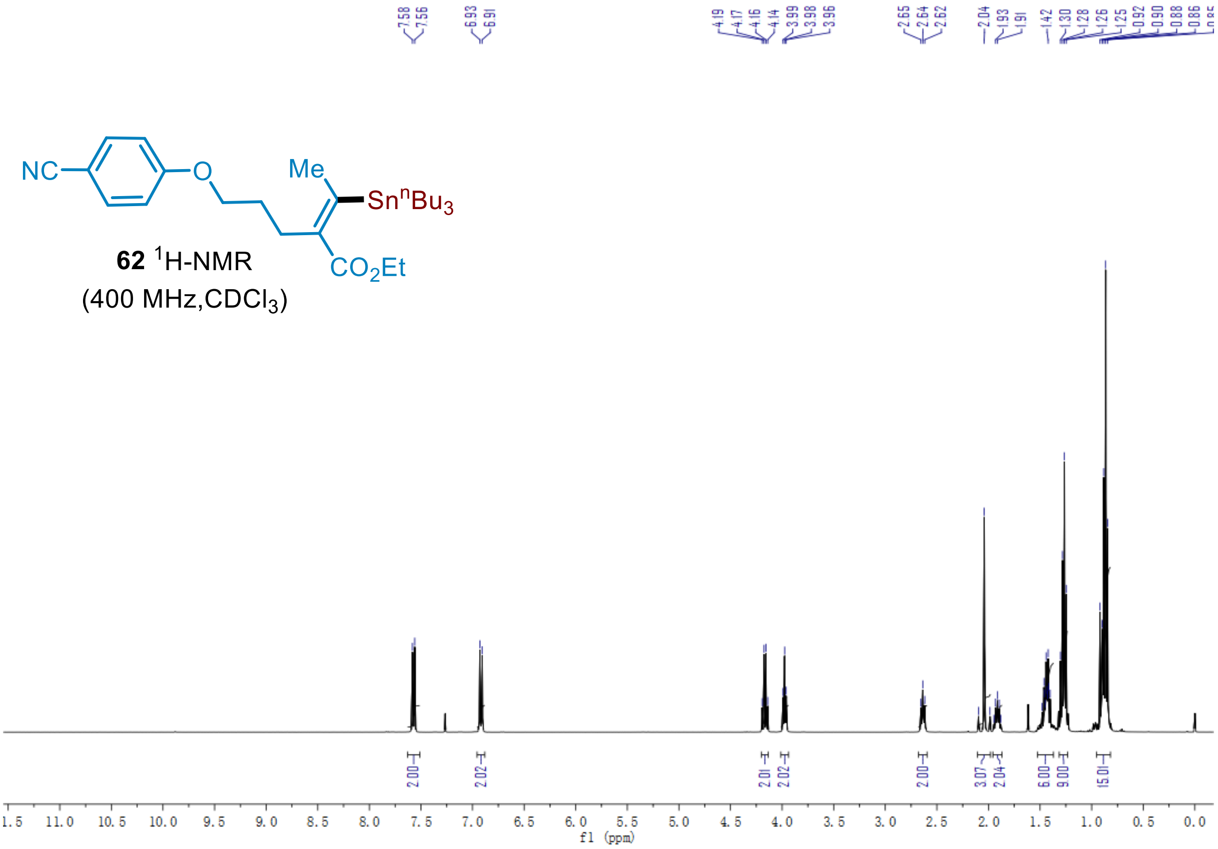

Supplementary Figure 224. **^{1}H-NMR** (400 MHz, $CDCl_3$, 298K) of **62**

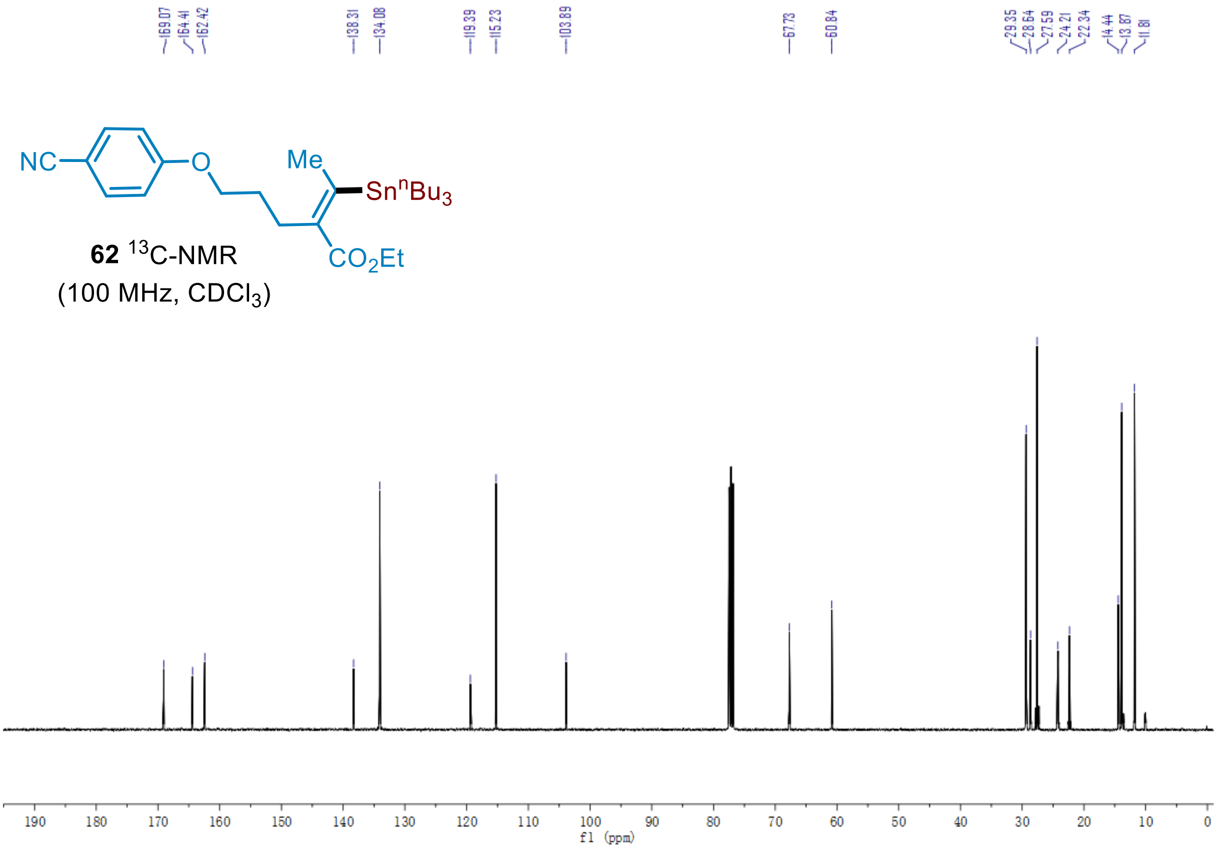

Supplementary Figure 225. **^{13}C-NMR** (100 MHz, $CDCl_3$, 298K) of **62**

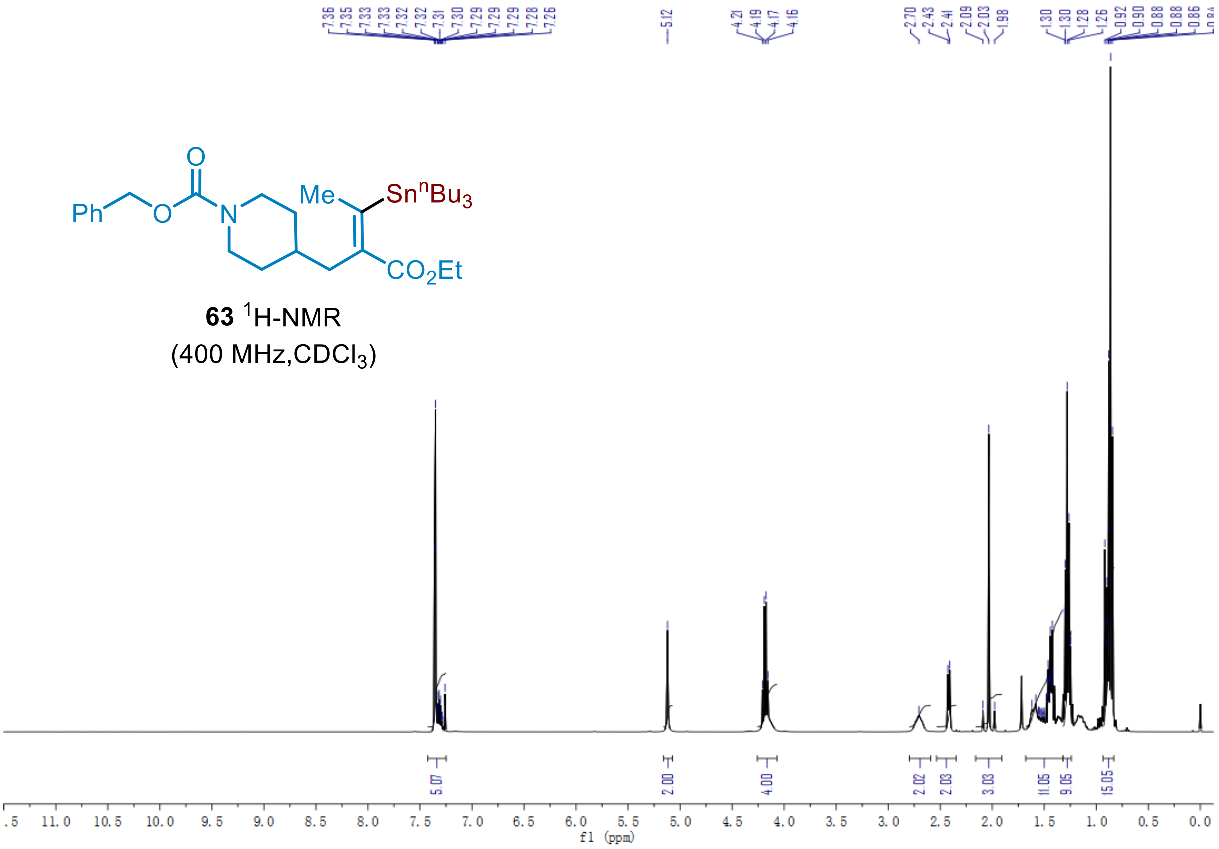

Supplementary Figure 226. **^{1}H-NMR** (400 MHz, $CDCl_3$, 298K) of **63**

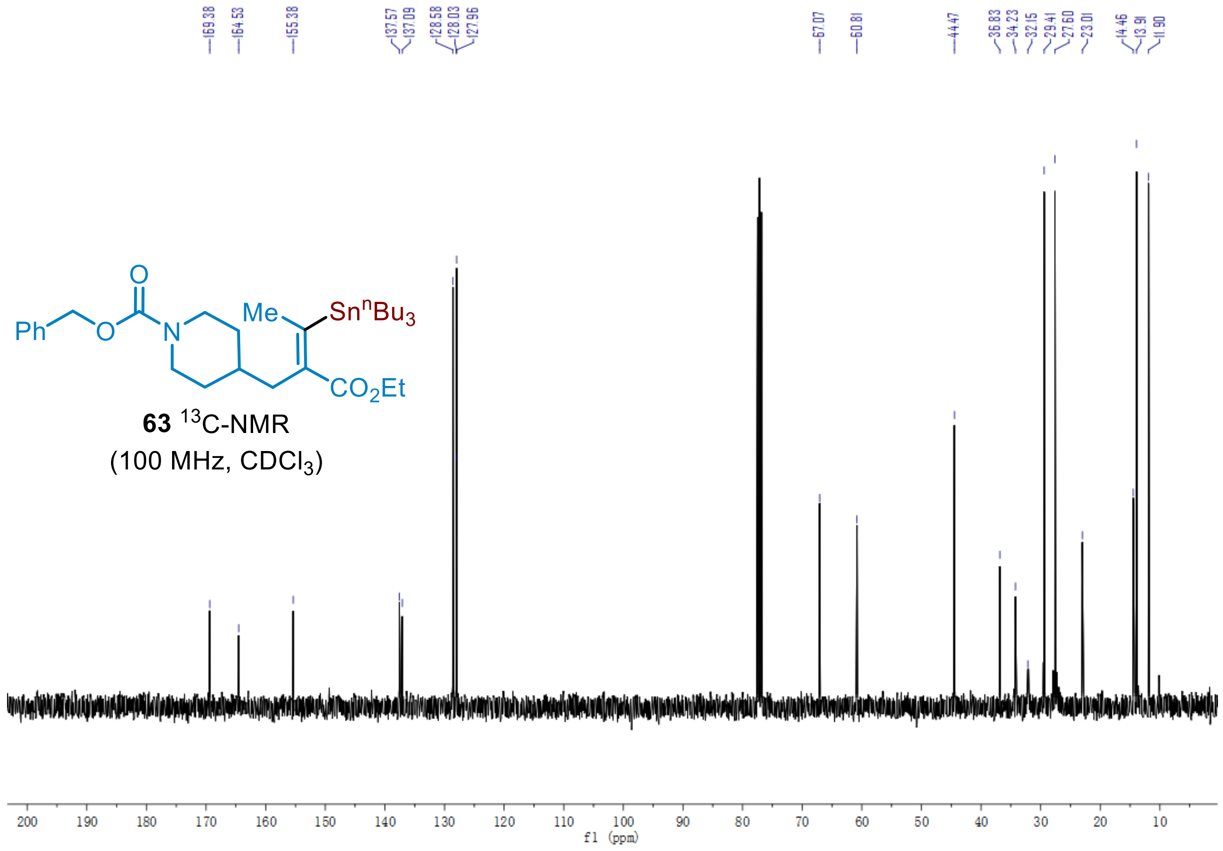

Supplementary Figure 227. **^{13}C-NMR** (100 MHz, $CDCl_3$, 298K) of **63**

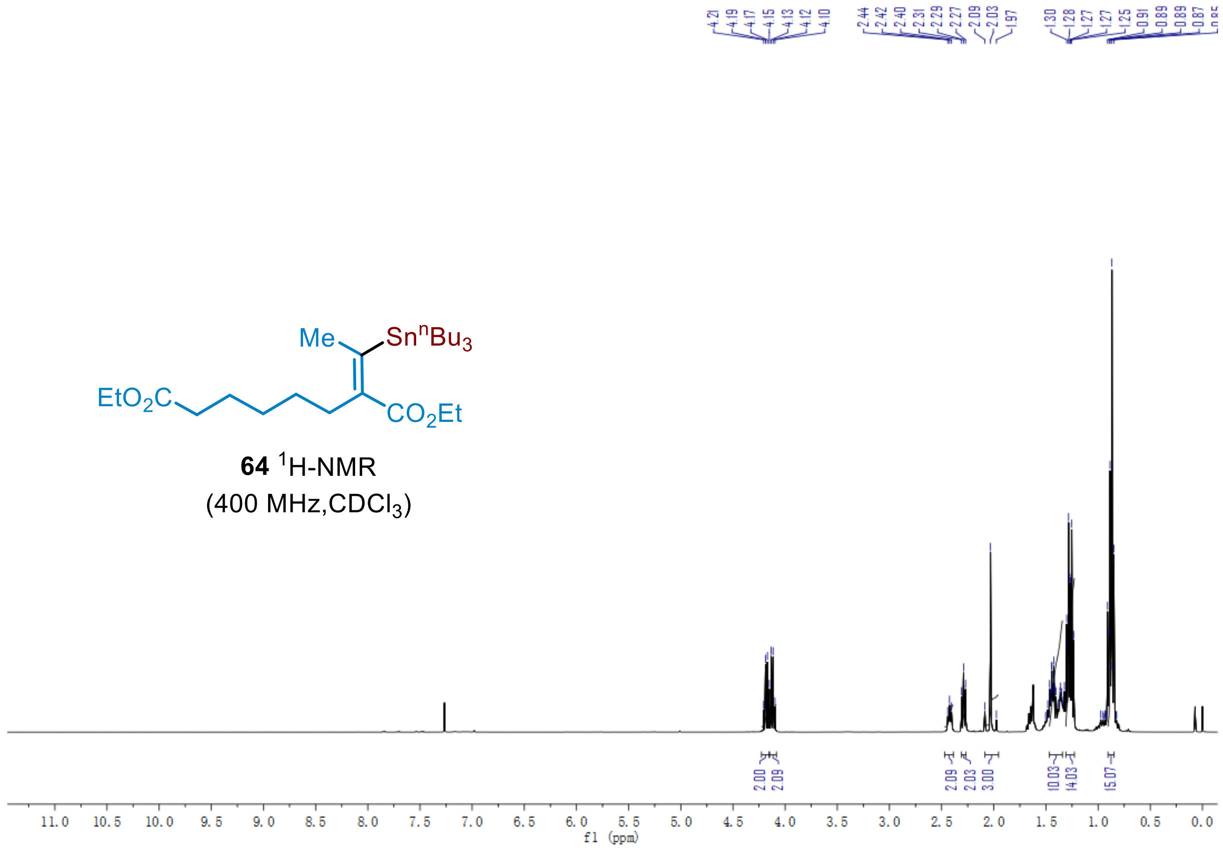

Supplementary Figure 228. **^{1}H-NMR** (400 MHz, $CDCl_3$, 298K) of **64**

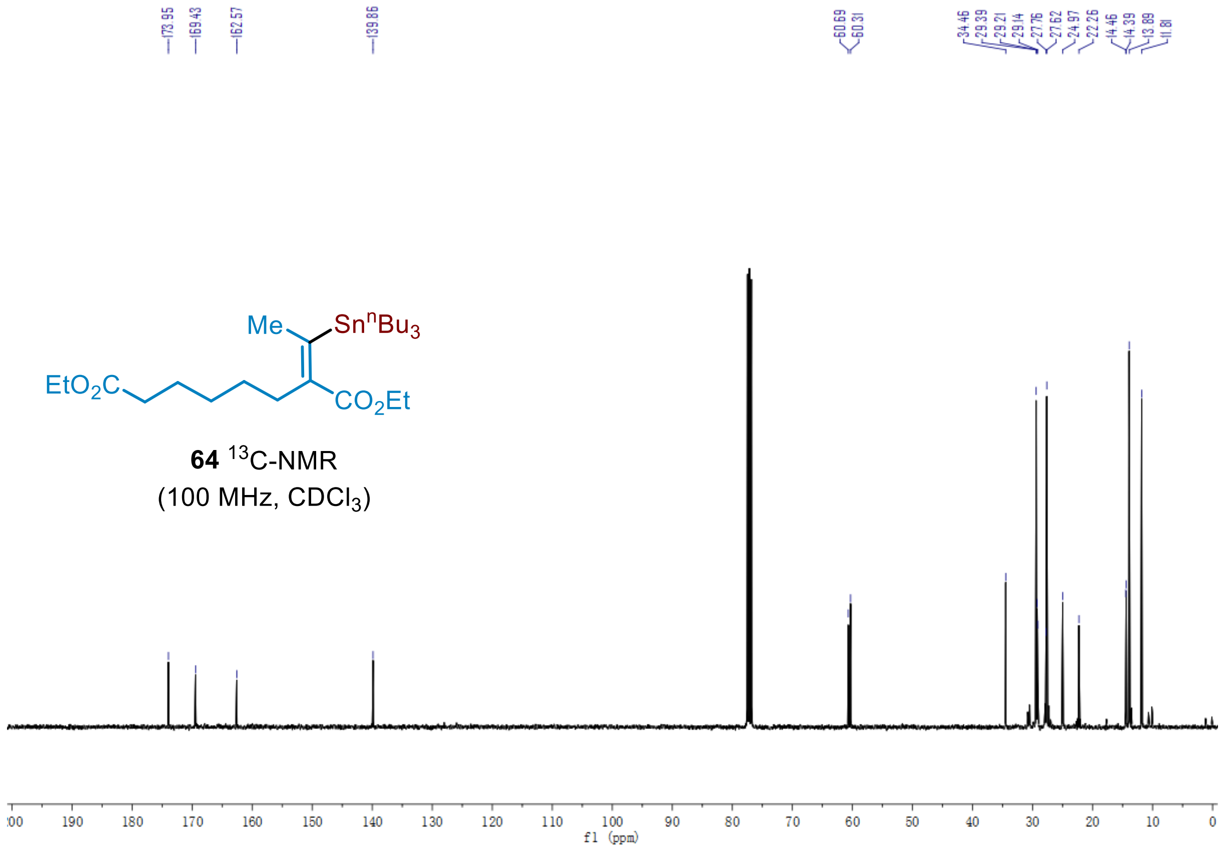

Supplementary Figure 229. **^{13}C-NMR** (100 MHz, $CDCl_3$, 298K) of **64**

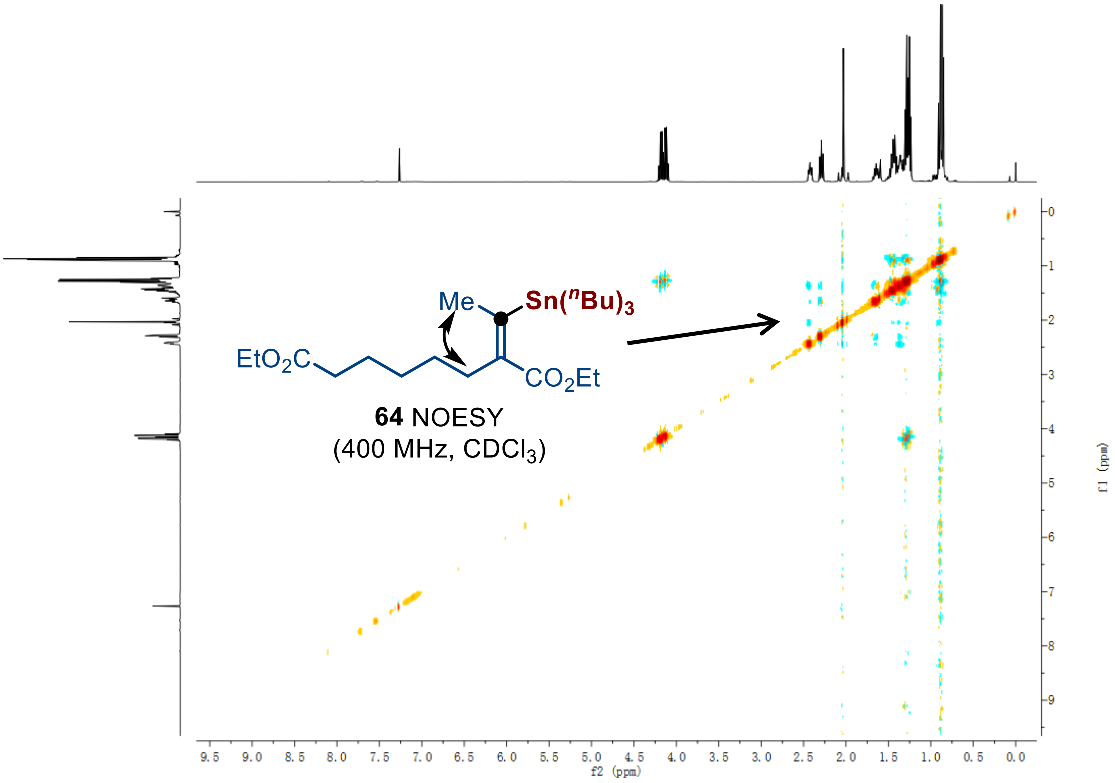

Supplementary Figure 230. NOESY of **64**

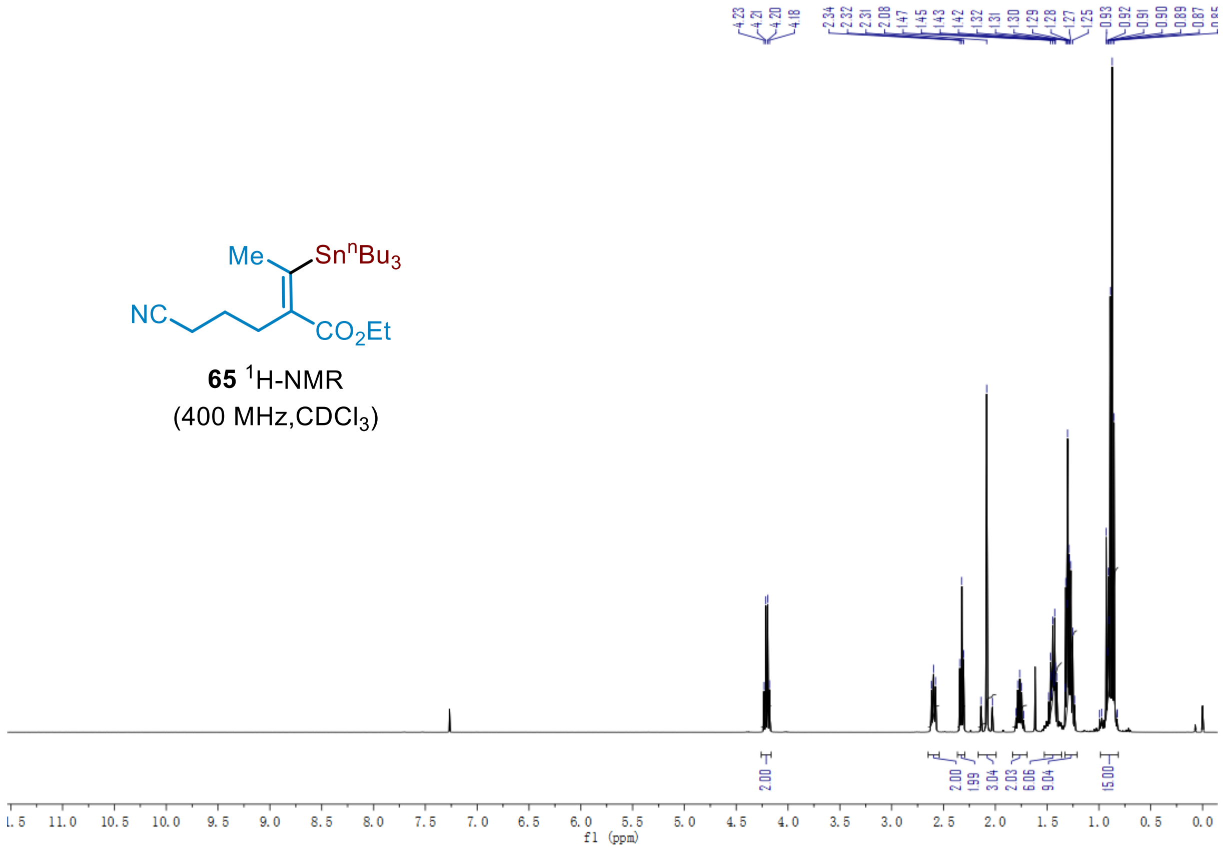

Supplementary Figure 231. **^{1}H-NMR** (400 MHz, CDCl$_3$, 298K) of **65**

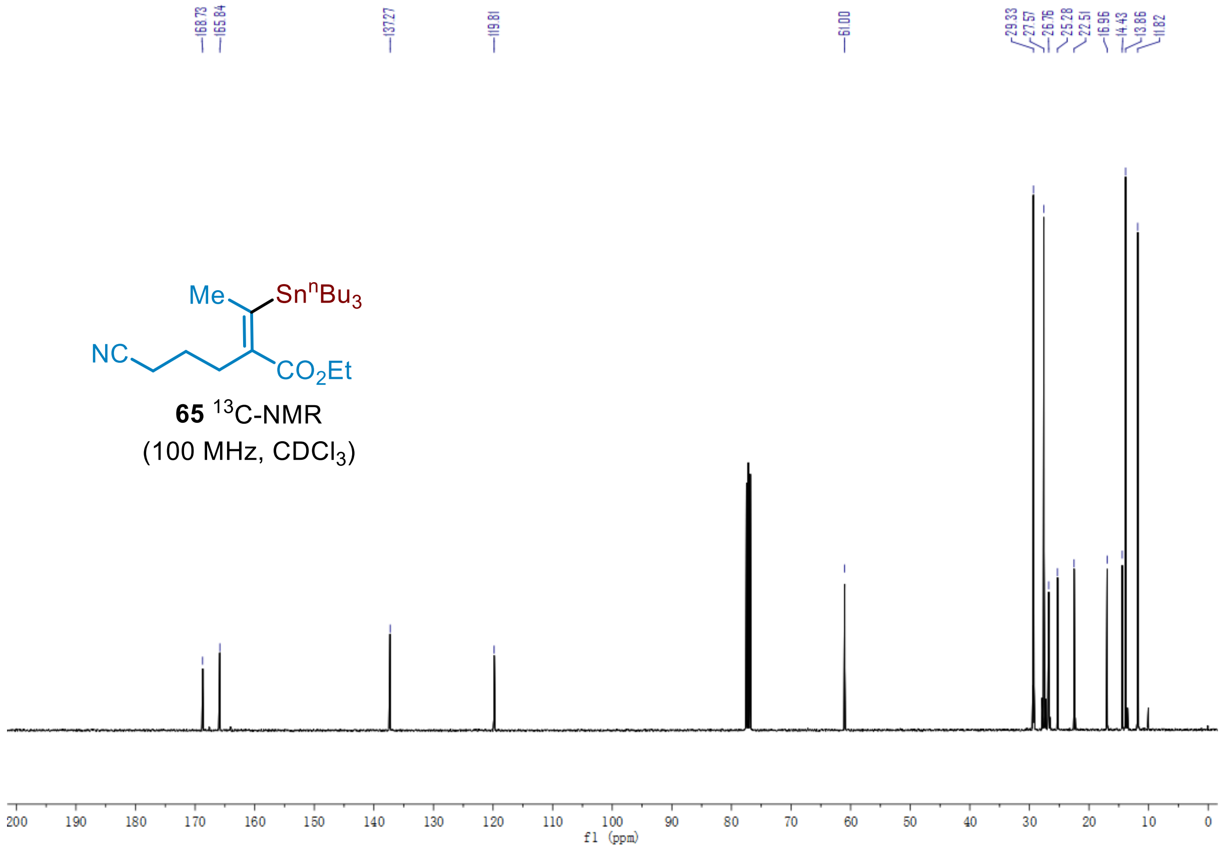

Supplementary Figure 232. **^{13}C-NMR** (100 MHz, CDCl$_3$, 298K) of **65**

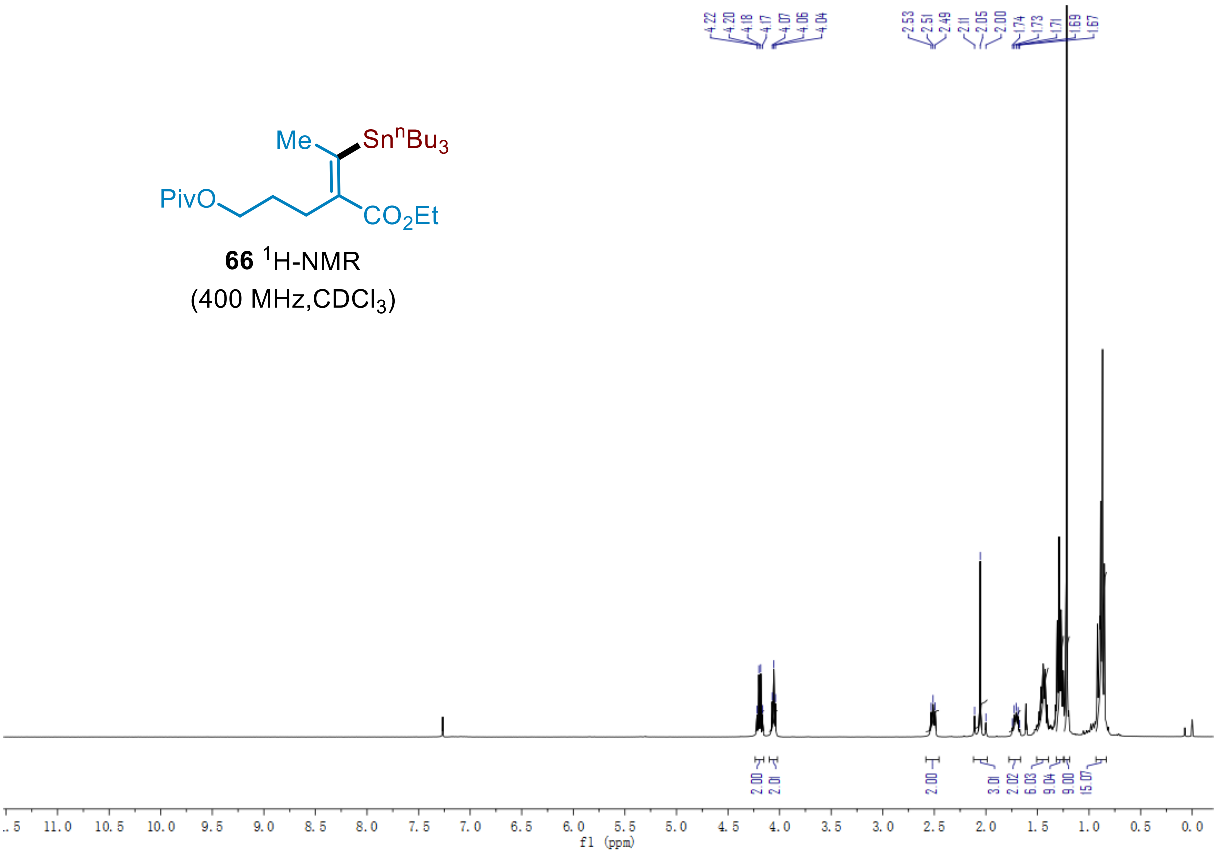

Supplementary Figure 233. **^{1}H-NMR** (400 MHz, $CDCl_3$, 298K) of **66**

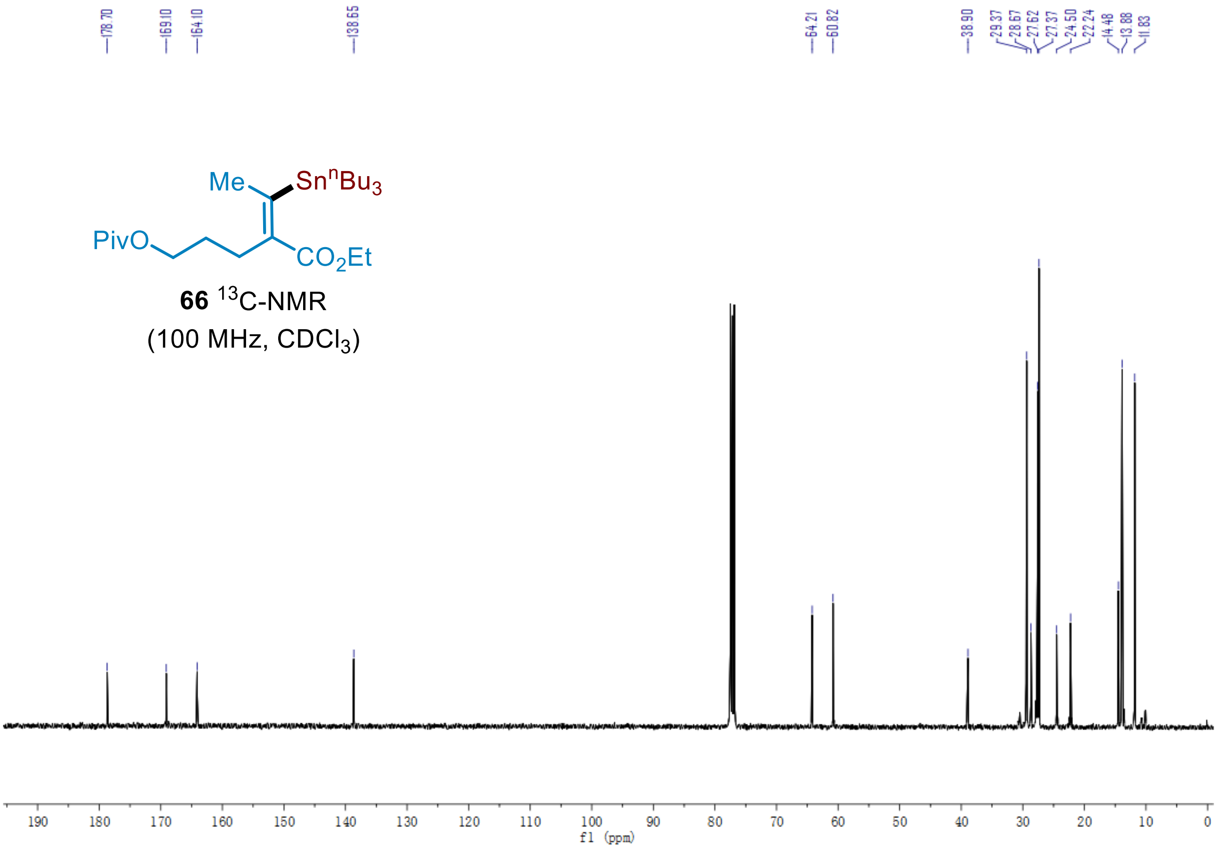

Supplementary Figure 234. **^{13}C-NMR** (100 MHz, $CDCl_3$, 298K) of **66**

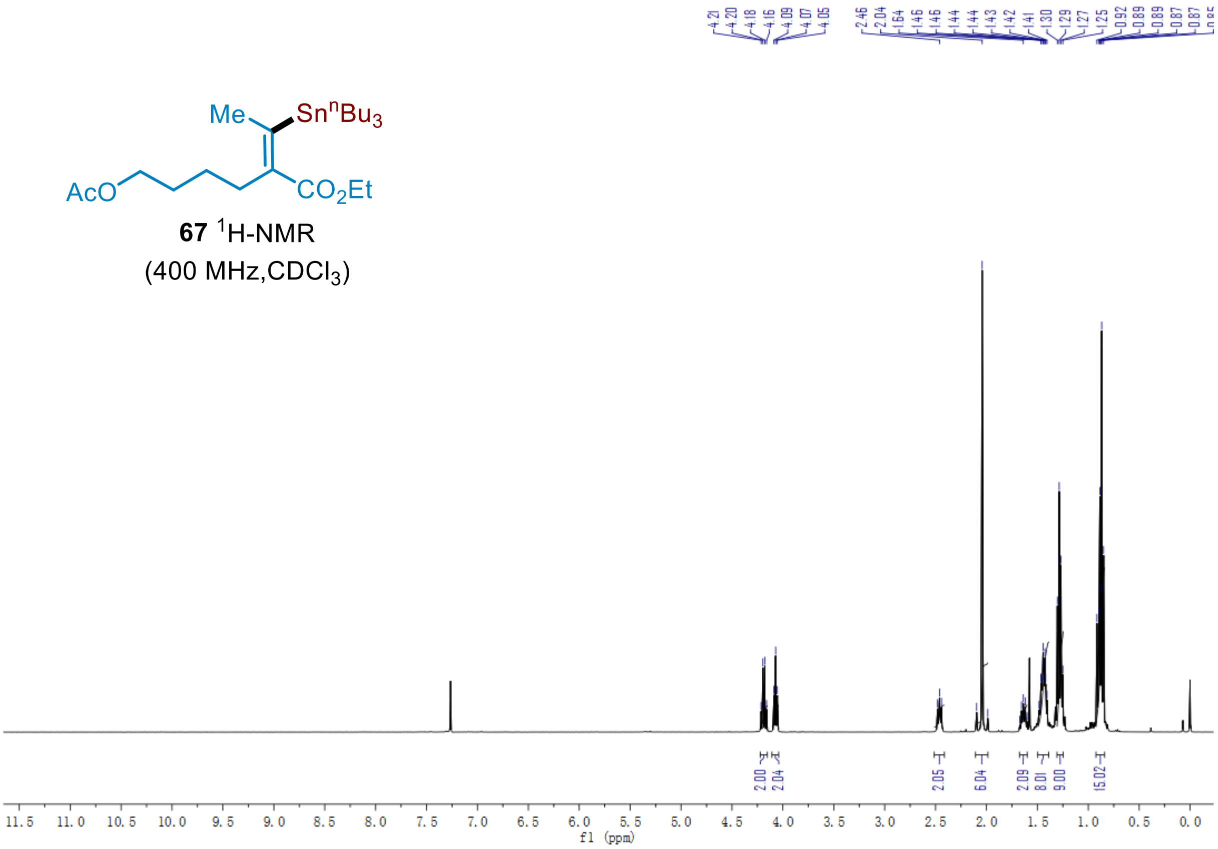

Supplementary Figure 235. **^{1}H-NMR** (400 MHz, $CDCl_3$, 298K) of **67**

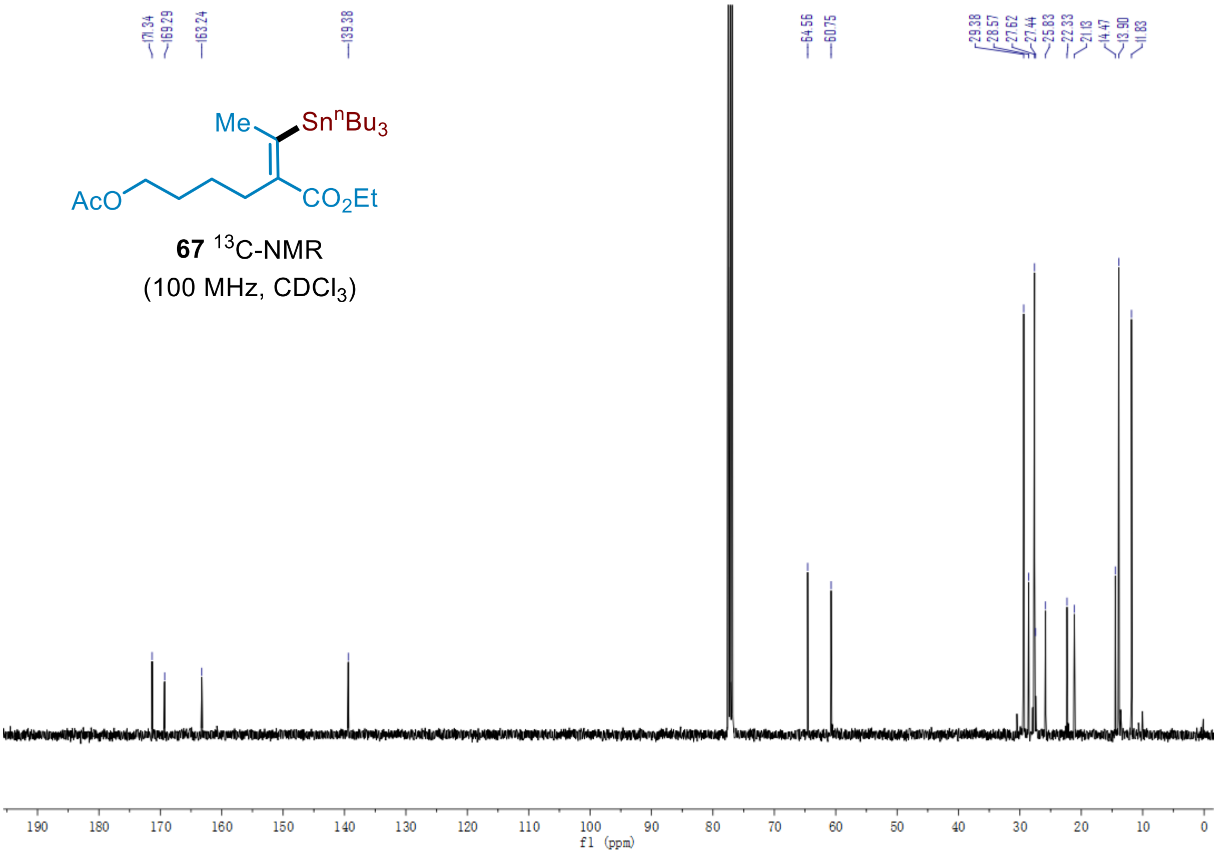

Supplementary Figure 236. **^{13}C-NMR** (100 MHz, $CDCl_3$, 298K) of **67**

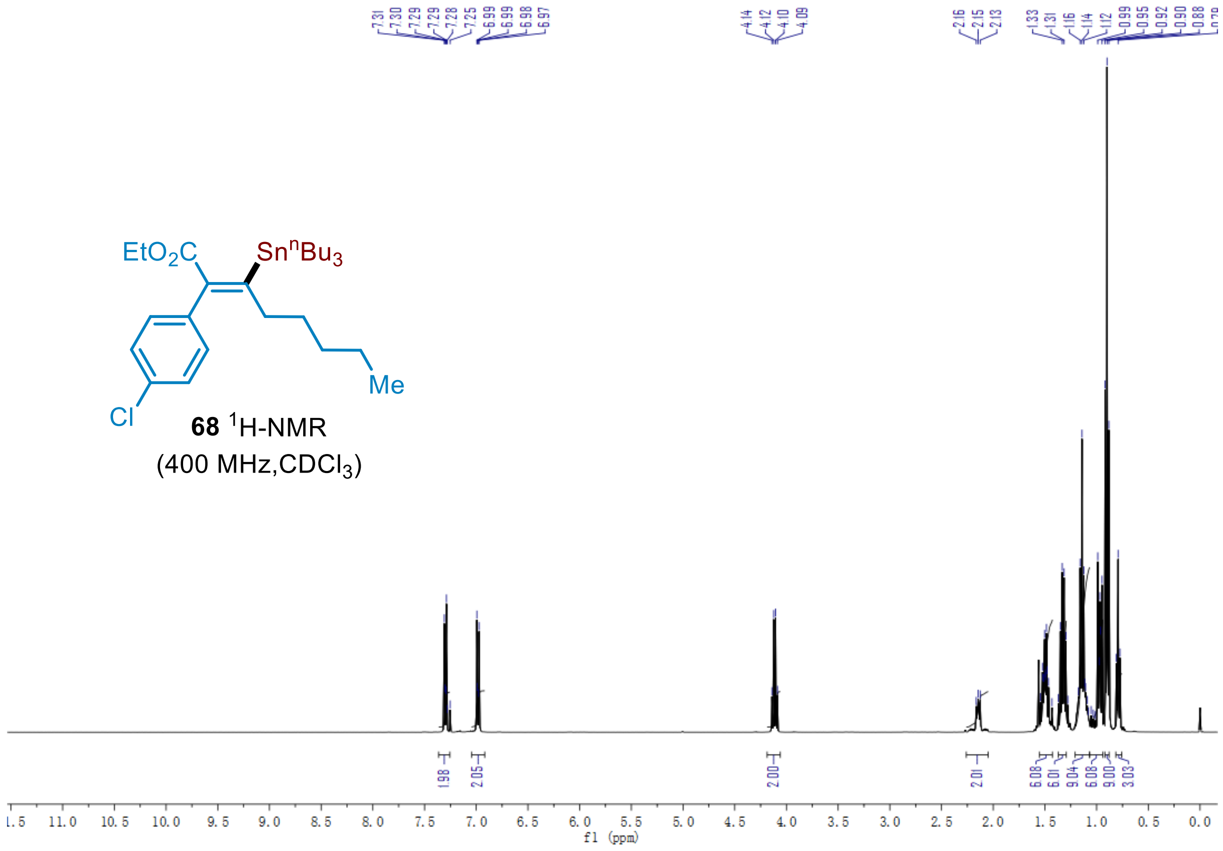

Supplementary Figure 237. **^{1}H-NMR** (400 MHz, $CDCl_3$, 298K) of **68**

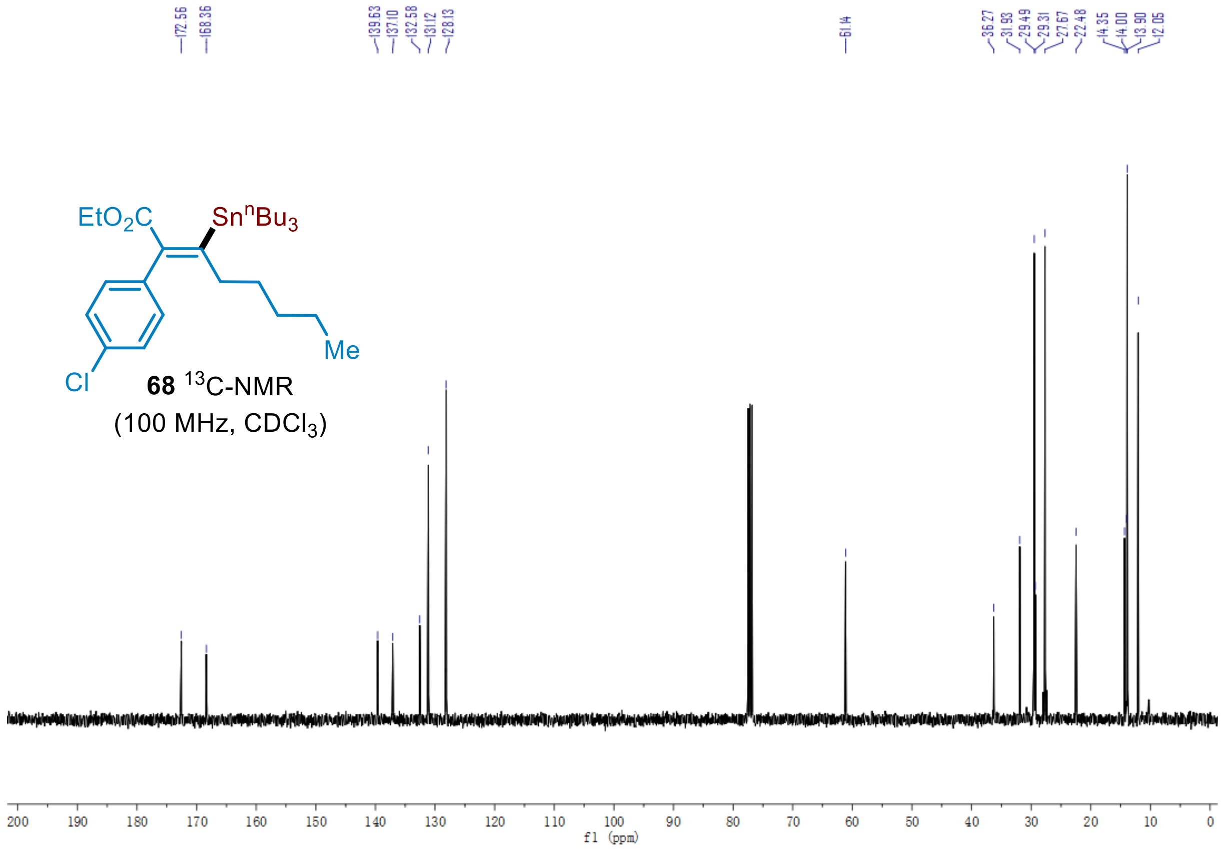

Supplementary Figure 238. **^{13}C-NMR** (100 MHz, $CDCl_3$, 298K) of **68**

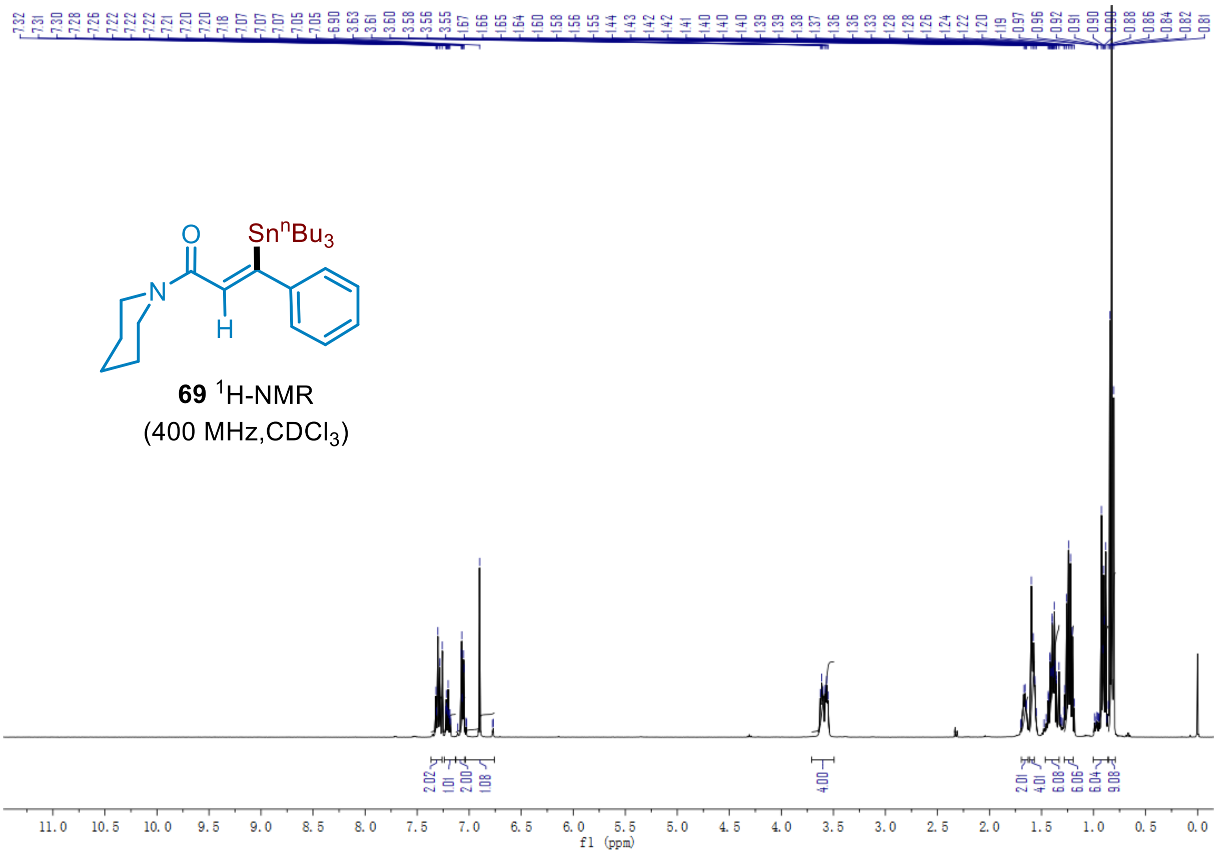

Supplementary Figure 239. **^{1}H-NMR** (400 MHz, $CDCl_3$, 298K) of **69**

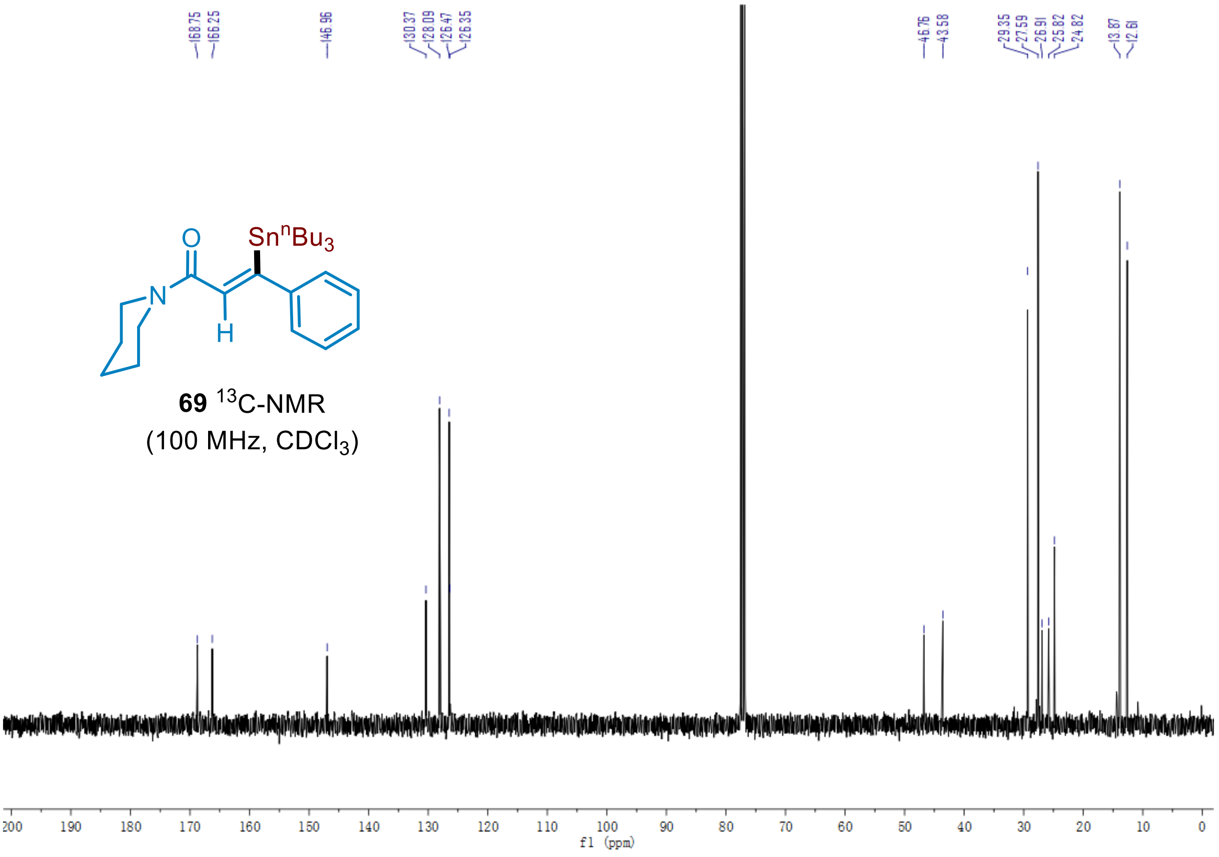

Supplementary Figure 240. **^{13}C-NMR** (100 MHz, $CDCl_3$, 298K) of **69**

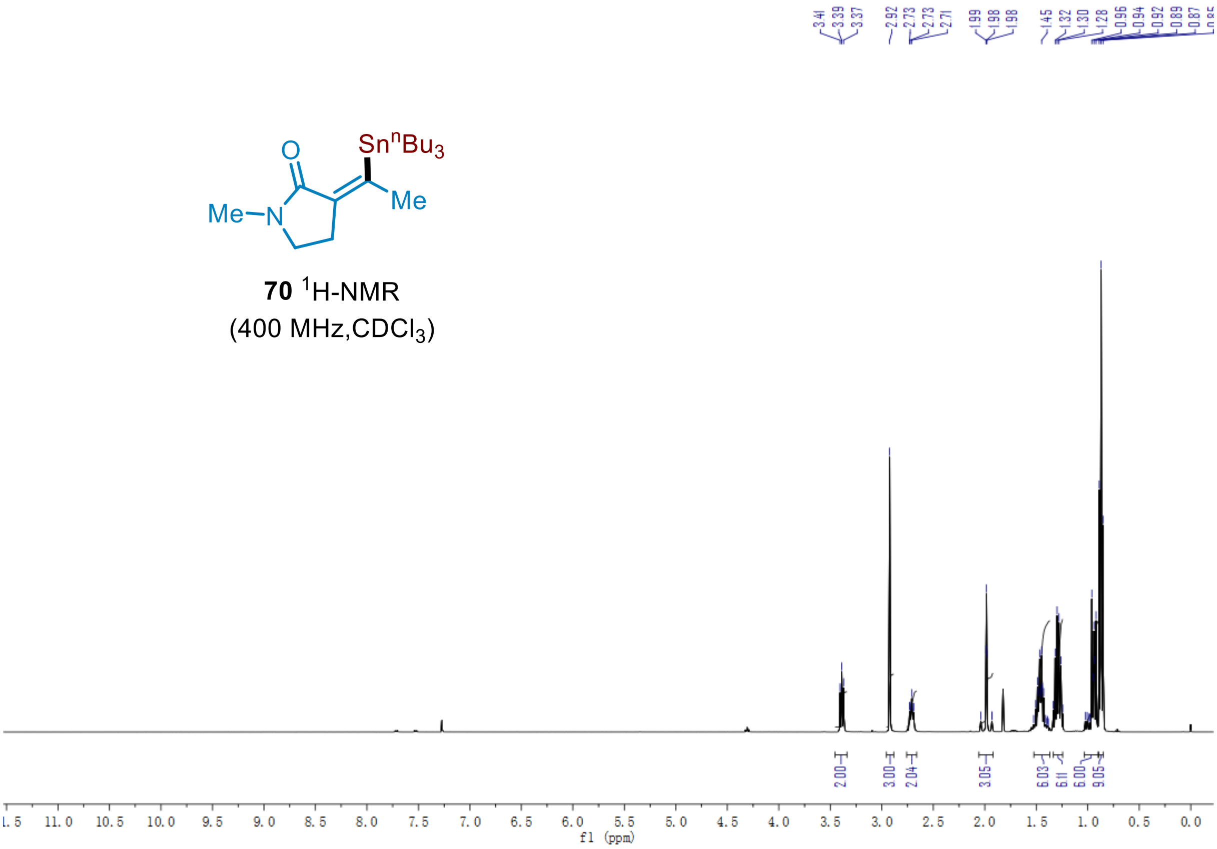

Supplementary Figure 241. **^{1}H-NMR** (400 MHz, $CDCl_3$, 298K) of **70**

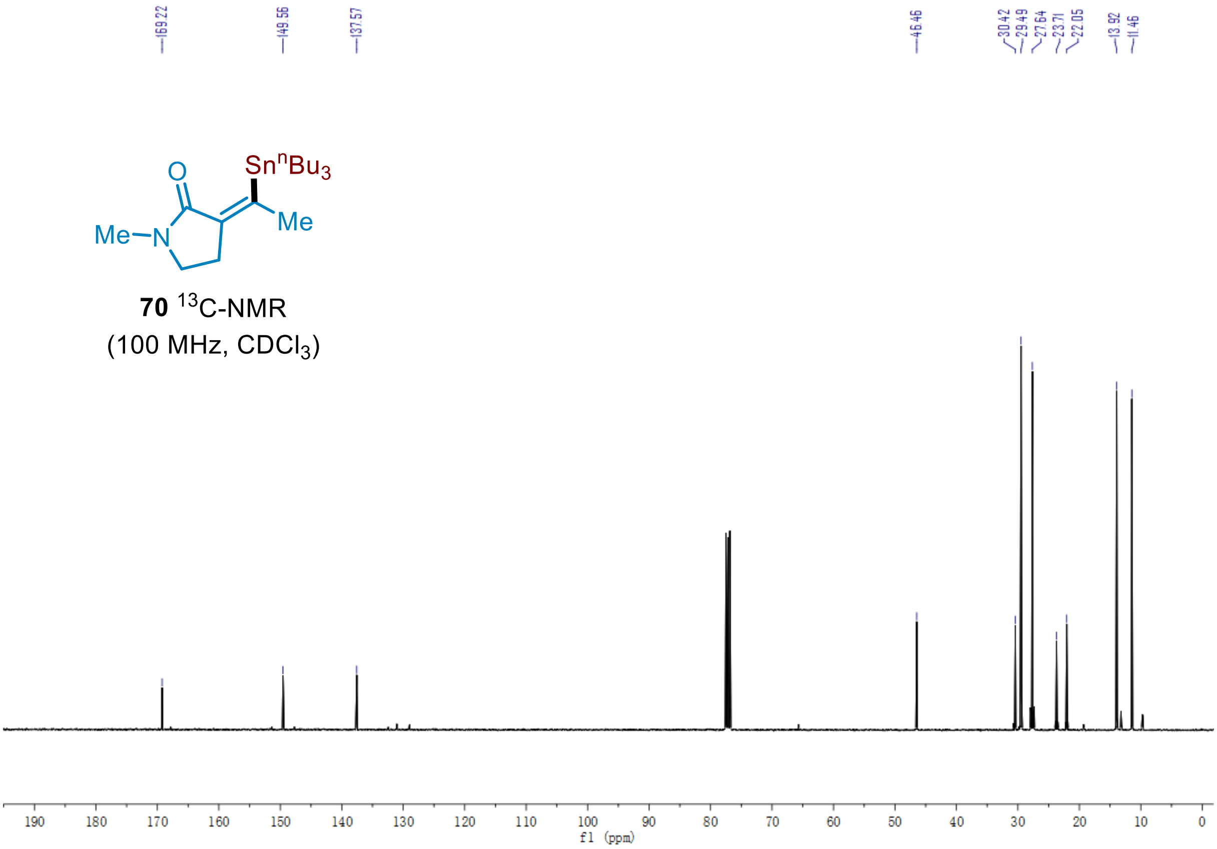

Supplementary Figure 242. **^{13}C-NMR** (100 MHz, $CDCl_3$, 298K) of **70**

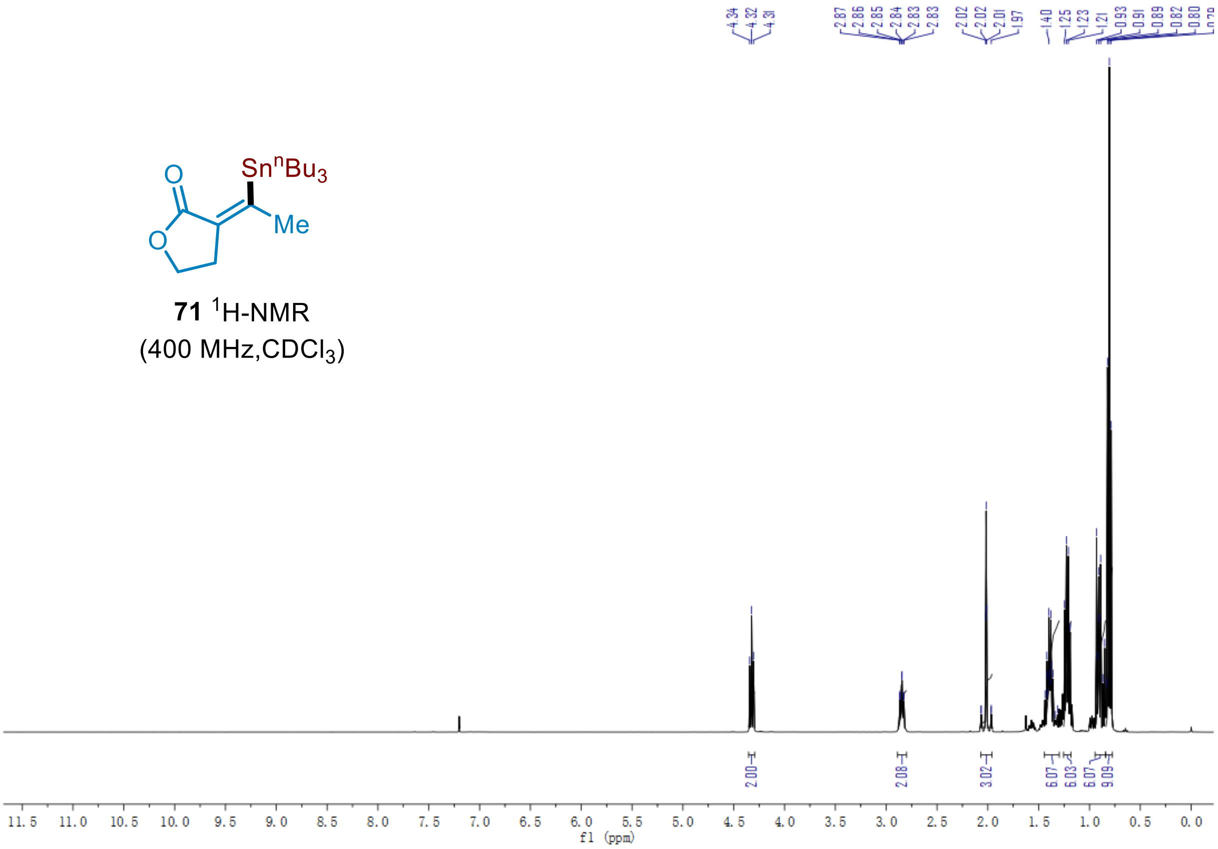

Supplementary Figure 243. **^{1}H-NMR** (400 MHz, $CDCl_3$, 298K) of **71**

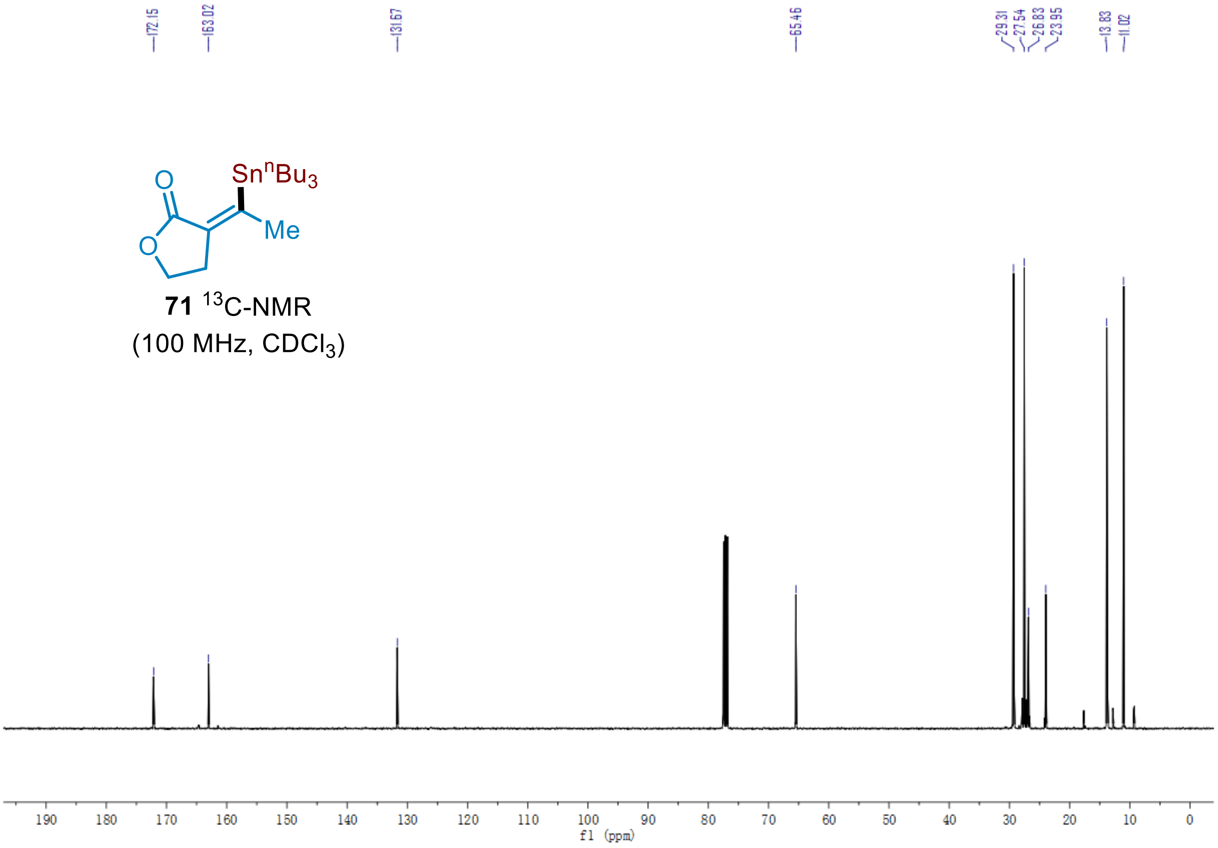

Supplementary Figure 244. **^{13}C-NMR** (100 MHz, $CDCl_3$, 298K) of **71**

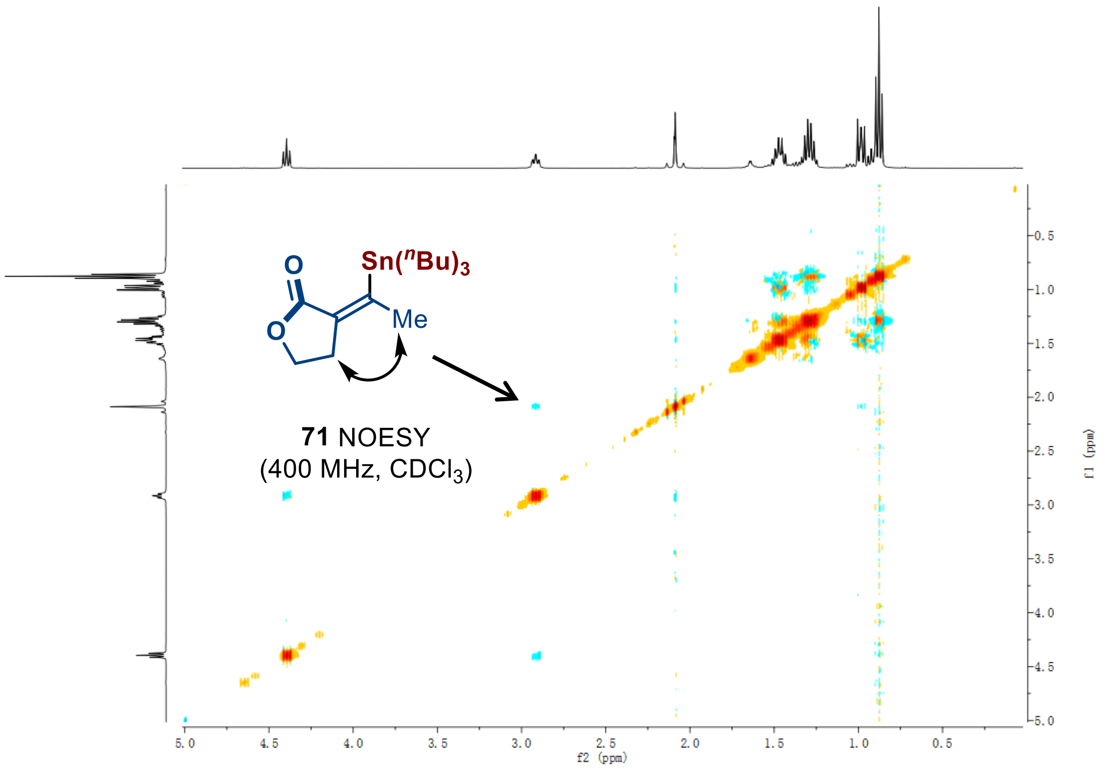

Supplementary Figure 245. **NOESY** of **71**

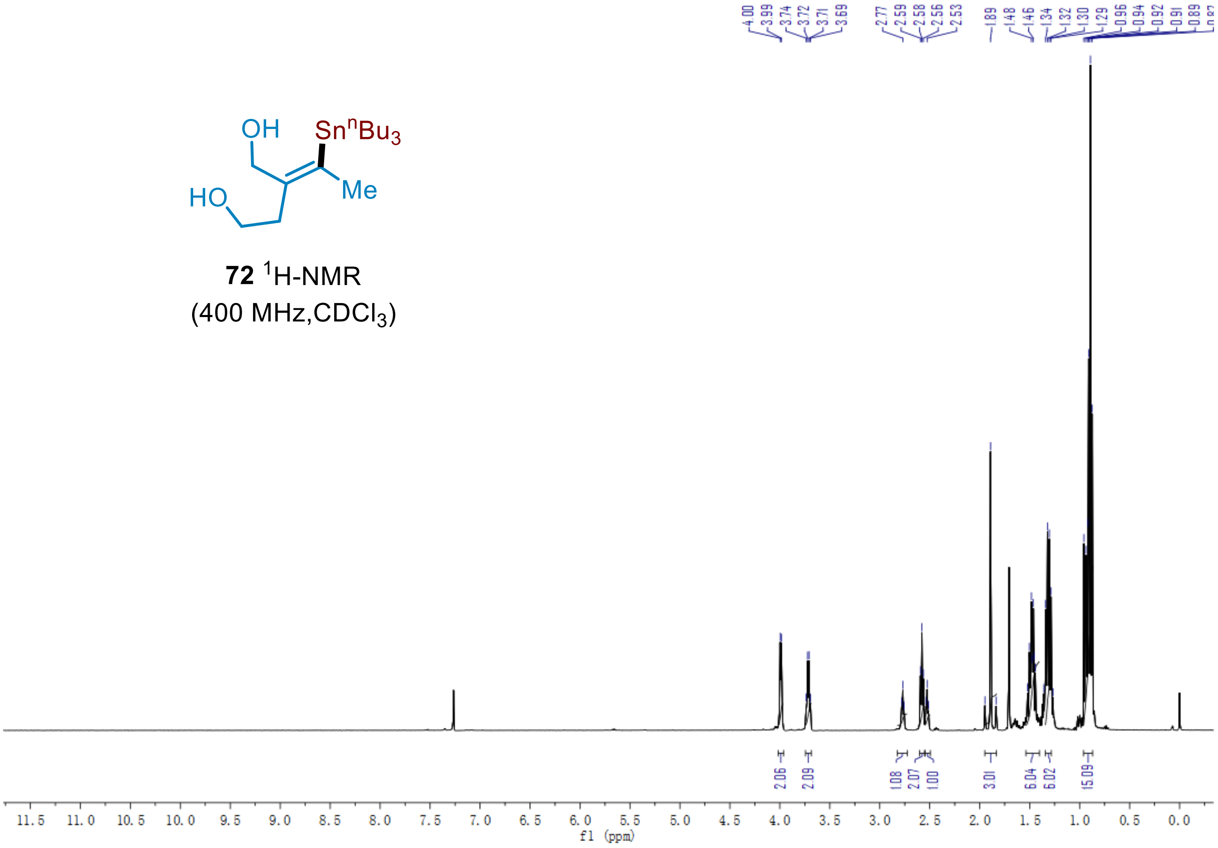

Supplementary Figure 246. **^{1}H-NMR** (400 MHz, $CDCl_3$, 298K) of **72**

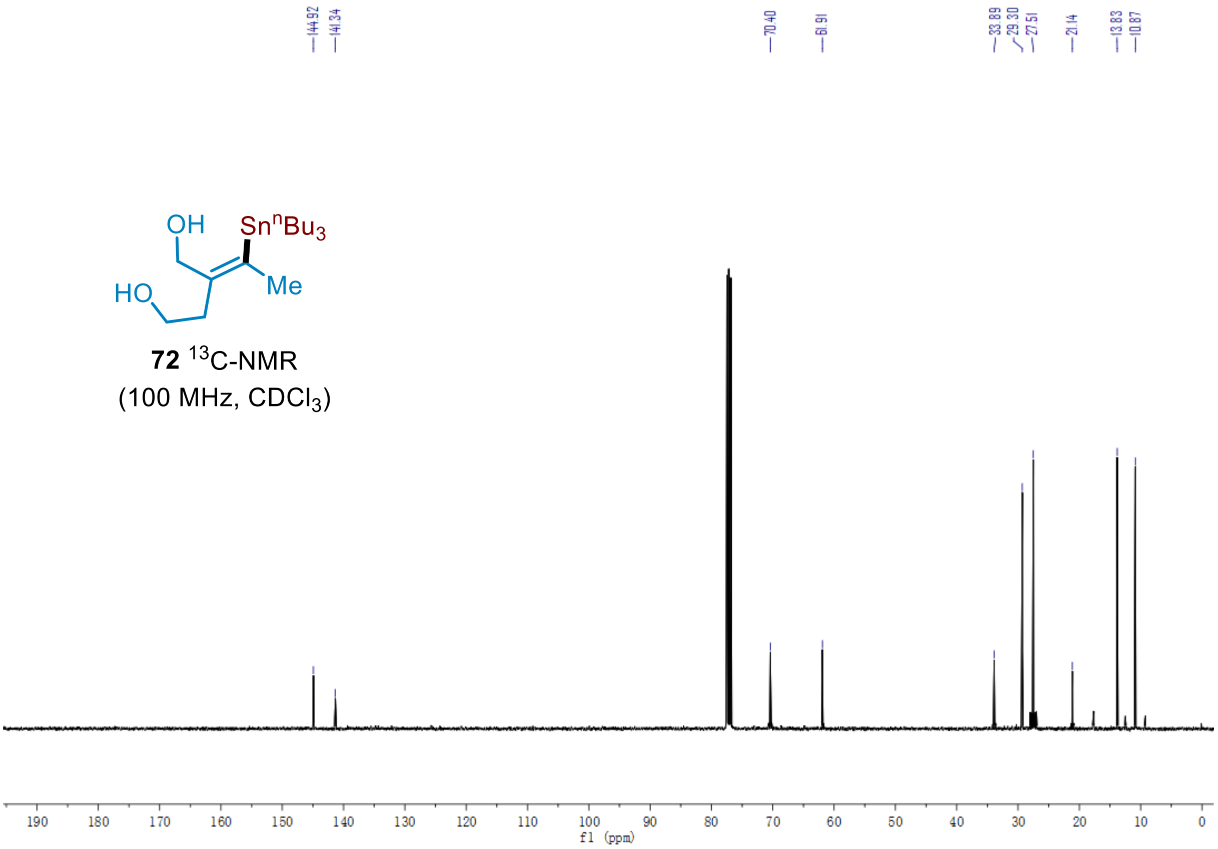

Supplementary Figure 247. **^{13}C-NMR** (100 MHz, CDCl$_3$, 298K) of **72**

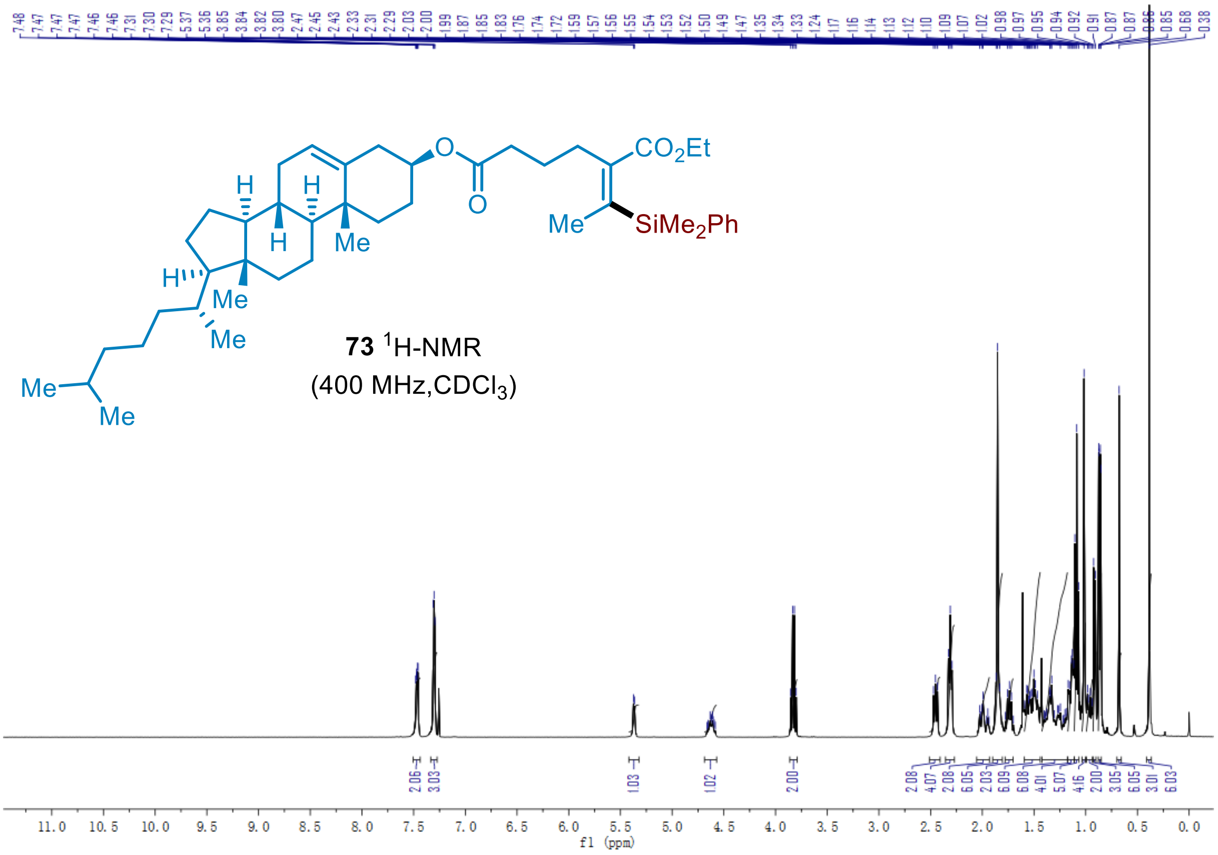

Supplementary Figure 248. **^{1}H-NMR** (400 MHz, CDCl$_3$, 298K) of **73**

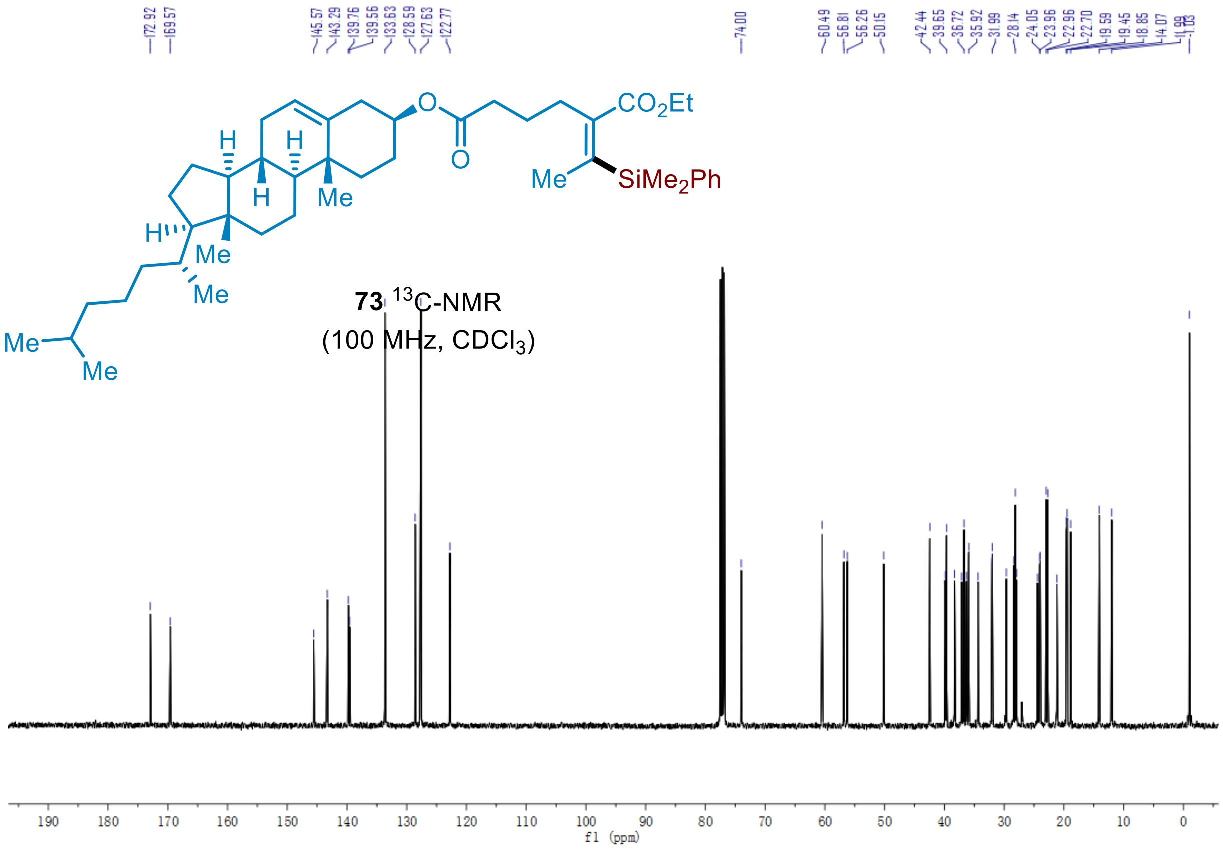

Supplementary Figure 249. ^{13}**C-NMR** (100 MHz, $CDCl_3$, 298K) of **73**

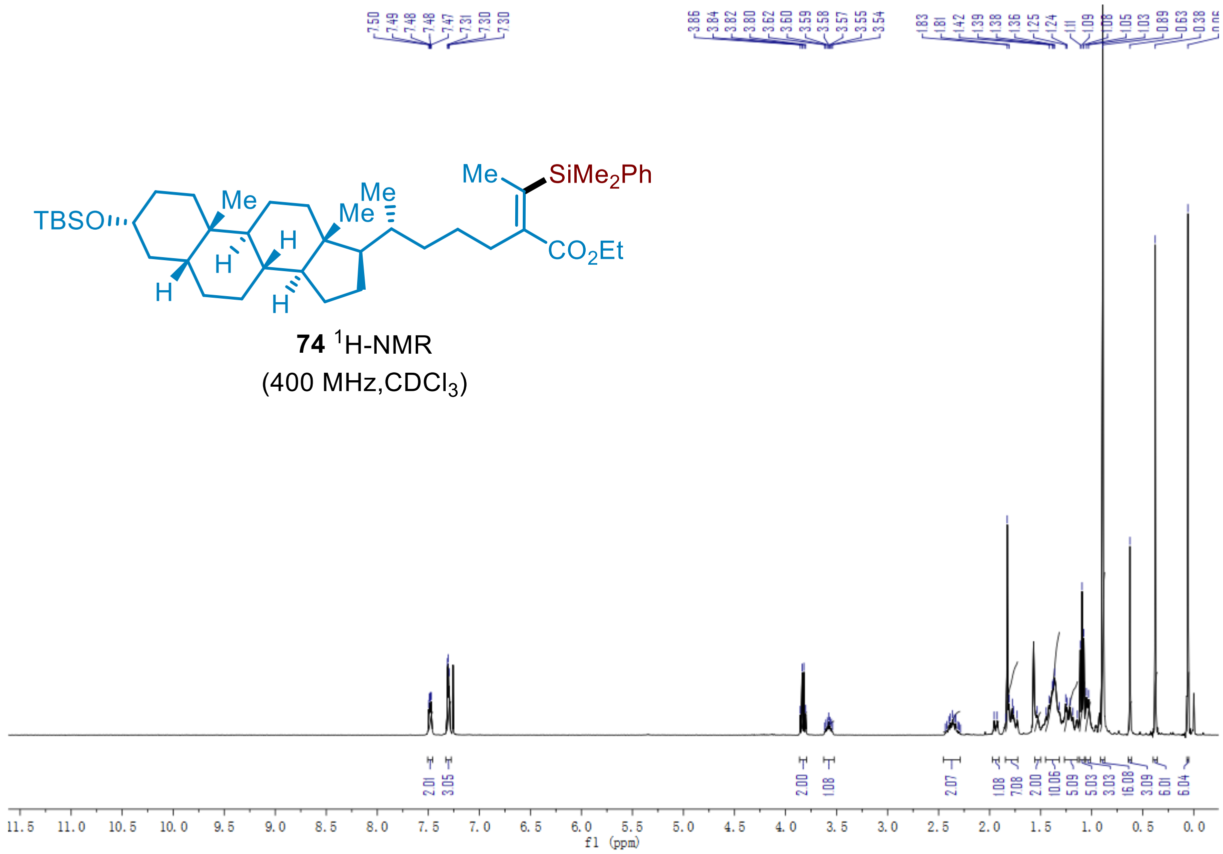

Supplementary Figure 250. ^{1}**H-NMR** (400 MHz, $CDCl_3$, 298K) of **74**

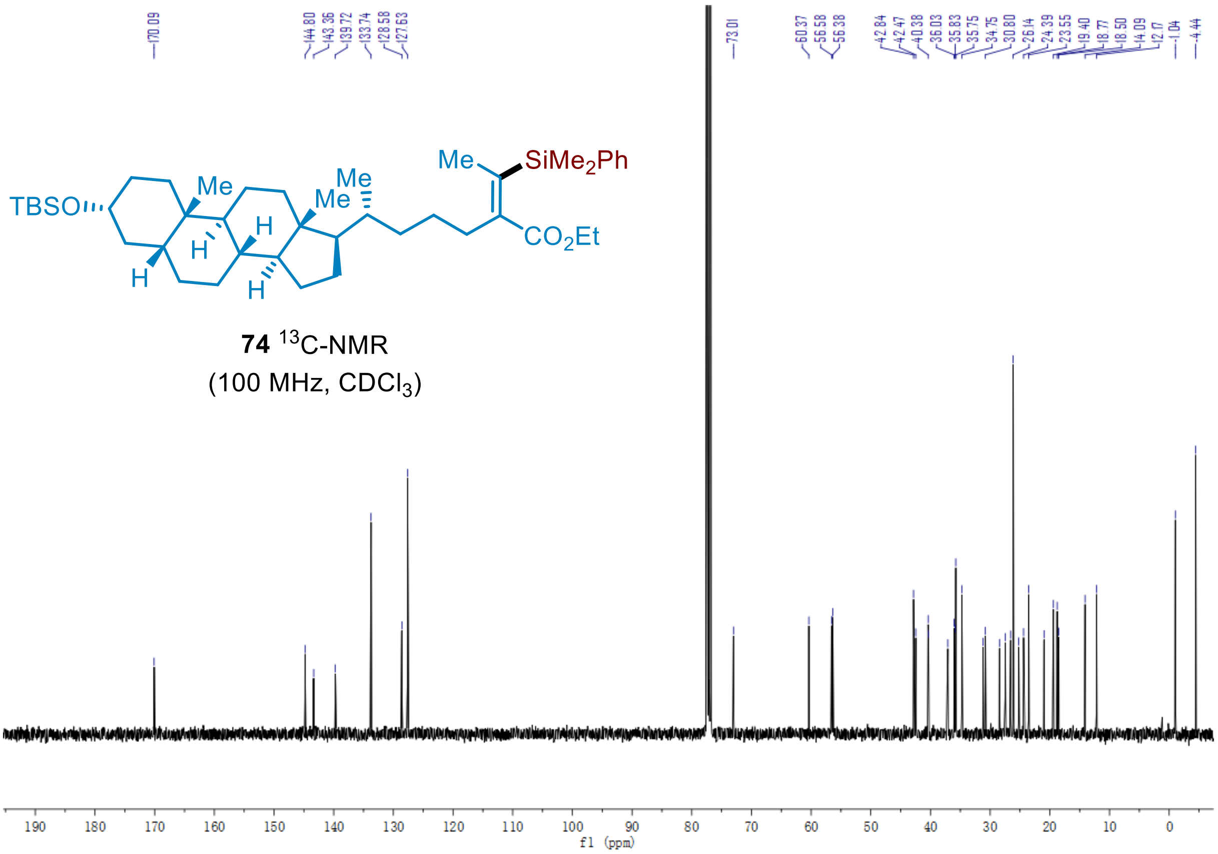

Supplementary Figure 251. **^{13}C-NMR** (100 MHz, $CDCl_3$, 298K) of **74**

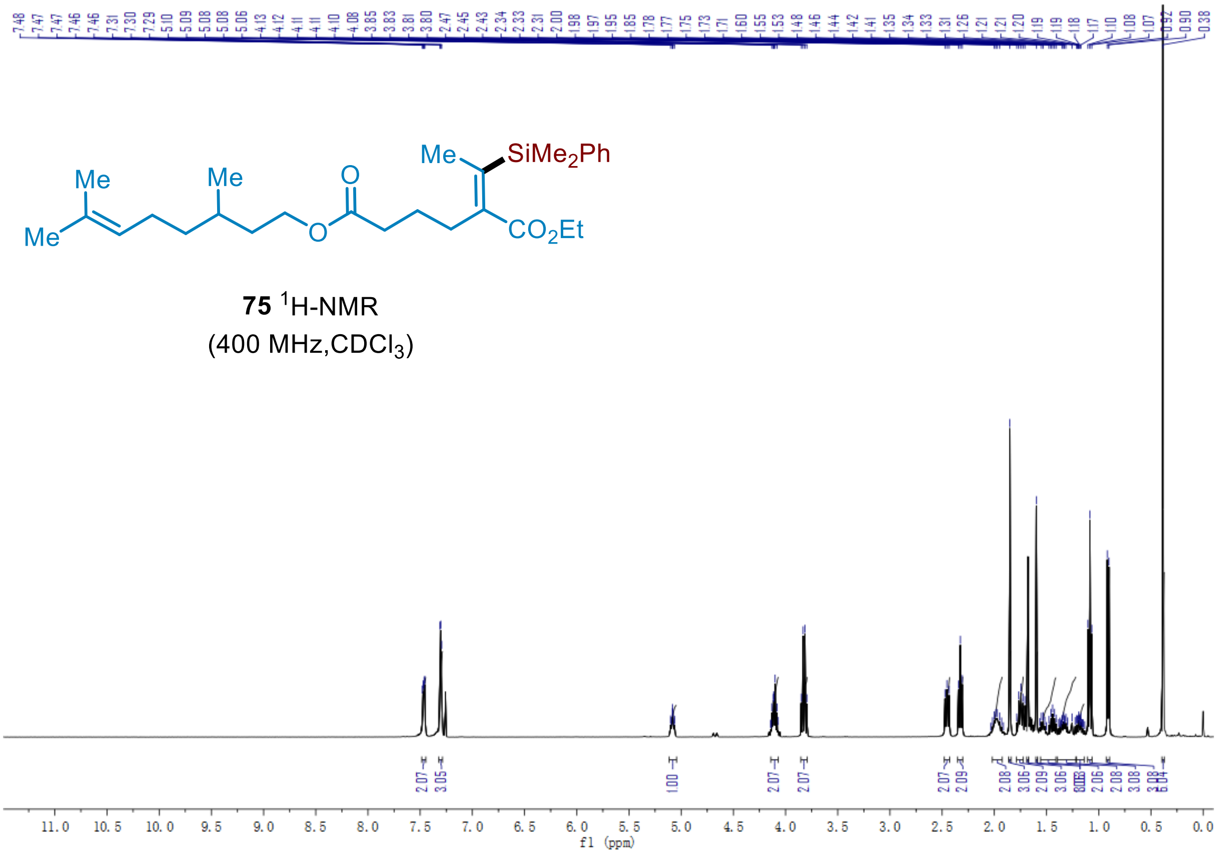

Supplementary Figure 252. **^{1}H-NMR** (400 MHz, $CDCl_3$, 298K) of **75**

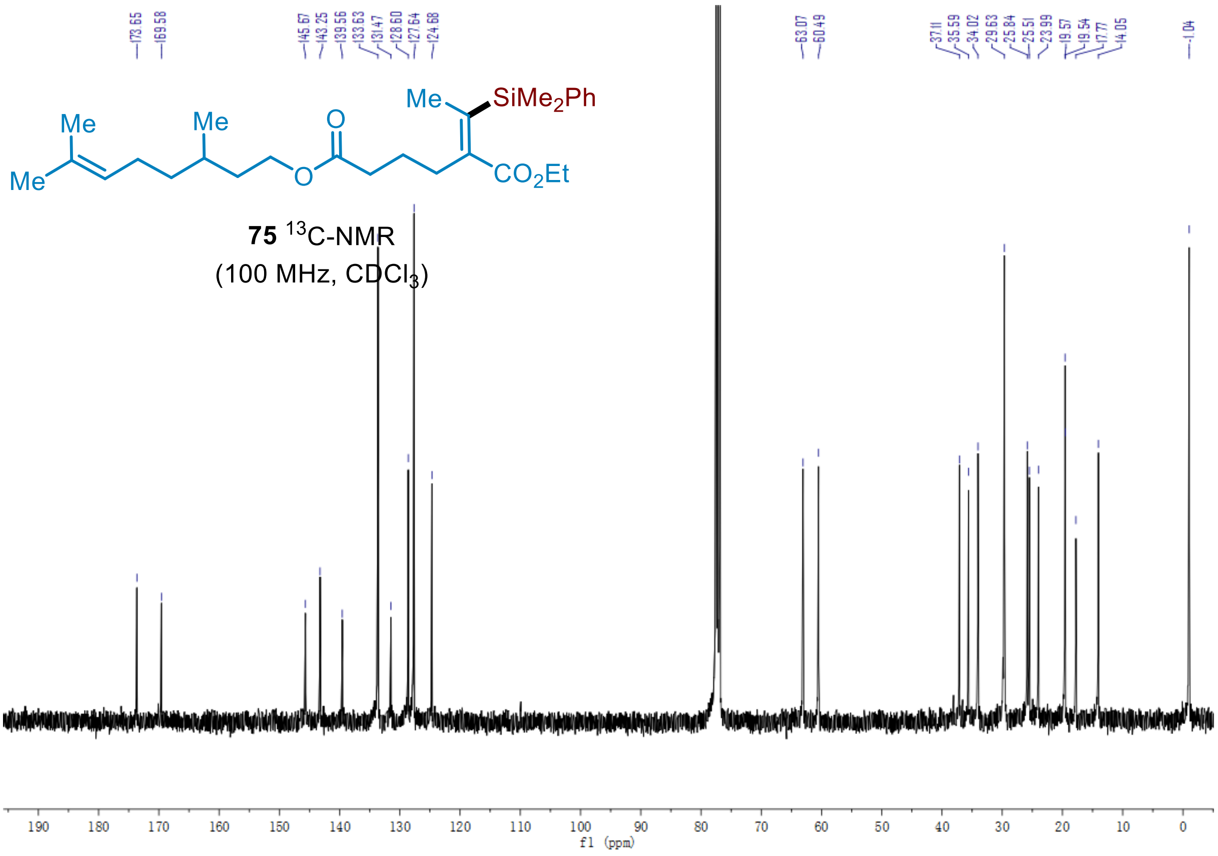

Supplementary Figure 253. **^{13}C-NMR** (100 MHz, $CDCl_3$, 298K) of **75**

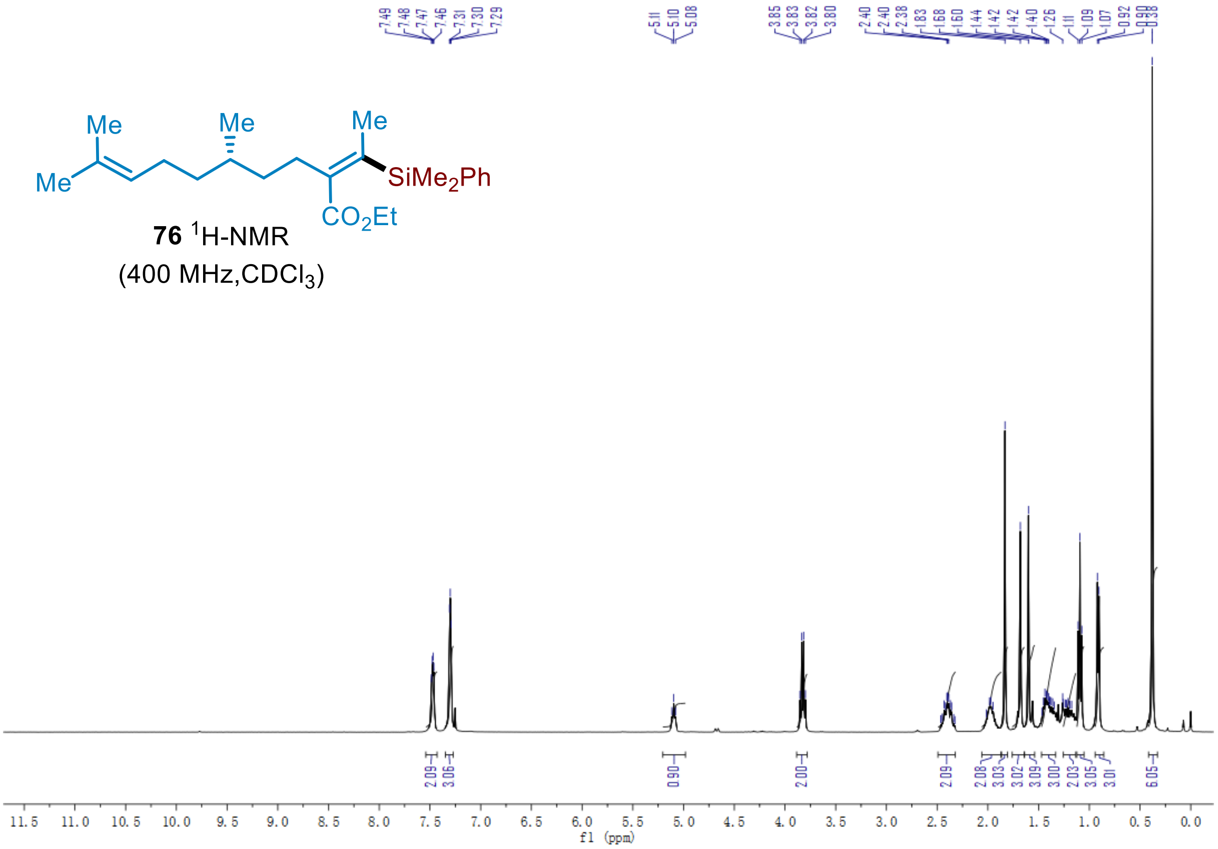

Supplementary Figure 254. **^{1}H-NMR** (400 MHz, $CDCl_3$, 298K) of **76**

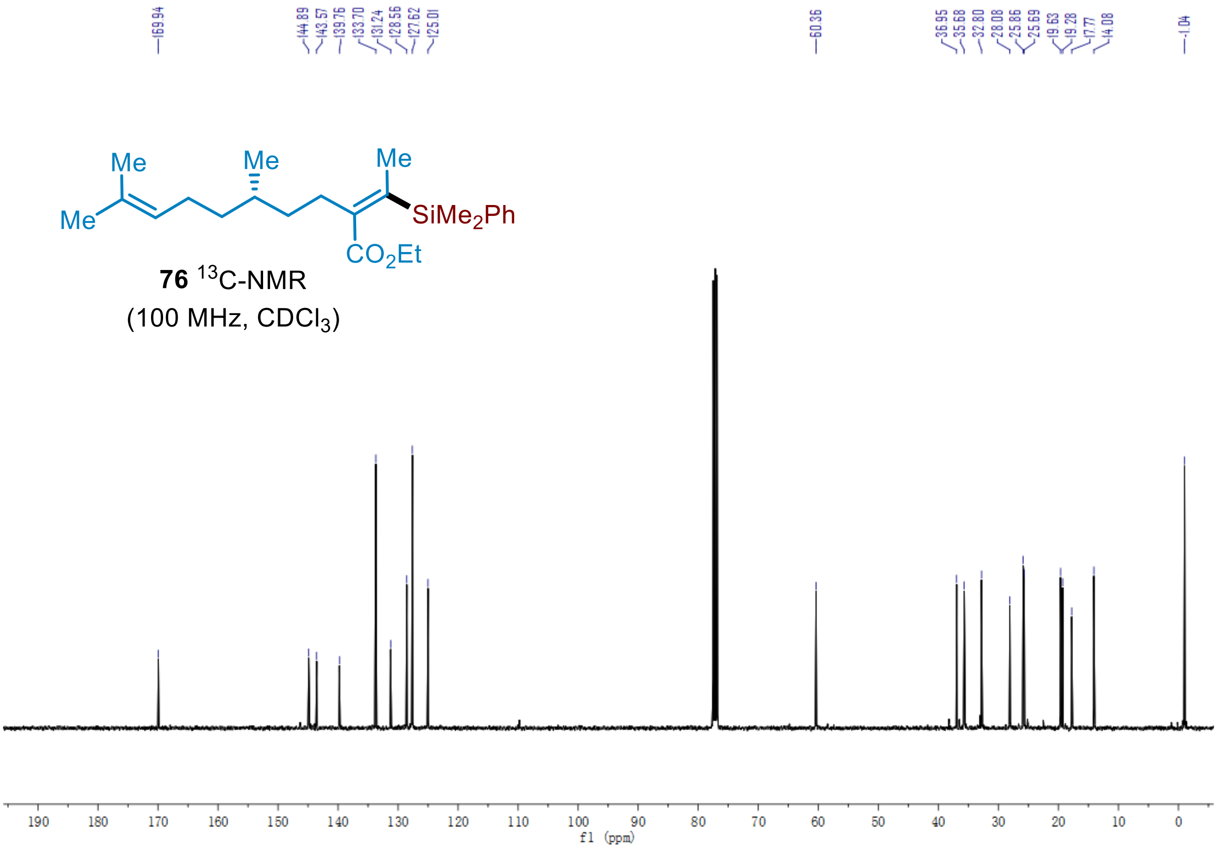

Supplementary Figure 255. **^{13}C-NMR** (100 MHz, $CDCl_3$, 298K) of **76**

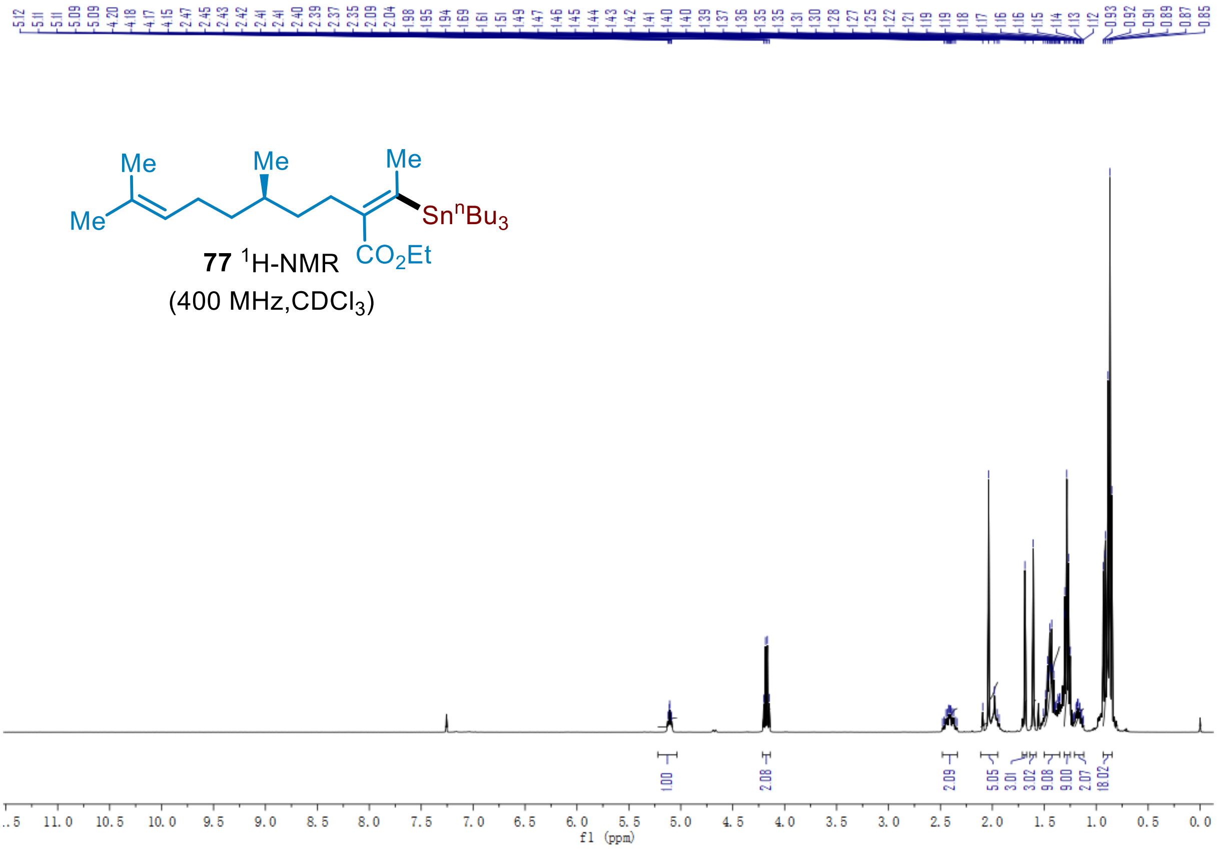

Supplementary Figure 256. **^{1}H-NMR** (400 MHz, $CDCl_3$, 298K) of **77**

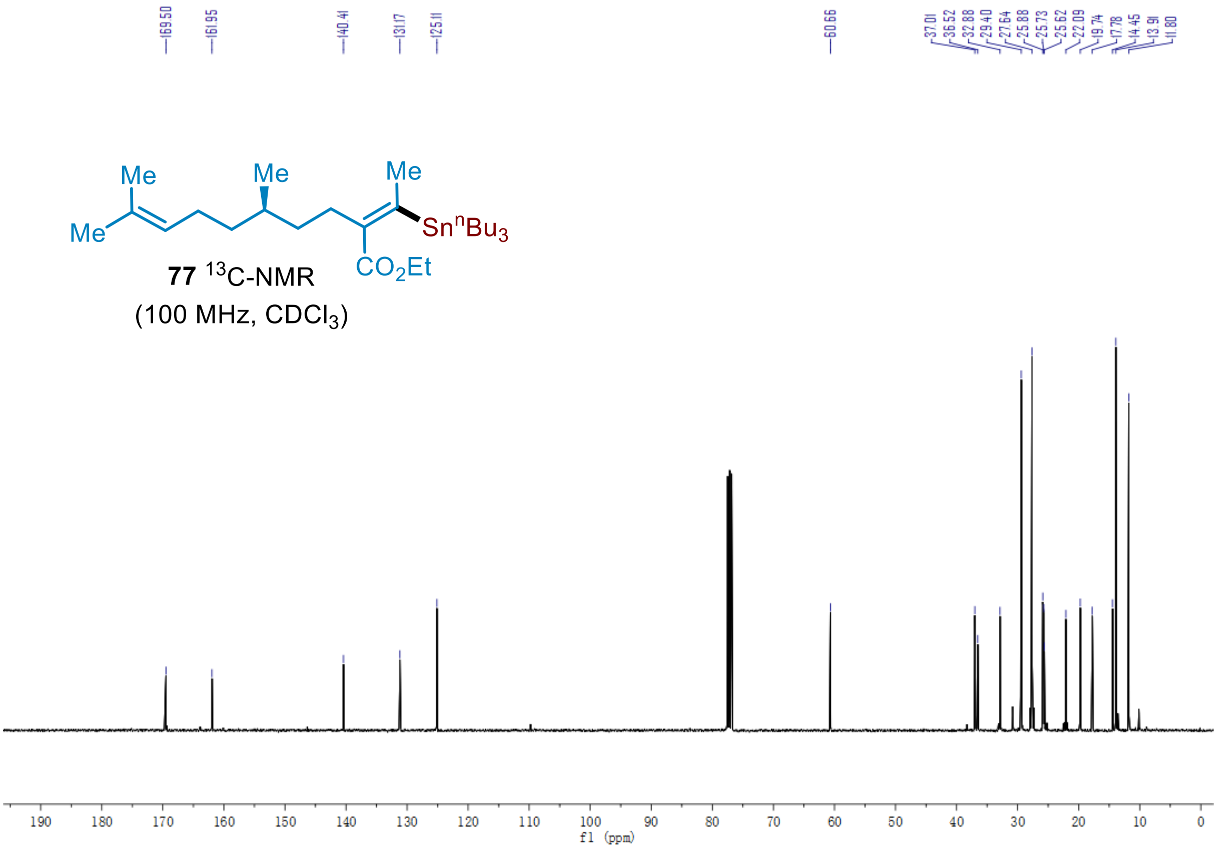

Supplementary Figure 257. **^{13}C-NMR** (100 MHz, CDCl$_3$, 298K) of **77**

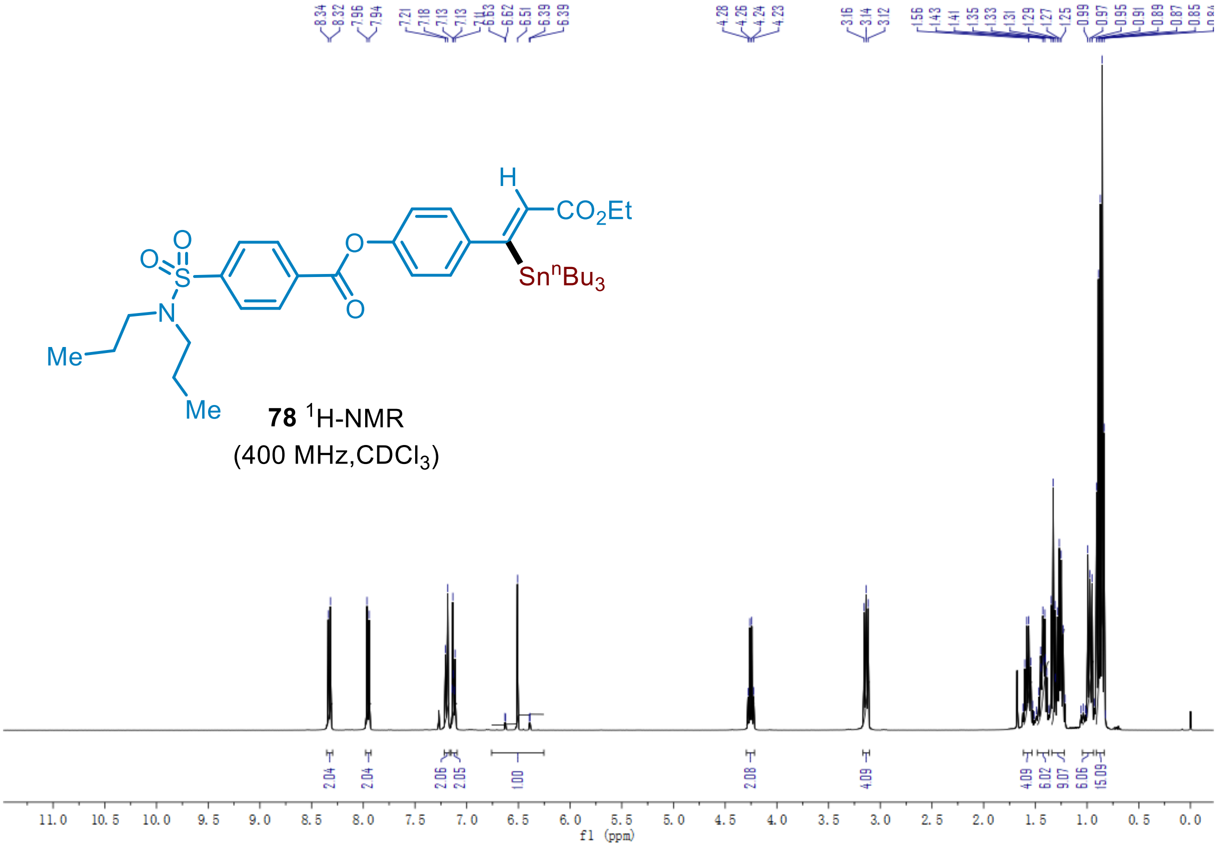

Supplementary Figure 258. **^{1}H-NMR** (400 MHz, CDCl$_3$, 298K) of **78**

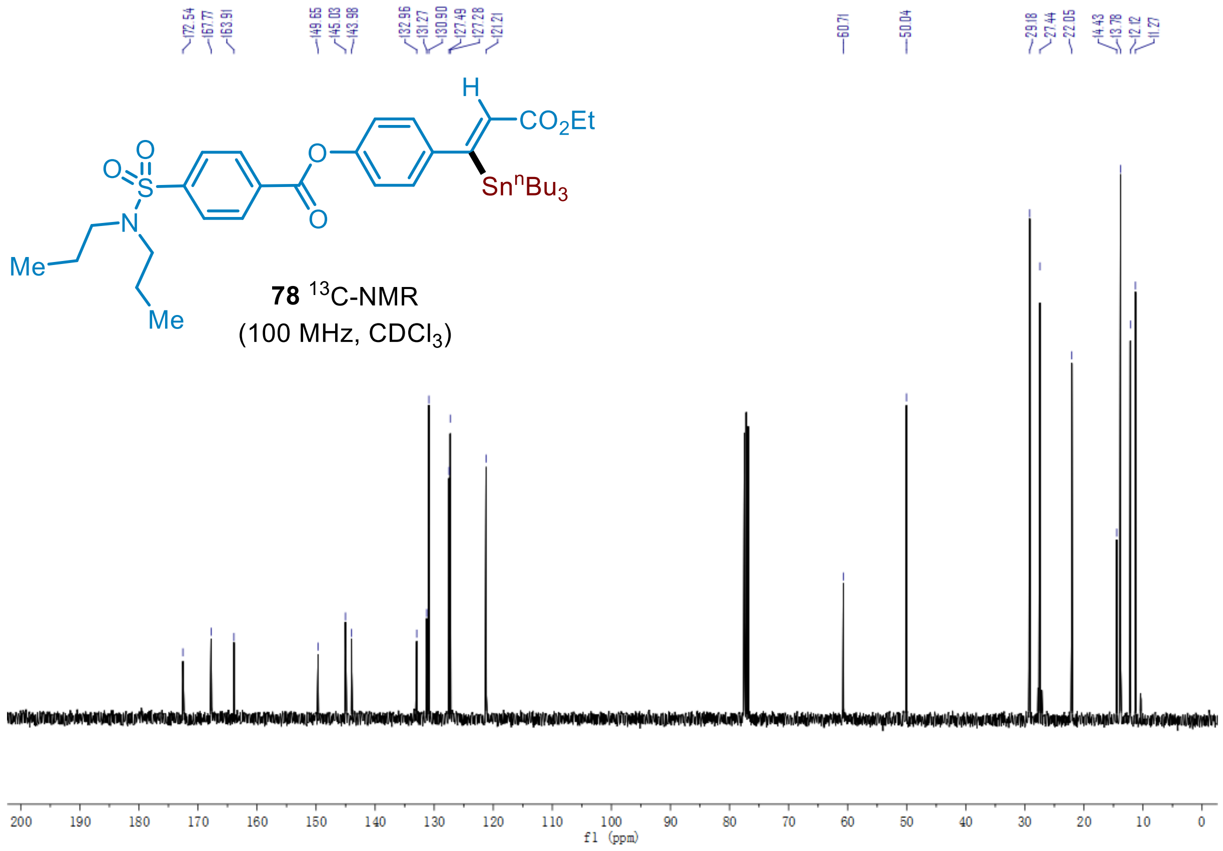

Supplementary Figure 259. **^{13}C-NMR** (100 MHz, $CDCl_3$, 298K) of **78**

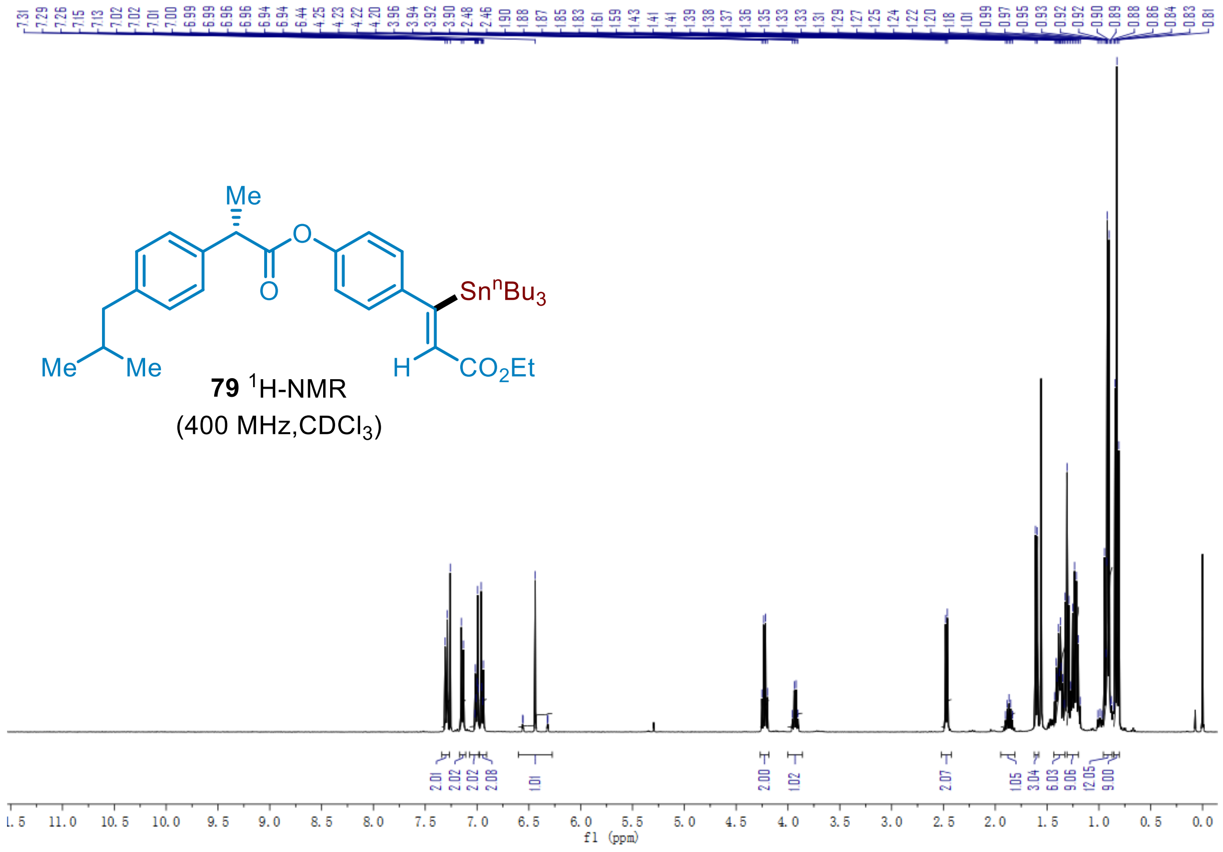

Supplementary Figure 260. **^{1}H-NMR** (400 MHz, $CDCl_3$, 298K) of **79**

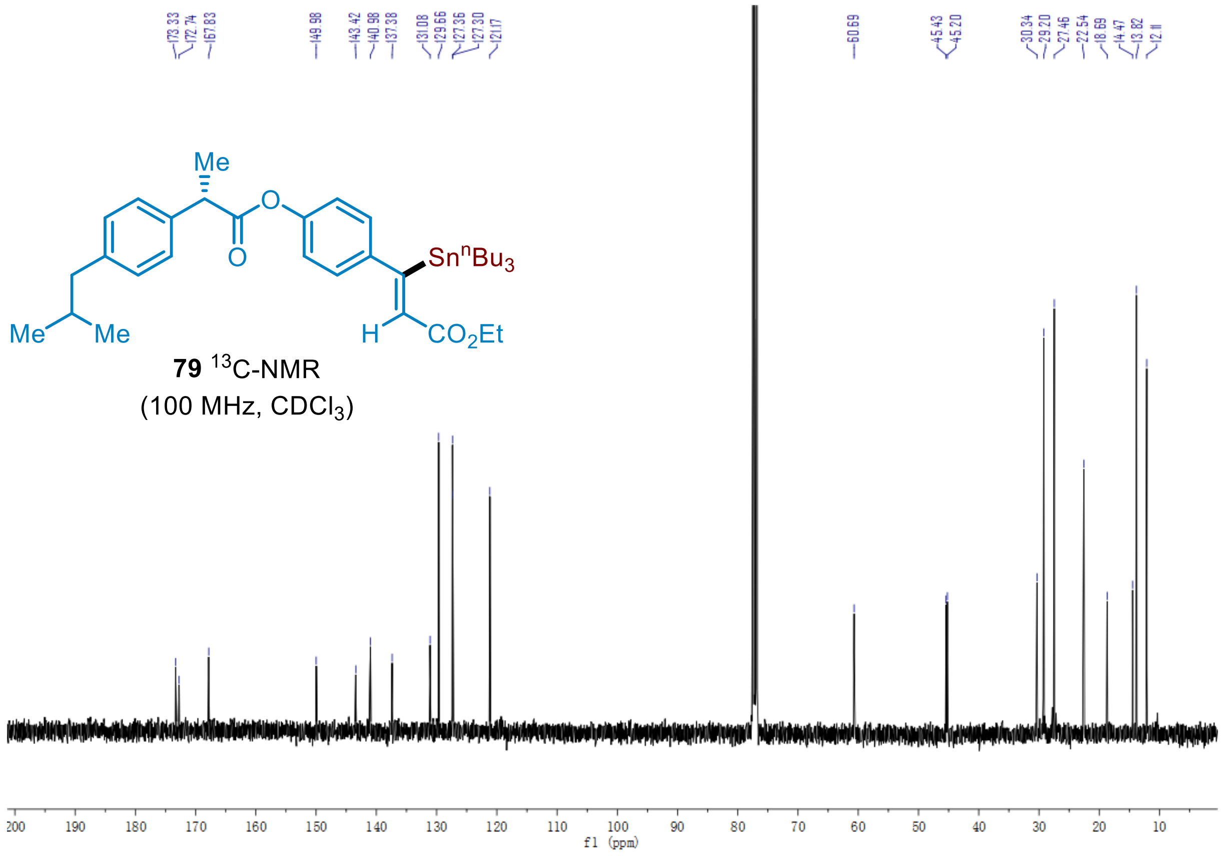

Supplementary Figure 261. **^{13}C-NMR** (100 MHz, CDCl$_3$, 298K) of **79**

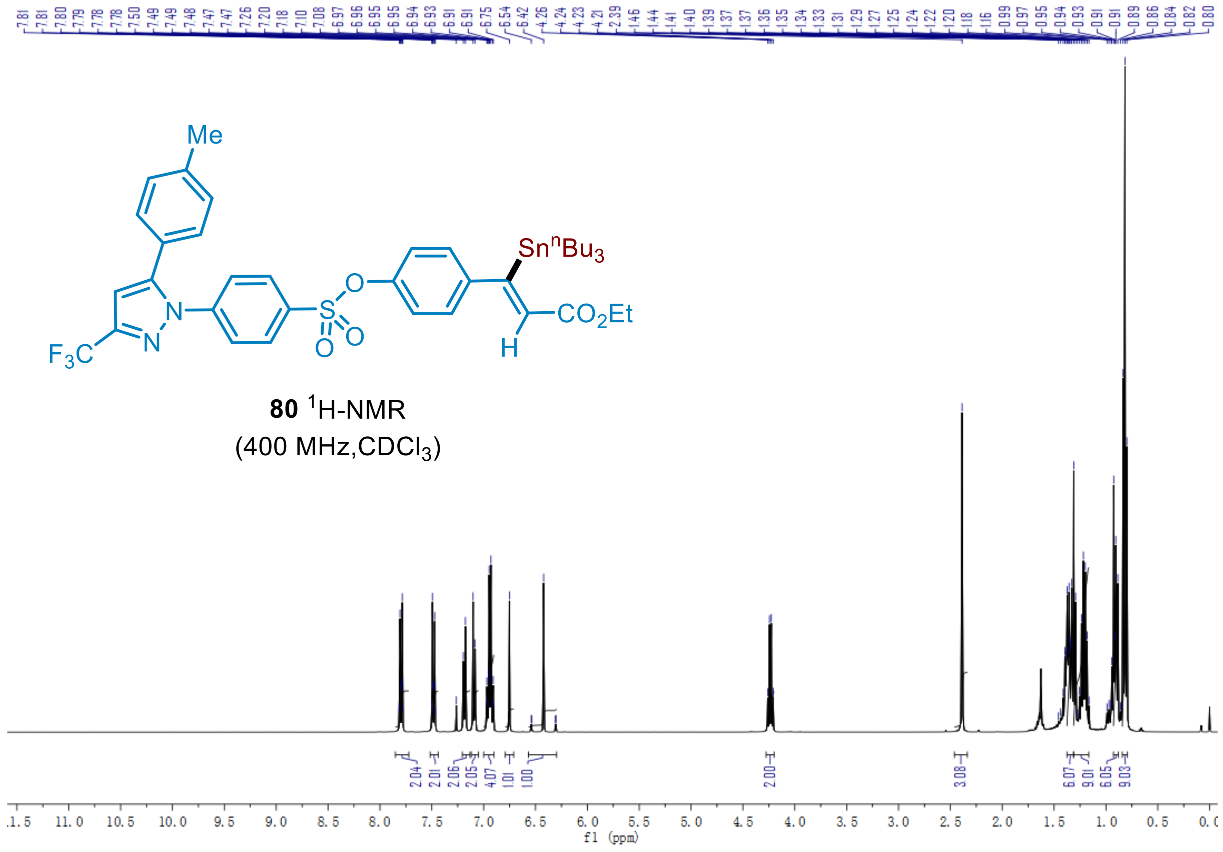

Supplementary Figure 262. **^{1}H-NMR** (400 MHz, CDCl$_3$, 298K) of **80**

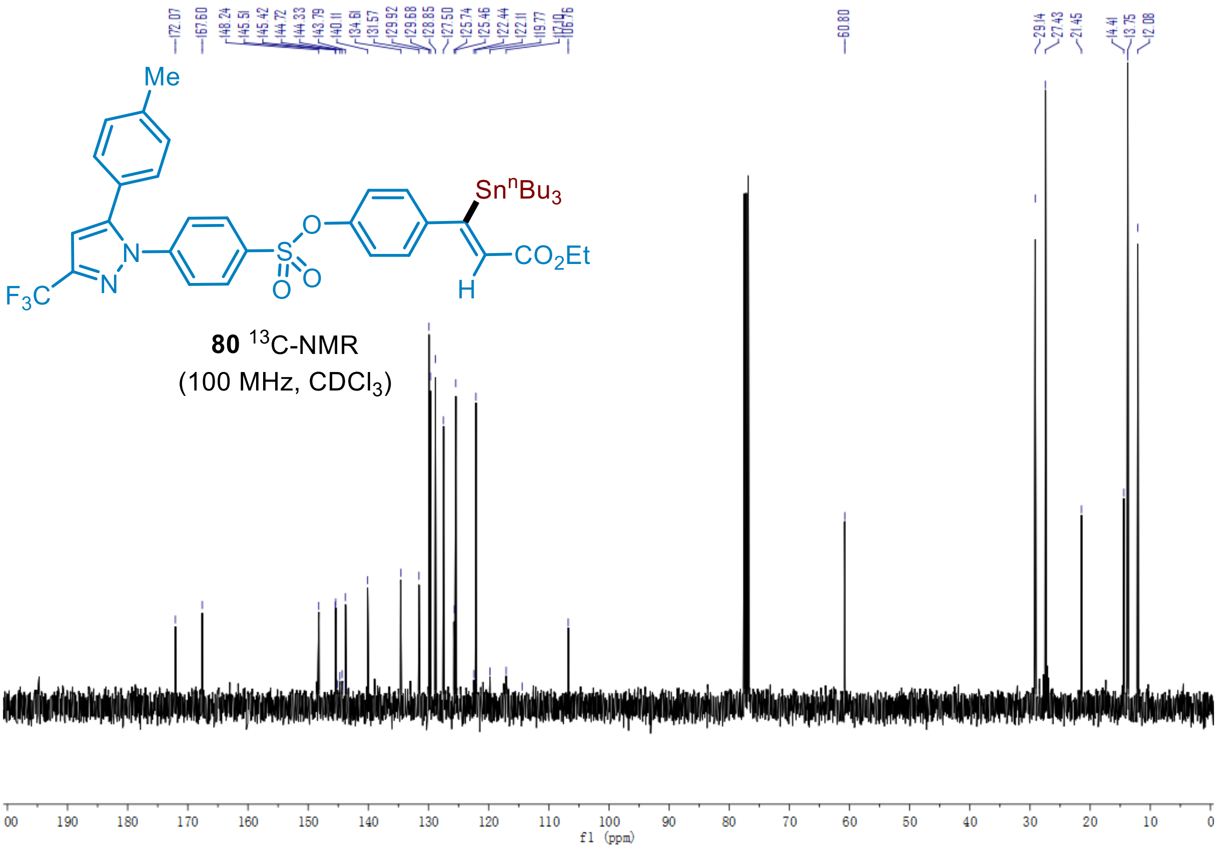

Supplementary Figure 263. **^{13}C-NMR** (100 MHz, $CDCl_3$, 298K) of **80**

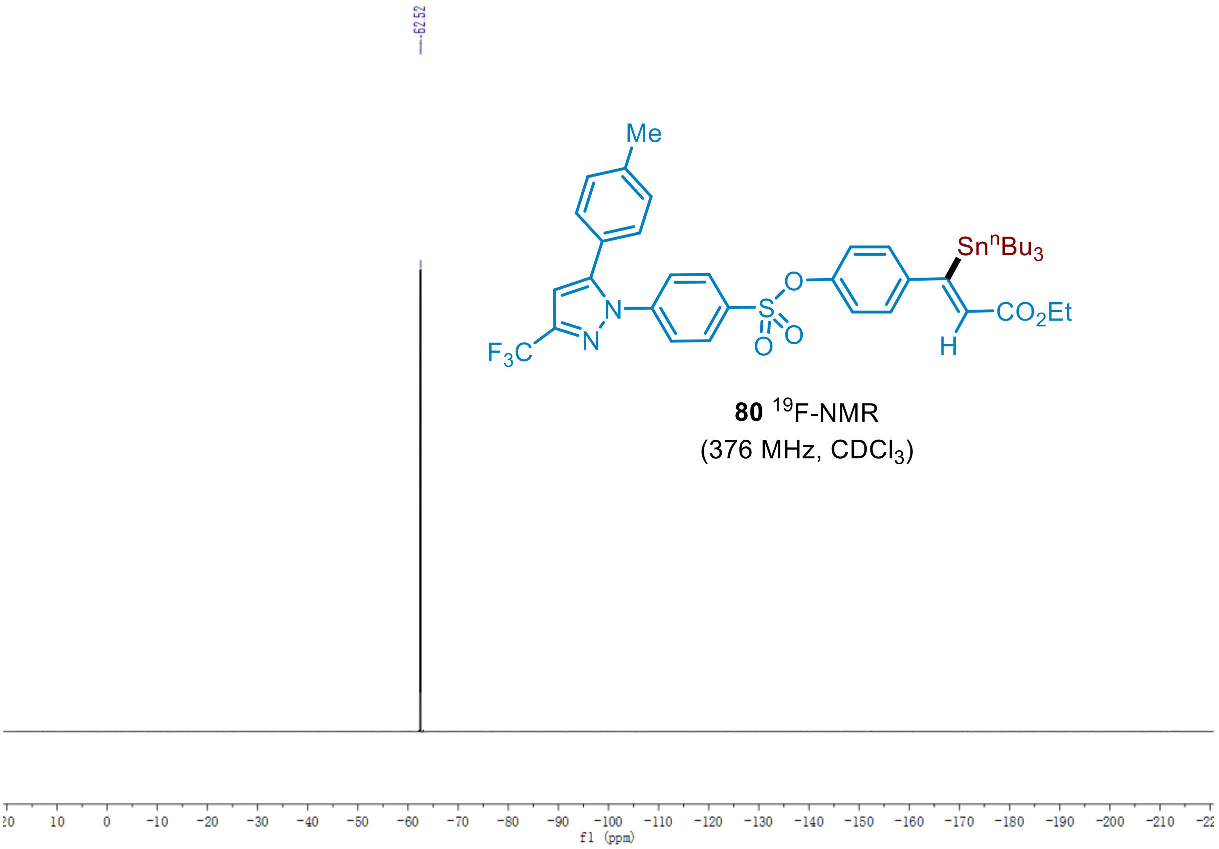

Supplementary Figure 264. **^{19}F-NMR** (376 MHz, $CDCl_3$, 298K) of **80**

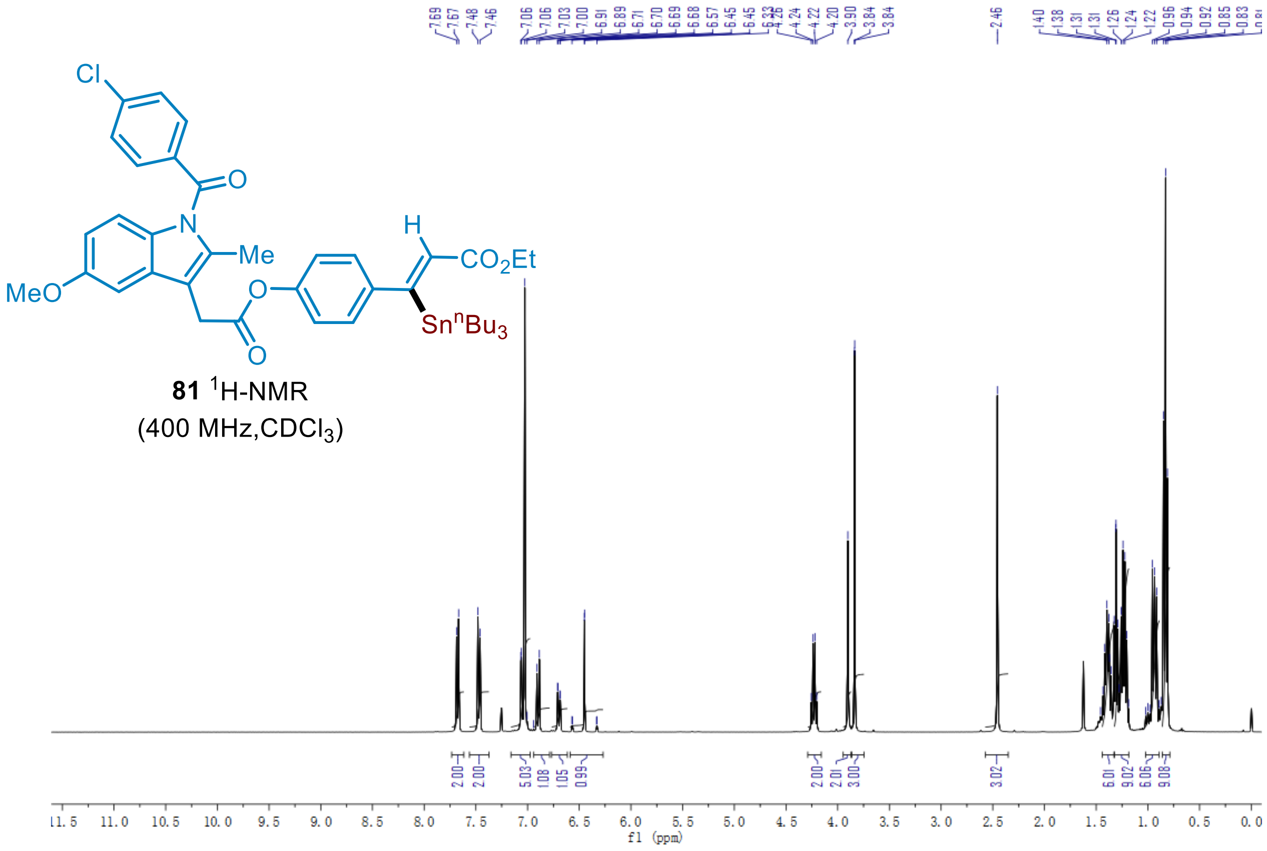

Supplementary Figure 265. **^{1}H-NMR** (400 MHz, $CDCl_3$, 298K) of **81**

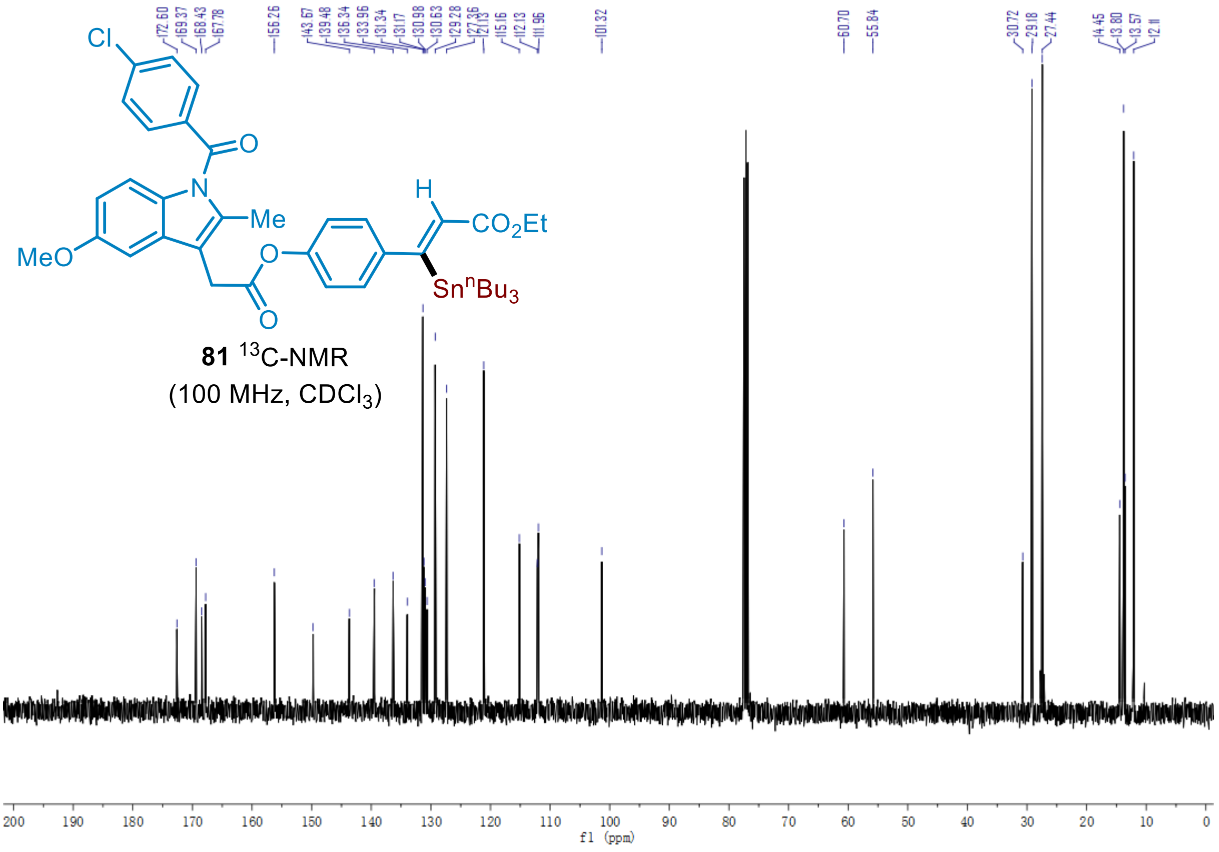

Supplementary Figure 266. **^{13}C-NMR** (100 MHz, $CDCl_3$, 298K) of **81**

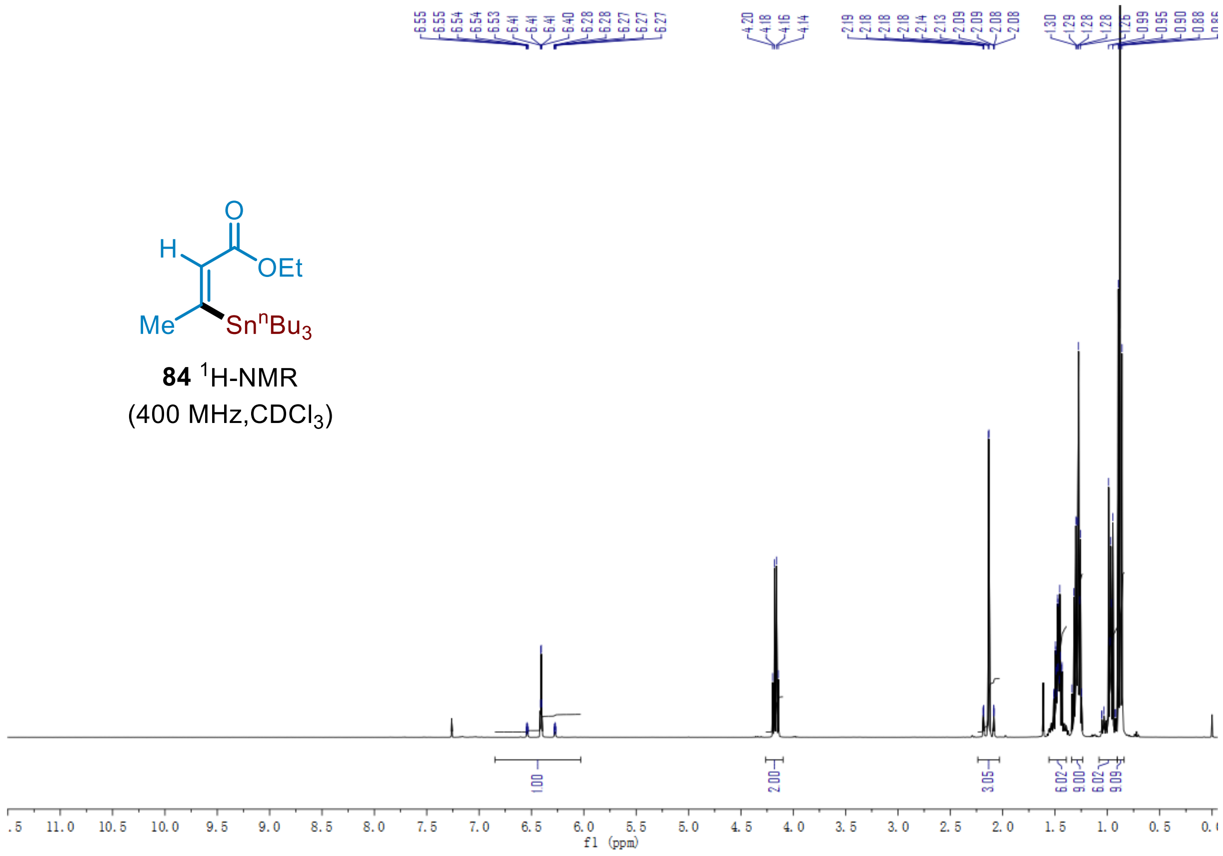

Supplementary Figure 267. **^{1}H-NMR** (400 MHz, $CDCl_3$, 298K) of **84**

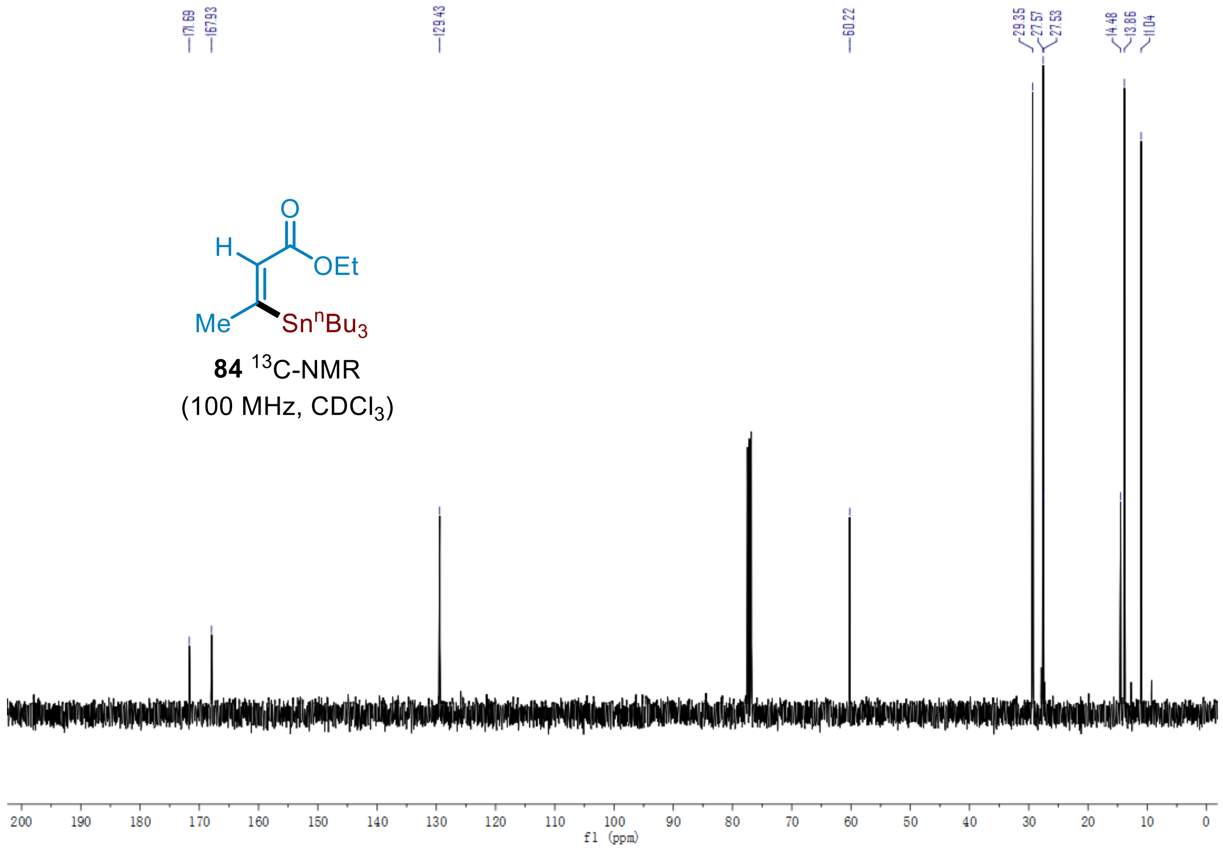

Supplementary Figure 268. **^{13}C-NMR** (100 MHz, $CDCl_3$, 298K) of **84**

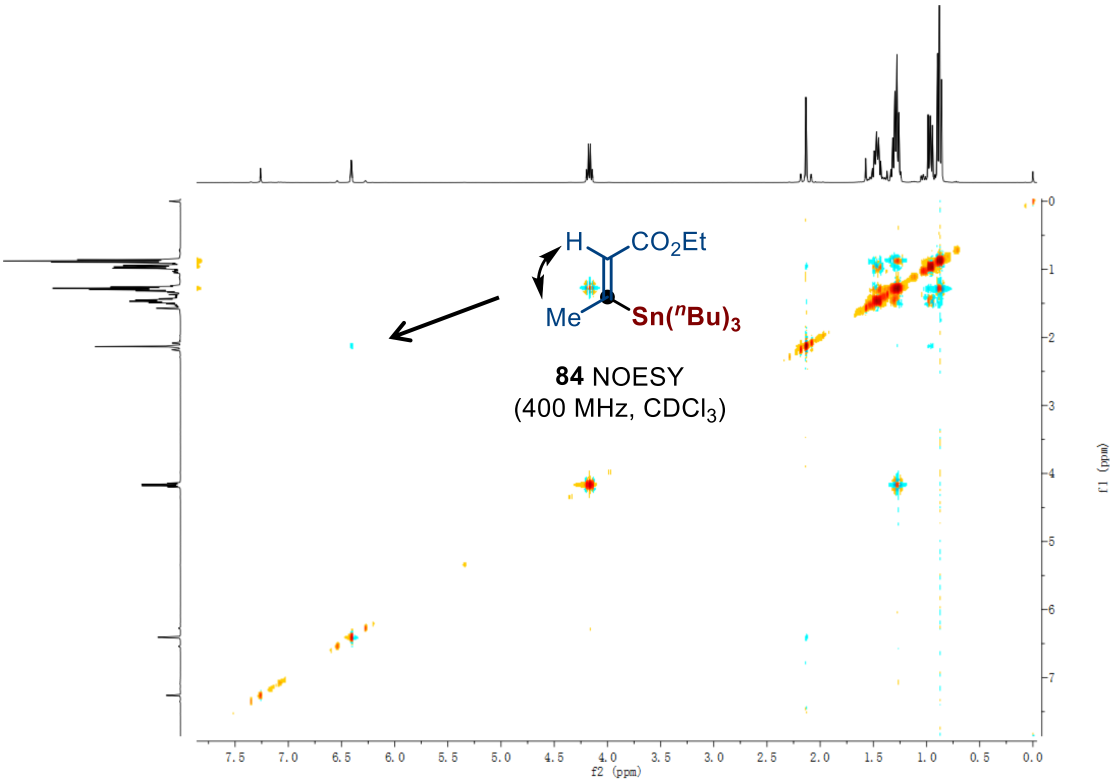

Supplementary Figure 269. **NOESY** of **84**

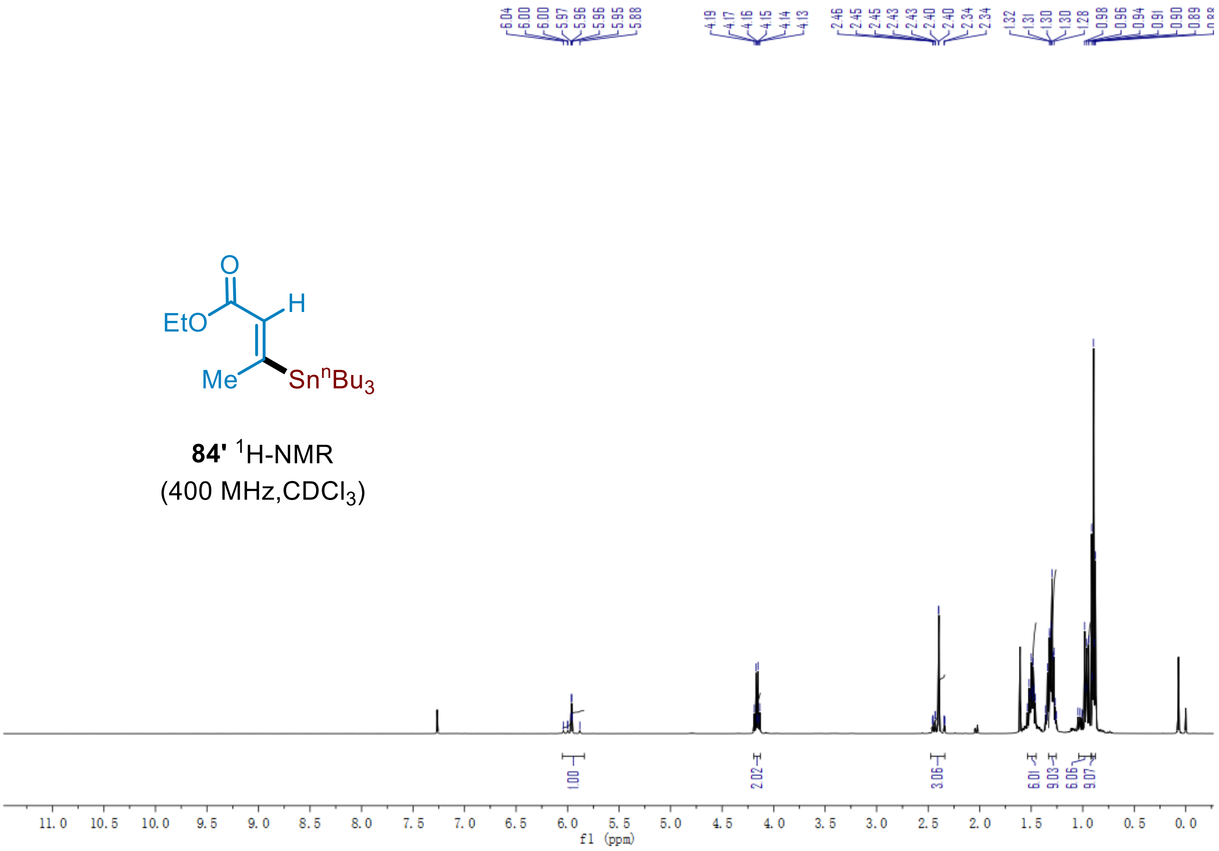

Supplementary Figure 270. **^{1}H-NMR** (400 MHz, $CDCl_3$, 298K) of **84'**

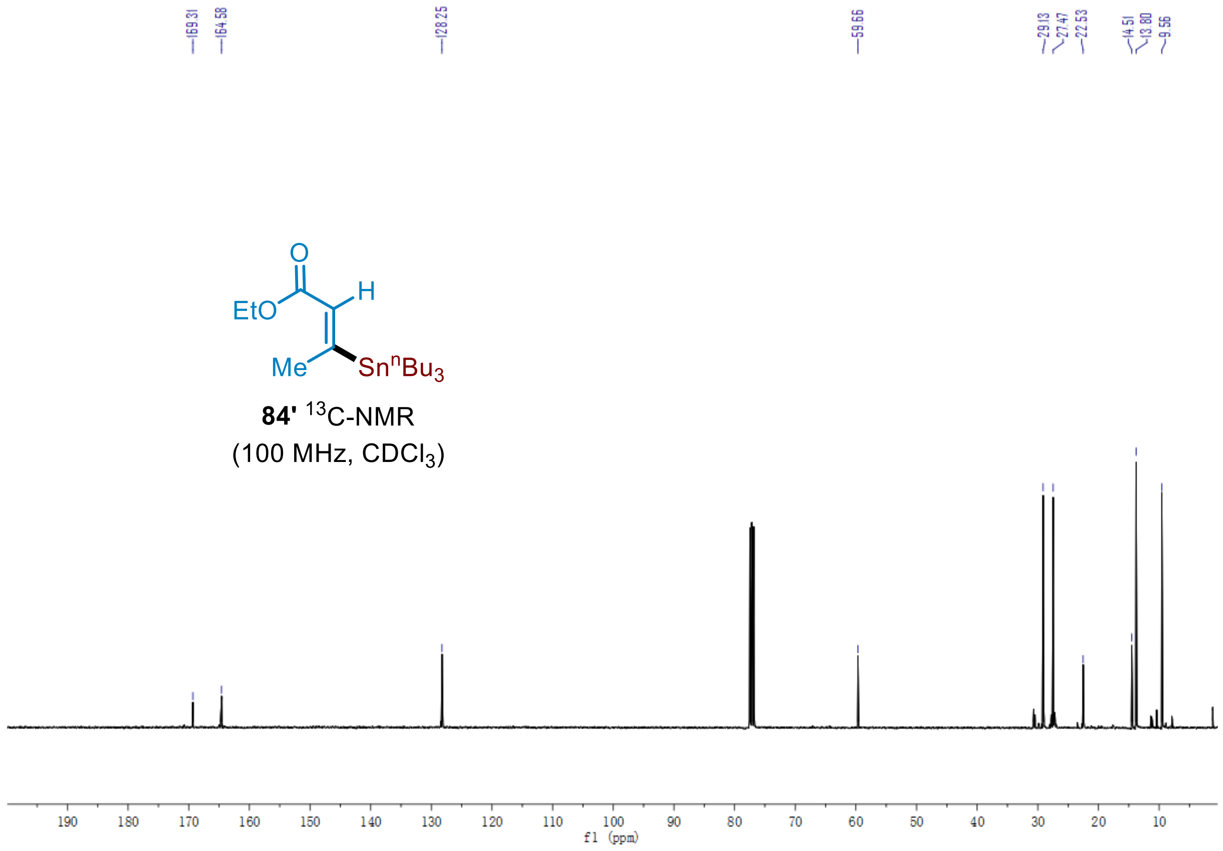

Supplementary Figure 271. **^{13}C-NMR** (100 MHz, $CDCl_3$, 298K) of **84'**

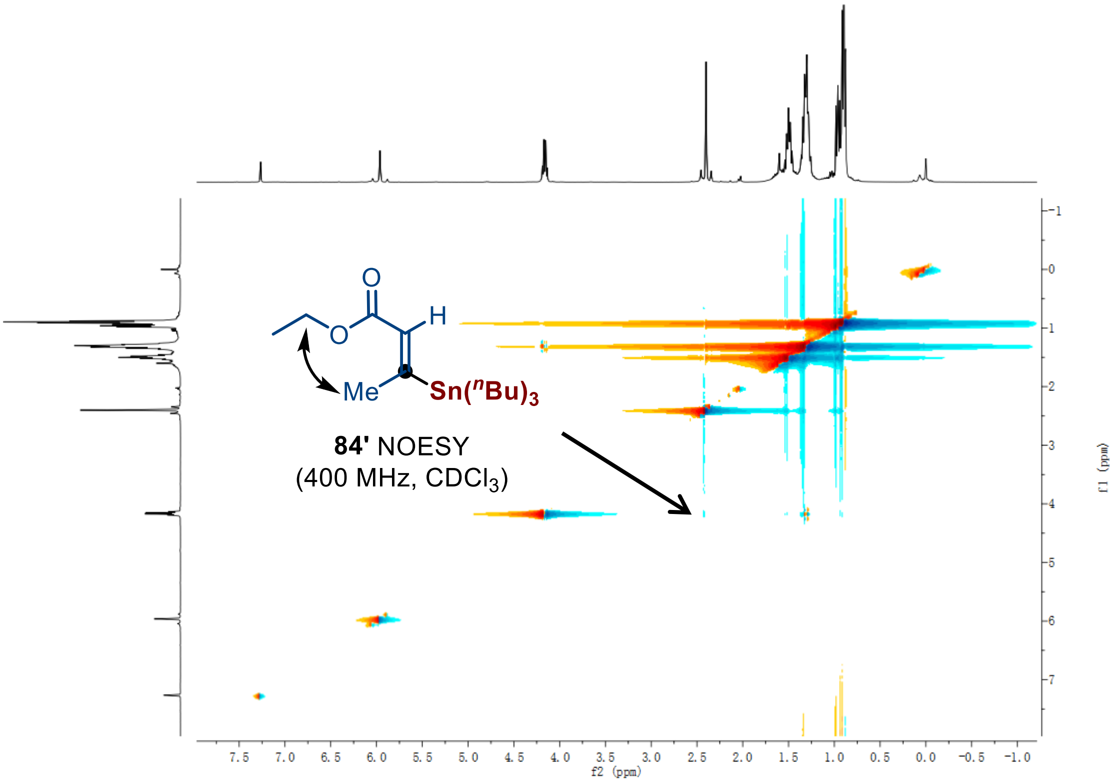

Supplementary Figure 272. **NOESY** of **84'**

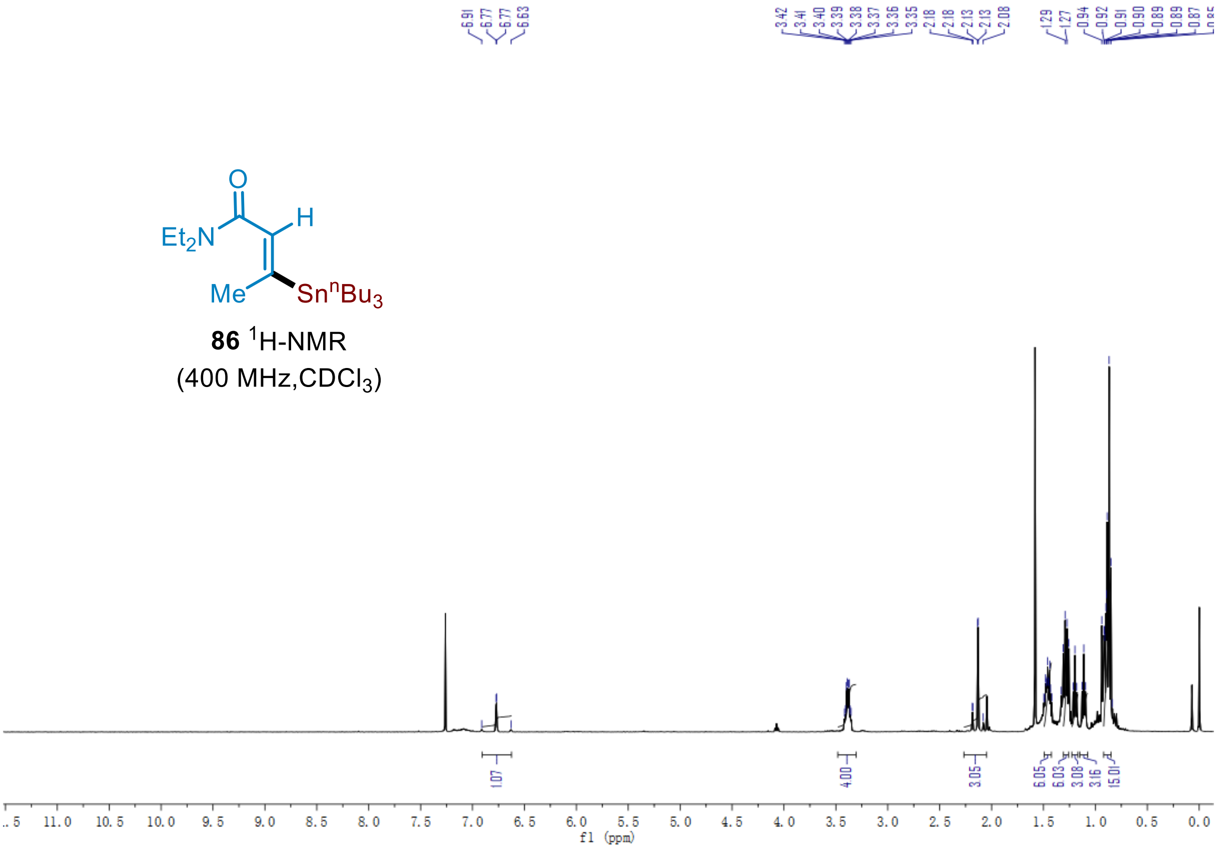

Supplementary Figure 273. **^{1}H-NMR** (400 MHz, $CDCl_3$, 298K) of **86**

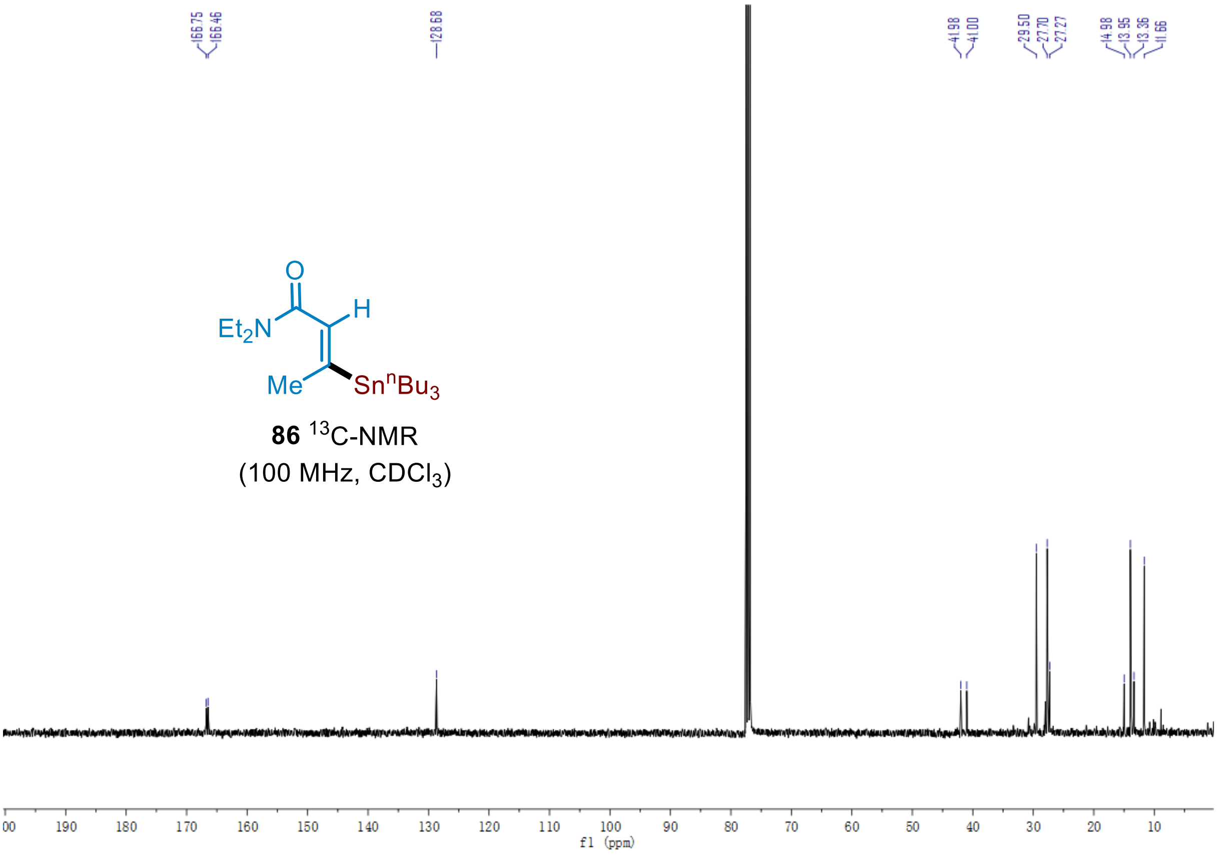

Supplementary Figure 274. **^{13}C-NMR** (100 MHz, $CDCl_3$, 298K) of **86**

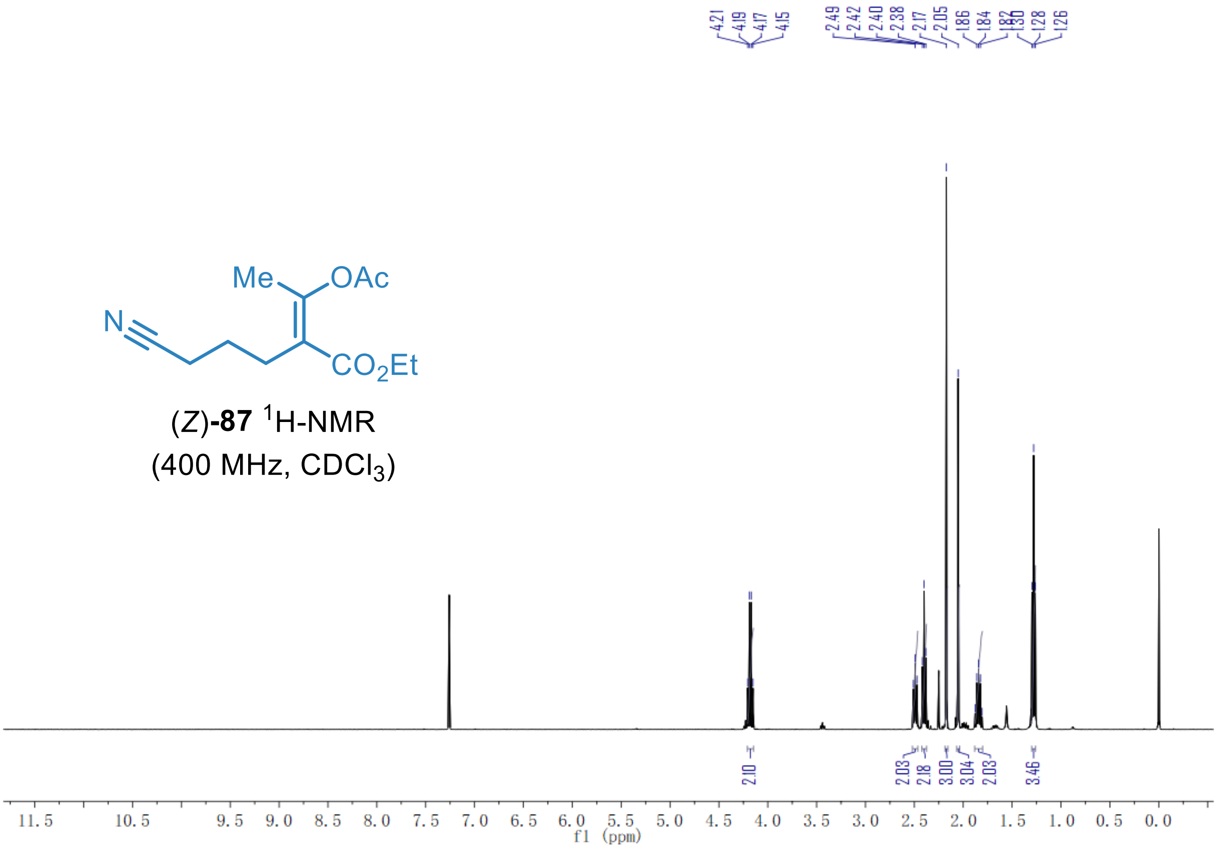

Supplementary Figure 275. **^{1}H-NMR** (400 MHz, $CDCl_3$, 298K) of (*Z*)-**87**

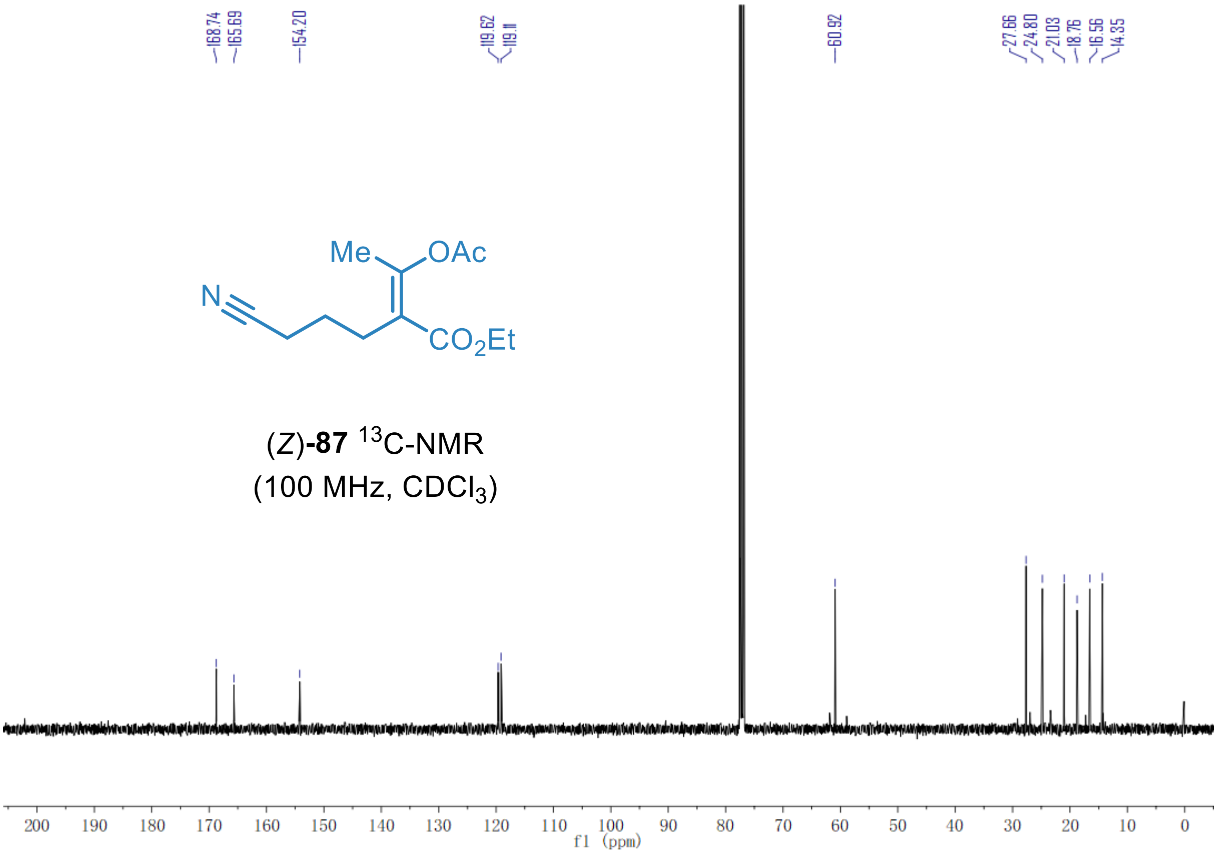

Supplementary Figure 276. **^{13}C-NMR** (100 MHz, $CDCl_3$, 298K) of (*Z*)-**87**

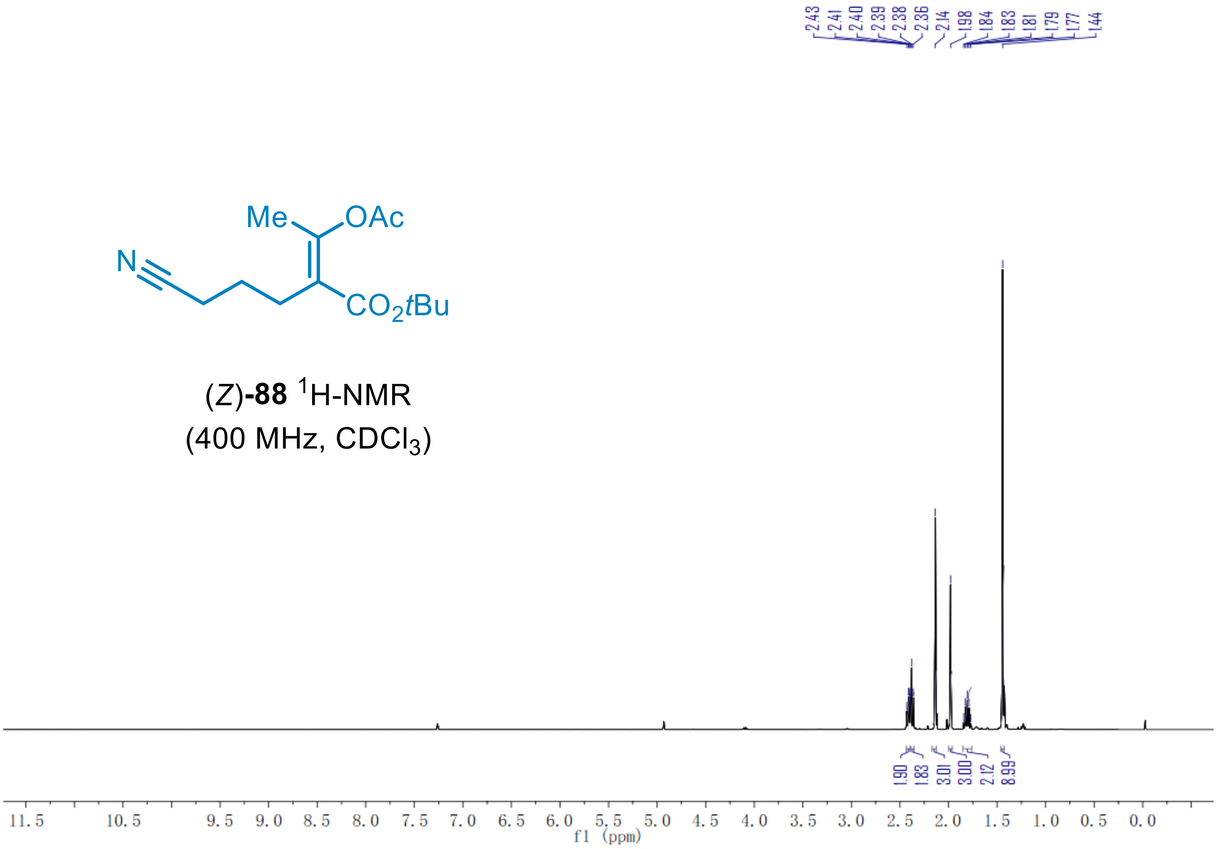

Supplementary Figure 277. **^{1}H-NMR** (400 MHz, $CDCl_3$, 298K) of (*Z*)-**88**

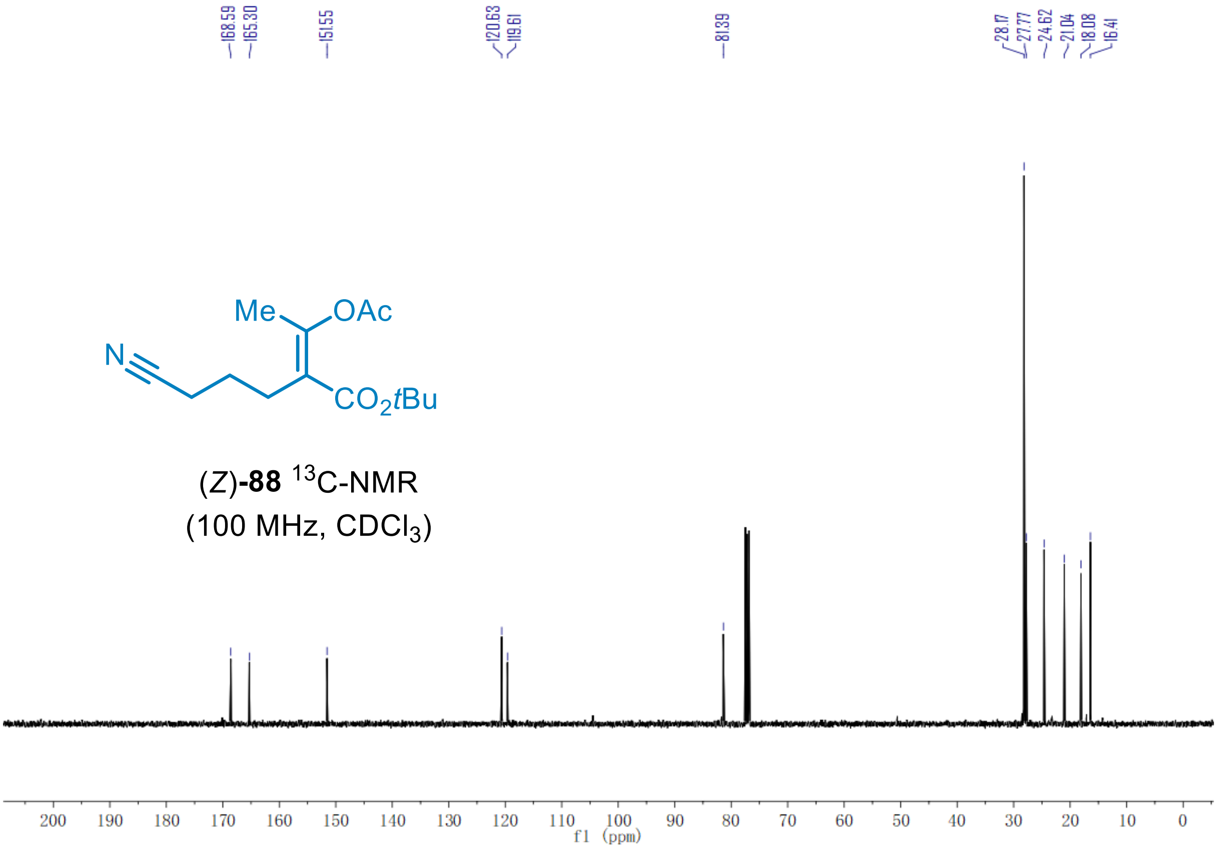

Supplementary Figure 278. **^{13}C-NMR** (100 MHz, $CDCl_3$, 298K) of (*Z*)-**88**

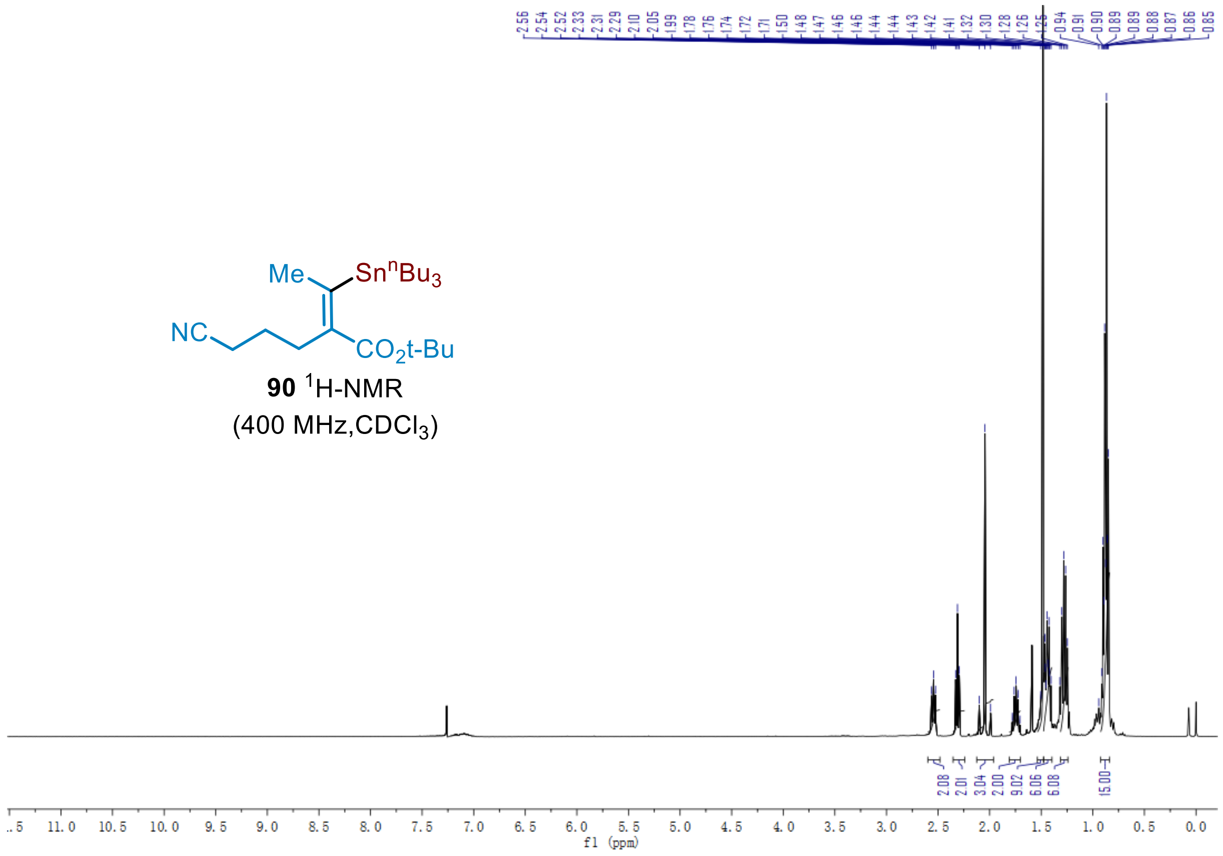

Supplementary Figure 279. **^{1}H-NMR** (400 MHz, $CDCl_3$, 298K) of **90**

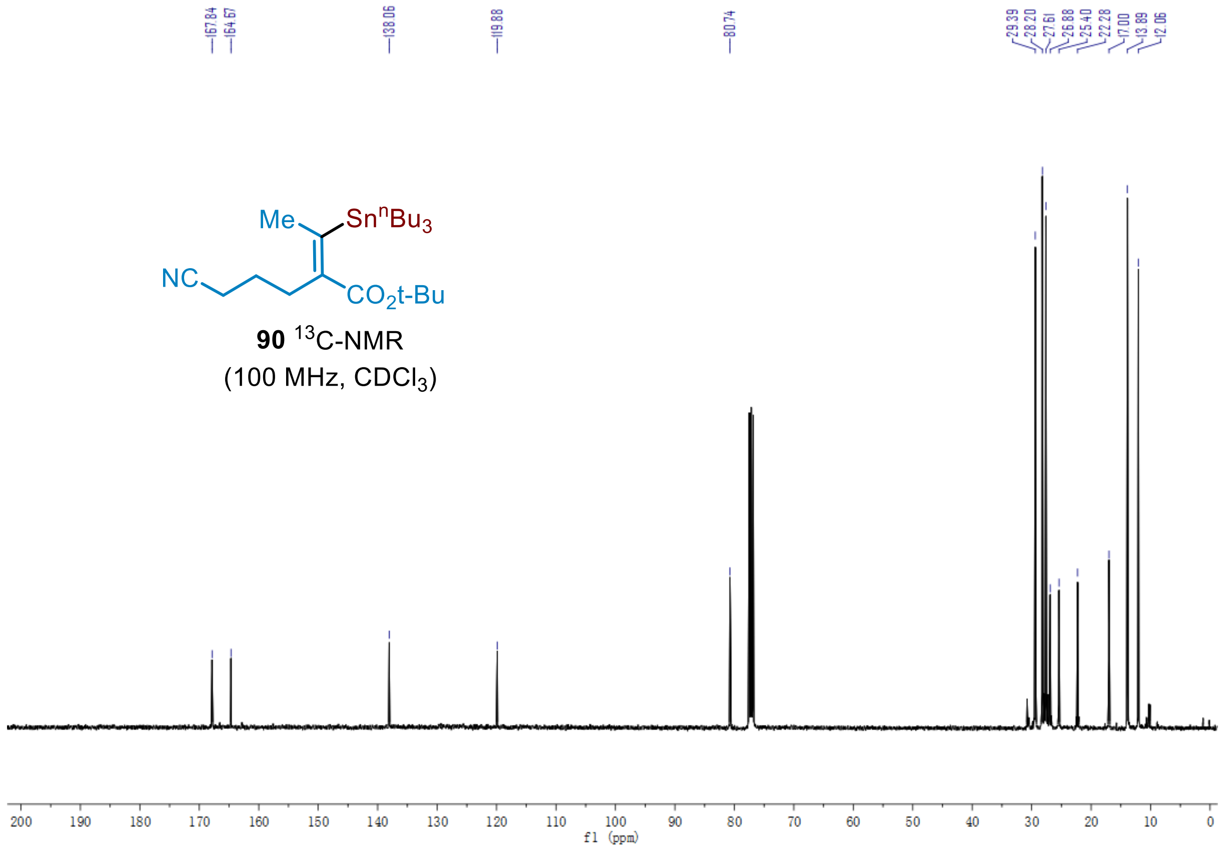

Supplementary Figure 280. **^{13}C-NMR** (100 MHz, $CDCl_3$, 298K) of **90**

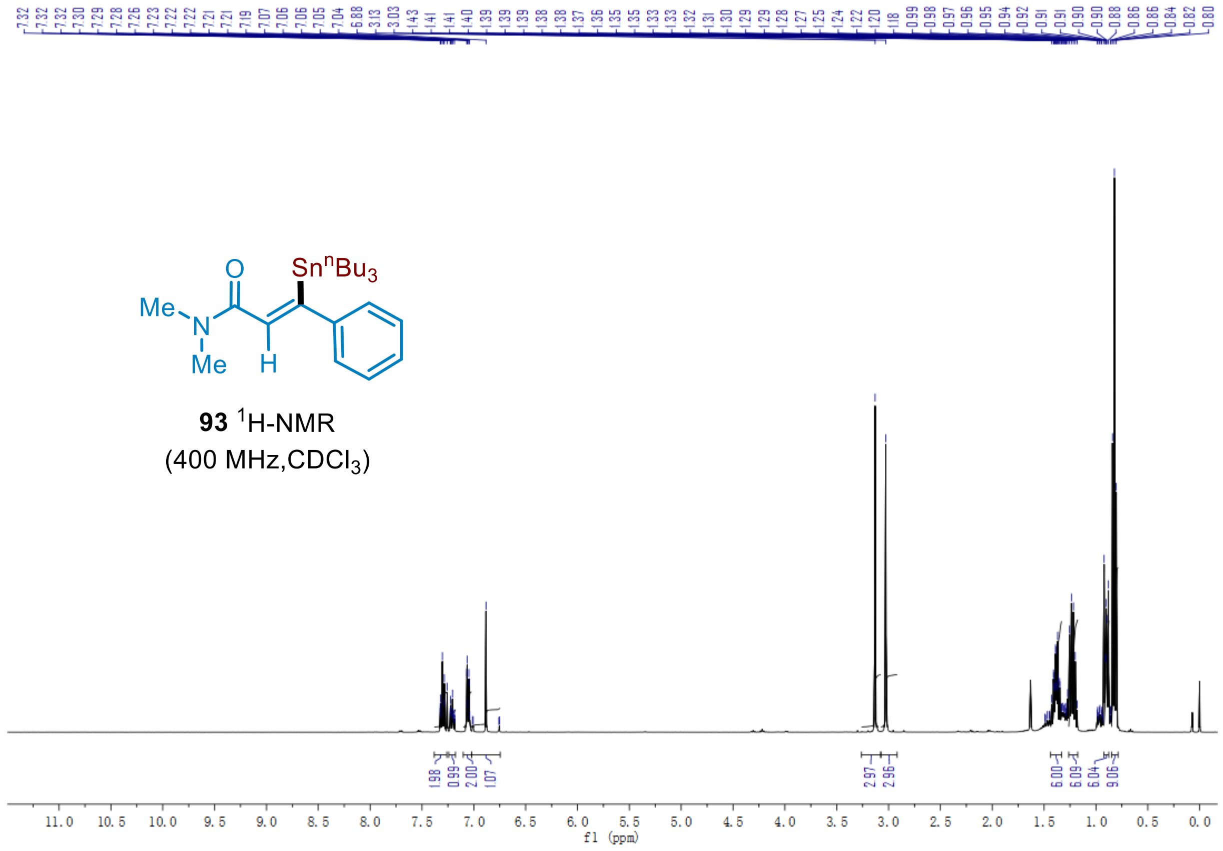

Supplementary Figure 281. **^{1}H-NMR** (400 MHz, $CDCl_3$, 298K) of **93**

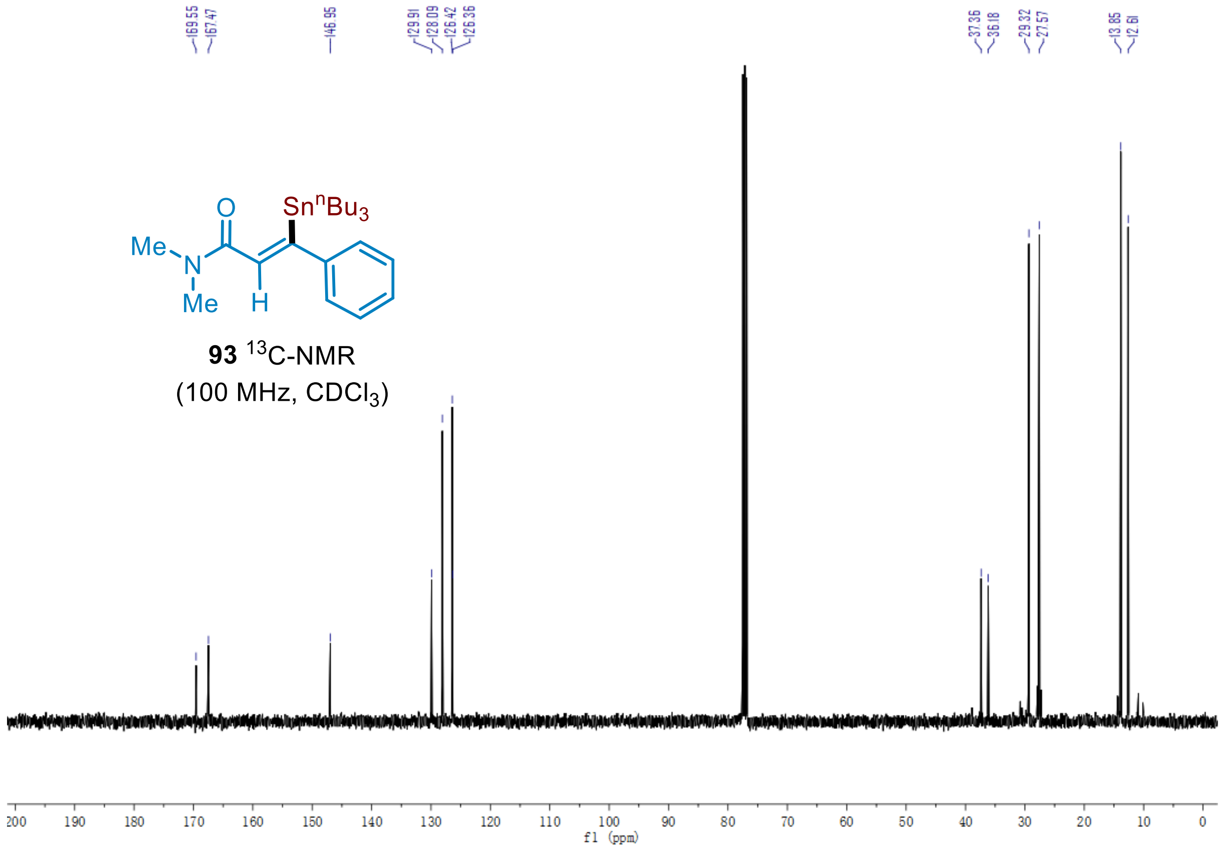

Supplementary Figure 282. **^{13}C-NMR** (100 MHz, $CDCl_3$, 298K) of **93**

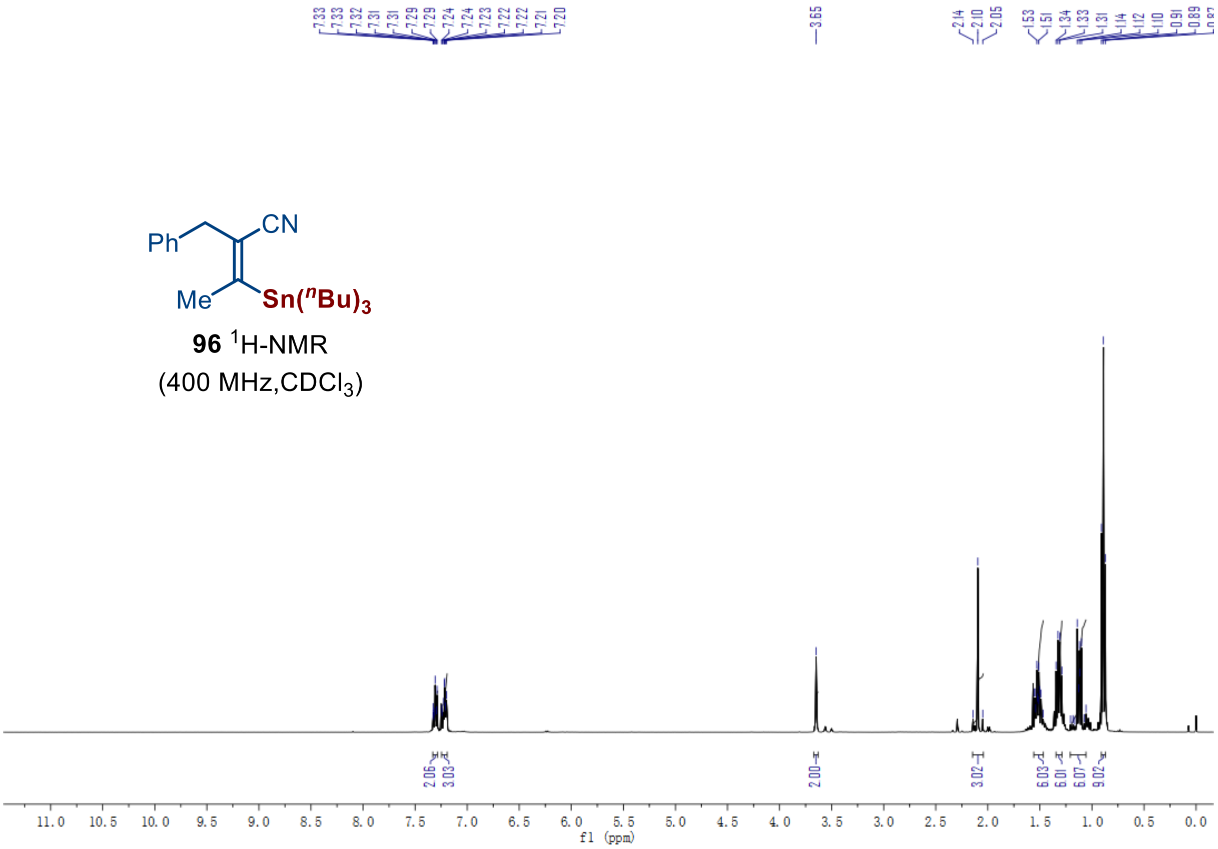

Supplementary Figure 283. **^{1}H-NMR** (400 MHz, $CDCl_3$, 298K) of **96**

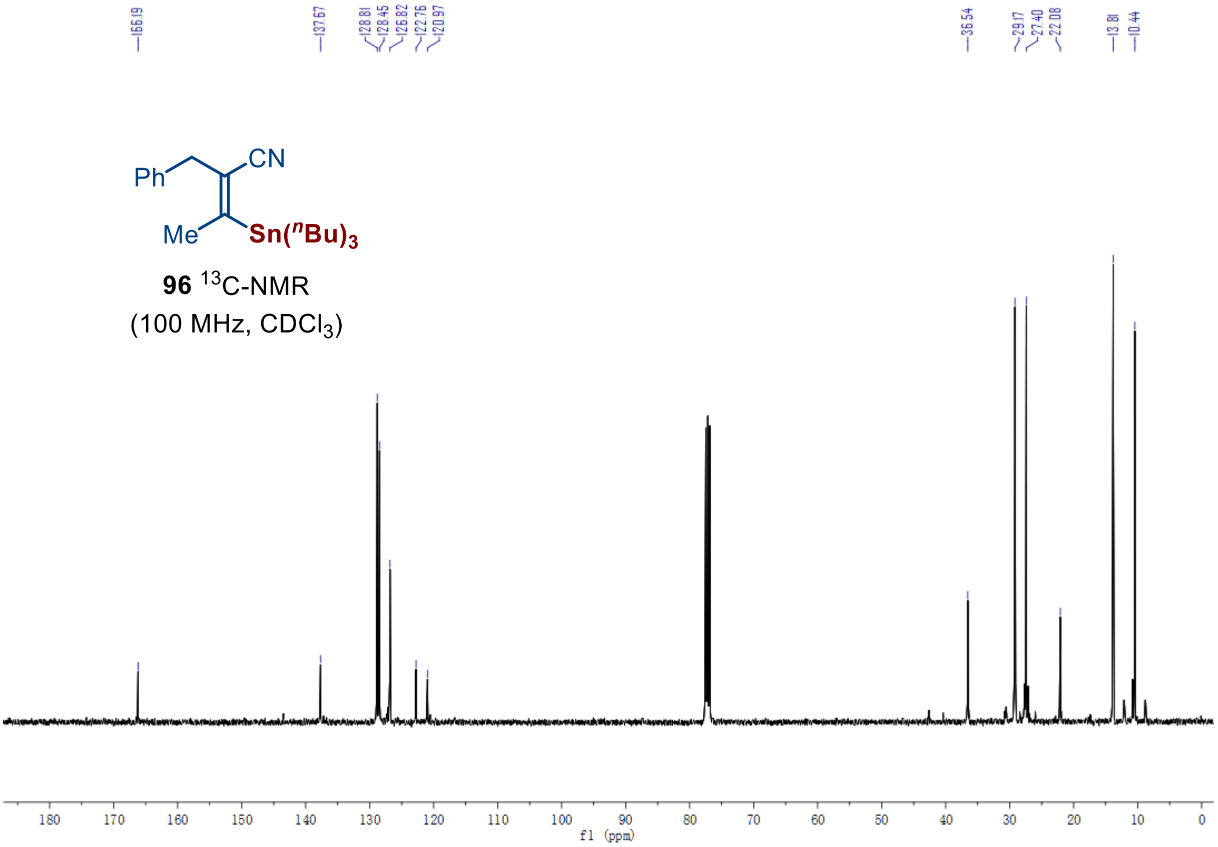

Supplementary Figure 284. **^{13}C-NMR** (100 MHz, $CDCl_3$, 298K) of **96**

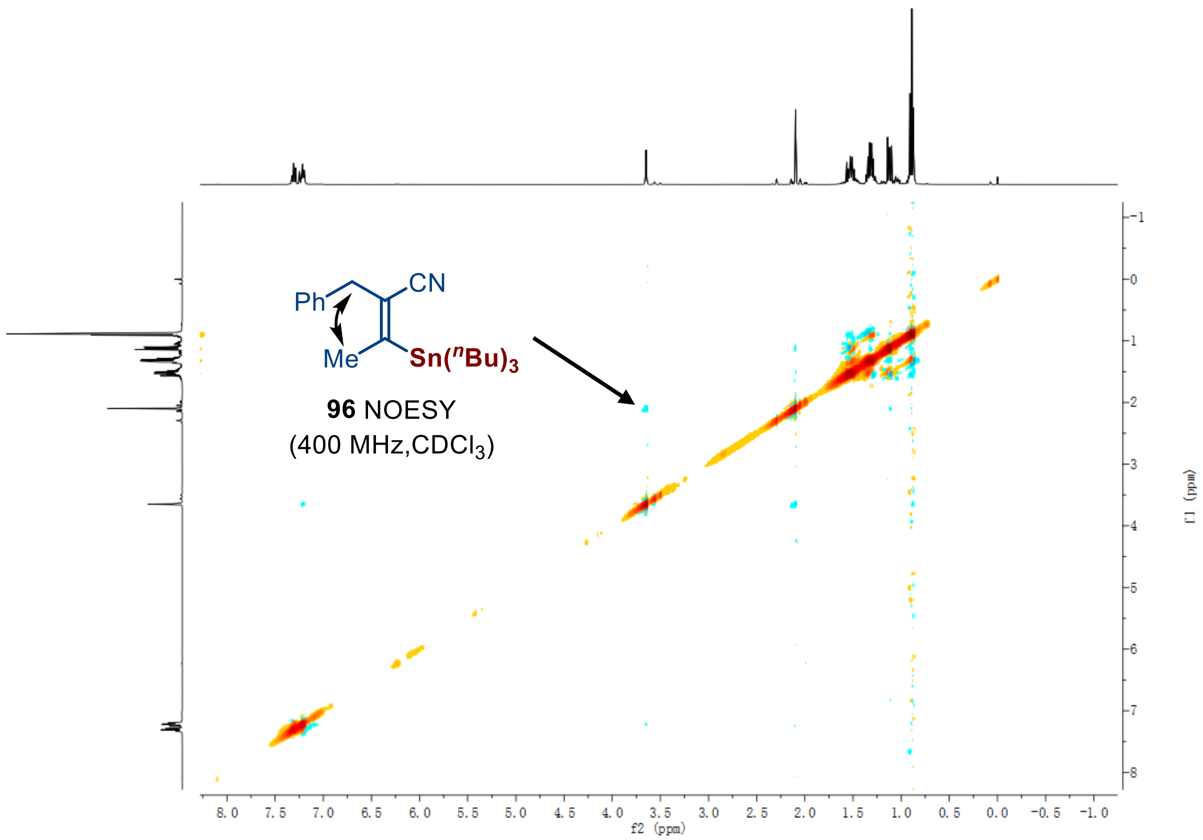

Supplementary Figure 285. **NOESY** of **96**

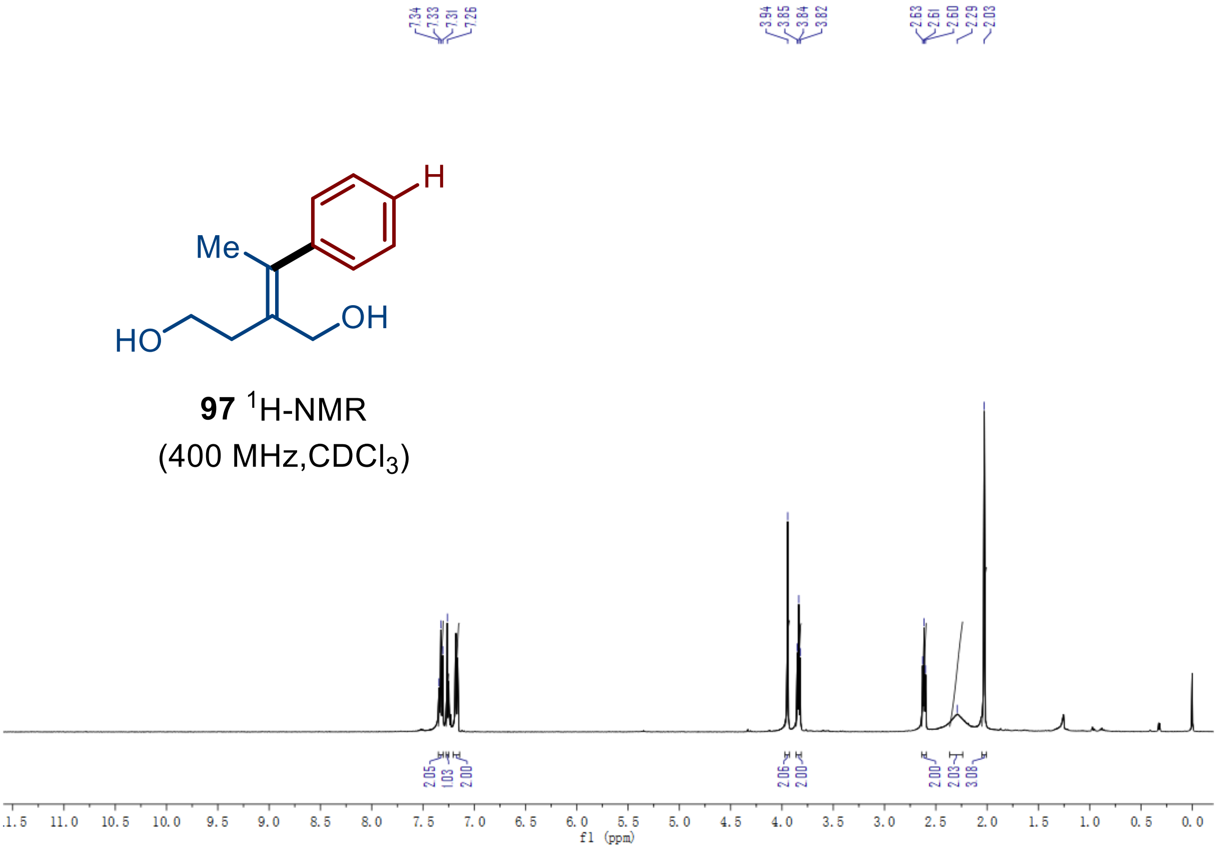

Supplementary Figure 286. **^{1}H-NMR** (400 MHz, $CDCl_3$, 298K) of **97**

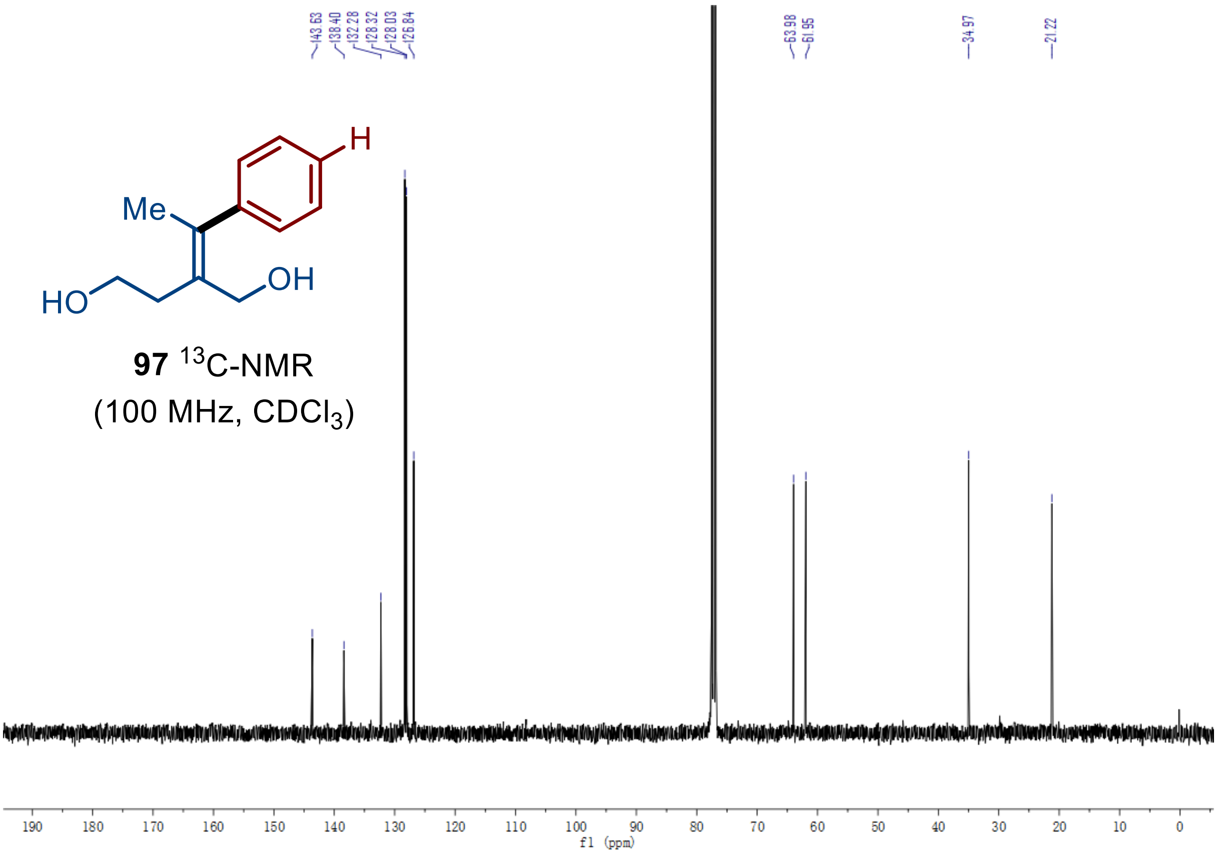

Supplementary Figure 287. **^{13}C-NMR** (100 MHz, $CDCl_3$, 298K) of **97**

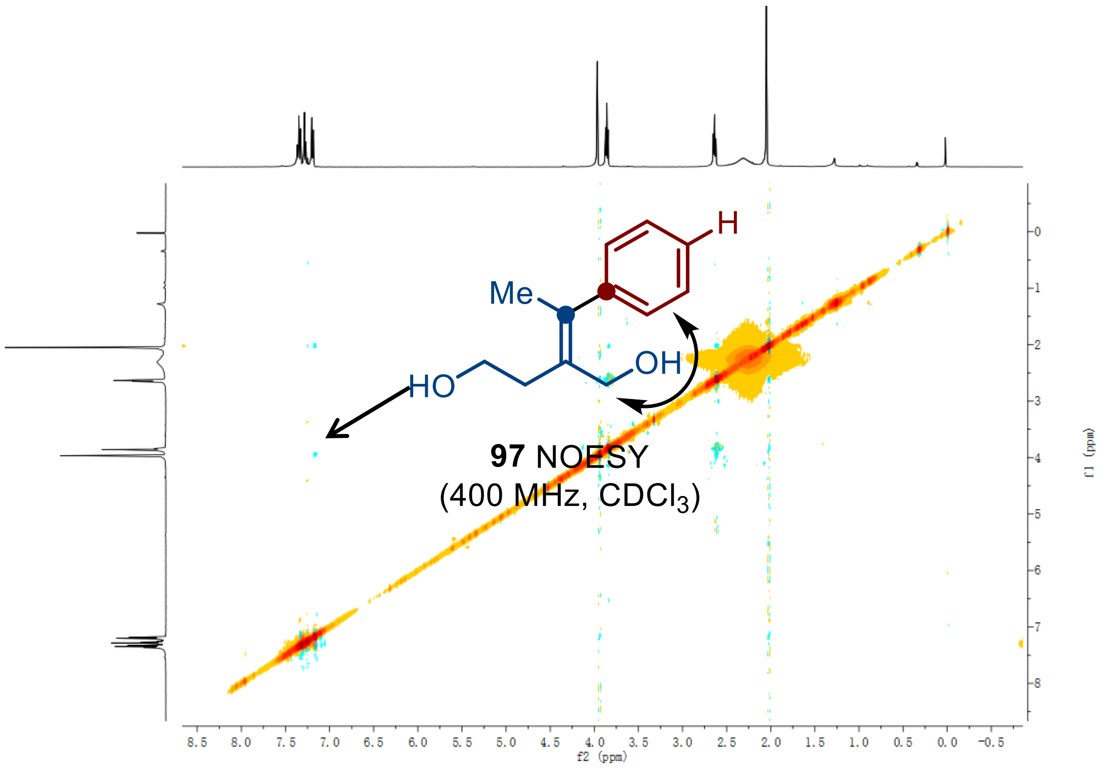

Supplementary Figure 288. **NOESY** of **97**

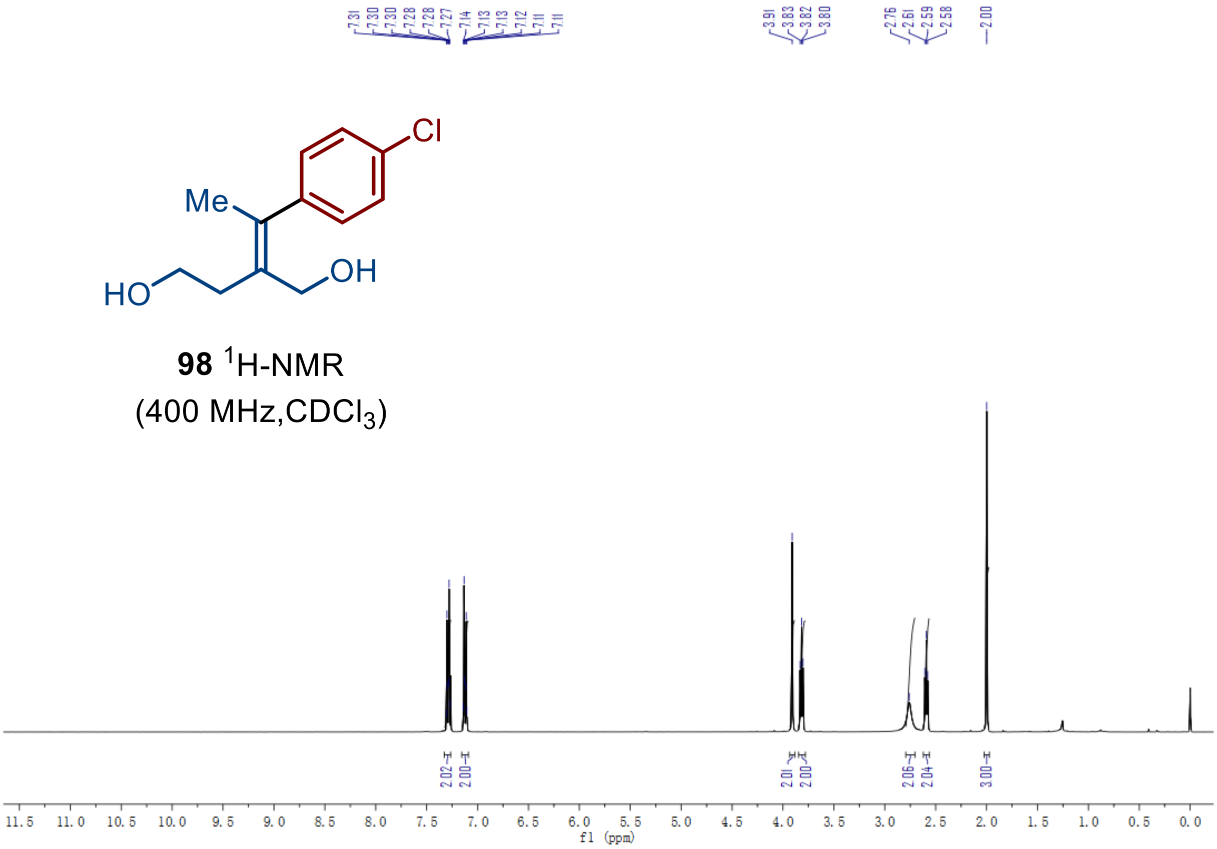

Supplementary Figure 289. **^{1}H-NMR** (400 MHz, $CDCl_3$, 298K) of **98**

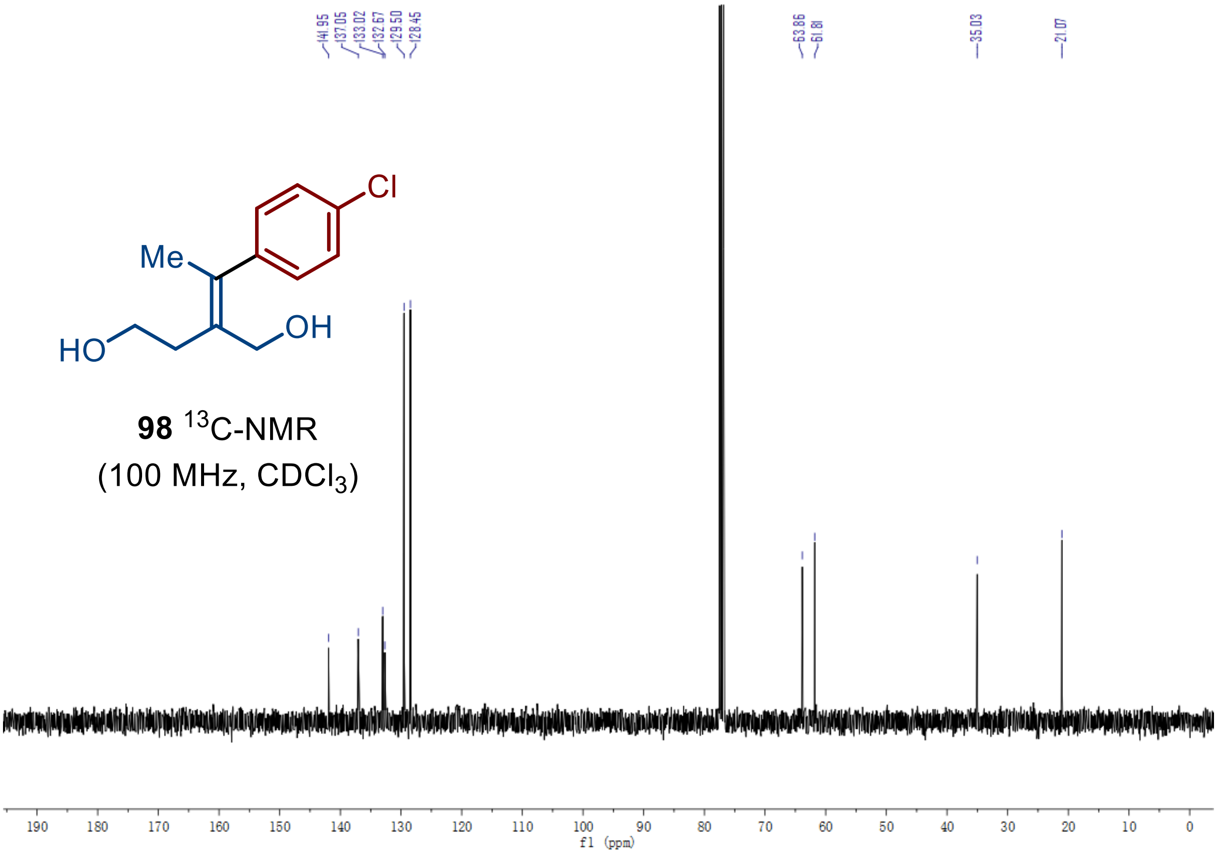

Supplementary Figure 290. **^{13}C-NMR** (100 MHz, $CDCl_3$, 298K) of **98**

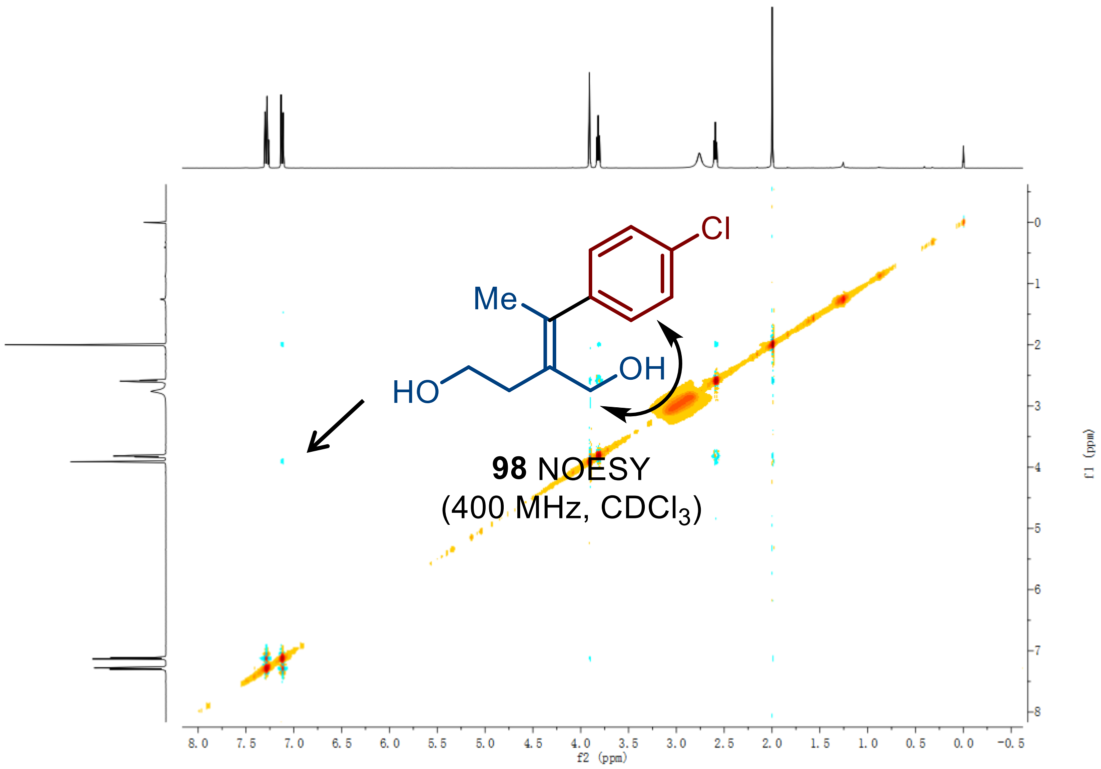

Supplementary Figure 291. **NOESY** of **98**

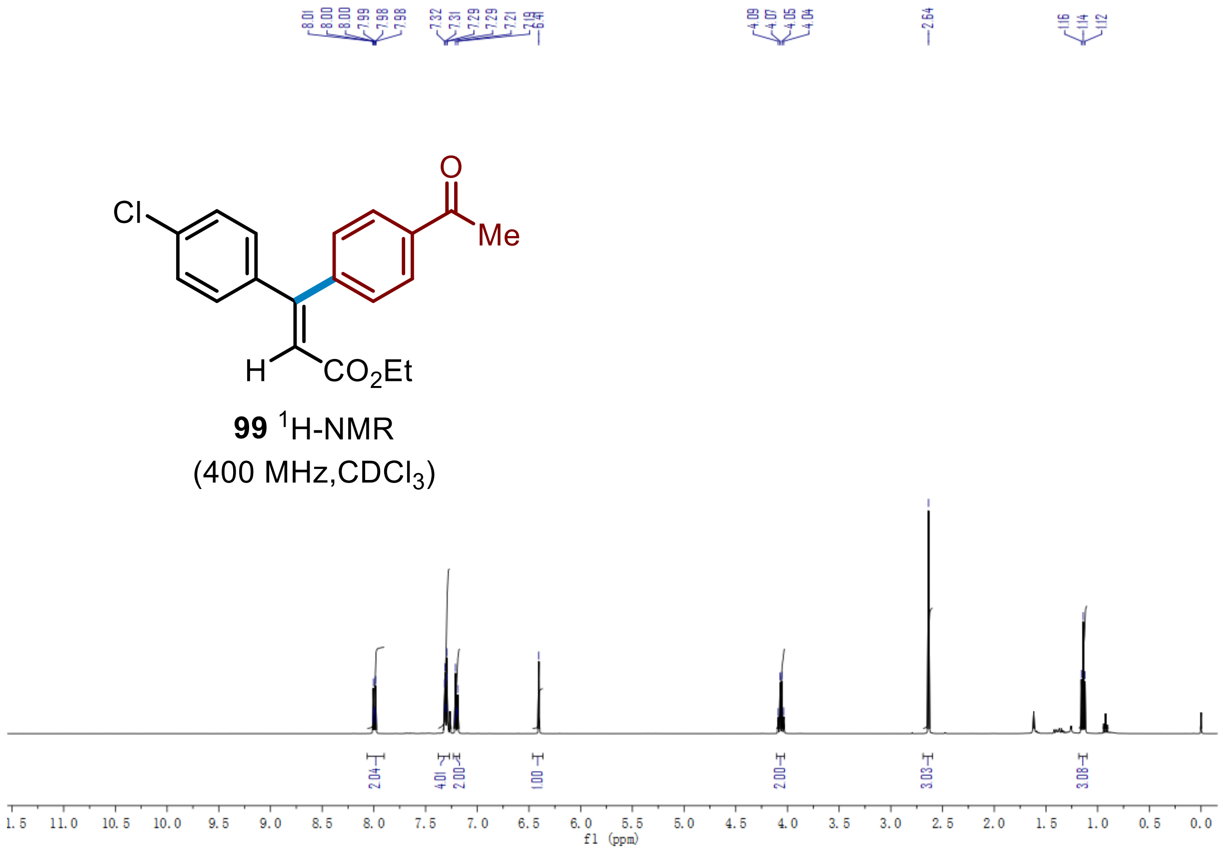

Supplementary Figure 292. **^{1}H-NMR** (400 MHz, $CDCl_3$, 298K) of **99**

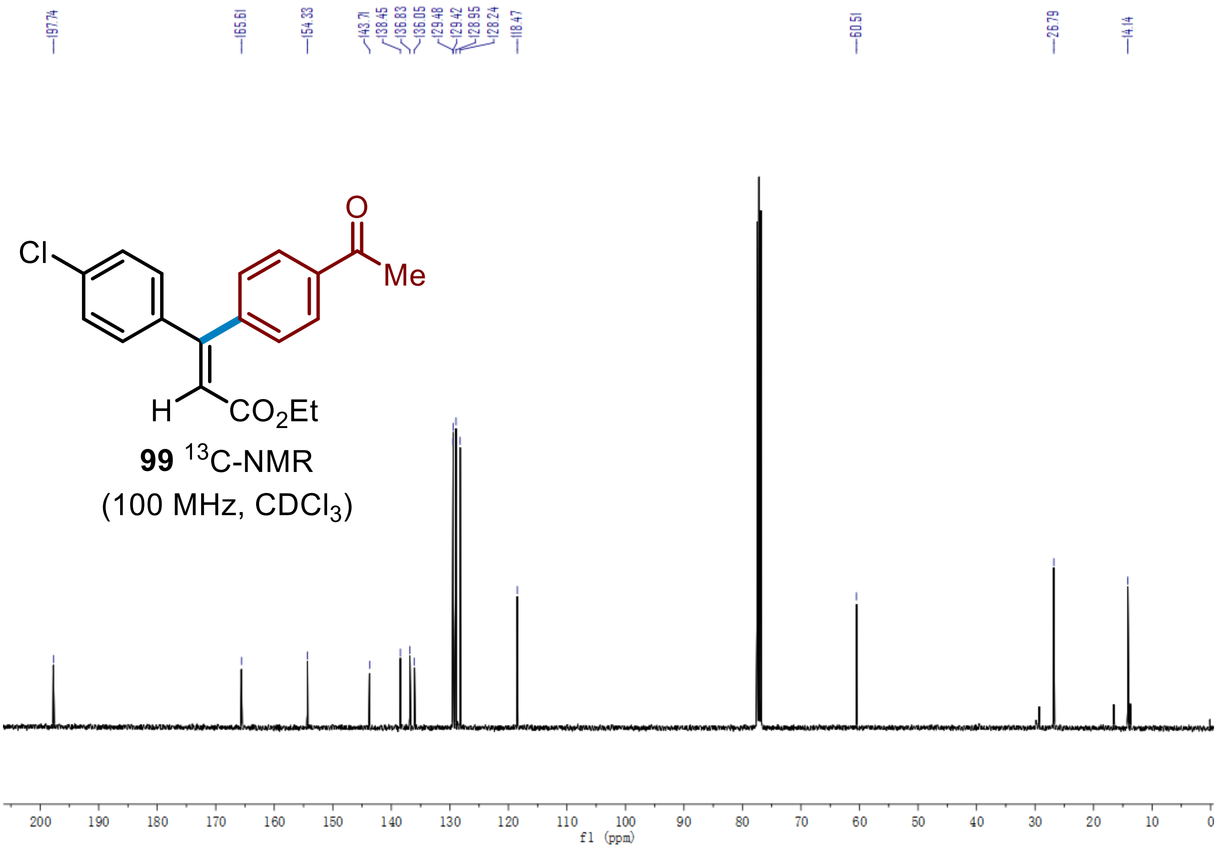

Supplementary Figure 293. **^{13}C-NMR** (100 MHz, $CDCl_3$, 298K) of **99**

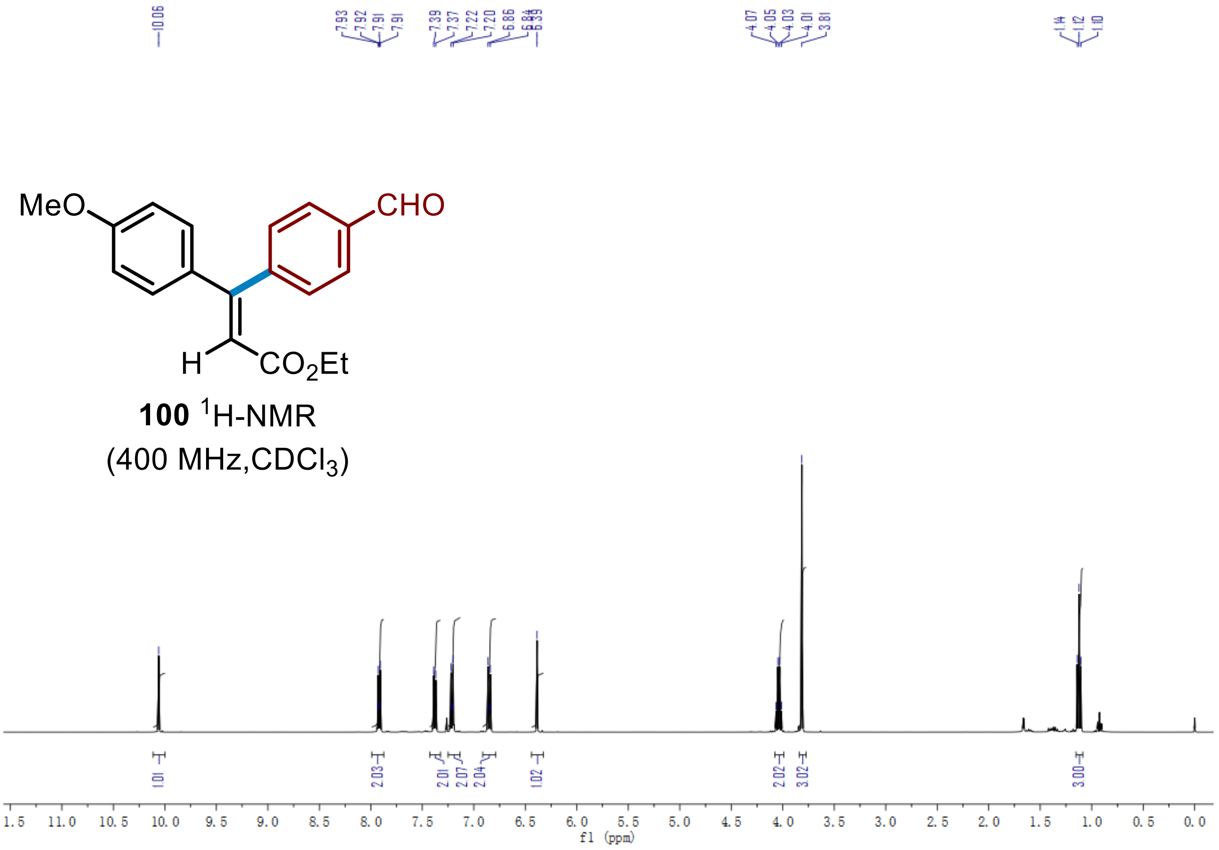

Supplementary Figure 294. **^{1}H-NMR** (400 MHz, $CDCl_3$, 298K) of **100**

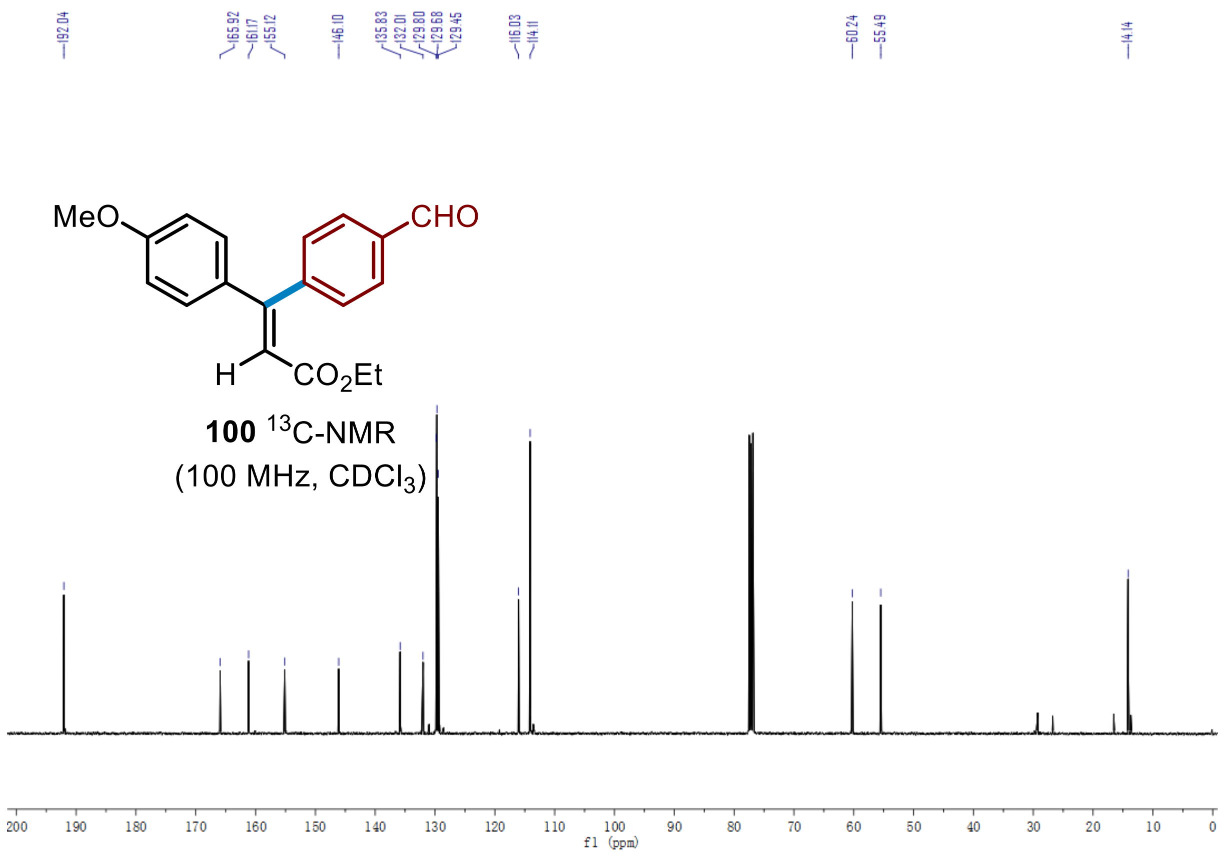

Supplementary Figure 295. **^{13}C-NMR** (100 MHz, $CDCl_3$, 298K) of **100**

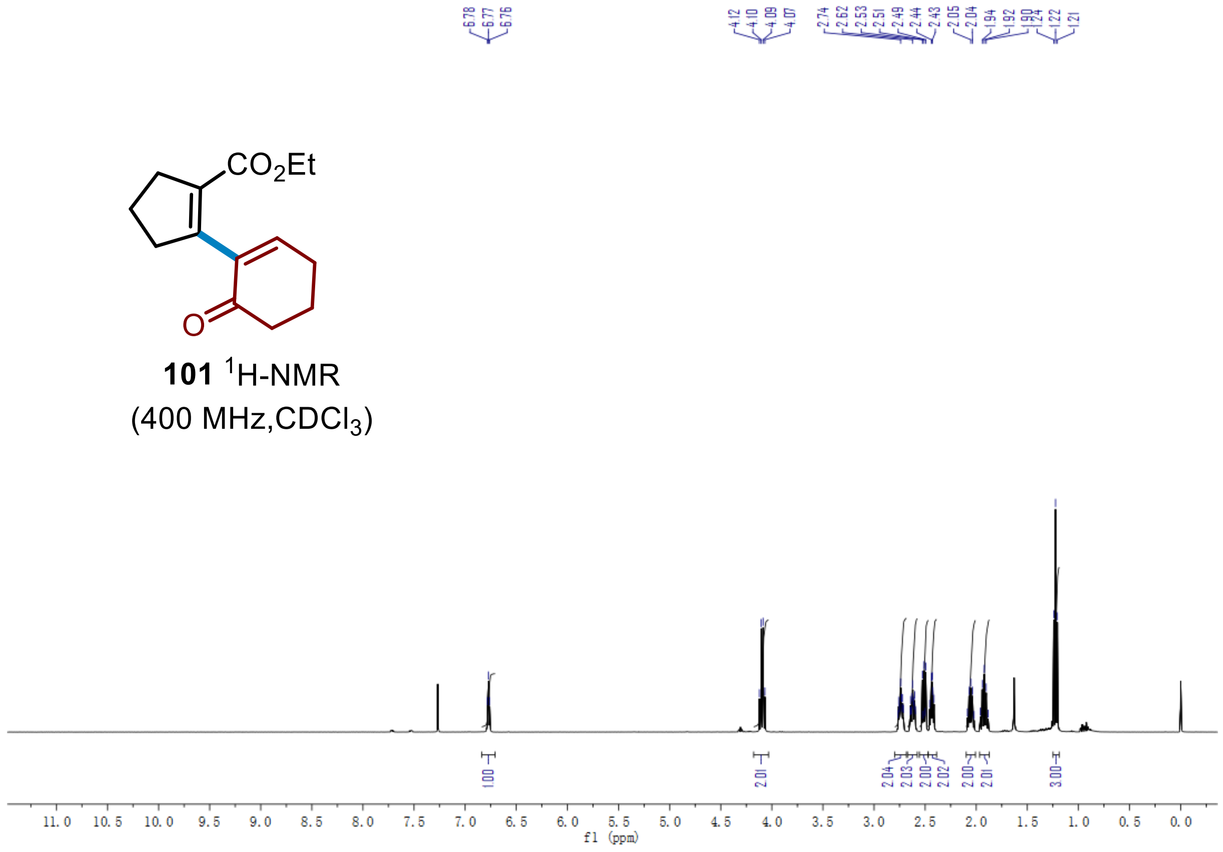

Supplementary Figure 296. **^{1}H-NMR** (400 MHz, $CDCl_3$, 298K) of **101**

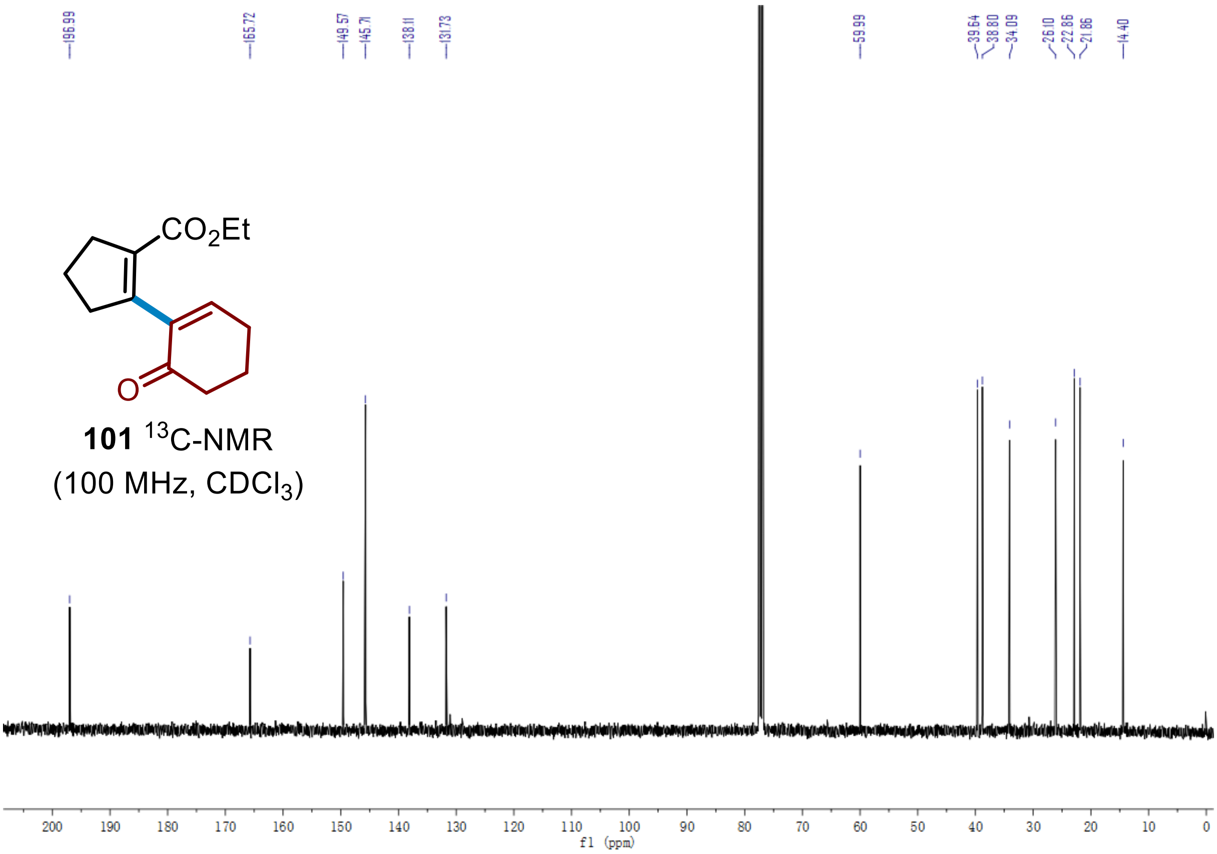

Supplementary Figure 297. **^{13}C-NMR** (100 MHz, CDCl$_3$, 298K) of **101**

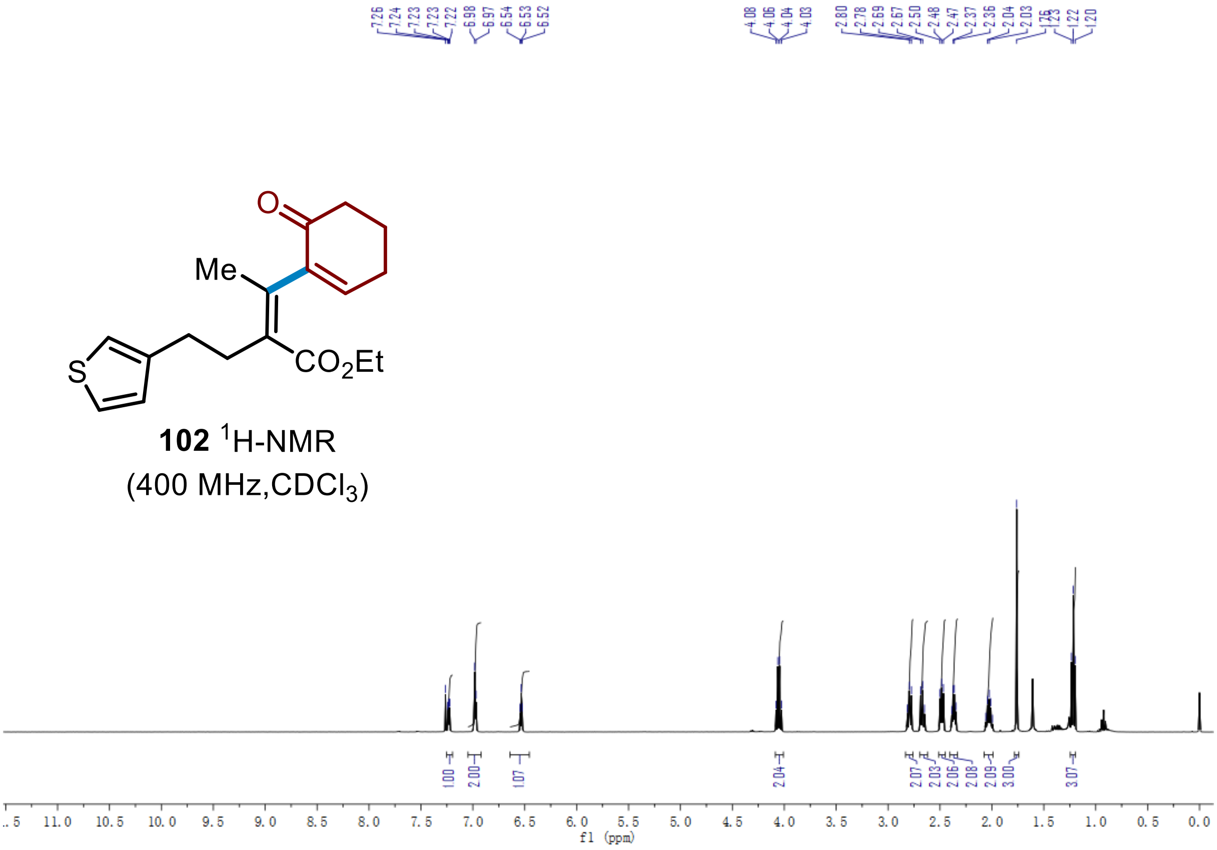

Supplementary Figure 298. **^{1}H-NMR** (400 MHz, CDCl$_3$, 298K) of **102**

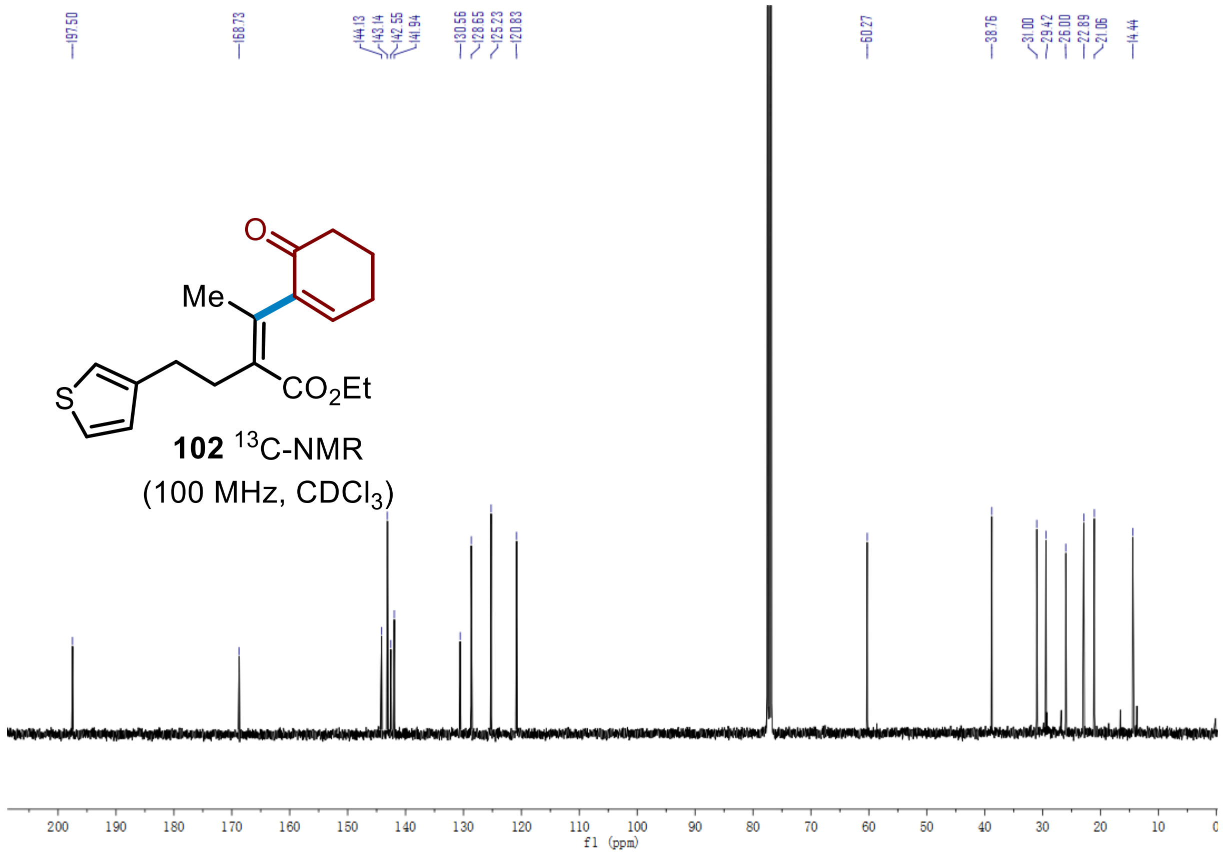

Supplementary Figure 299. **^{13}C-NMR** (100 MHz, $CDCl_3$, 298K) of **102**

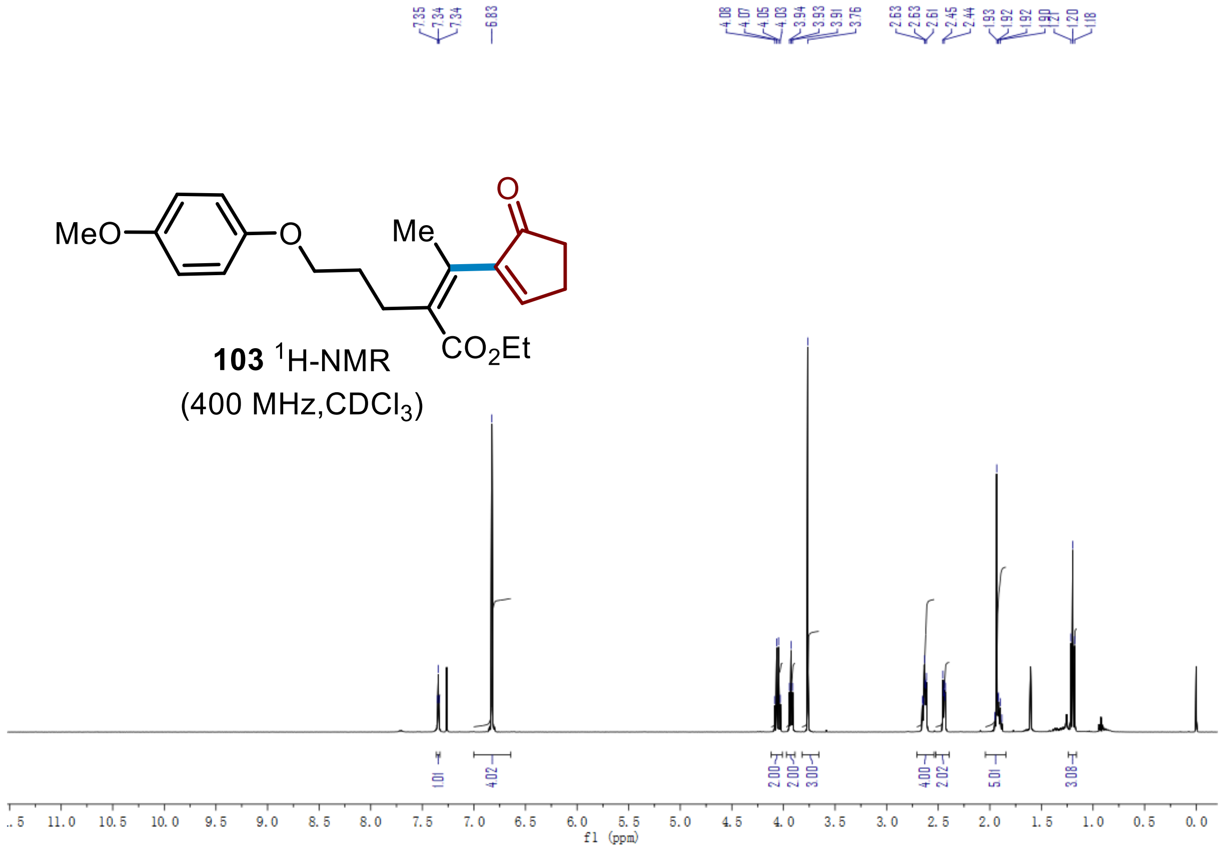

Supplementary Figure 300. **^{1}H-NMR** (400 MHz, $CDCl_3$, 298K) of **103**

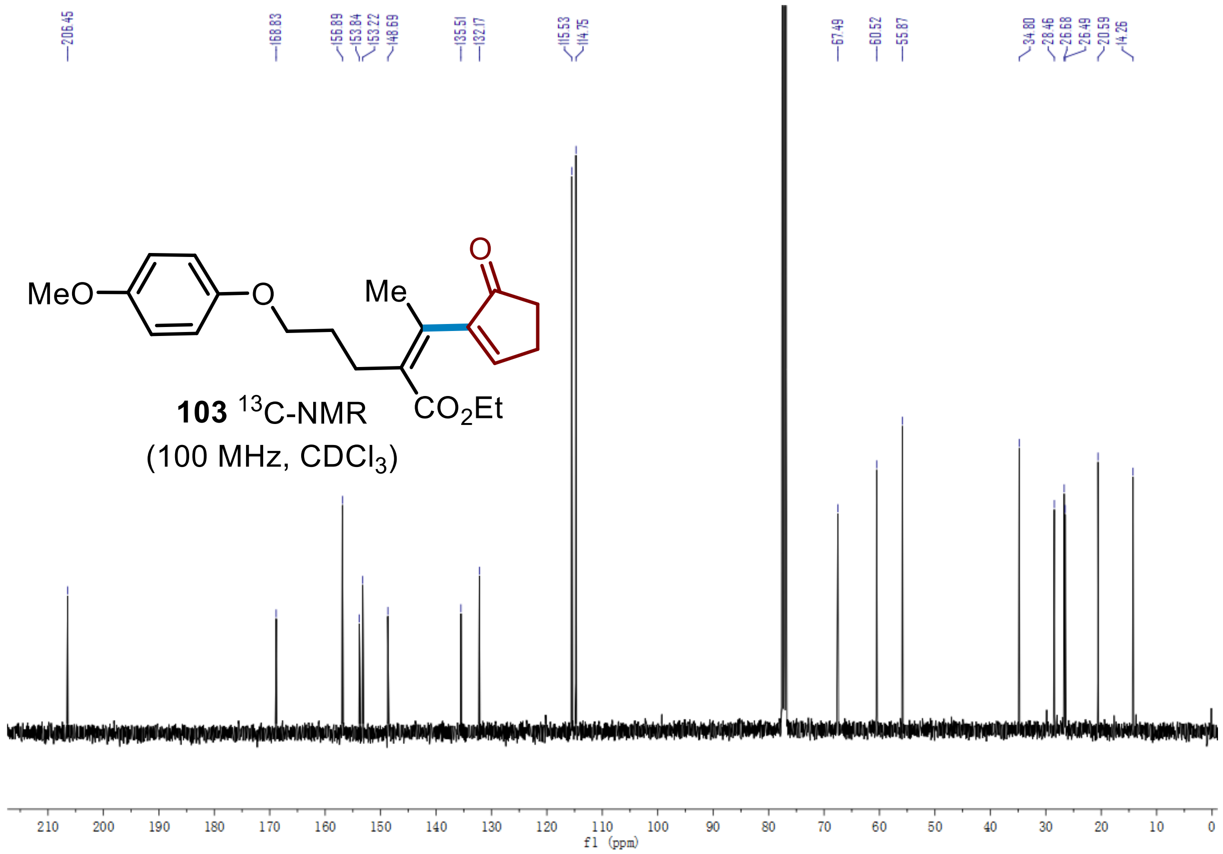

Supplementary Figure 301. **^{13}C-NMR** (100 MHz, $CDCl_3$, 298K) of **103**

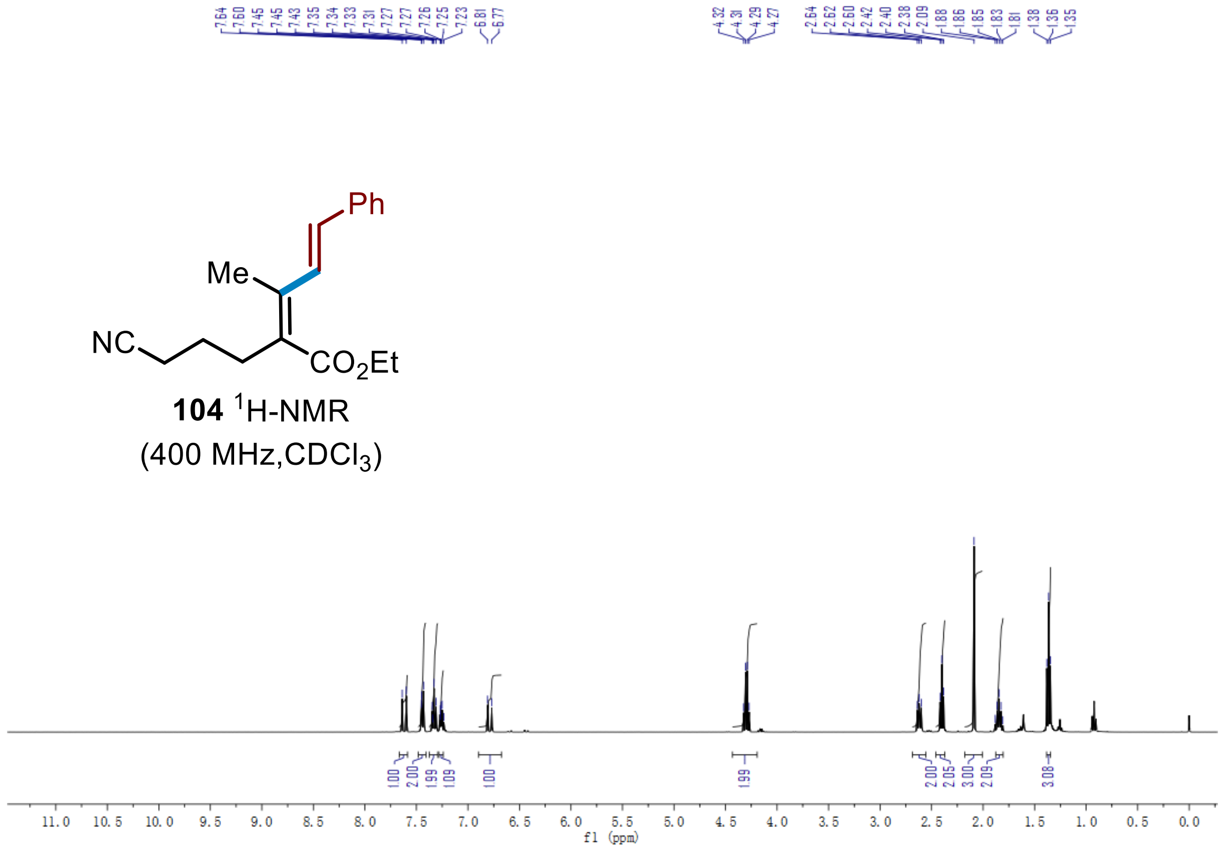

Supplementary Figure 302. **^{1}H-NMR** (400 MHz, $CDCl_3$, 298K) of **104**

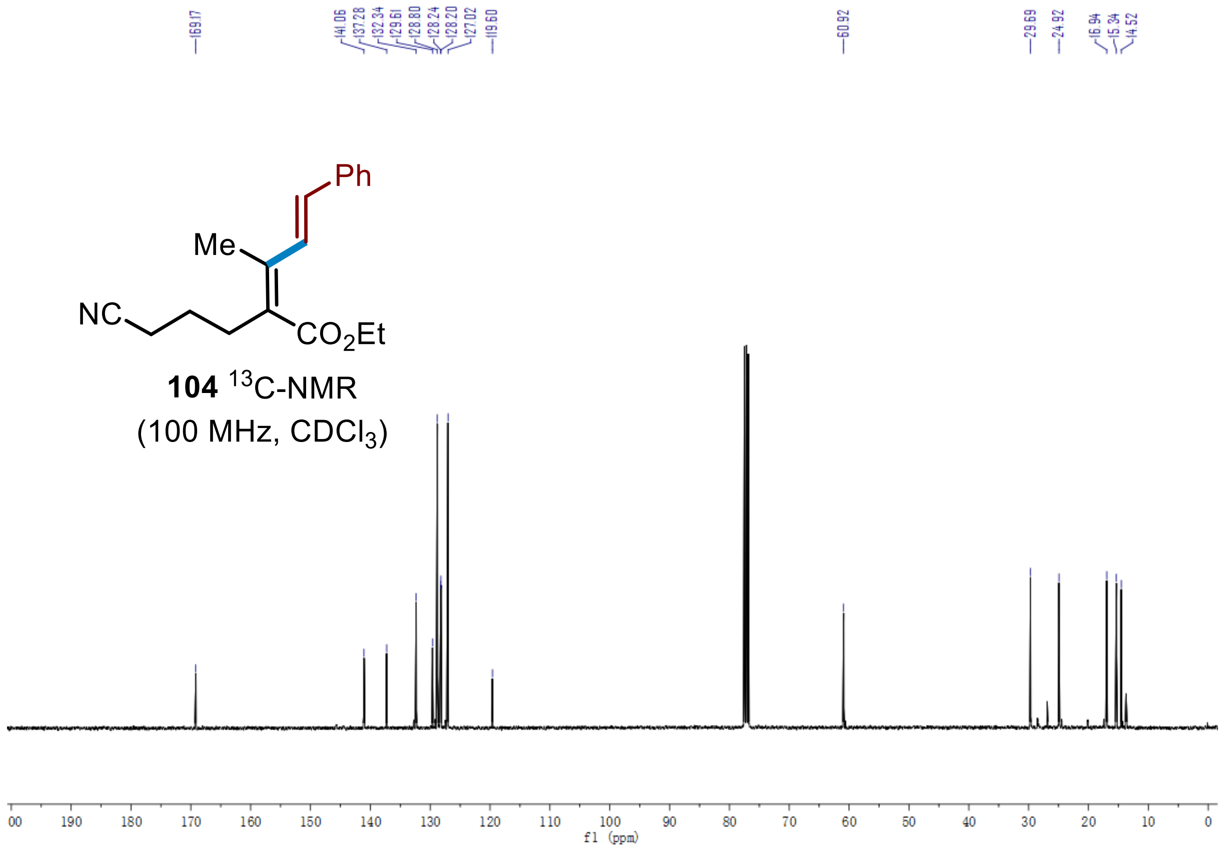

Supplementary Figure 303. **^{13}C-NMR** (100 MHz, $CDCl_3$, 298K) of **104**

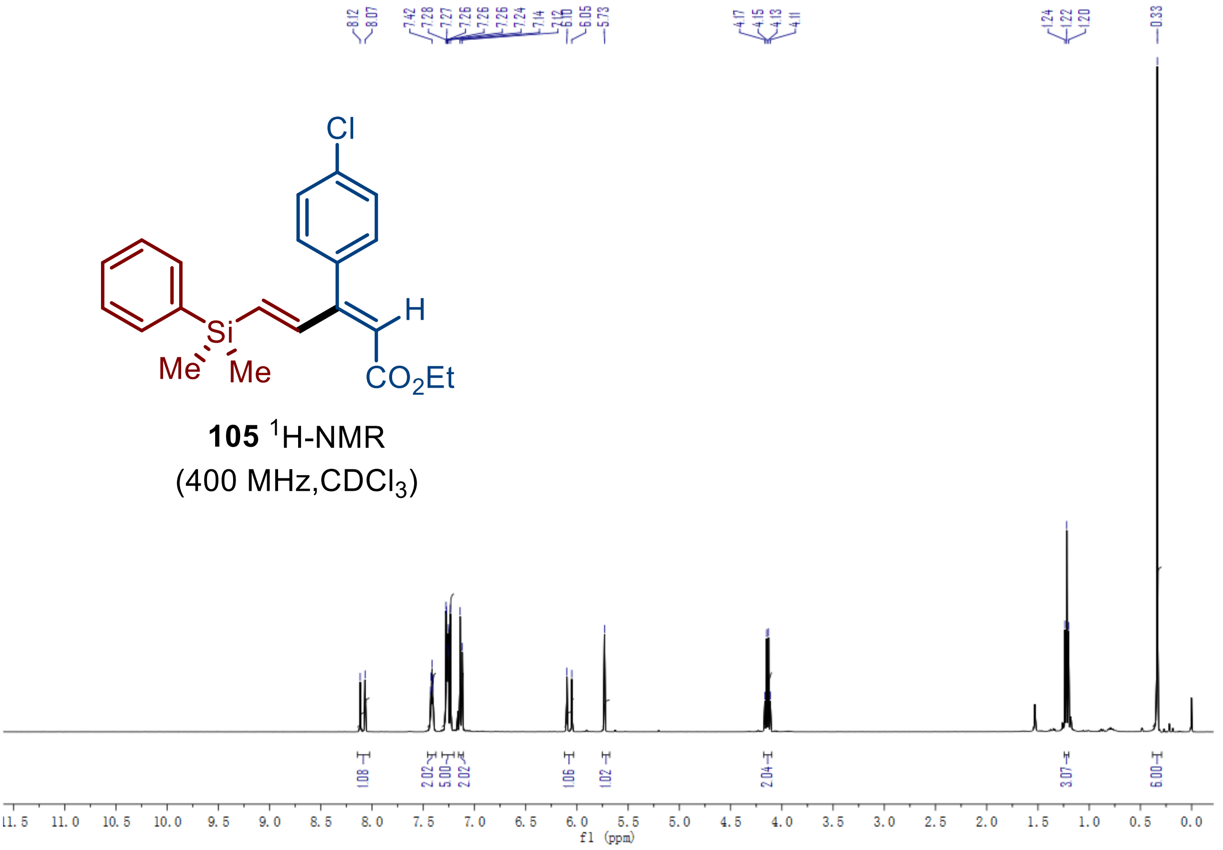

Supplementary Figure 304. **^{1}H-NMR** (400 MHz, $CDCl_3$, 298K) of **105**

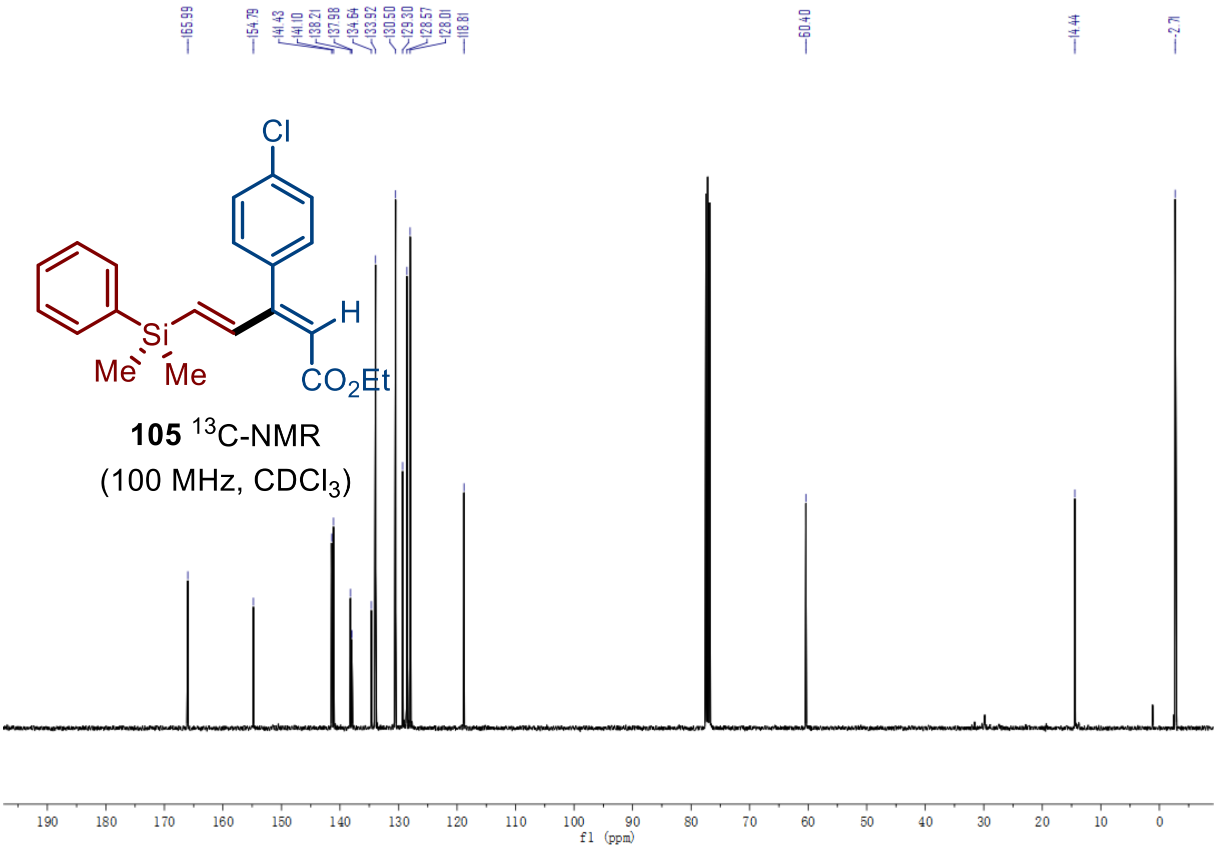

Supplementary Figure 305. **^{13}C-NMR** (100 MHz, $CDCl_3$, 298K) of **105**

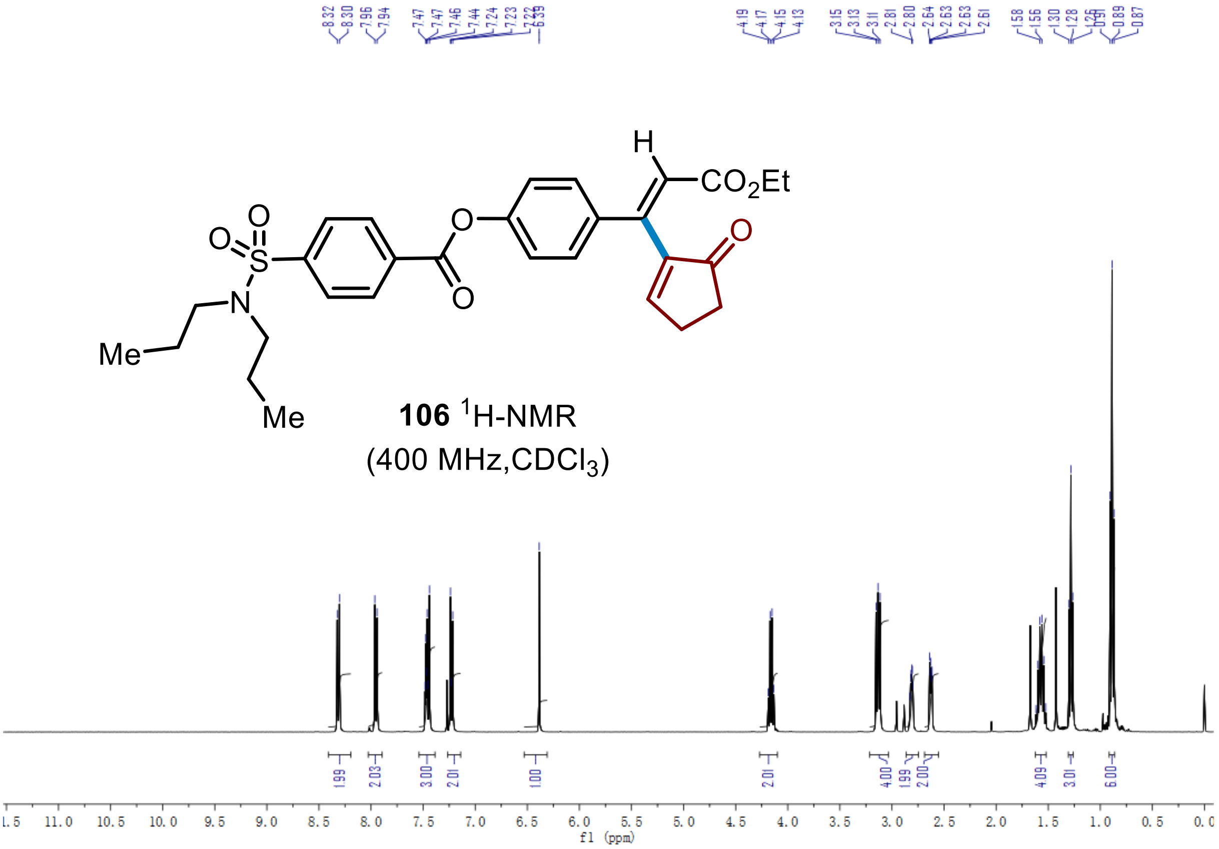

Supplementary Figure 306. **^{1}H-NMR** (400 MHz, $CDCl_3$, 298K) of **106**

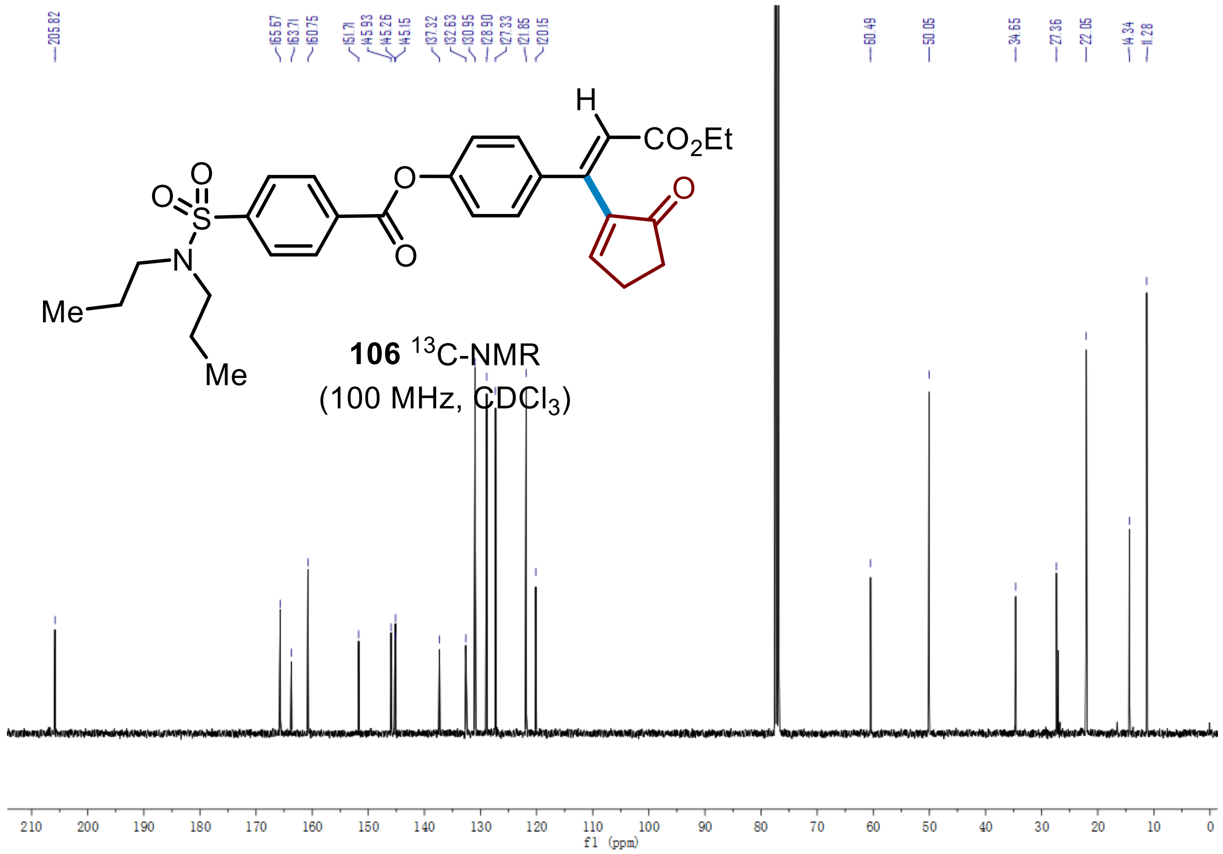

Supplementary Figure 307. **^{13}C-NMR** (100 MHz, CDCl$_3$, 298K) of **106**

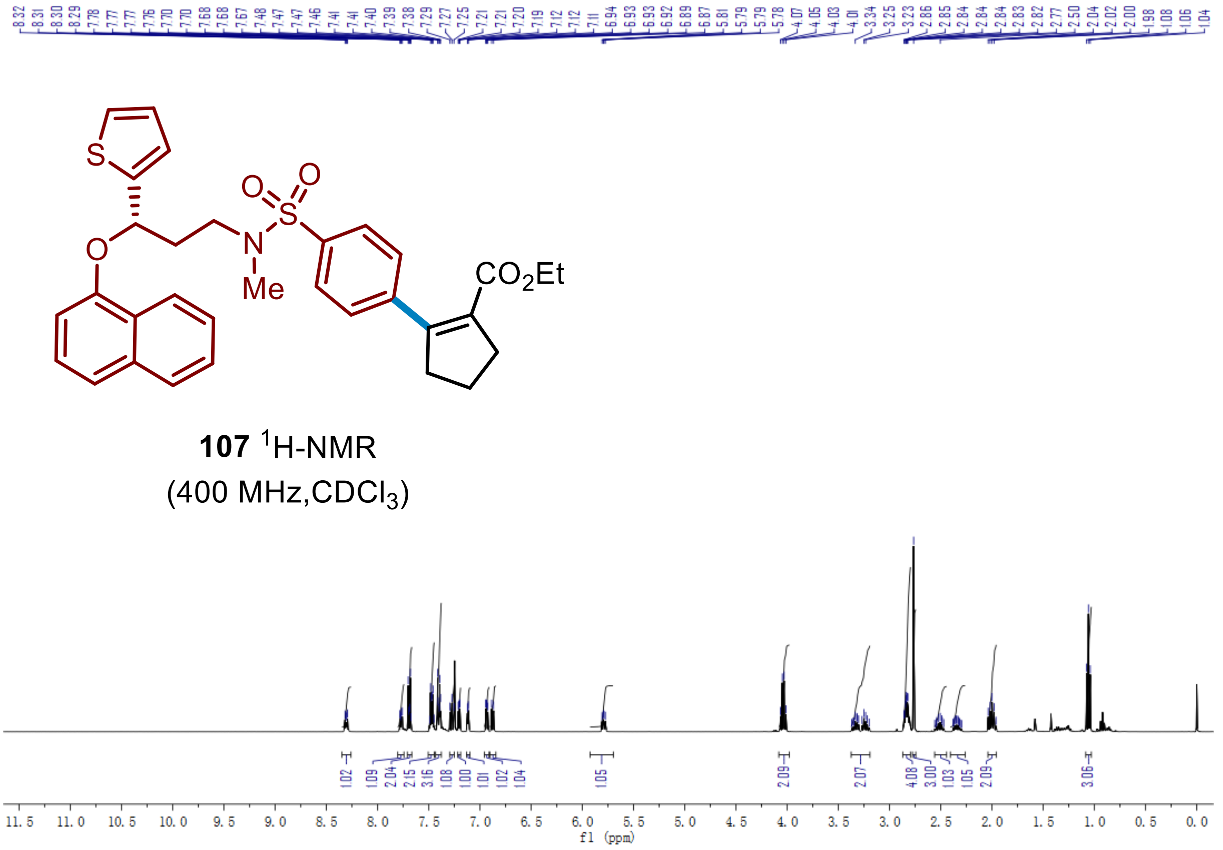

Supplementary Figure 308. **^{1}H-NMR** (400 MHz, CDCl$_3$, 298K) of **107**

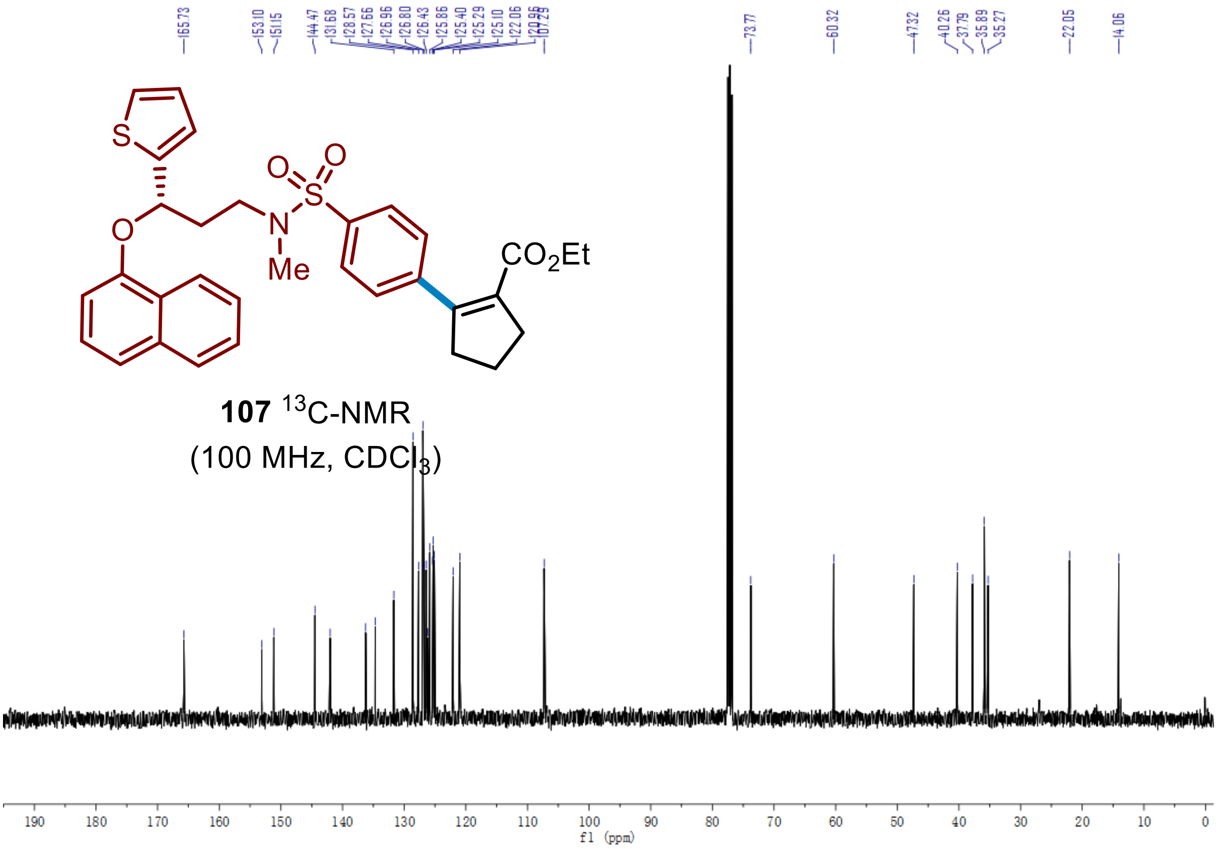

Supplementary Figure 309. **^{13}C-NMR** (100 MHz, $CDCl_3$, 298K) of **107**

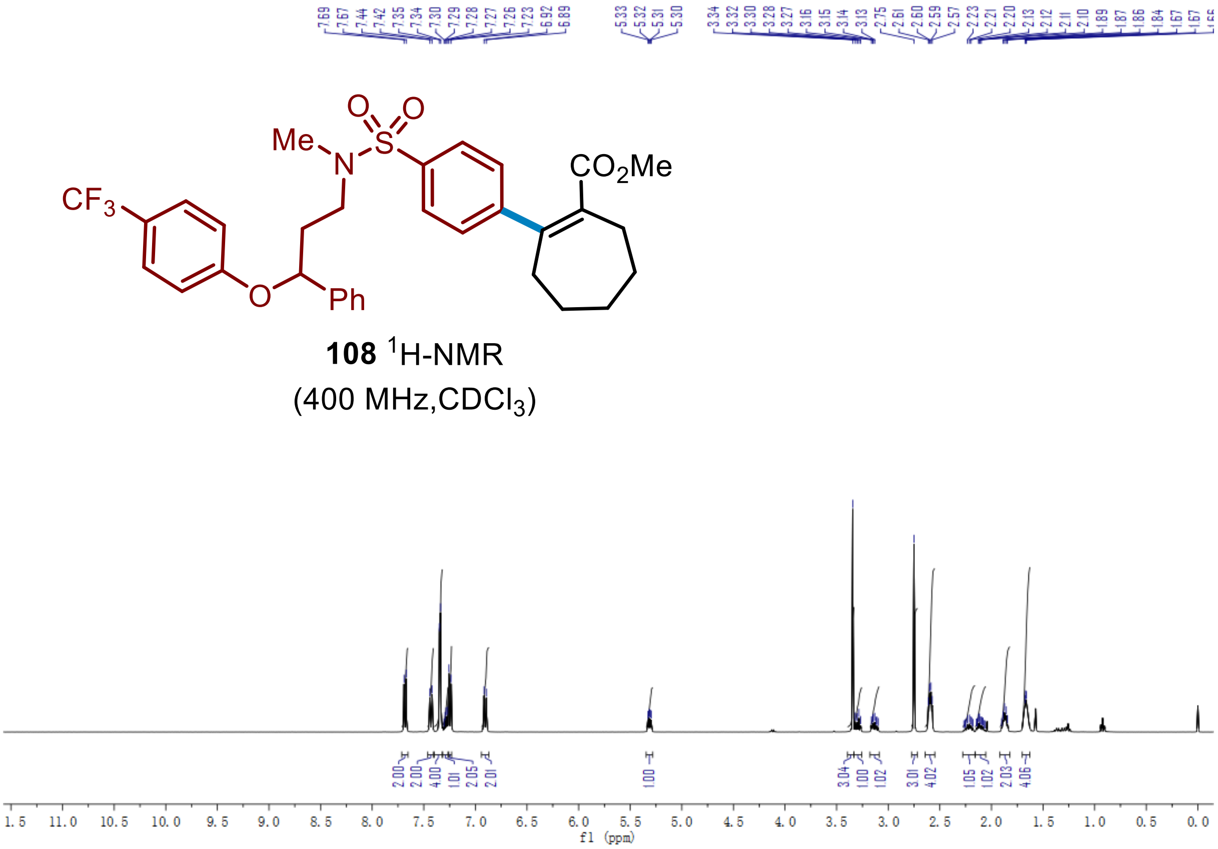

Supplementary Figure 310. **^{1}H-NMR** (400 MHz, $CDCl_3$, 298K) of **108**

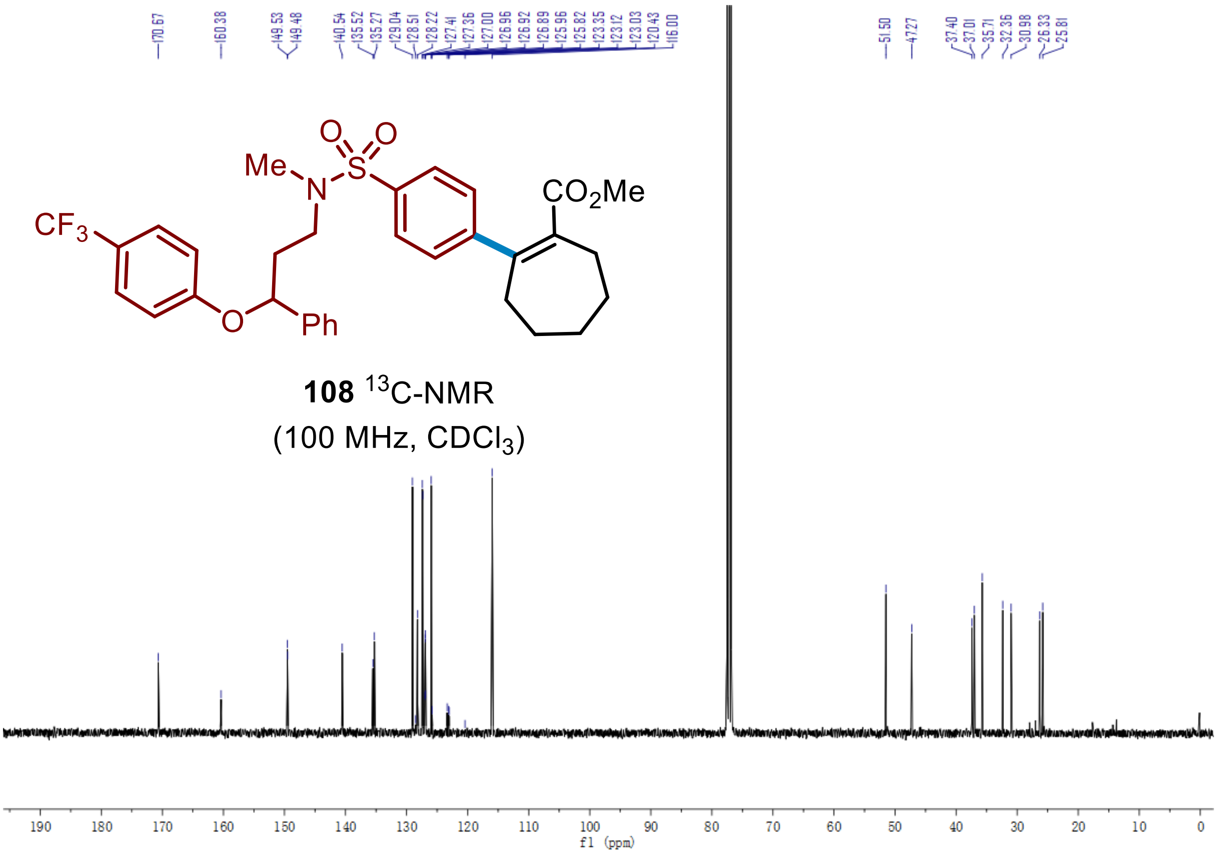

Supplementary Figure 311. **^{13}C-NMR** (100 MHz, $CDCl_3$, 298K) of **108**

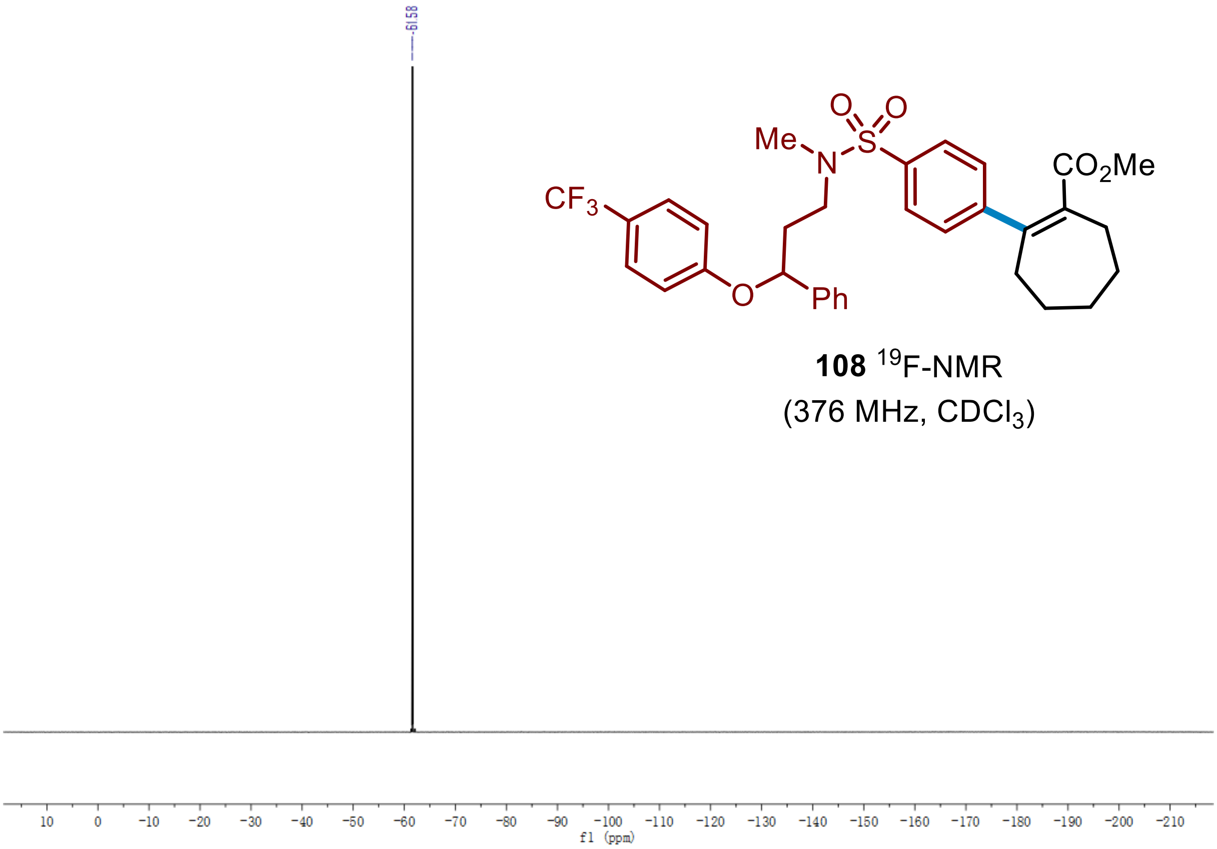

Supplementary Figure 312. **^{19}F-NMR** (376 MHz, $CDCl_3$, 298K) of **108**

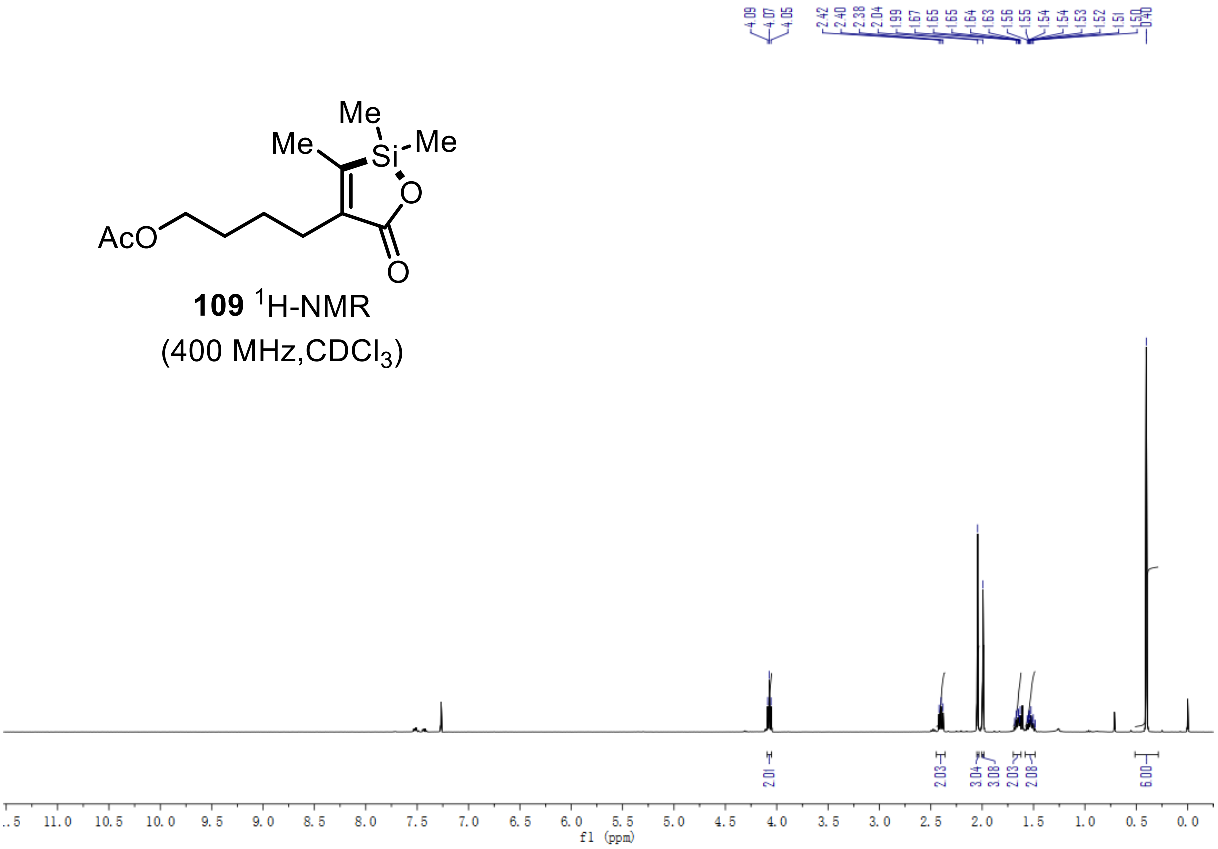

Supplementary Figure 313. **^{1}H-NMR** (400 MHz, $CDCl_3$, 298K) of **109**

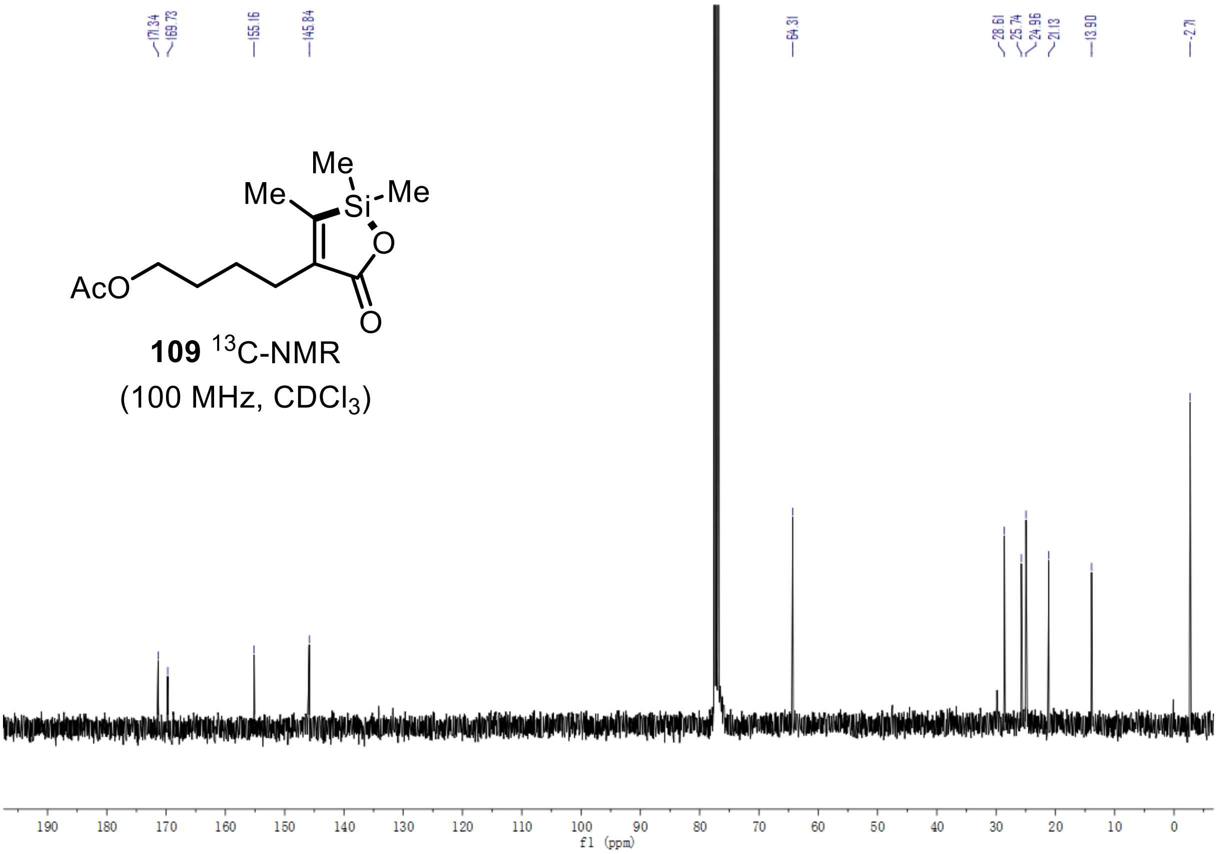

Supplementary Figure 314. **^{13}C-NMR** (100 MHz, $CDCl_3$, 298K) of **109**

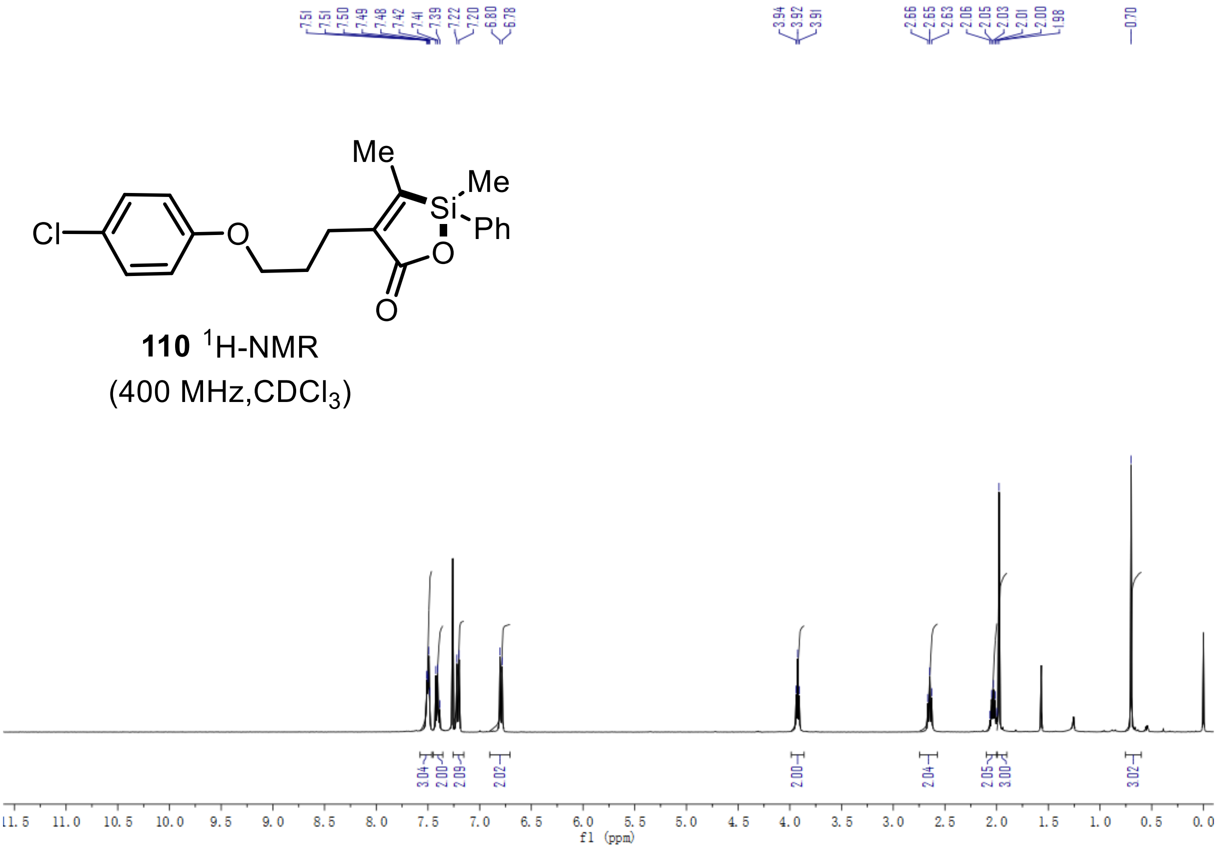

Supplementary Figure 315. ^{1}H-NMR (400 MHz, $CDCl_3$, 298K) of **110**

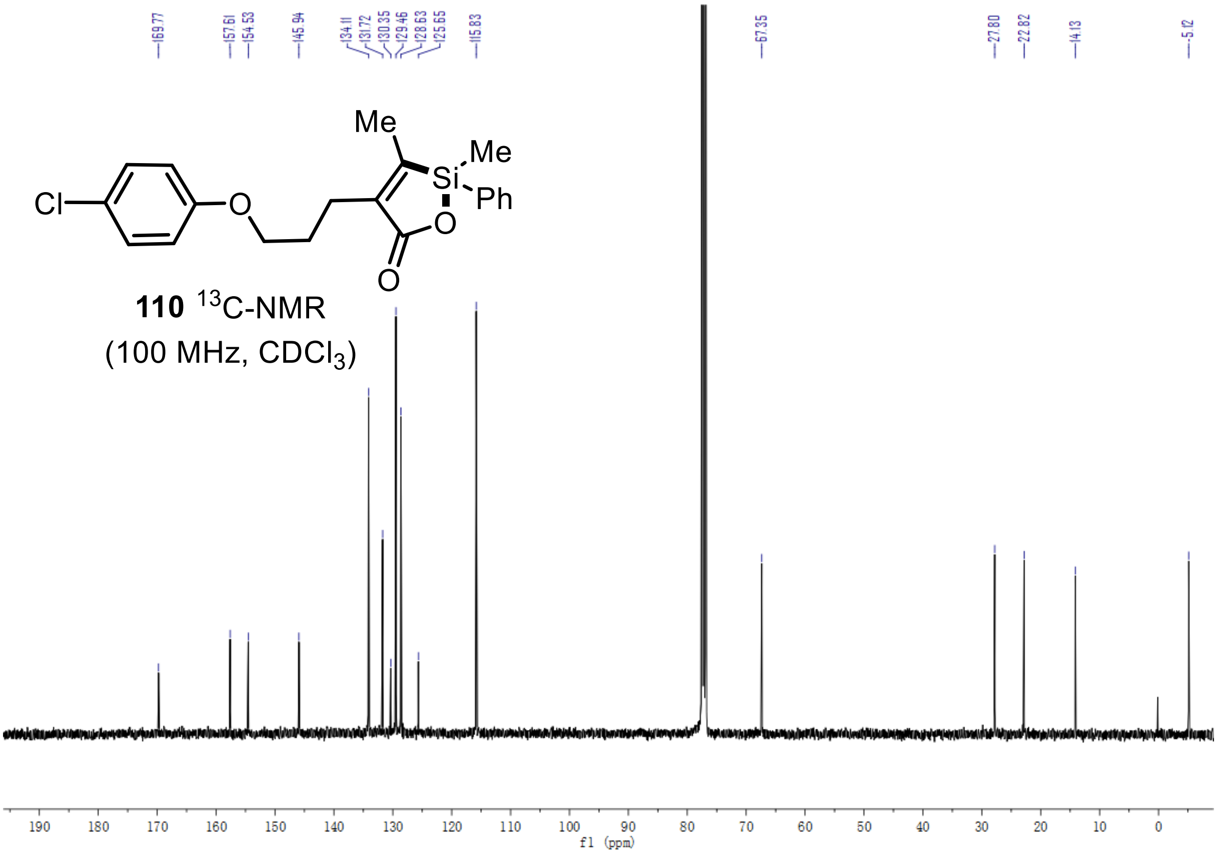

Supplementary Figure 316. **^{13}C-NMR** (100 MHz, $CDCl_3$, 298K) of **110**

Supplementary References:

[1] M. Dong, J. Wang, S. Wu, Y. Zhao, Y. Ma, Y. Xing, F. Cao, L. Li, Z. Li, H. Zhu, *Adv. Synth. Catal.* **2019**, *361*, 4602-4610.

[2] N. S. Y. Loy, S. Choi, S. Kim, C.-M. Park, *Chem. Commun.* **2016**, *52*, 7336-7339.

[3] L. A. Stubbing, F. F. Li, D. P. Furkert, V. E. Caprio, M. A. Brimble, *Tetrahedron.* **2012**, *68*, 6948-6956.

[4] G. Gu, J. Lu, O. Yu, J. Wen, Q. Yin, X. Zhang, *Org. Lett.* **2018**, *20*, 1888-1892.

[5] M. Hu, C. Ni, L. Li, Y. Han, J. Hu, *J. Am. Chem. Soc.* **2015**, *137*, 14496-14501.

[6] A. Bongers, C. Clavette, W. Gan, S. I. Gorelsky, L. Betit, K. Lavergne, T. Markiewicz, P. J. Moon, N. Das Neves, N. K. Obhi, A. B. Toderian, A. M. Beauchemin, *J. Org. Chem.* **2017**, *82*, 1175-1194.

[7] K. Chegaev, B. Rolando, D. Cortese, E. Gazzano, I. Buondonno, L. Lazzarato, M. Fanelli, C. M. Hattinger, M. Serra, C. Riganti, R. Fruttero, D. Ghigo, A. Gasco, *J. Med. Chem.* **2016**, *59*, 4881-4889.

[8] M. Kenny, J. Christensen, S. J. Coles, V. Franckevičius, *Org. Lett.* **2015**, *17*, 3926-3929.

[9] H. Zhang, X. Wu, Y. Wei, C. Zhu, *Org. Lett.* **2019**, *21*, 7568-7572.

[10] J. Li, Q. Ren, X. Cheng, K. Karaghiosoff, P. Knochel, *J. Am. Chem. Soc.* **2019**, *141*, 18127-18135.

[11] K. Gorlitzer, *Arch. Pharm. (Weinheim)* **1975**, *308*, 272-286.

[12] R.-D. He, C.-L. Li, Q.-Q. Pan, P. Guo, X.-Y. Liu, X.-Z. Shu, *J. Am. Chem. Soc.* **2019**, *141*, 12481-12486.

[13] F. Wang, W. Sun, Y. Wang, Y. Jiang, T.-P. Loh, *Org. Lett.* **2018**, *20*, 1256-1260.

[14] Y. Wang, L. Lu, D. J. Burton, *J. Org. Chem.* **2005**, *70*, 10743-10746.
